# Measurement properties of 72 movement biomarkers aiming to discriminate non‑specific chronic low back pain patients from an asymptomatic population

**DOI:** 10.1038/s41598-023-33504-5

**Published:** 2023-04-20

**Authors:** Florent Moissenet, Stéphane Armand, Stéphane Genevay

**Affiliations:** 1 Kinesiology Laboratory, Geneva University Hospitals, University of Geneva, Geneva, Switzerland; 2 Department of Rheumatology, Geneva University Hospitals, Geneva, Switzerland

**Keywords:** Diagnostic markers, Musculoskeletal system, Biomedical engineering

## Abstract

The identification of relevant and valid biomarkers to distinguish patients with non-specific chronic low back pain (NSCLBP) from an asymptomatic population in terms of musculoskeletal factors could contribute to patient follow-up and to evaluate therapeutic strategies. Several parameters related to movement impairments have been proposed in the literature in that respect. However, most of them were assessed in only one study, and only 8% were evaluated in terms of reliability, validity and interpretability. The aim of this study was to consolidate the current knowledge about movement biomarkers to discriminate NSCLBP patients from an asymptomatic population. For that, an experimental protocol was established to assess the reliability, validity and interpretability of a set of 72 movement biomarkers on 30 asymptomatic participants and 30 NSCLBP patients. Correlations between the biomarkers and common patient reported outcome measures were also analysed. Four biomarkers reached at least a good level in reliability (ICC ≥ 0.75) and validity (significant difference between asymptomatic participants and NSCLBP patients, *p* ≤ 0.01) domains and could thus be possibly considered as valuable biomarkers: maximal lumbar sagittal angle, lumbar sagittal angle range of motion, mean lumbar sagittal angular velocity, and maximal upper lumbar sagittal angle during trunk sagittal bending. These four biomarkers demonstrated typically larger values in asymptomatic participants than in NSCLBP patients. They are in general weakly correlated with patient reported outcome measures, arguing for a potential interest in including related musculoskeletal factors in the establishment of a valuable diagnosis and in guiding treatment response.

## Introduction

Non-specific chronic low back pain (NSCLBP) is a complex disorder where peripheral and central pain mechanisms are involved and influenced by various factors such as social, psychological or musculoskeletal factors interacting with each other^1,2^. To date, many studies have pointed out that social and psychological factors are involved in the persistence of the pain^3,4^. However, the role of musculoskeletal factors is still unclear and the benefit of neuromechanics in establishing a valuable diagnosis and in guiding treatment remains debated. To move forward, Chlolewicki et al.^5^, in a recent special issue on low back pain, emphasised the potential of several movement parameters to better understand the multifactorial biopsychosocial problem of low back pain. They also underlined the importance of additional research on quantitative biomarkers, including movement biomarkers, to support the development of more effective treatments of low back pain.

In line with this suggestion, a recent systematic review from our group identified 121 movement biomarkers with the potential to discriminate NSCLBP patients from an asymptomatic population^6^. For all these parameters, a thorough extraction of their description (e.g. variable of interest, related motor task) and measurement properties (or psychometric properties), i.e. reliability, validity, and interpretability, was performed. Amongst other findings, this systematic review highlighted the fact that (1) most of the reported potential biomarkers (90%) have been assessed in only one study, and (2) only 8% of them were evaluated in terms of reliability, validity and interpretability. Nevertheless, 31 potential movement biomarkers for which an extensive measurement properties assessment was already made available were identified, and 17 of them demonstrated good to excellent levels in terms of reliability and validity.

To be raised at the biomarker level, a parameter must give objective indications of patient state and must be measured accurately and reproducibly^7^. Hence, in view of the numerous parameters already proposed, the aim of this study was to consolidate the current knowledge about movement biomarkers rather than propose new parameters. For that, an experimental protocol was established so as to assess the reliability, validity and interpretability of a subset of 72 movement biomarkers. Material and temporal issues guided the choice of these 72 biomarkers among the 121 biomarkers highlighted in the previously published systematic review^6^. Reliability, validity and interpretability of these biomarkers were assessed, as well as their correlation with common patient reported outcome measures (PROMs).

## Methods

### Study design

This is a monocentric prospective study approved by the Cantonal Research Ethics Commission of Geneva (CER 14–126). The experimental procedure was based on a modified form of the protocol proposed by Rose-Dulcina et al.^8^ was developed in compliance with the 1964 Helsinki Declaration and later amendments. One operator recorded and pre-processed a set of kinematic parameters at each session for each participant. All participants were evaluated twice using the same protocol at one-week interval (± 0.0 week). This time interval follows McGinley et al. recommendations for reliability studies^9^, and avoids strong variations in pain. Based on original articles, 72 movement biomarkers were computed under Matlab (R2019b, The MathWorks, USA). To assess their reliability, validity and interpretability^10^, 30 asymptomatic participants and 30 NSCLBP patients were required to have a 90% chance of detecting, as significant at the 5% level, a change in a biomarker corresponding to an effect size of 0.8^11^. The large effect size was chosen because the biomarkers should be very strong predictors to distinguish NSLBP patients from asymptomatic participants.

### Participants

Asymptomatic participants and NSCLBP patients were recruited respectively from the Geneva area and at the outpatient clinics in the Department of Rheumatology and Department of Rehabilitation and Physical Medicine at Geneva University Hospitals between May 2020 and August 2021. The inclusion criteria of asymptomatic participants were as follows: aged between 18 and 60 years, no back pain for at least 6 months, and no motor dysfunction in the tasks required by the present protocol. Exclusion criteria were: pain in any part of the body, pregnancy, body mass index (BMI) over 32 kg. m^−2^, and inability to understand French. The inclusion criteria of patients were as follows: aged between 18 and 60 years, suffering from NSCLBP, at least 3 months duration of the current episode of pain^12^, average pain intensity over 3/10 on a visual analogue scale during the last week^13^. Exclusion criteria were: pain in other parts of the body (except leg pain radiation from the lower back), specific low back pathology such as infection, tumour, osteoporosis, fracture, structural deformity, inflammatory disorder, radicular syndrome or cauda equina syndrome^1^, plus the same exclusion criteria as mentioned for asymptomatic participants. All participants (i.e. asymptomatic participants and NSCLBP patients) included in our study provided written informed consent prior to their participation.

### Data collection

The psychosocial profile of NSCLBP patients was explored using patient-reported specific questionnaires to evaluate anxiety and depression (HADS)^14^, functional disability (ODI)^15^, pain catastrophising (PCS)^16^, avoidance belief (FABQ)^17^, in their French version. The French version of the Core Outcome Measure Index (COMI), a multidimensional questionnaire, was also used for this purpose^13^. The pain intensity over the last week was reported using a visual analogue scale^18^.

**Figure 1:**
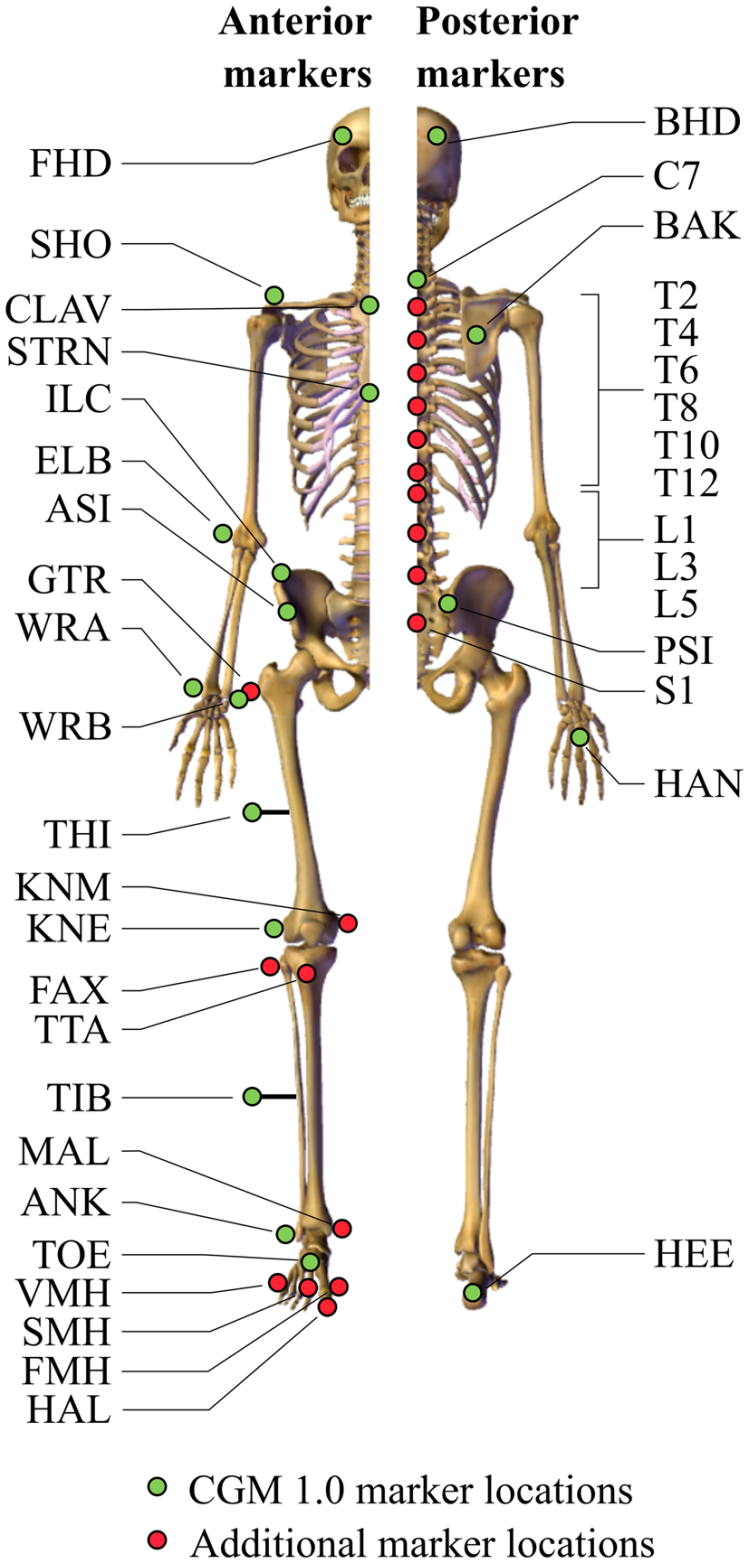
Placement of cutaneous reflective markers (arm and leg markers were equipped bilaterally. CGM: Conventional Gait Model^19^).

A 12-camera optoelectronic system sampled at 100 Hz (Oqus7+ , Qualisys, Sweden) was used to track the three-dimensional (3D) trajectories of a set of 64 cutaneous reflective markers (14 mm of diameter). The markerset (Fig. 1) was based on the full body Conventional Gait Model (CGM) 1.0^19^ and completed with additional markers on lower limbs and on a set of vertebrae spinous processes^20^. Marker placement was achieved by anatomical palpation following the guideline provided by Van Sint Jan^21^ and remained unchanged during the sessions. The same experienced operator performed both anatomical palpation and marker placement on all participants.

Once equipped, participants were asked to execute a set of 18 motor tasks after a static trial in T-pose. The task order remained the same across sessions and participants to ease the data collection process. Only a subset of 8 tasks is reported in the present study. This subset consists in (1) two-legged standing, (2) barefoot walking, (3) trunk forward bending, (4) bilateral trunk lateral bending, (5) bilateral trunk rotation, (6) weight lifting, (7) sitting and (8) sit-to-stand (available in Supplementary Table 2). Low back pain was assessed after each task to quantify the pain intensity generated by the task using a visual analogue scale.

### Data pre-processing

Labelling of 3D marker trajectories was performed in the Qualisys Tracking Manager software (QTM 2019.3, Qualisys, Sweden). Labelled marker trajectories were exported in the standard c3d file format (https://www.c3d.org) and then imported and processed under Matlab (R2019b, The MathWorks, USA) using the Biomechanics ToolKit (BTK)^22^. When gaps were no longer than 10% of the trial duration, markers trajectories were interpolated using a reconstruction based on marker inter-correlations obtained from a principal component analysis^23^. Otherwise, they were rigidly reconstructed in the least-squares sense based on the marker position during the static trial^24^. Then, trajectories were smoothed using a moving average approach over each window of 15 frames. Gait cycles were identified through automatic detection of foot strike and foot off events based on 3D marker trajectories^25^. Other movement cycles were identified through automatic detection of transitions (e.g. right to left, up to down) using a custom written Matlab code. All pre-processing Matlab codes are available on a GitLab repository (see Data Availability Statement section).

### Biomarkers selection and computation

In a recent systematic review of our group^6^, 121 movement biomarkers have already been identified in the literature. In particular, 17 biomarkers were highlighted as having been previously extensively assessed in terms of measurement properties, with at least good levels in explored domains (e.g. reliability, validity). Using the previously defined experimental procedure, a subset of 72 movement biomarkers was computable, including all of the 17 highlighted biomarkers. The complete list of the movement biomarkers explored in the present study is provided in Table 1.

**Table 1:** Characteristics of the movement biomarkers assessed in this study.

Biomarker ID*	Measured variable	Related motor task	Original article(s)
BMo1	Head anterior–posterior displacement (std)	Sit to stand	Tajali et al.^59^
BMo3	Hip sagittal angle (rom)	Sit to stand	Pourahmadi et al.^60^
BMo4	Hip sagittal angle (rom)	Stand to sit	Pourahmadi et al.^60^
*BMo5*	*Lower lumbar sagittal angle (max)*	*Trunk sagittal bending*	*Hidalgo et al.*^35^
BMo6	Lower lumbar sagittal angular velocity (max)	Trunk sagittal bending	Hidalgo et al.^35^
BMo7	Lower thorax curvature (max)	Sit to stand	Hemming et al.^61^
BMo8	Lower thorax curvature (max)	Stand to sit	Hemming et al.^61^
*BMo9*	*Lower thorax sagittal angle (max)*	*Trunk sagittal bending*	*Hidalgo et al.*^35^
BMo10	Lumbar contribution to thorax angle (rom)	Trunk sagittal bending	Laird et al.^58^
**BMo12**	**Lumbar sagittal angle (max)**	**Trunk sagittal bending**	**Hidalgo **et al.^35^; **Hidalgo **et al.^62^
BMo14	Lumbar transversal angle (rom)	Trunk rotation	Vaisy et al.^63^
***BMo15***	***Lumbar sagittal angle (rom)***	***Trunk sagittal bending***	***Vaisy *et al**.^63^
BMo17	Lumbar sagittal angular velocity (max)	Trunk sagittal bending	Hidalgo et al.^35^
***BMo18***	***Lumbar sagittal angular velocity (mean)***	***Trunk sagittal bending***	***Vaisy *et al*.***^63^
BMo23	Lumbar/hip ratio of sagittal angle (rom)	Sit to stand	Pourahmadi et al.^60^
BMo24	Lumbar/hip ratio of sagittal angle (rom)	Stand to sit	Pourahmadi et al.^60^
BMo25	Lumbar/hip relative phase difference (max)	Sit to stand	Pourahmadi et al.^60^
BMo26	Lumbar/hip relative phase difference (mean)	Sit to stand	Pourahmadi et al.^60^
BMo27	Lumbar/hip relative phase difference (mean)	Stand to sit	Pourahmadi et al.^60^
BMo28	Lumbar/hip relative phase difference (min)	Sit to stand	Pourahmadi et al.^60^
BMo29	Lumbar/pelvis absolute relative phase (mean)	Trunk sagittal bending	Mokhtarinia et al.^64^
BMo30	Lumbar/pelvis deviation phase (mean)	Trunk sagittal bending	Mokhtarinia et al.^64^
BMo31	Lumbopelvic sagittal angle (max)	Sit to stand	Christe et al.^65^
BMo33	Lumbopelvic sagittal angle (rom)	Sit to stand	Pourahmadi et al.^60^
BMo34	Lumbopelvic sagittal angle (rom)	Stand to sit	Pourahmadi et al.^60^
BMo35	Lumbopelvic sagittal angular velocity (max)	Sit to stand	Christe et al.^65^
*BMo37*	*Pelvis sagittal angle (rom)*	*Trunk sagittal bending*	*Neblett et al.*^66^
BMo38	Pelvis sagittal angle (rom)	Trunk rotation	Taniguchi et al.^67^
BMo41	Pelvis sagittal angular velocity (mean)	Trunk sagittal bending	Vaisy et al.^63^
BMo42	Pelvis/thigh deviation phase (mean)	Trunk sagittal bending	Mokhtarinia et al.^64^
*BMo43*	*Scapular belt transversal angle (max)*	*Trunk rotation*	*Hidalgo et al.*^35^
BMo44	Thoracopelvic sagittal angle (max)	Trunk sagittal bending	Ahern et al.^68^; Larivière et al.^69^; Neblett et al.^66^
BMo45	Thoracopelvic sagittal angle (rom)	Trunk rotation	Taniguchi et al.^67^
BMo46	Thorax frontal angle (rom)	Trunk rotation	Taniguchi et al.^67^
BMo47	Thorax frontal angle (rom)	Trunk sagittal bending	Bourigua et al.^29^
*BMo48*	*Thorax frontal angular velocity (max)*	*Trunk lateral bending*	*Bourigua et al.*^29^
*BMo49*	*Thorax sagittal angle (rom)*	*Trunk sagittal bending*	*Neblett et al.*^66^
BMo50	Thorax sagittal angle (rom)	Trunk lateral bending	Vaisy et al.^63^
BMo51	Thorax sagittal angle (rom)	Trunk rotation	Taniguchi et al.^67^
BMo53	Thorax sagittal angular velocity (max)	Trunk sagittal bending	Bourigua et al.^29^
BMo54	Thorax transversal angle (rom)	Trunk rotation	Bourigua et al.^29^
BMo55	Thorax transversal angular velocity (max)	Trunk rotation	Bourigua et al.^29^
**BMo57**	**Upper lumbar sagittal angle (max)**	**Trunk sagittal bending**	**Hidalgo **et al*.*^35^
BMo58	Upper lumbar sagittal angular velocity (max)	Trunk sagittal bending	Hidalgo et al.^35^
*BMo59*	*Upper thorax sagittal angle (max)*	*Trunk sagittal bending*	*Hidalgo et al.*^35^
BMo60	Upper/lower lumbar sagittal angle (max)	Sit to stand	Christe et al.^65^
BMo61	Upper/lower lumbar sagittal angular velocity (max)	Sit to stand	Christe et al.^65^
BMo62	Upper/lower thorax sagittal angle (max)	Sit to stand	Christe et al.^65^
BMo63	Upper/lower thorax sagittal angular velocity (max)	Sit to stand	Christe et al.^65^
BMo72	Lumbopelvic sagittal angle (max)	Sitting	Dankaerts et al.^70^
*BMo75*	*Thorax sagittal angle (max)*	*Two-legged standing*	*Bourigua et al.*^29^
BMo77	Hip sagittal angle (min)	Trunk sagittal bending	Falla et al.^71^
BMo79	Hip/knee deviation phase (mean)	Trunk sagittal bending	Pranata et al.^72^
BMo80	Lumbopelvic sagittal angle (max)	Trunk sagittal bending	Matheve et al.^73^
*BMo81*	*Thoracolumbar sagittal angle (max)*	*Trunk sagittal bending*	*Falla et al.*^71^
BMo82	Thorax linear acceleration (max)	Trunk sagittal bending	Larivière et al.^74^
BMo83	Thorax linear velocity (max)	Trunk sagittal bending	Larivière et al.^74^
BMo84	Upper lumbar curvature (max)	Trunk sagittal bending	Hemming et al.^61^
BMo85	Thorax angular acceleration (max)	Trunk sagittal bending	Larivière et al.^74^
BMo86	Thorax angular velocity (max)	Trunk sagittal bending	Larivière et al.^74^
BMo87	Lumbar/pelvis frontal angle (coefficient of variation)	Walking	Vogt et al.^75^
BMo89	Lumbar/pelvis sagittal angle (coefficient of variation)	Walking	Vogt et al.^75^
BMo91	Lumbar/pelvis transversal angle (coefficient of variation)	Walking	Vogt et al.^75^
BMo98	Lumbopelvic frontal angle (rom)	Walking	Christe et al.^76^
BMo99	Pelvis frontal angle (coefficient of variation)	Walking	Vogt et al.^75^
BMo101	Pelvis sagittal angle (coefficient of variation)	Walking	Vogt et al.^75^
BMo103	Pelvis transversal angle (coefficient of variation)	Walking	Vogt et al.^75^
BMo105	Pelvis/thigh sagittal deviation phase during stance (mean)	Walking	Ebrahimi et al.^77^
BMo106	Pelvis/thigh sagittal deviation phase during swing (mean)	Walking	Ebrahimi et al.^77^
BMo107	Shank/foot sagittal relative phase during swing (mean)	Walking	Ebrahimi et al.^77^
BMo108	Thoracolumbar transversal angle (max)	Walking	Christe et al.^76^
BMo112	Thorax/pelvis sagittal deviation phase during stance (mean)	Walking	Ebrahimi et al.^77^
BMo113	Thorax/pelvis sagittal deviation phase during swing (mean)	Walking	Ebrahimi et al.^77^
BMo114	Thorax/pelvis sagittal relative phase during stance (mean)	Walking	Ebrahimi et al.^77^
BMo115	Thorax/pelvis sagittal relative phase during swing (mean)	Walking	Ebrahimi et al.^77^
BMo118	Thigh/shank sagittal relative phase during swing (mean)	Walking	Ebrahimi et al.^77^

*Biomarker identifiers (ID) used in the systematic review of Moissenet et al.^6^. Biomarkers having reached at least good levels in the reliability domain are highlighted in italic, in reliability and validity in bold italic, and in reliability, validity and interpretability in bold (see Table 2 for measurement properties rating).

The selected biomarkers were computed under Matlab while reproducing the methodology reported in the related original article (Table 1). All Matlab codes are available on a GitLab repository (see Data Availability Statement section). For homogenisation purposes, the same kinematic computation pipeline was applied to each biomarker. The definition of joint centres and segment coordinate systems proposed by Dumas and Wojtusch^26^ were used and followed the recommendations of the International Society of Biomechanics (ISB)^27^. Joint kinematics was computed using the 3D Kinematics and Inverse Dynamics toolbox proposed by Dumas and freely available on the MathWorks File Exchange^28^. For bilateral trunk lateral bending and trunk rotation tasks, previous studies^29,30^ did not report asymmetry in the related range of motion. Hence, only the values related to the right-side trunk lateral bending and trunk rotation were analysed.

### Statistical analysis

For each biomarker listed in Table 1, the measurement properties were assessed according to the Consensus-Based Standards for the Selection of Health Measurement Instruments (COSMIN) checklist^10^. Only reliability, validity and interpretability domains were explored in this study. All computations related to the statistical analyses were performed in R 4.1.2 and RStudio (version 2021.09.0 build 351)^31^.

### Reliability

Test–retest reliability (i.e. intra-rater between-session reliability) and intra-rater reliability (i.e. intra-rater within-session reliability) were assessed for each biomarker of each group using an intra-class correlation^10^, respectively ${ICC}_{test-retest}$ and ${ICC}_{intra-rater}$.

Variance components were computed first from a single measure, two-way mixed effects model with the *lme4* package (1.1–28)^32^. The total variance was computed as the sum of the variance of class components:

1 $$\begin{array}{*{20}c} {\sigma_{total}^{2} = \sigma_{participant}^{2} + \sigma_{session}^{2} + \sigma_{cycle}^{2} + \sigma_{residual}^{2} } \\ \end{array}$$

where ${\sigma }_{participant}^{2}$, ${\sigma }_{session}^{2}$, ${\sigma }_{cycle}^{2}$ and ${\sigma }_{residual}^{2}$ are the participant, session, cycle and residual variance, respectively. Following the methodology proposed by Chia and Sangeux^33^, ICC estimates were then obtained as follow:

2 $$\begin{array}{*{20}c} {ICC_{test - retest} = \frac{{\sigma_{total}^{2} - \left( {\sigma_{session}^{2} + \sigma_{residual}^{2} } \right)}}{{\sigma_{total}^{2} }}} \\ \end{array}$$

3 $$\begin{array}{*{20}c} {ICC_{intra - rater} = \frac{{\sigma_{total}^{2} - \left( {\sigma_{cycle}^{2} + \sigma_{residual}^{2} } \right)}}{{\sigma_{total}^{2} }}} \\ \end{array}$$

ICC estimates were classified as poor (< 0.5), moderate (0.5 to 0.75), good (0.75 to 0.90), and excellent (≥ 0.90)^34^. In order to ensure correct interpretations, ICC estimates were completed with the standard error of measurement (SEM) computed as follow:

4 $$\begin{array}{*{20}c} {SEM_{test - retest} = \sqrt {\sigma_{total}^{2} \times \left( {1 - ICC_{test - retest} } \right)} } \\ \end{array}$$

5 $$\begin{array}{*{20}c} {SEM_{intra - rater} = \sqrt {\sigma_{total}^{2} \times \left( {1 - ICC_{intra - rater} } \right)} } \\ \end{array}$$

As each SEM value is related to a biomarker with a specific range and unit of measurement, SEM% was also computed^35^. SEM% estimates were classified as poor (> 50%), moderate (33% to 50%), good (16.5% to 33%), and excellent (≤ 16.5%) as no clear criteria for SEM% were found available in the literature. To be recognised as a suitable biomarker, the later had to reach at least good levels in patient test–retest reliability and related standard error of measurement (%).

### Validity

As goal standards were not available for the selected biomarkers^6^, criterion validity could not be reported. Construct validity was assessed instead for each biomarker based on the first session records. This validation was achieved by testing the hypothesis that a biomarker produced a statistically significant difference (higher or lower, depending on the biomarker) between asymptomatic participants and NSCLBP patients^10^.

For each biomarker, a Shapiro–Wilk normality test was performed first with the *rstatix* package (0.7.0)^36^ to assess the normality of the datasets. Biomarkers having demonstrated a normal distribution were then assessed using a Student’s t-test with a 95% confidence level (α = 0.05), others were assessed using a Mann–Whitney U-test, both using the *stats* package (3.6.2)^37,38^. *P* value estimates were classified as poor (> 0.05), moderate (0.01 to 0.05), good (0.001 to 0.01), and excellent (≤ 0.001).

Furthermore, sensitivity and specificity of each biomarker were reported using a receiver operating characteristic curve (ROC curve). Area under the ROC curve (*AUC*) and Youden index (i.e. the value providing the best trade-off between sensitivity and specificity) were also computed. The *pROC* package (1.18.0) was used for this purpose^39^. AUC estimates were classified as poor (< 60%), moderate (60% to 70%), good (70% to 80%), and excellent (≥ 80%)^40^.

### Interpretability

In order to assess if a difference between two measurements of the same biomarker can be considered as a true change rather than a measurement error, the minimal detectable change at a specified confidence interval of 95% (MDC_95_) was reported for each biomarker of each group^10^.

MDC_95_ was computed both in the context of intra-rater between-session and intra-rater within-session, for both groups:

6 $$\begin{array}{*{20}c} {MDC_{test - retest} = 1.96 \times \sqrt 2 \times SEM_{test - retest} } \\ \end{array}$$

7 $$\begin{array}{*{20}c} {MDC_{intra - rater} = 1.96 \times \sqrt 2 \times SEM_{intra - rater} } \\ \end{array}$$

As each MDC_95_ value is related to a biomarker with a specific range and unit of measurement, MDC% was also computed^35^. MDC% estimates were classified as poor (> 50%), moderate (33% to 50%), good (16.5% to 33%), and excellent (≤ 16.5%) as no clear criteria for MDC% were found available in the literature.

### Identification of the most suitable movement biomarkers

To ease the identification of the most suitable biomarkers, a subset of measurement properties was highlighted using a Circos plot^41^: *ICC* test–retest patient, *SEM*% test–retest patient, *p* value, *AUC*, *MDC* test–retest patient. Measurement properties rating is reported in Table 2. For each biomarker, the primary characteristics, i.e. corresponding ICF 2nd level category, variable category, and region of interest, were also reported on this plot.

**Table 2:** Rating used for each measurement property evaluated.

Rating	ICC^34^	SEM (%)	*p* value	AUC^40^ (%)	MDC (%)
Excellent	≥ 0.90	≤ 16.5	≤ 0.001	≥ 80	≤ 16.5
Good	< 0.90	> 16.5	> 0.001	< 80	> 16.5
	≥ 0.75	≤ 33	≤ 0.010	≥ 70	≤ 33
Moderate	< 0.75	> 33	> 0.01	< 70	> 33
	≥ 0.50	≤ 50	≤ 0.05	≥ 60	≤ 50
Poor	< 0.50	> 50	> 0.05	< 60	> 50

*ICC* intra-class correlation, *SEM* standard error of measurement, *AUC* area under curve, *MDC* minimal detectable change.

### Correlation between biomarker values and PROMs

Correlations were expressed for the most suitable biomarkers having demonstrated good to excellent levels in reliability and validity domains. The relationship between the mean value across NSCLBP patients at the initial session of these biomarkers and patient reported outcome measures (PROMs) was assessed by computing the matrix of Pearson’s r correlation coefficients with the *Hmisc* package (4.6–0)^42^. Correlations between biomarkers were also estimated. Correlations were classified as no relationship (r < 0.25, underline), weak relationship (0.25 ≤ r < 0.50, bolditalic), moderate relationship (0.50 ≤ r < 0.75, italic), and strong relationship (0.75 ≥ r, bold).

## Results

### Participants

**Figure 2:**
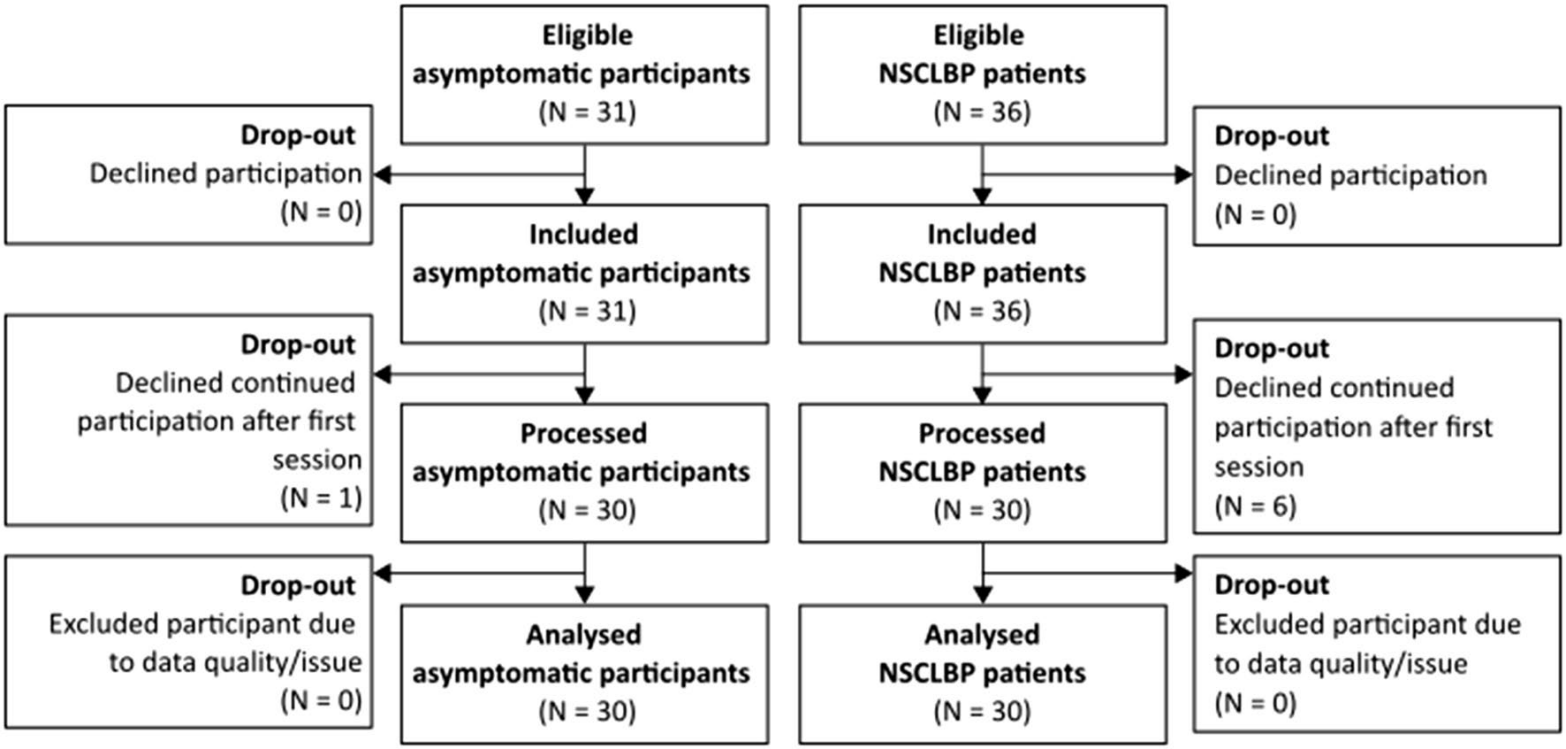
Participant flow diagram.

Thirty asymptomatic participants and 30 NSCLBP patients were recruited in this study (respectively 1 and 6 drop-outs due to a decline in continued participation after the first session, see Fig. 2 for participant flow diagram). Detailed participant information is reported in Table 3. The groups of asymptomatic participants and NSCLBP patients showed no significant difference in terms of age, height, body mass and BMI (Table 3).

**Table 3:** Participants characteristics.

	Asymptomatic participants	NSCLBP patients	*p* value*	Mean difference [95% CI]
N	30 (11F, 19 M)	30 (11F, 19 M)	–	–
Age (years)	36.2 (11.7)	41.1 (11.3)	0.10	− 4.9 [− 10.9 to 1.0]
Height (m)	1.73 (0.09)	1.74 (0.07)	0.38	− 0.02 [− 0.06 to 0.02]
Body mass (kg)	67.0 (10.3)	70.6 (12.6)	0.22	− 3.7 [− 9.6 to 2.3]
BMI (kg.m^-2^)	22.5 (2.7)	23.2 (3.5)	0.38	− 0.8 [− 9.6 to 2.3]
HADS-A (range 0–21)	–	7.15 (3.86)	–	–
HADS-D (range 0–21)	–	3.96 (3.54)	–	–
ODI (%)	–	35.63 (31.49)	–	–
PCS (range 0–52)	–	15.67 (11.08)	–	–
FABQ/pa (range 0–24)	–	8.06 (7.21)	–	–
FABQ/w (range 0–42)	–	9.54 (13.37)	–	–
COMI (range 0–10)	–	3.20 (2.76)	–	–
VAS pain (range 0–10)	–	3.92 (1.72)	–	–

*t test for independent samples (bold characters: *p* < 0.05).

Data reported as mean (std). CI confidence interval, NSCLBP non‑specific chronic low back pain, HADS hospital anxiety and depression scale, HADS-A HADS subscale related to anxiety, HADS-D HADS subscale related to depression, ODI oswestry disability index, PCS pain catastrophizing scale, FABQ fear avoidance belief questionnaire, FABQ/pa FAQB subscale related to physical activities. FABQ/w FABQ subscale related to work-related items. COMI core outcome measure index, VAS visual analog scale of pain (mean clinical pain over the past week).

### Identification of the most suitable movement biomarkers

**Figure 3:**
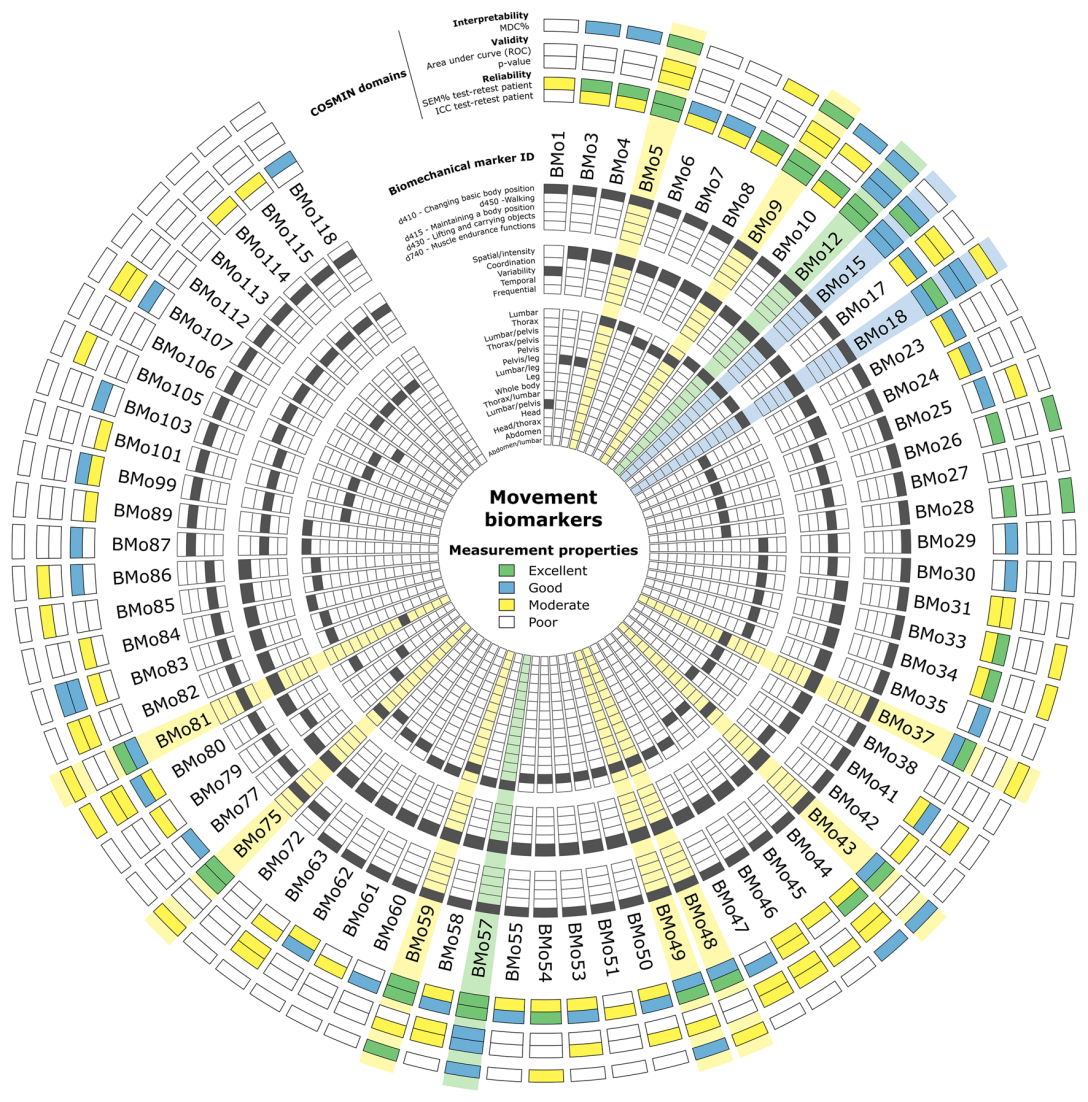
Circos plot^41^ synthesising the main characteristics and measurement properties of each movement biomarker. Biomarkers having reached at least good levels in the reliability domain are highlighted in yellow, in reliability and validity in blue, and in reliability, validity and interpretability in green.

Of the 72 assessed biomarkers, only 13 biomarkers (18%) reached at least a good level in the reliability domain, 4 biomarkers (6%) reached at least a good level in the reliability and validity domains, and 2 biomarkers (3%) reached at least a good level in reliability, validity and interpretability domains (Fig. 3). These 13 biomarkers were: maximal lower lumbar sagittal angle (BMo5), maximal lower thorax sagittal angle (BMo9), maximal lumbar sagittal angle (BMo12), lumbar sagittal angle range of motion (BMo15), mean lumbar sagittal angular velocity (BMo18), pelvis sagittal angle range of motion (BMo37), maximal scapular belt transversal angle (BMo43), maximal thorax frontal angular velocity (BMo48), thorax sagittal angle range of motion (BMo49), maximal upper lumbar sagittal angle (BMo57), maximal upper thorax sagittal angle (BMo59), maximal thorax sagittal angle (BMo75), and maximal thoracolumbar sagittal angle (BMo81) during trunk sagittal bending.

Eleven (85%) of these 13 most suitable biomarkers were related to the ICF 2nd level category^43^ d410 “Changing basic body position”, while the two others were related to d415 “Maintaining a body position” and d430 “Lifting and carrying objects”. All of these biomarkers were related to a spatial/intensity variable (i.e. angular value or angular velocity) and to the spine/pelvis region (thorax: 46%, lumbar: 38%, thorax/lumbar: 8%, pelvis: 8%). It must be noticed that all of the 4 biomarkers having reached at least a good level in reliability and validity domains were related to the lumbar sagittal angle (i.e. range of motion, maximum amplitude and velocity) during the trunk forward bending task.

### Measurement properties of the most suitable movement biomarkers

Measurement properties of these 13 biomarkers are reported in Table 4. Measurement properties, as well as boxplots and receiver operating characteristic (ROC) curves of all of the 72 assessed biomarkers, are available in Supplementary Table 1 and Supplementary Data 1.

**Table 4:** Measurement properties of the most suitable biomarkers.

Biomarker ID*	Units	Reliability									Validity			Interpret
		Intra–rater				Test–retest					–			Test–retest
		ICC		SEM		ICC		SEM			p	AUC	Youden	MDC_95_
		A	P	A	P	A	P	A		P	A vs. P	A vs. P	A vs. P	P
*BMo5*	*°*	*0.96*	*0.97*	*2.8*	*3.0*	*0.90*	*0.96*	*4.7*		*3.8*	* ≤ 0.05*	*0.69*	*0.33*	*10.5*
*BMo9*	*°*	*0.96*	*0.96*	*3.6*	*4.1*	*0.91*	*0.93*	*5.2*		*5.5*	* ≤ 0.05*	*0.68*	*0.30*	*15.3*
**BMo12**	**°**	**0.96**	**0.98**	**3.7**	**3.5**	**0.85**	**0.92**	**7.0**		**6.1**	≤ **0.01**	**0.73**	**0.40**	**16.9**
***BMo15***	***°***	***0.98***	***0.97***	***2.0***	***1.7***	***0.71***	***0.77***	***7.0***		***5.0***	** ≤ *0.001***	***0.77***	***0.43***	***13.9***
***BMo18***	***° s***^***−1***^	***0.69***	***0.85***	***2.4***	***1.5***	***0.82***	***0.84***	***1.8***		***1.6***	** ≤ *0.01***	***0.70***	***0.40***	***4.4***
*BMo37*	*° s*^**−1**^	*0.96*	*0.95*	*2.6*	*4.0*	*0.67*	*0.77*	*7.8*		*8.6*	*0.376*	*0.58*	*0.20*	*23.8*
*BMo43*	*°*	*0.77*	*0.92*	*5.0*	*3.1*	*0.62*	*0.86*	*6.3*		*4.0*	*0.151*	*0.59*	*0.20*	*11.2*
*BMo48*	*° s*^**−1**^	*0.70*	*0.85*	*5.9*	*4.1*	*0.66*	*0.78*	*6.3*		*5.0*	*0.258*	*0.58*	*0.19*	*13.8*
*BMo49*	*°*	*0.95*	*0.96*	*3.9*	*4.5*	*0.87*	*0.89*	*6.0*		*7.4*	*0.067*	*0.65*	*0.37*	*20.5*
**BMo57**	**°**	**0.94**	**0.96**	**3.4**	**4.0**	**0.90**	**0.95**	**4.7**		**4.9**	≤ **0.01**	**0.72**	**0.40**	**13.6**
*BMo59*	*°*	*0.96*	*0.95*	*3.8*	*4.1*	*0.94*	*0.91*	*4.7*		*5.5*	*0.058*	*0.69*	*0.43*	*15.2*
*BMo75*	*°*	*0.97*	*0.98*	*0.5*	*0.6*	*0.81*	*0.91*	*1.5*		*1.2*	*0.323*	*0.58*	*0.20*	*3.3*
*BMo81*	*°*	*0.94*	*0.98*	*2.9*	*1.7*	*0.67*	*0.86*	*6.6*		*4.4*	*0.791*	*0.52*	*0.17*	*12.2*

*Biomarker identifiers (ID) used in the systematic review of Moissenet et al.^6^. See Table 1 for the characteristics.

Biomarkers having reached at least good levels in the reliability domain are highlighted in italic, in reliability and validity in bold italic, and in reliability, validity and interpretability in bold (see Table 2 for measurement properties rating): ICC test–retest patient, SEM test–retest patient, *p* value, AUC, MDC test–retest patient (items used for the identification of the most suitable movement biomarkers using the Circos plot, Fig. 3).

*A* asymptomatic participants, *P* patients, *ICC* intra-class correlation, *SEM* standard error of measurement, *p* p value (Student t-test or Mann–Whitney U-test), *AUC* area under curve of the ROC (Receiver Operating Characteristic) curve, *Youden* Youden index of the ROC curve, *MDC* minimal detectable change (test–retest reliability).

Concerning reliability, these 13 biomarkers demonstrated moderate to excellent test–retest reliability (intra-rater between-session) in asymptomatic participants (intra-class correlation ICC = 0.62–0.94) and good to excellent test–retest reliability in NSCLBP patients (ICC = 0.77–0.96). Test–retest standard error of measurement (SEM) of angular values ranged from 1.5 to 7.0° for asymptomatic participants and from 1.2 to 7.4° for NSCLBP patients. Test–retest SEM of angular velocities ranged from 1.8 to 7.8° s^−1^ for asymptomatic participants and from 1.6 to 8.6° s^−1^ for NSCLBP patients. In all cases, intra-rater within-session reliability (intra-rater) was higher than intra-rater between-session reliability (test–retest).

**Figure 4:**
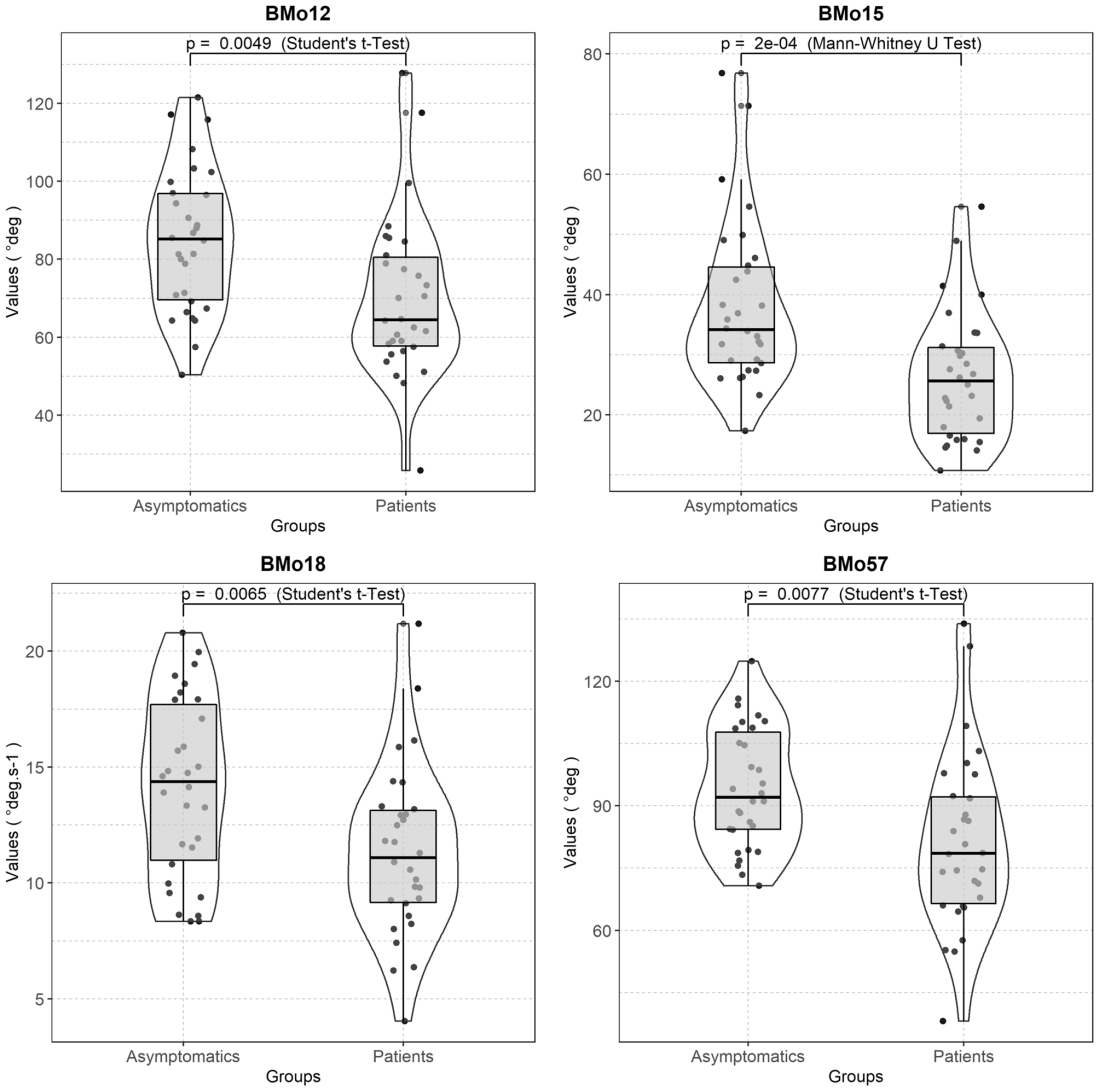
Boxplots summarising the values distribution measured on asymptomatic participants and NSLBP patients for the biomarkers having reached at least good levels in the reliabililty and validity domains.

Concerning validity, only 4 biomarkers among these 13 biomarkers demonstrated at least a good construct validity between asymptomatic participants and NSCLBP patients. Three biomarkers (i.e. BMo12, BMo18, and BMo57) demonstrated a good construct validity (*p* ≤ 0.01) with values typically larger in asymptomatic participants than in NSCLBP patients (BMo12: 84.9 ± 18.3 vs. 70.1 ± 20.7 deg, BMo18: 14.1 ± 3.8 vs. 11.4 ± 3.7 deg, BMo57: 94.2 ± 14.5 vs. 81.3 ± 21.0 deg). Only 1 biomarker (i.e. BMo15) demonstrated an excellent construct validity (p ≤ 0.001) with values typically larger in asymptomatic participants than in NSCLBP patients (BMo15: 38.2 ± 13.7 vs. 26.3 ± 10.7 deg). Boxplots are reported in Fig. 4 to support these results. These 4 biomarkers showed an area under the ROC curve (AUC) ranging from 0.70 to 0.77, with a Youden index ranging from 0.40 to 0.43.

Concerning interpretability, test–retest minimal detectable changes at a specified confidence interval of 95% (MDC_95_) of angular values ranged from 3.3 to 20.5° for NSCLBP patients. Test–retest SEM of angular velocities were ranged from 4.4 to 23.8° s^−1^ for NSCLBP patients.

### Correlation between biomarkers values and PROMs

Overall, biomarkers showed moderate to strong positive relationships between them (Table 5). Almost only negative relationships were obtained between biomarkers and PROMs. ODI and the physical activities (pa) subscale of the FABQ were the most correlated PROMs with biomarkers. Only the relationship between BMo57 and FABQ/pa was moderate and strong.

**Table 5:** Correlations between biomarkers values and PROMs.

Biomarker ID*	BMo12	BMo15	BMo18	BMo57	BMo59	HADS-A	HADS-D	ODI	PCS	FABQ/pa	FABQ/w	COMI	VAS
BMo12	**1**	***0.66***	**0.84**	**0.95**	**0.87**	0.02	− 0.16	* − 0.27*	− 0.11	* − 0.41*	− 0.10	− 0.03	− 0.19
BMo15		**1**	***0.71***	***0.54***	*0.45*	*0.26*	* − 0.29*	− 0.16	0.00	*− 0.25*	− 0.16	− 0.05	− 0.02
BMo18			**1**	**0.82**	**0.78**	0.13	* − 0.32*	* − 0.35*	− 0.18	* − 0.44*	− 0.20	− 0.24	− 0.10
BMo57				**1**	**0.95**	− 0.08	− 0.19	* − 0.36*	− 0.18	*** − 0.52***	− 0.04	− 0.14	* − 0.25*

*Biomarker identifiers (ID) used in the systematic review of Moissenet et al.^6^. See Table 1 for the characteristics.

Data reported as r value (Pearson correlation coefficient). *PROMs* patient reported outcome measures, *HADS* hospital anxiety and depression scale, *HADS-A* HADS subscale related to anxiety, *HADS-D* HADS subscale related to depression, *ODI* oswestry disability index, *PCS* pain catastrophizing scale. *FABQ* fear avoidance belief questionnaire. *FABQ/pa* FAQB subscale related to physical activities. *FABQ/w* FABQ subscale related to work-related items, *COMI* core outcome measure index, *VAS* visual analog scale of pain (mean clinical pain over the past week). Correlations were classified as no relationship (r < 0.25), weak relationship (0.25 ≤ r < 0.50, italic), moderate relationship (0.50 ≤ r < 0.75, bold italic), and strong relationship (0.75 ≥ r, bold).

## Discussion

A recent systematic review allowed us to identify a set of 121 potential movement biomarkers to discriminate NSCLBP patients from an asymptomatic population^6^. It highlighted the fact that most of the findings related to the proposed biomarkers need to be consolidated by completing the assessment of their measurement properties and by reproducing the results in additional studies. In line with these observations, the present study aimed to define a protocol allowing to reproduce many of these biomarkers and to assess their reliability, validity and interpretability. The main findings are:

- 72 biomarkers previously proposed and assessed in the literature were reproduced in a single dataset;
- 13 biomarkers reached at least a good level in the reliability domain;
- Only 4 biomarkers reached at least a good level in reliability and validity domains and could thus be possibly considered as valuable biomarkers;
- All of these 4 biomarkers were only related to the lumbar sagittal angle during trunk forward bending task;
- All of these 4 biomarkers were in general weakly correlated with patient reported outcome measures, arguing for a potential interest in including related musculoskeletal factors in the establishment of a valuable diagnosis and in guiding treatment response.

A first observation is that for 67 of the 72 (93%) potential movement biomarkers included in this study, the measurement properties were previously assessed by only one study. The present results thus allow us to consolidate or discuss the current knowledge about these biomarkers. In particular, among the 31 movement biomarkers highlighted by the previously published systematic review^6^ as having already been extensively assessed in terms of measurement properties, only 2 reached at least a good level of reliability and validity in our study. For all other biomarkers, this level was not reached (n = 25) or not assessed (n = 4). Hence, the present results confirm previously reported low levels of reliability and validity for 10 biomarkers (i.e. BMo10, BMo23, BMo26, BMo27, BMo28, BMo29, BMo30, BMo33, BMo34, and BMo42) and high levels of reliability and validity for 2 biomarkers (i.e. BMo12 and BMo57). However, we were unable to reproduce the previously published promising results for 15 biomarkers (i.e. BMo3, BMo4, BMo5, BMo6, BMo9, BMo17, BMo24, BMo25, BMo37, BMo43, BMo44, BMo49, BMo58, BMo59, and BMo80). The poor replication of results from the literature (only 44% of similar reliability and validity levels) calls for more research and consolidation of knowledge before these biomarkers can be used in practice. This could be explained by the high heterogeneity across NSCLBP patients^44^ or by variations in the experimental protocol, e.g. task consign, type of sensors, data pre-processing. On the one hand, identifying subgroups of patients might help to avoid comparison of patients with large differences in terms of low back pain profiles. However, such a classification remains challenging^44^ and would require a large database established on sufficiently NSCLBP patients. On the other hand, as already pointed out in a systematic review from our group^6^, there is currently a lack of consensus concerning a robust and standardised biomechanical approach to assess low back pain. A first step towards such a consensus could be to generalise protocol sharing, as it has been done by Rose-Dulcina et al.^8^. It must be noticed that this might be required before to constitute a valuable database through a multi-centric study.

On the whole, among all the biomarkers included in the study, only 4 of them demonstrated a good or excellent level of reliability and validity, i.e. BMo12 (maximal lumbar sagittal angle during trunk sagittal bending), BMo15 (range of motion of lumbar sagittal angle during trunk sagittal bending), BMo18 (mean lumbar sagittal angular velocity during trunk sagittal bending), and BMo57 (maximal upper lumbar sagittal angle during trunk sagittal bending). All these biomarkers are related to the lumbar sagittal angle (i.e. range of motion, maximum amplitude or velocity) during trunk sagittal bending. This result is in line with the meta-analysis conducted by Laird et al. on 35 studies^45^. Indeed, their study identified that, on average, NSCLBP patients have less lumbar range of motion than asymptomatic participants during trunk forward bending. From another point of view, these results point out that none of the other movement parameters or motor tasks demonstrated at least a good level of reliability and validity. However, it must be kept in mind that only kinematic-related biomarkers were included in this study. Other parameters (e.g. spatiotemporal parameters) and tasks (e.g. walking) could demonstrate valuable results, as recently highlighted by Smith et al. in a systematic review focused on walking and running tasks^46^. Based on the AUC, the diagnostic power of these biomarkers ranged from 70 to 77%, which can be considered as good^40^. Using the Youden index as cut-off, a specificity over 90% with a sensitivity ranging between 45 and 50% was observed for BMo12, BMo15, and BMo57. The specificity was lower (62%) for BMo18 with a higher sensitivity (76%). Concerning their interpretability, the MDC_95_ values related to test–retest reliability (intra-rater between-session) in NSCLBP patients were estimated to 16.9°, 13.9°, 4.4° s^−1^, and 13.6° for BMo12, BMo15, BMo18, and BMo57 respectively. This MDC_95_ range is consistent with previously reported values obtained in asymptomatic participants during gait (13.3°) and sit-to-stand (12.9°)^47^, and in NSCLBP patients during gait (up to 14.7°)^48^ and trunk forward bending (up to 19°)^35^. These values are close to the threshold used for clinical interpretation in gait analysis^9^ (i.e. 5° of SEM, 14° of MDC_95_). However, MDC% (i.e. the minimal detectable change divided by the average value of the parameter) was estimated at 24.7% for BMo12 and BMo57, respectively, which was considered here as good^49^.

Considering NSCLBP has a multifactorial problem composed, among others, of psychosocial and biomechanical factors, Cholewicki et al. recently excluded the potential of an isolated use of biomechanics to guide diagnosis and improve treatment strategies^5^. Instead, a multidimensional approach should be considered^5^. As we only demonstrated weak relationships with PROMs, the 4 biomarkers highlighted in this study may have the potential to bring complementary elements of analysis to the psychosocial factors reported by PROMs. Hence, integrating these biomarkers into clinical studies along with well recognised social and psychological factors could improve our understanding of this complex disease and open the scientific community to new therapeutical approaches. Hopefully, integration of the measurement of these biomarkers in a clinical routine is feasible. Indeed, while a complex and costly motion capture system was used in this study, devices with higher clinical applicability and the ability to measure lumbar sagittal angle are already available (e.g. inertial measurement units—IMUs, markerless motion capture systems) and can be used to assess these biomarkers^50^.

Our results must be interpreted carefully since this work has several limitations. First, all the potential movement biomarkers highlighted in the previously published systematic review^6^ were not included in this study. This choice can be justified by material or temporal issues (and thus a limited clinical applicability): 30 biomarkers would require additional or specific devices (e.g. moveable platform^51^, treadmill^52^, specific setup allowing lumbar rotation measurements^53^), 5 biomarkers numerous continuous cycles of a repeated motor task (e.g. 40 cycles of lifting-lowering movements^54^), and the remaining related to additional motor tasks (e.g. squat^55^) could not be included in our experimental protocol due to time constraint. However, it must be noticed that all of the 17 potential biomarkers highlighted as having been previously extensively assessed in terms of measurement properties, with at least good levels in explored domains (e.g. reliability, validity)^6^, were available with our protocol. Second, only the measurement properties of kinematic-related biomarkers were assessed. Other types of parameters (e.g. spatiotemporal parameters, electromyographic parameters, kinetic parameters) have already been reported in the literature and should be similarly analysed in future studies. Hence, while the present study puts forward the trunk sagittal bending task, it does not necessarily mean that other motor tasks might not be explored for other types of parameters. Third, the execution order of the motor tasks was not randomly defined across participants and sessions. Consequently, biomarkers related to the motor tasks performed at the end of the protocol may have been impacted by fatigue. A Borg rating of perceived exertion^56^ could have been performed to monitor the fatigue expressed by the participant. Only pain was monitored in our protocol all along the motor tasks executions and no significant increase of pain was observed in the participants. Fourth, while an extensive assessment of the measurement properties has been achieved in this study, several domains remain to be explored. In particular, following the COSMIN checklist^10^, reliability and interpretability domains have not been fully investigated, and responsiveness is missing. Concerning reliability, inter-rater reliability should be assessed before generalising the use of a biomarker. The exploration of this subdomain was unfortunately not possible within the time frame of this study. However, it could be investigated in future studies only on the most suitable biomarkers highlighted here, thus drastically reducing the length of the protocol. Concerning responsiveness, an additional experimental session several months later would have been required to investigate the biomarker value variations with a modified clinical status (e.g. increase/decrease of the pain). Concerning interpretability, the reported minimal detectable change (MDC) should be completed with the computation of a minimal important change (MIC) of the biomarkers^10,57^. Again, an additional experimental session would have been required.

NSCLBP is a complex disorder where central and peripheral pain processes are influenced by various factors such as social, psychological or musculoskeletal factors which interact with each other^1,2^. The present results suggest that musculoskeletal factors such as the lumbar sagittal angle during trunk sagittal bending could bring relevant additional information to the psychosocial state of the patient to establish a valuable diagnosis and to guide treatment. From a practical perspective, this is an encouraging result as this kind of parameter can be easily recorded using embedded sensors such as inertial measurement units (IMU)^58^ which offer high clinical applicability often associated with low costs. A similar study will be carried out within our group on the muscular activity biomarkers highlighted in the previously published systematic review^6^.

## Supplementary Information

Supplementary Information 1.

Supplementary Information 2.

Supplementary Information 3.

Supplementary Information 4.

## Data Availability

Raw data with 3D marker trajectories are available in standard c3d file format (https://www.c3d.org) under the Creative Common licence CC BY-NC (https://creativecommons.org/licenses/by-nc/4.0/) on the Geneva University data repository Yareta (https://doi.org/10.26037/yareta:aawpwqaunbcmbnxs2i3optmaei). Contact author can be contacted for any request about the data (Florent Moissenet, florent.moissenet@unige.ch). Matlab codes used to pre-process data (https://gitlab.unige.ch/KLab/KLAB_Preprocessing_toolbox) and compute biomarkers (https://gitlab.unige.ch/KLab/NSLBP-BIO_Toolbox) are shared in open access through dedicated Gitlab repositories.
