# Supplementary material for: Measurement properties of 72 movement biomarkers aiming to discriminate non‑specific chronic low back pain patients from an asymptomatic population
Source: Sci Rep. 2023 Apr 20;13:6483. doi: 10.1038/s41598-023-33504-5 (PMC10119171; doi:10.1038/s41598-023-33504-5)
Supplement: Supplementary file 1 — Supplementary Information 1. [file 41598_2023_33504_MOESM1_ESM.docx]

| **Measurement properties of 72 movement biomarkers aiming to discriminate non‑specific chronic low back pain patients from an asymptomatic population** |
| --- |
| Florent Moissenet^1^, Stéphane Armand^1^, Stéphane Genevay^2^ |
| *^1^ Kinesiology Laboratory, Geneva University Hospitals and University of Geneva, Geneva, Switzerland* |
| *^2^ Department of Rheumatology, Geneva University Hospitals, Geneva, Switzerland* |
|  |
| **Supplementary Information File -** Full measurement properties of all of the 72 movement biomarkers |

**Supplementary Data 1** - Normality, boxplot and ROC of each movement biomarker (File: Supplementary Data 1 - Normality, boxplot and ROC of each movement biomarker.zip, normality: BMo*_normality.png files, boxplot: BMo*_boxplot.png files, ROC: BMo*_ROC.png files).

**Supplementary Table 1** - Full measurement properties of all of the 72 movement biomarkers (File: Supplementary Table 1 - Full measurement properties of all of the 72 movement biomarkers.xlsx).

**Supplementary Table 2** - Description of the motor tasks explored in this study (File: Supplementary Table 2 - Description of the motor tasks explored in this study.xlsx).
