# Supplementary figures and images for: Measurement properties of 72 movement biomarkers aiming to discriminate non‑specific chronic low back pain patients from an asymptomatic population (part 2 of 2)
Source: Sci Rep. 2023 Apr 20;13:6483. doi: 10.1038/s41598-023-33504-5 (PMC10119171; doi:10.1038/s41598-023-33504-5)

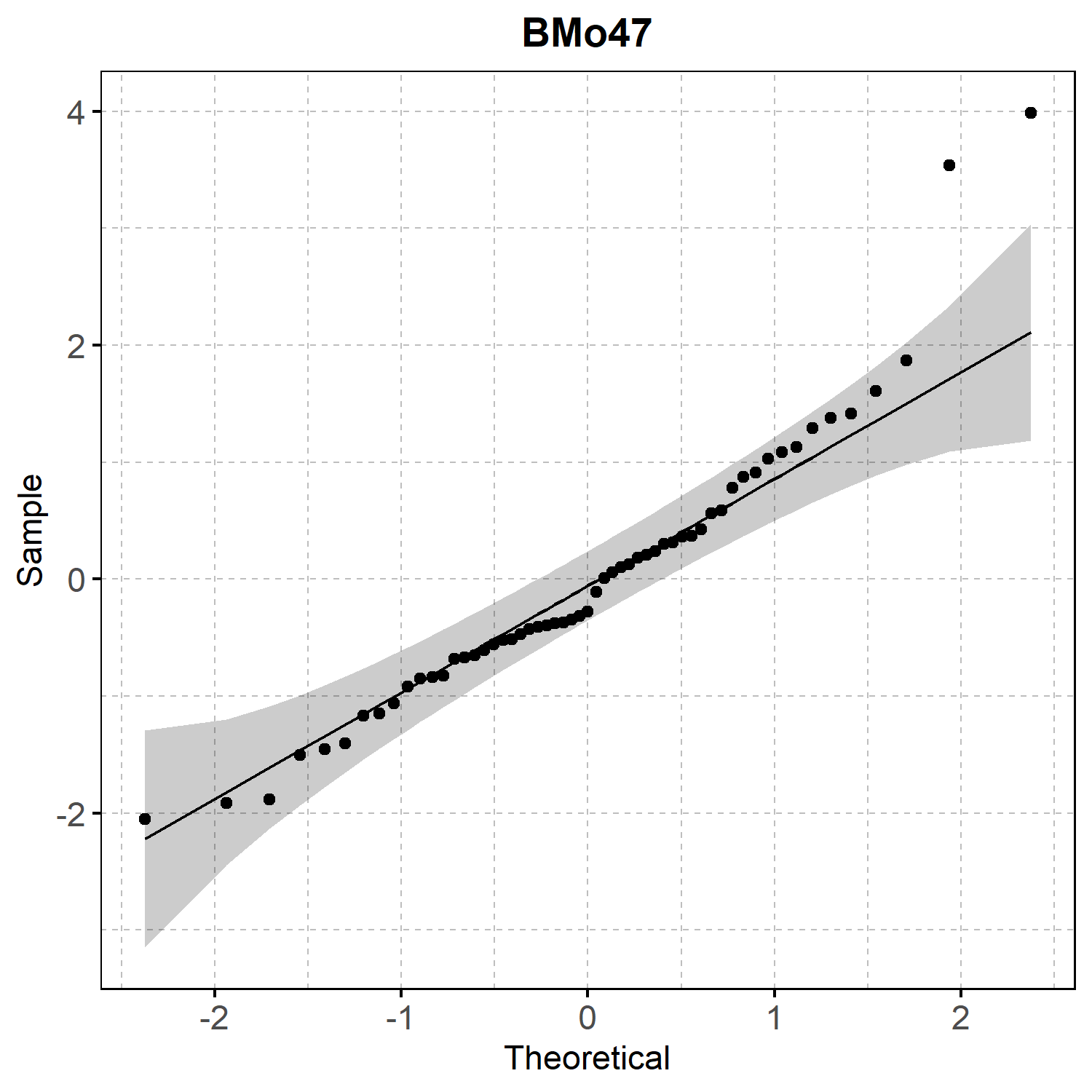

Supplement: Supplementary file 2 — Supplementary Information 2. [file 41598_2023_33504_MOESM2_ESM.zip › BMo047_normality.png]

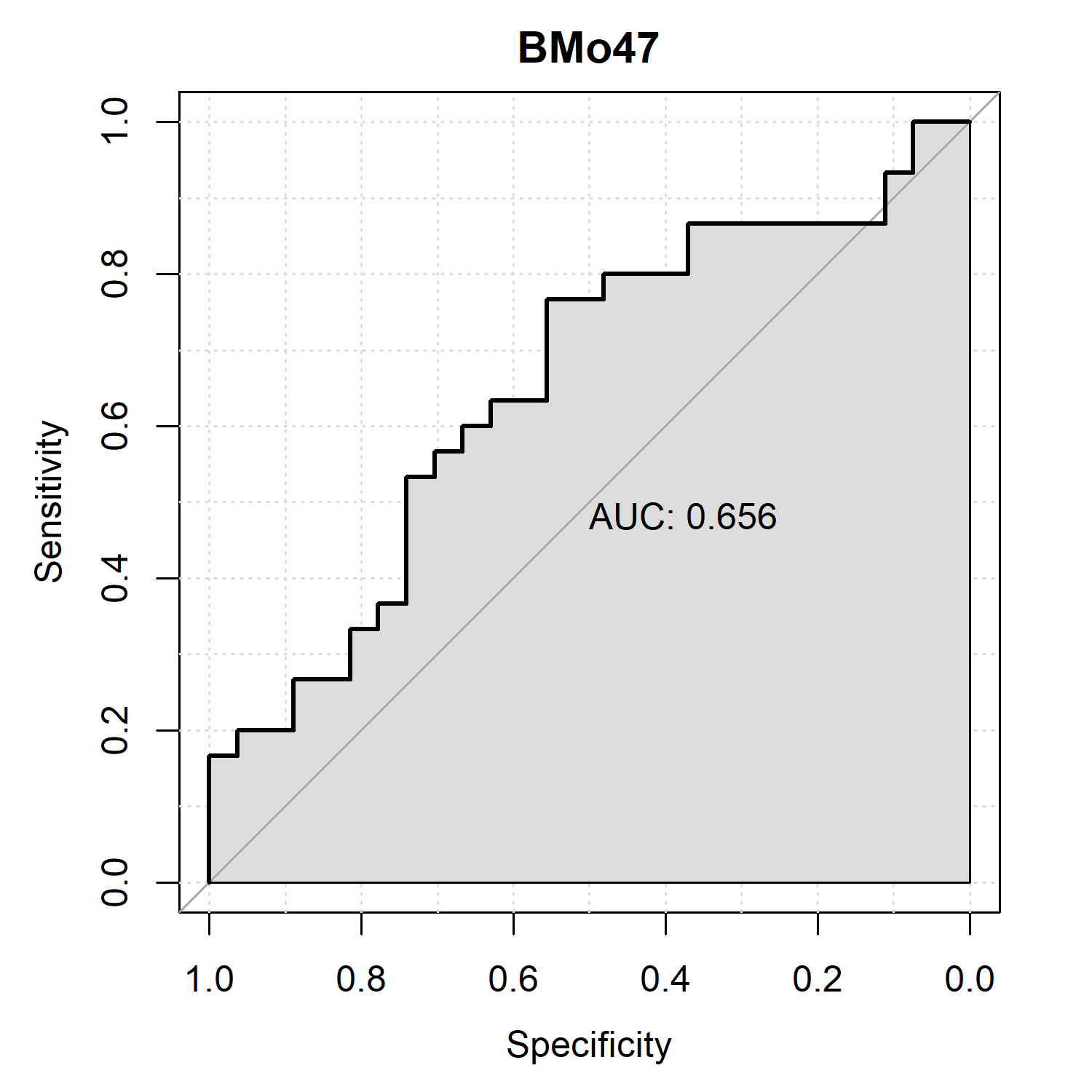

Supplement: Supplementary file 2 — Supplementary Information 2. [file 41598_2023_33504_MOESM2_ESM.zip › BMo047_ROC.png]

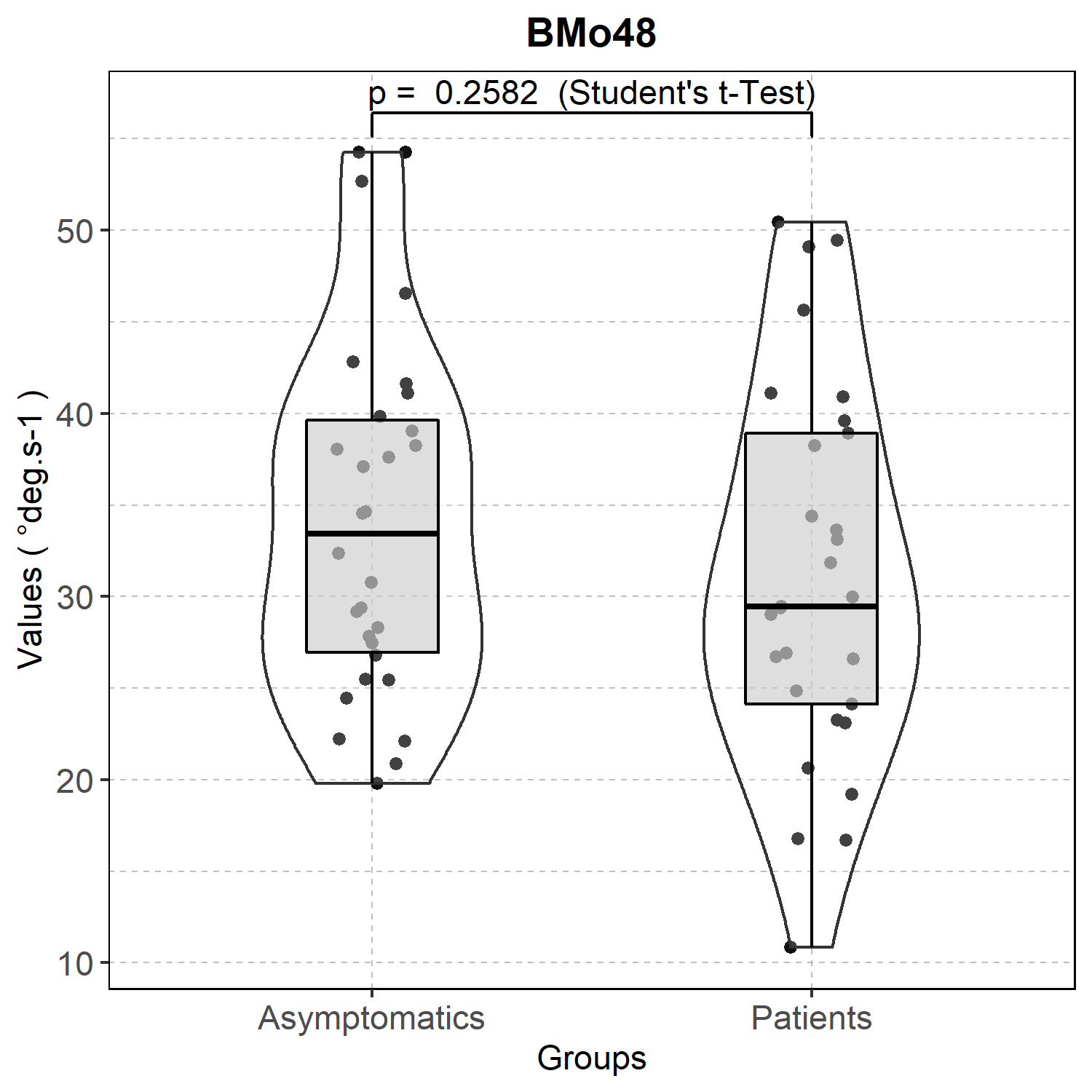

Supplement: Supplementary file 2 — Supplementary Information 2. [file 41598_2023_33504_MOESM2_ESM.zip › BMo048_boxplot.png]

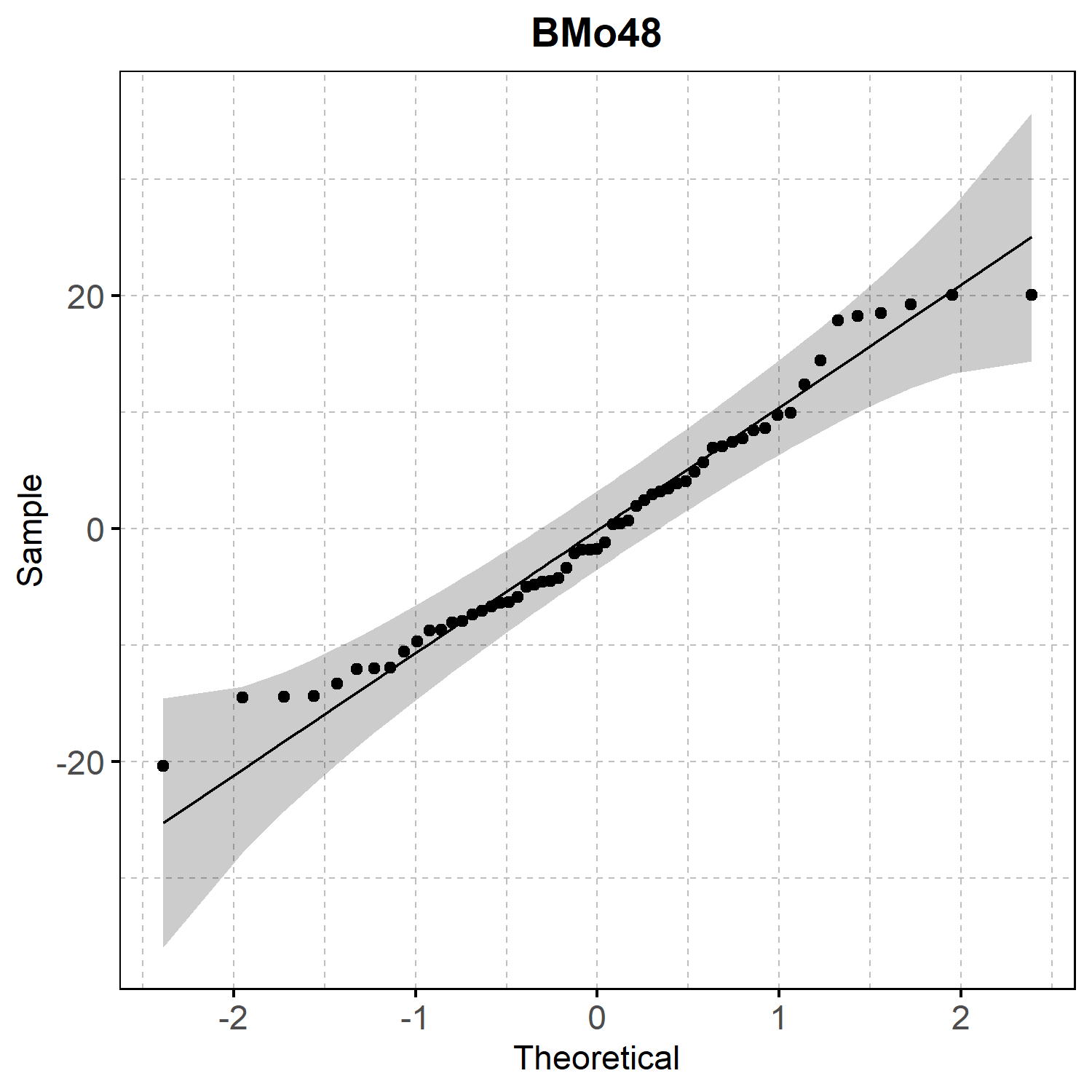

Supplement: Supplementary file 2 — Supplementary Information 2. [file 41598_2023_33504_MOESM2_ESM.zip › BMo048_normality.png]

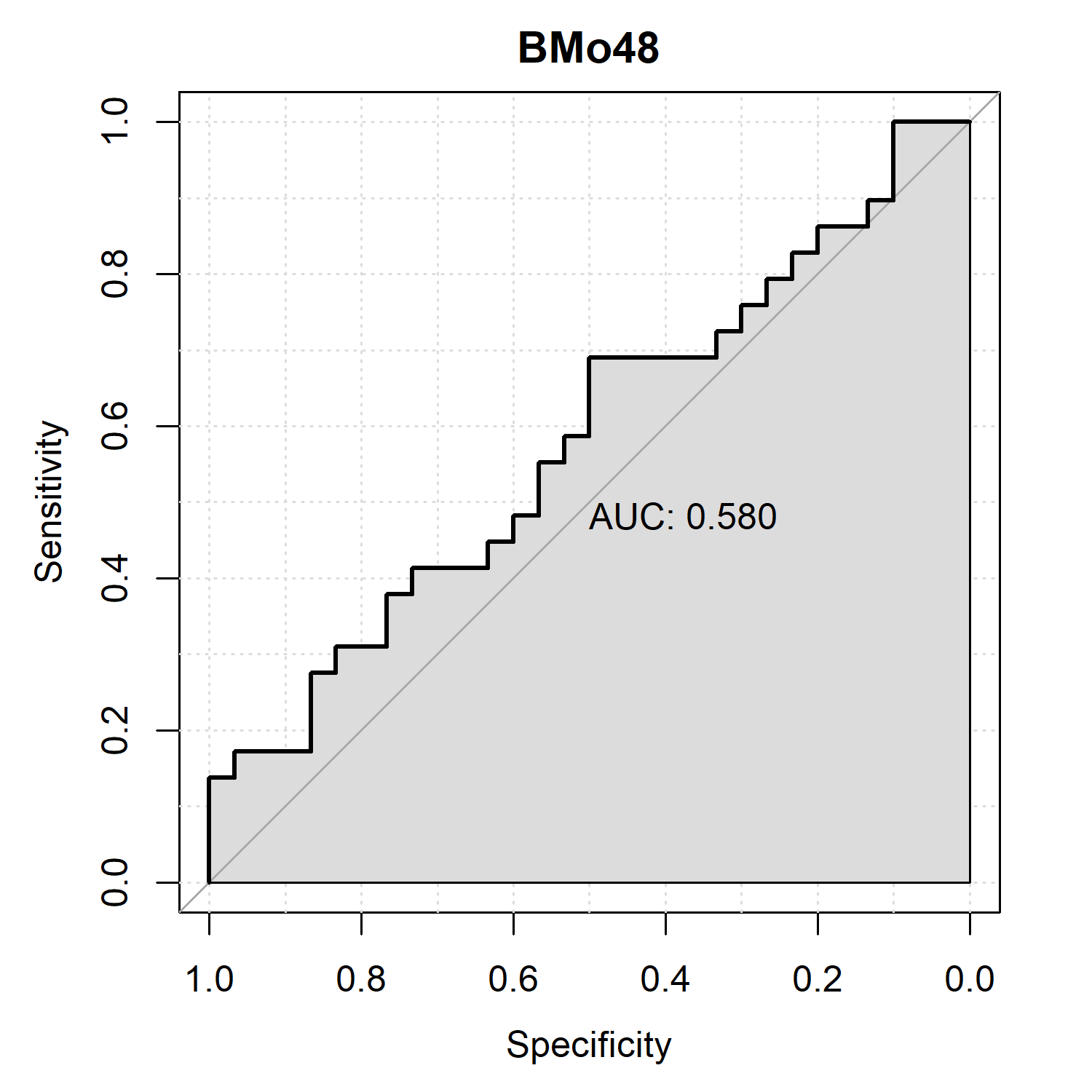

Supplement: Supplementary file 2 — Supplementary Information 2. [file 41598_2023_33504_MOESM2_ESM.zip › BMo048_ROC.png]

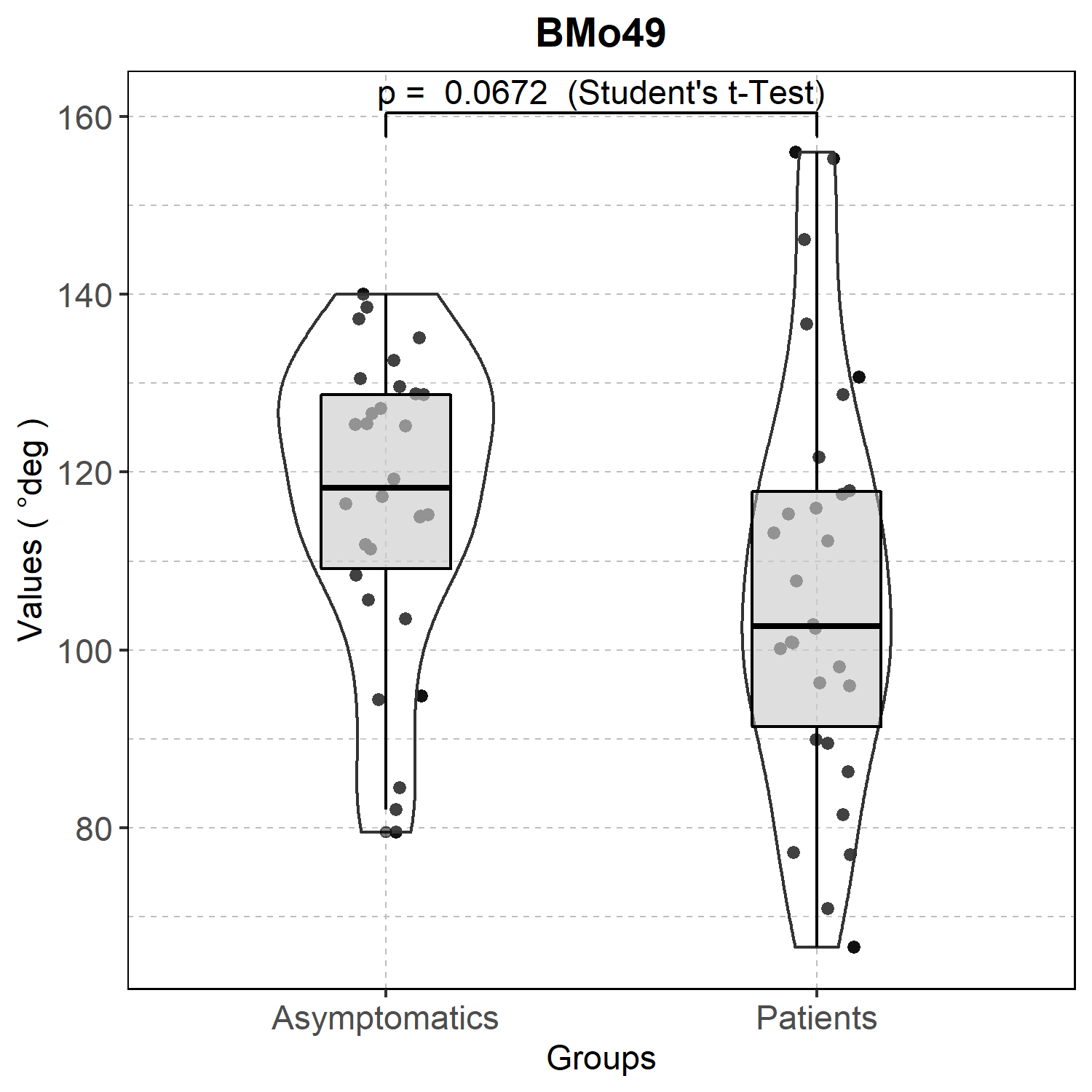

Supplement: Supplementary file 2 — Supplementary Information 2. [file 41598_2023_33504_MOESM2_ESM.zip › BMo049_boxplot.png]

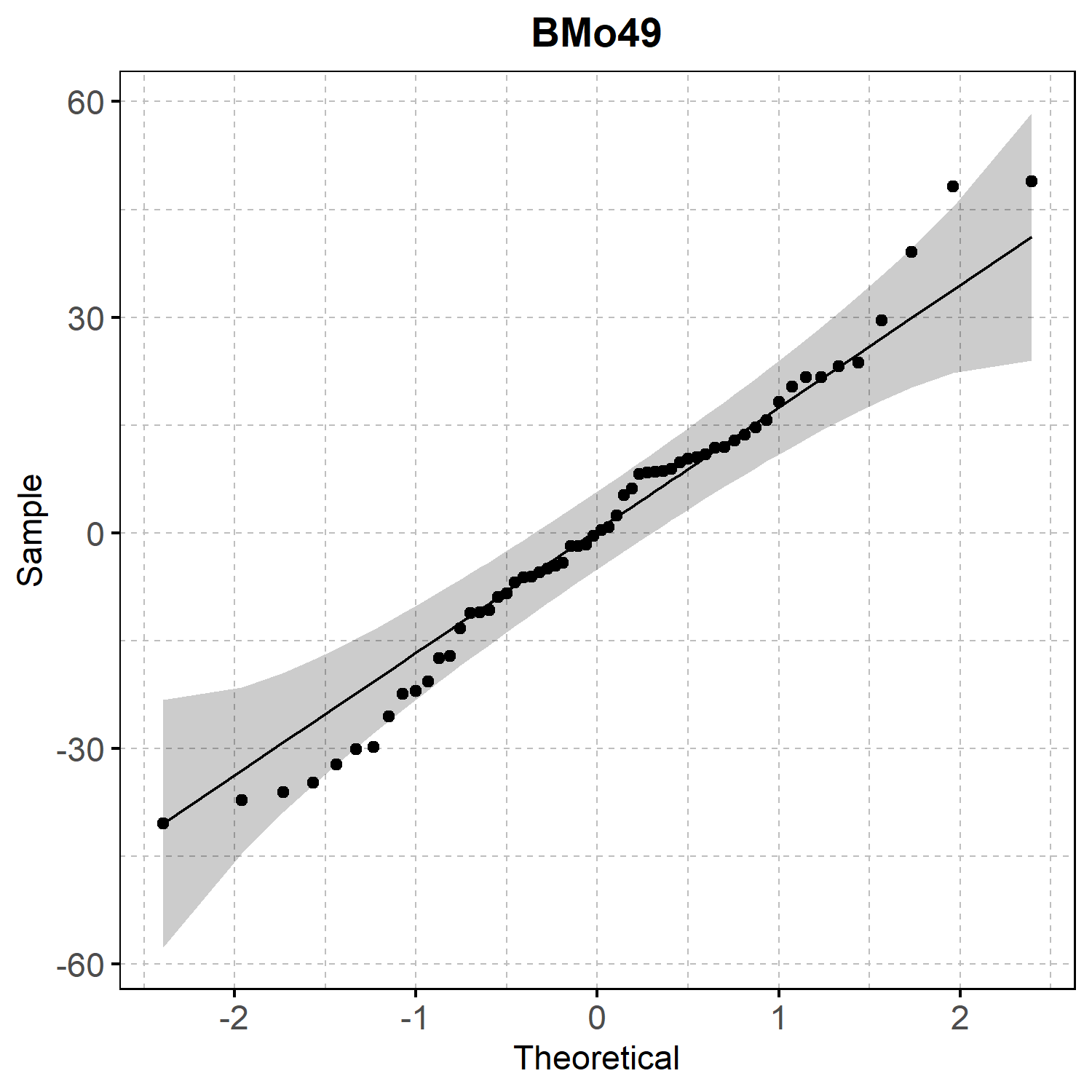

Supplement: Supplementary file 2 — Supplementary Information 2. [file 41598_2023_33504_MOESM2_ESM.zip › BMo049_normality.png]

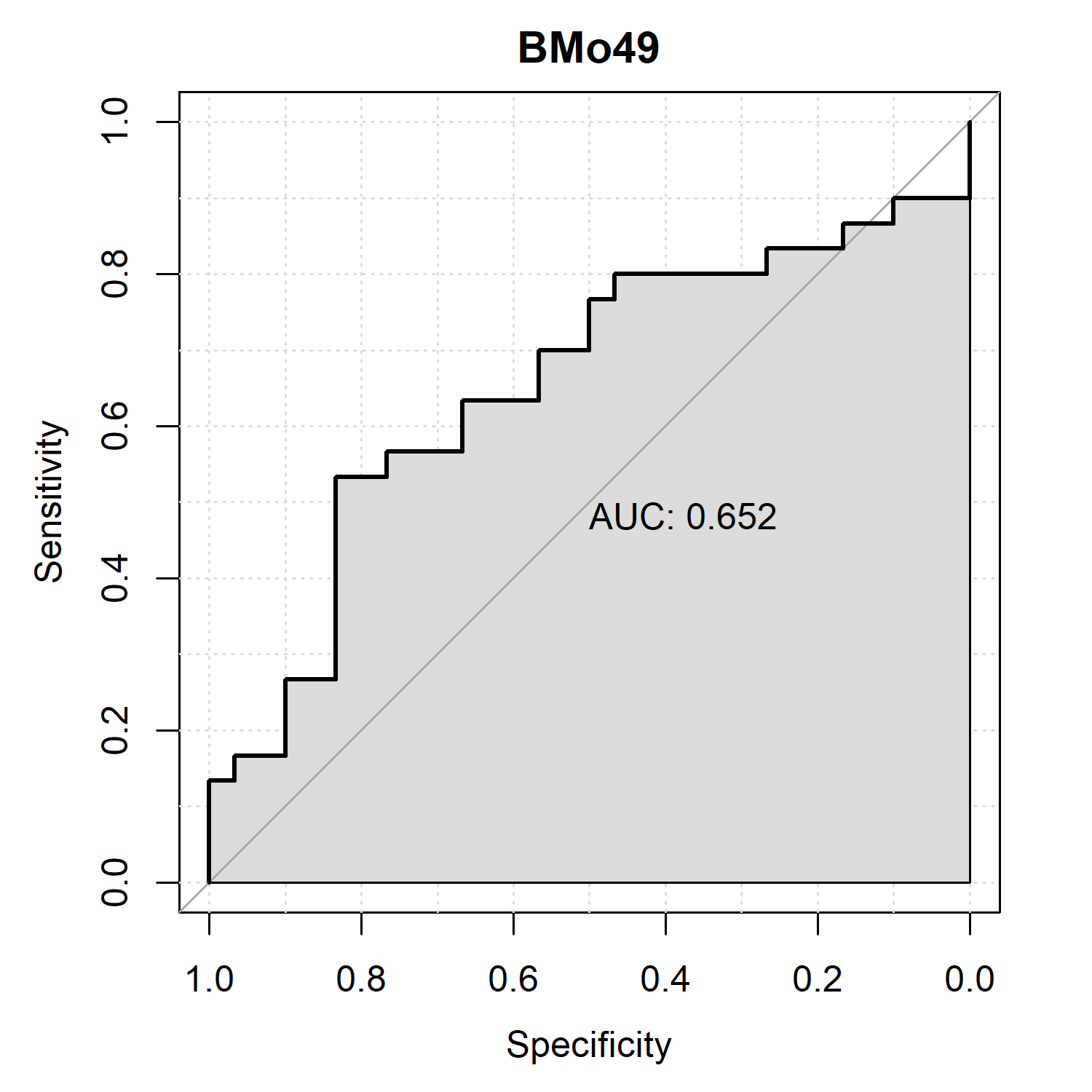

Supplement: Supplementary file 2 — Supplementary Information 2. [file 41598_2023_33504_MOESM2_ESM.zip › BMo049_ROC.png]

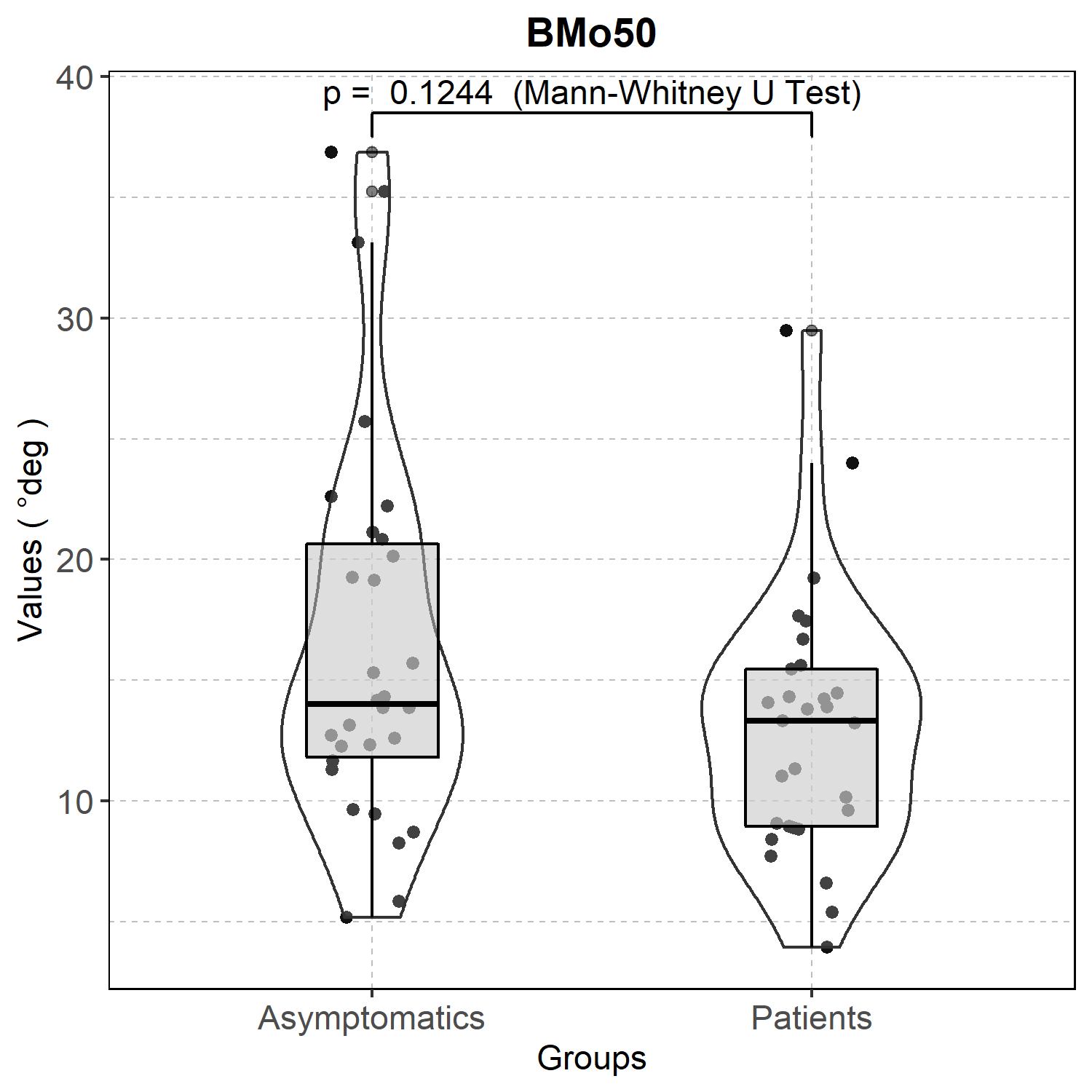

Supplement: Supplementary file 2 — Supplementary Information 2. [file 41598_2023_33504_MOESM2_ESM.zip › BMo050_boxplot.png]

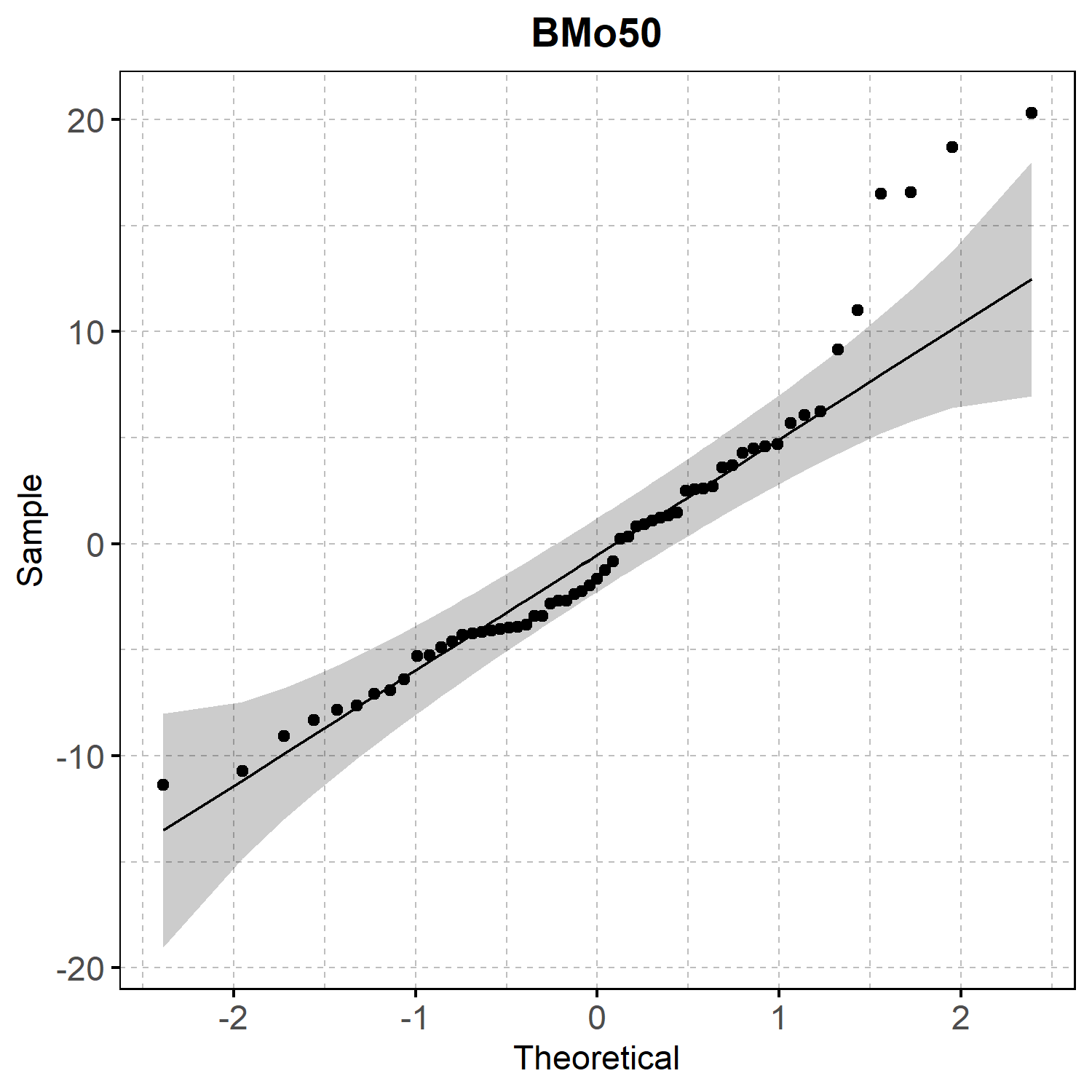

Supplement: Supplementary file 2 — Supplementary Information 2. [file 41598_2023_33504_MOESM2_ESM.zip › BMo050_normality.png]

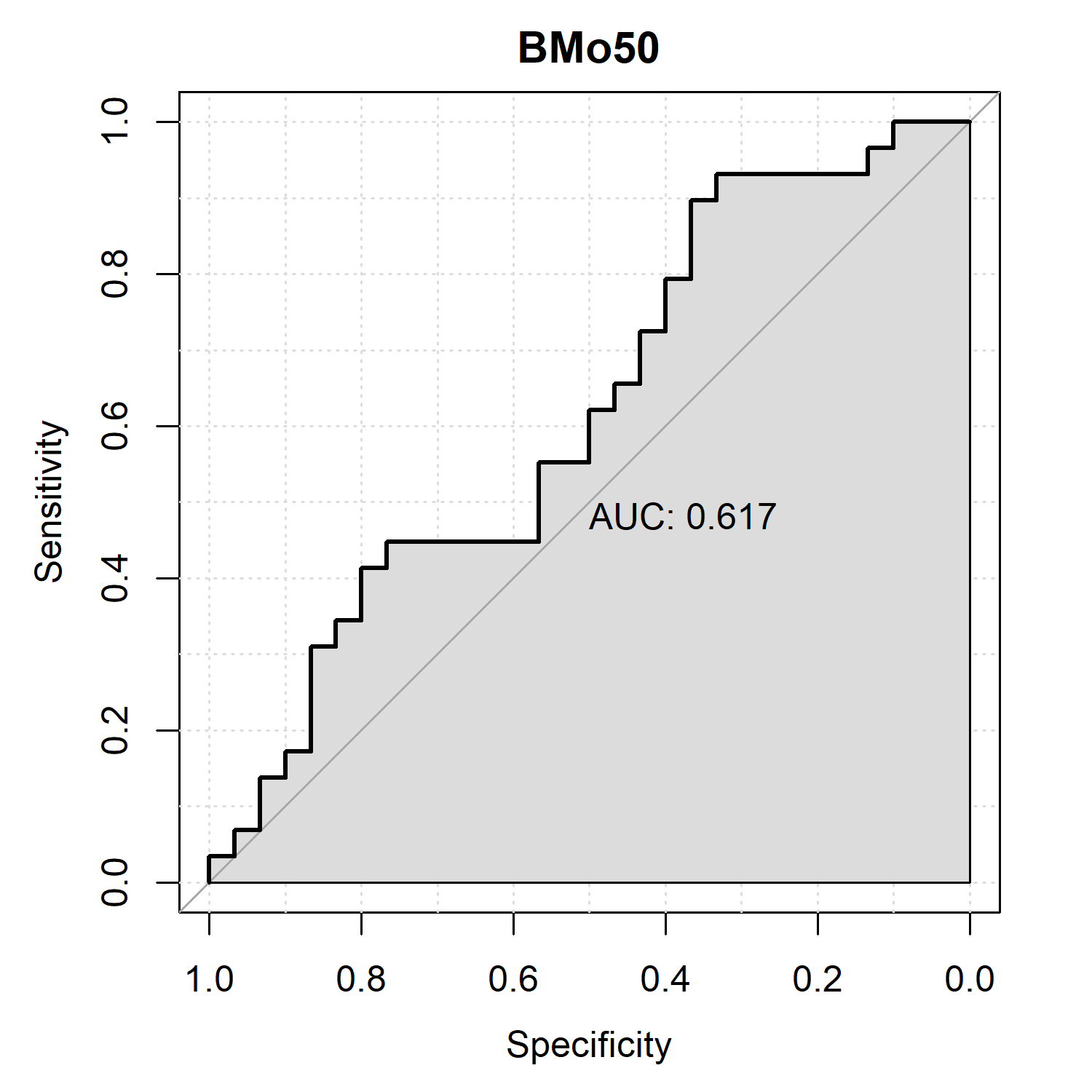

Supplement: Supplementary file 2 — Supplementary Information 2. [file 41598_2023_33504_MOESM2_ESM.zip › BMo050_ROC.png]

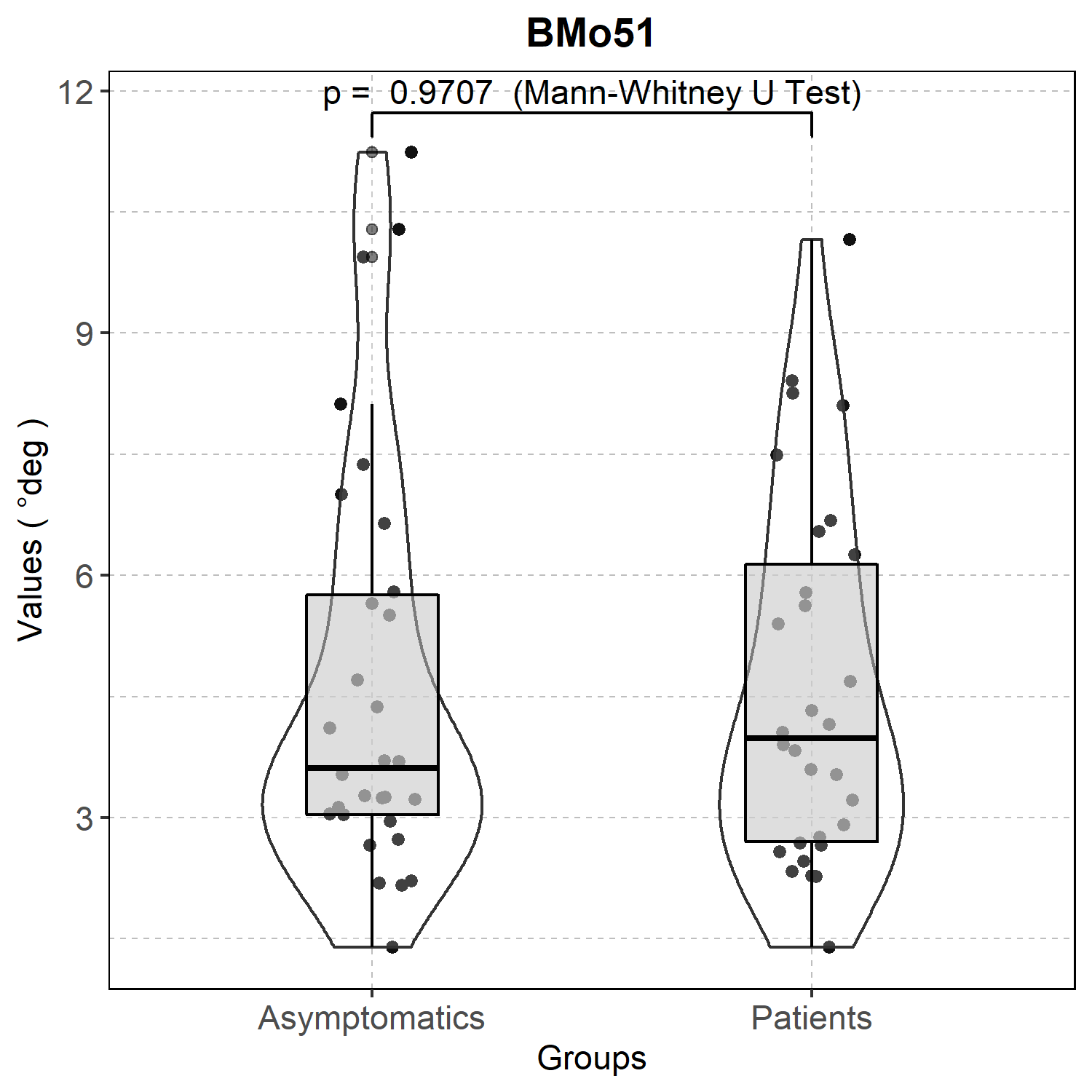

Supplement: Supplementary file 2 — Supplementary Information 2. [file 41598_2023_33504_MOESM2_ESM.zip › BMo051_boxplot.png]

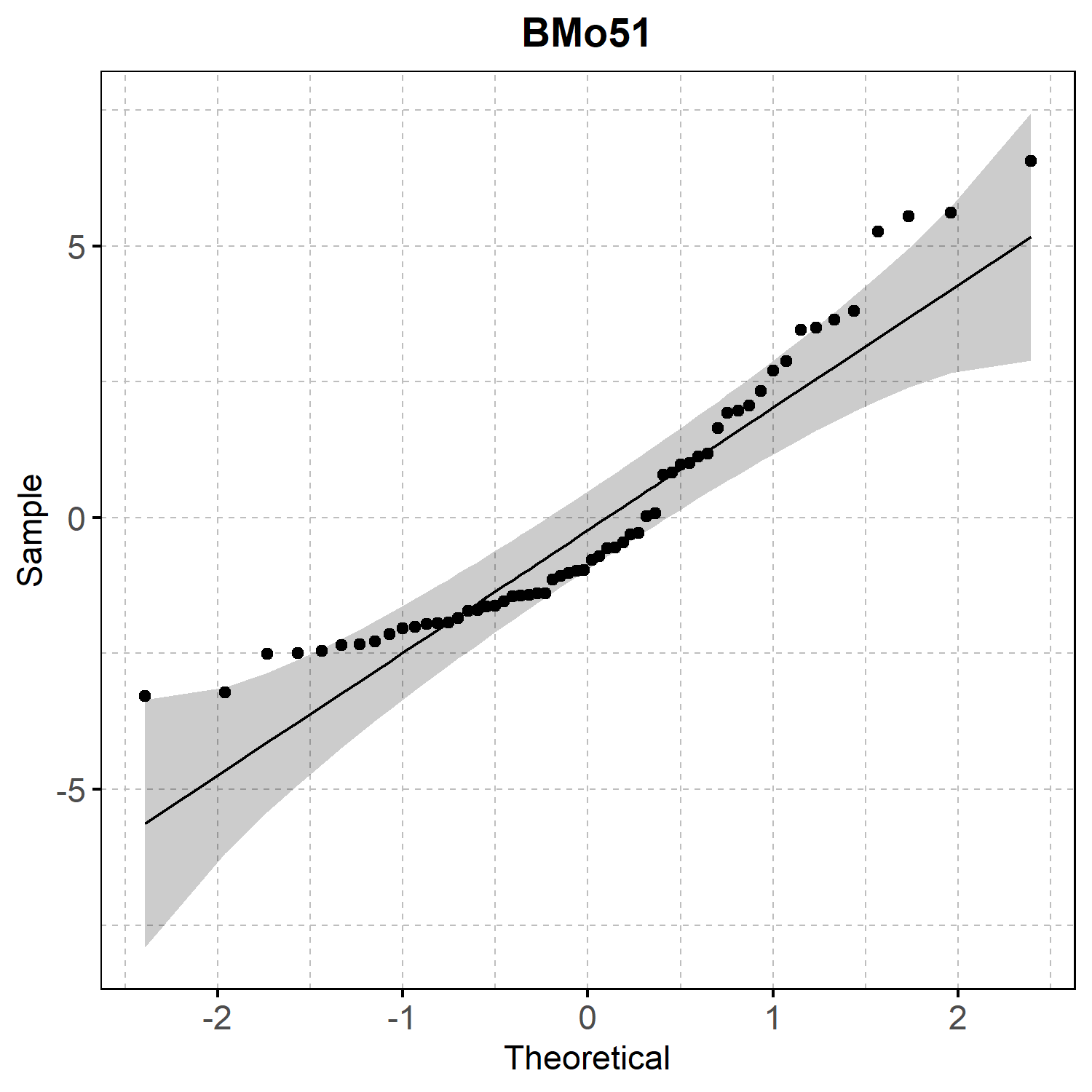

Supplement: Supplementary file 2 — Supplementary Information 2. [file 41598_2023_33504_MOESM2_ESM.zip › BMo051_normality.png]

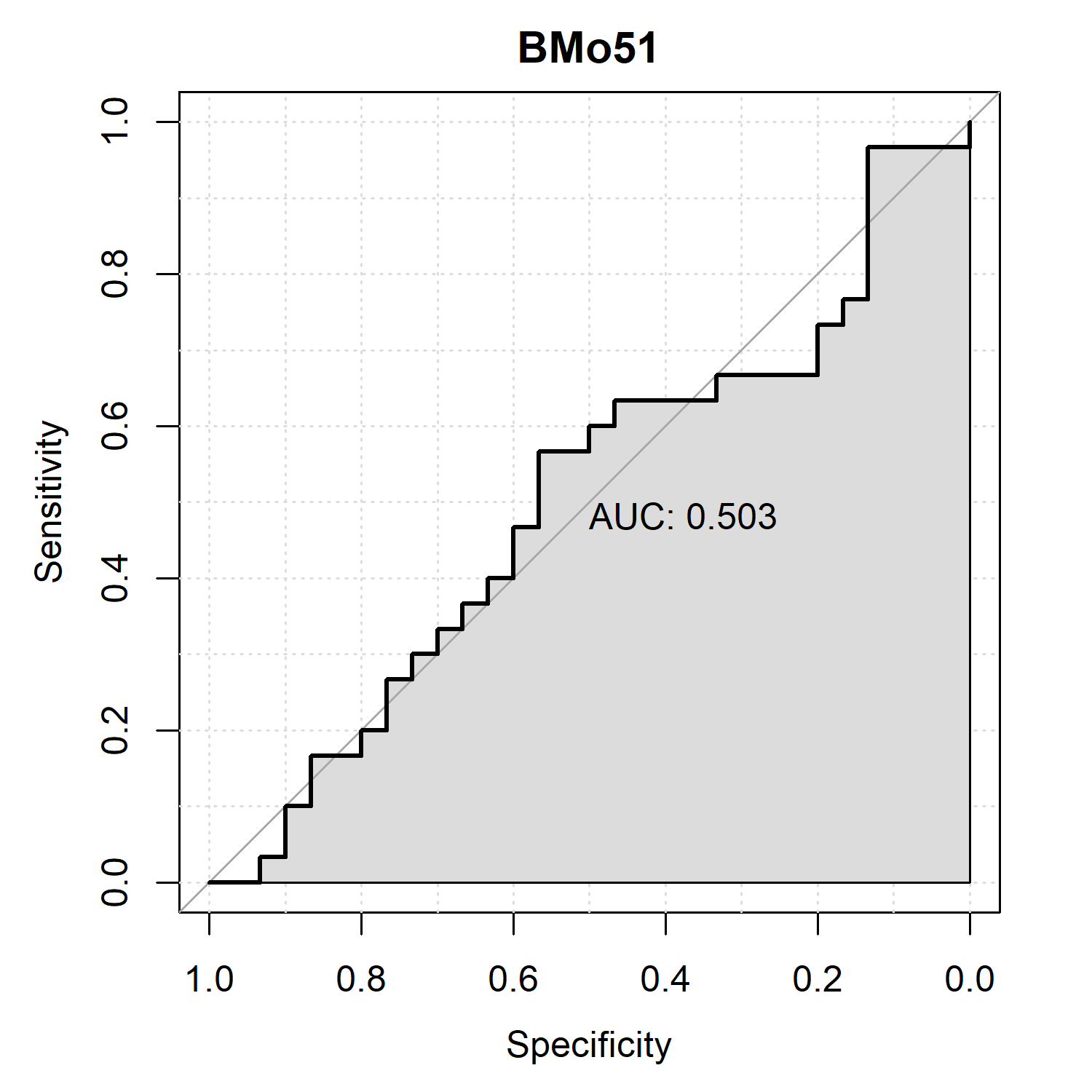

Supplement: Supplementary file 2 — Supplementary Information 2. [file 41598_2023_33504_MOESM2_ESM.zip › BMo051_ROC.png]

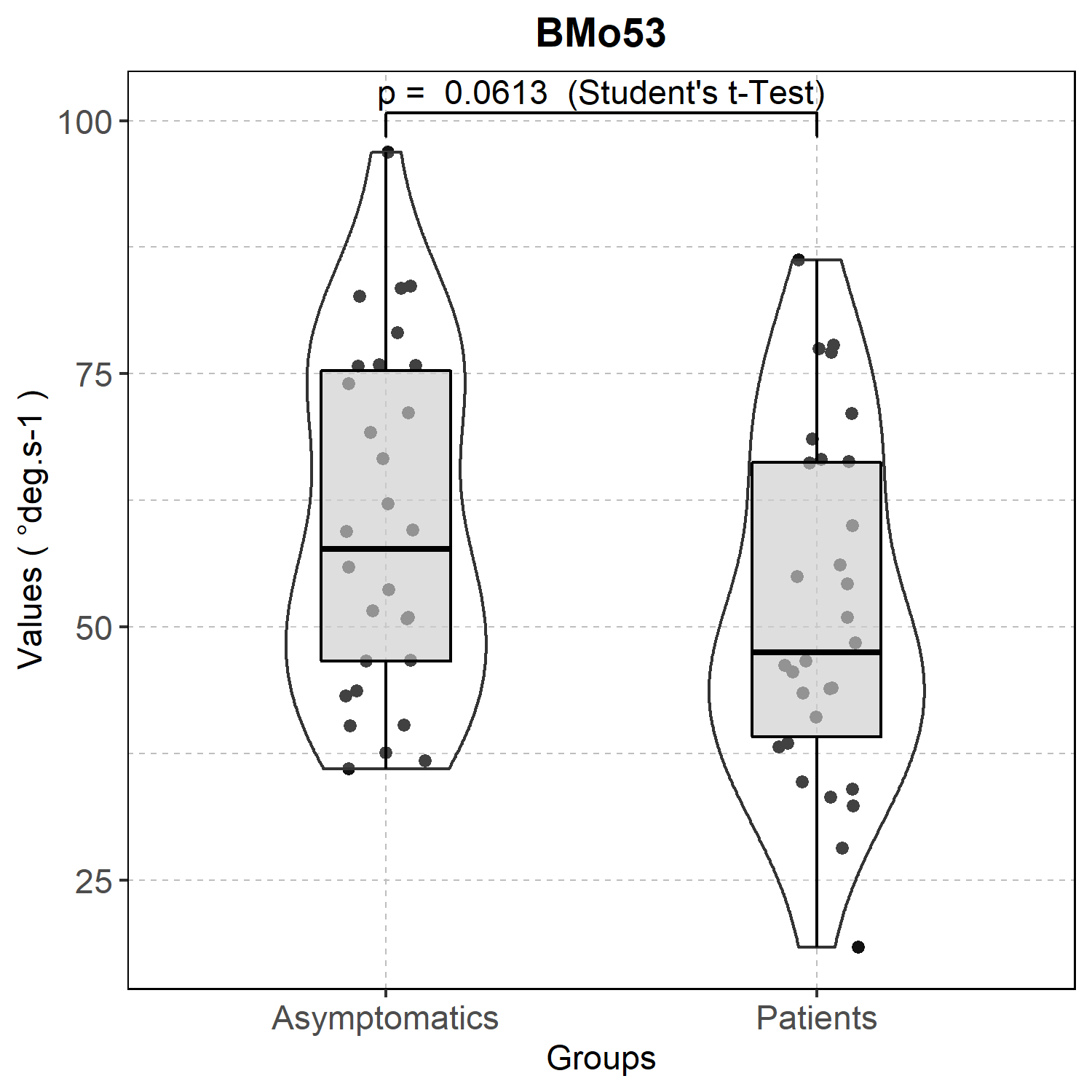

Supplement: Supplementary file 2 — Supplementary Information 2. [file 41598_2023_33504_MOESM2_ESM.zip › BMo053_boxplot.png]

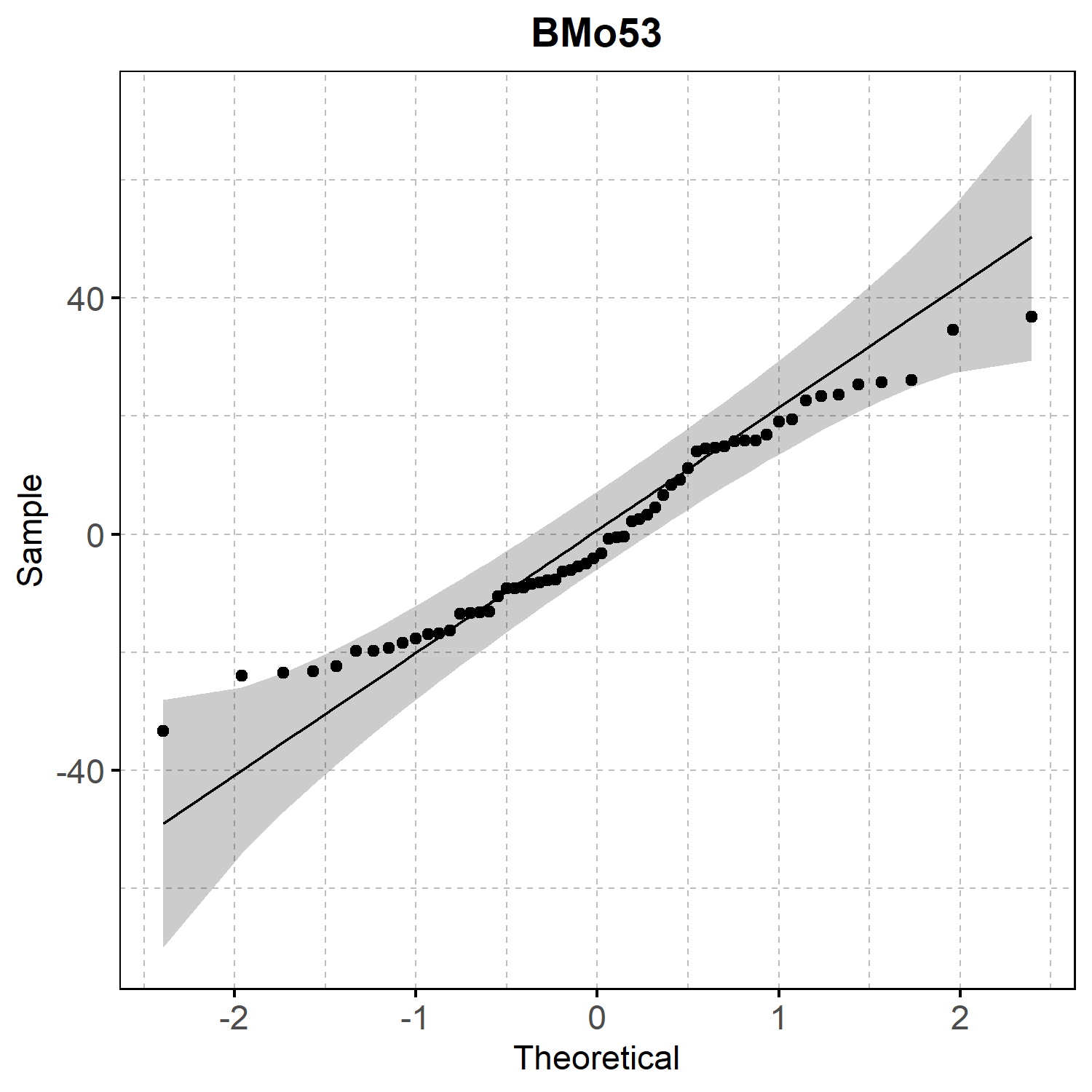

Supplement: Supplementary file 2 — Supplementary Information 2. [file 41598_2023_33504_MOESM2_ESM.zip › BMo053_normality.png]

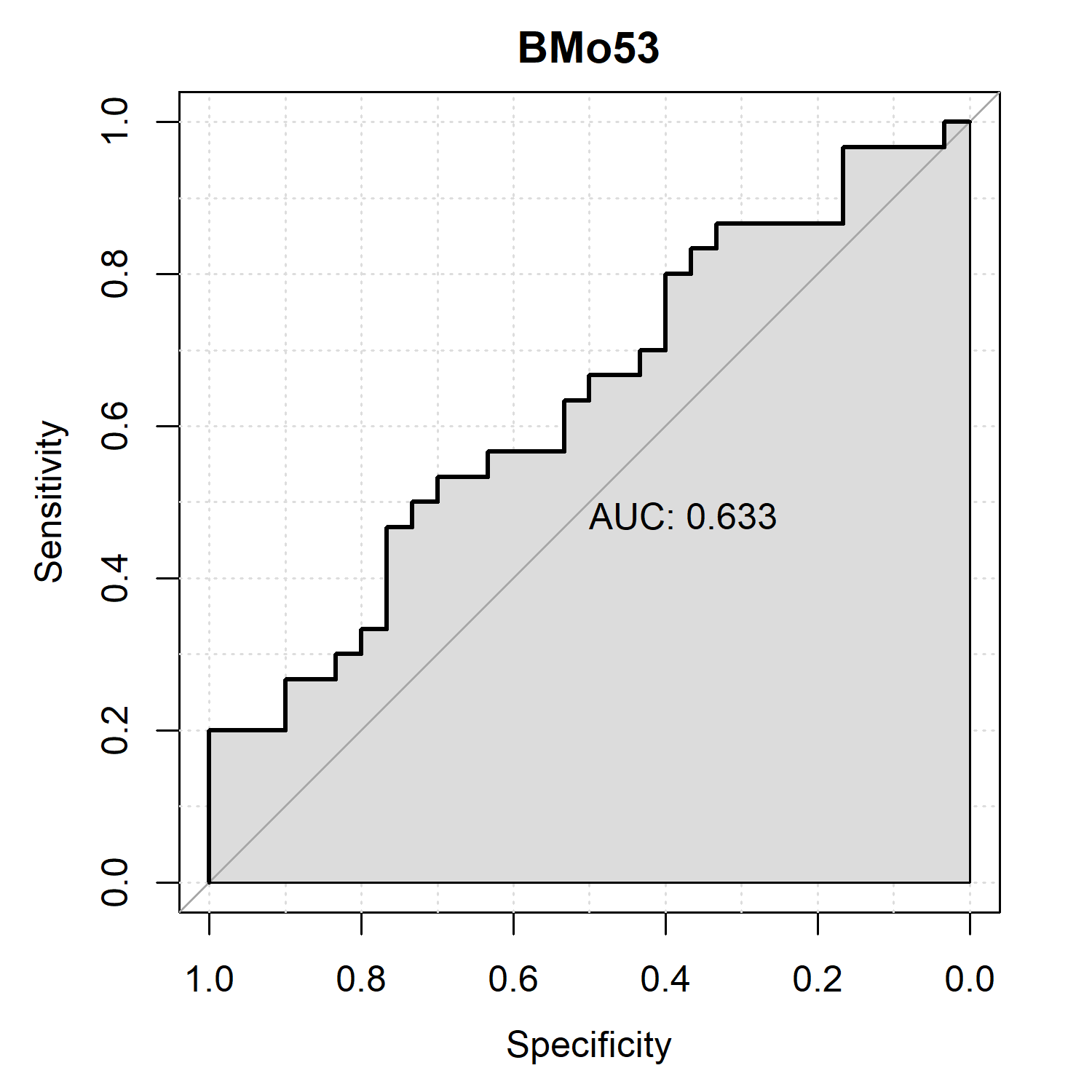

Supplement: Supplementary file 2 — Supplementary Information 2. [file 41598_2023_33504_MOESM2_ESM.zip › BMo053_ROC.png]

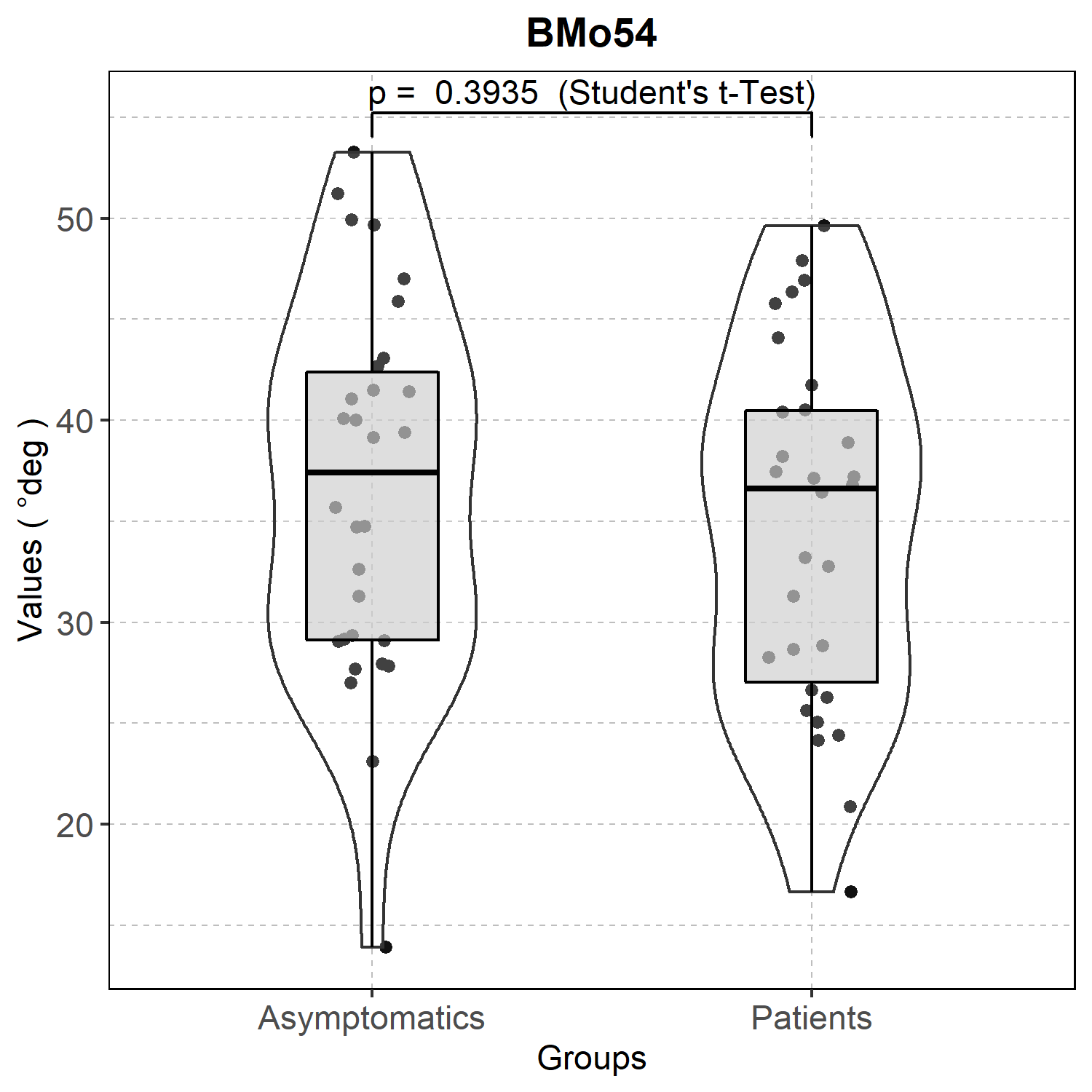

Supplement: Supplementary file 2 — Supplementary Information 2. [file 41598_2023_33504_MOESM2_ESM.zip › BMo054_boxplot.png]

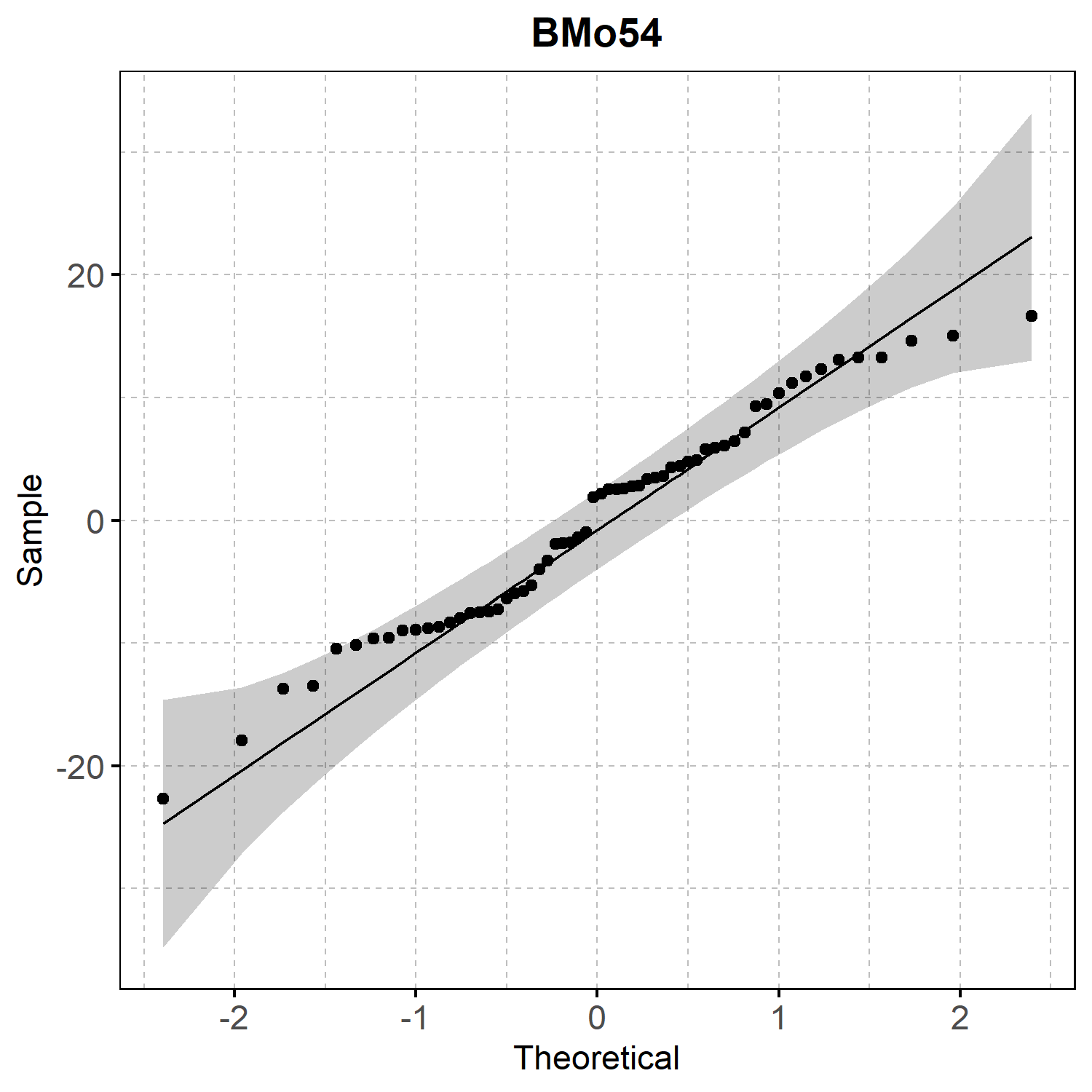

Supplement: Supplementary file 2 — Supplementary Information 2. [file 41598_2023_33504_MOESM2_ESM.zip › BMo054_normality.png]

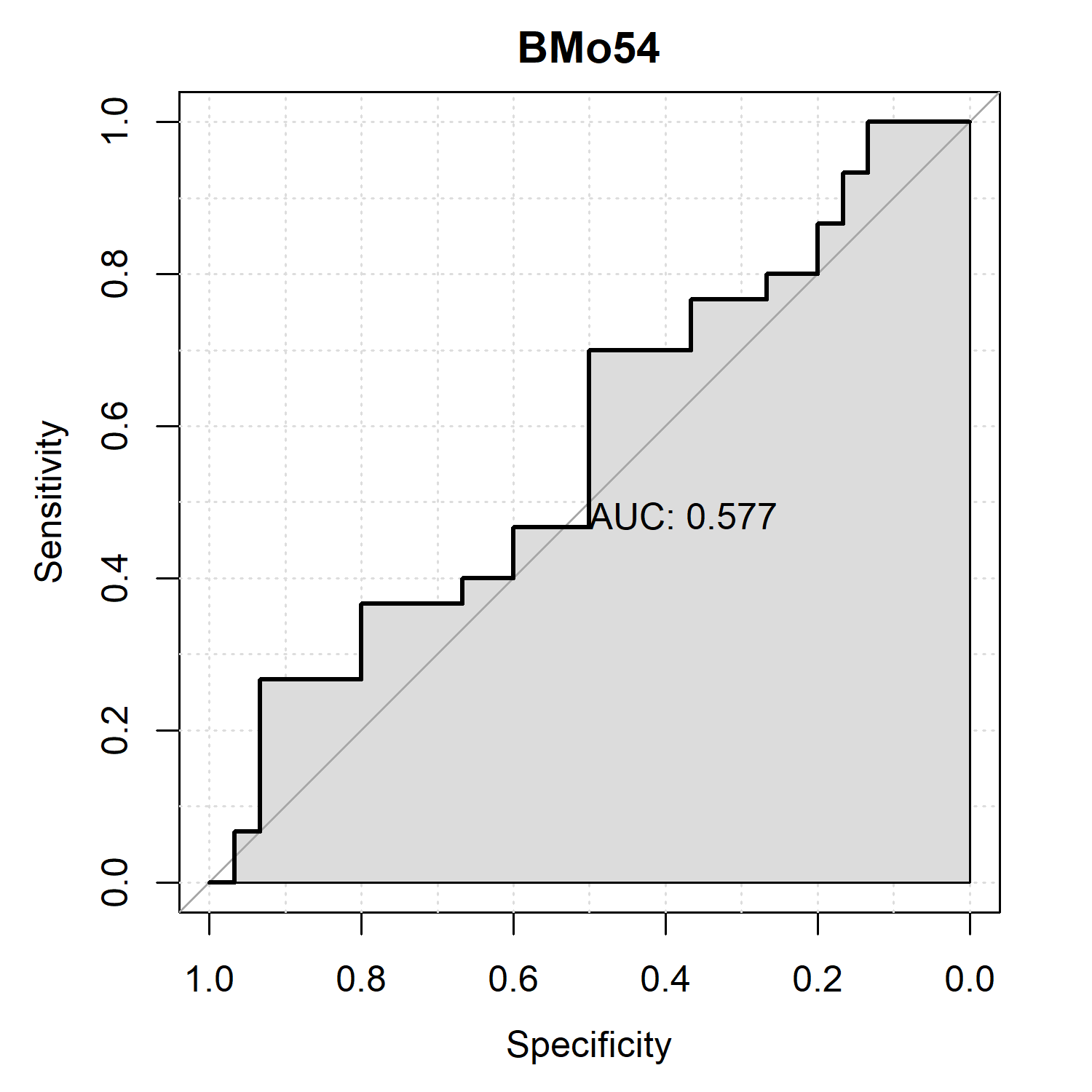

Supplement: Supplementary file 2 — Supplementary Information 2. [file 41598_2023_33504_MOESM2_ESM.zip › BMo054_ROC.png]

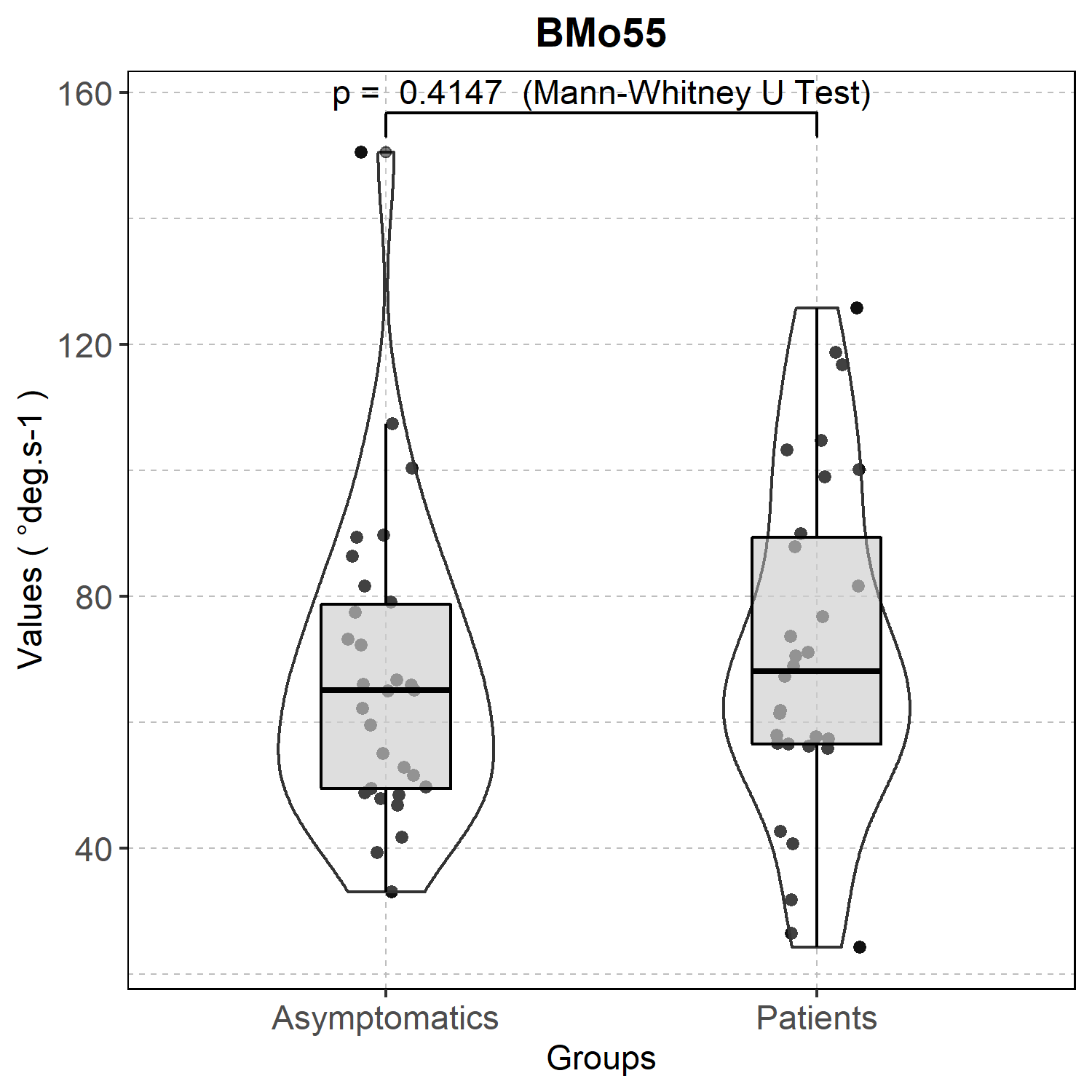

Supplement: Supplementary file 2 — Supplementary Information 2. [file 41598_2023_33504_MOESM2_ESM.zip › BMo055_boxplot.png]

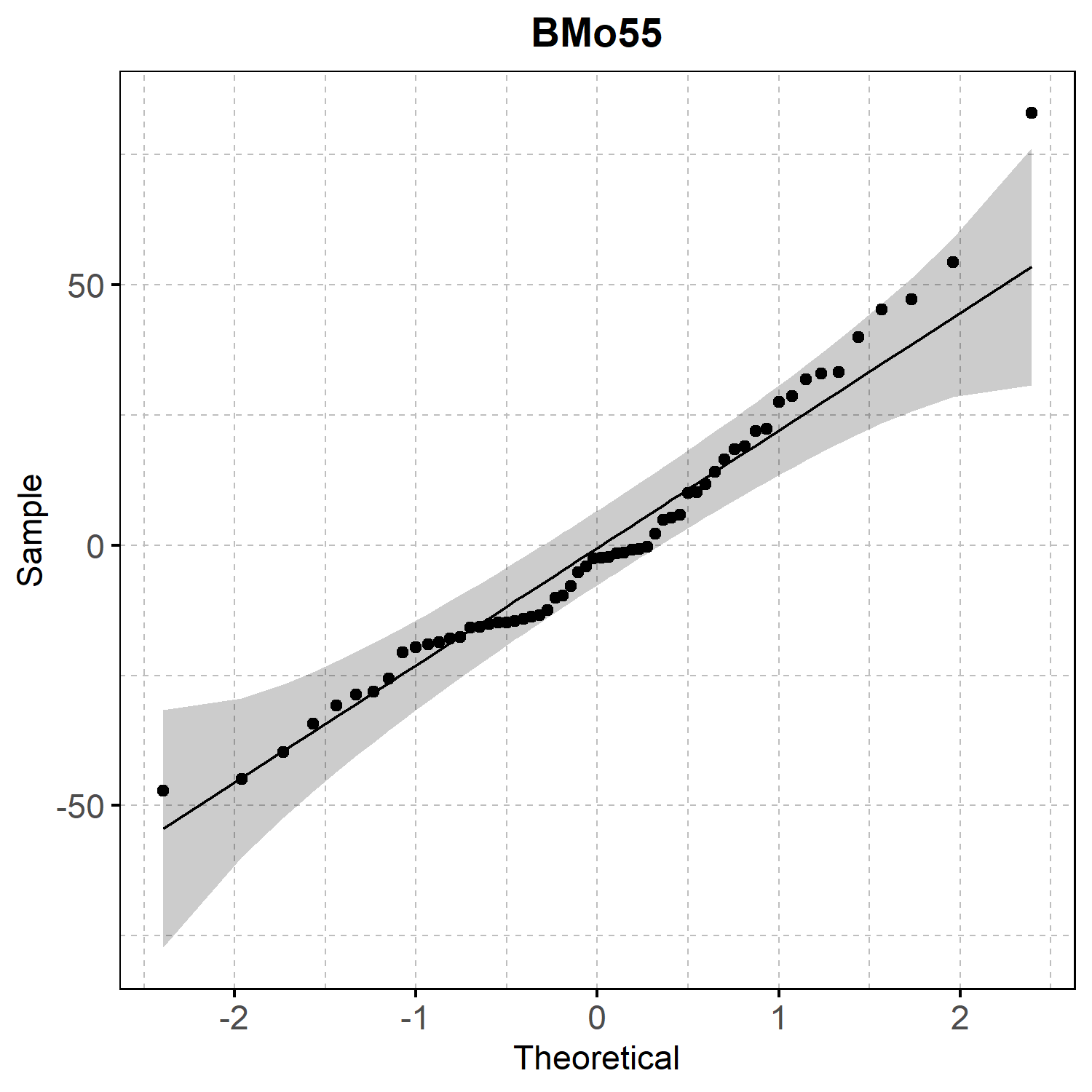

Supplement: Supplementary file 2 — Supplementary Information 2. [file 41598_2023_33504_MOESM2_ESM.zip › BMo055_normality.png]

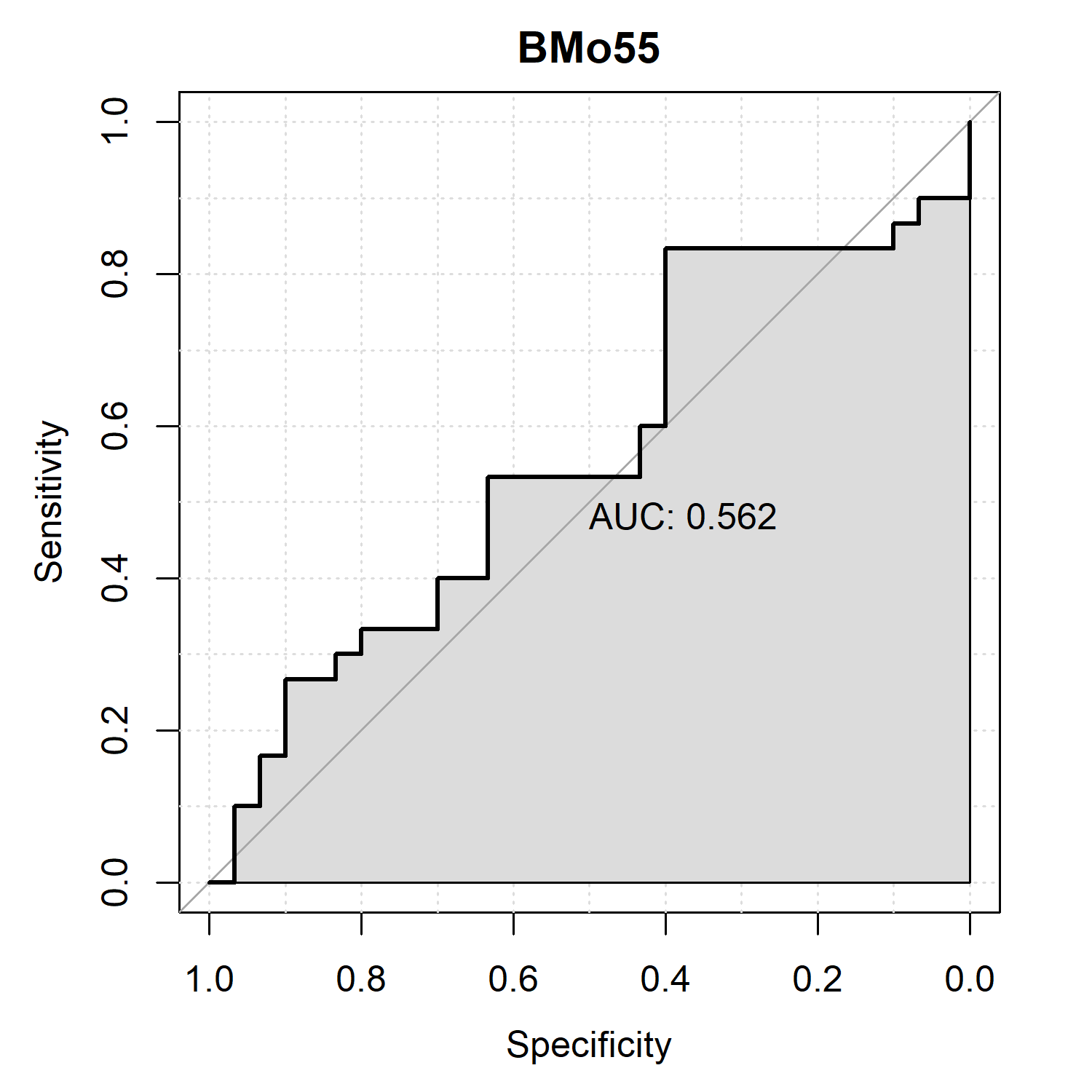

Supplement: Supplementary file 2 — Supplementary Information 2. [file 41598_2023_33504_MOESM2_ESM.zip › BMo055_ROC.png]

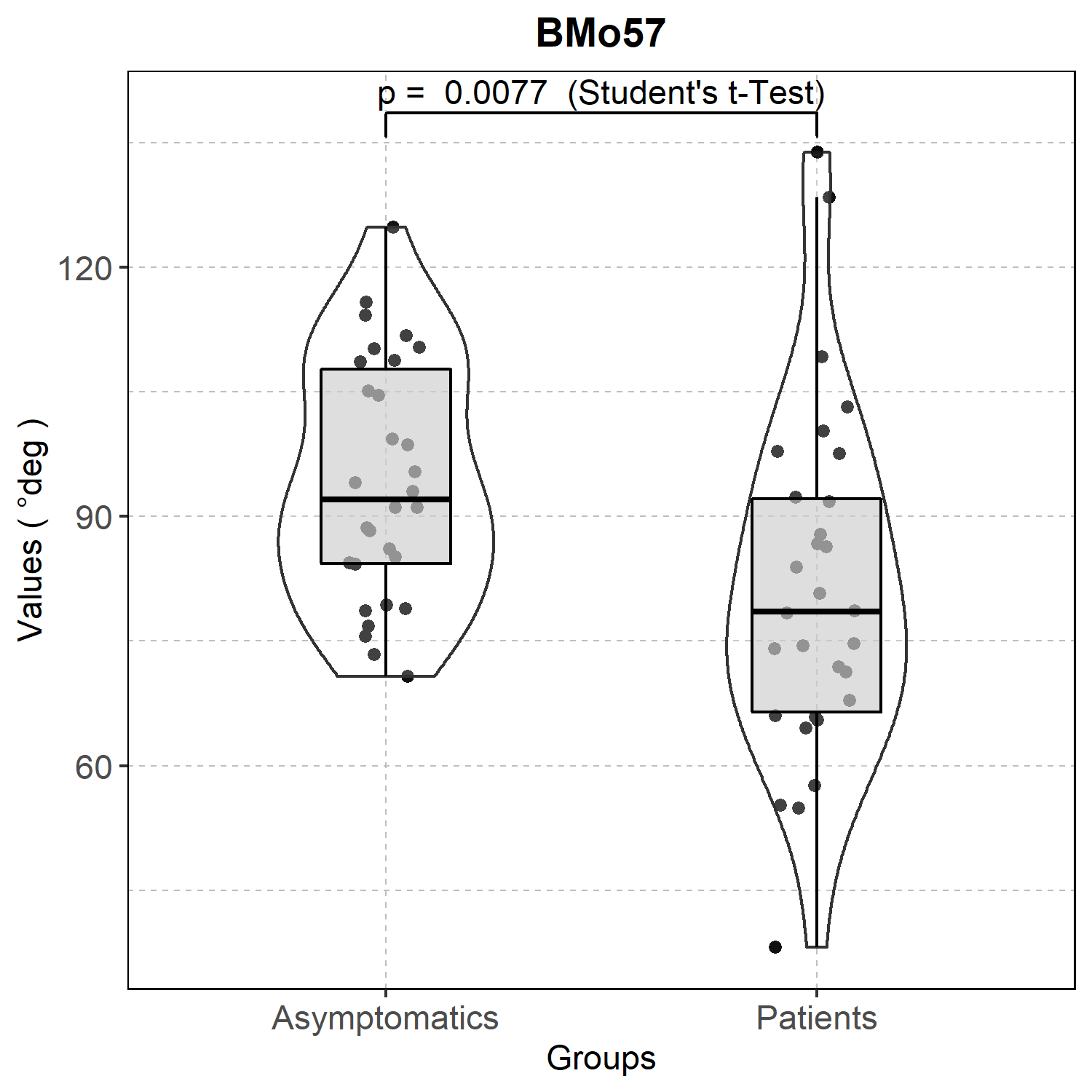

Supplement: Supplementary file 2 — Supplementary Information 2. [file 41598_2023_33504_MOESM2_ESM.zip › BMo057_boxplot.png]

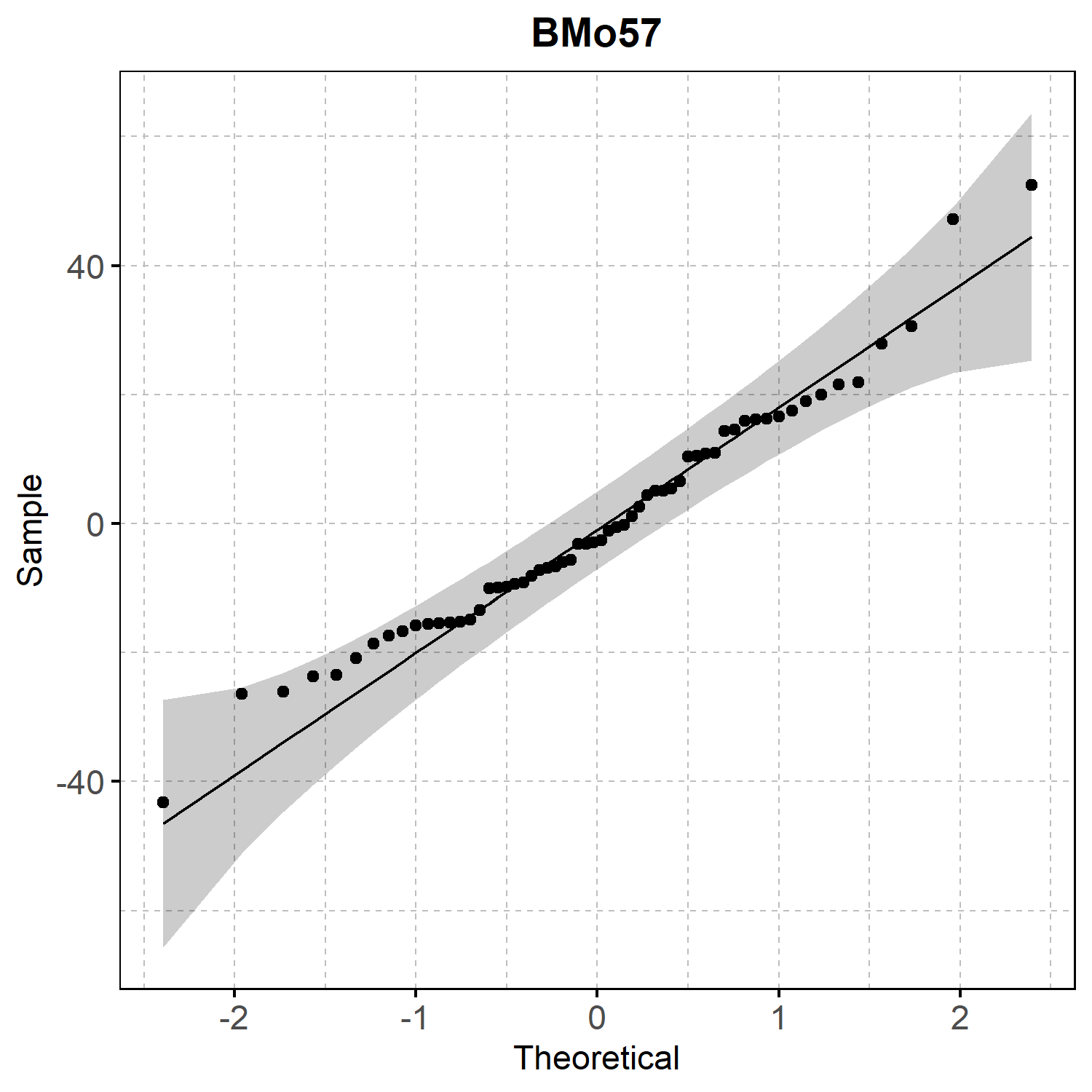

Supplement: Supplementary file 2 — Supplementary Information 2. [file 41598_2023_33504_MOESM2_ESM.zip › BMo057_normality.png]

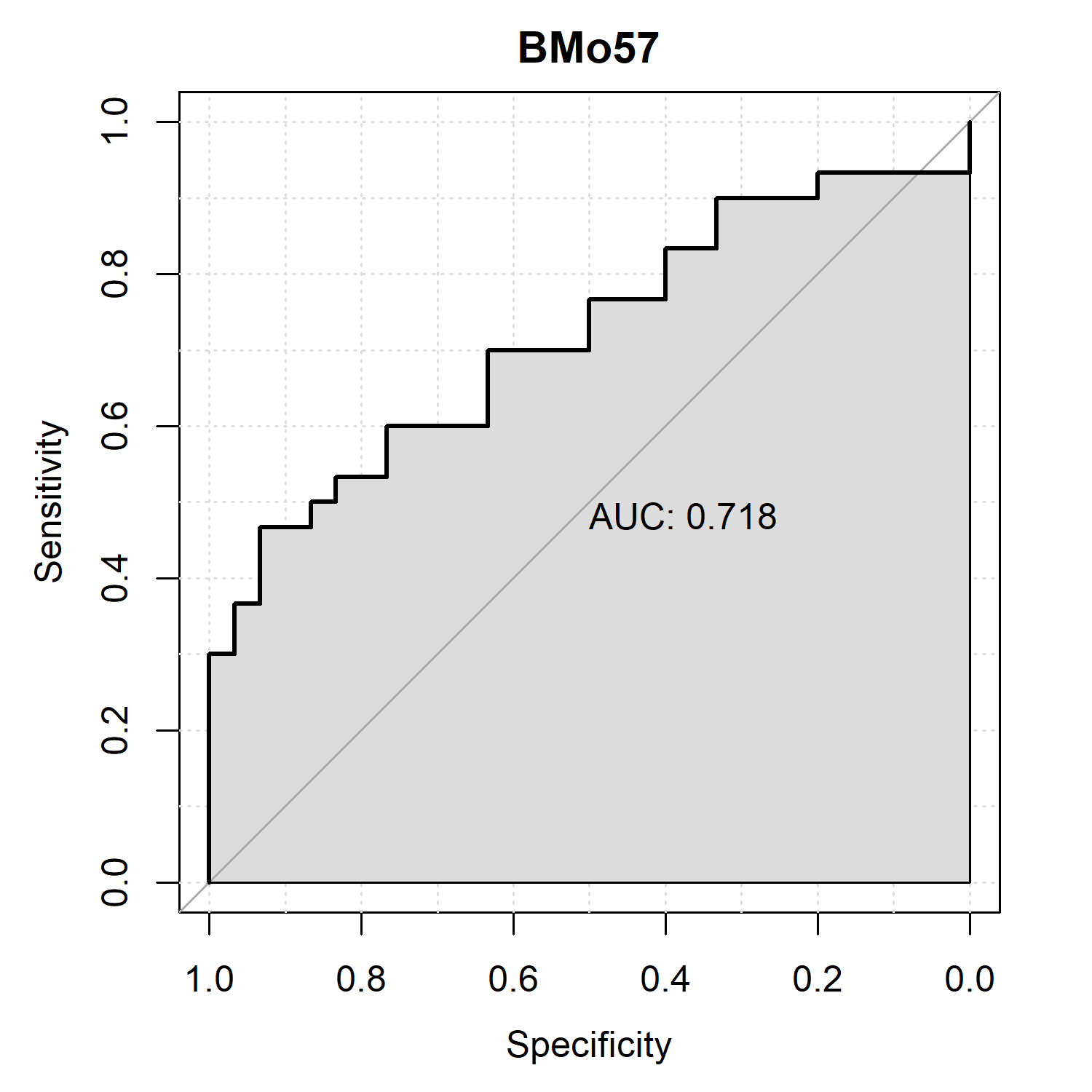

Supplement: Supplementary file 2 — Supplementary Information 2. [file 41598_2023_33504_MOESM2_ESM.zip › BMo057_ROC.png]

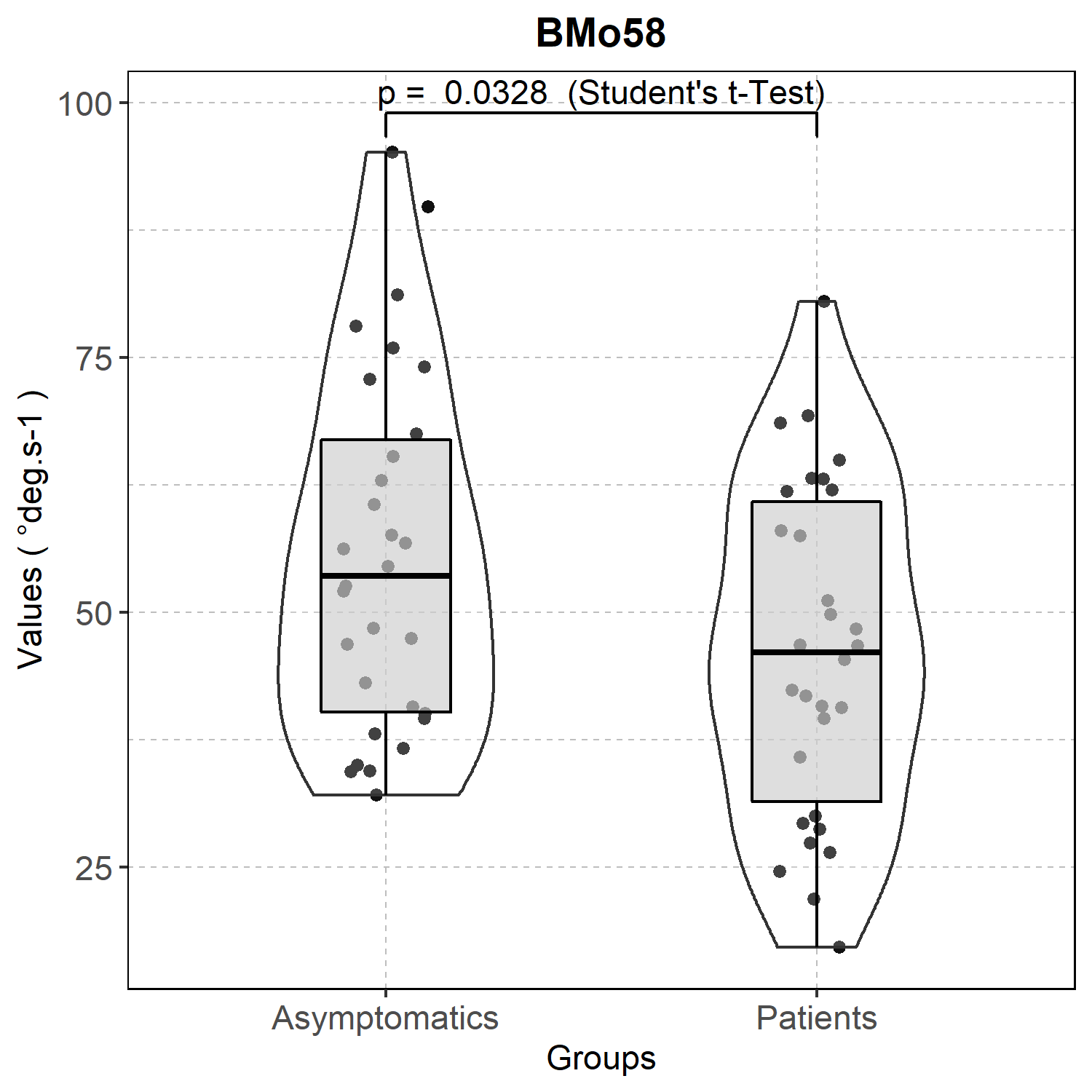

Supplement: Supplementary file 2 — Supplementary Information 2. [file 41598_2023_33504_MOESM2_ESM.zip › BMo058_boxplot.png]

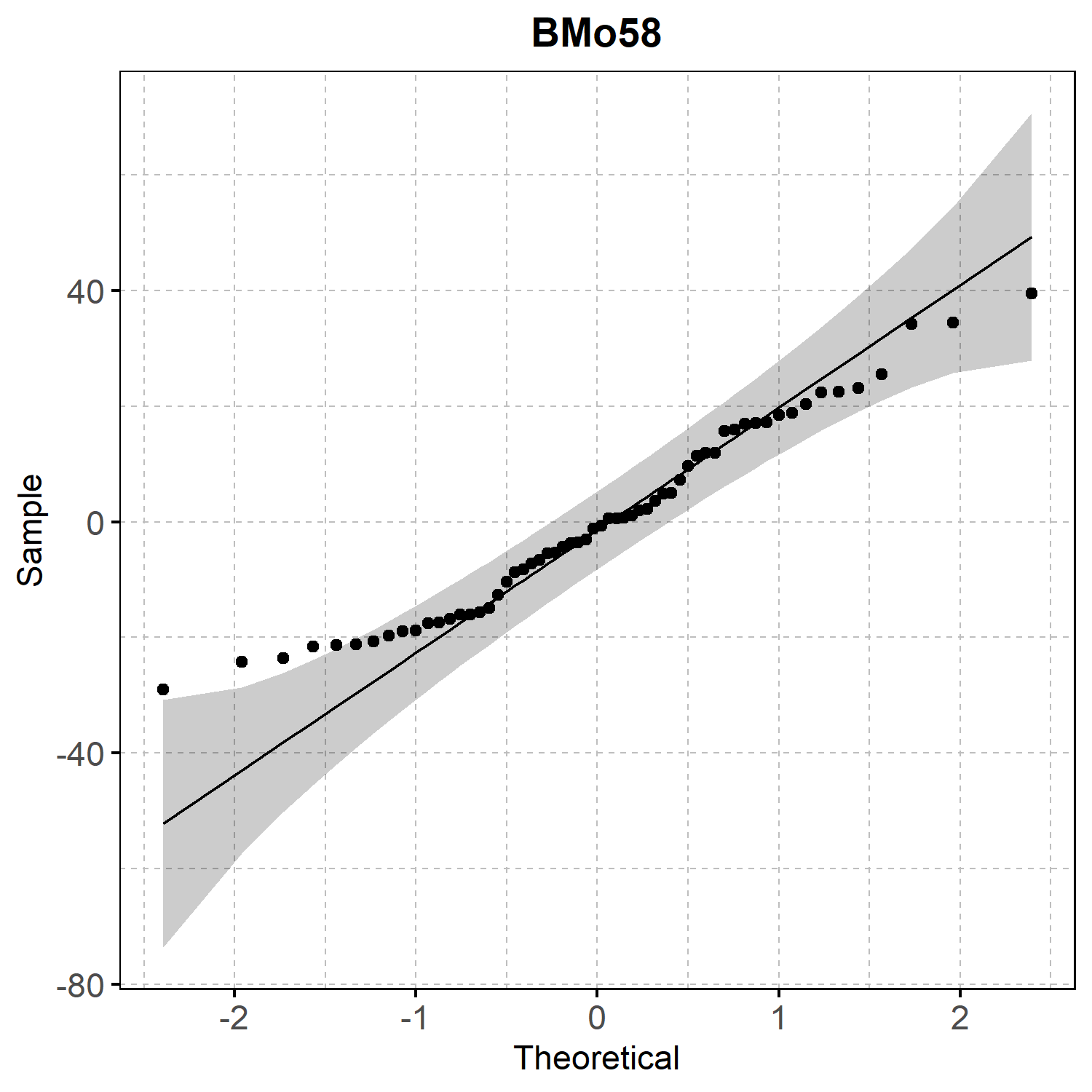

Supplement: Supplementary file 2 — Supplementary Information 2. [file 41598_2023_33504_MOESM2_ESM.zip › BMo058_normality.png]

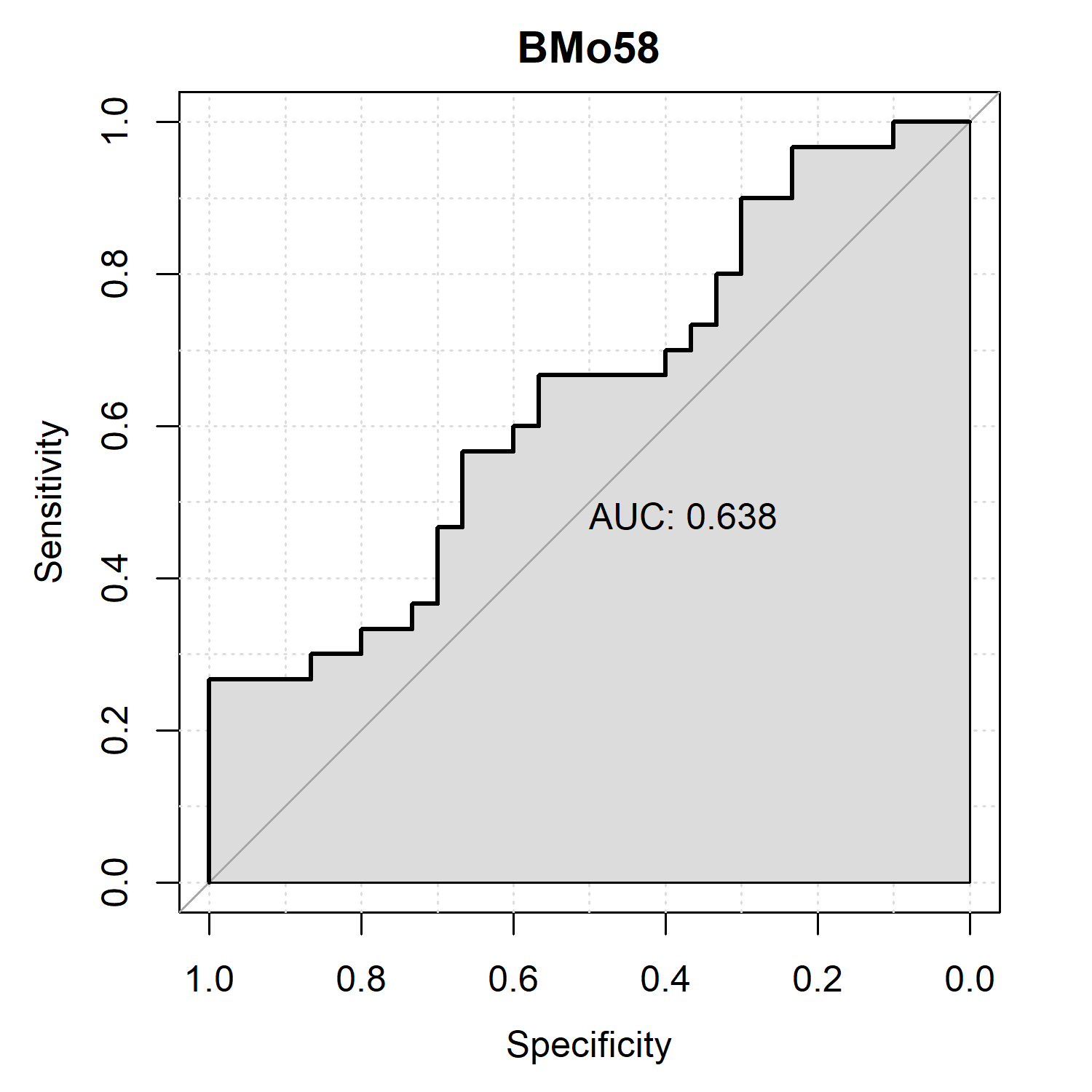

Supplement: Supplementary file 2 — Supplementary Information 2. [file 41598_2023_33504_MOESM2_ESM.zip › BMo058_ROC.png]

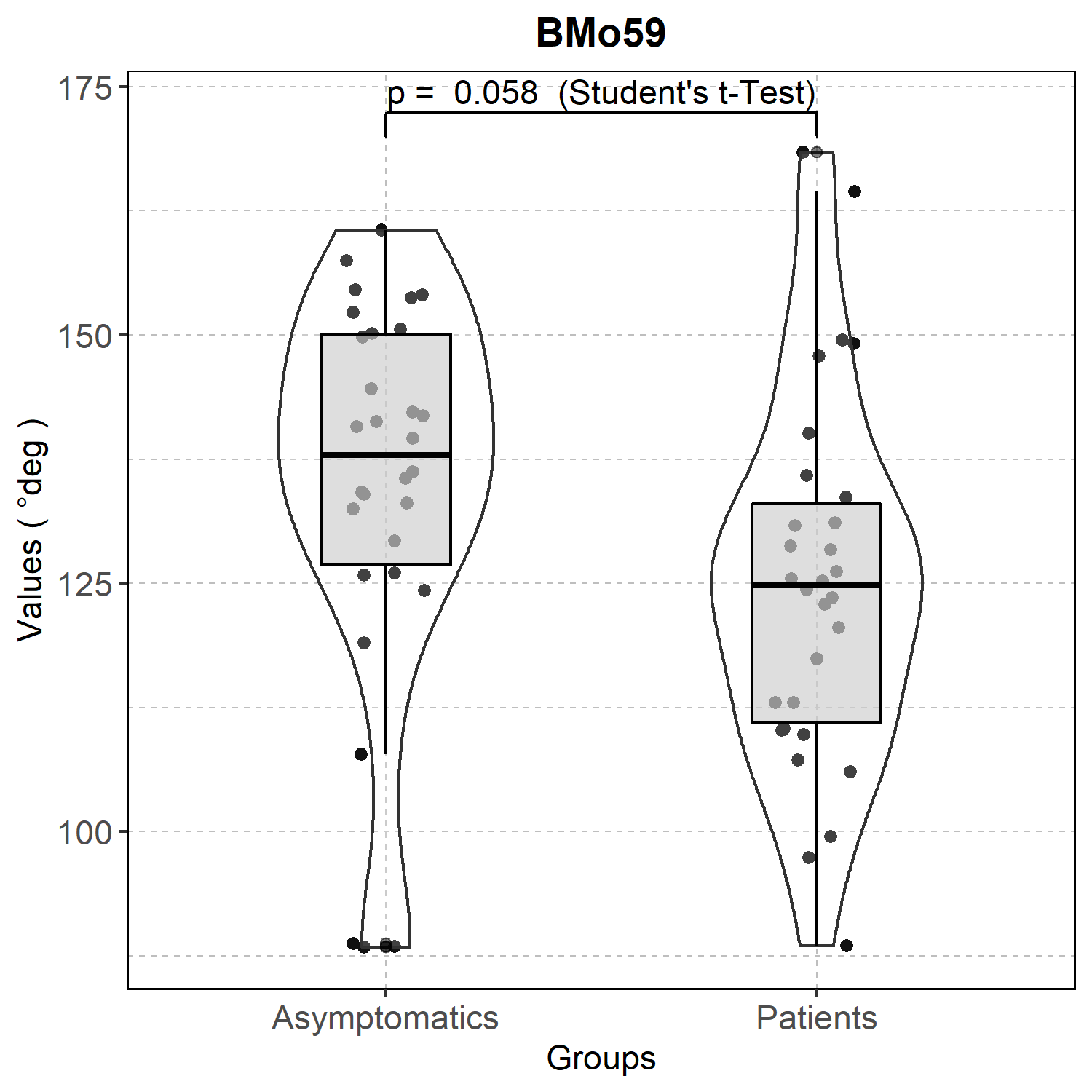

Supplement: Supplementary file 2 — Supplementary Information 2. [file 41598_2023_33504_MOESM2_ESM.zip › BMo059_boxplot.png]

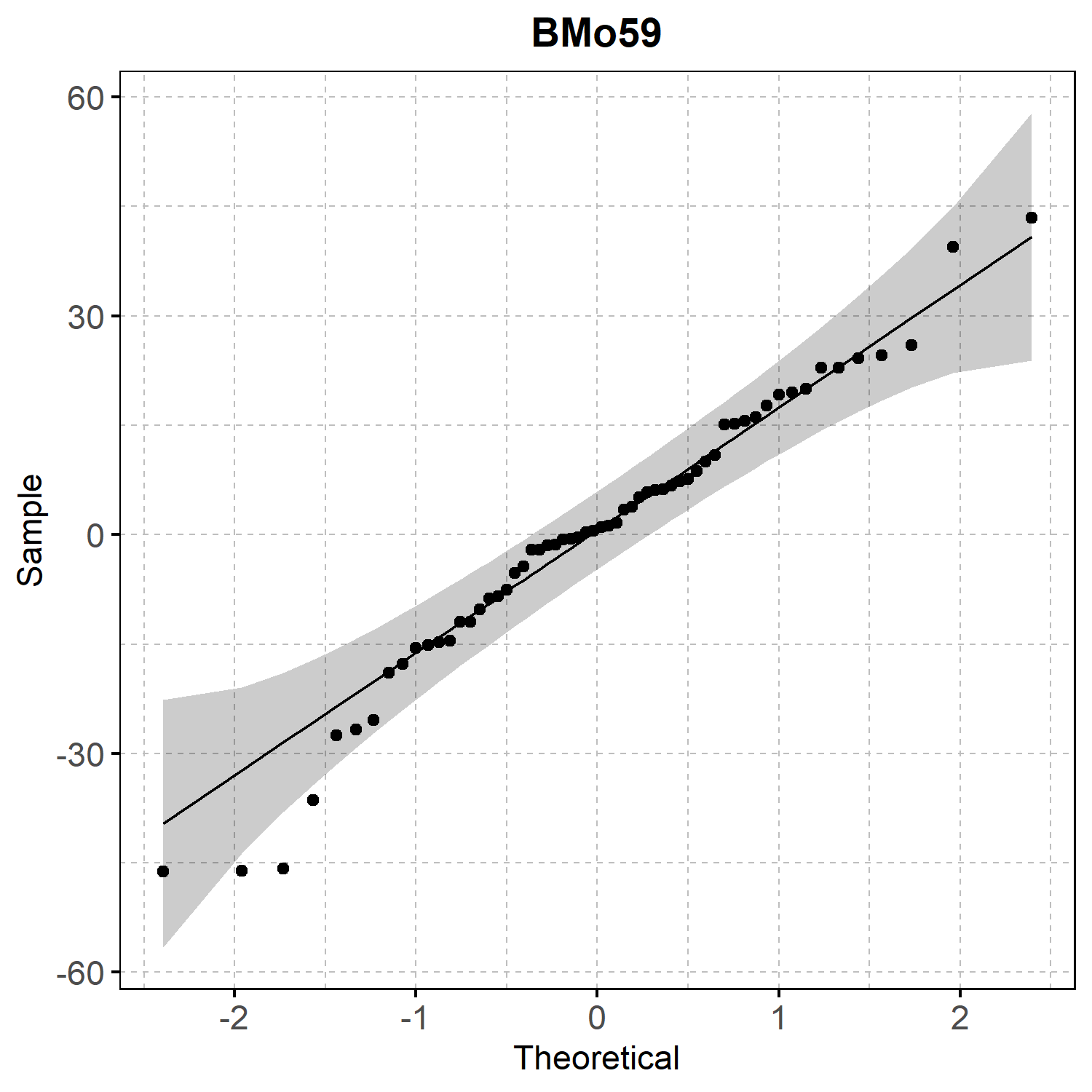

Supplement: Supplementary file 2 — Supplementary Information 2. [file 41598_2023_33504_MOESM2_ESM.zip › BMo059_normality.png]

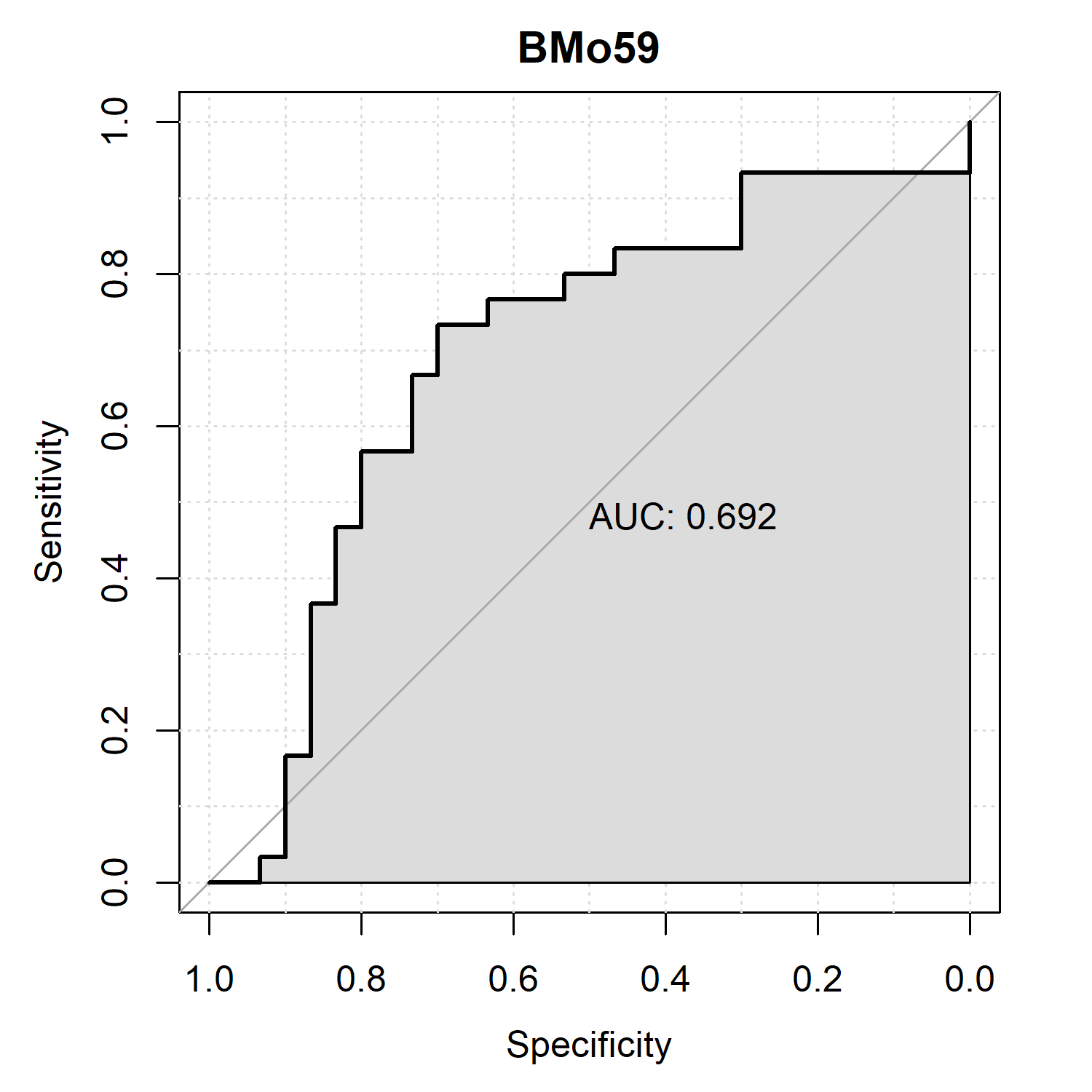

Supplement: Supplementary file 2 — Supplementary Information 2. [file 41598_2023_33504_MOESM2_ESM.zip › BMo059_ROC.png]

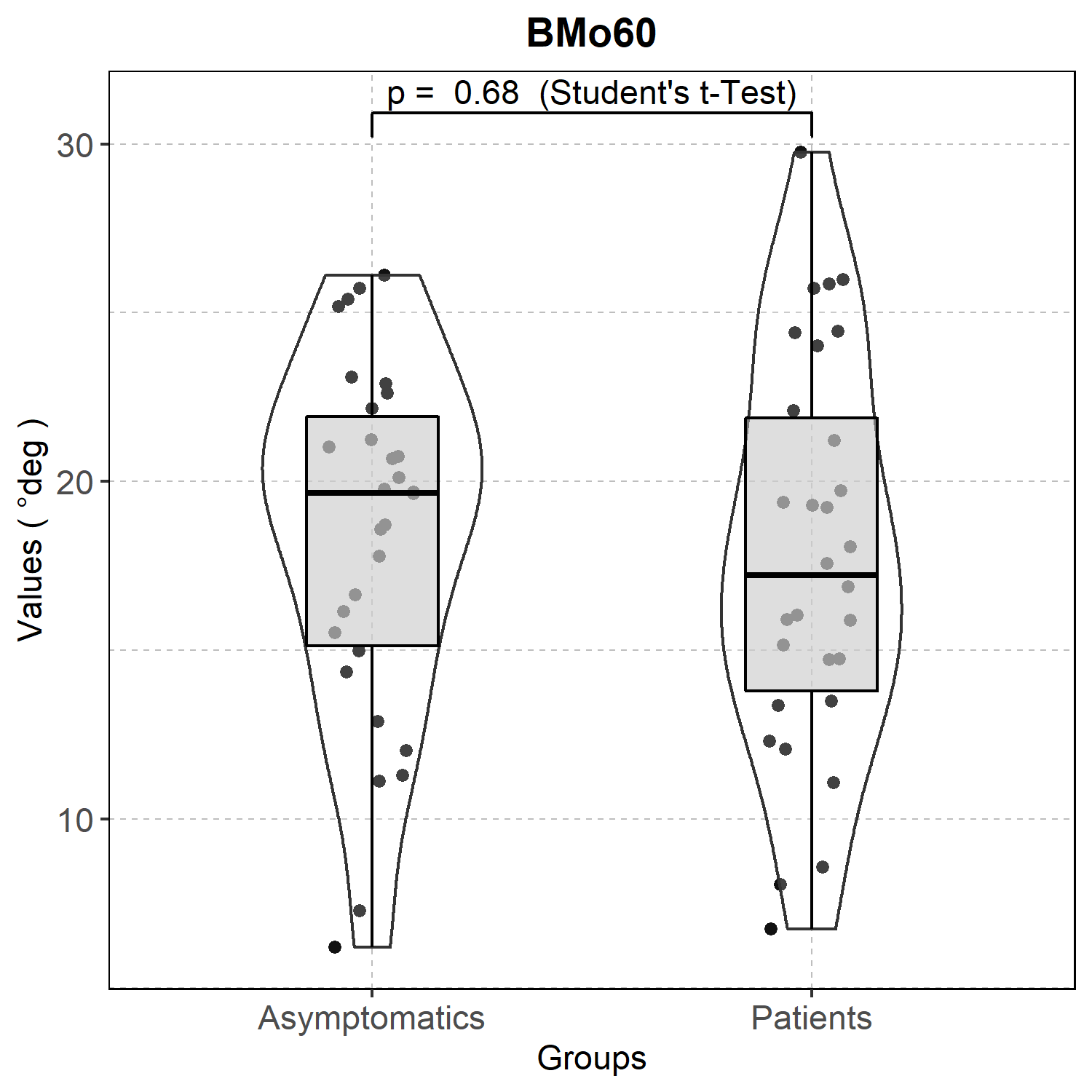

Supplement: Supplementary file 2 — Supplementary Information 2. [file 41598_2023_33504_MOESM2_ESM.zip › BMo060_boxplot.png]

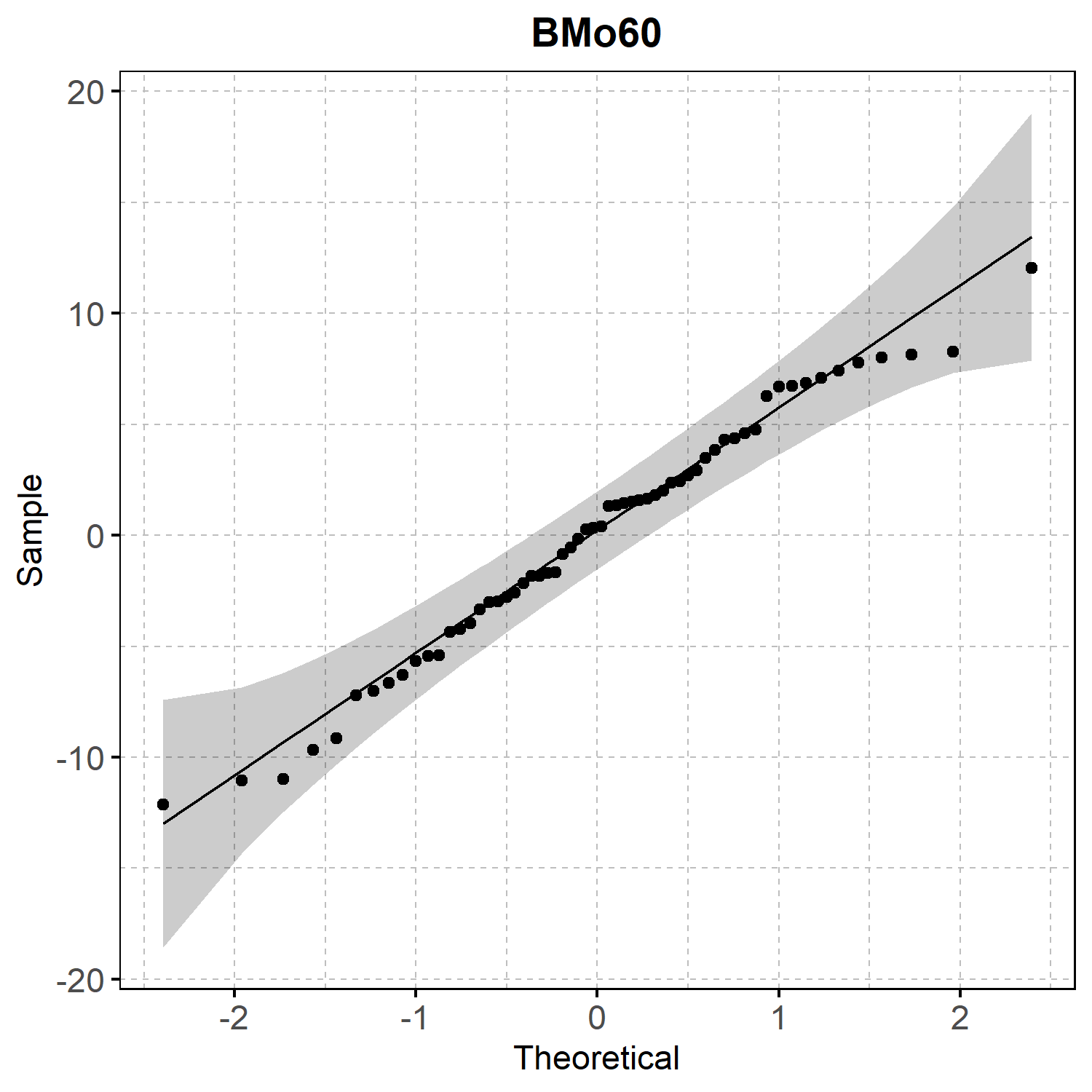

Supplement: Supplementary file 2 — Supplementary Information 2. [file 41598_2023_33504_MOESM2_ESM.zip › BMo060_normality.png]

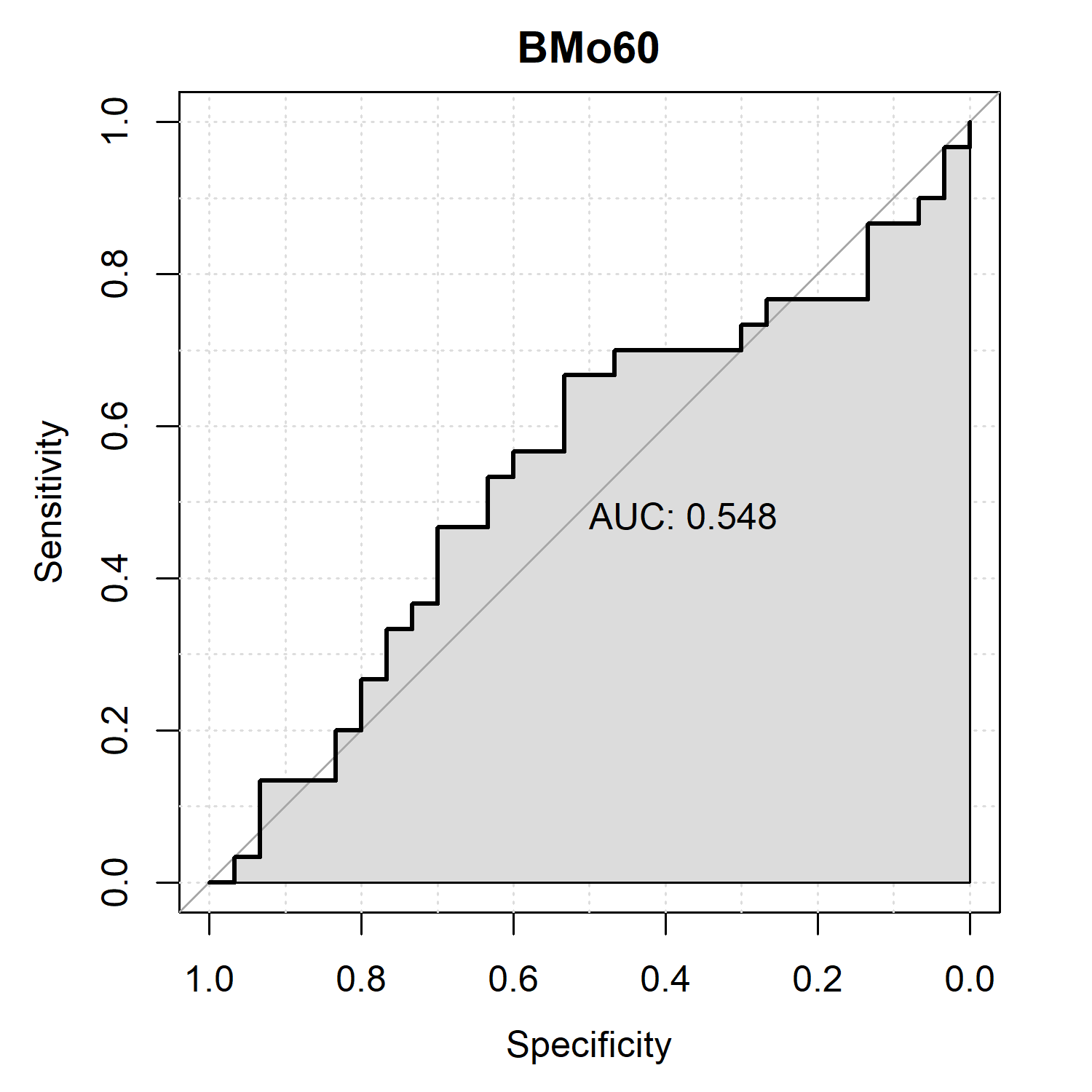

Supplement: Supplementary file 2 — Supplementary Information 2. [file 41598_2023_33504_MOESM2_ESM.zip › BMo060_ROC.png]

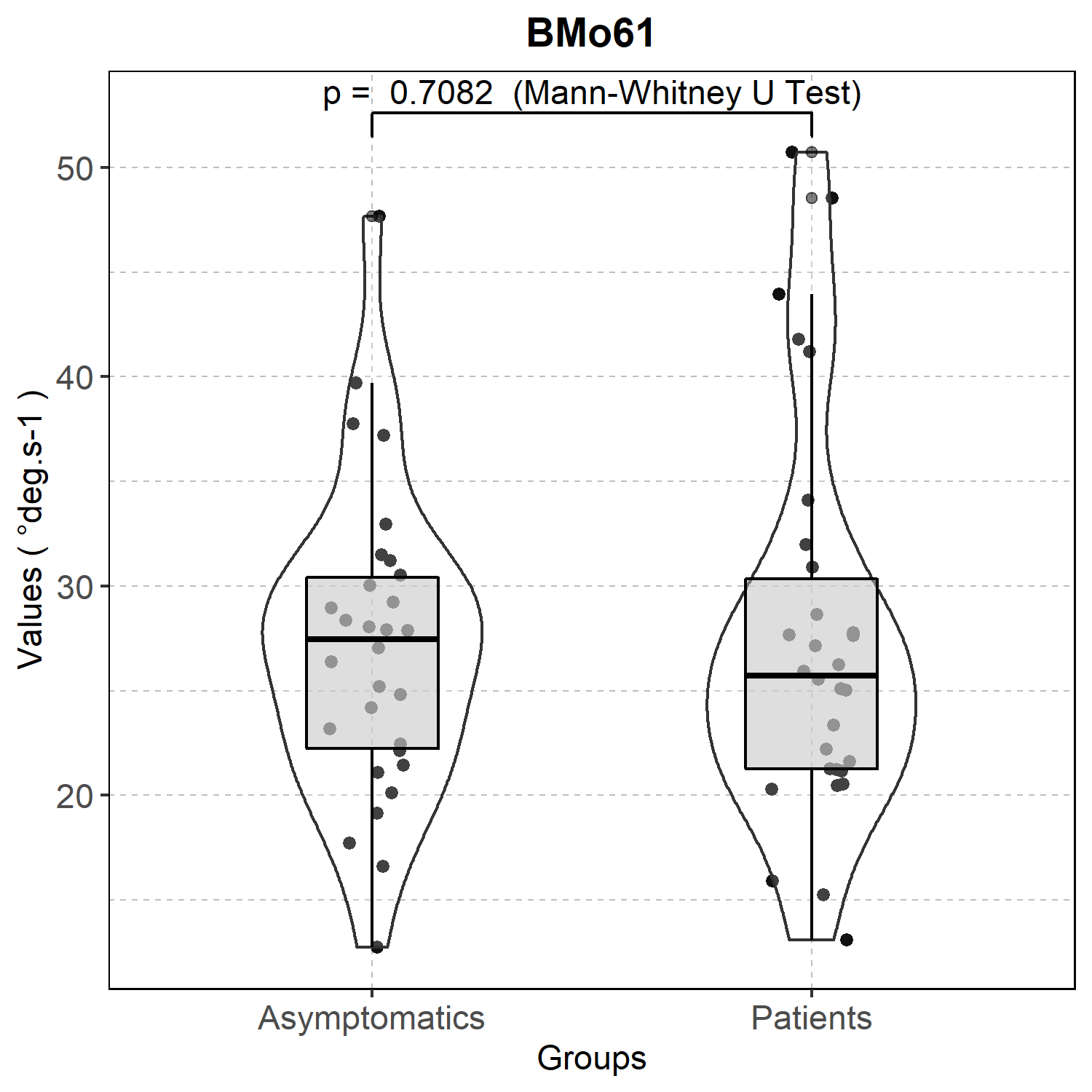

Supplement: Supplementary file 2 — Supplementary Information 2. [file 41598_2023_33504_MOESM2_ESM.zip › BMo061_boxplot.png]

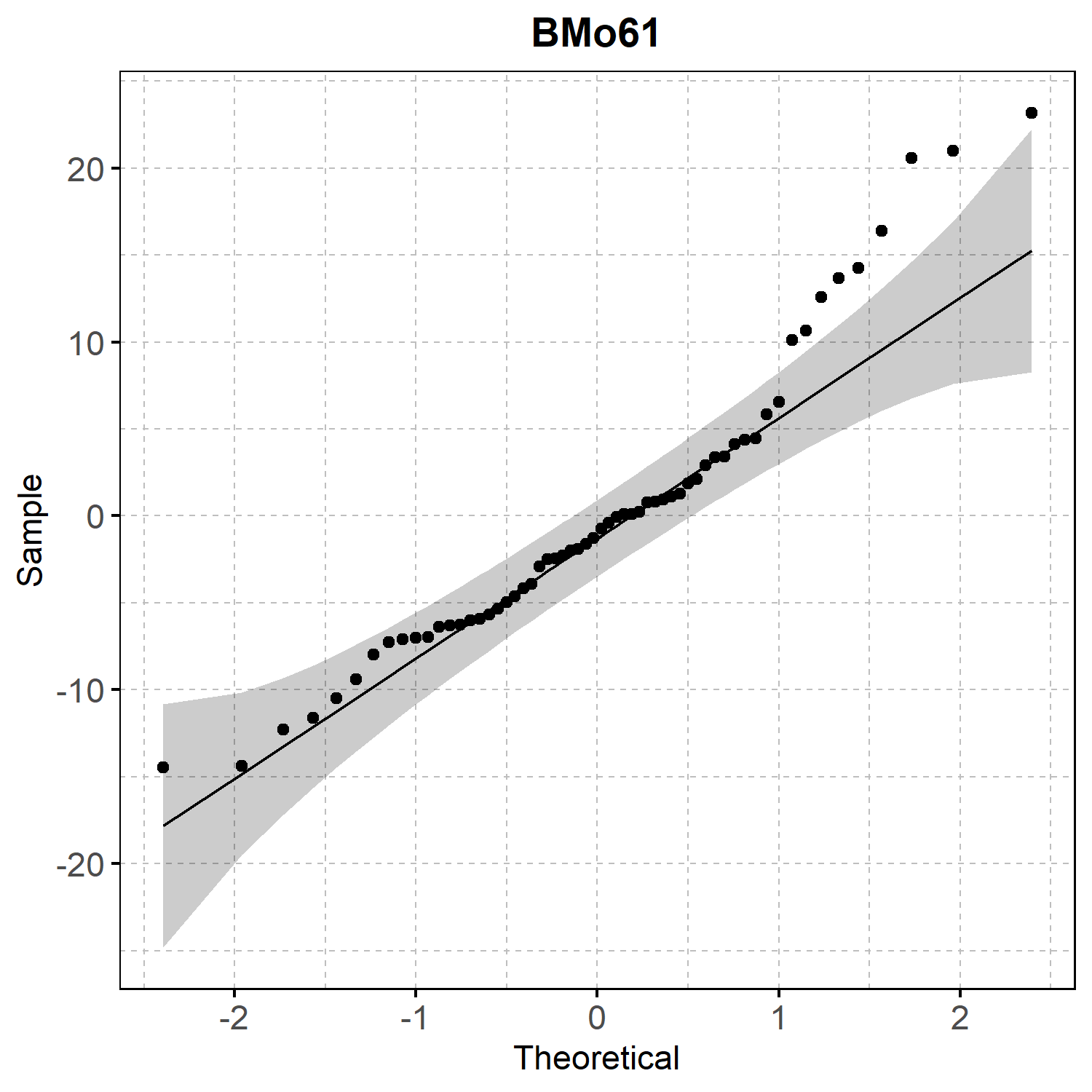

Supplement: Supplementary file 2 — Supplementary Information 2. [file 41598_2023_33504_MOESM2_ESM.zip › BMo061_normality.png]

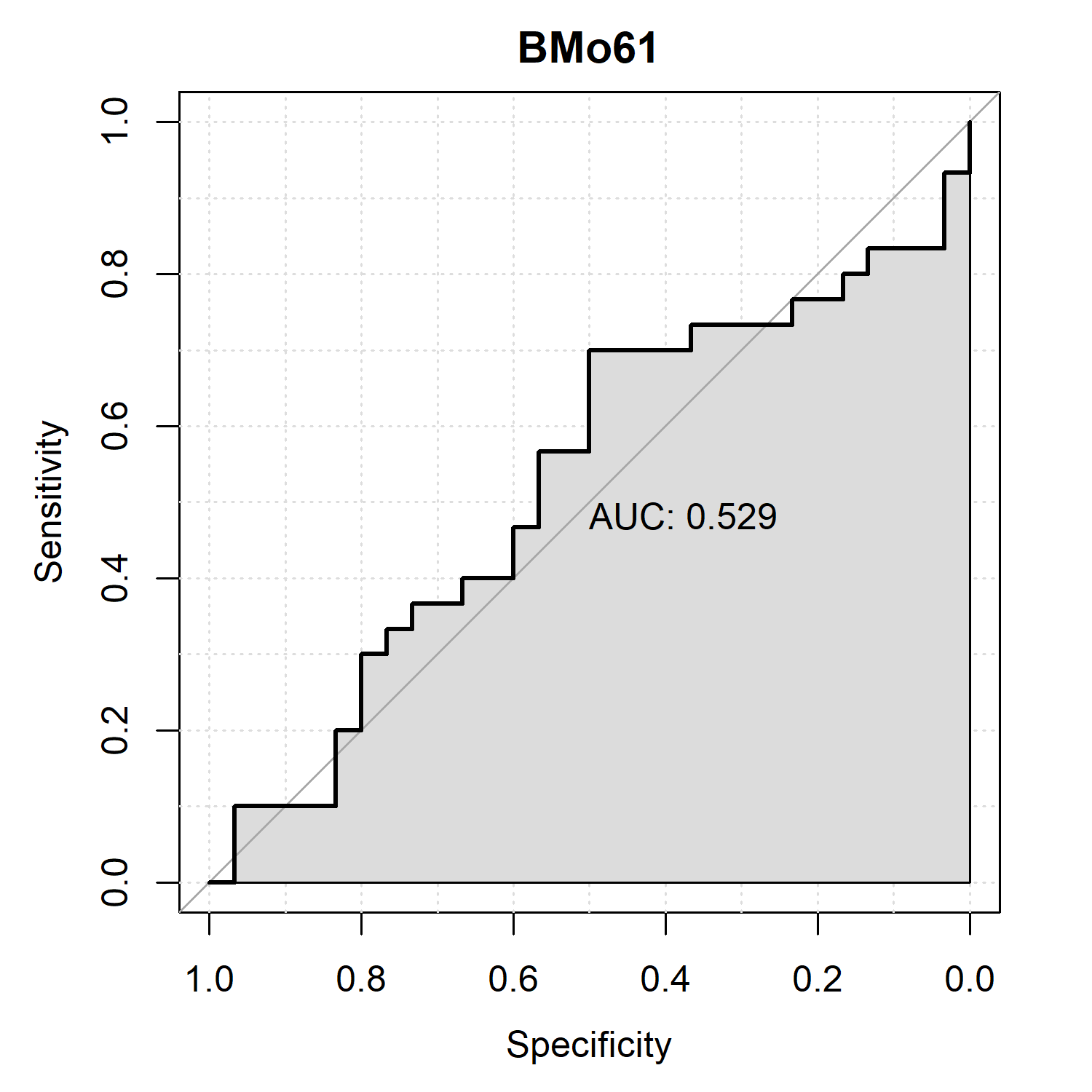

Supplement: Supplementary file 2 — Supplementary Information 2. [file 41598_2023_33504_MOESM2_ESM.zip › BMo061_ROC.png]

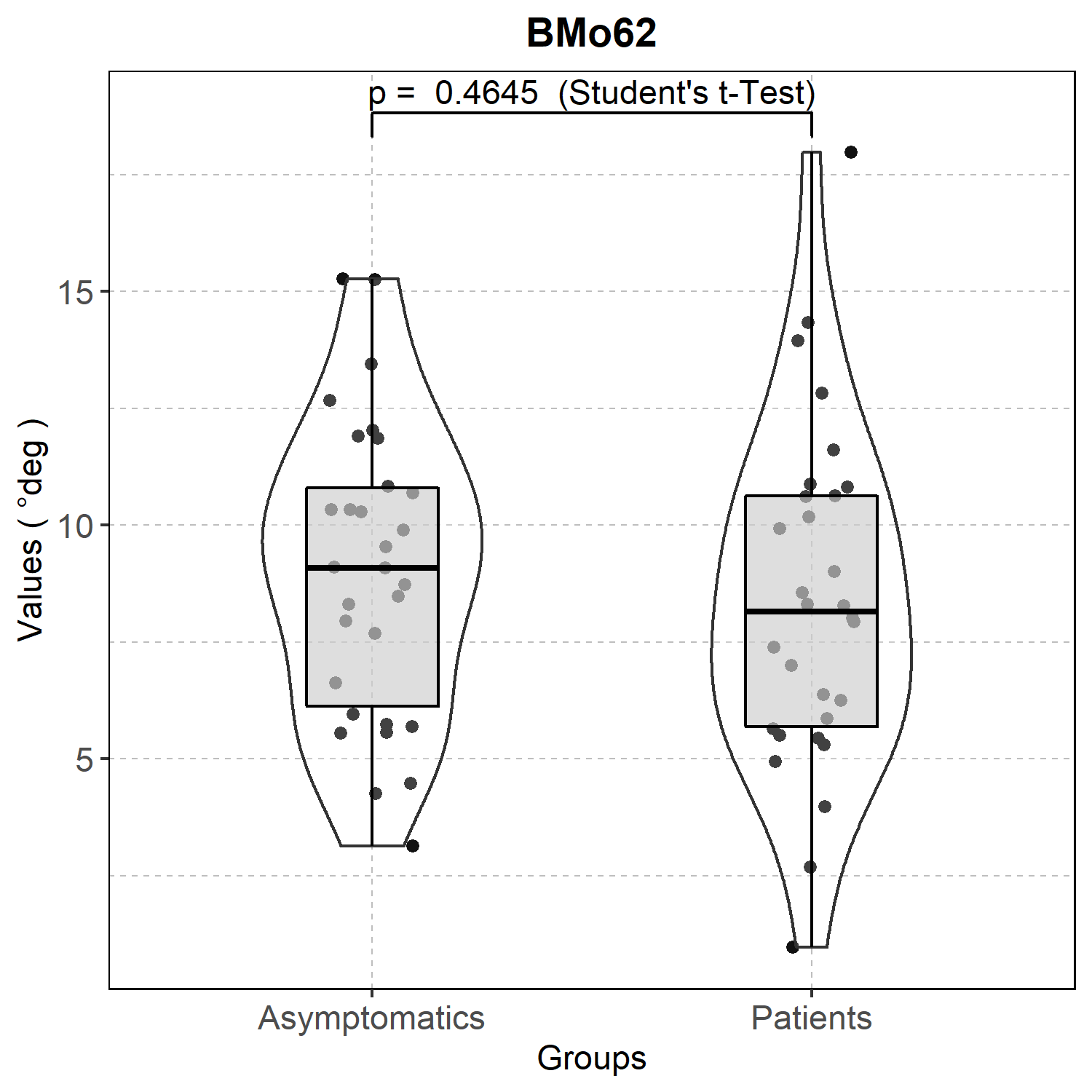

Supplement: Supplementary file 2 — Supplementary Information 2. [file 41598_2023_33504_MOESM2_ESM.zip › BMo062_boxplot.png]

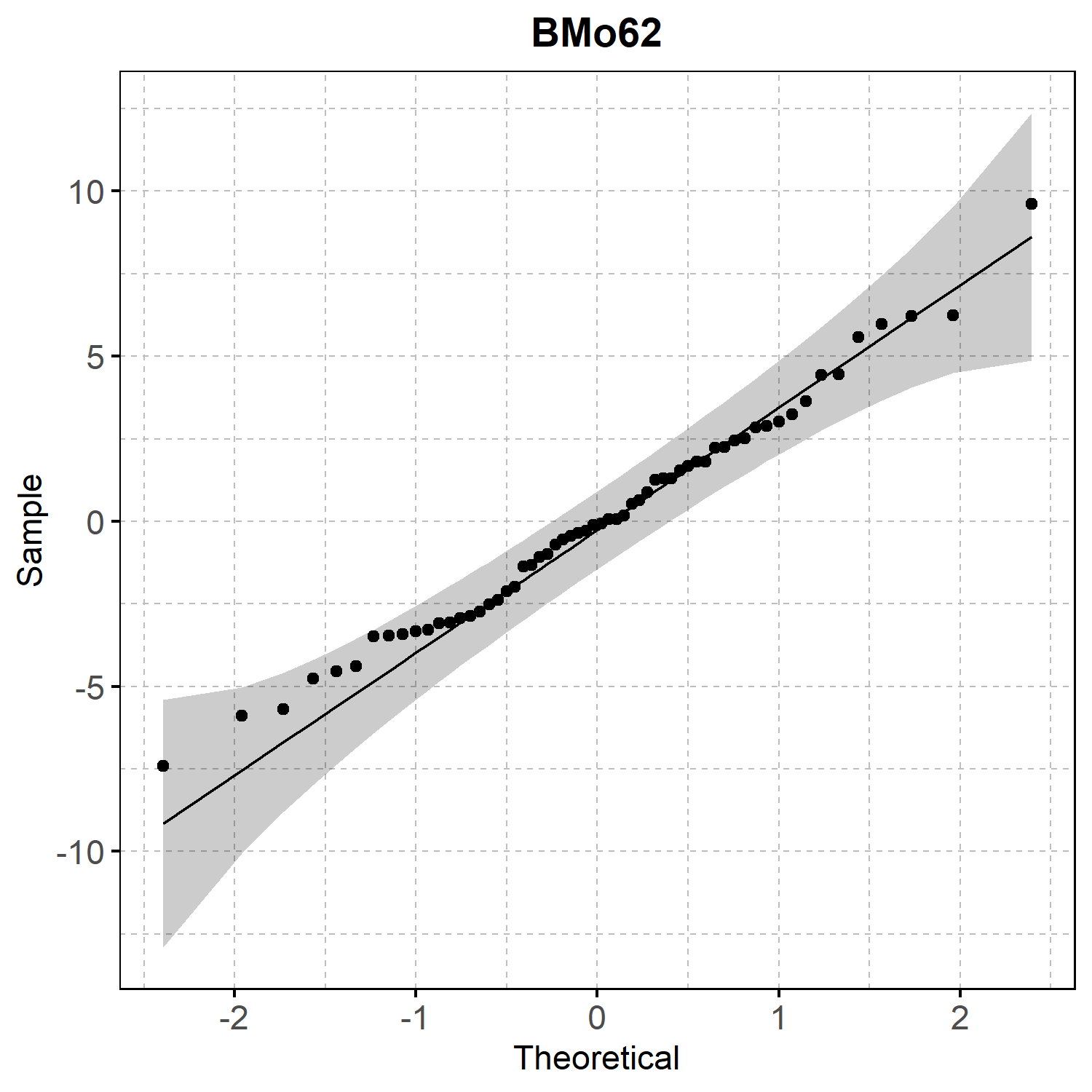

Supplement: Supplementary file 2 — Supplementary Information 2. [file 41598_2023_33504_MOESM2_ESM.zip › BMo062_normality.png]

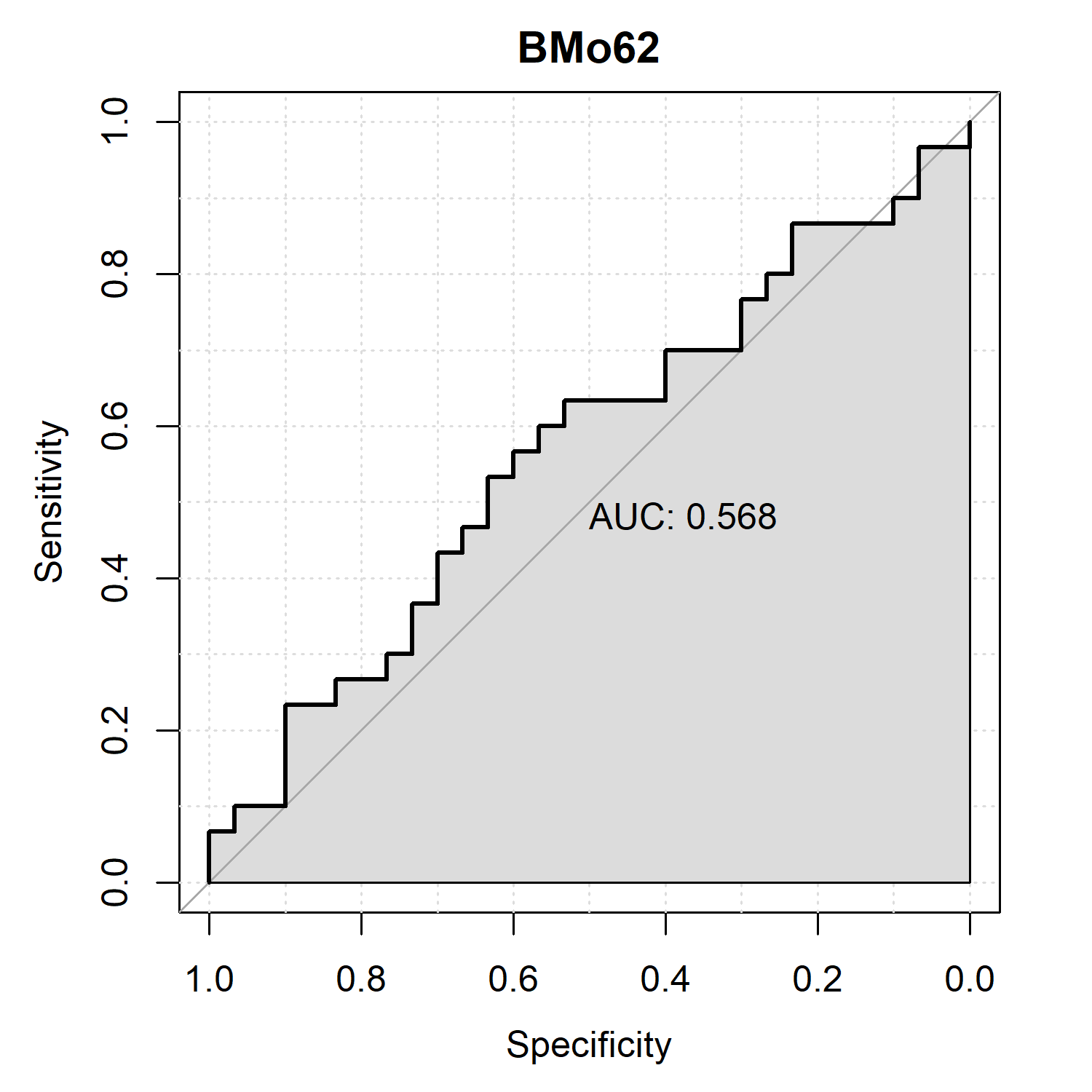

Supplement: Supplementary file 2 — Supplementary Information 2. [file 41598_2023_33504_MOESM2_ESM.zip › BMo062_ROC.png]

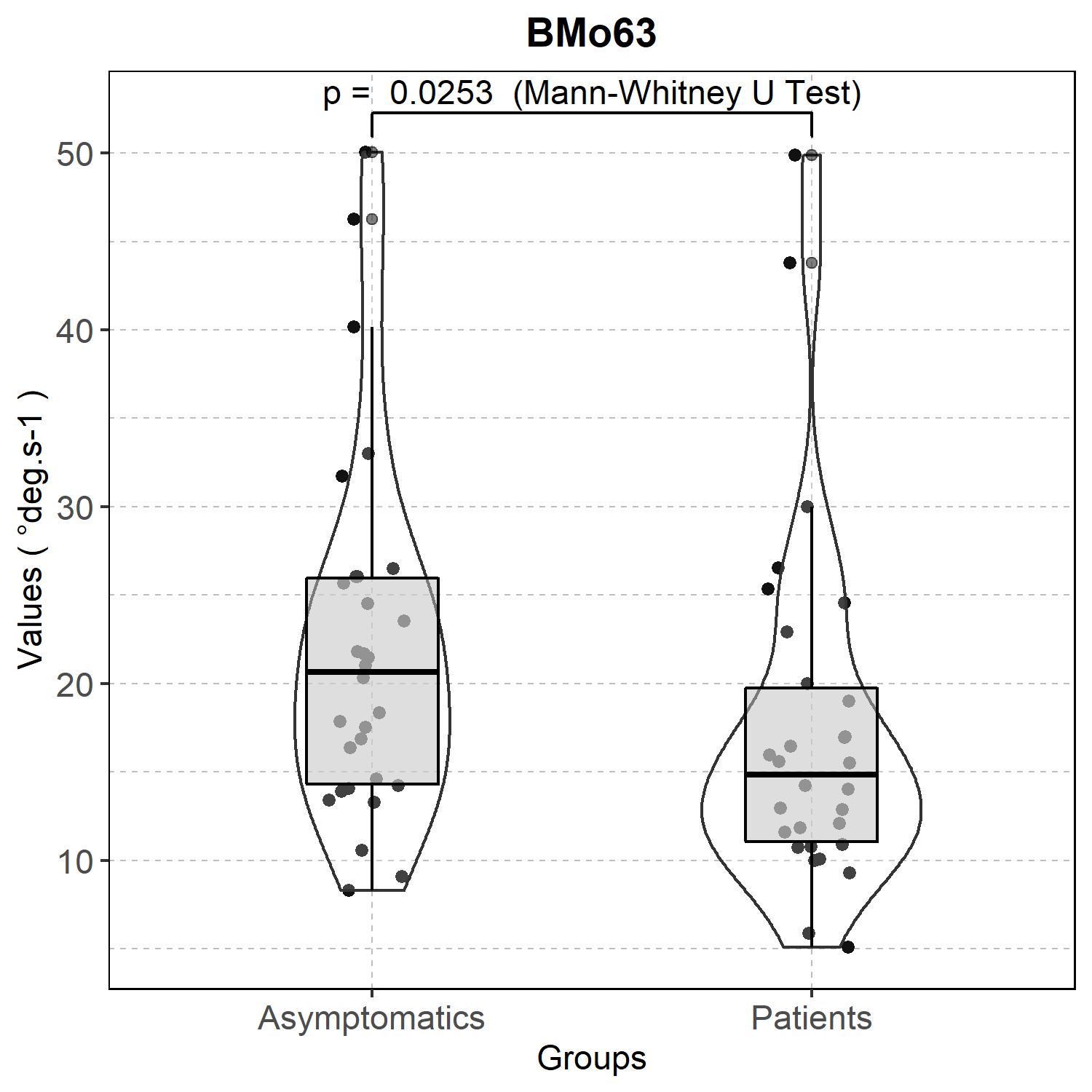

Supplement: Supplementary file 2 — Supplementary Information 2. [file 41598_2023_33504_MOESM2_ESM.zip › BMo063_boxplot.png]

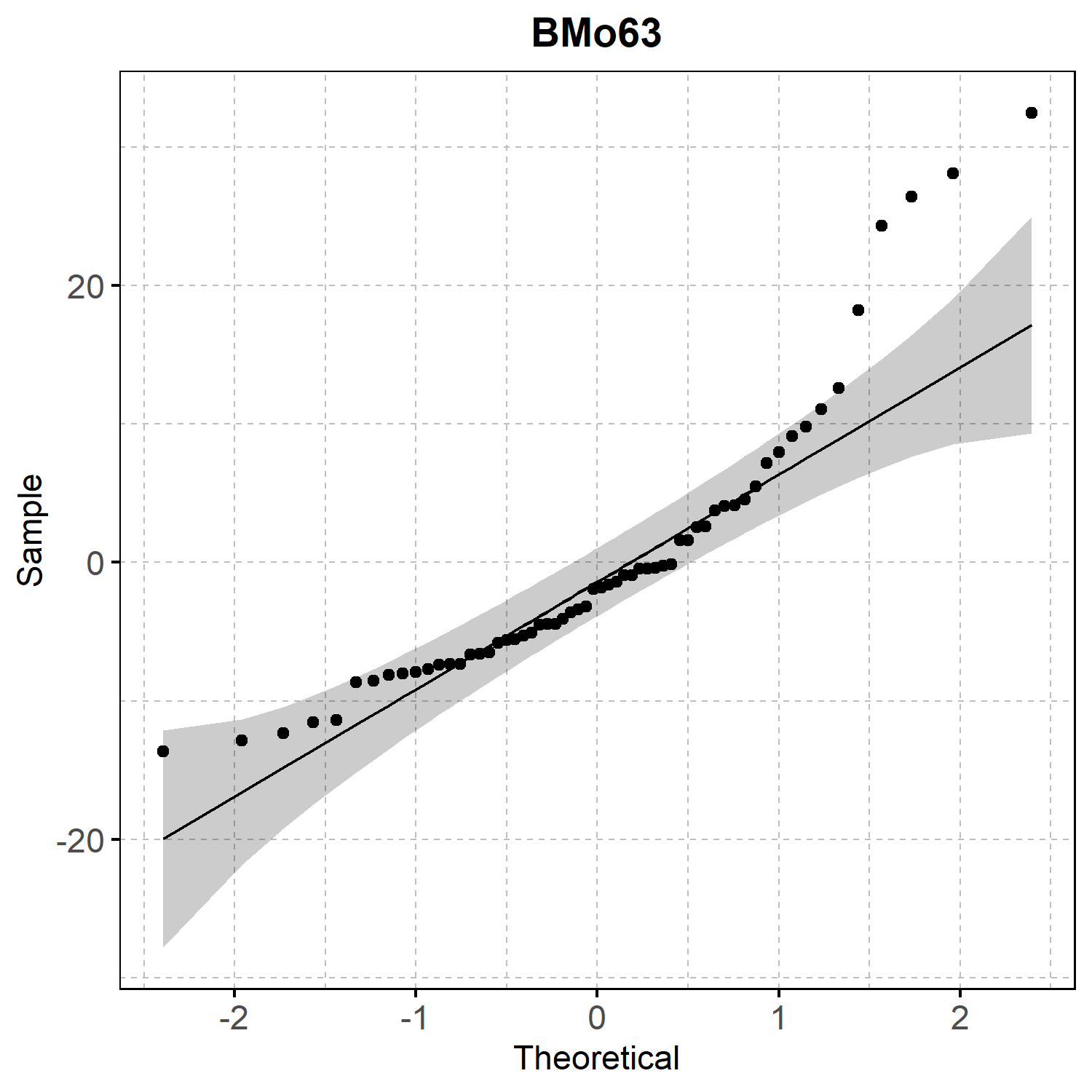

Supplement: Supplementary file 2 — Supplementary Information 2. [file 41598_2023_33504_MOESM2_ESM.zip › BMo063_normality.png]

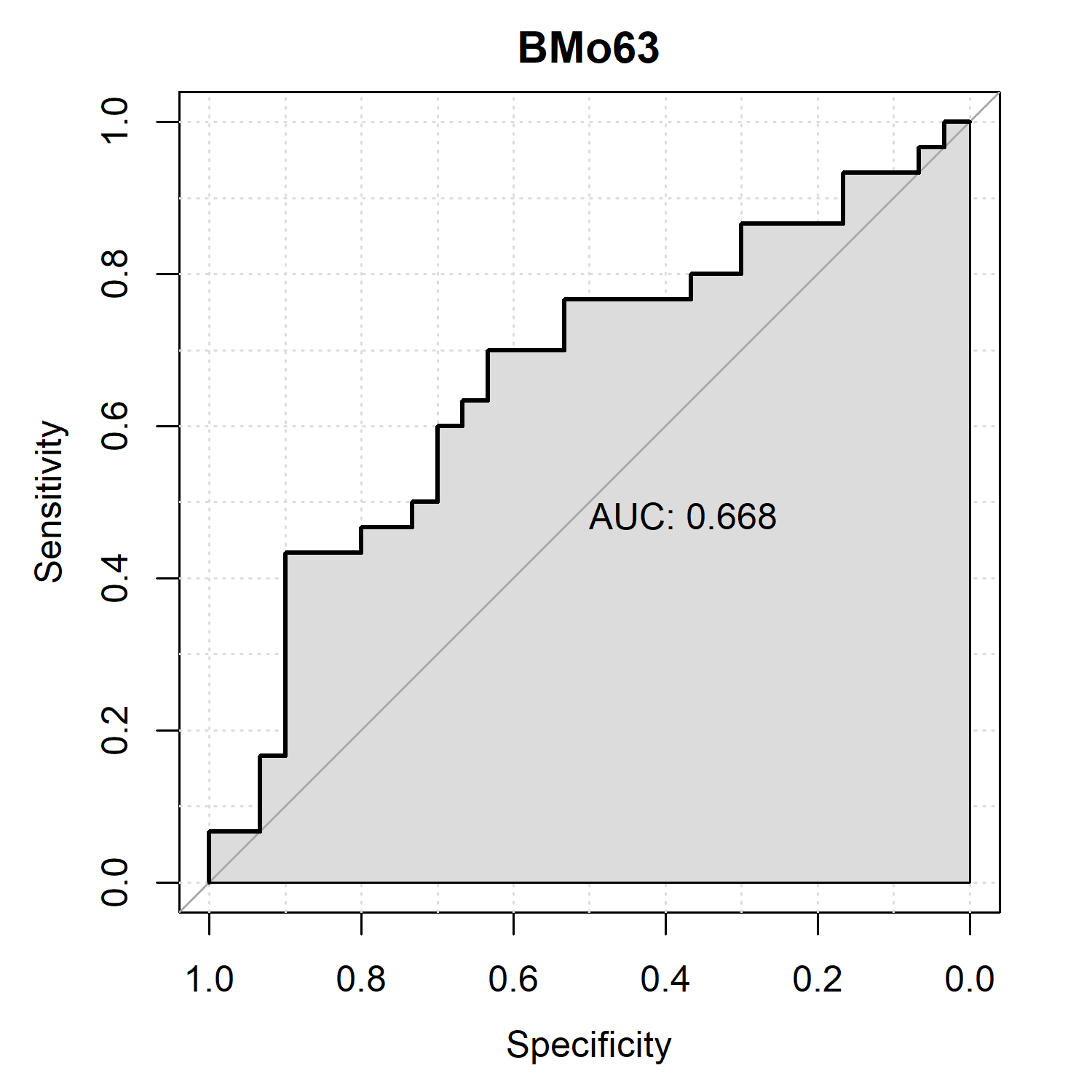

Supplement: Supplementary file 2 — Supplementary Information 2. [file 41598_2023_33504_MOESM2_ESM.zip › BMo063_ROC.png]

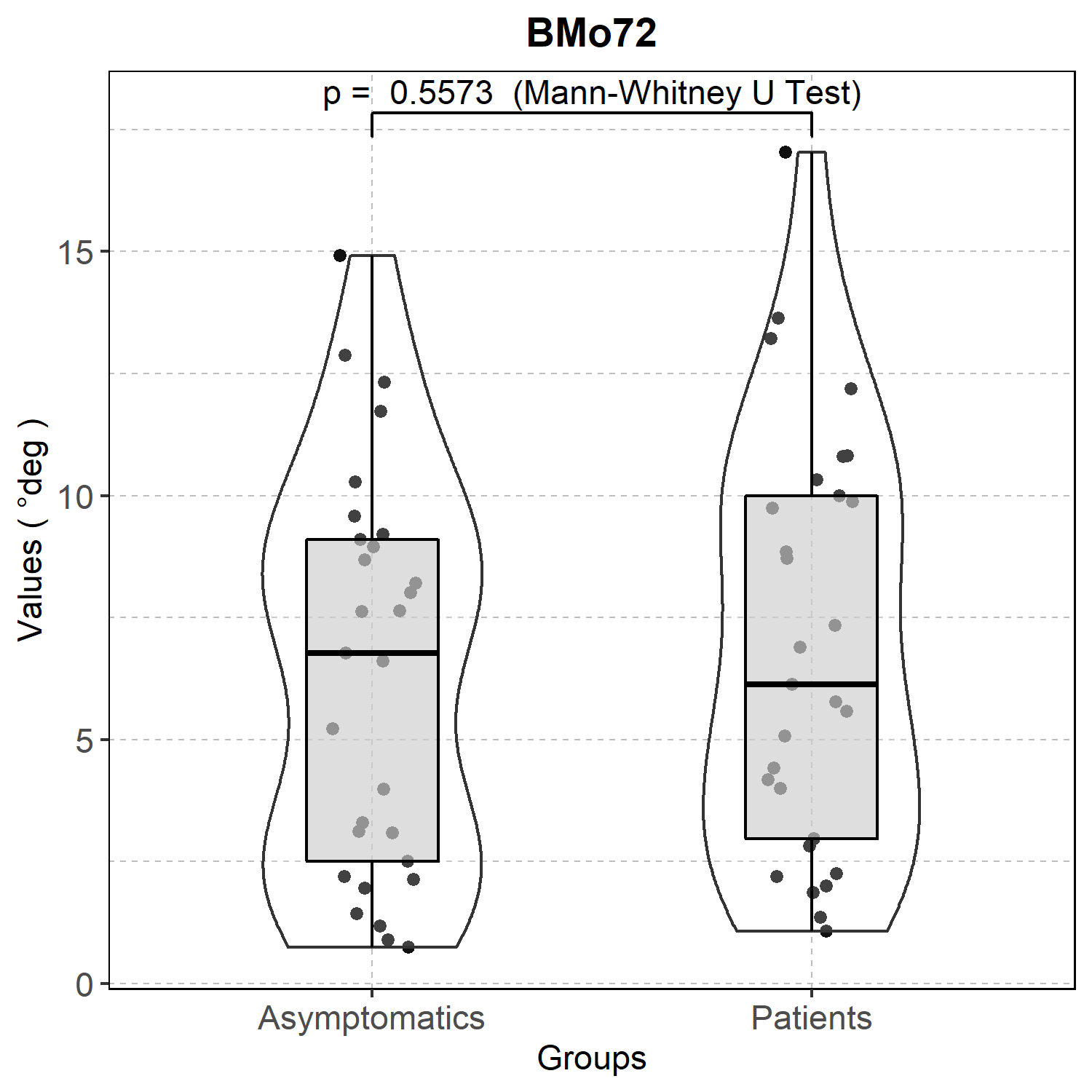

Supplement: Supplementary file 2 — Supplementary Information 2. [file 41598_2023_33504_MOESM2_ESM.zip › BMo072_boxplot.png]

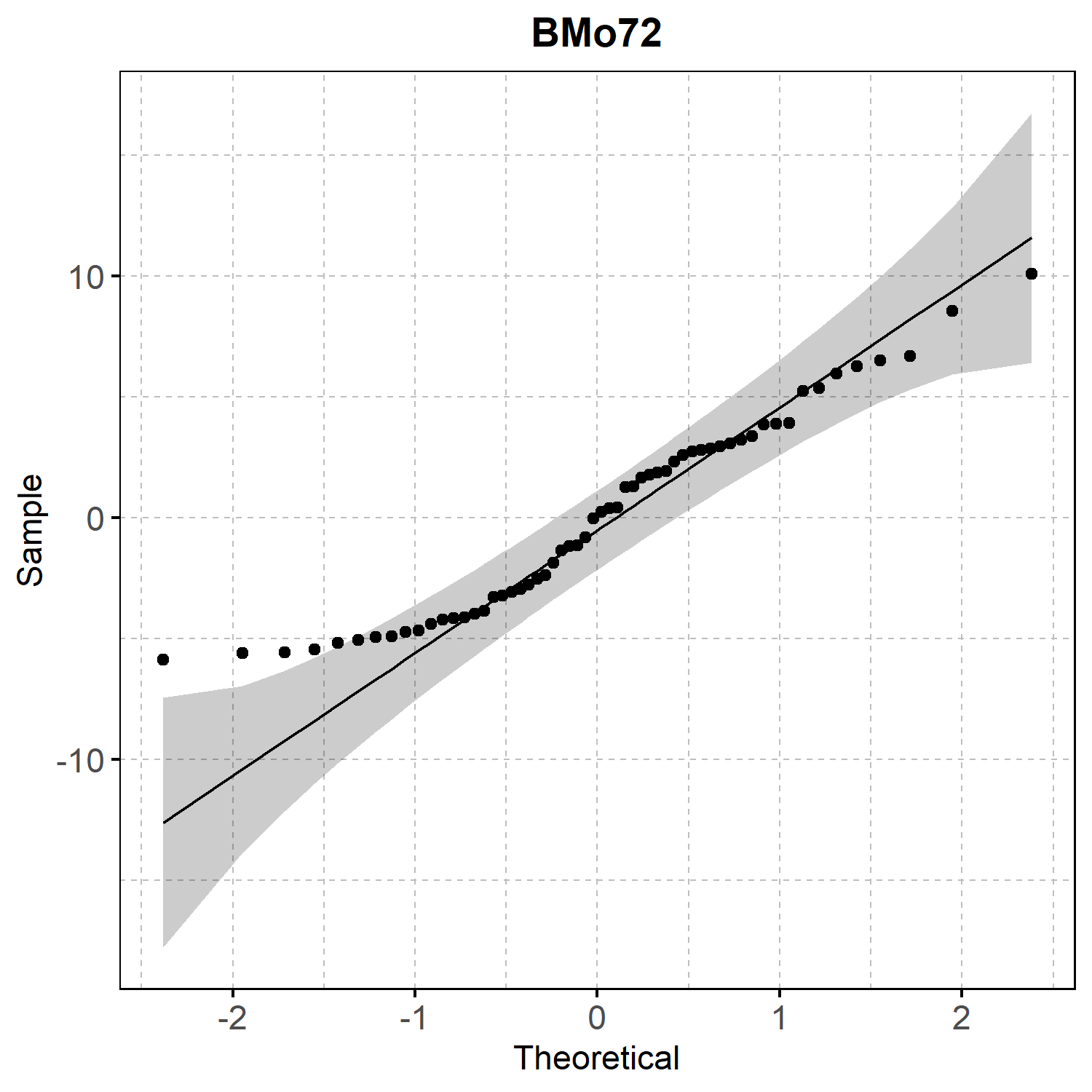

Supplement: Supplementary file 2 — Supplementary Information 2. [file 41598_2023_33504_MOESM2_ESM.zip › BMo072_normality.png]

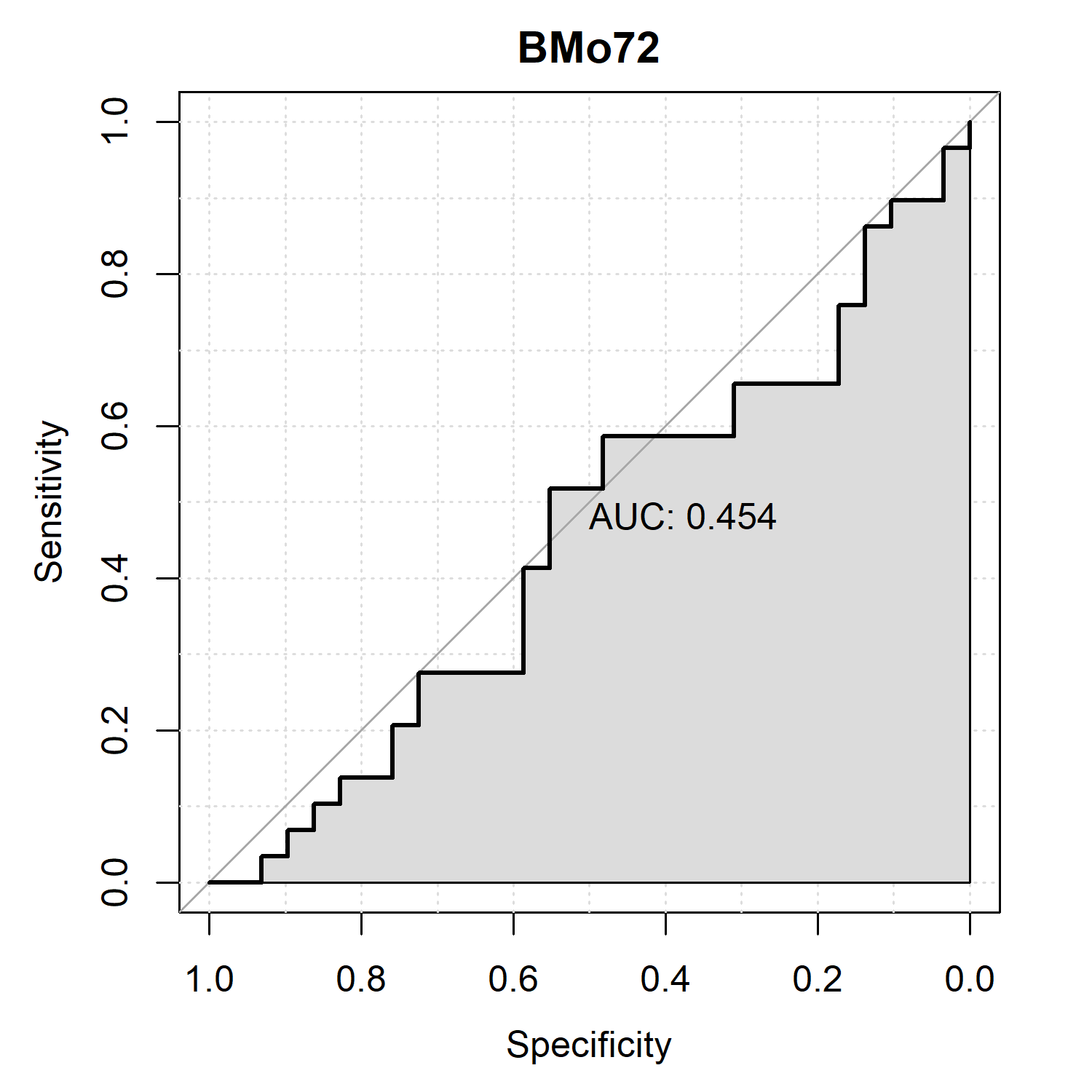

Supplement: Supplementary file 2 — Supplementary Information 2. [file 41598_2023_33504_MOESM2_ESM.zip › BMo072_ROC.png]

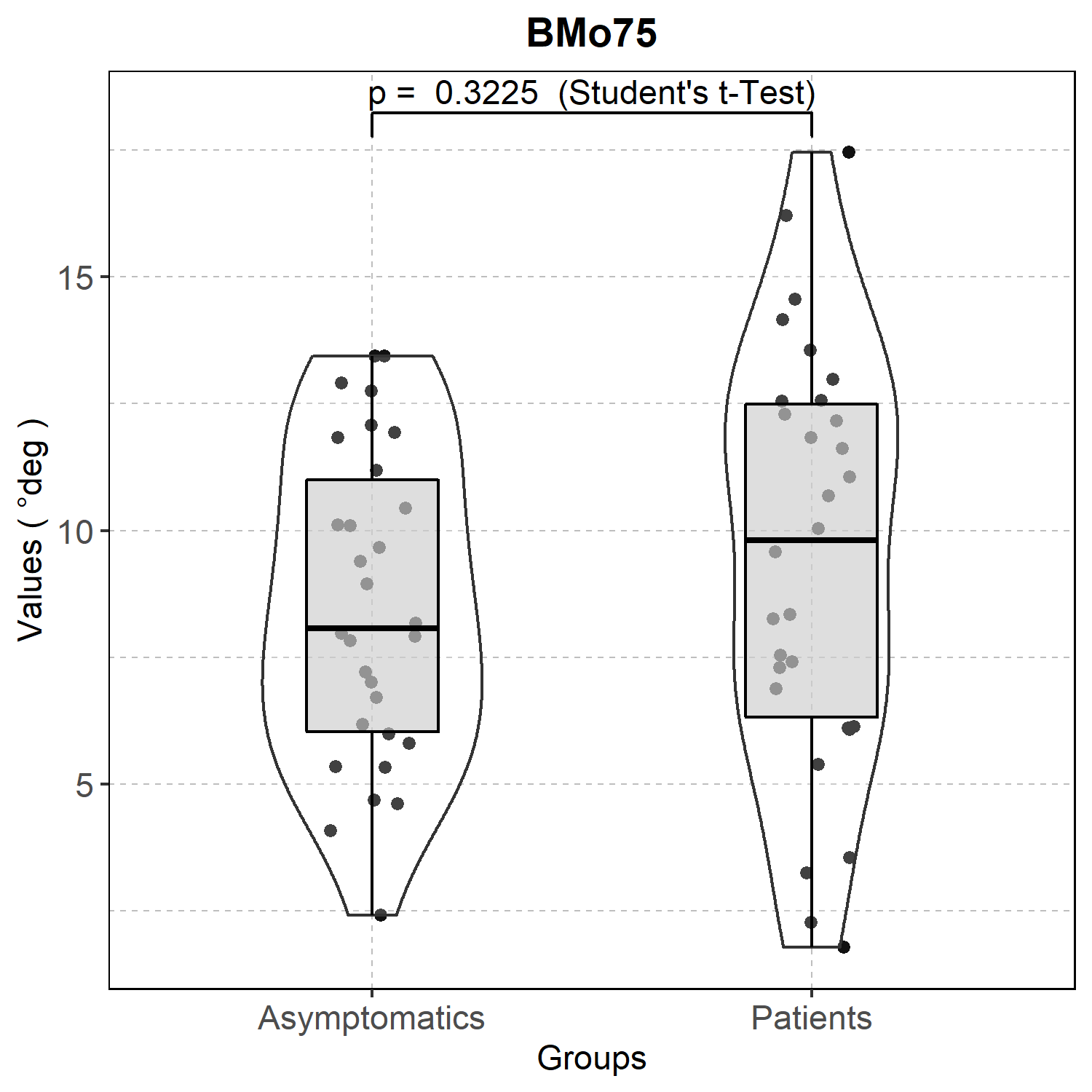

Supplement: Supplementary file 2 — Supplementary Information 2. [file 41598_2023_33504_MOESM2_ESM.zip › BMo075_boxplot.png]

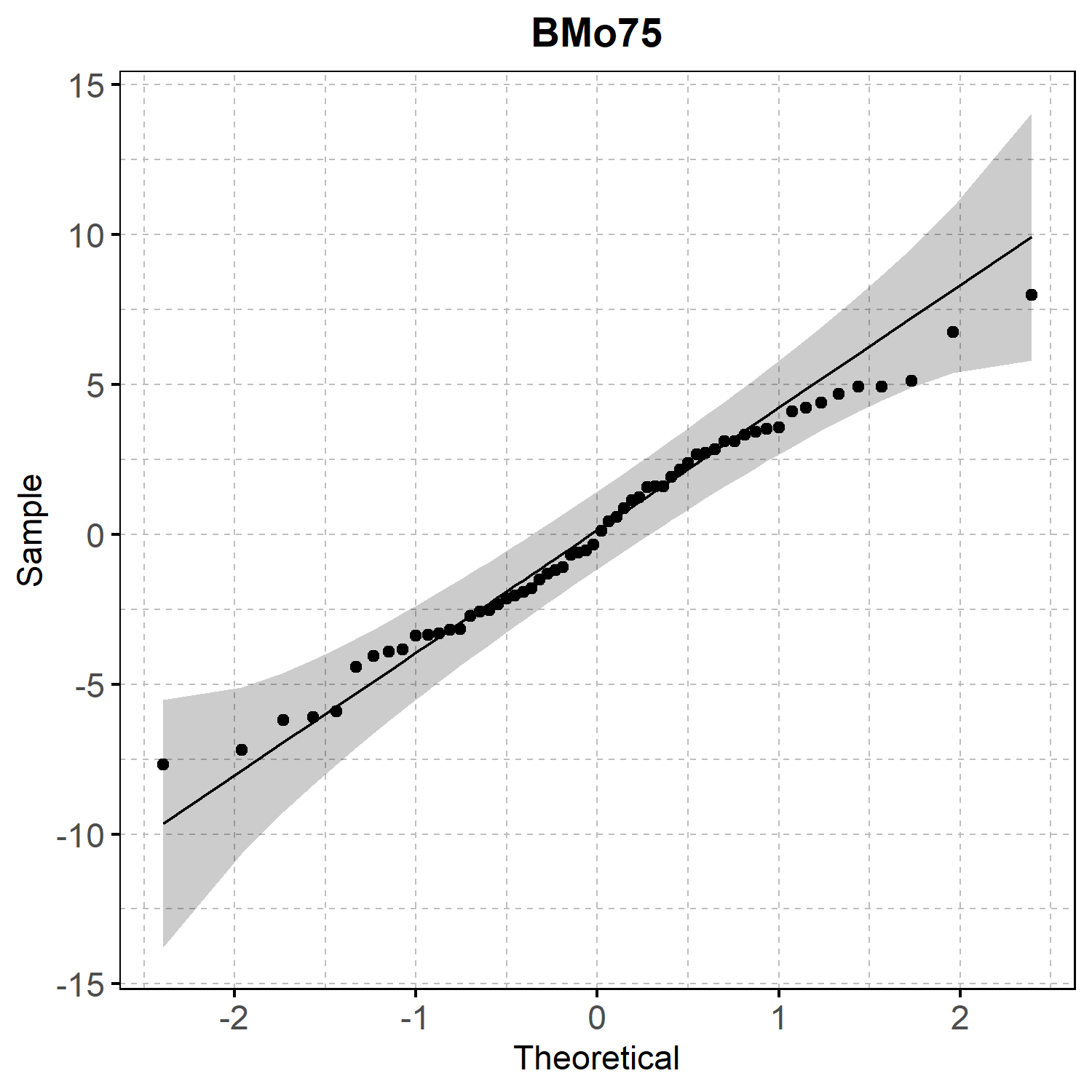

Supplement: Supplementary file 2 — Supplementary Information 2. [file 41598_2023_33504_MOESM2_ESM.zip › BMo075_normality.png]

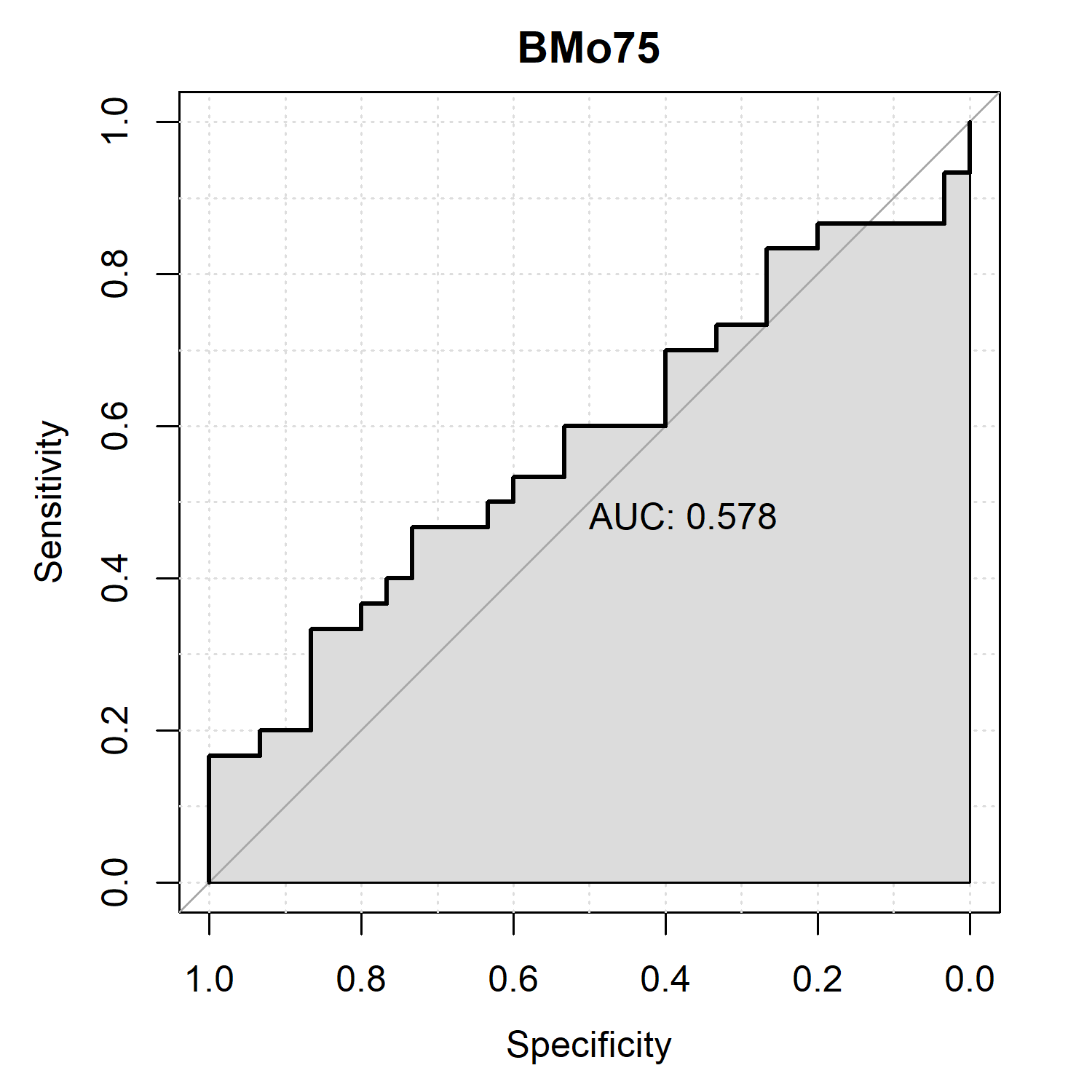

Supplement: Supplementary file 2 — Supplementary Information 2. [file 41598_2023_33504_MOESM2_ESM.zip › BMo075_ROC.png]

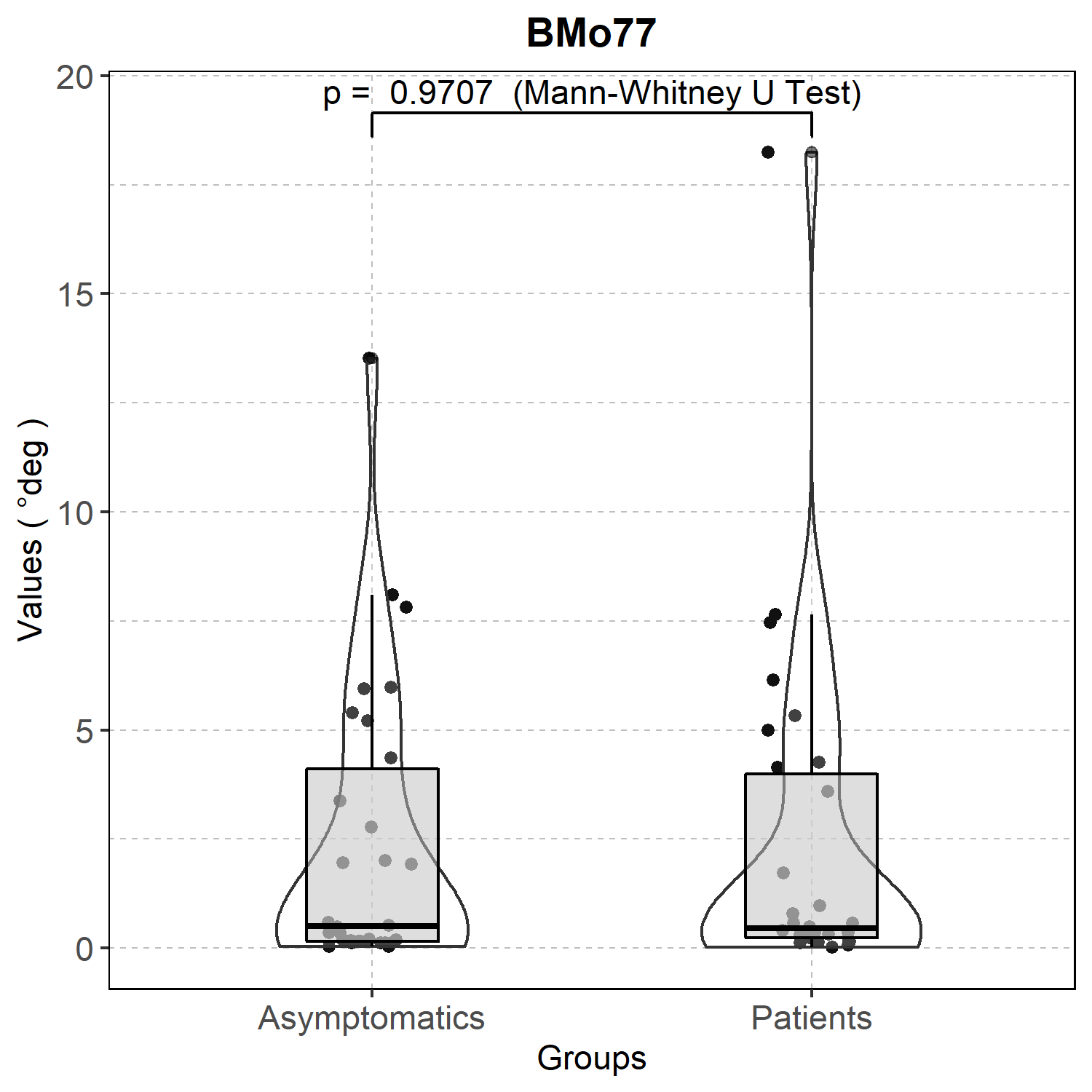

Supplement: Supplementary file 2 — Supplementary Information 2. [file 41598_2023_33504_MOESM2_ESM.zip › BMo077_boxplot.png]

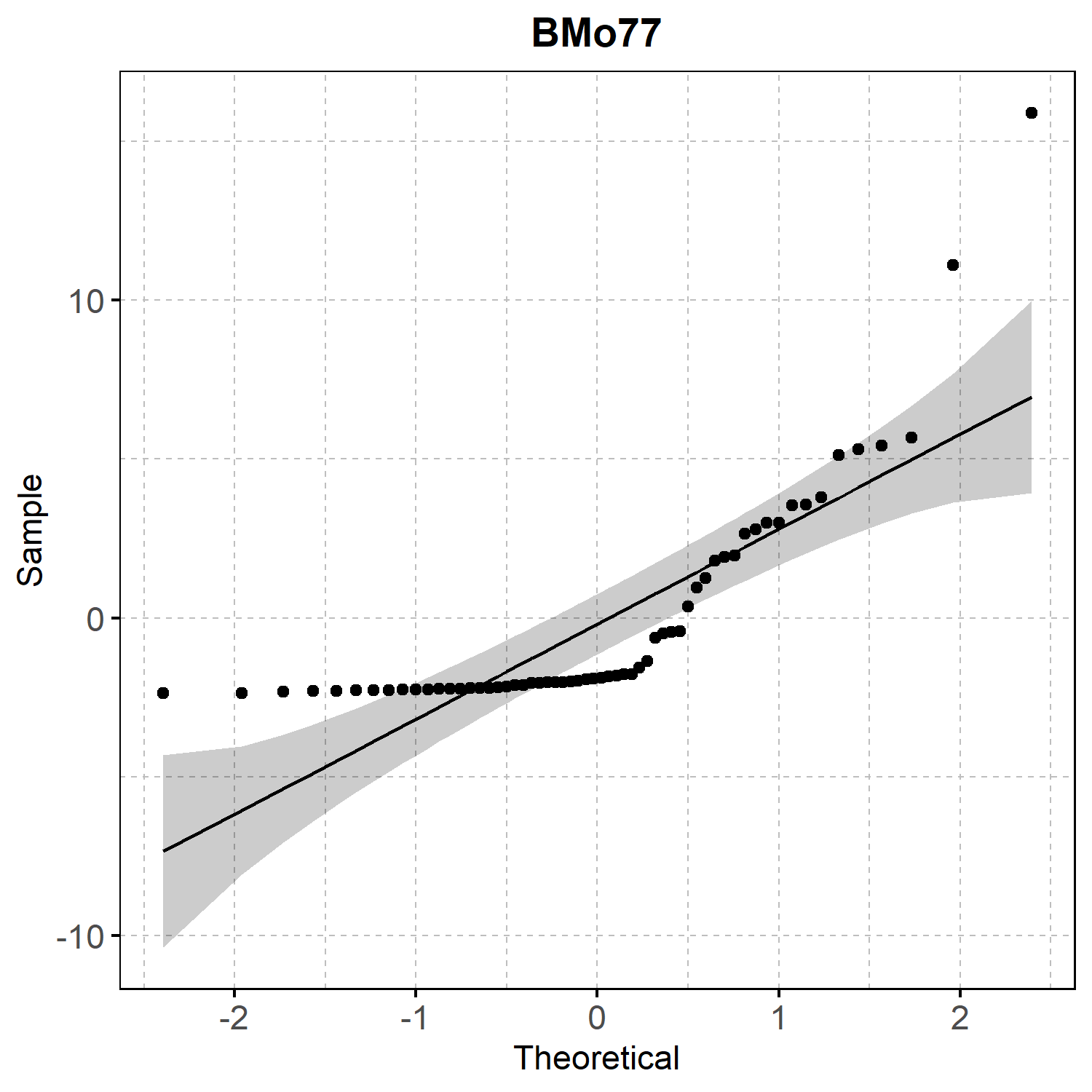

Supplement: Supplementary file 2 — Supplementary Information 2. [file 41598_2023_33504_MOESM2_ESM.zip › BMo077_normality.png]

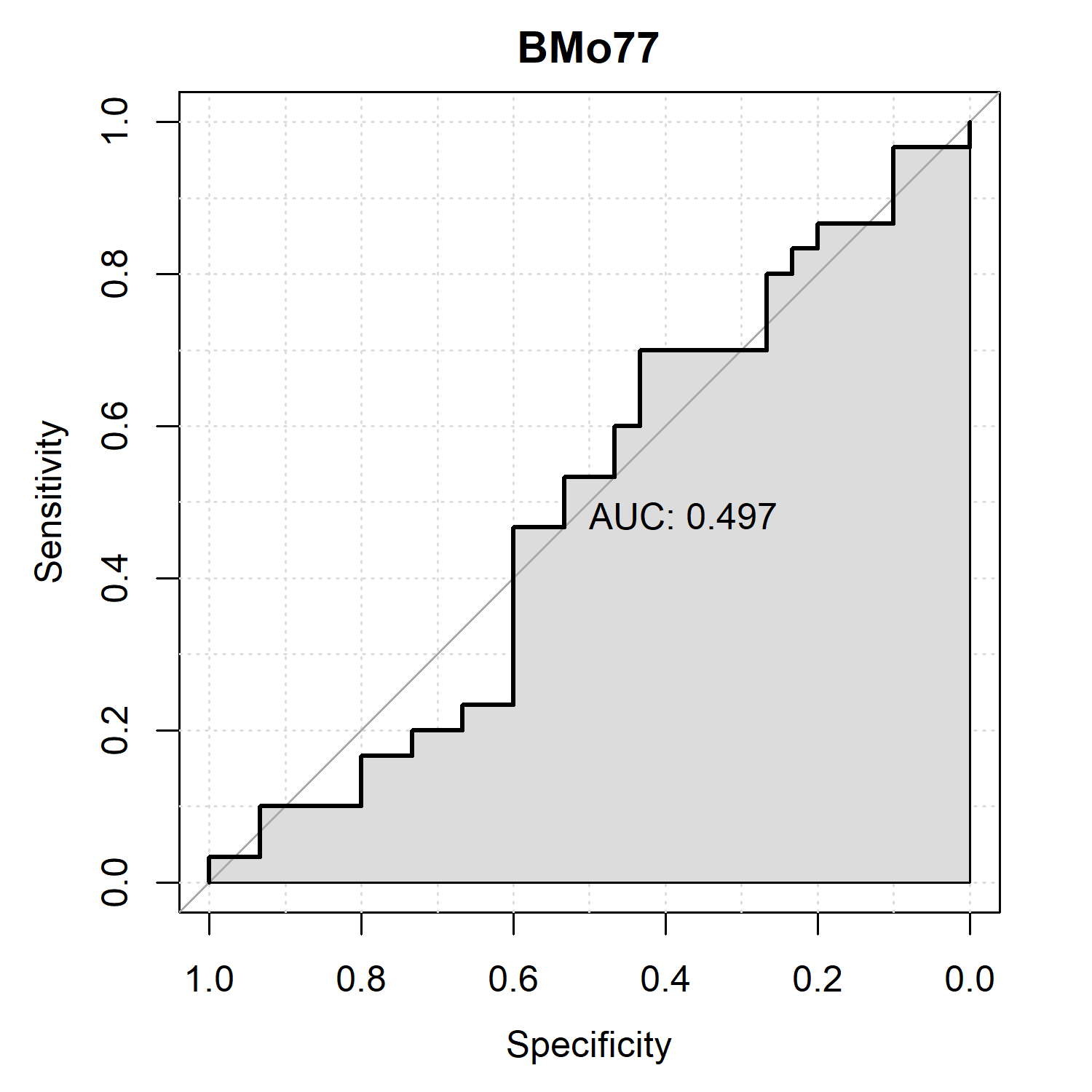

Supplement: Supplementary file 2 — Supplementary Information 2. [file 41598_2023_33504_MOESM2_ESM.zip › BMo077_ROC.png]

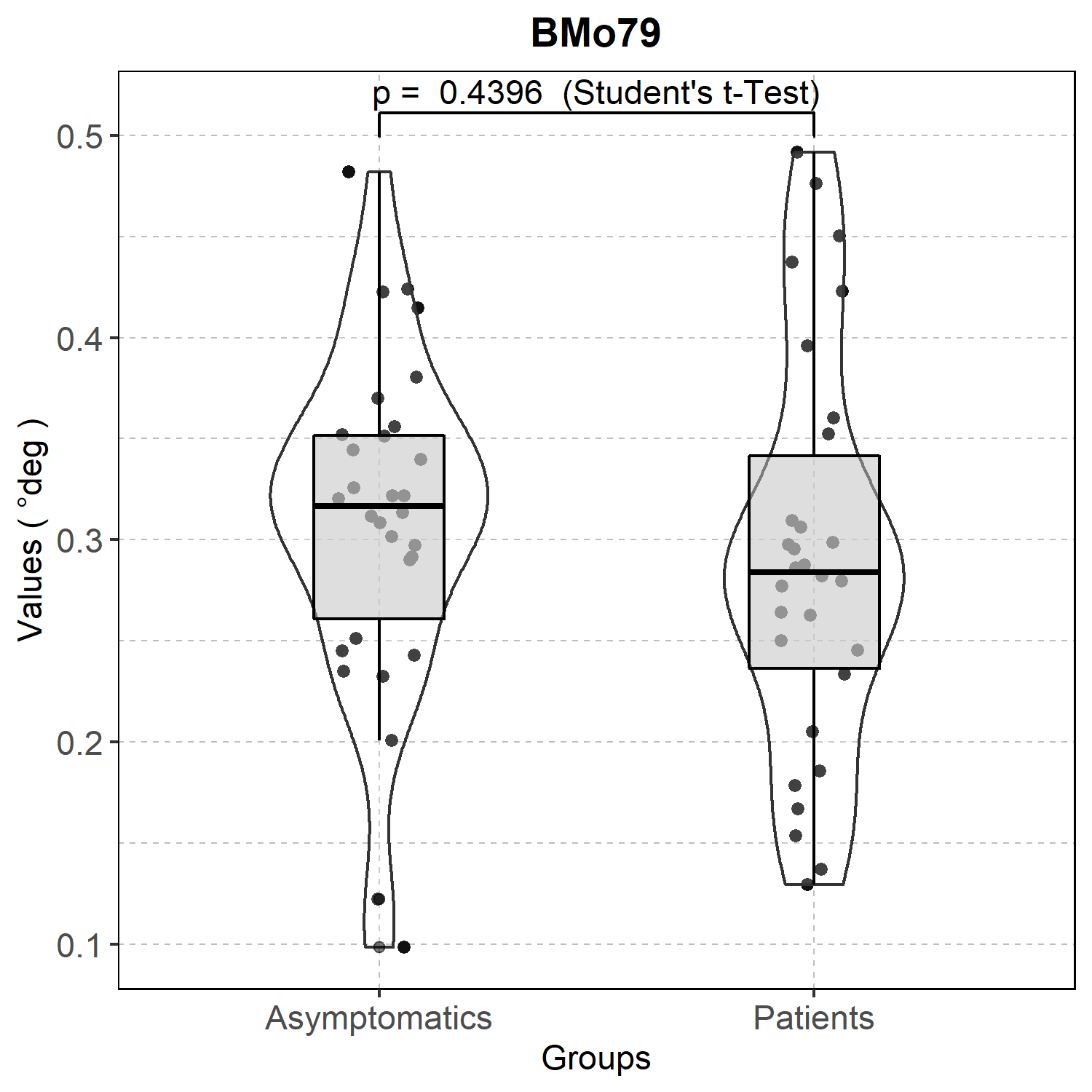

Supplement: Supplementary file 2 — Supplementary Information 2. [file 41598_2023_33504_MOESM2_ESM.zip › BMo079_boxplot.png]

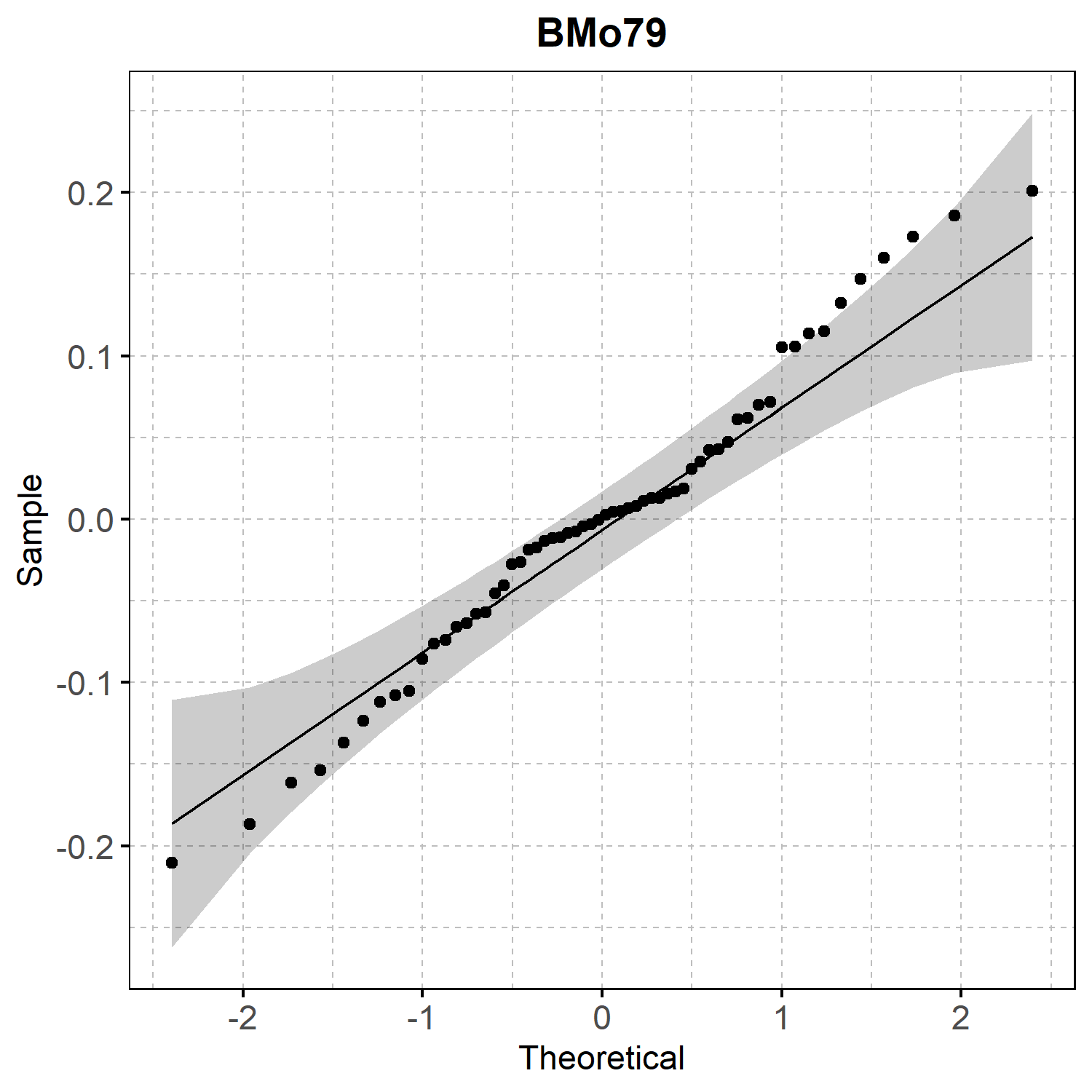

Supplement: Supplementary file 2 — Supplementary Information 2. [file 41598_2023_33504_MOESM2_ESM.zip › BMo079_normality.png]

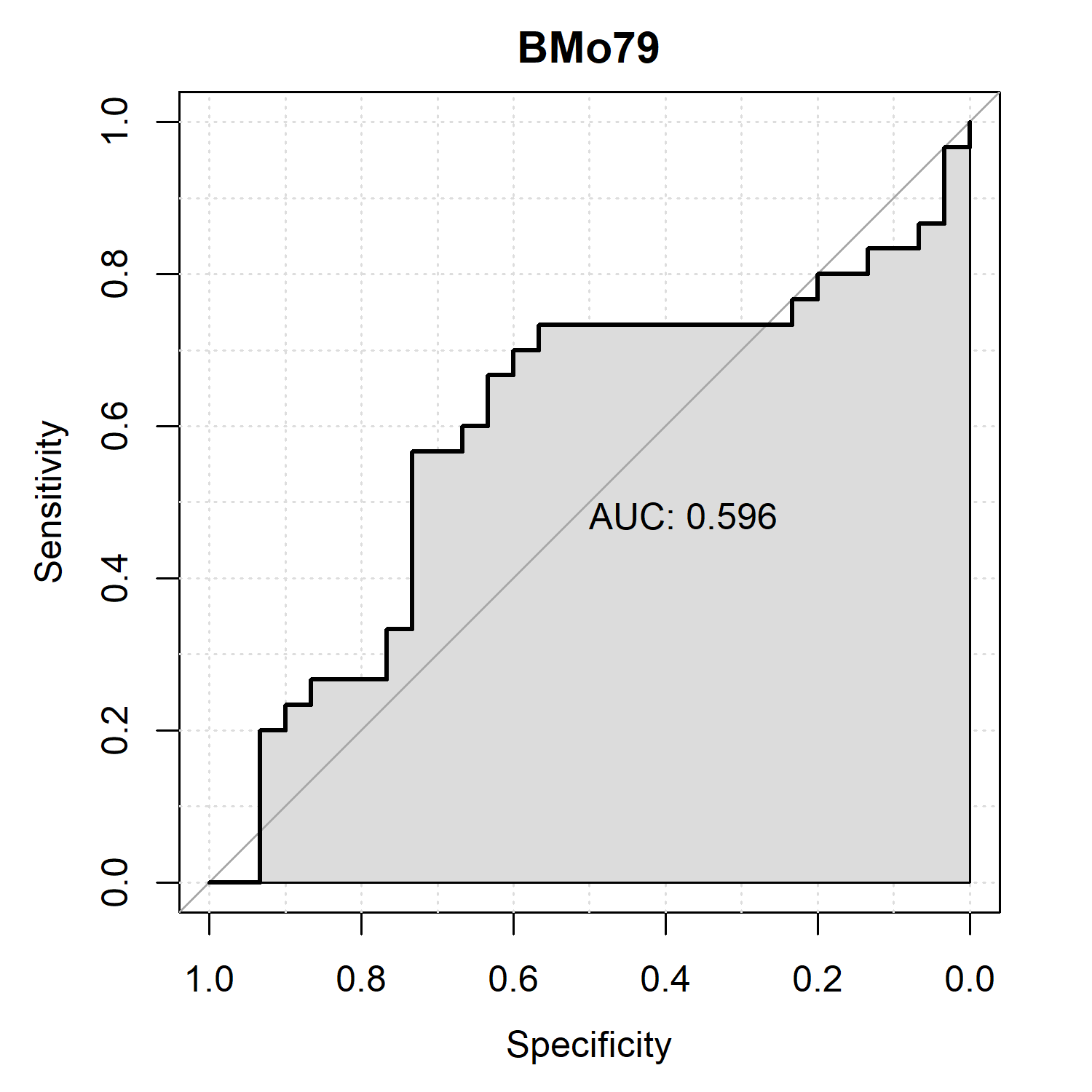

Supplement: Supplementary file 2 — Supplementary Information 2. [file 41598_2023_33504_MOESM2_ESM.zip › BMo079_ROC.png]

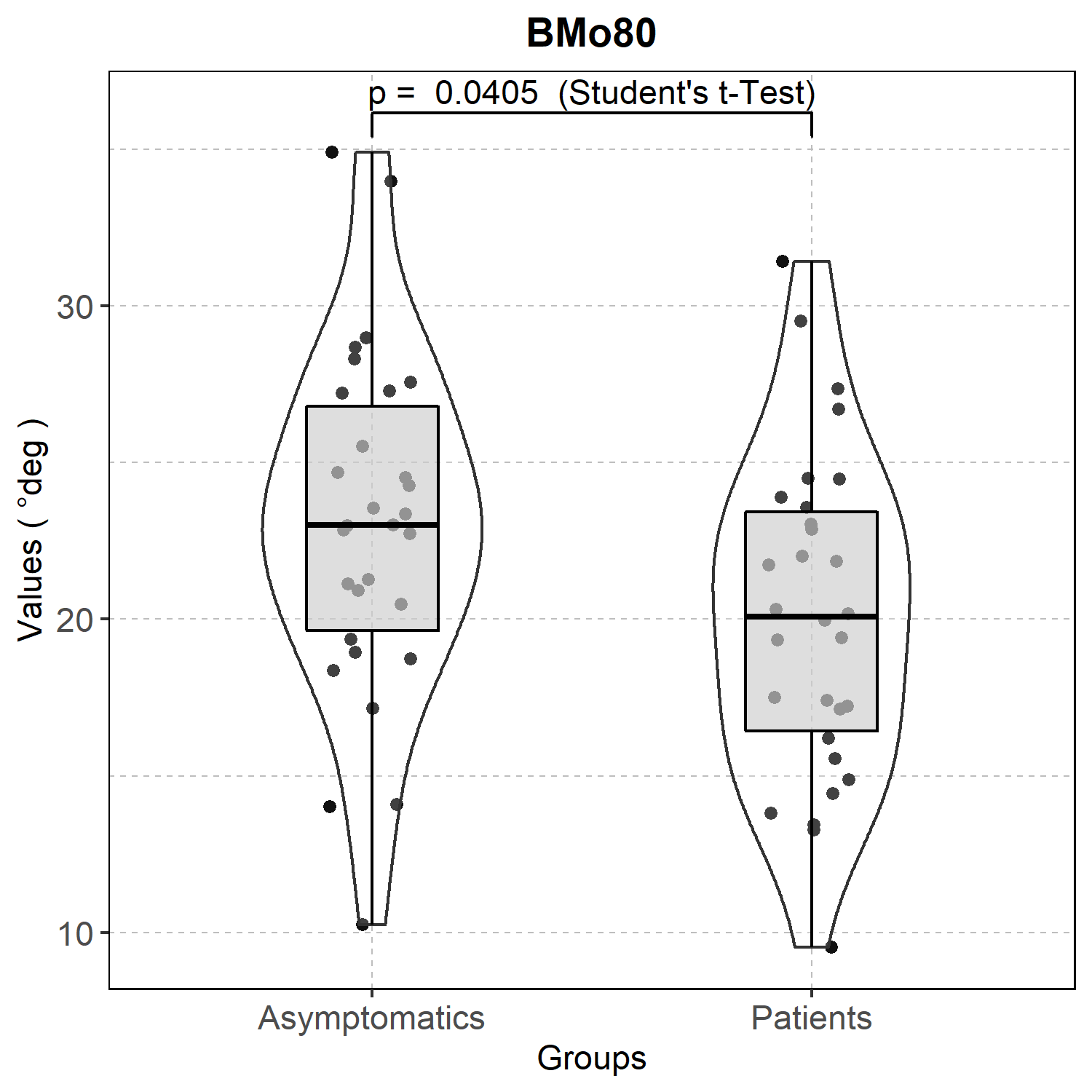

Supplement: Supplementary file 2 — Supplementary Information 2. [file 41598_2023_33504_MOESM2_ESM.zip › BMo080_boxplot.png]

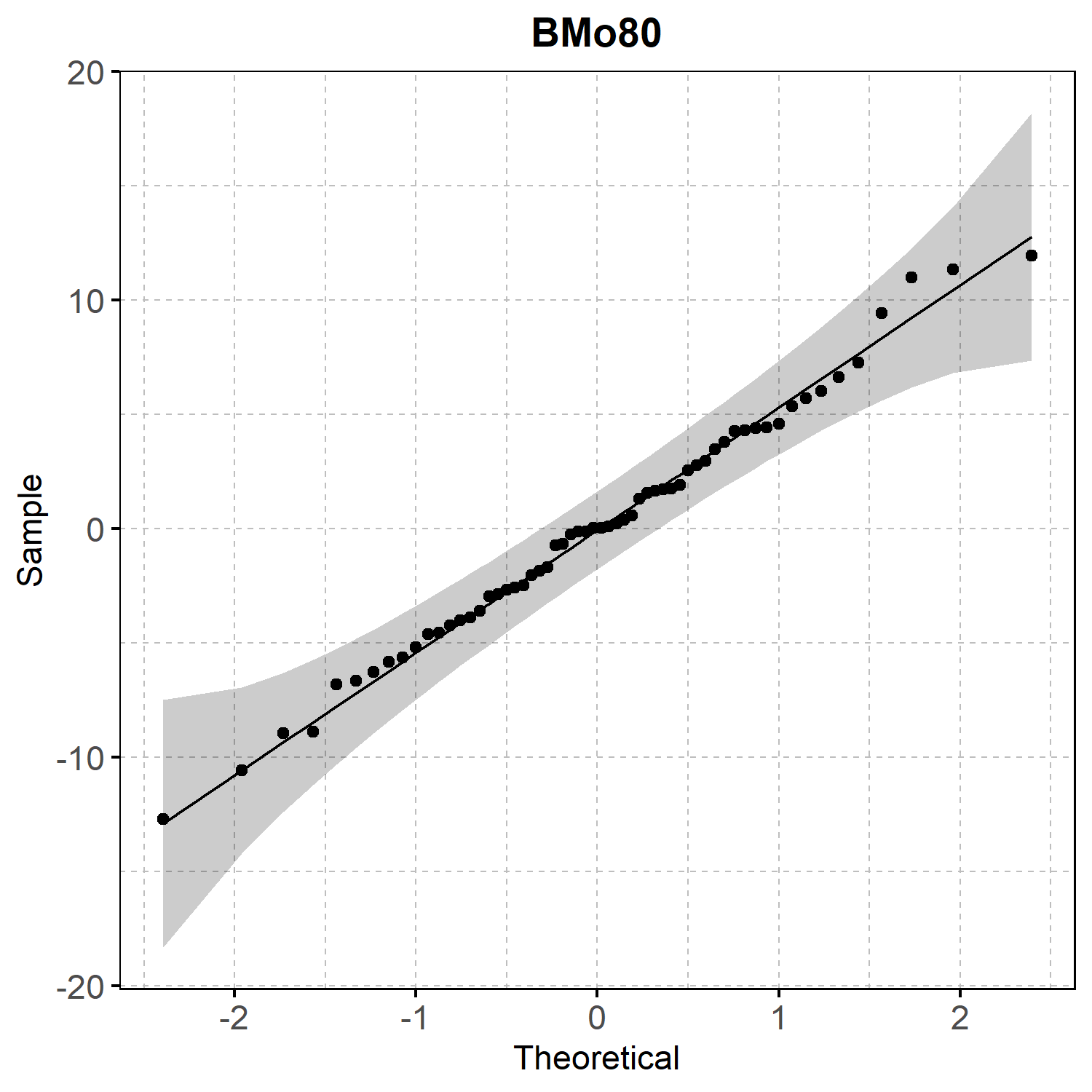

Supplement: Supplementary file 2 — Supplementary Information 2. [file 41598_2023_33504_MOESM2_ESM.zip › BMo080_normality.png]

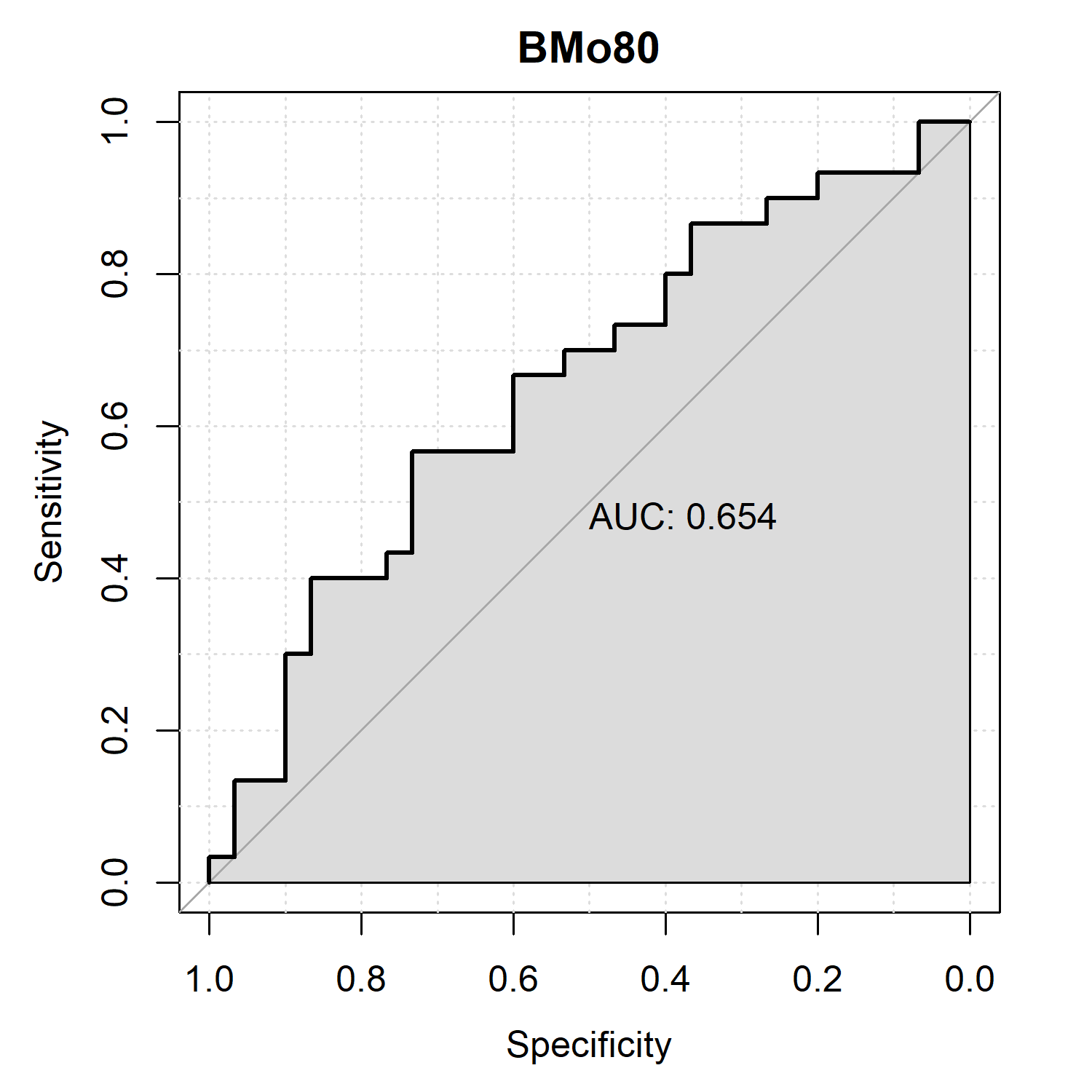

Supplement: Supplementary file 2 — Supplementary Information 2. [file 41598_2023_33504_MOESM2_ESM.zip › BMo080_ROC.png]

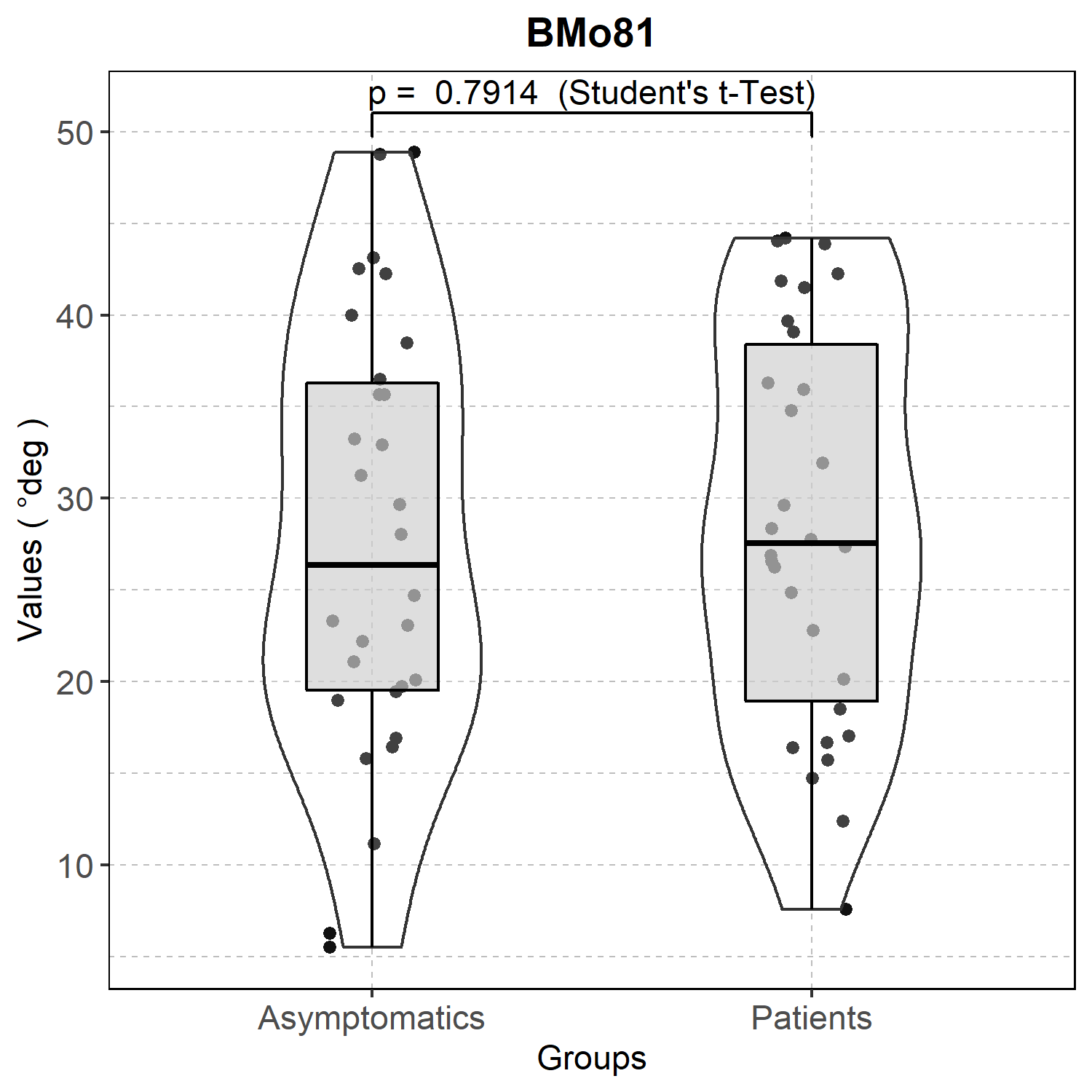

Supplement: Supplementary file 2 — Supplementary Information 2. [file 41598_2023_33504_MOESM2_ESM.zip › BMo081_boxplot.png]

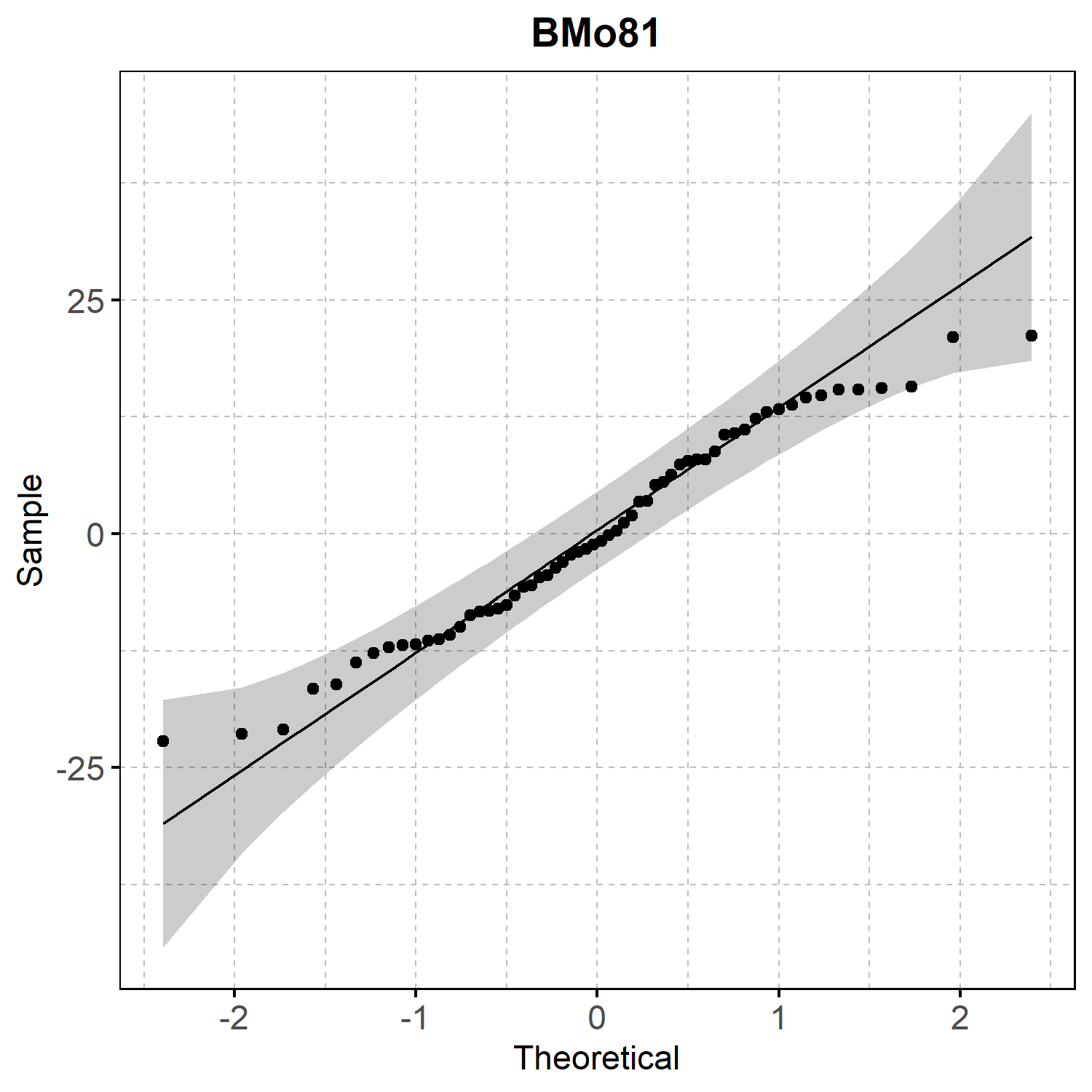

Supplement: Supplementary file 2 — Supplementary Information 2. [file 41598_2023_33504_MOESM2_ESM.zip › BMo081_normality.png]

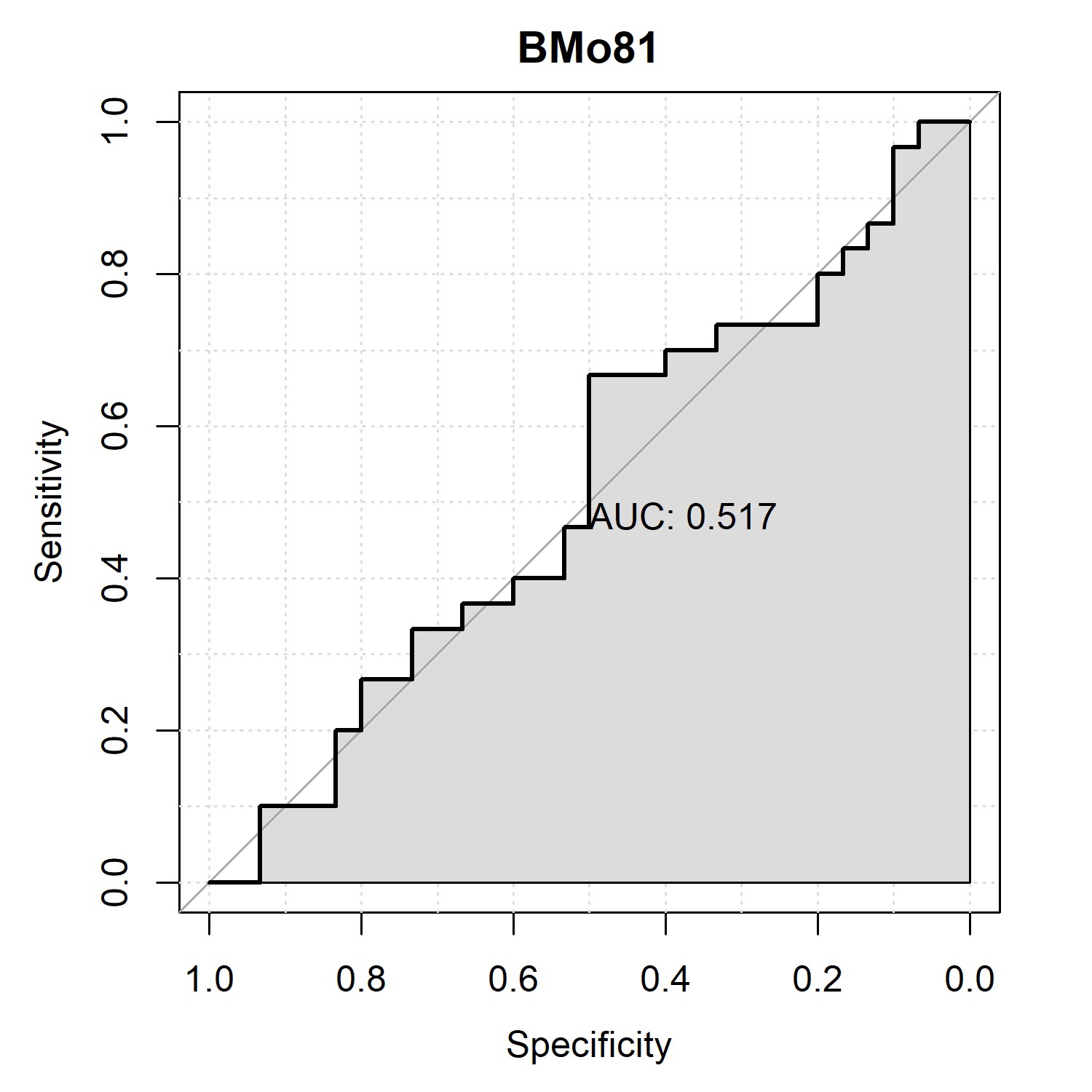

Supplement: Supplementary file 2 — Supplementary Information 2. [file 41598_2023_33504_MOESM2_ESM.zip › BMo081_ROC.png]

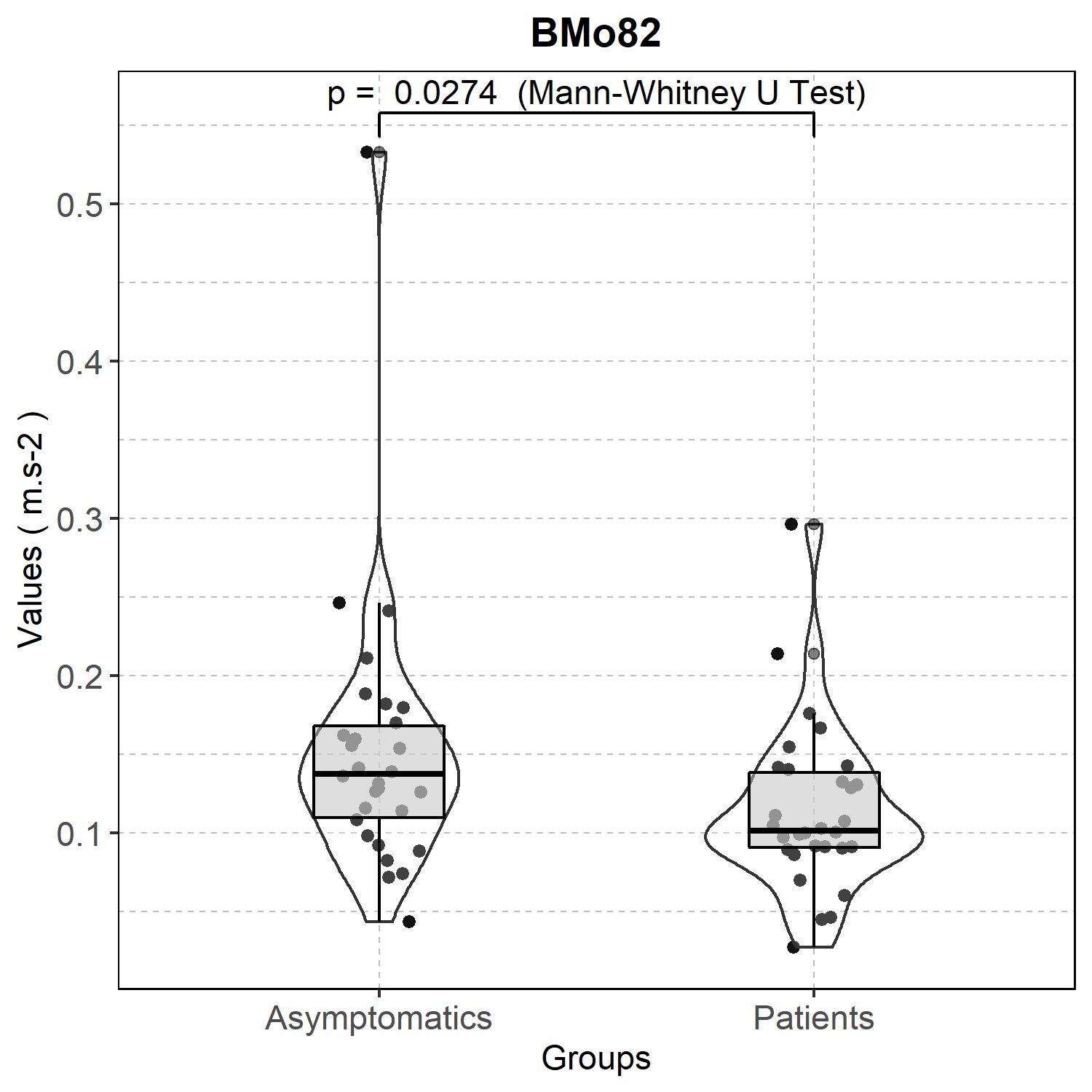

Supplement: Supplementary file 2 — Supplementary Information 2. [file 41598_2023_33504_MOESM2_ESM.zip › BMo082_boxplot.png]

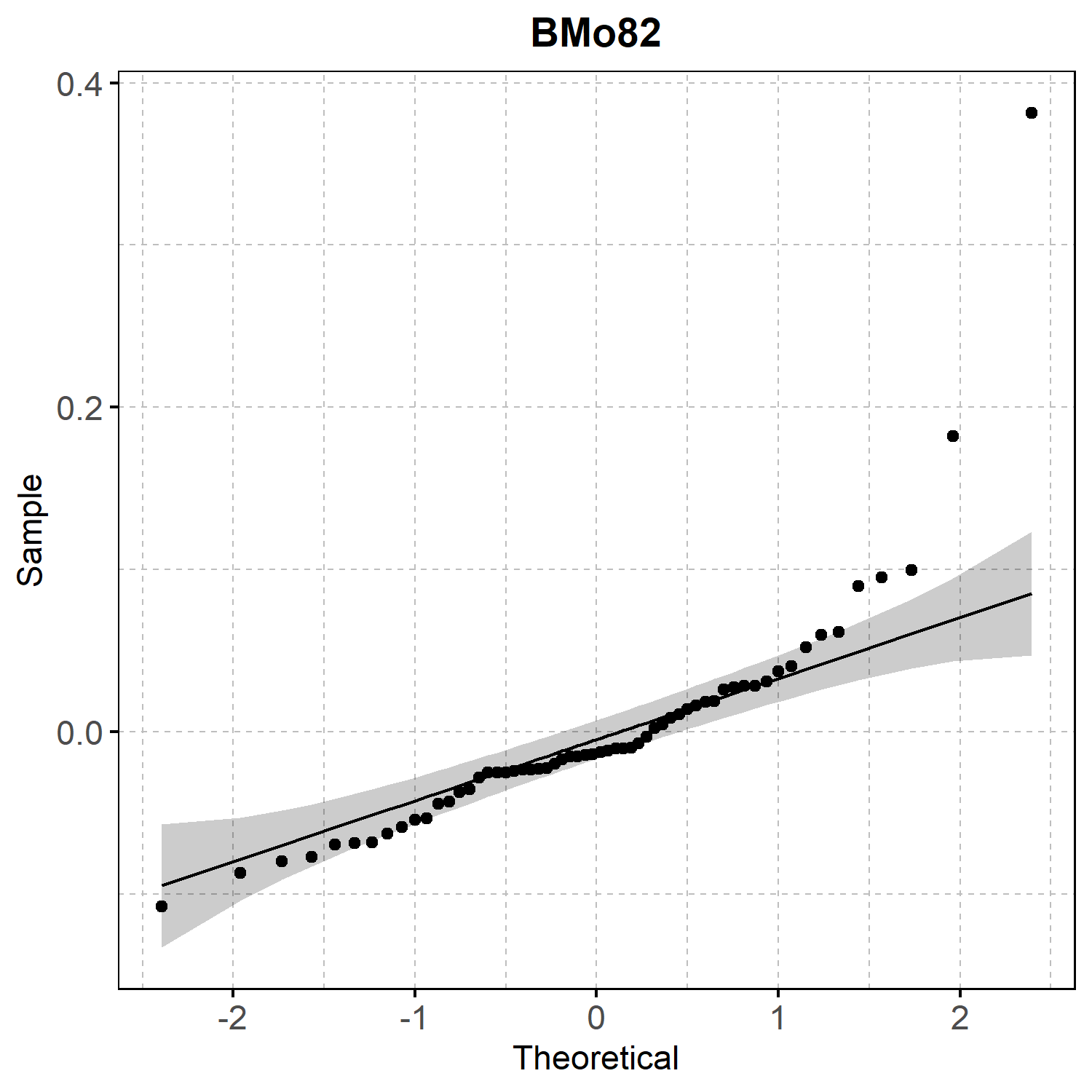

Supplement: Supplementary file 2 — Supplementary Information 2. [file 41598_2023_33504_MOESM2_ESM.zip › BMo082_normality.png]

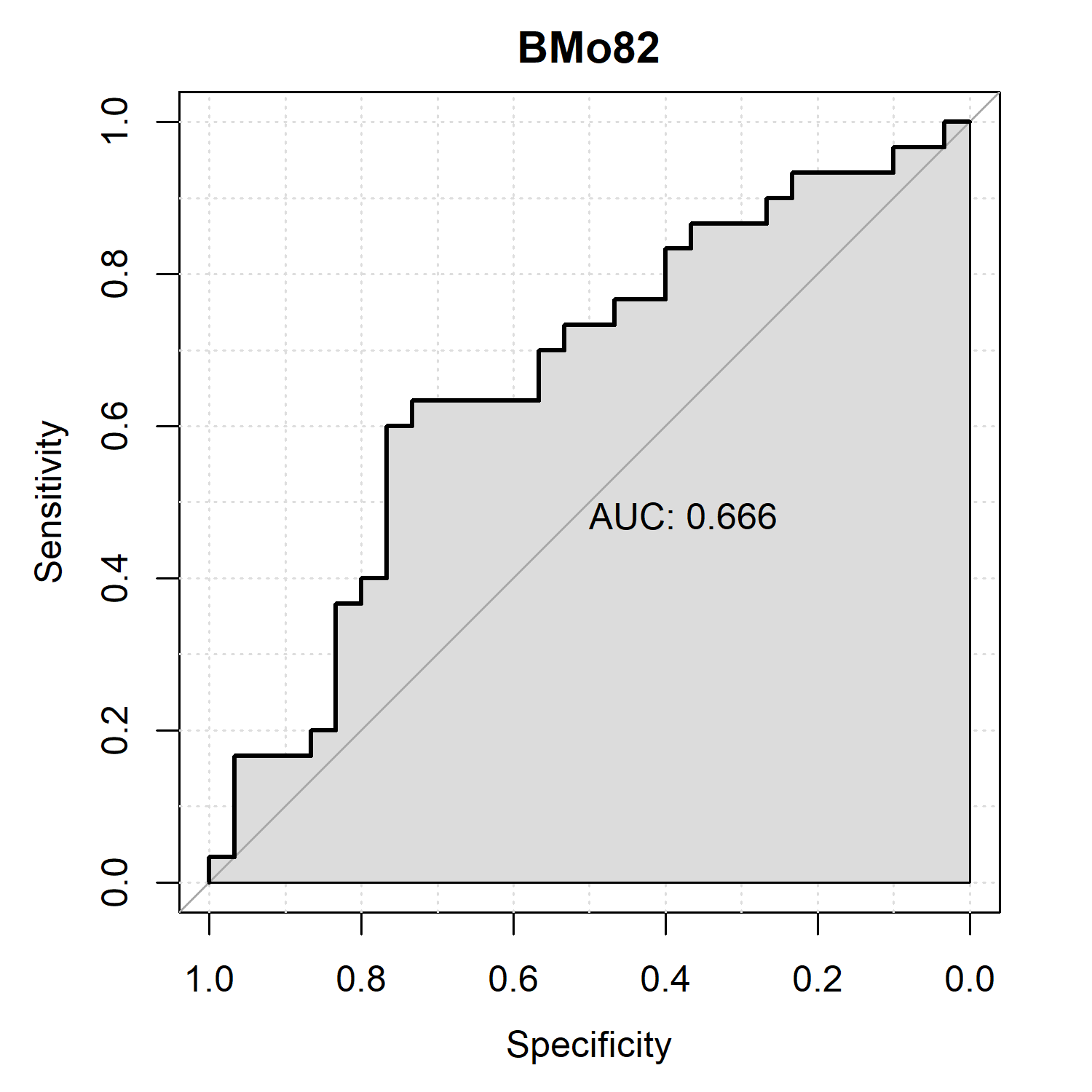

Supplement: Supplementary file 2 — Supplementary Information 2. [file 41598_2023_33504_MOESM2_ESM.zip › BMo082_ROC.png]

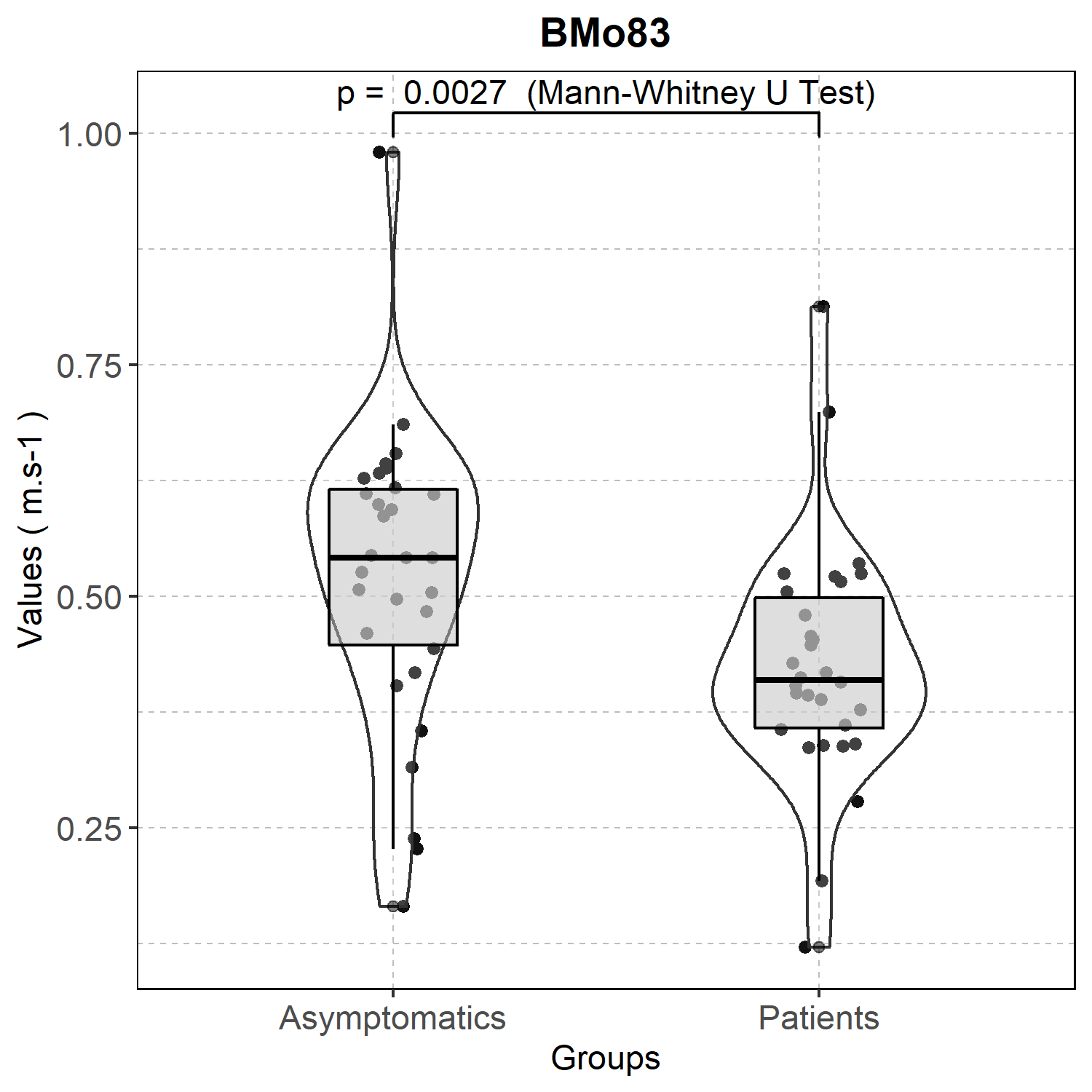

Supplement: Supplementary file 2 — Supplementary Information 2. [file 41598_2023_33504_MOESM2_ESM.zip › BMo083_boxplot.png]

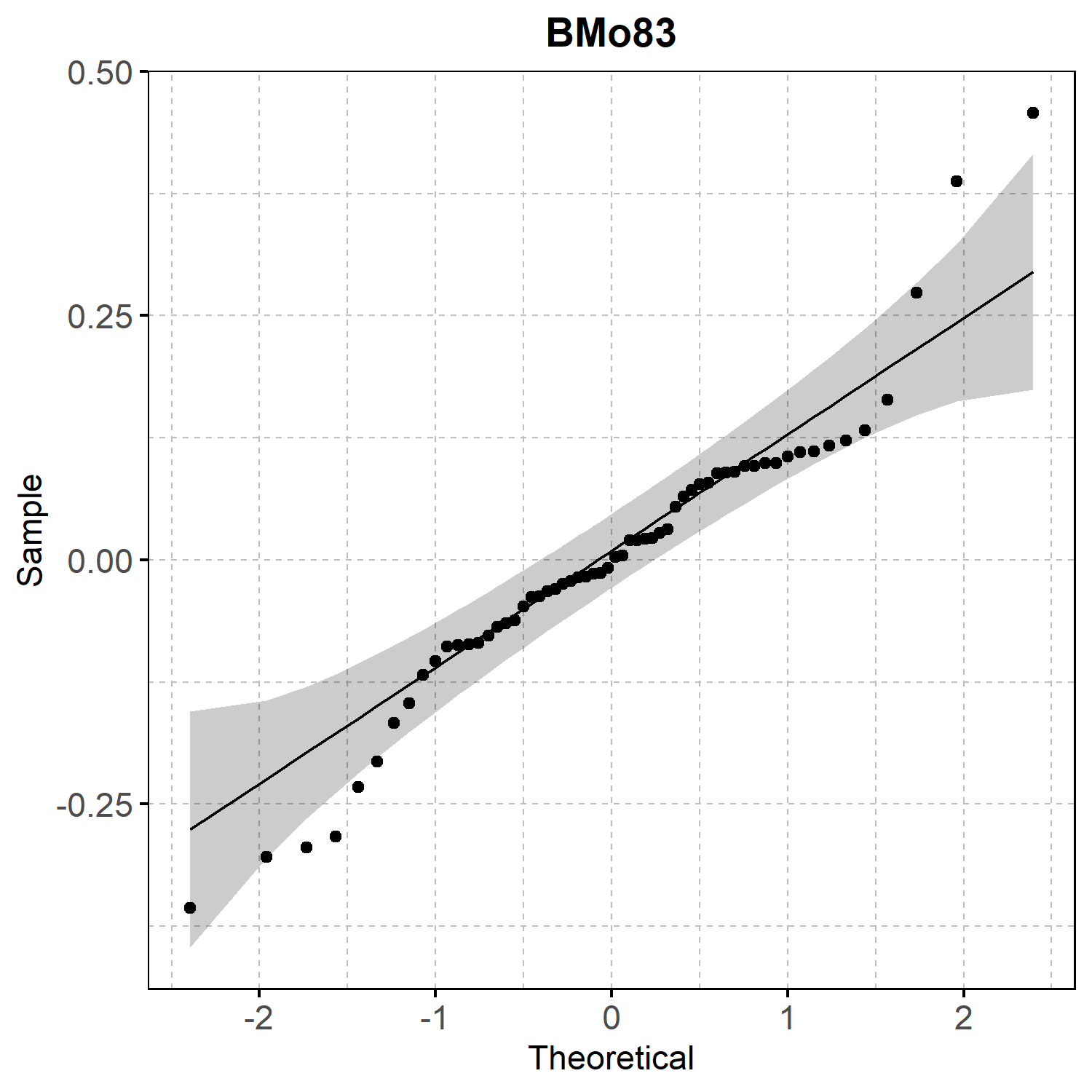

Supplement: Supplementary file 2 — Supplementary Information 2. [file 41598_2023_33504_MOESM2_ESM.zip › BMo083_normality.png]

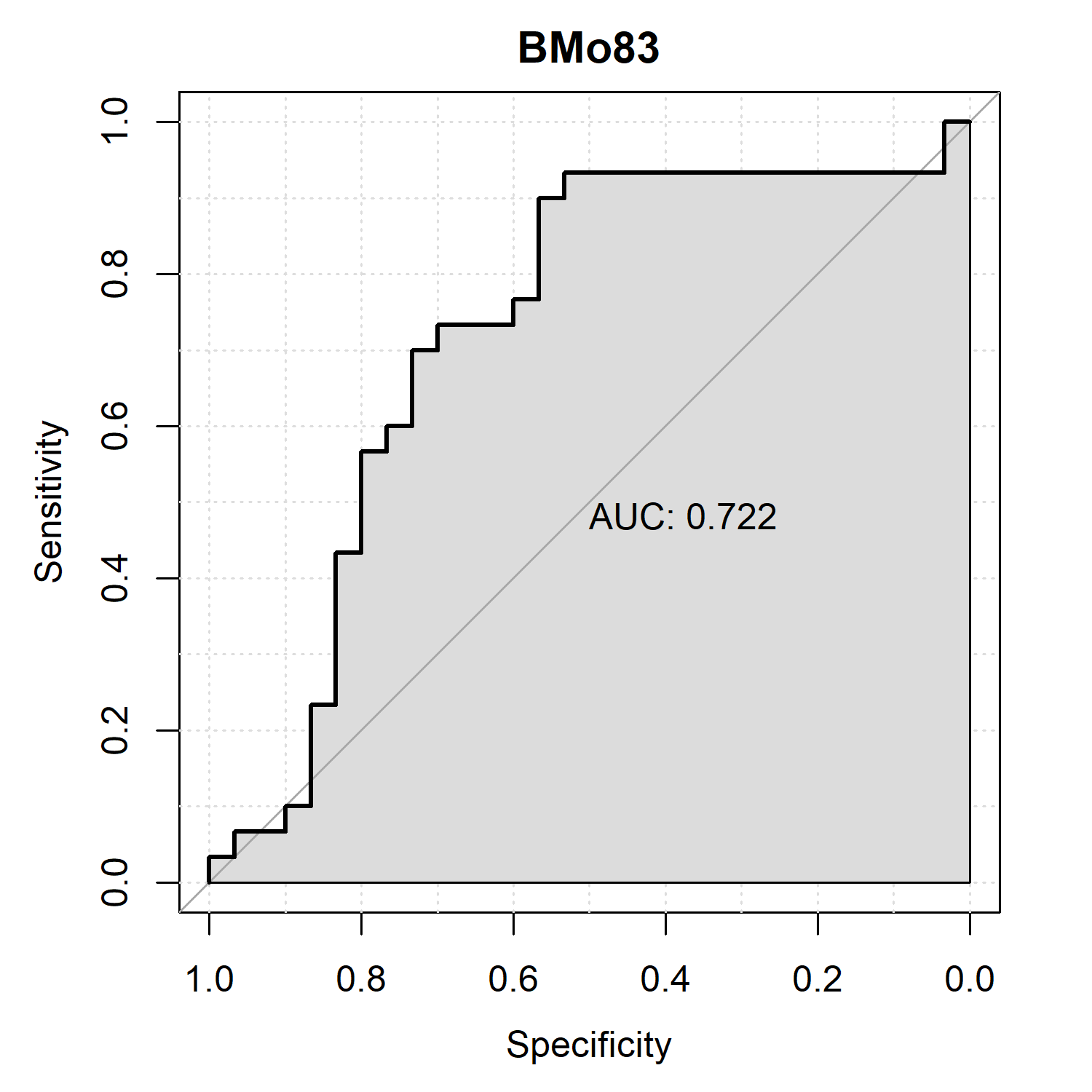

Supplement: Supplementary file 2 — Supplementary Information 2. [file 41598_2023_33504_MOESM2_ESM.zip › BMo083_ROC.png]

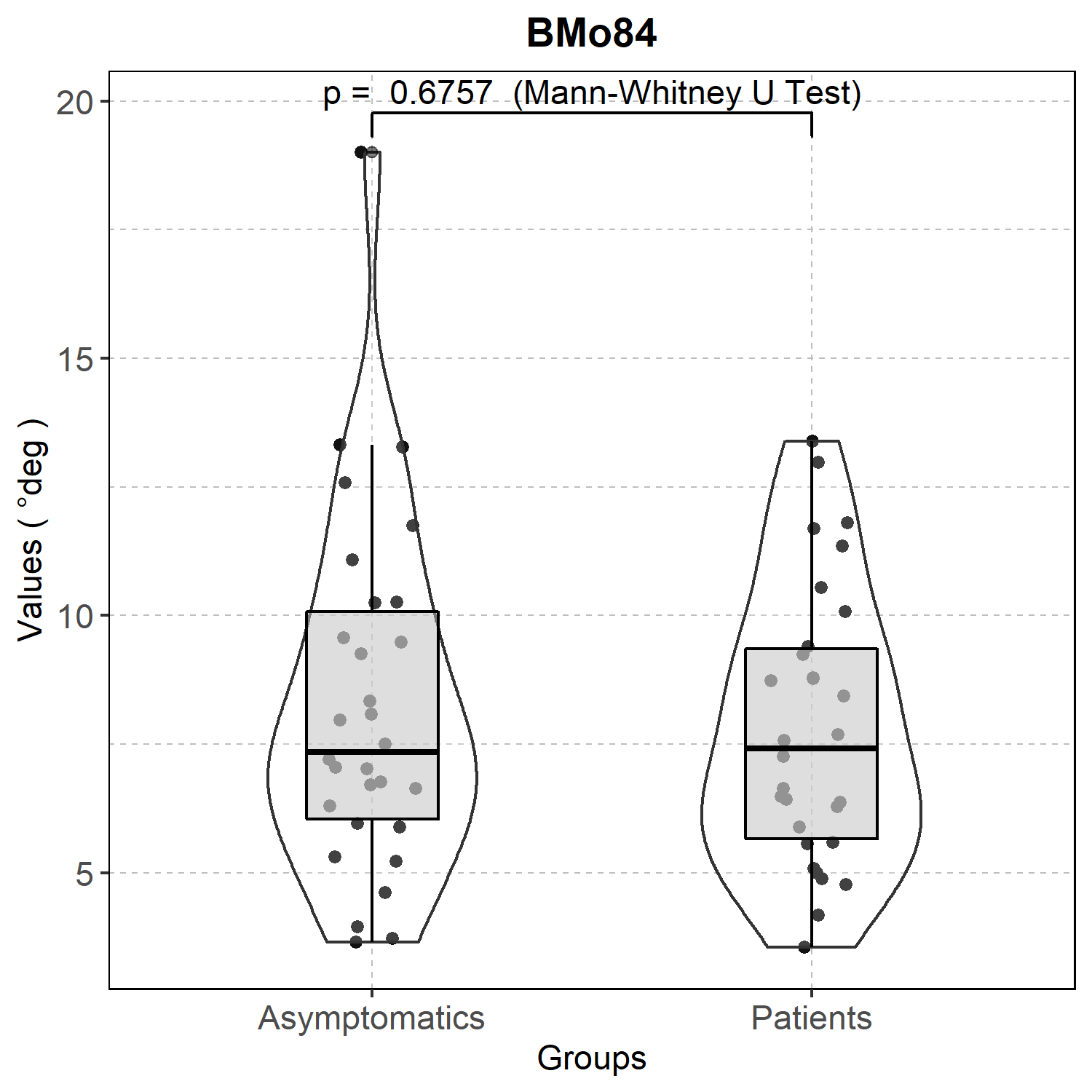

Supplement: Supplementary file 2 — Supplementary Information 2. [file 41598_2023_33504_MOESM2_ESM.zip › BMo084_boxplot.png]

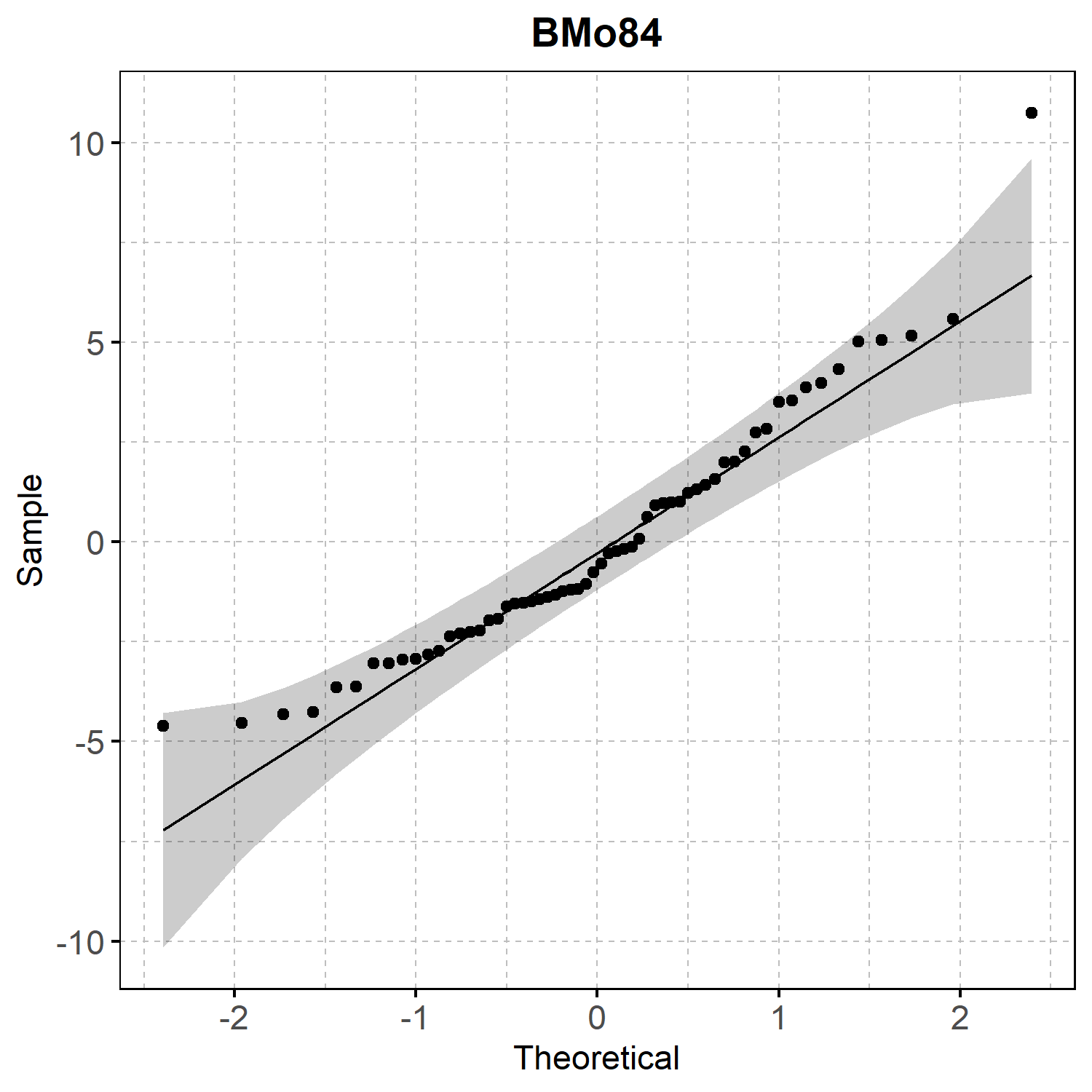

Supplement: Supplementary file 2 — Supplementary Information 2. [file 41598_2023_33504_MOESM2_ESM.zip › BMo084_normality.png]

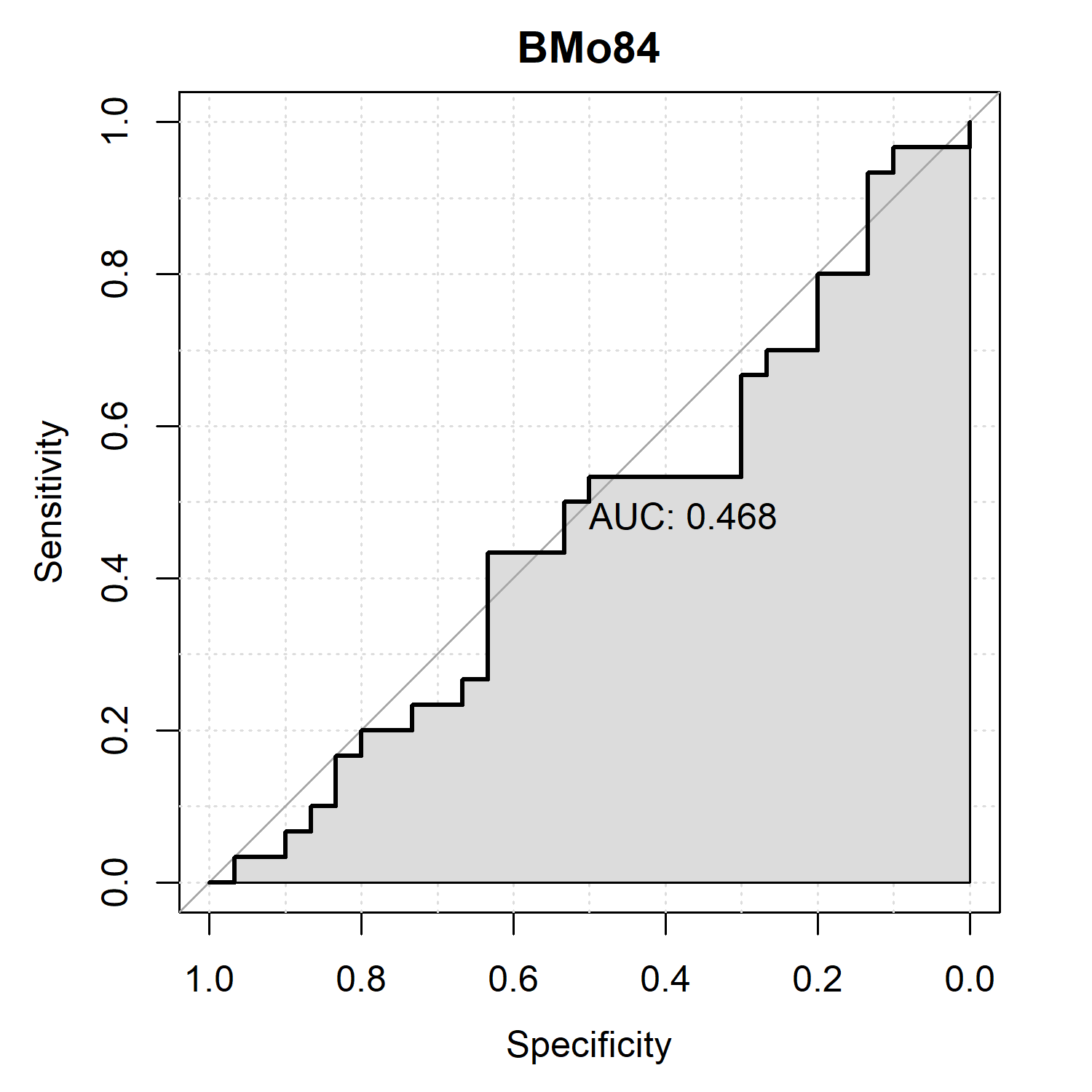

Supplement: Supplementary file 2 — Supplementary Information 2. [file 41598_2023_33504_MOESM2_ESM.zip › BMo084_ROC.png]

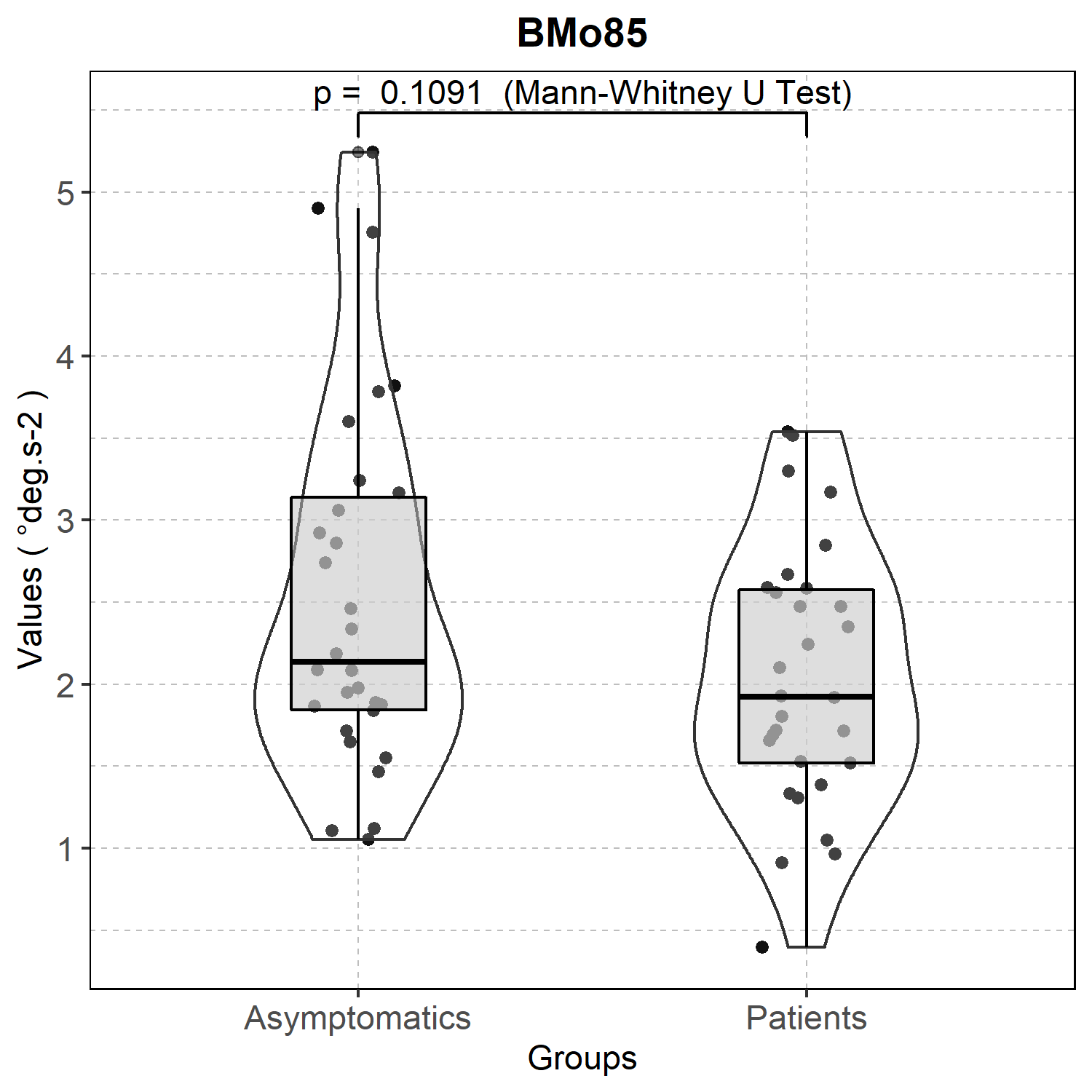

Supplement: Supplementary file 2 — Supplementary Information 2. [file 41598_2023_33504_MOESM2_ESM.zip › BMo085_boxplot.png]

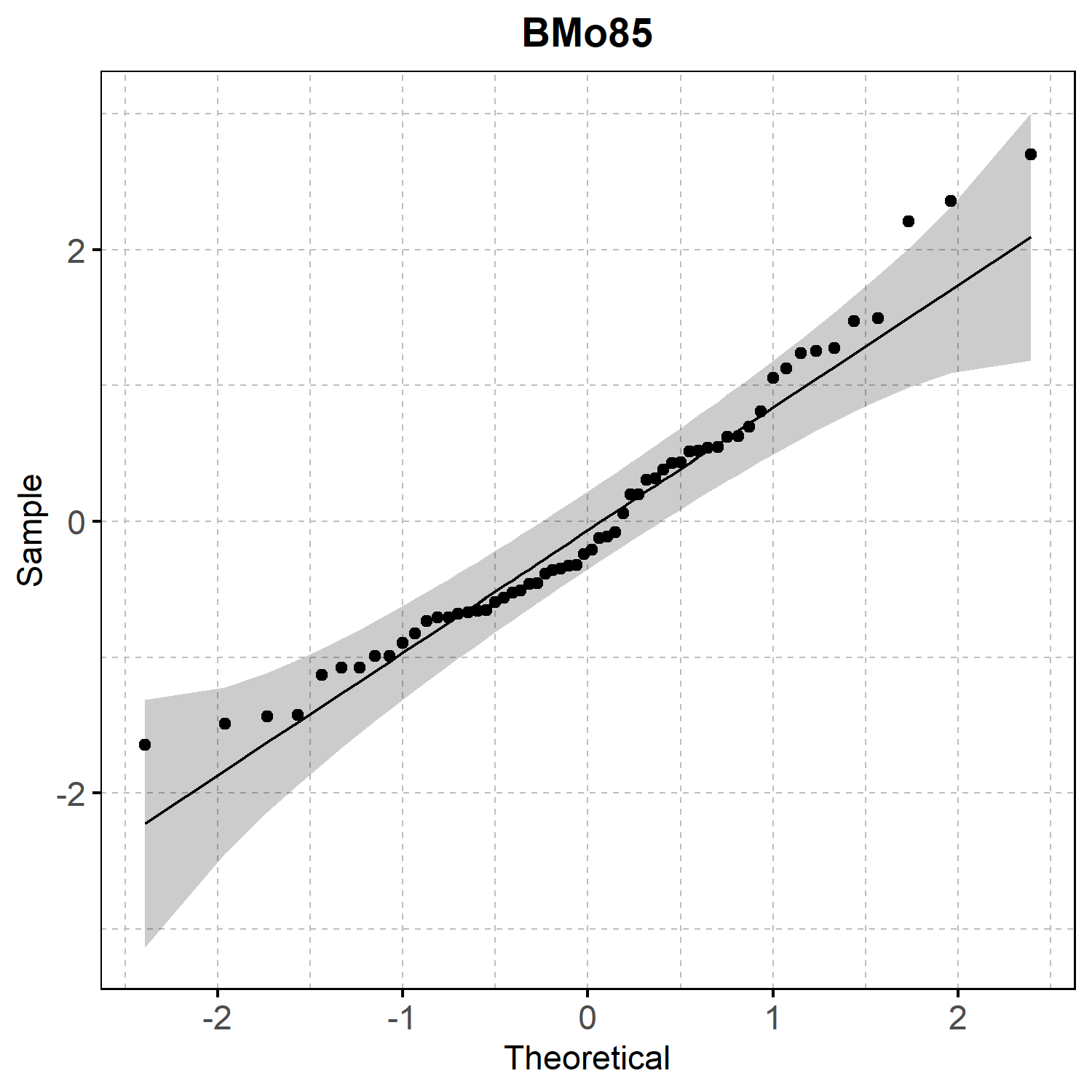

Supplement: Supplementary file 2 — Supplementary Information 2. [file 41598_2023_33504_MOESM2_ESM.zip › BMo085_normality.png]

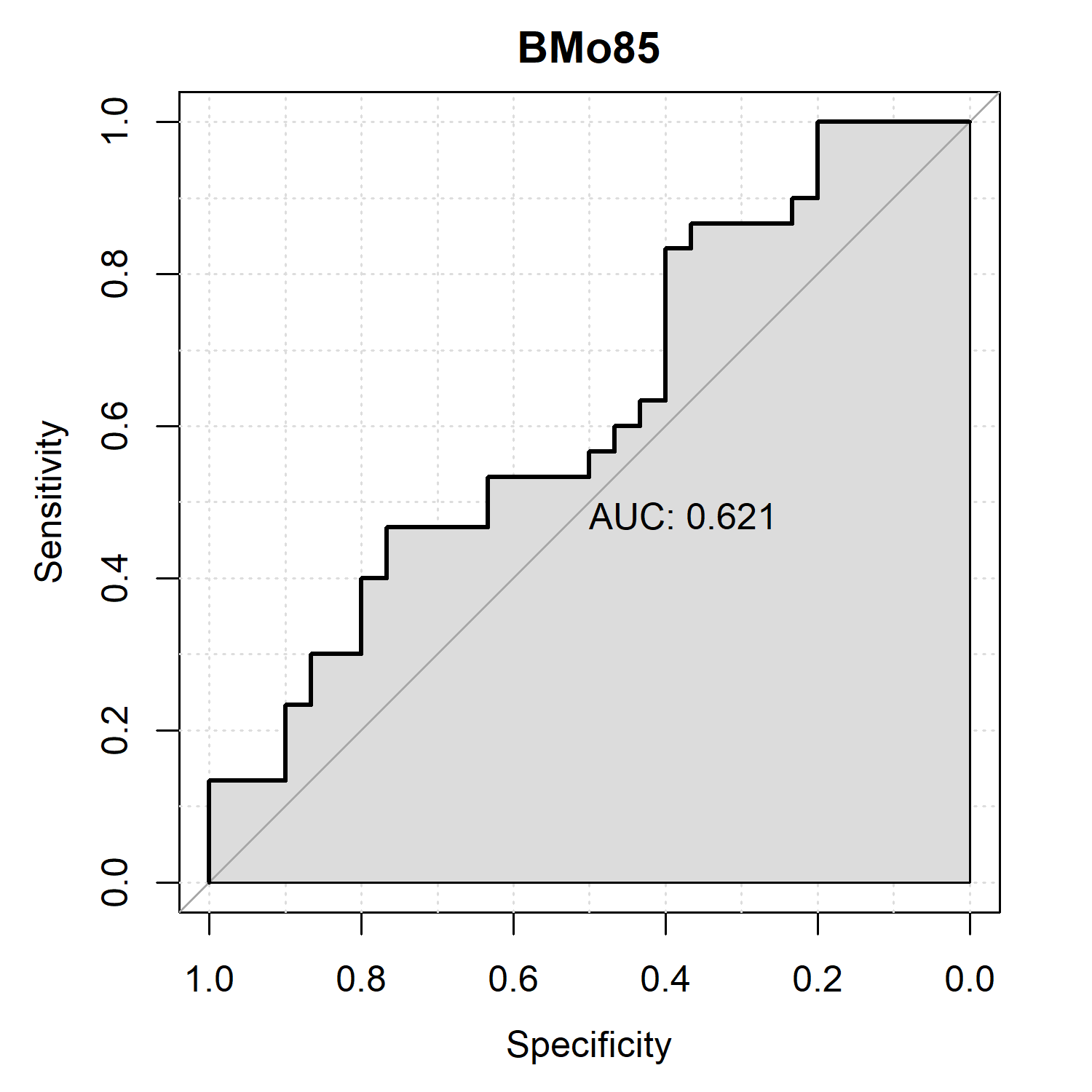

Supplement: Supplementary file 2 — Supplementary Information 2. [file 41598_2023_33504_MOESM2_ESM.zip › BMo085_ROC.png]

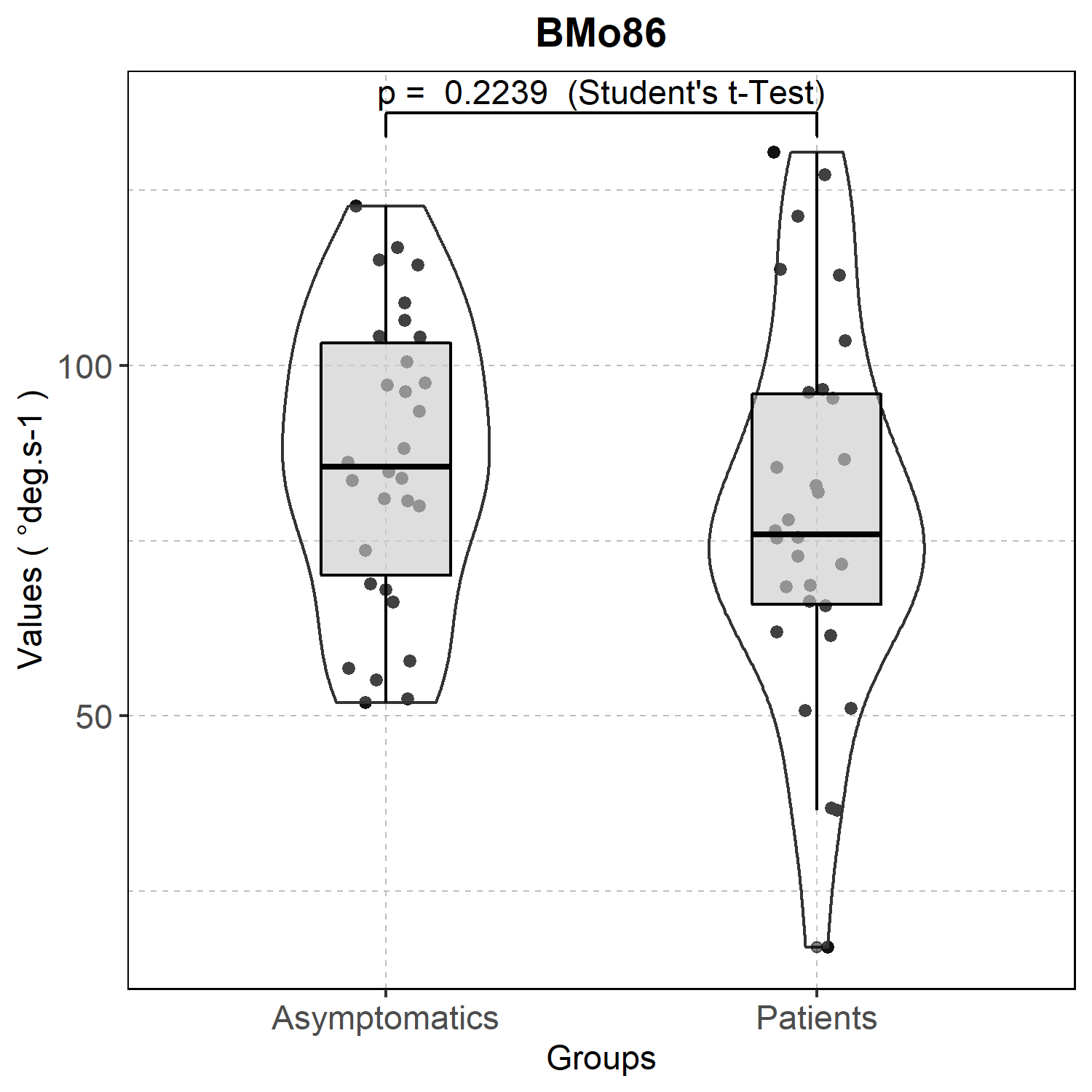

Supplement: Supplementary file 2 — Supplementary Information 2. [file 41598_2023_33504_MOESM2_ESM.zip › BMo086_boxplot.png]

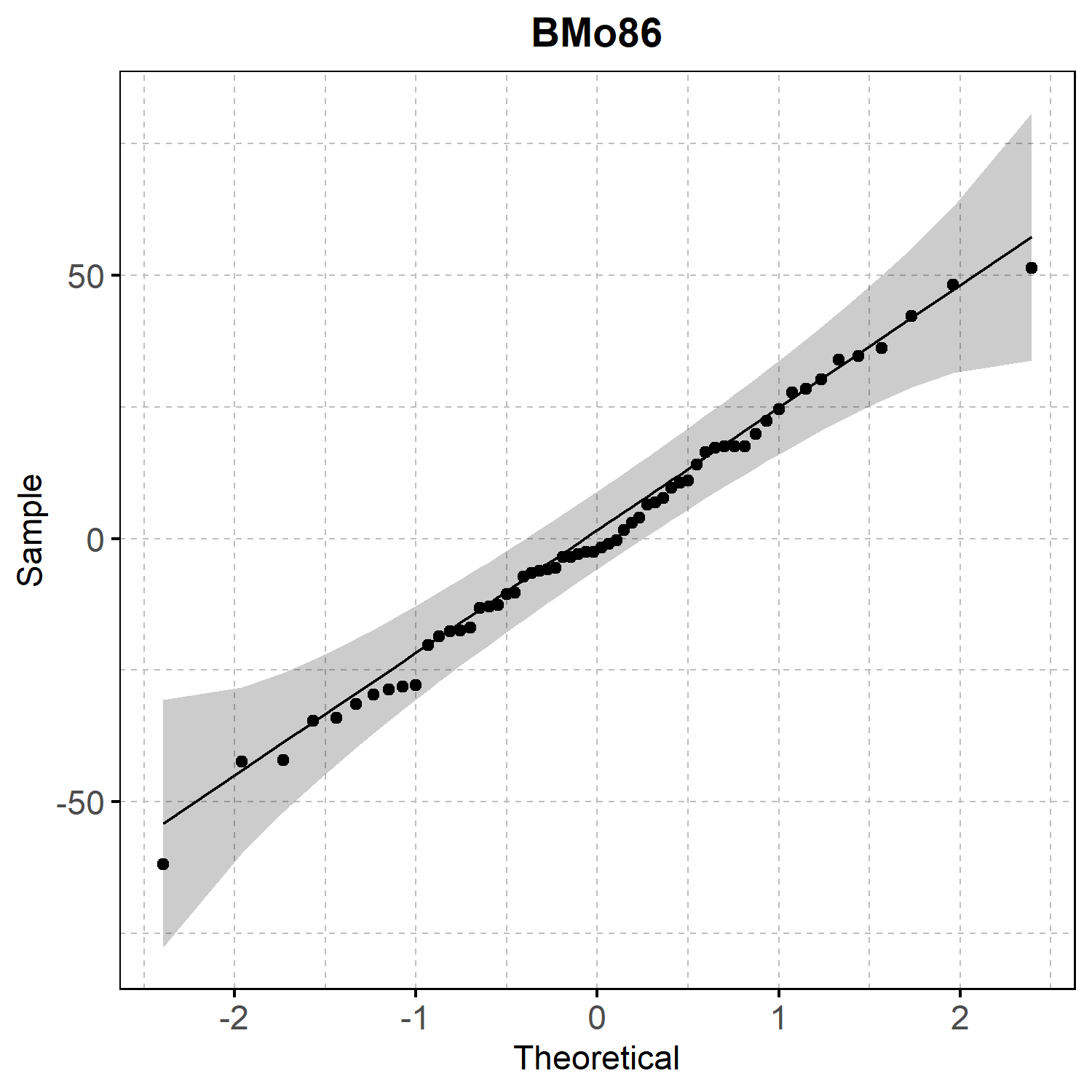

Supplement: Supplementary file 2 — Supplementary Information 2. [file 41598_2023_33504_MOESM2_ESM.zip › BMo086_normality.png]

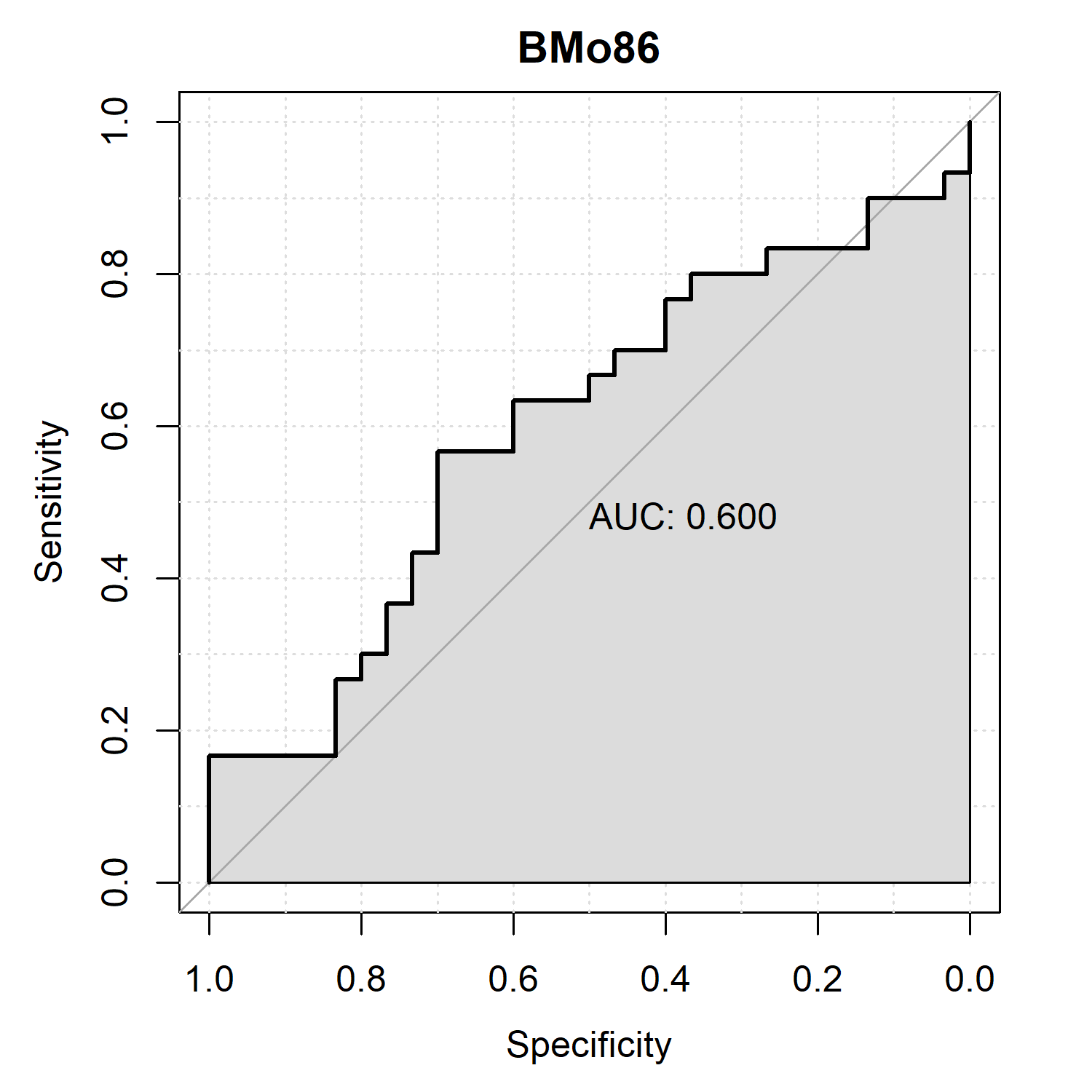

Supplement: Supplementary file 2 — Supplementary Information 2. [file 41598_2023_33504_MOESM2_ESM.zip › BMo086_ROC.png]

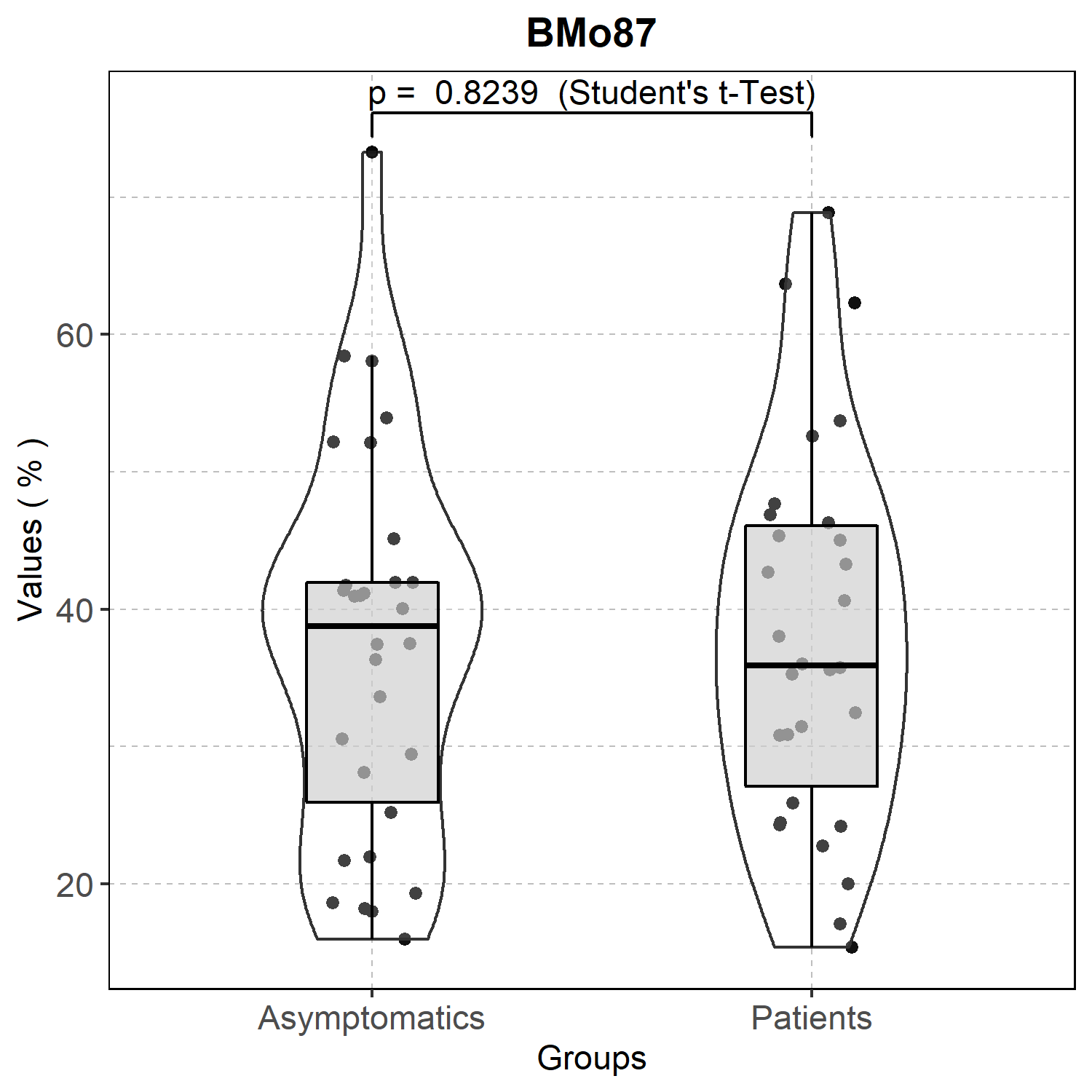

Supplement: Supplementary file 2 — Supplementary Information 2. [file 41598_2023_33504_MOESM2_ESM.zip › BMo087_boxplot.png]

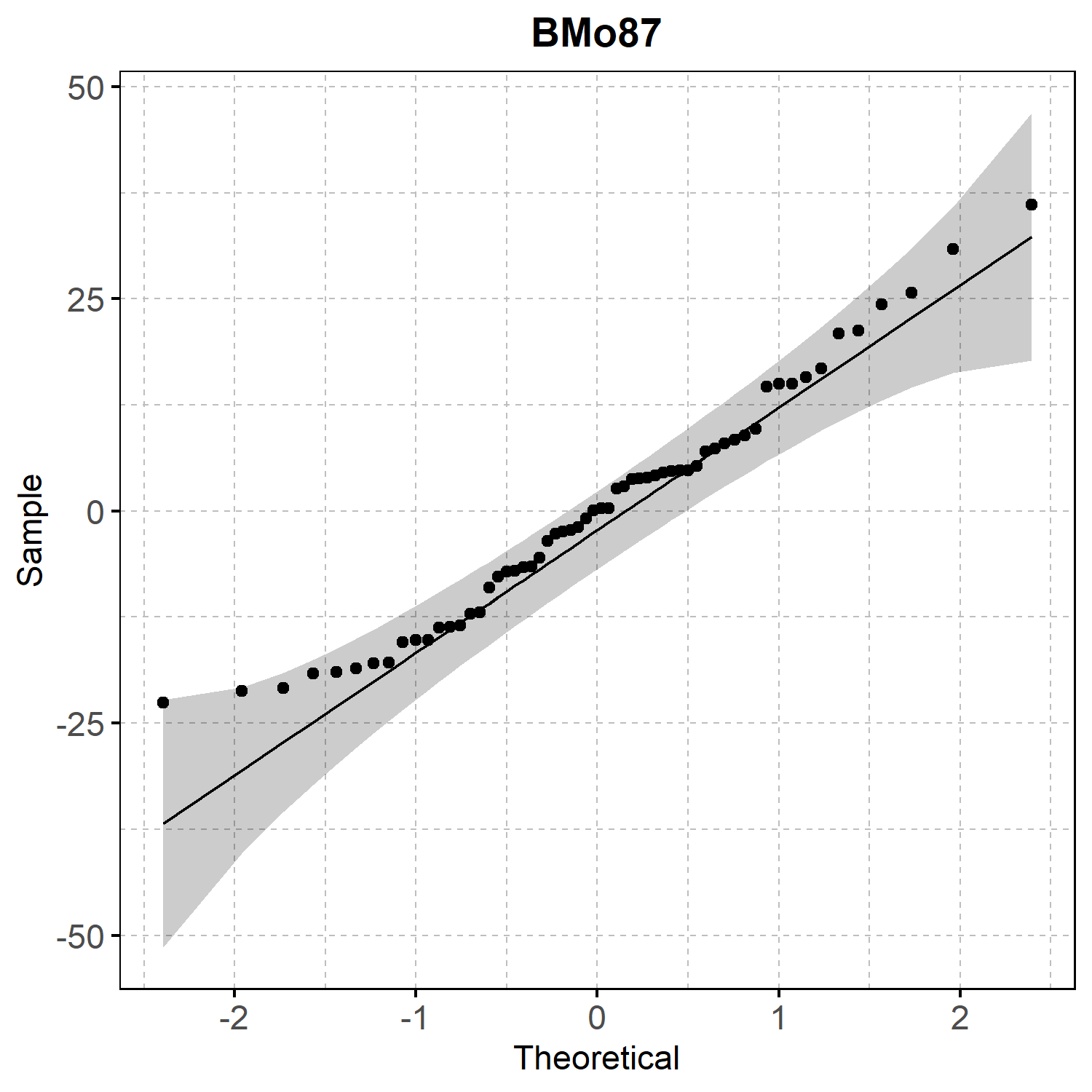

Supplement: Supplementary file 2 — Supplementary Information 2. [file 41598_2023_33504_MOESM2_ESM.zip › BMo087_normality.png]

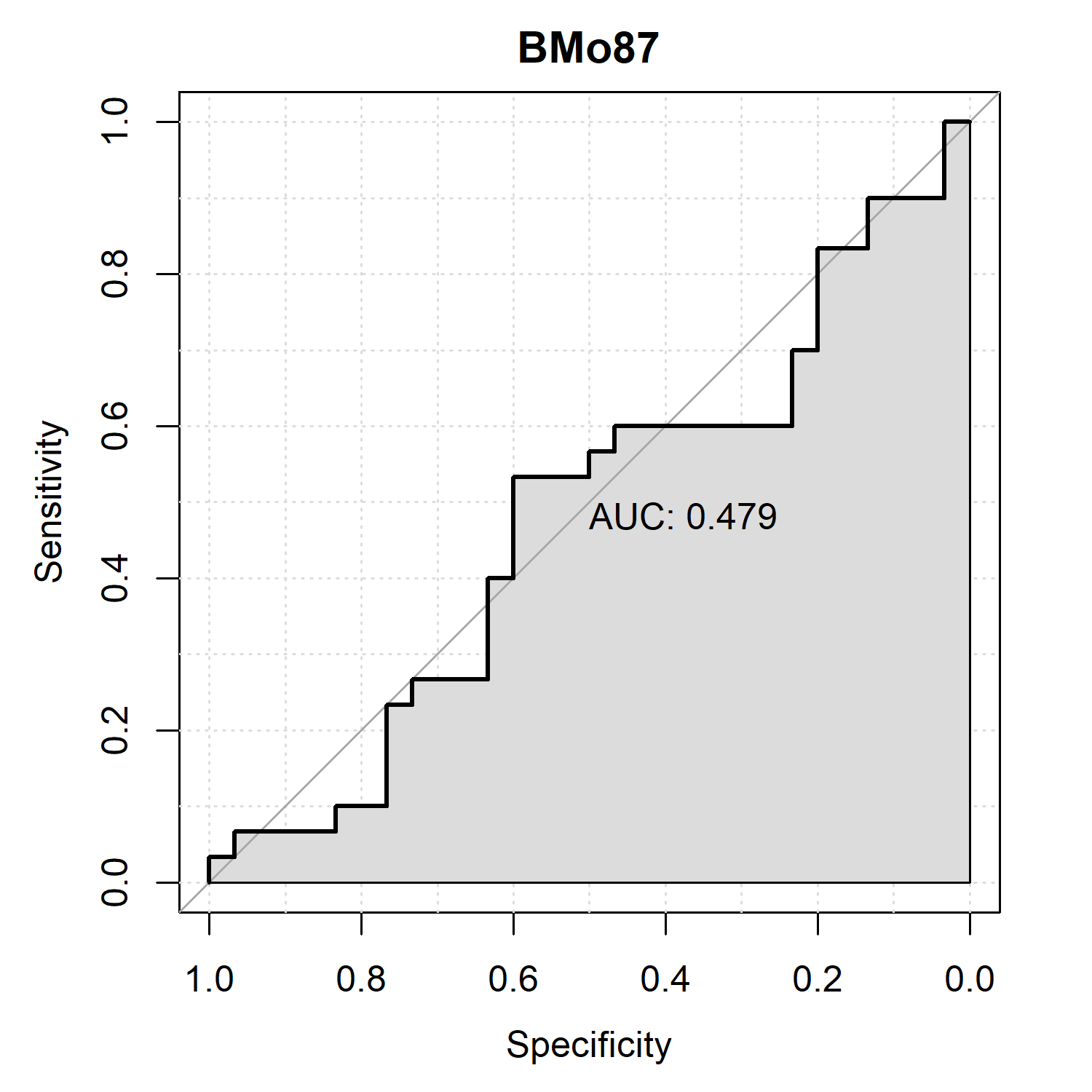

Supplement: Supplementary file 2 — Supplementary Information 2. [file 41598_2023_33504_MOESM2_ESM.zip › BMo087_ROC.png]

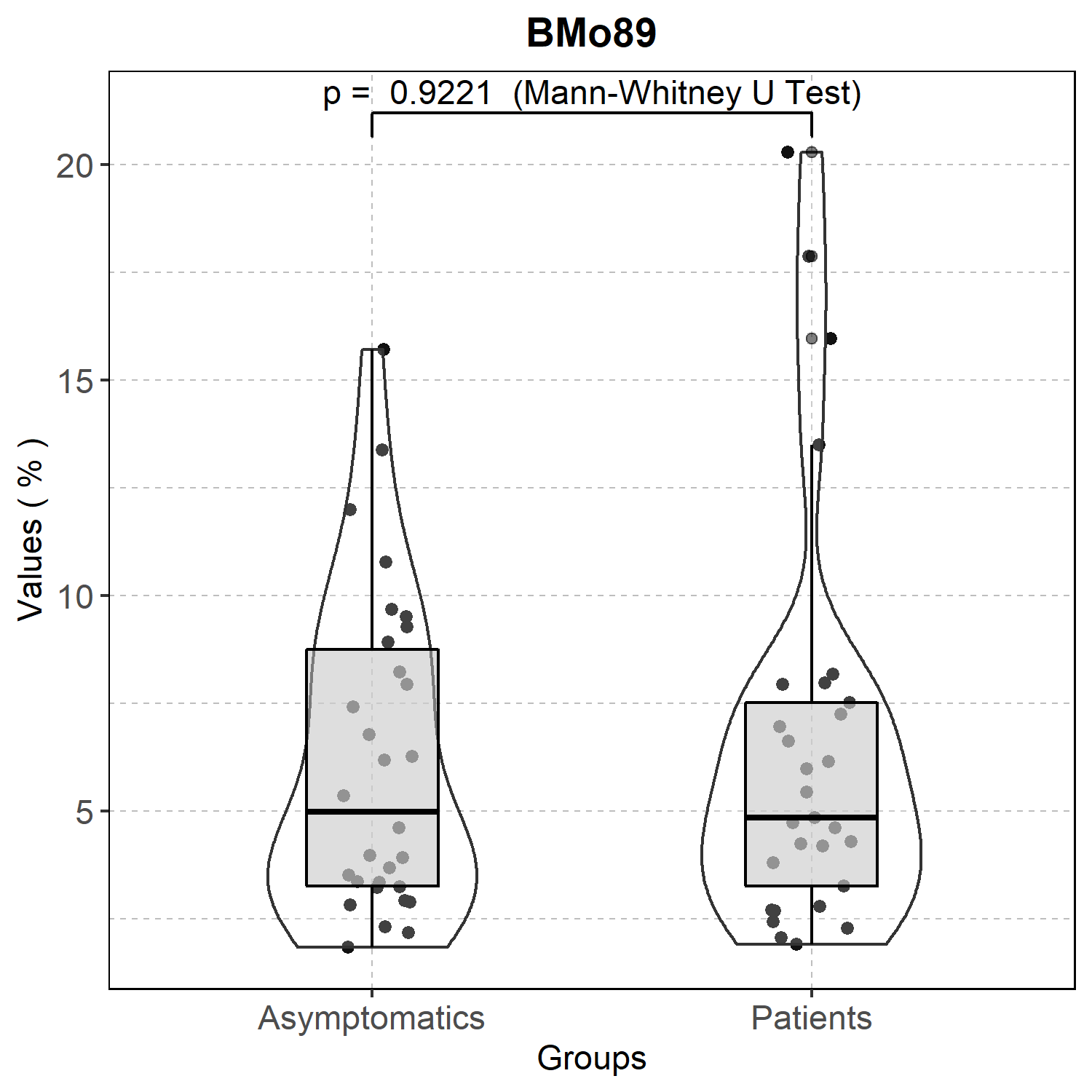

Supplement: Supplementary file 2 — Supplementary Information 2. [file 41598_2023_33504_MOESM2_ESM.zip › BMo089_boxplot.png]

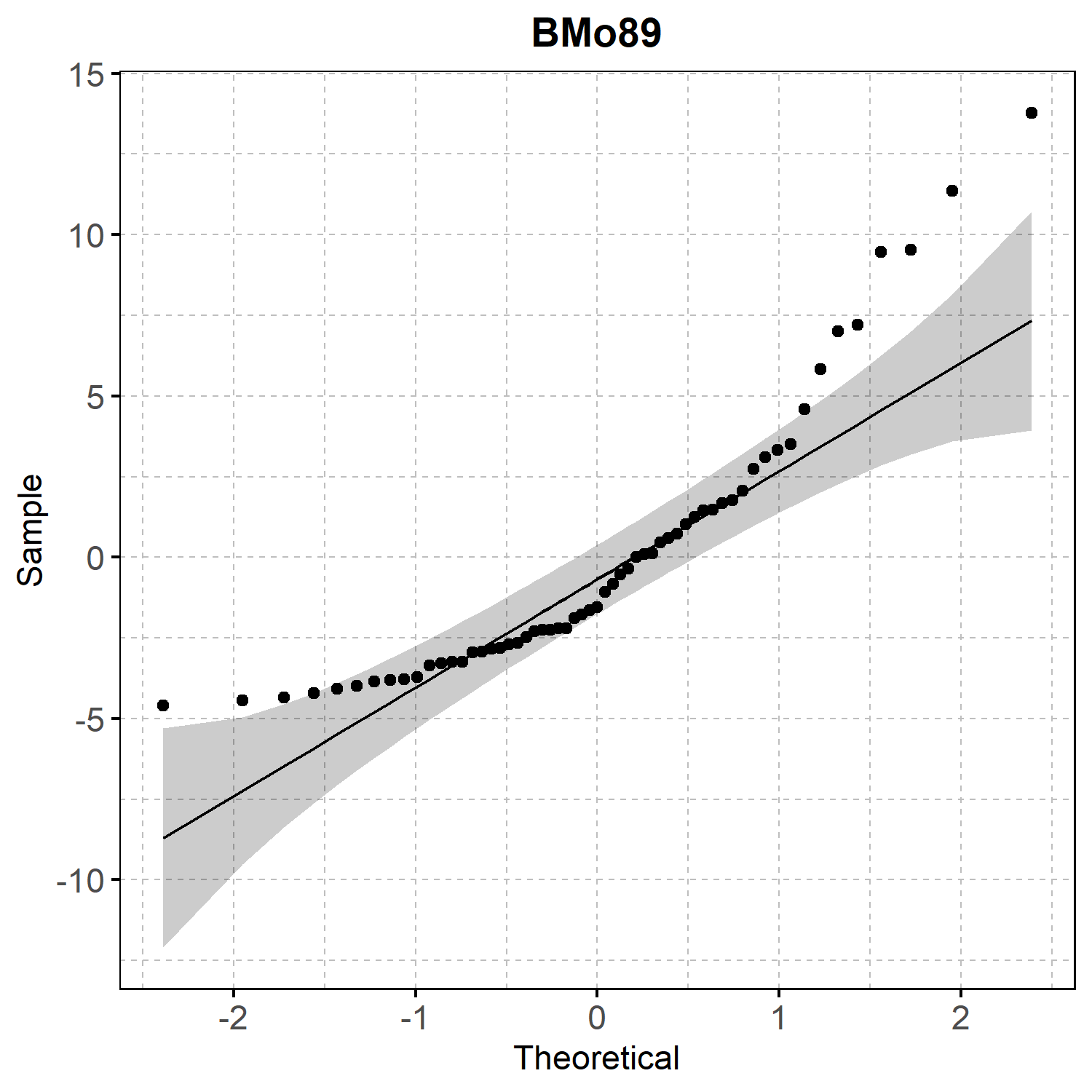

Supplement: Supplementary file 2 — Supplementary Information 2. [file 41598_2023_33504_MOESM2_ESM.zip › BMo089_normality.png]

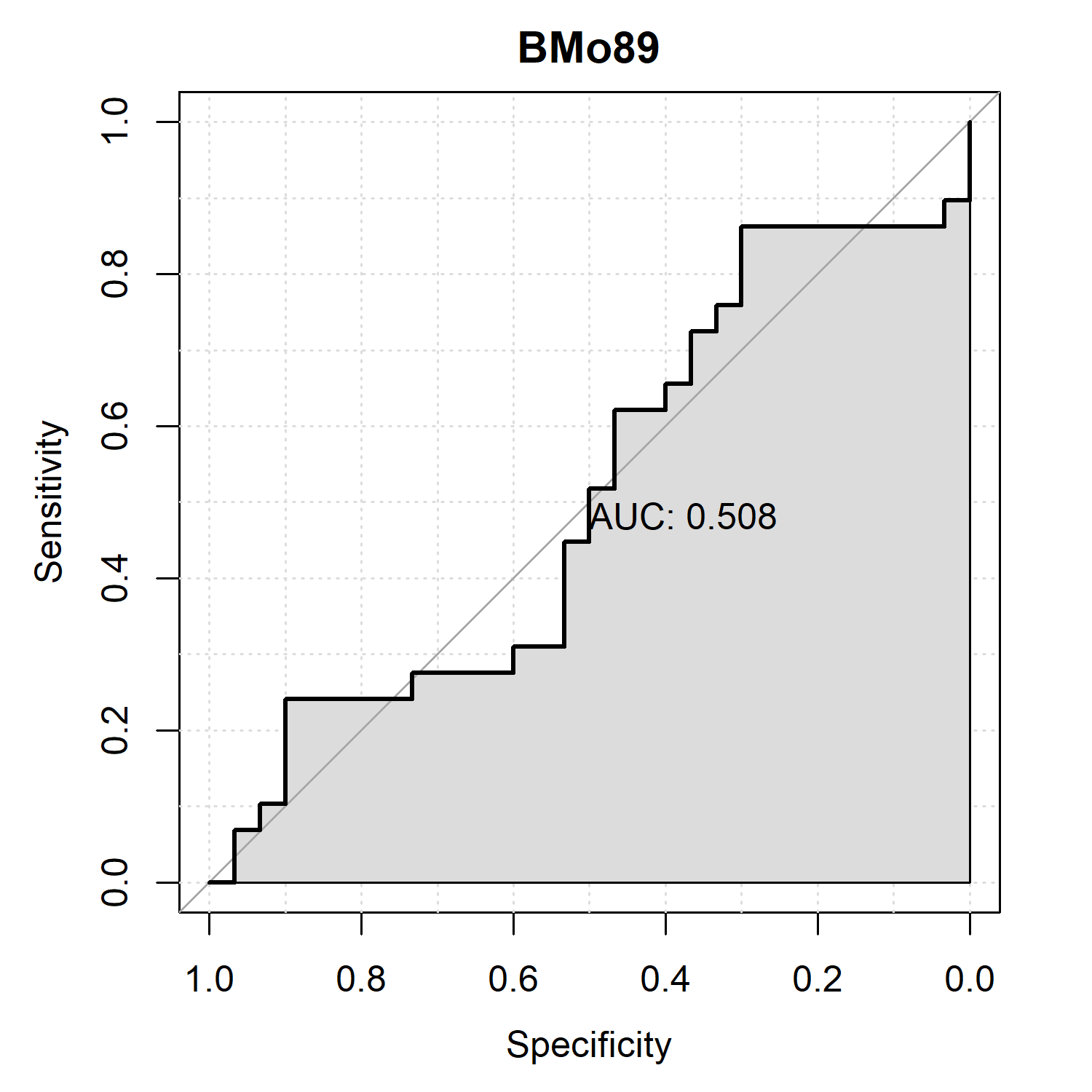

Supplement: Supplementary file 2 — Supplementary Information 2. [file 41598_2023_33504_MOESM2_ESM.zip › BMo089_ROC.png]

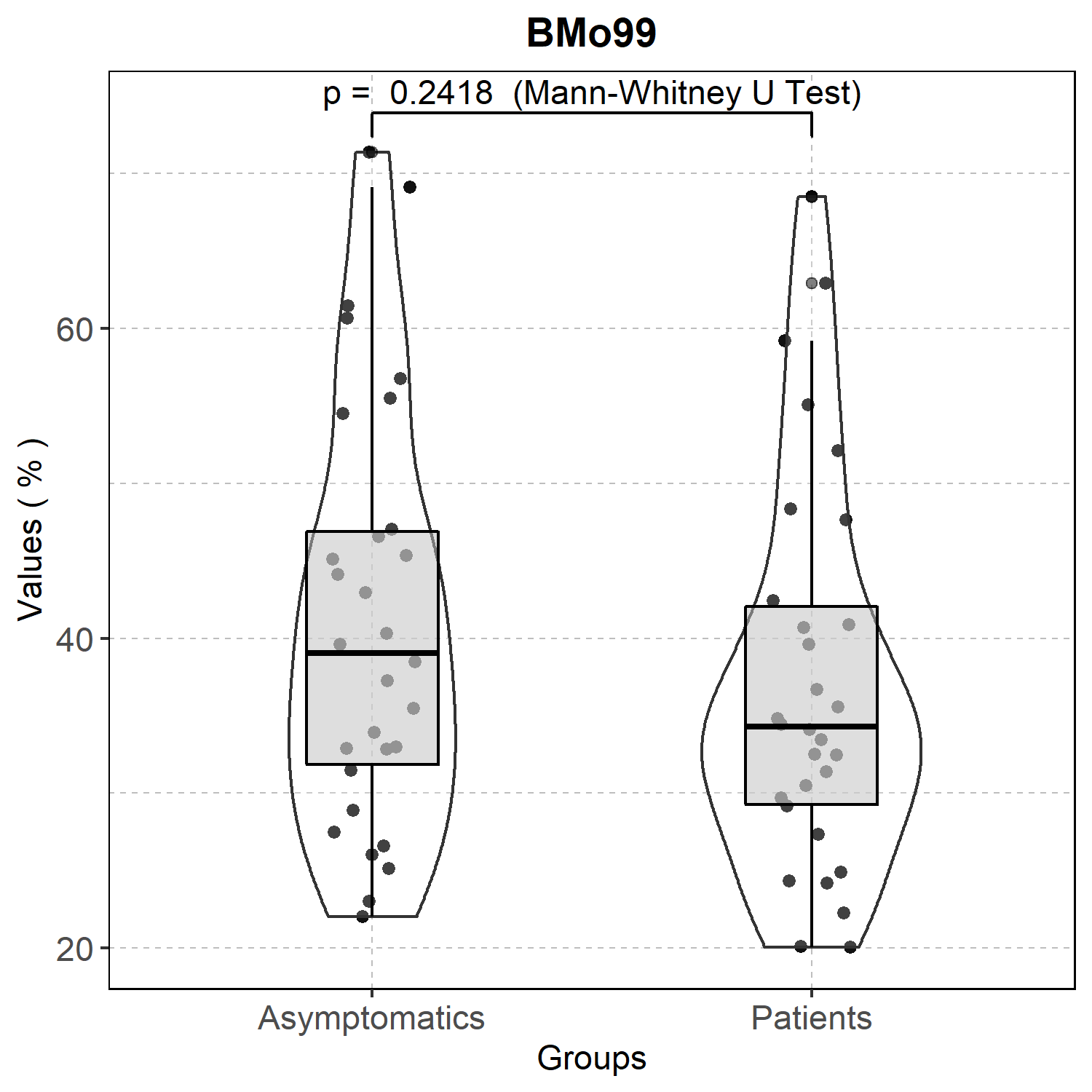

Supplement: Supplementary file 2 — Supplementary Information 2. [file 41598_2023_33504_MOESM2_ESM.zip › BMo099_boxplot.png]

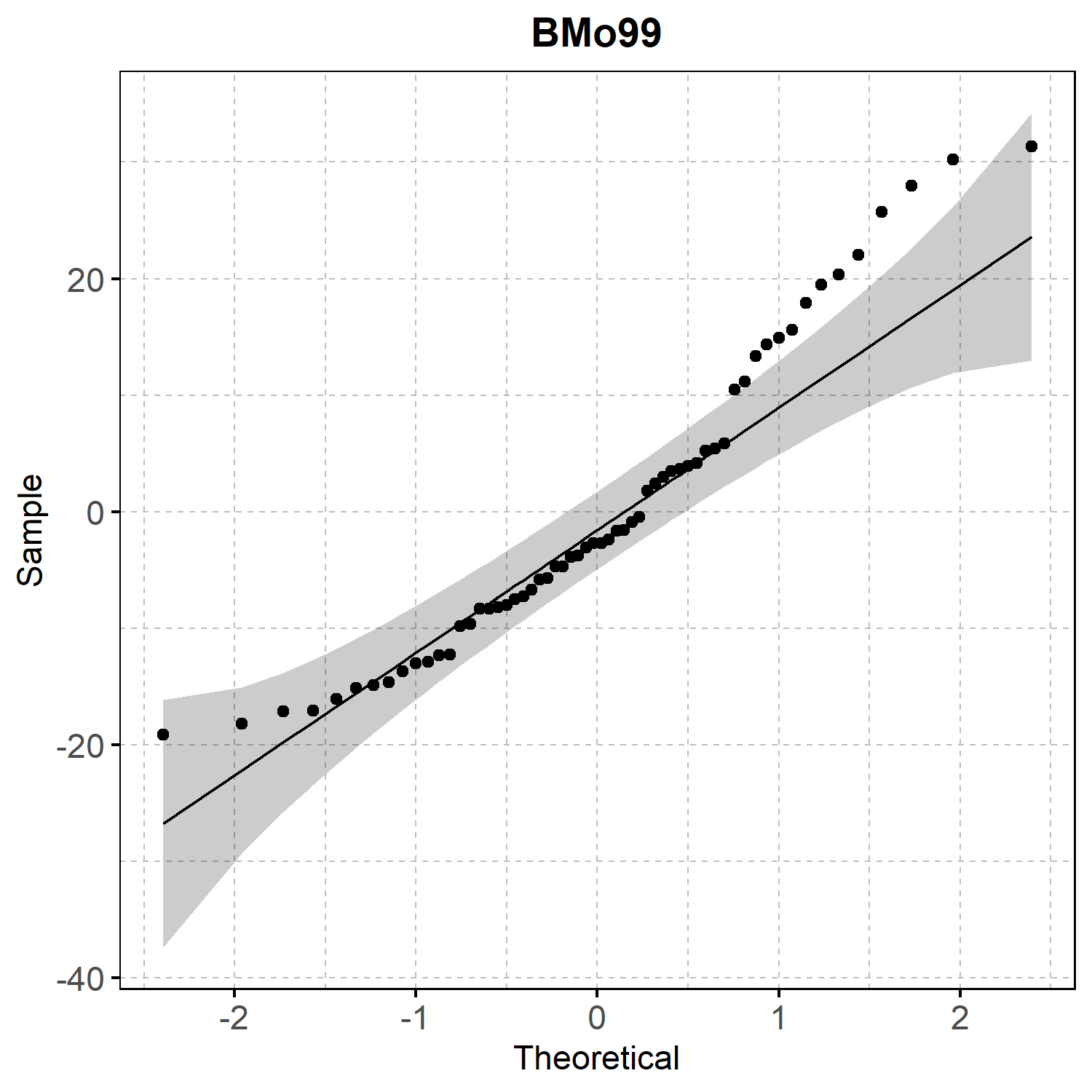

Supplement: Supplementary file 2 — Supplementary Information 2. [file 41598_2023_33504_MOESM2_ESM.zip › BMo099_normality.png]

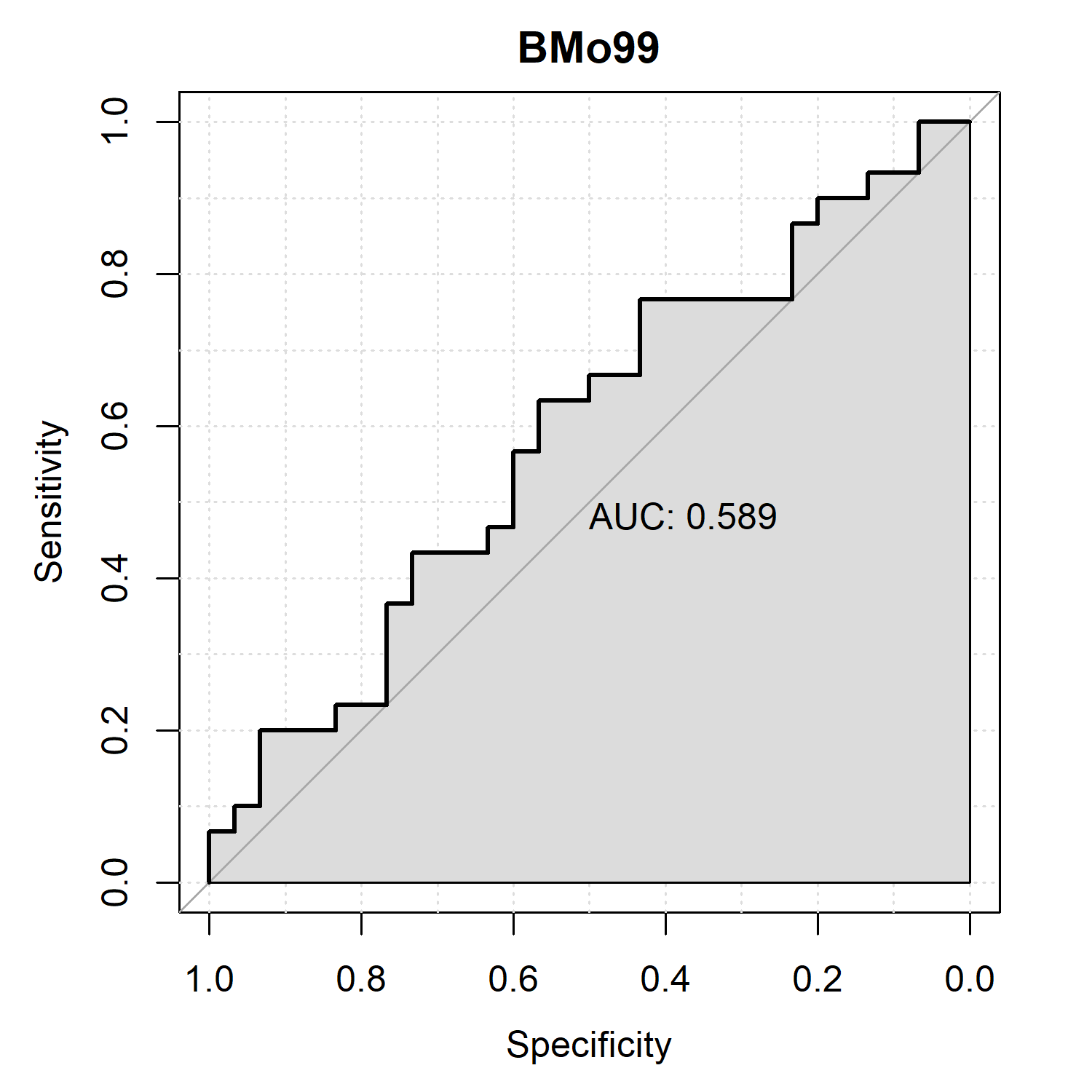

Supplement: Supplementary file 2 — Supplementary Information 2. [file 41598_2023_33504_MOESM2_ESM.zip › BMo099_ROC.png]

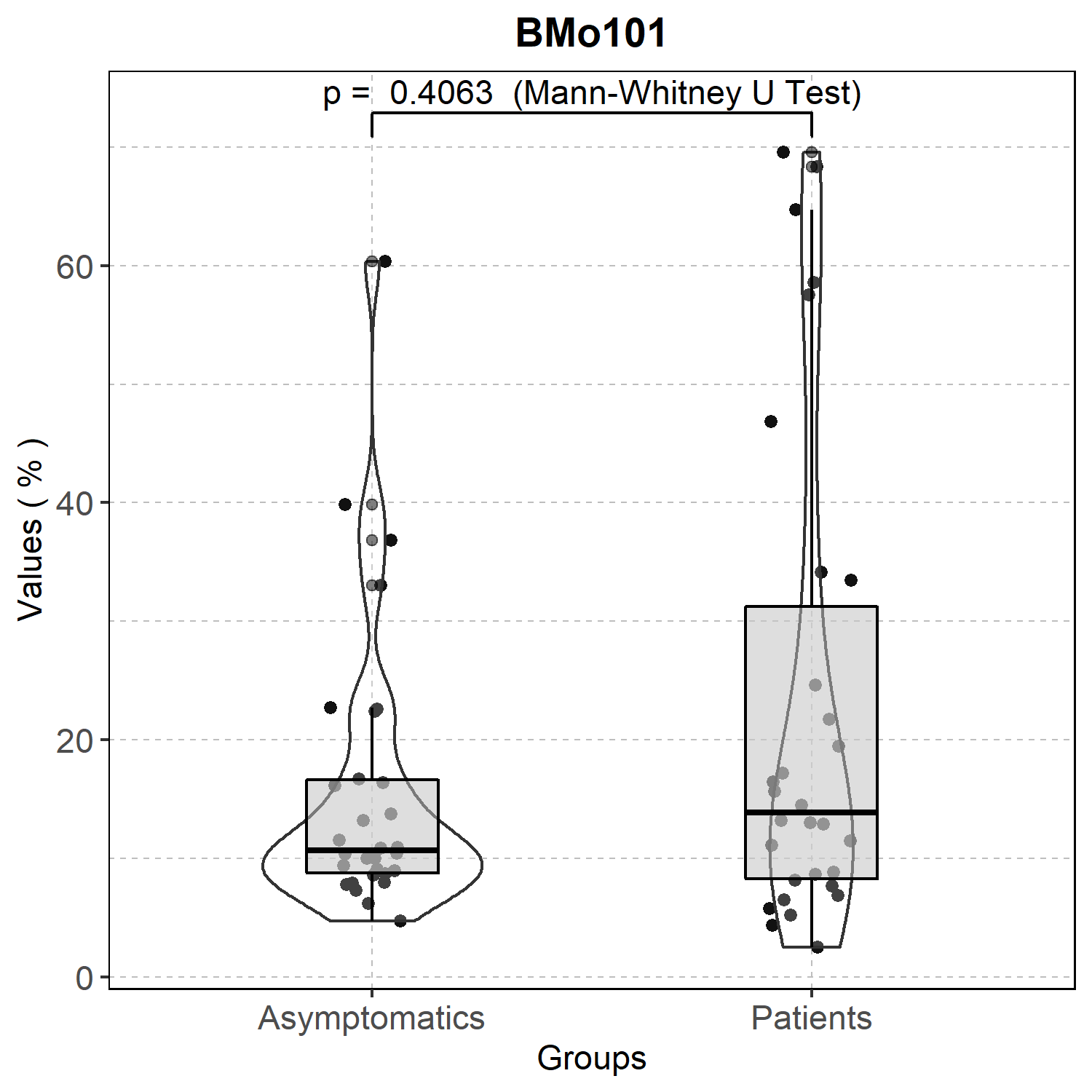

Supplement: Supplementary file 2 — Supplementary Information 2. [file 41598_2023_33504_MOESM2_ESM.zip › BMo101_boxplot.png]

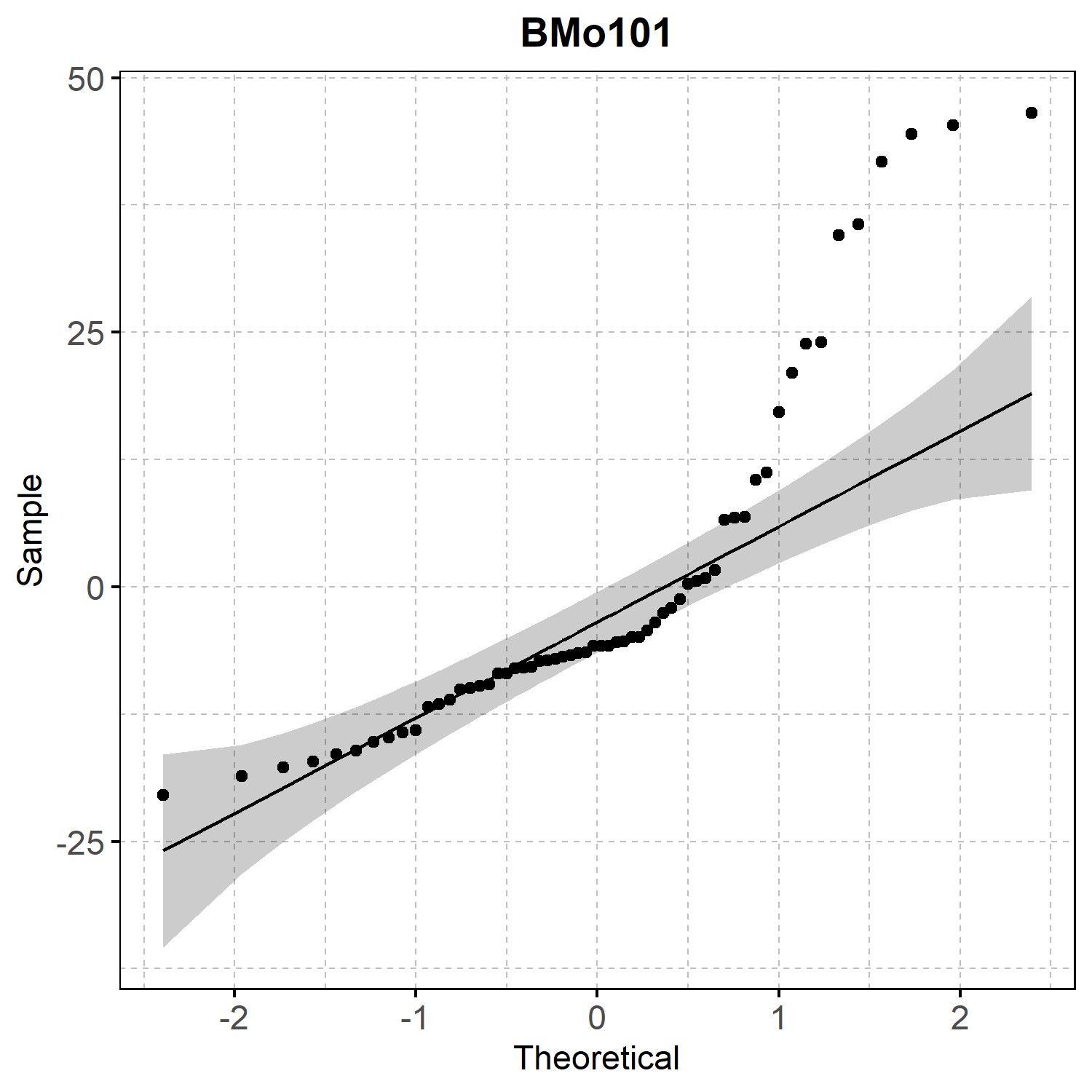

Supplement: Supplementary file 2 — Supplementary Information 2. [file 41598_2023_33504_MOESM2_ESM.zip › BMo101_normality.png]

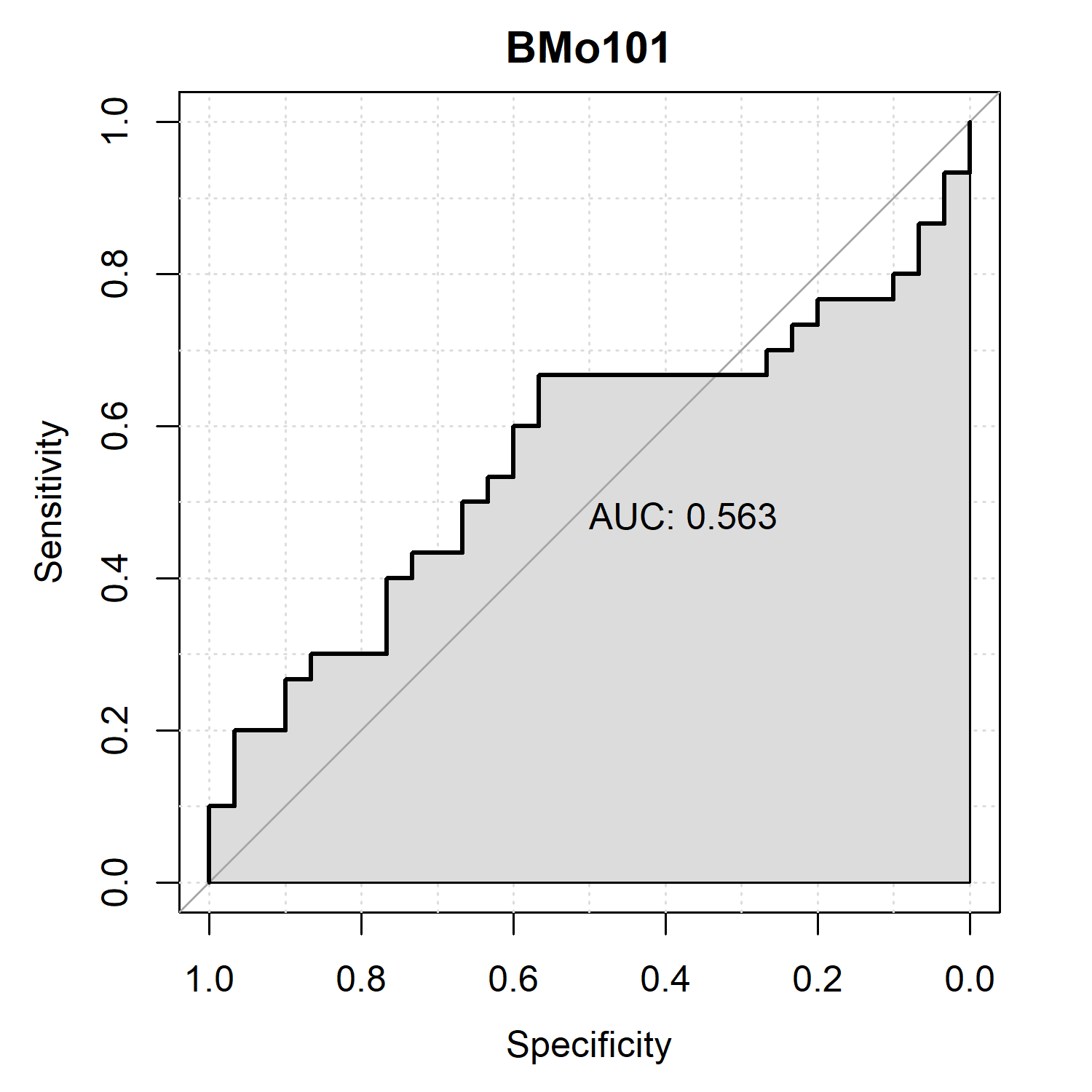

Supplement: Supplementary file 2 — Supplementary Information 2. [file 41598_2023_33504_MOESM2_ESM.zip › BMo101_ROC.png]

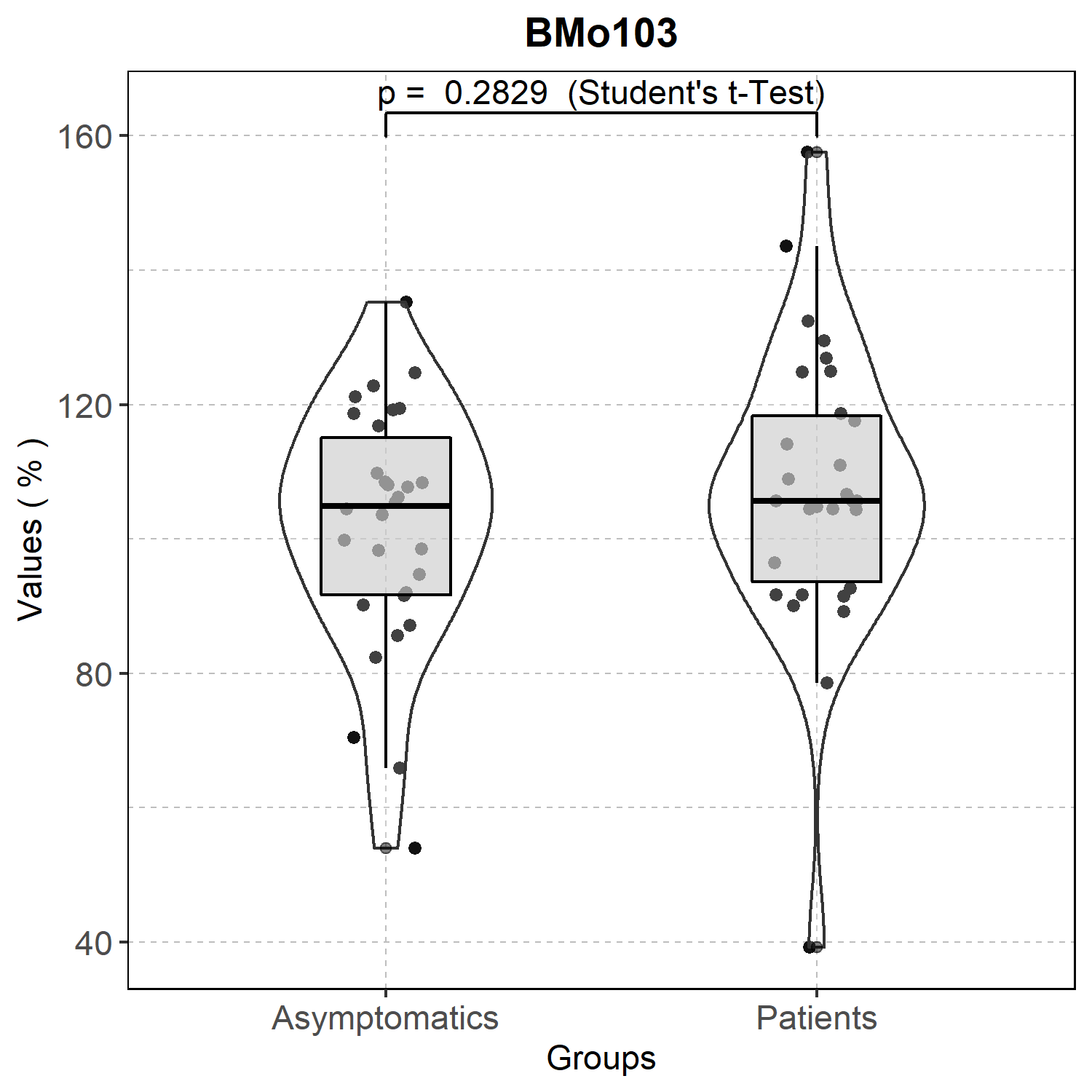

Supplement: Supplementary file 2 — Supplementary Information 2. [file 41598_2023_33504_MOESM2_ESM.zip › BMo103_boxplot.png]

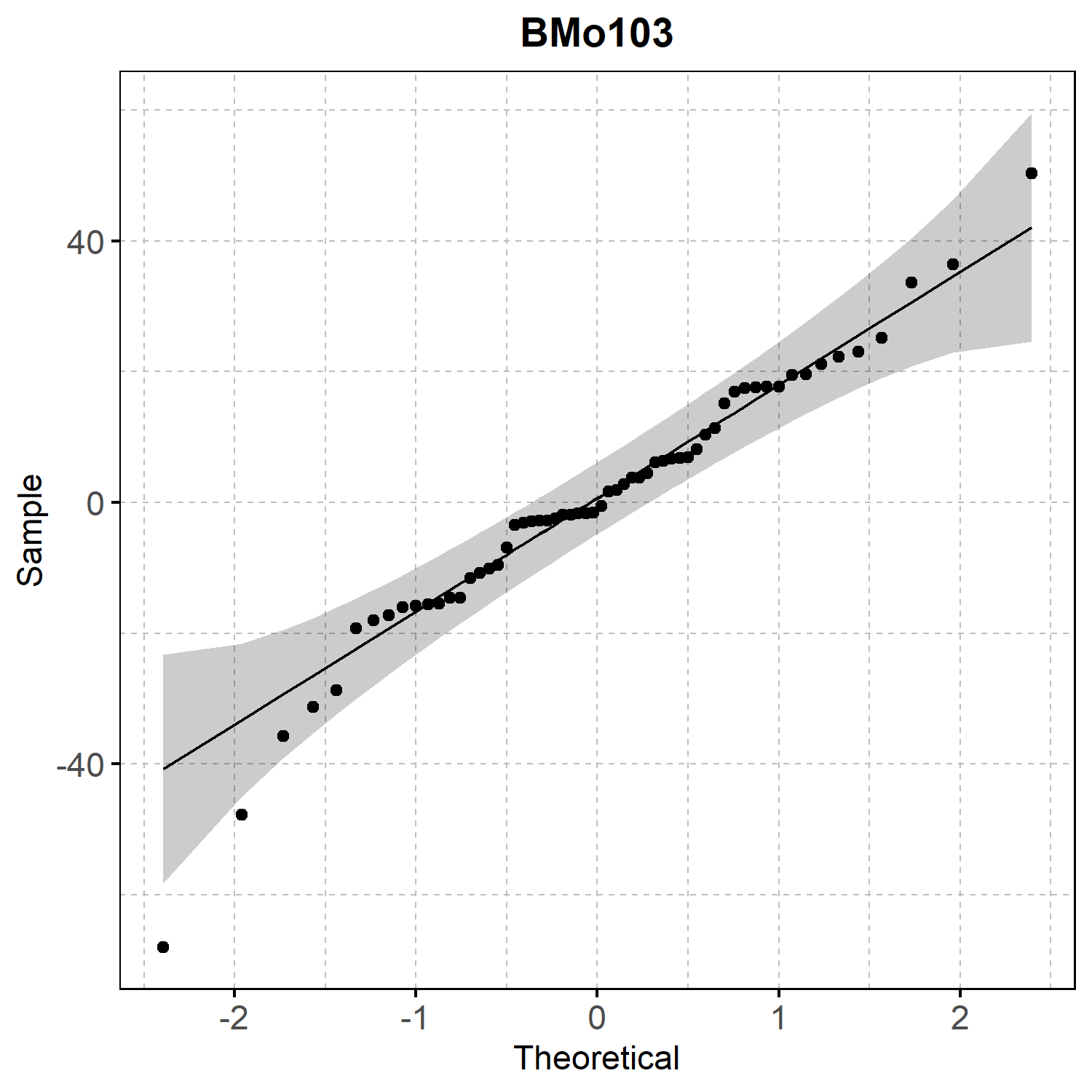

Supplement: Supplementary file 2 — Supplementary Information 2. [file 41598_2023_33504_MOESM2_ESM.zip › BMo103_normality.png]

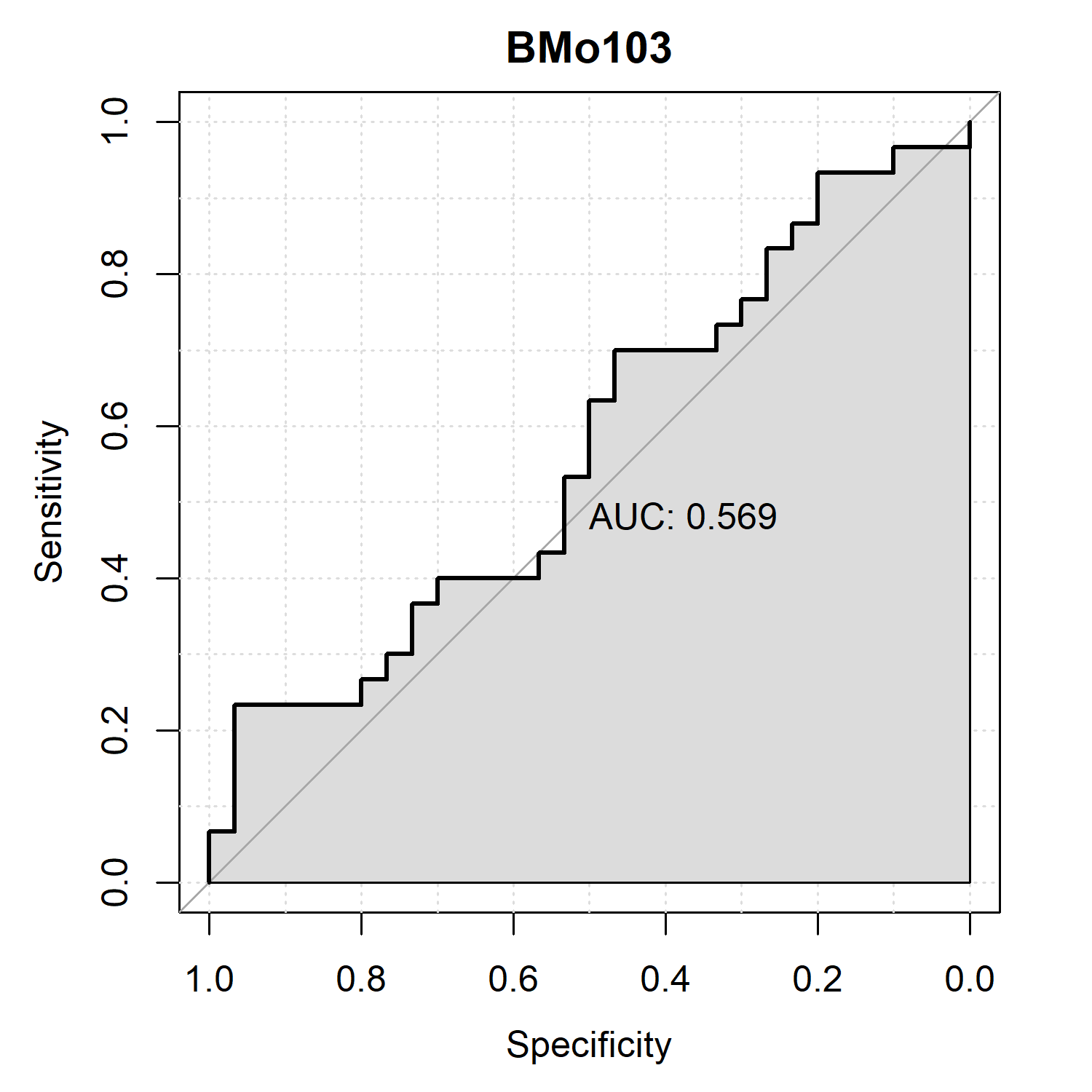

Supplement: Supplementary file 2 — Supplementary Information 2. [file 41598_2023_33504_MOESM2_ESM.zip › BMo103_ROC.png]

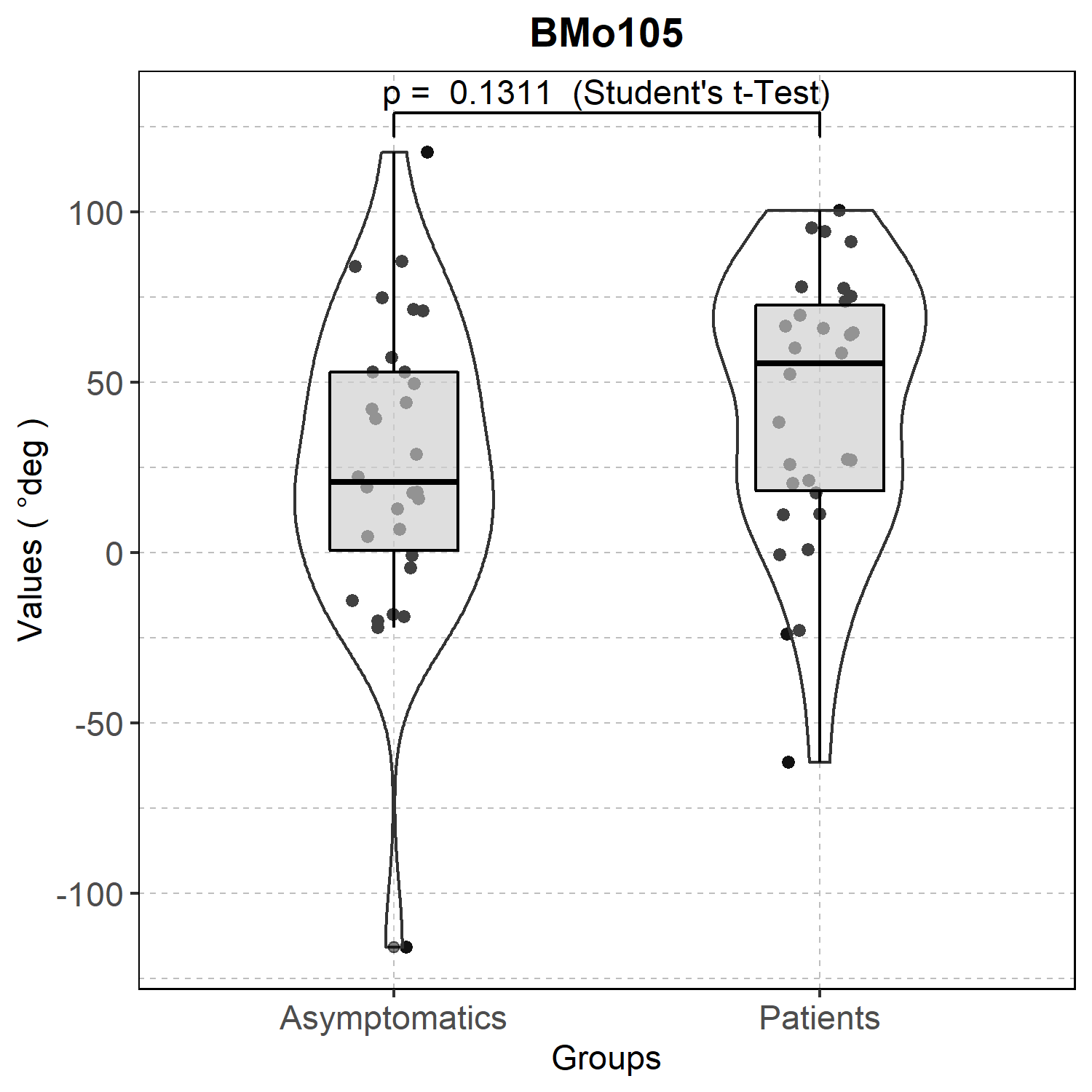

Supplement: Supplementary file 2 — Supplementary Information 2. [file 41598_2023_33504_MOESM2_ESM.zip › BMo105_boxplot.png]

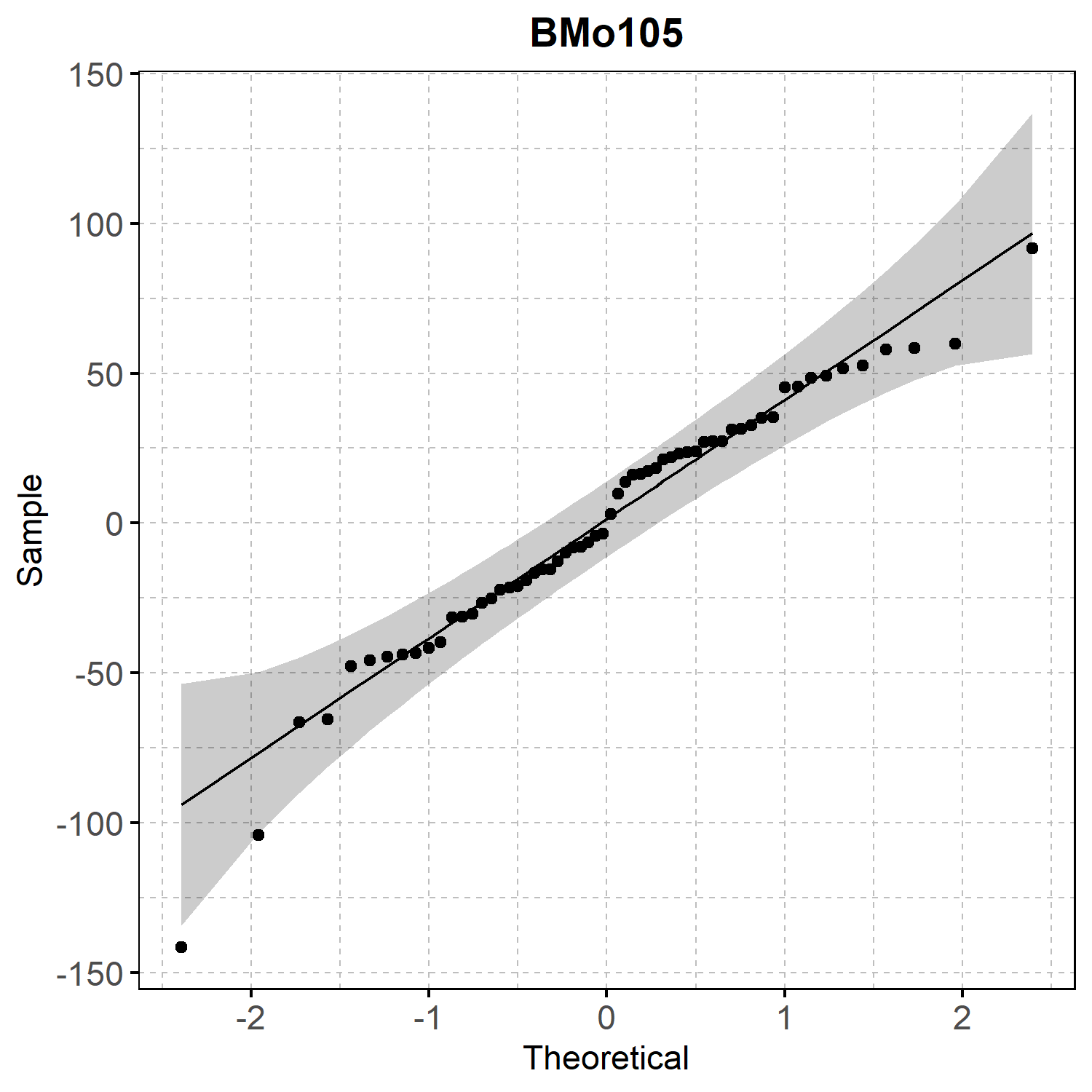

Supplement: Supplementary file 2 — Supplementary Information 2. [file 41598_2023_33504_MOESM2_ESM.zip › BMo105_normality.png]

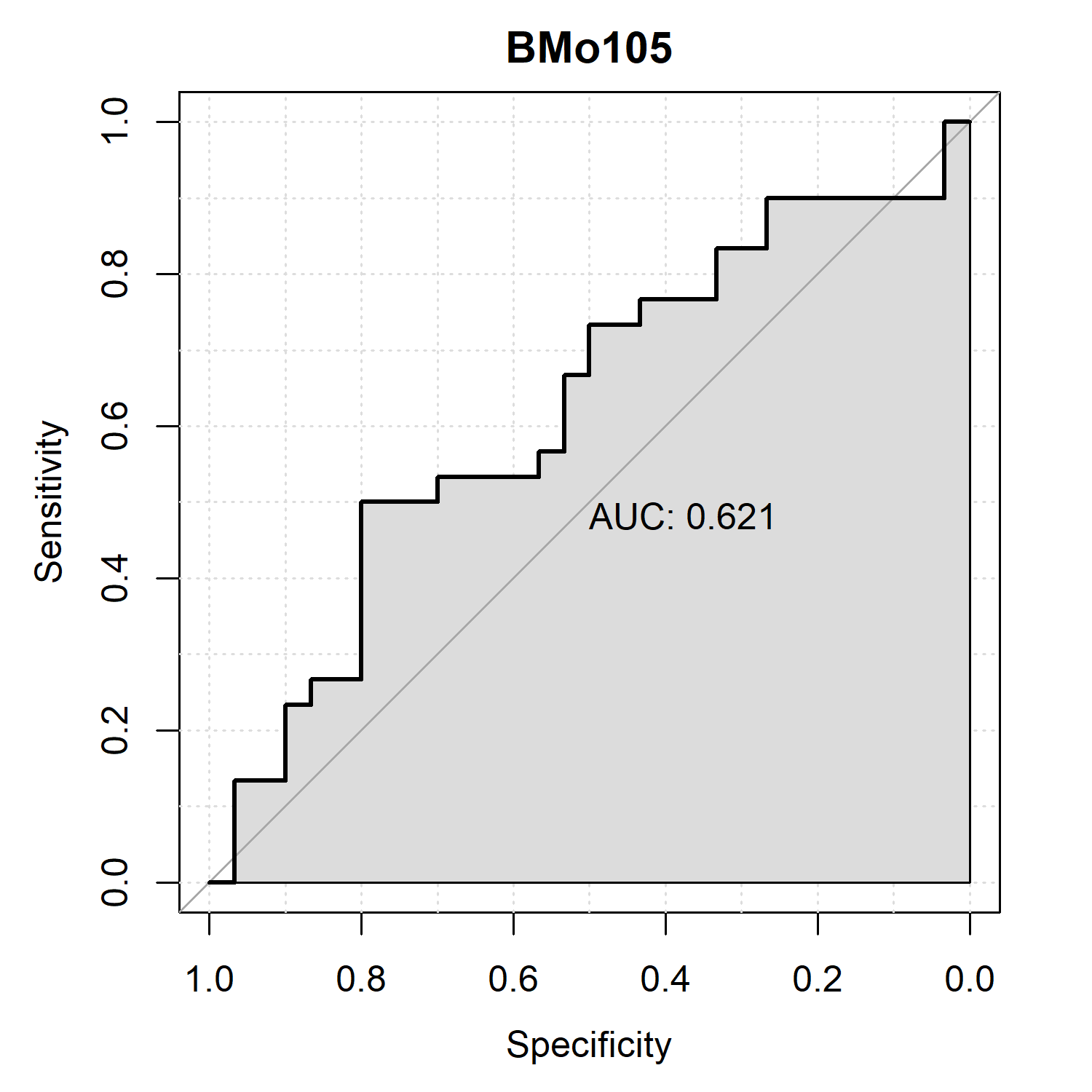

Supplement: Supplementary file 2 — Supplementary Information 2. [file 41598_2023_33504_MOESM2_ESM.zip › BMo105_ROC.png]

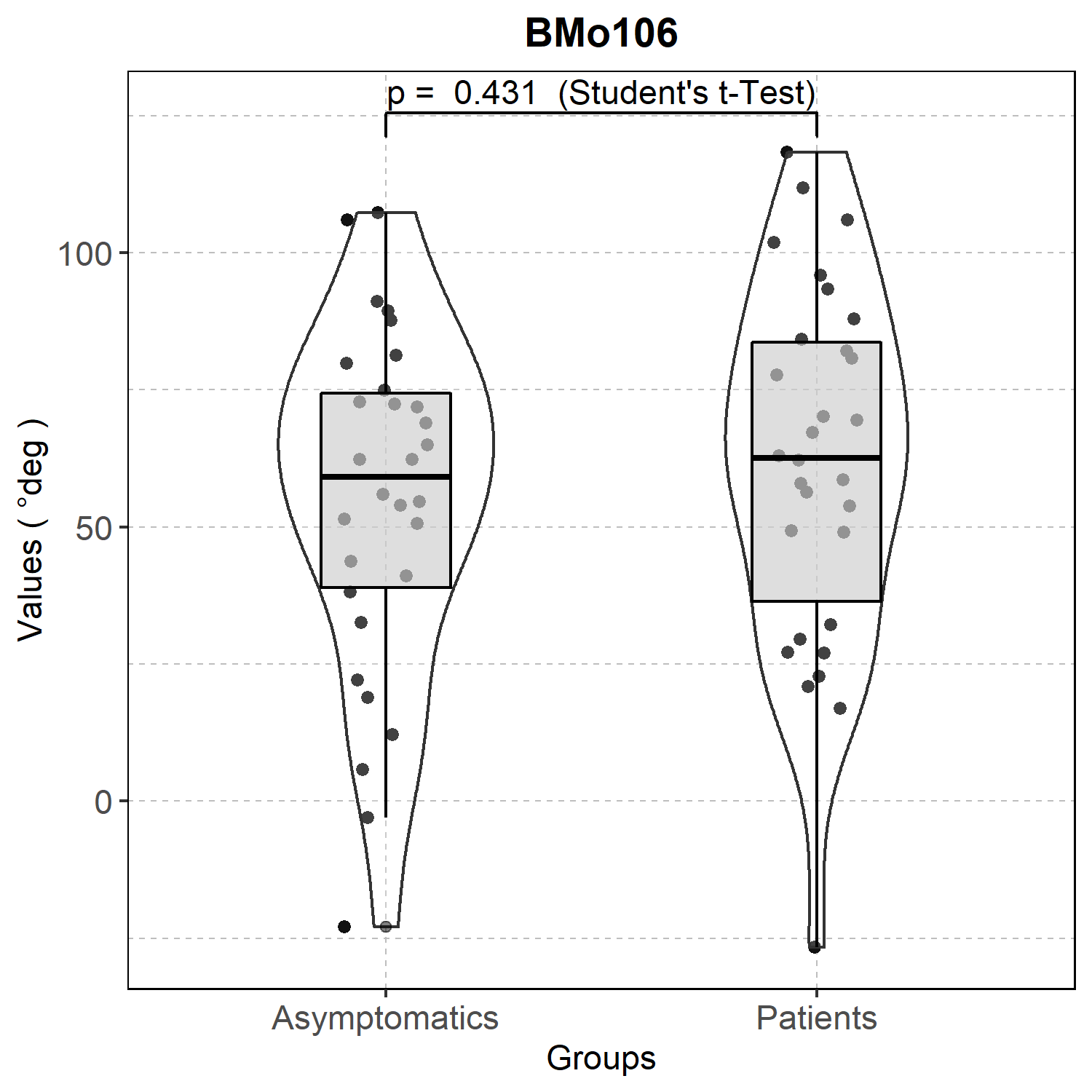

Supplement: Supplementary file 2 — Supplementary Information 2. [file 41598_2023_33504_MOESM2_ESM.zip › BMo106_boxplot.png]

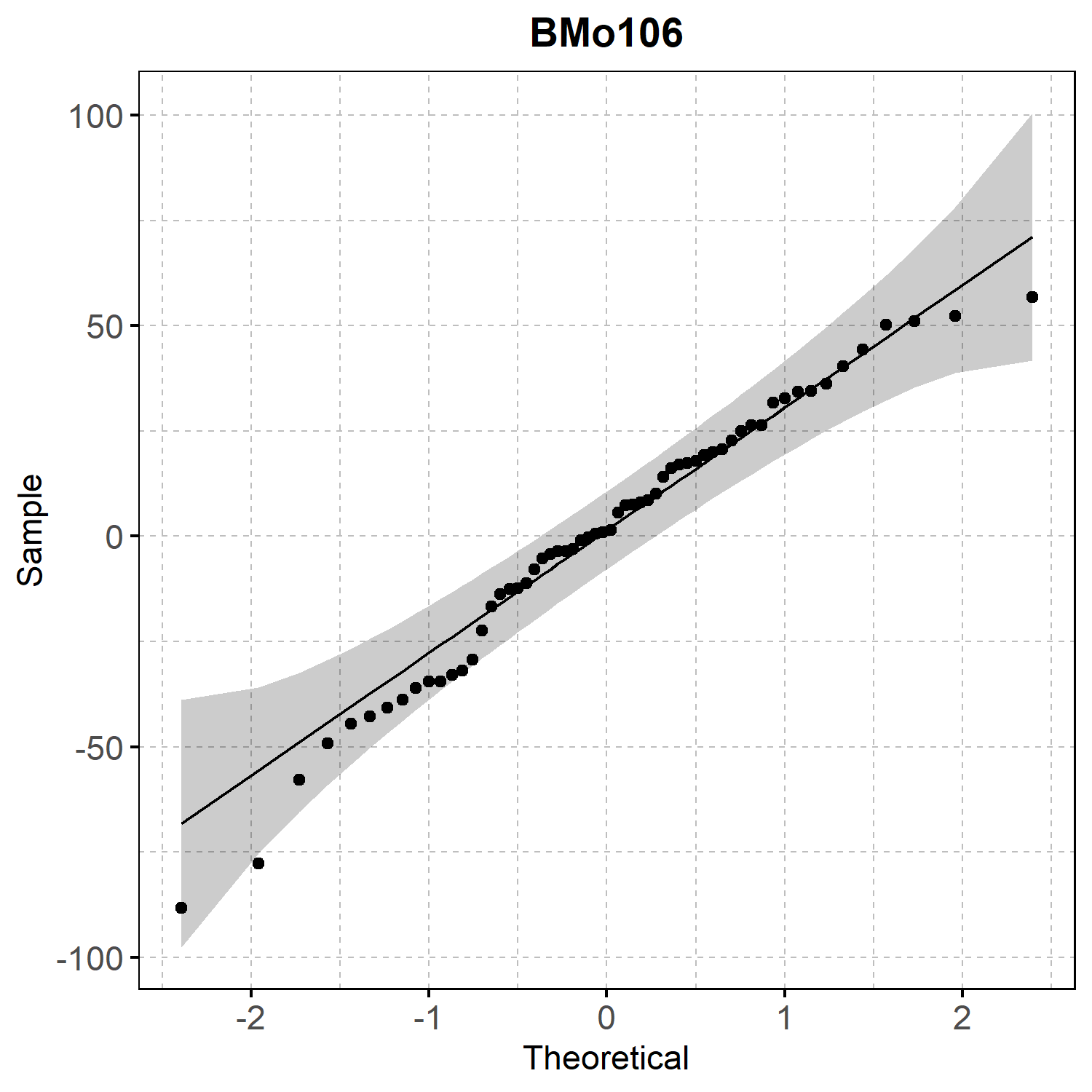

Supplement: Supplementary file 2 — Supplementary Information 2. [file 41598_2023_33504_MOESM2_ESM.zip › BMo106_normality.png]

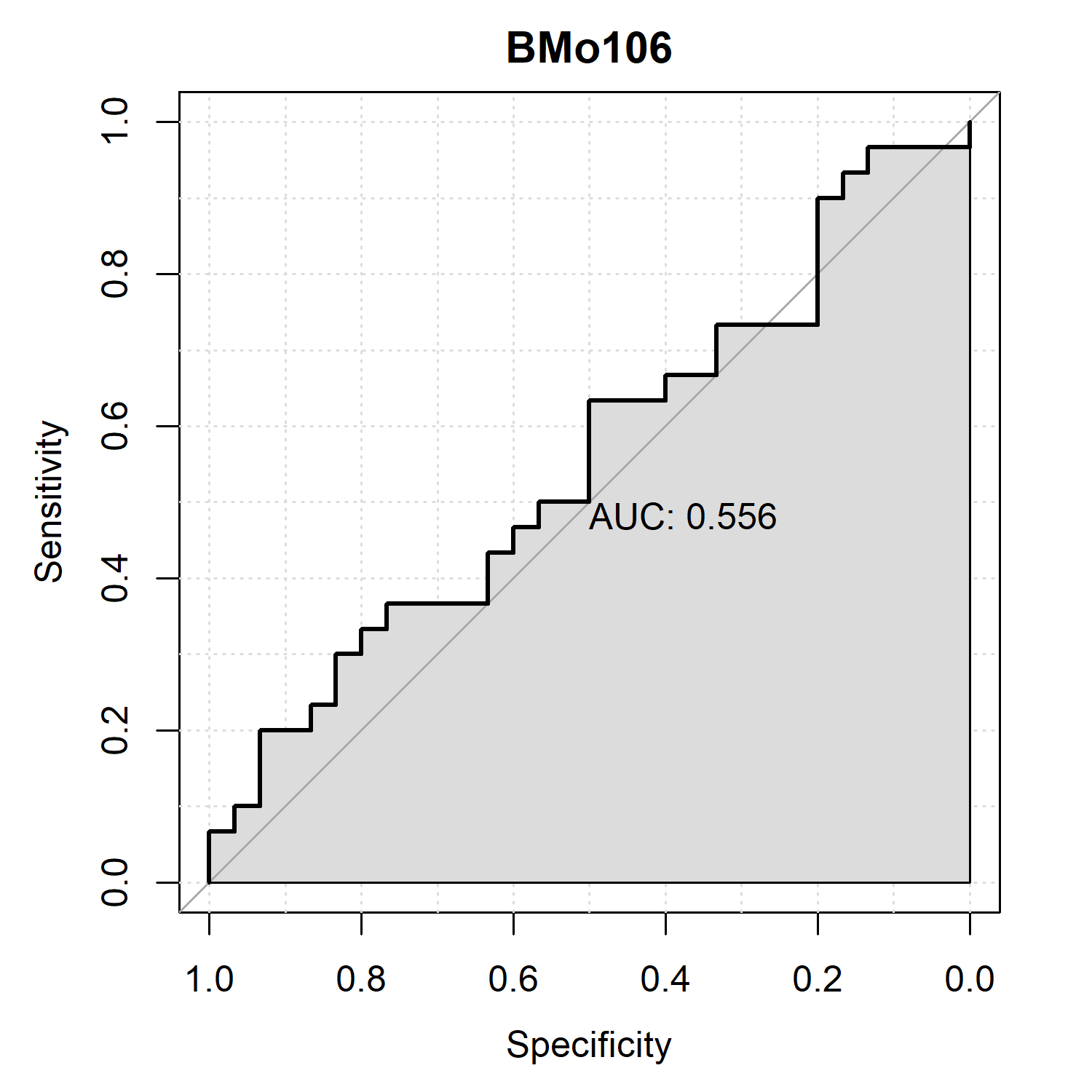

Supplement: Supplementary file 2 — Supplementary Information 2. [file 41598_2023_33504_MOESM2_ESM.zip › BMo106_ROC.png]

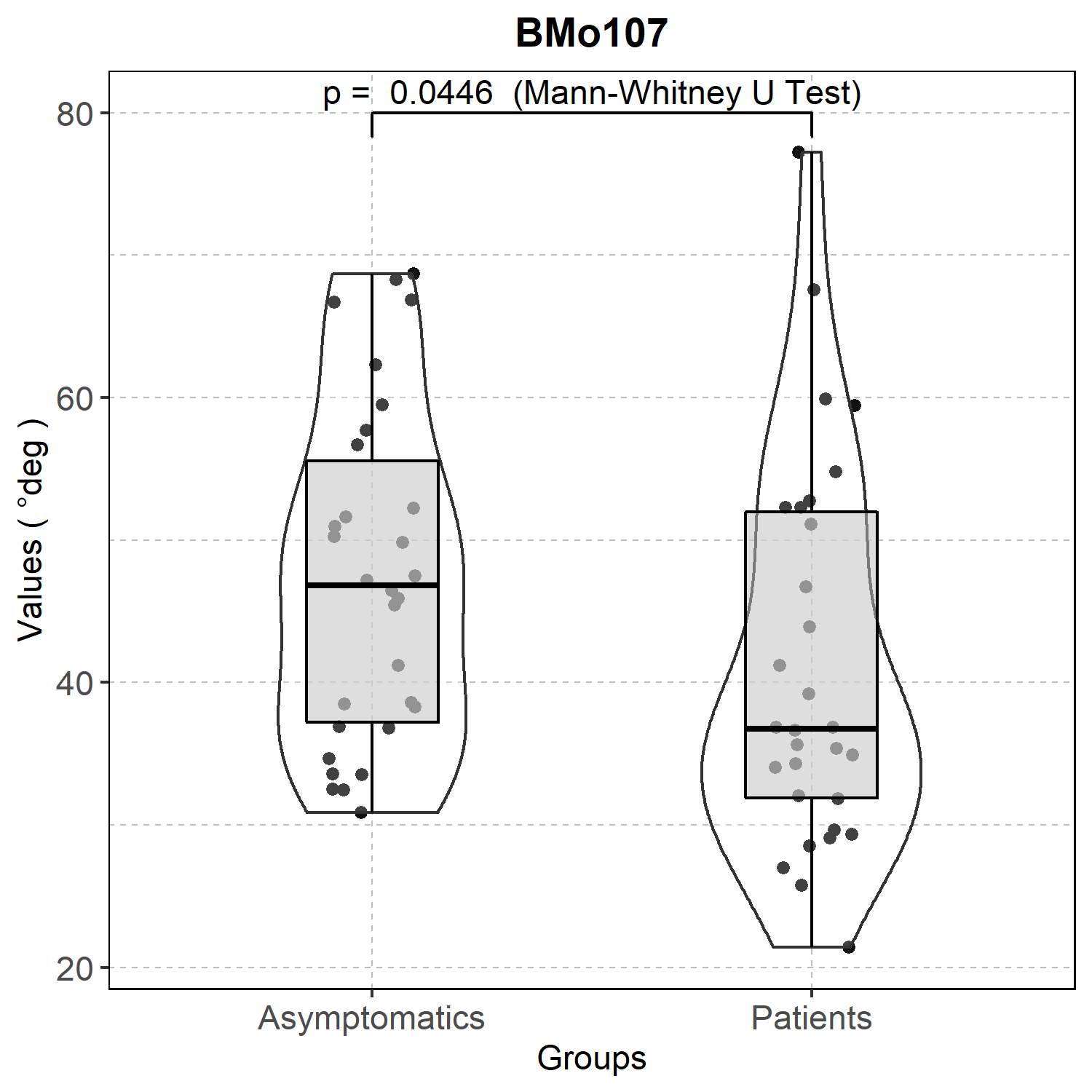

Supplement: Supplementary file 2 — Supplementary Information 2. [file 41598_2023_33504_MOESM2_ESM.zip › BMo107_boxplot.png]

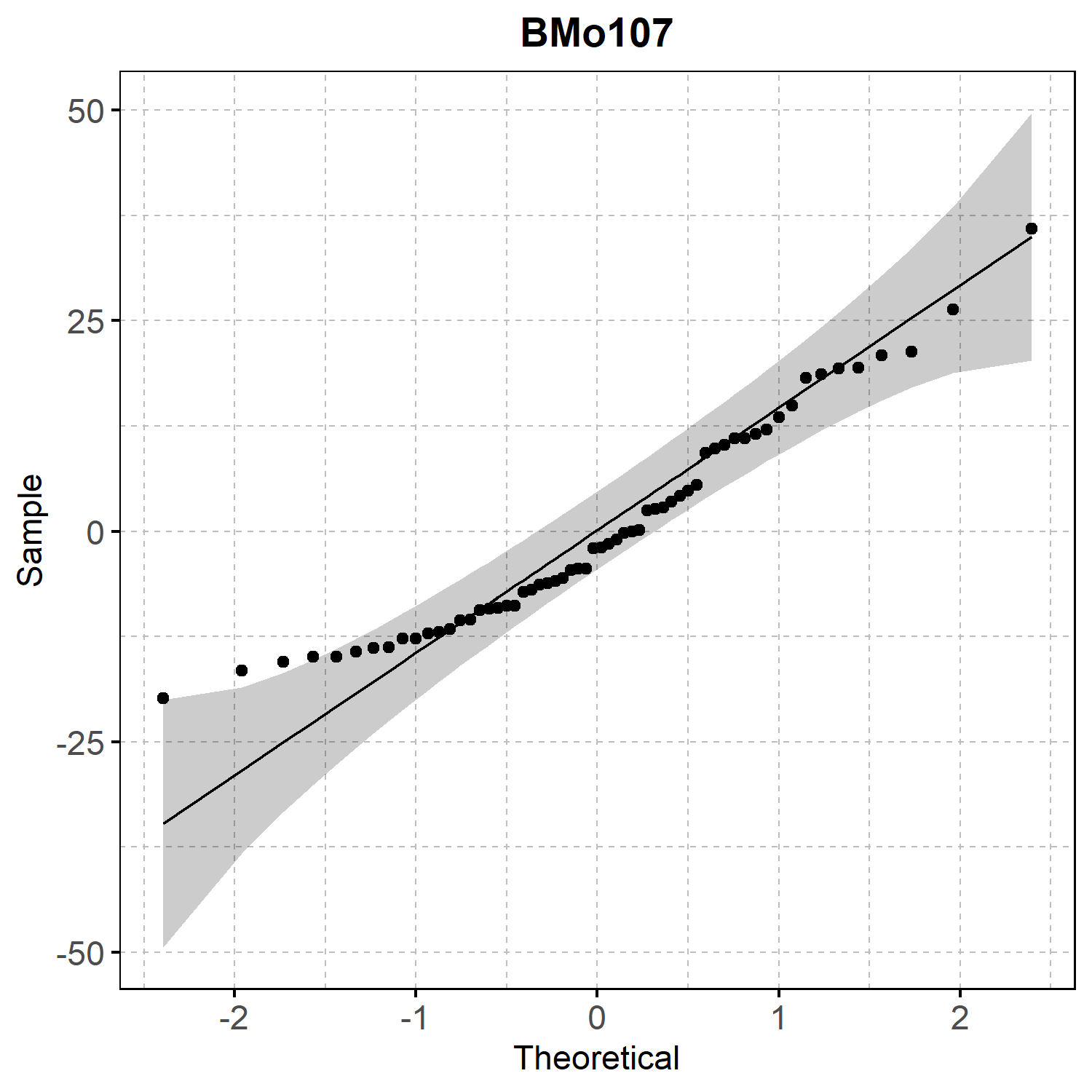

Supplement: Supplementary file 2 — Supplementary Information 2. [file 41598_2023_33504_MOESM2_ESM.zip › BMo107_normality.png]
