# Supplementary figures and images for: Measurement properties of 72 movement biomarkers aiming to discriminate non‑specific chronic low back pain patients from an asymptomatic population (part 1 of 2)
Source: Sci Rep. 2023 Apr 20;13:6483. doi: 10.1038/s41598-023-33504-5 (PMC10119171; doi:10.1038/s41598-023-33504-5)

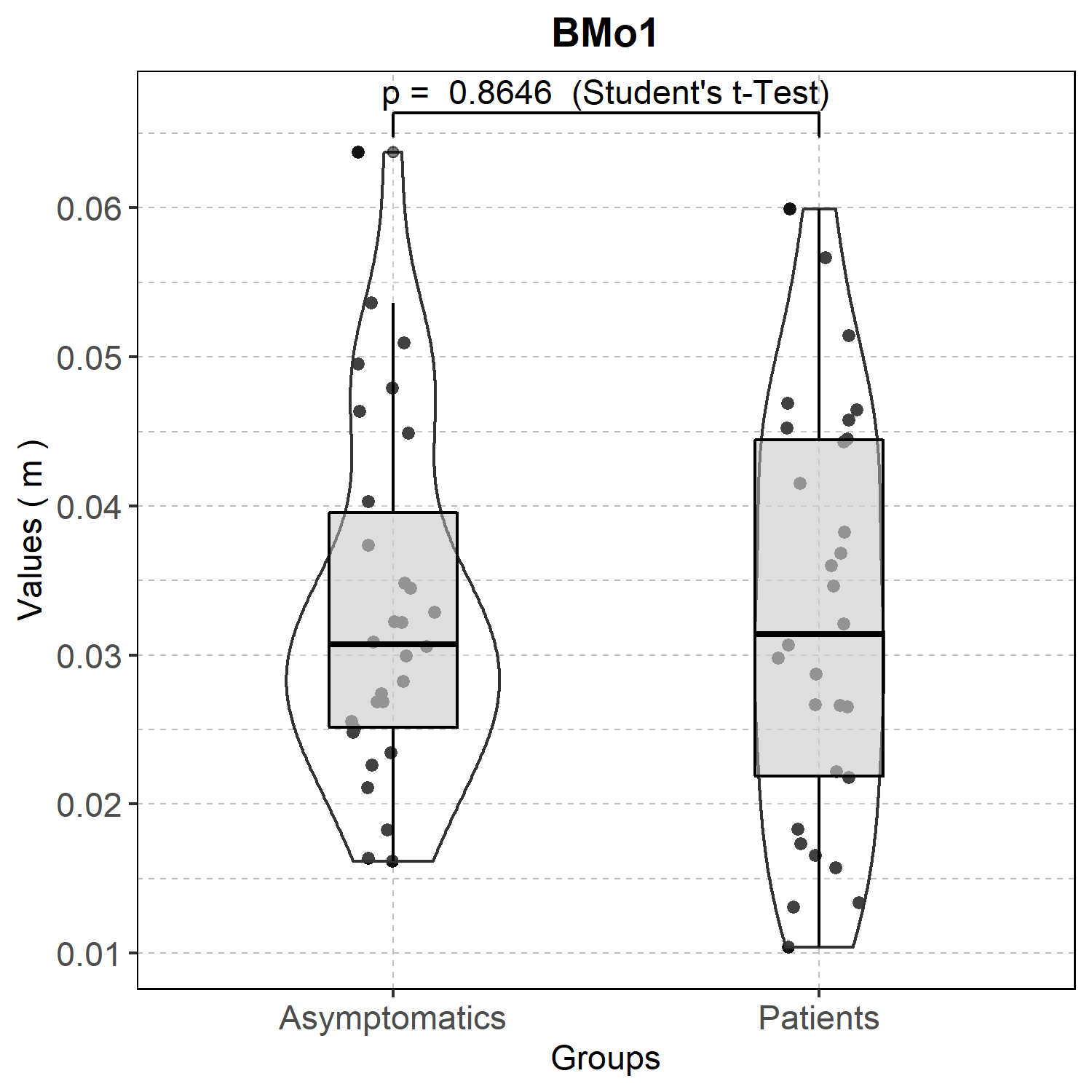

Supplement: Supplementary file 2 — Supplementary Information 2. [file 41598_2023_33504_MOESM2_ESM.zip › BMo001_boxplot.png]

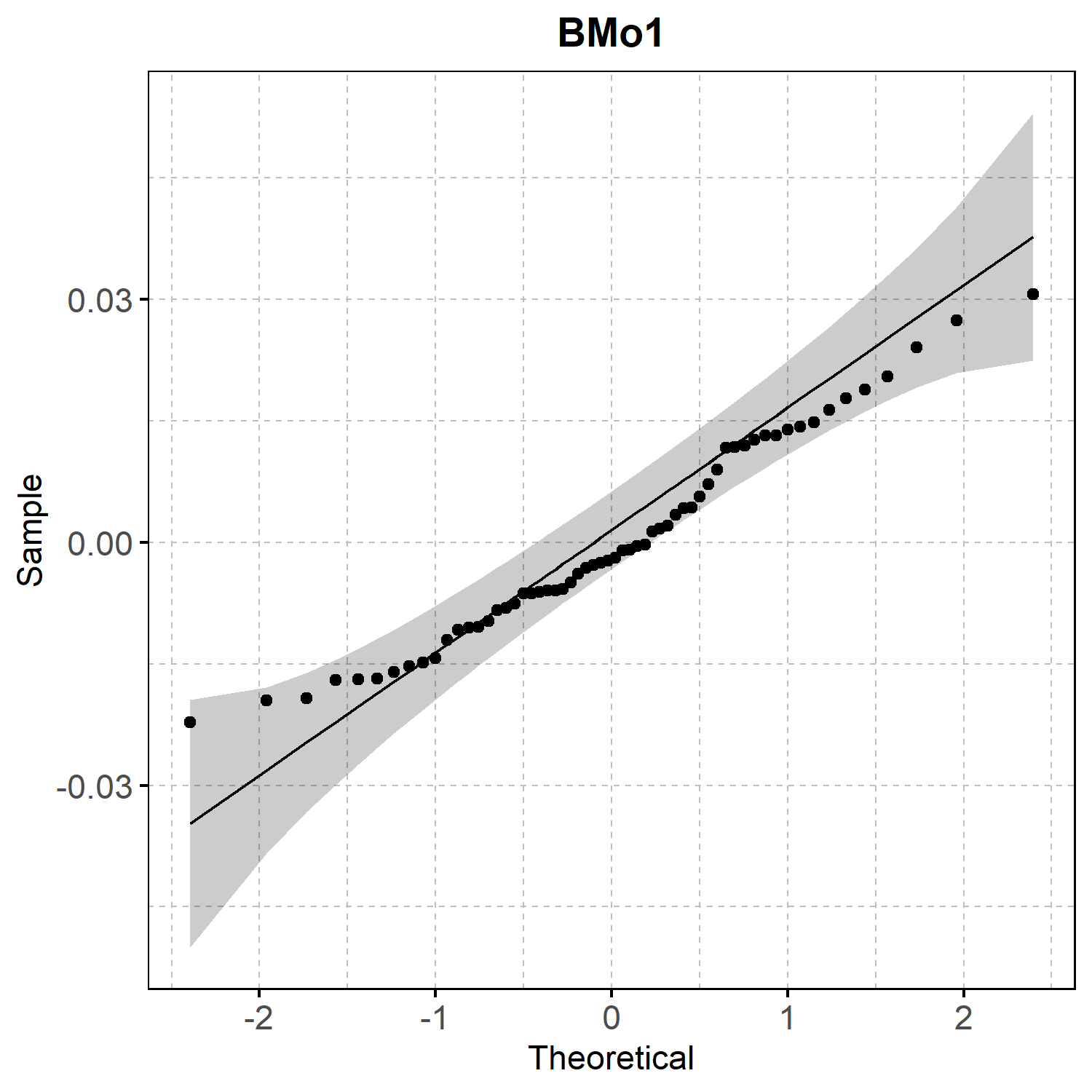

Supplement: Supplementary file 2 — Supplementary Information 2. [file 41598_2023_33504_MOESM2_ESM.zip › BMo001_normality.png]

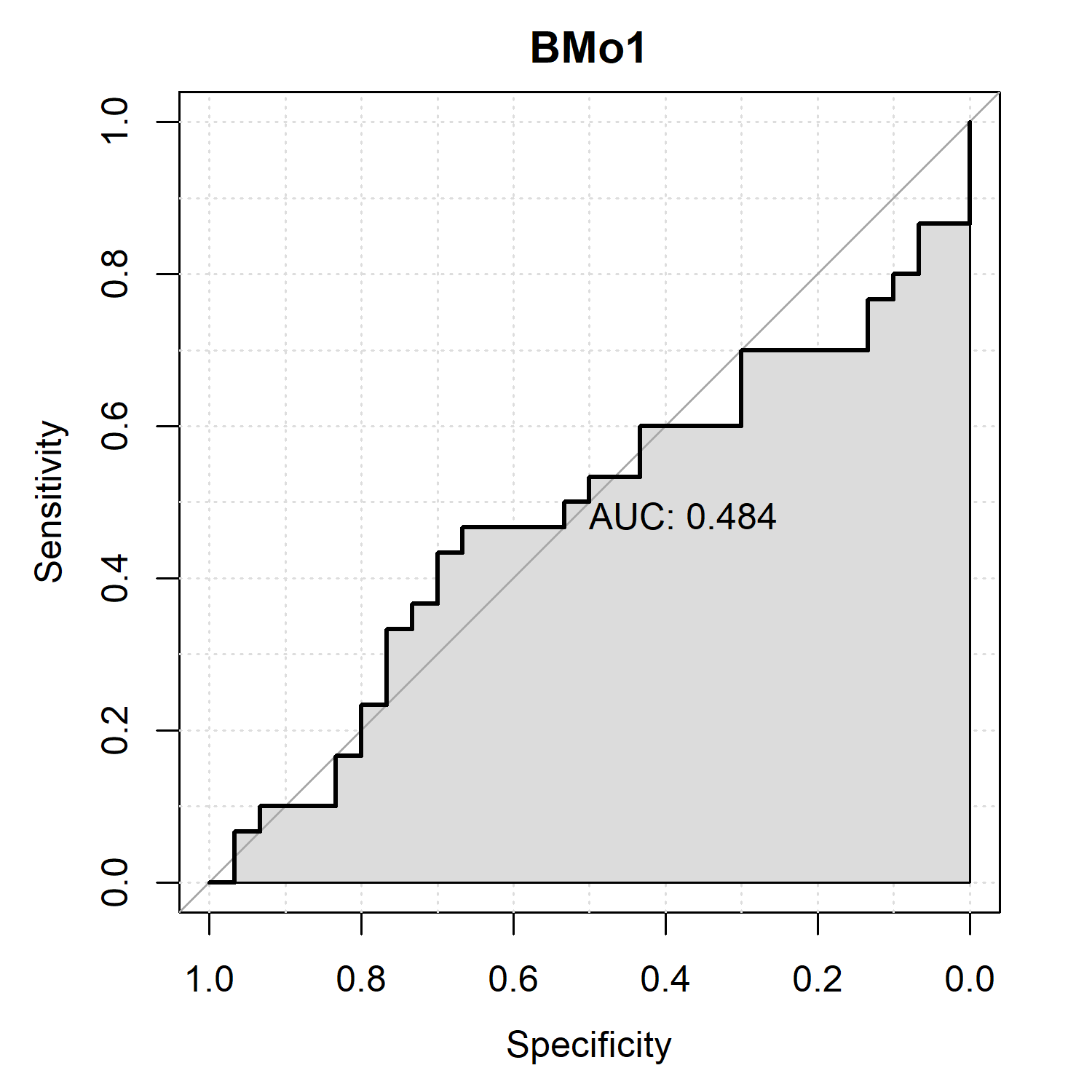

Supplement: Supplementary file 2 — Supplementary Information 2. [file 41598_2023_33504_MOESM2_ESM.zip › BMo001_ROC.png]

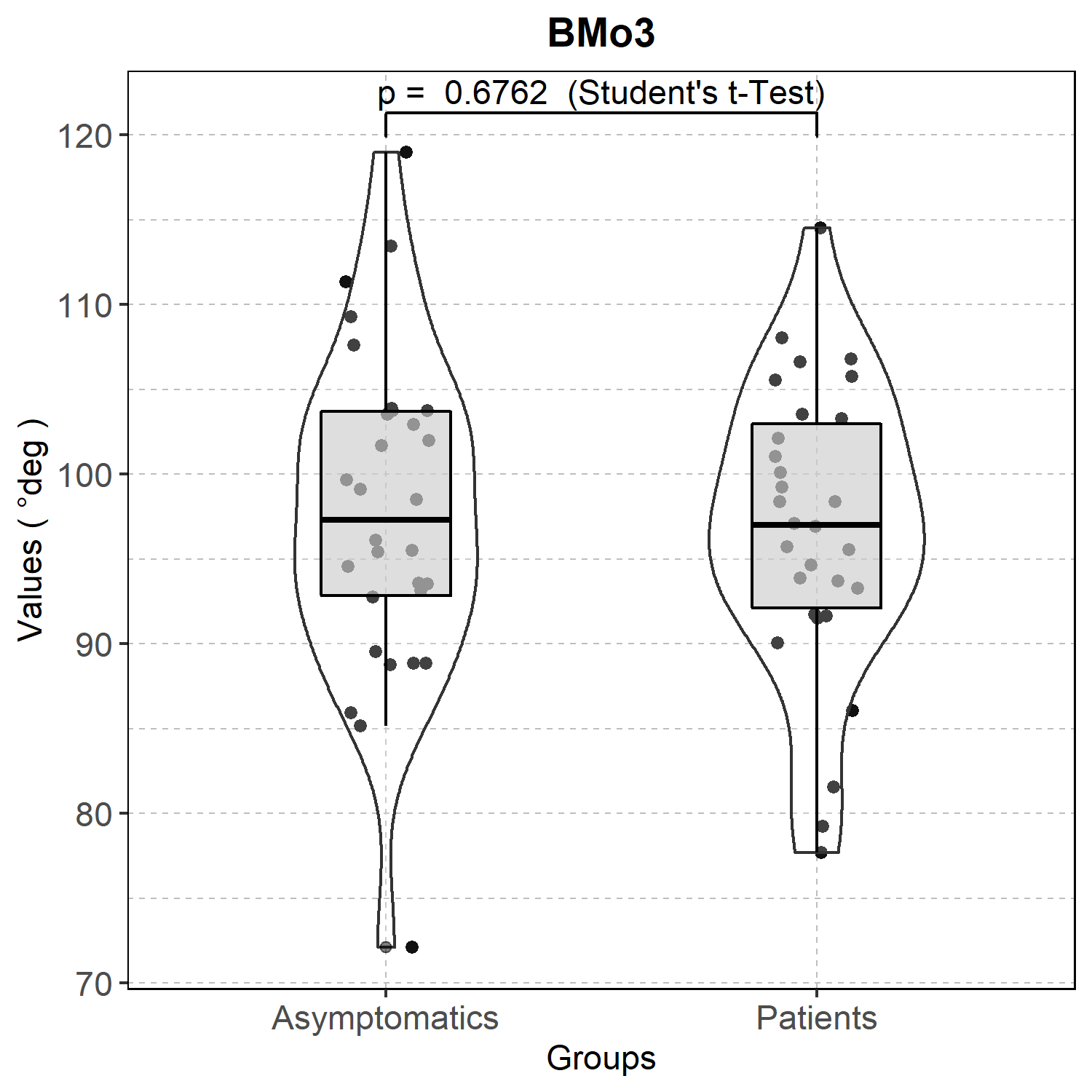

Supplement: Supplementary file 2 — Supplementary Information 2. [file 41598_2023_33504_MOESM2_ESM.zip › BMo003_boxplot.png]

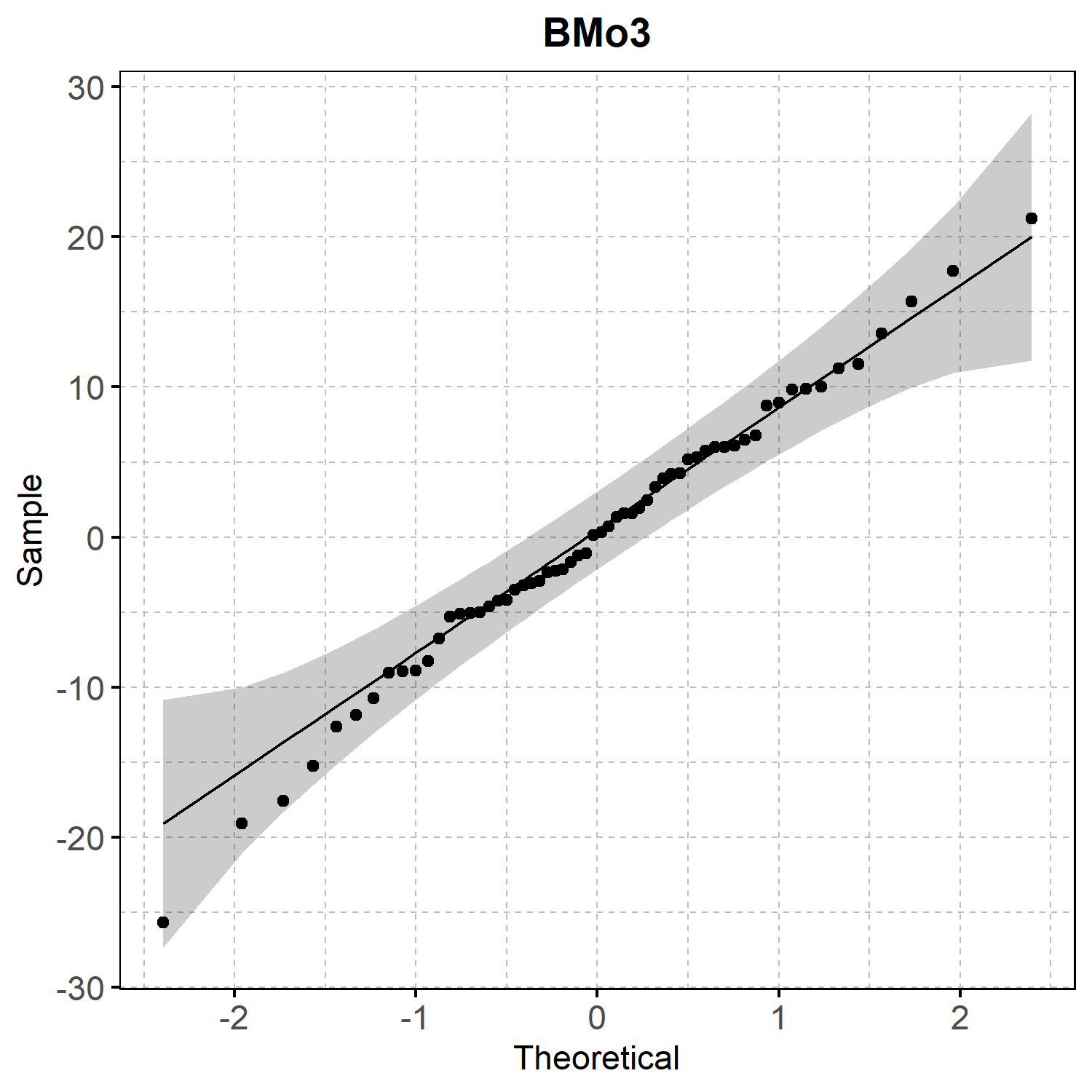

Supplement: Supplementary file 2 — Supplementary Information 2. [file 41598_2023_33504_MOESM2_ESM.zip › BMo003_normality.png]

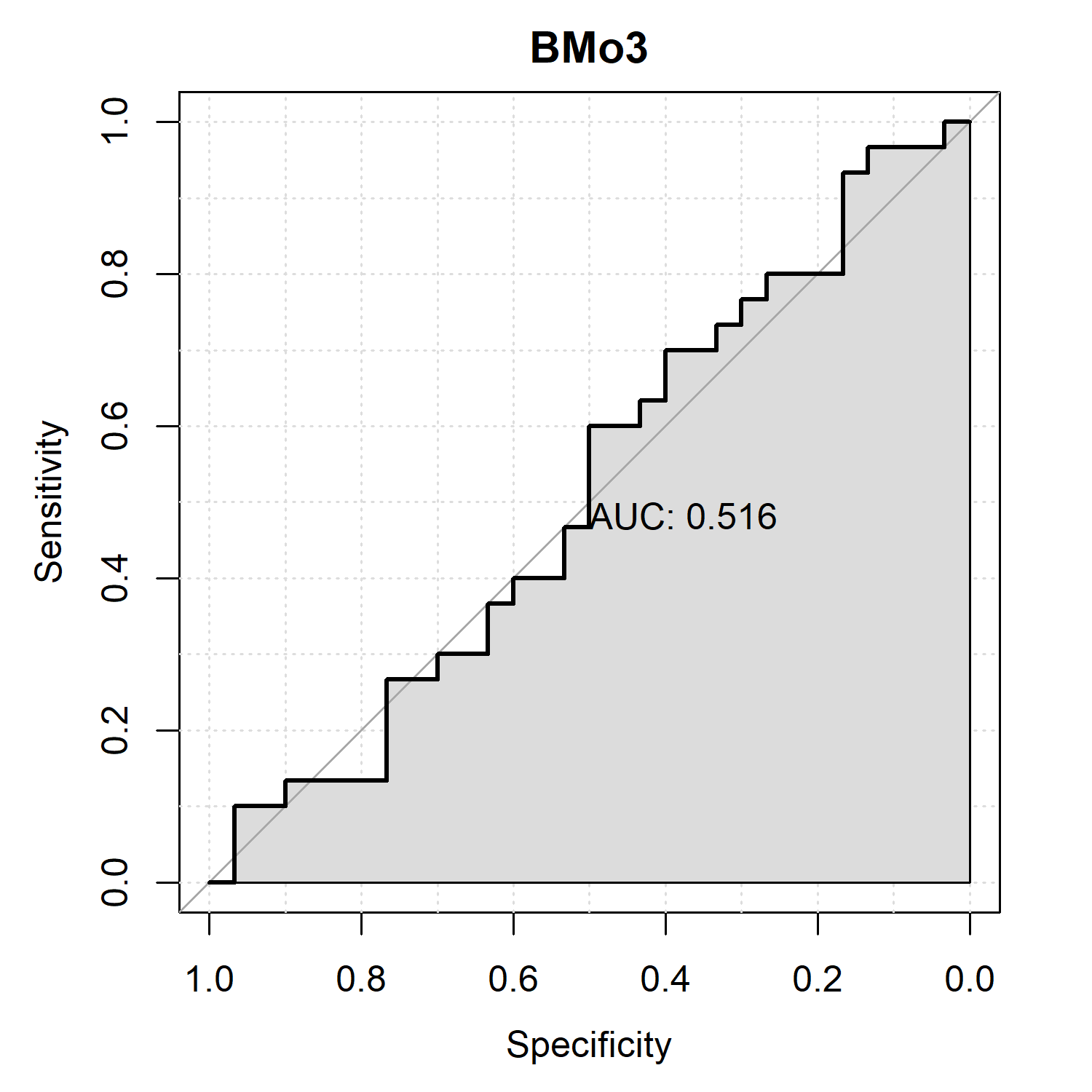

Supplement: Supplementary file 2 — Supplementary Information 2. [file 41598_2023_33504_MOESM2_ESM.zip › BMo003_ROC.png]

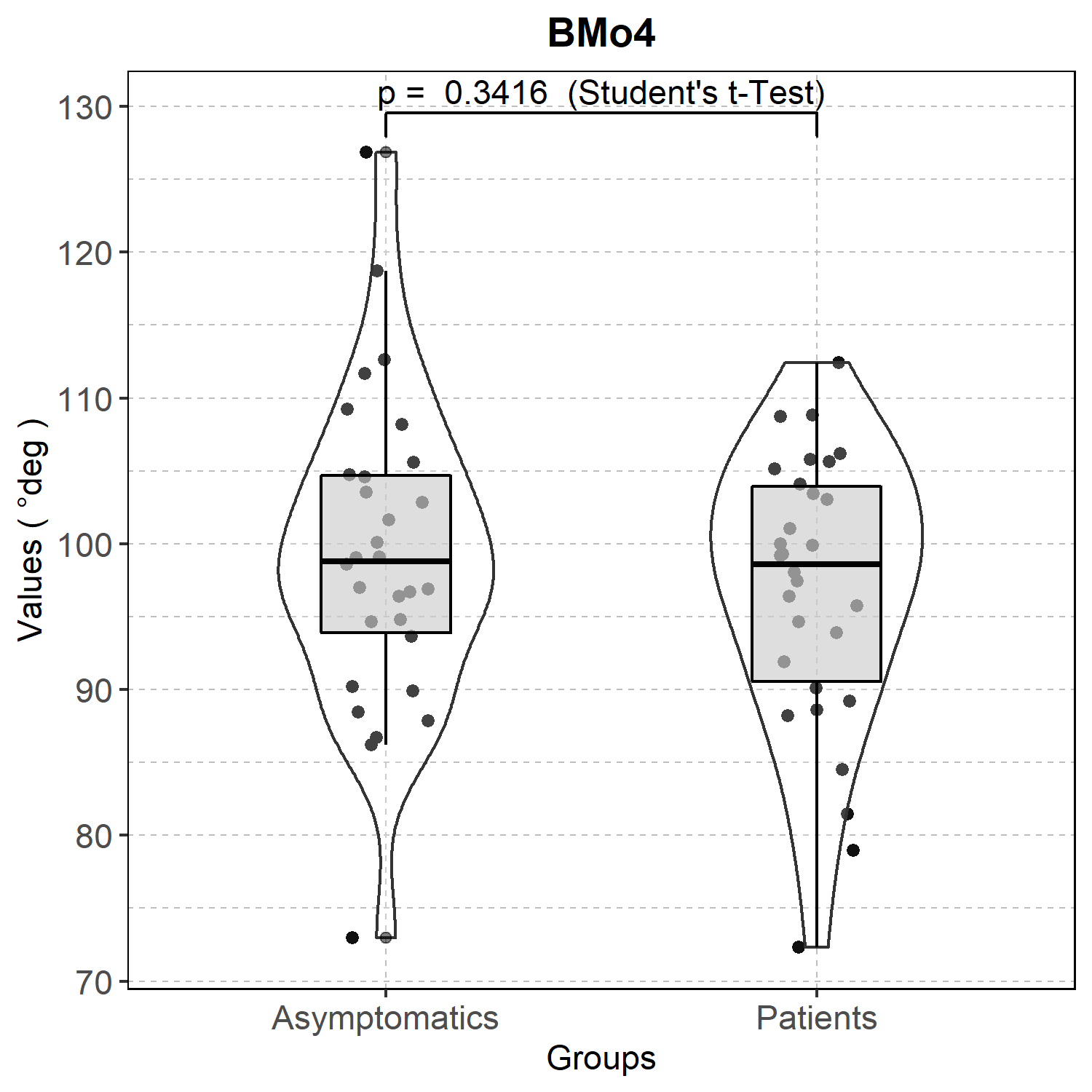

Supplement: Supplementary file 2 — Supplementary Information 2. [file 41598_2023_33504_MOESM2_ESM.zip › BMo004_boxplot.png]

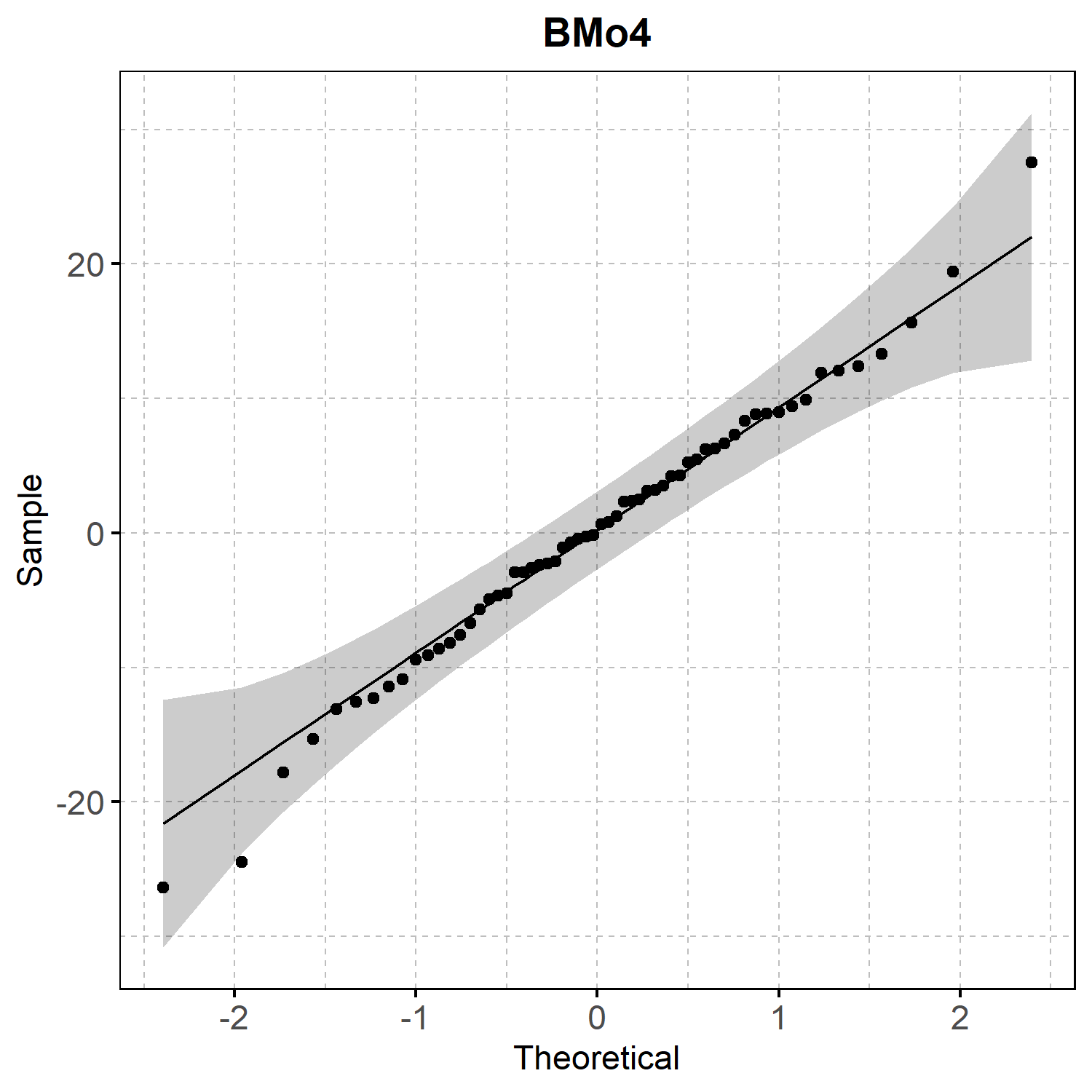

Supplement: Supplementary file 2 — Supplementary Information 2. [file 41598_2023_33504_MOESM2_ESM.zip › BMo004_normality.png]

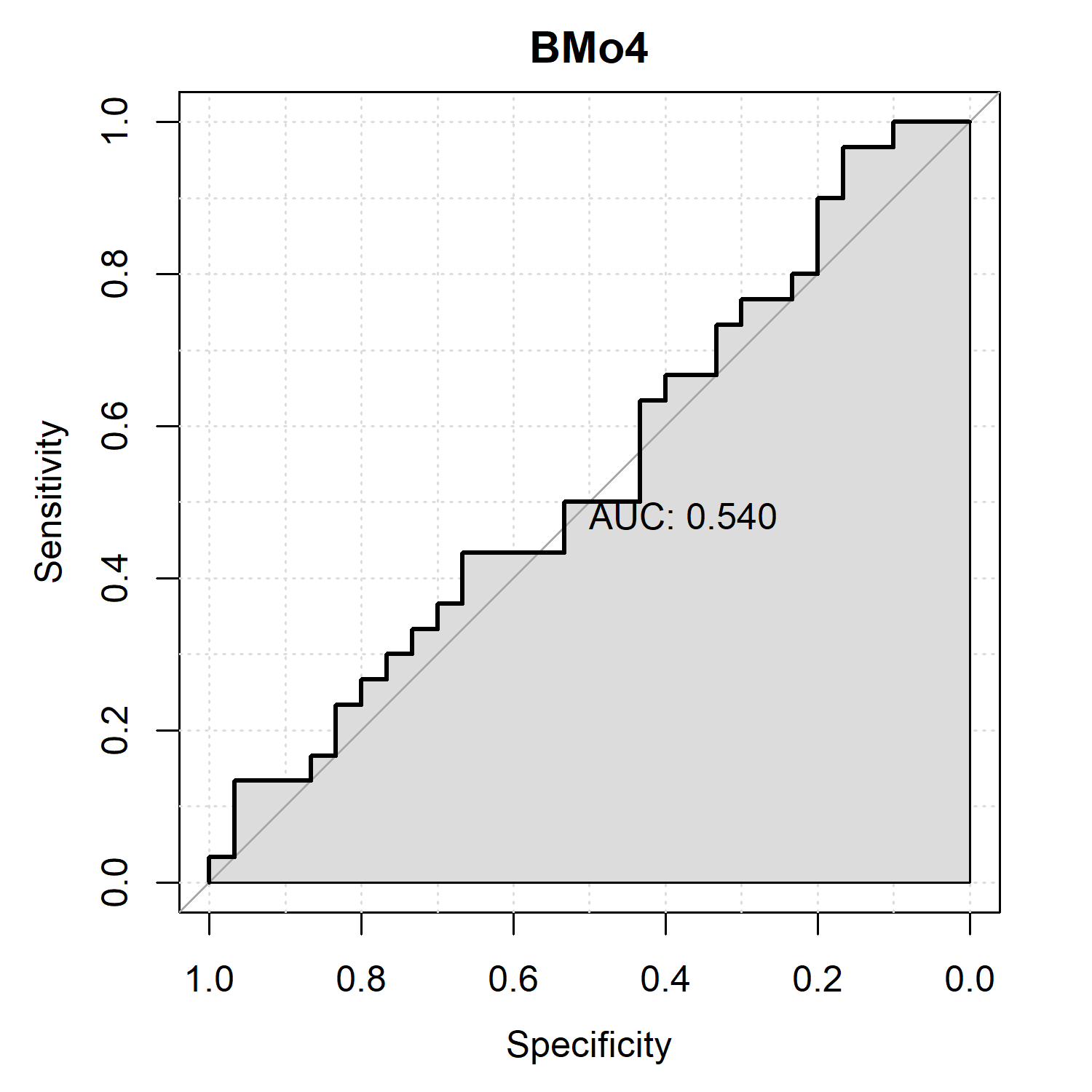

Supplement: Supplementary file 2 — Supplementary Information 2. [file 41598_2023_33504_MOESM2_ESM.zip › BMo004_ROC.png]

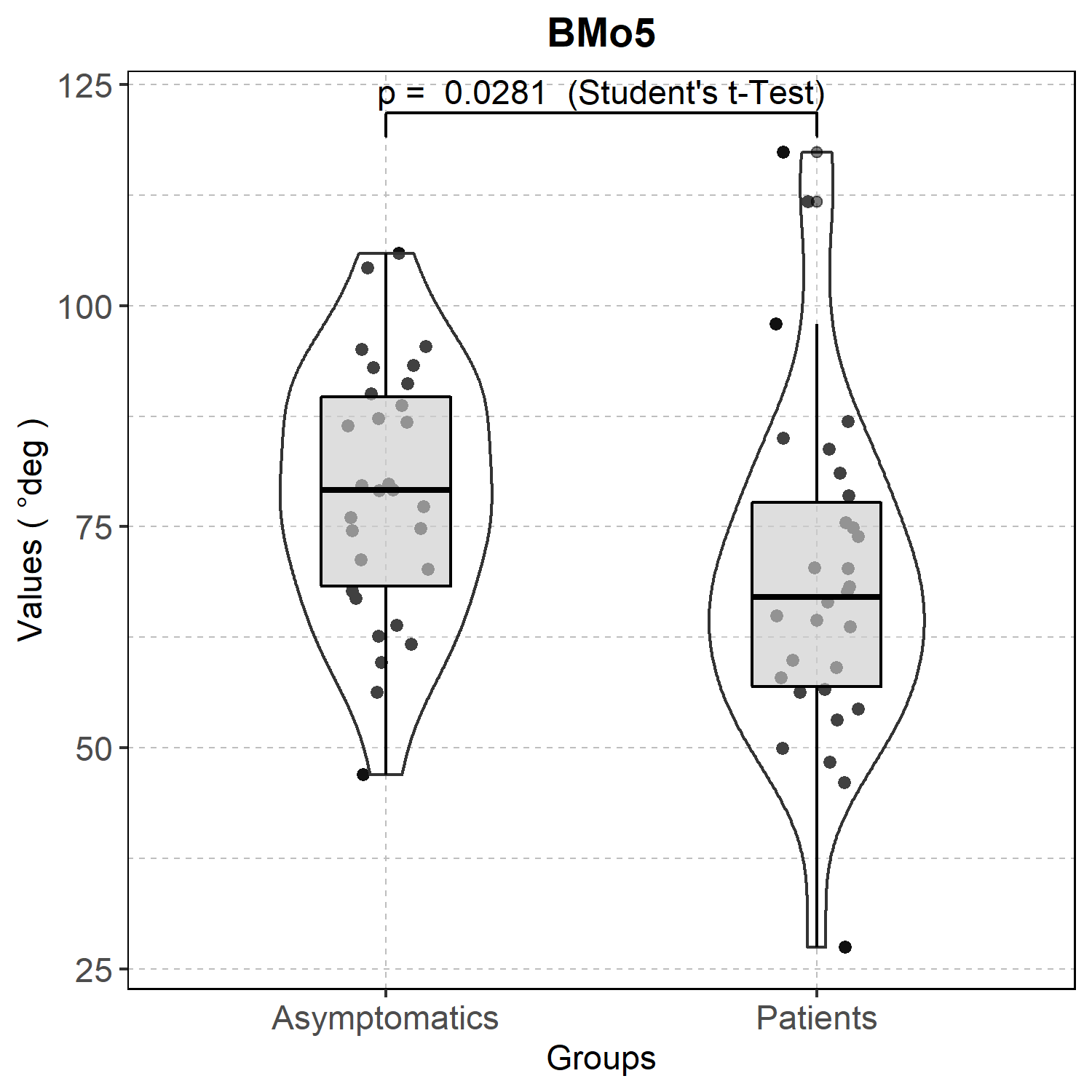

Supplement: Supplementary file 2 — Supplementary Information 2. [file 41598_2023_33504_MOESM2_ESM.zip › BMo005_boxplot.png]

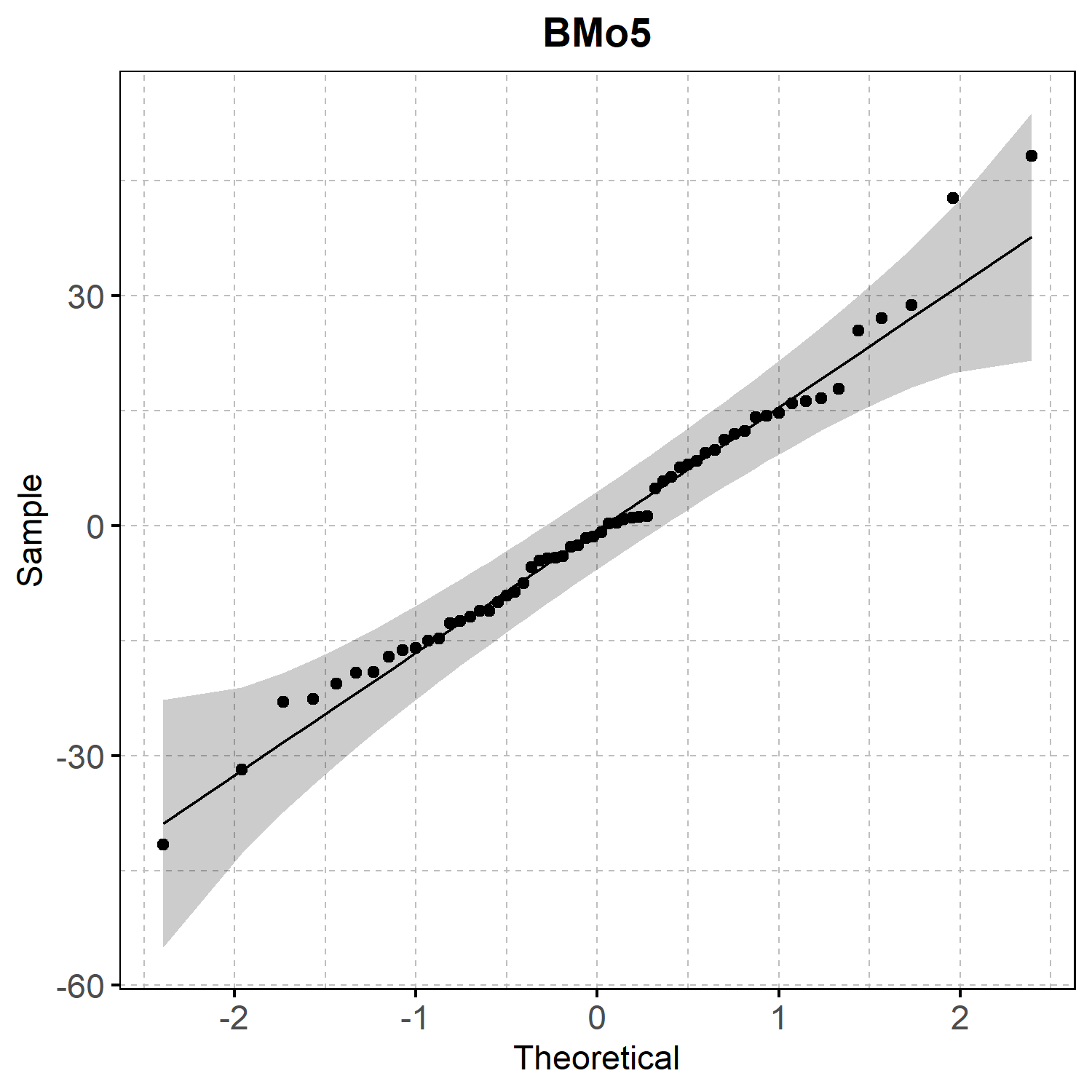

Supplement: Supplementary file 2 — Supplementary Information 2. [file 41598_2023_33504_MOESM2_ESM.zip › BMo005_normality.png]

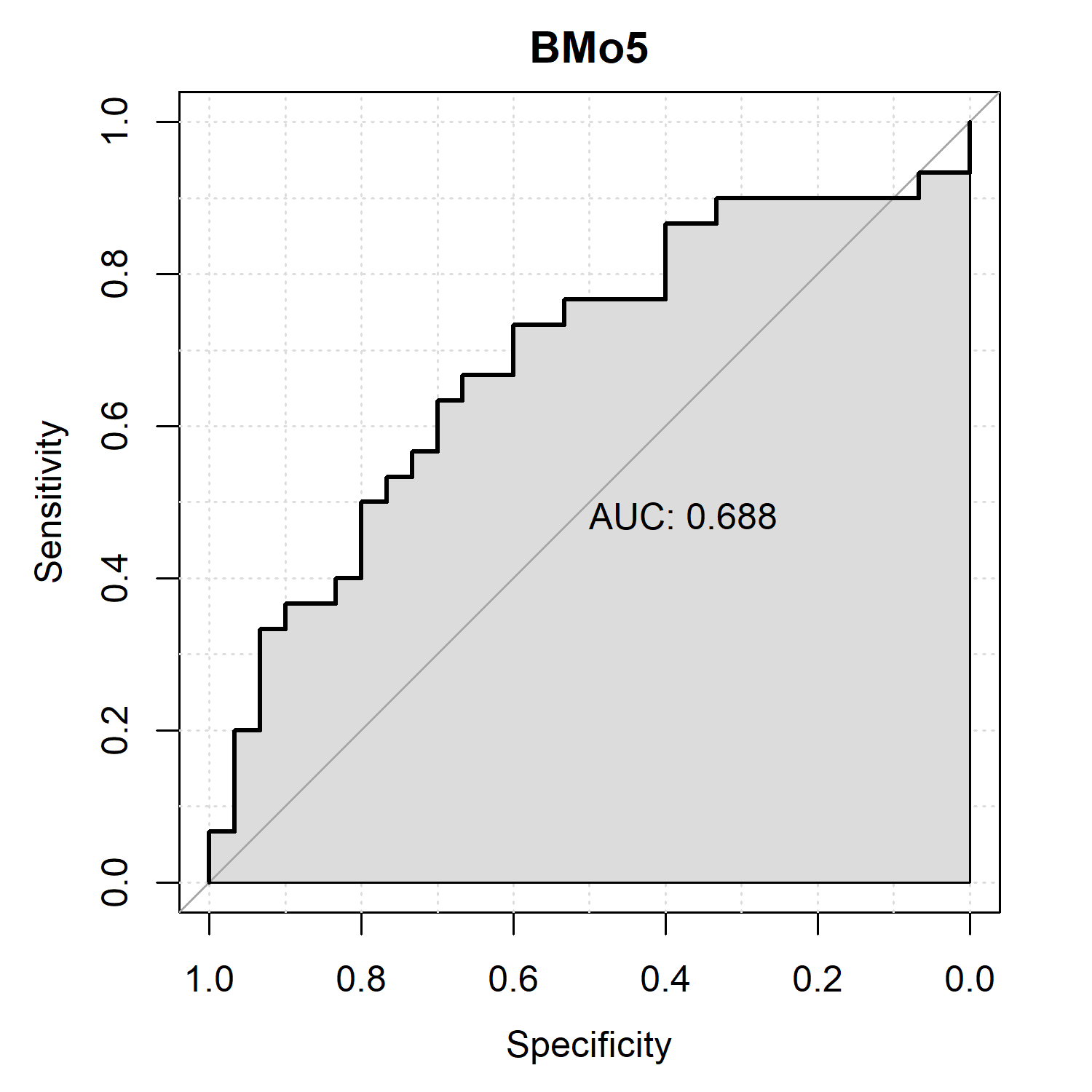

Supplement: Supplementary file 2 — Supplementary Information 2. [file 41598_2023_33504_MOESM2_ESM.zip › BMo005_ROC.png]

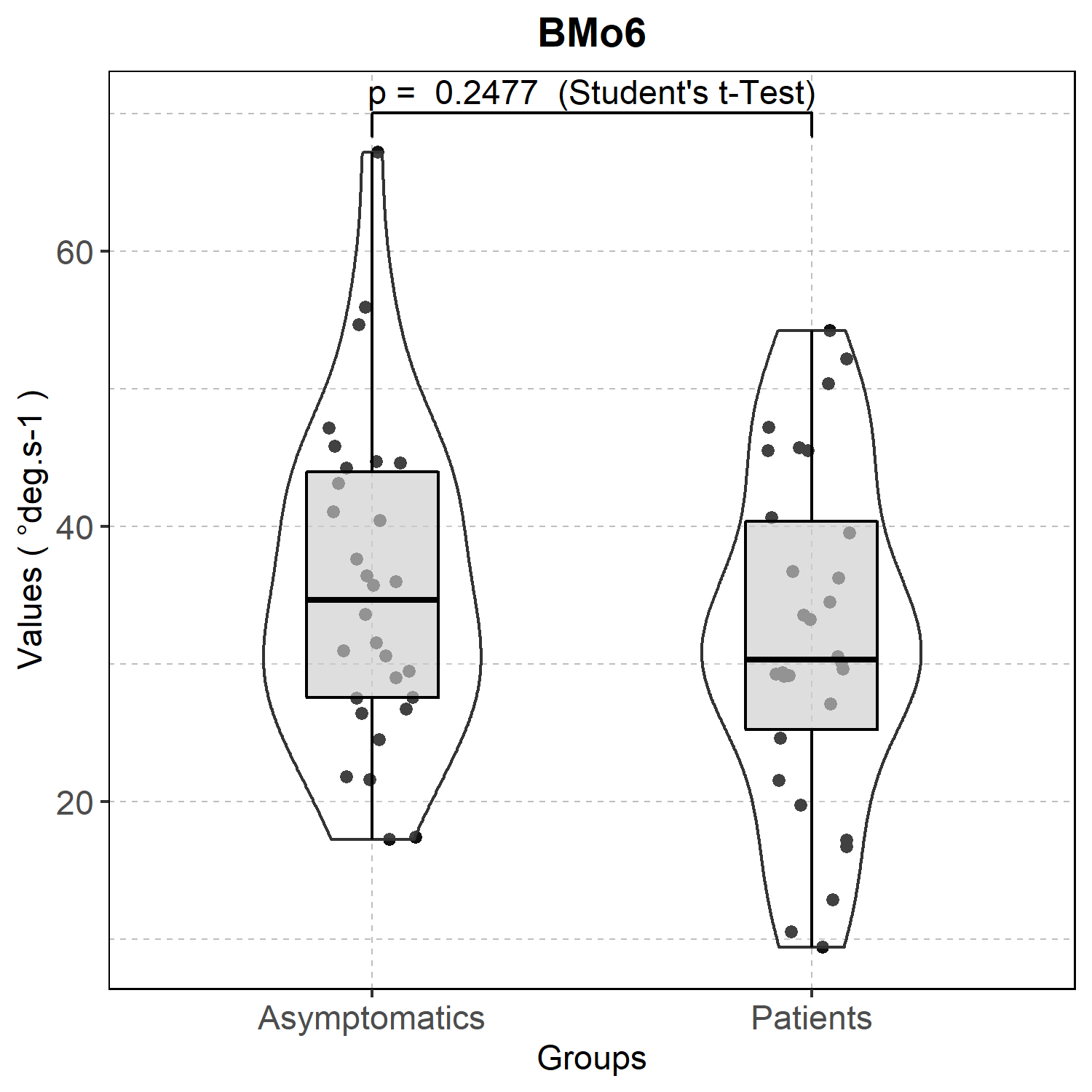

Supplement: Supplementary file 2 — Supplementary Information 2. [file 41598_2023_33504_MOESM2_ESM.zip › BMo006_boxplot.png]

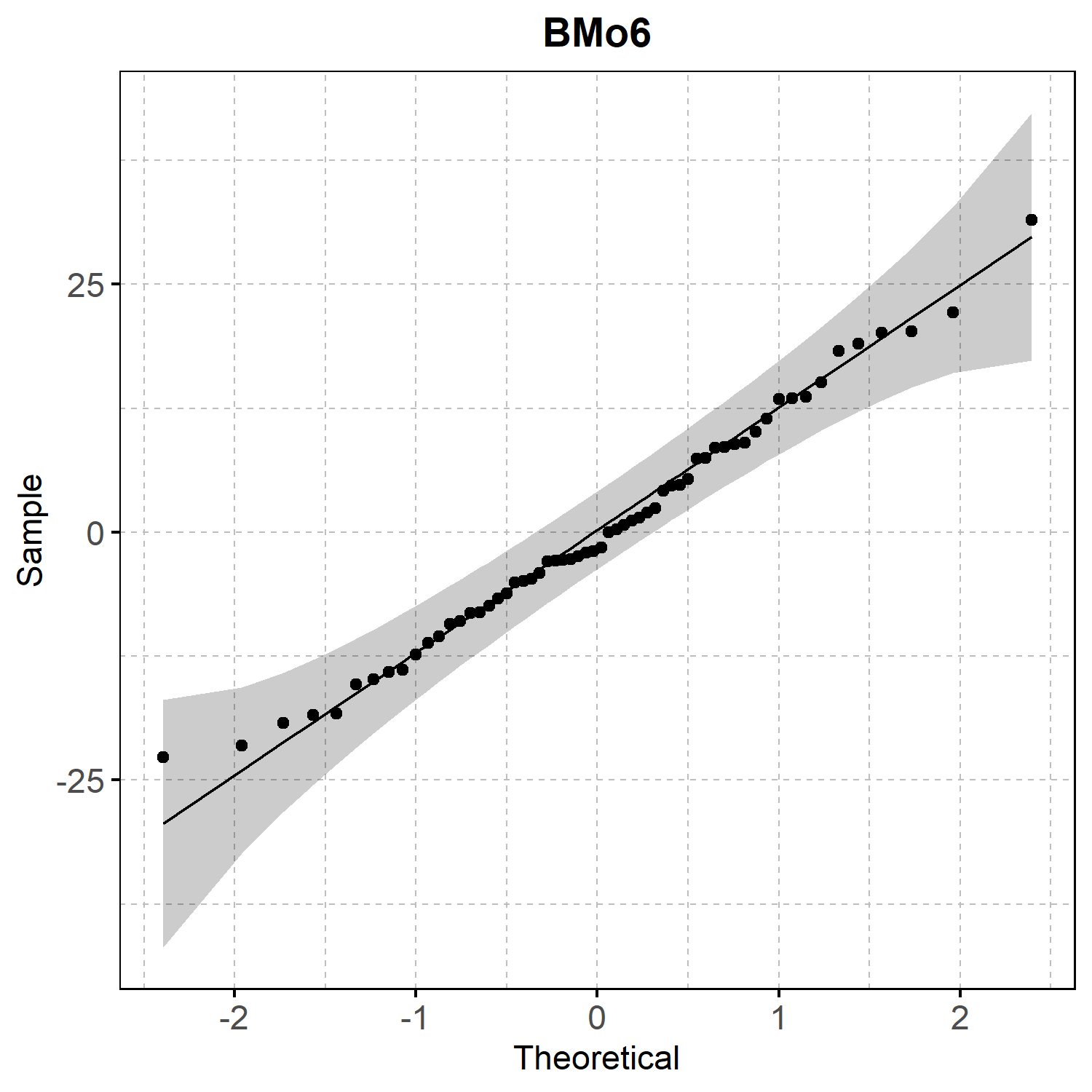

Supplement: Supplementary file 2 — Supplementary Information 2. [file 41598_2023_33504_MOESM2_ESM.zip › BMo006_normality.png]

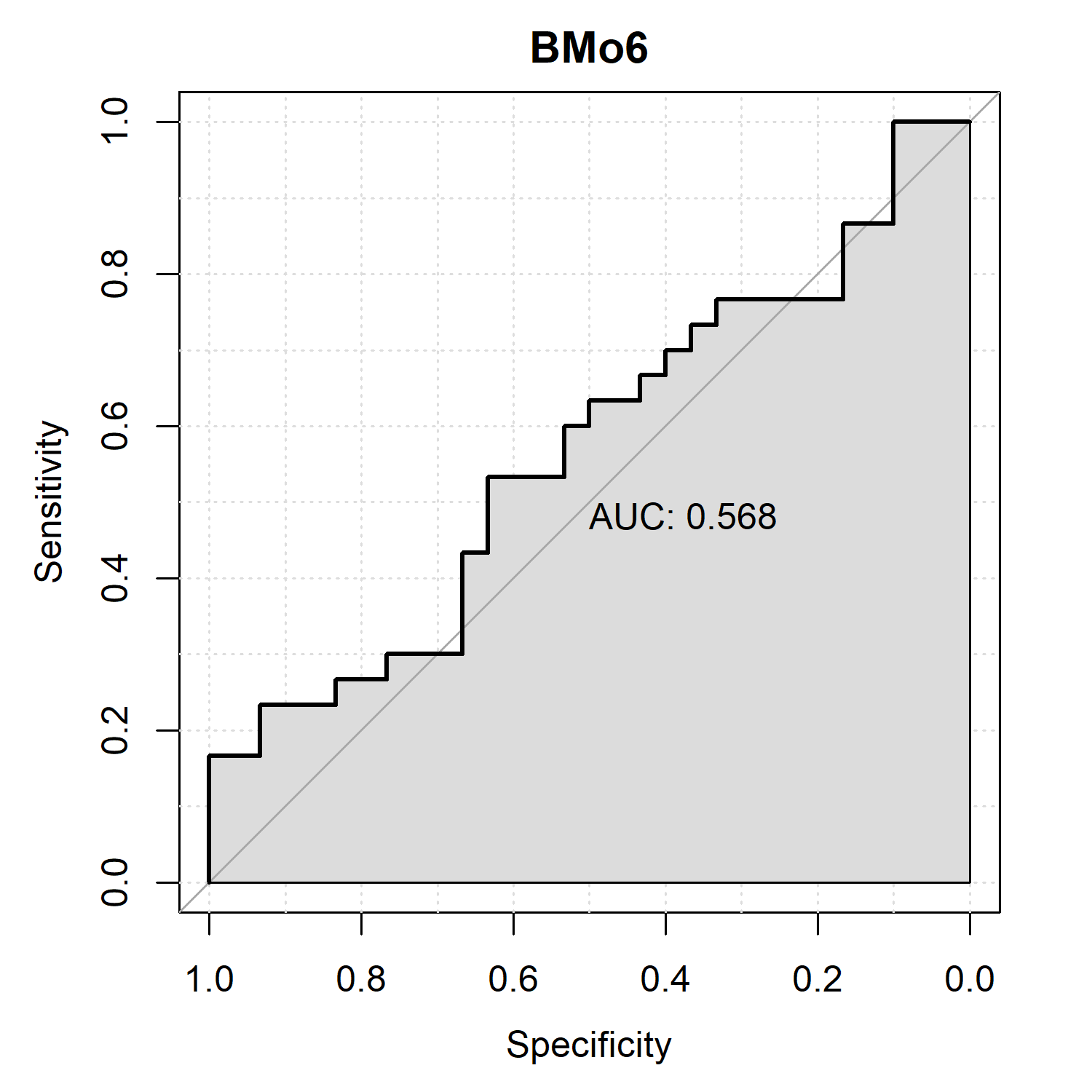

Supplement: Supplementary file 2 — Supplementary Information 2. [file 41598_2023_33504_MOESM2_ESM.zip › BMo006_ROC.png]

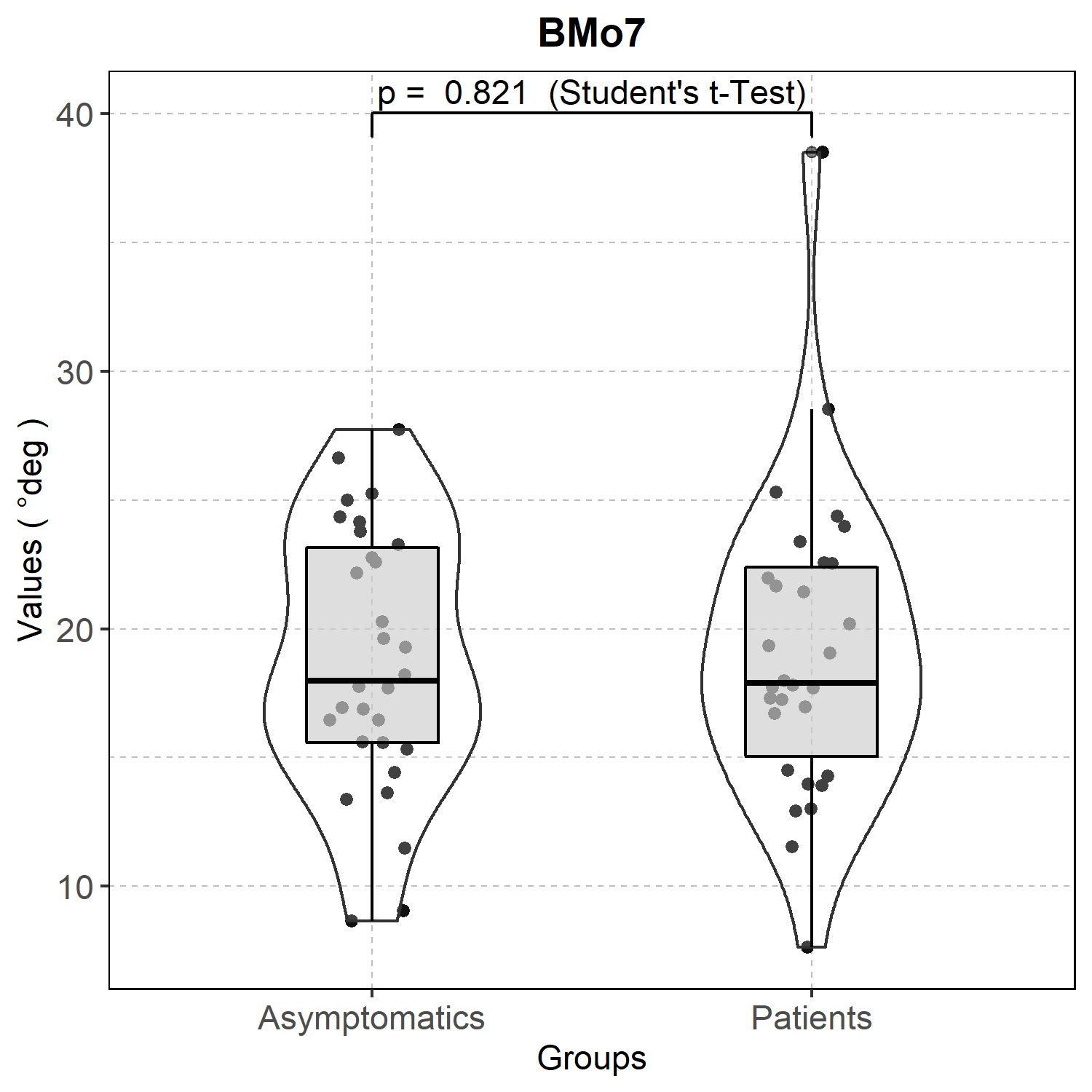

Supplement: Supplementary file 2 — Supplementary Information 2. [file 41598_2023_33504_MOESM2_ESM.zip › BMo007_boxplot.png]

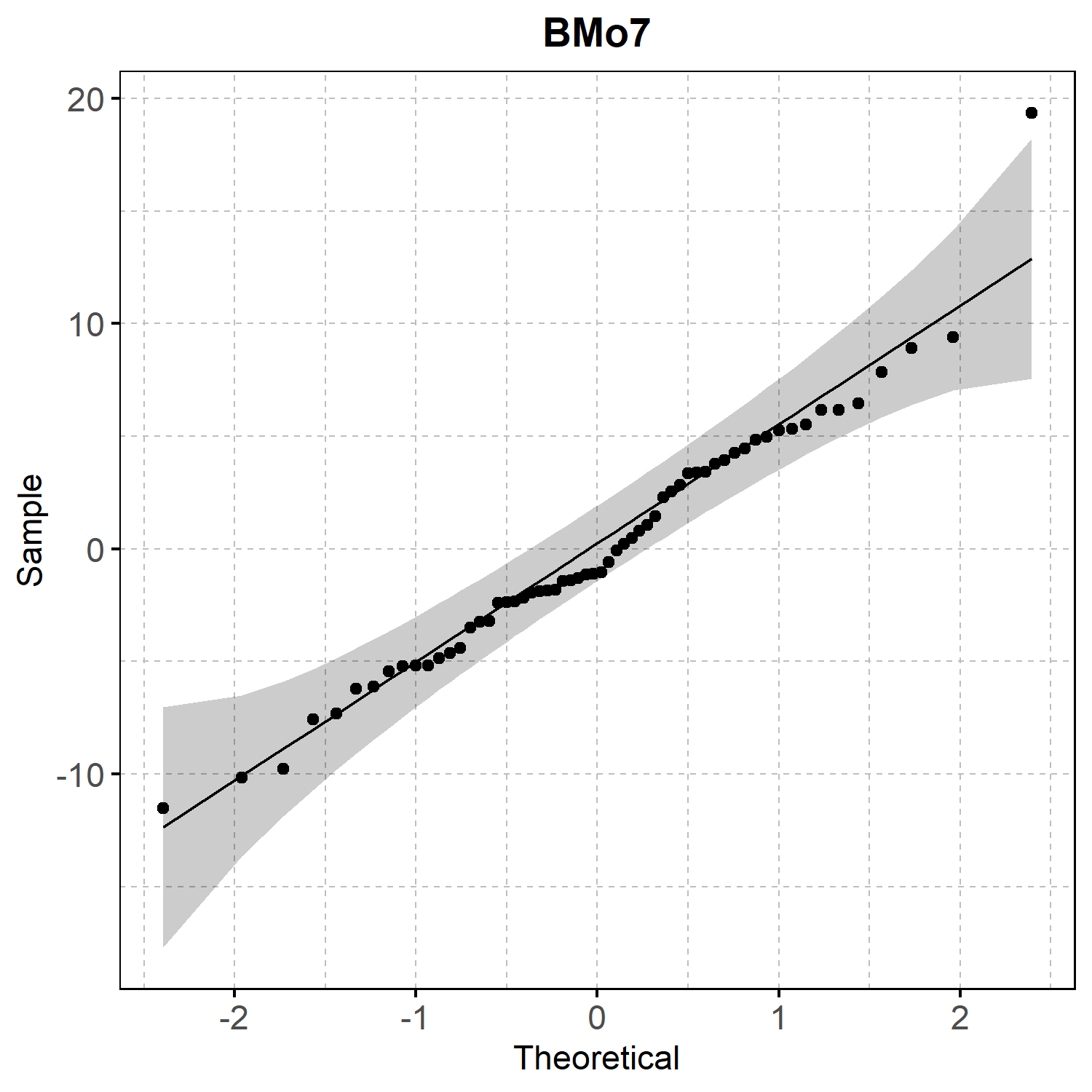

Supplement: Supplementary file 2 — Supplementary Information 2. [file 41598_2023_33504_MOESM2_ESM.zip › BMo007_normality.png]

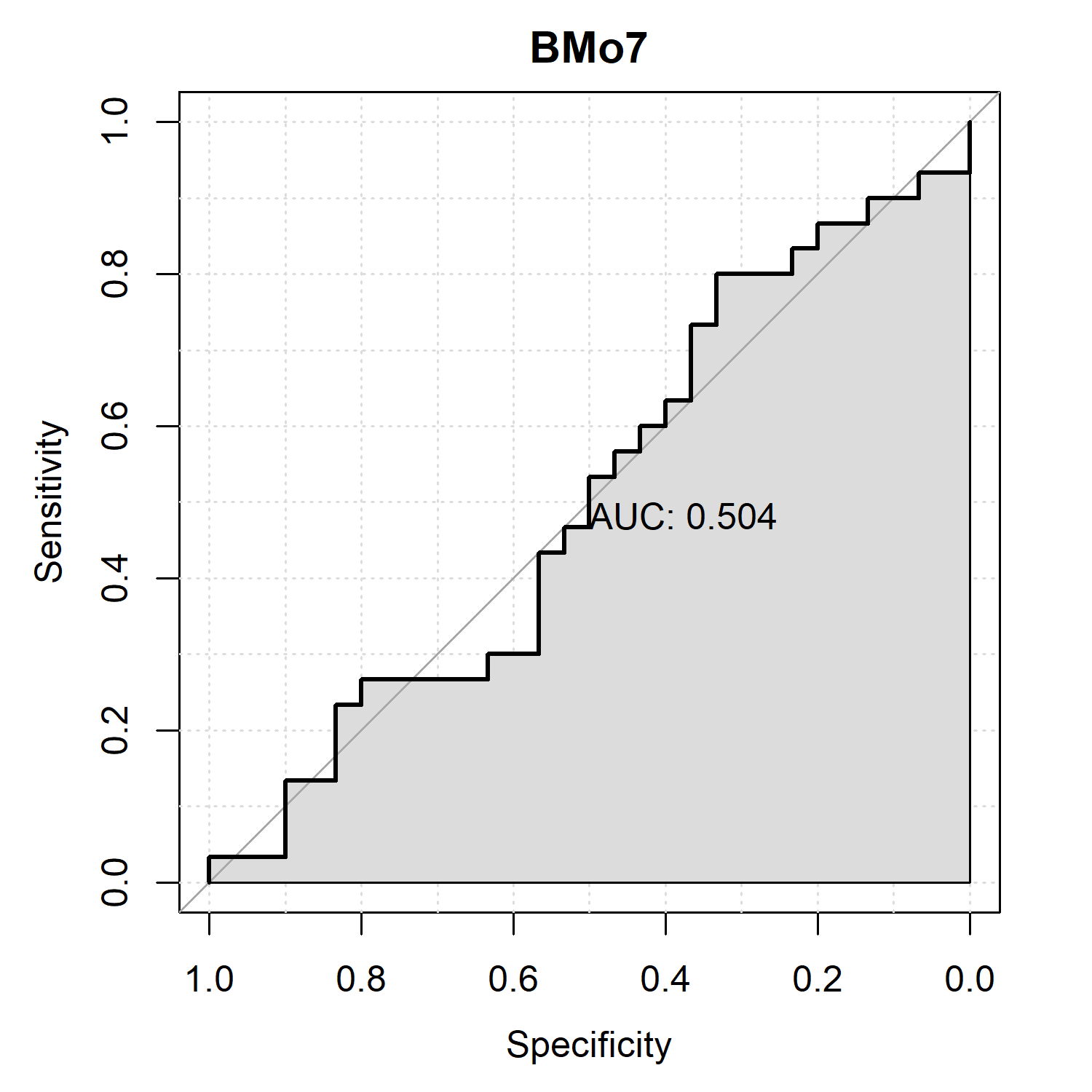

Supplement: Supplementary file 2 — Supplementary Information 2. [file 41598_2023_33504_MOESM2_ESM.zip › BMo007_ROC.png]

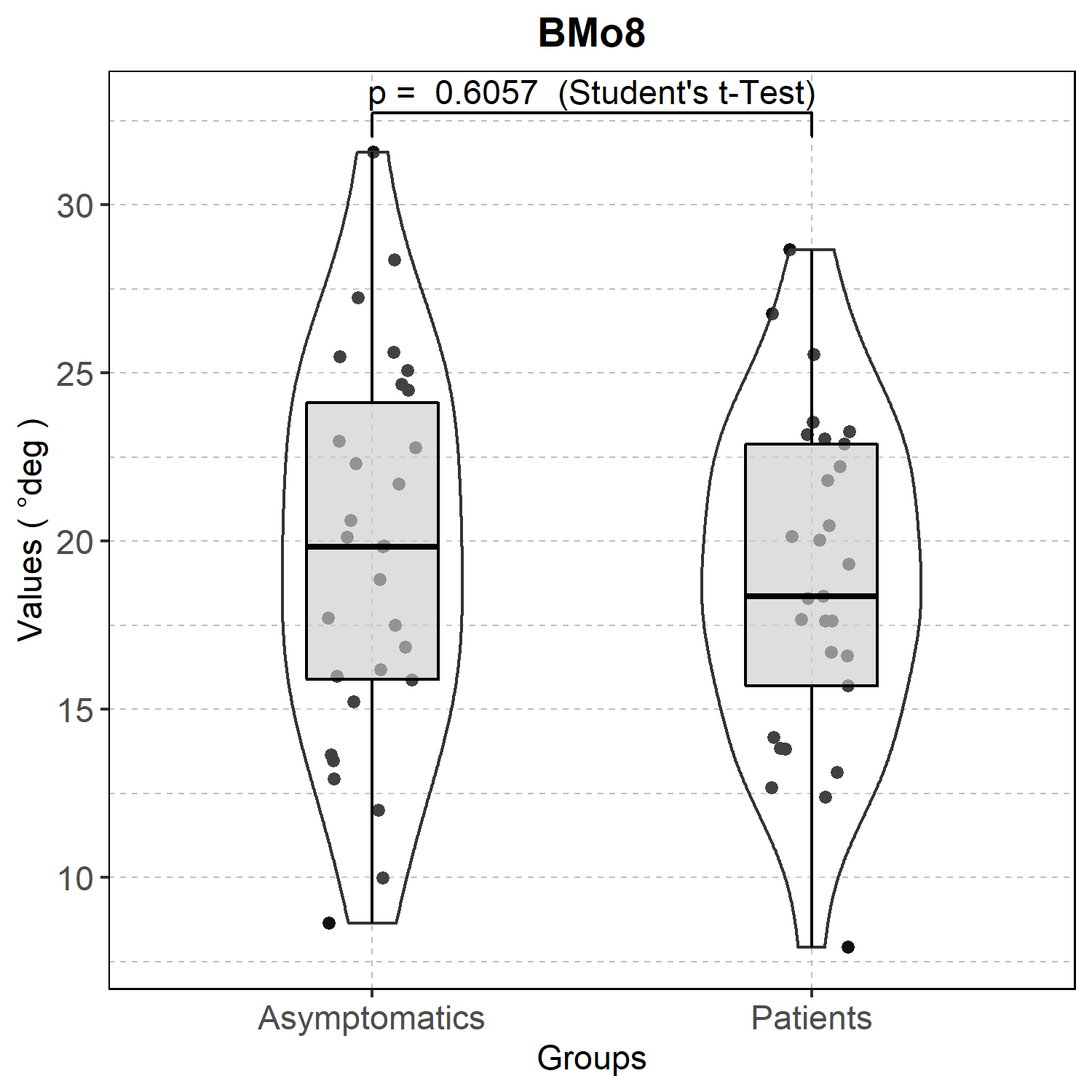

Supplement: Supplementary file 2 — Supplementary Information 2. [file 41598_2023_33504_MOESM2_ESM.zip › BMo008_boxplot.png]

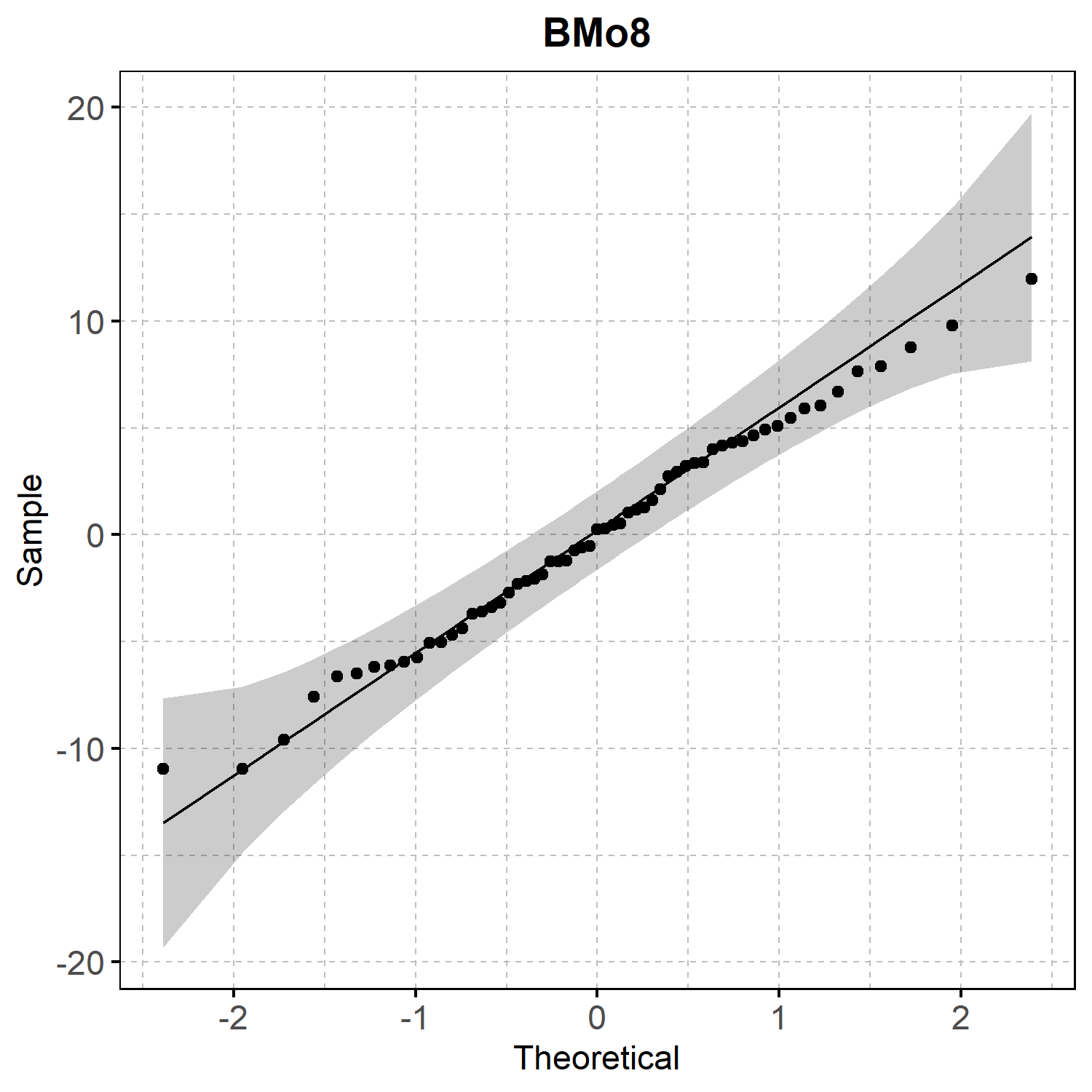

Supplement: Supplementary file 2 — Supplementary Information 2. [file 41598_2023_33504_MOESM2_ESM.zip › BMo008_normality.png]

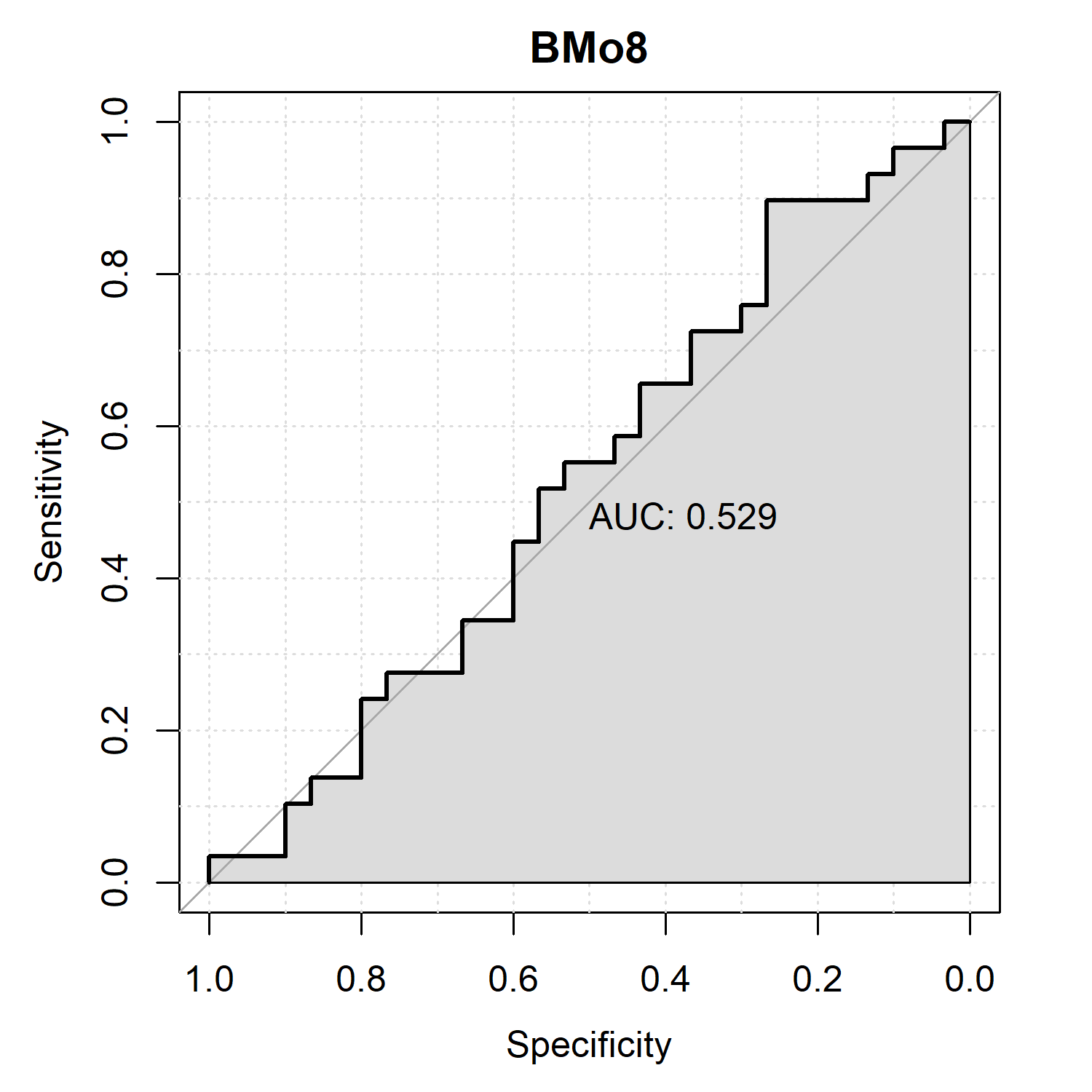

Supplement: Supplementary file 2 — Supplementary Information 2. [file 41598_2023_33504_MOESM2_ESM.zip › BMo008_ROC.png]

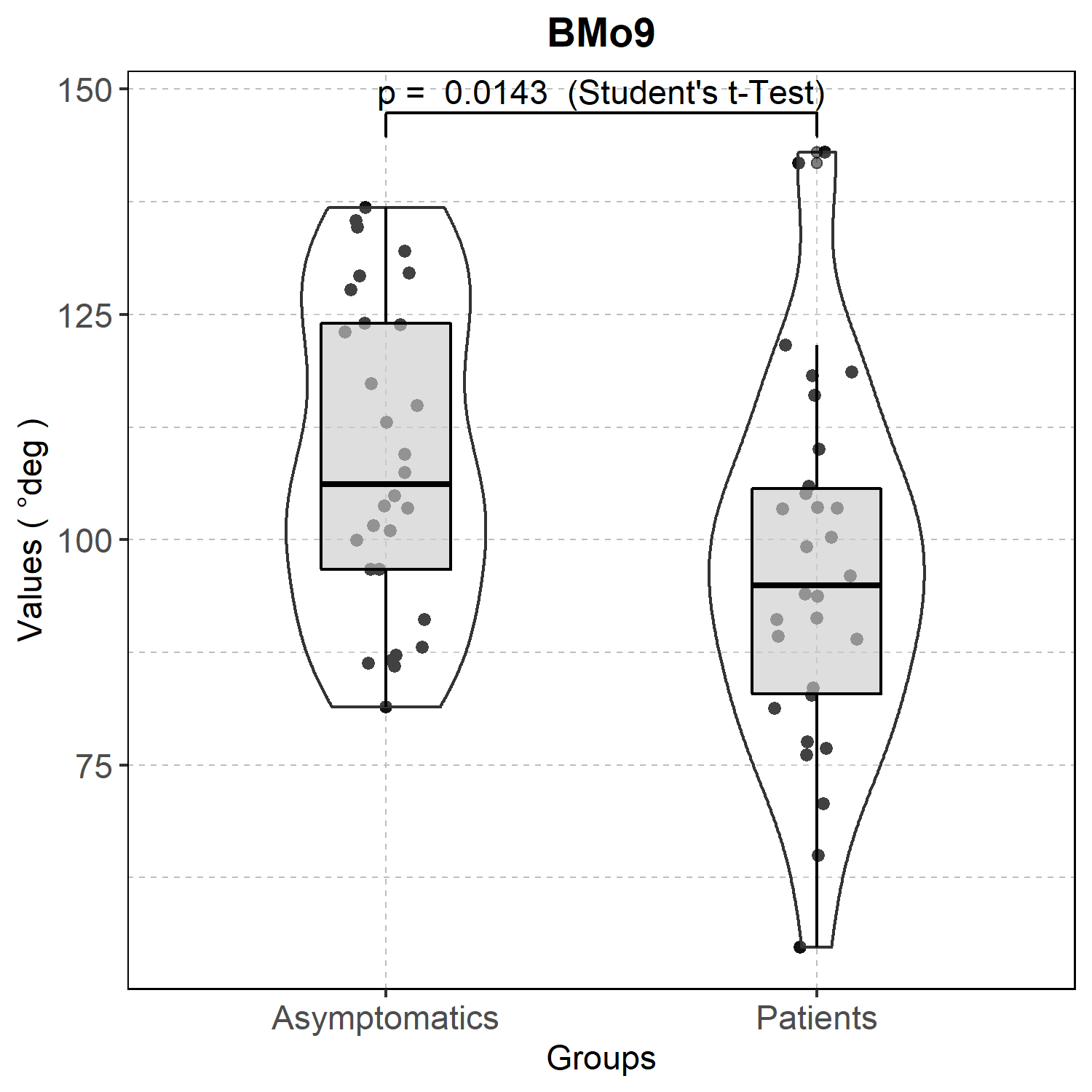

Supplement: Supplementary file 2 — Supplementary Information 2. [file 41598_2023_33504_MOESM2_ESM.zip › BMo009_boxplot.png]

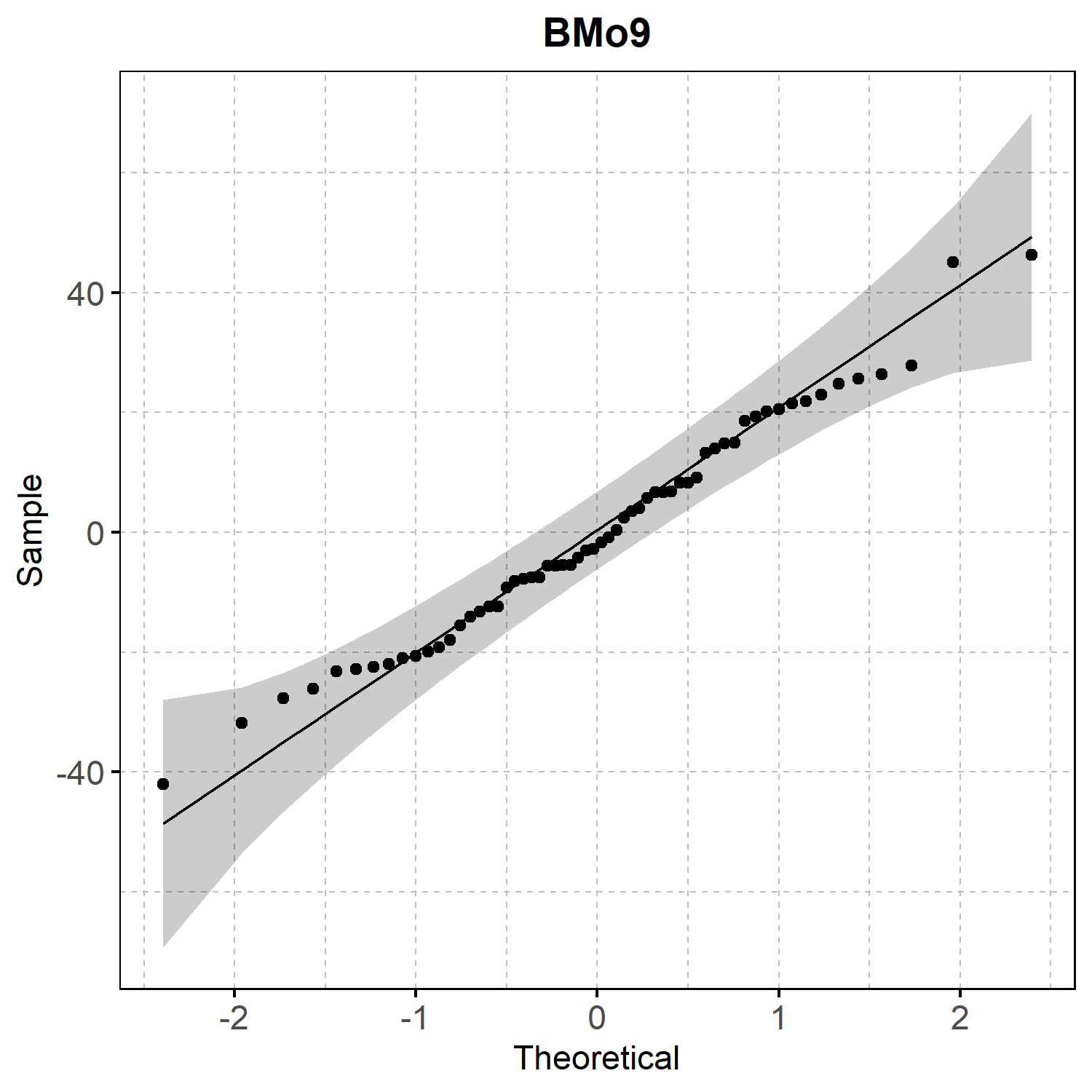

Supplement: Supplementary file 2 — Supplementary Information 2. [file 41598_2023_33504_MOESM2_ESM.zip › BMo009_normality.png]

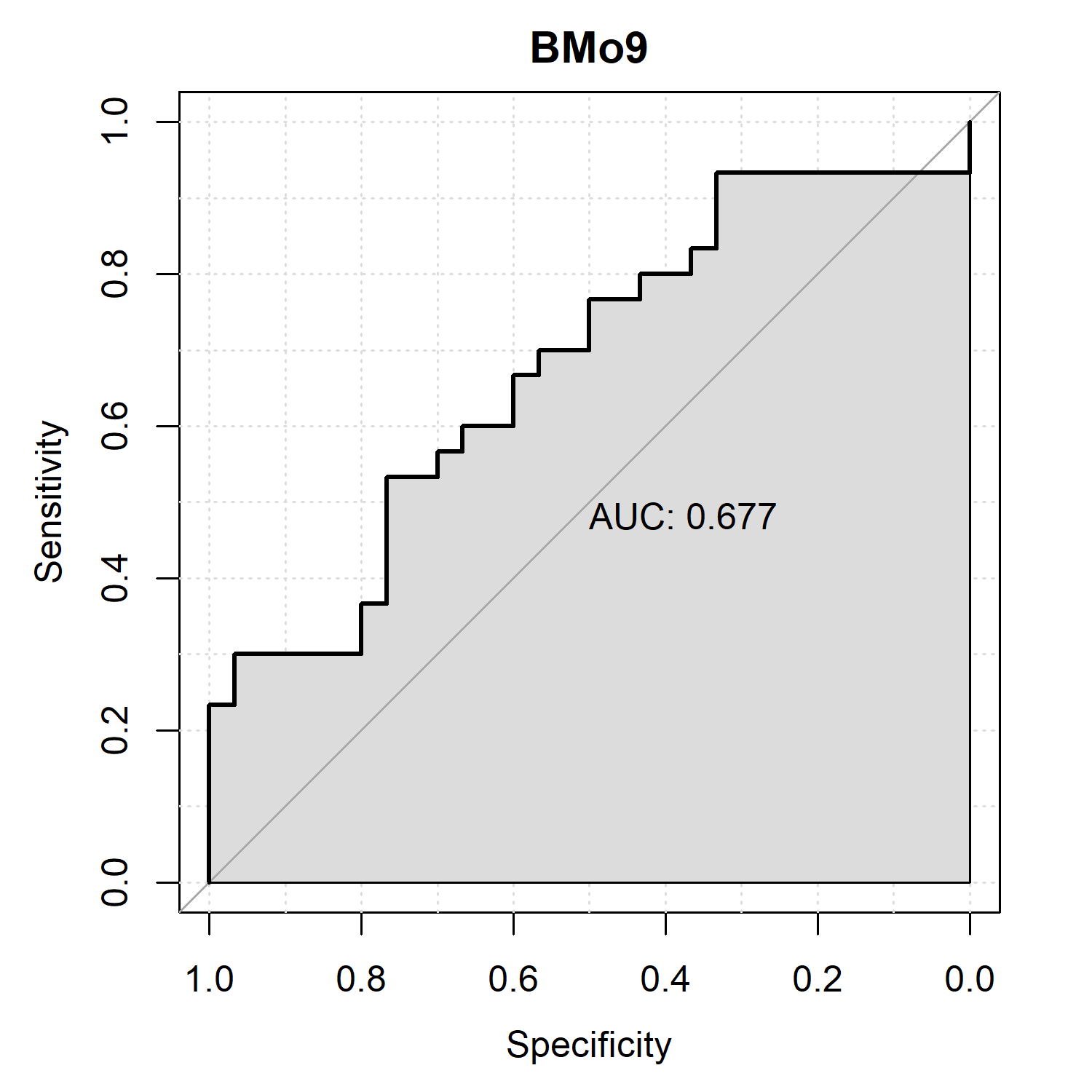

Supplement: Supplementary file 2 — Supplementary Information 2. [file 41598_2023_33504_MOESM2_ESM.zip › BMo009_ROC.png]

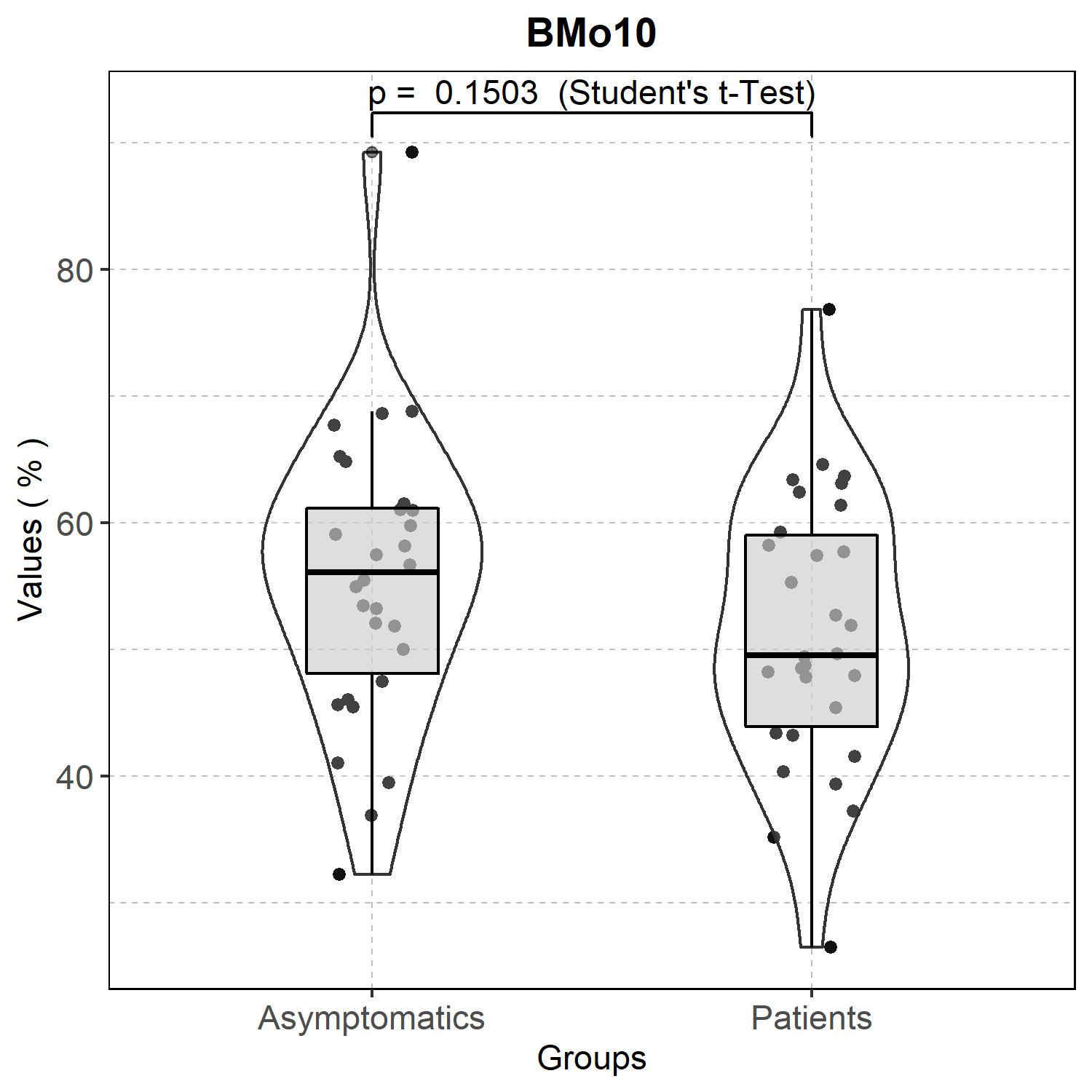

Supplement: Supplementary file 2 — Supplementary Information 2. [file 41598_2023_33504_MOESM2_ESM.zip › BMo010_boxplot.png]

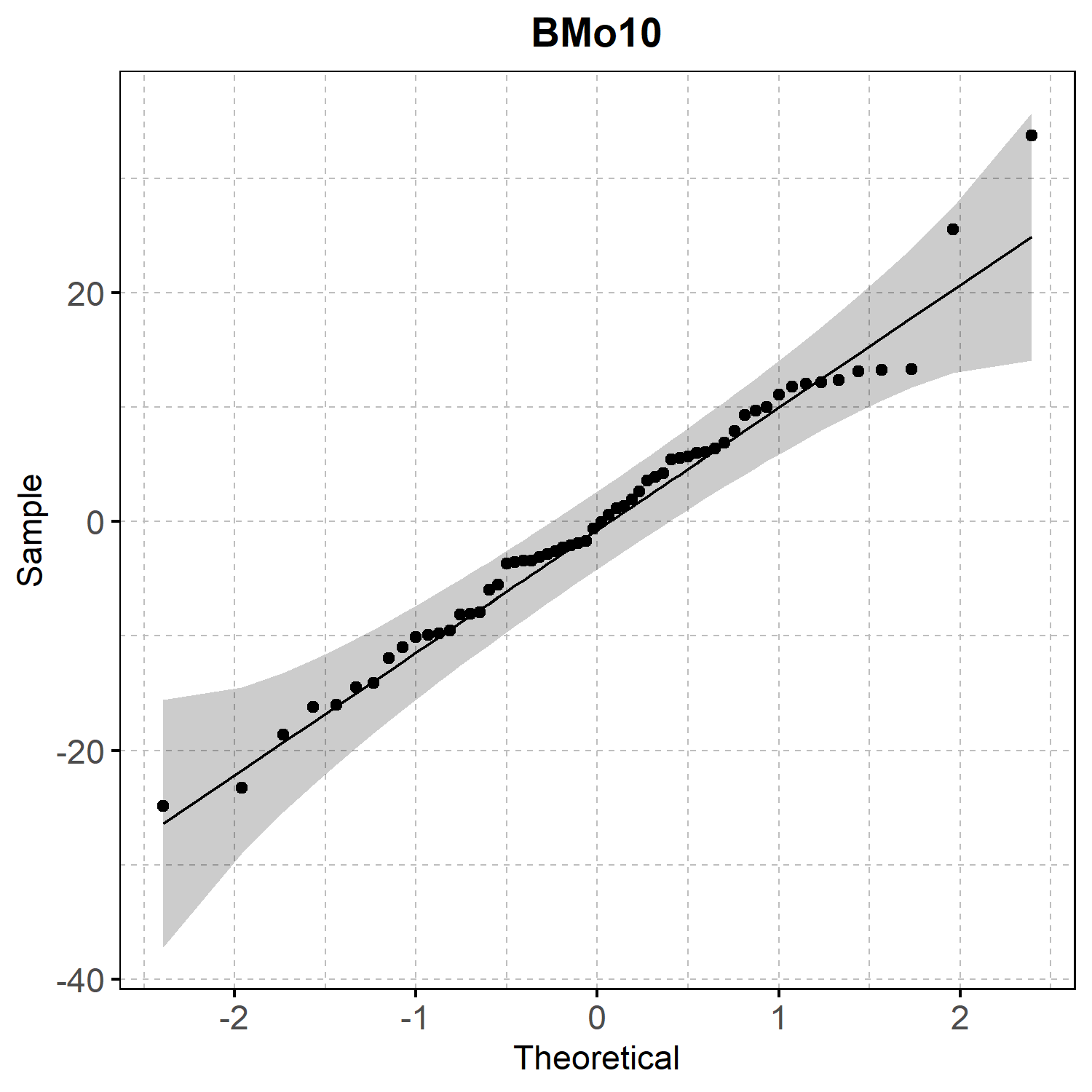

Supplement: Supplementary file 2 — Supplementary Information 2. [file 41598_2023_33504_MOESM2_ESM.zip › BMo010_normality.png]

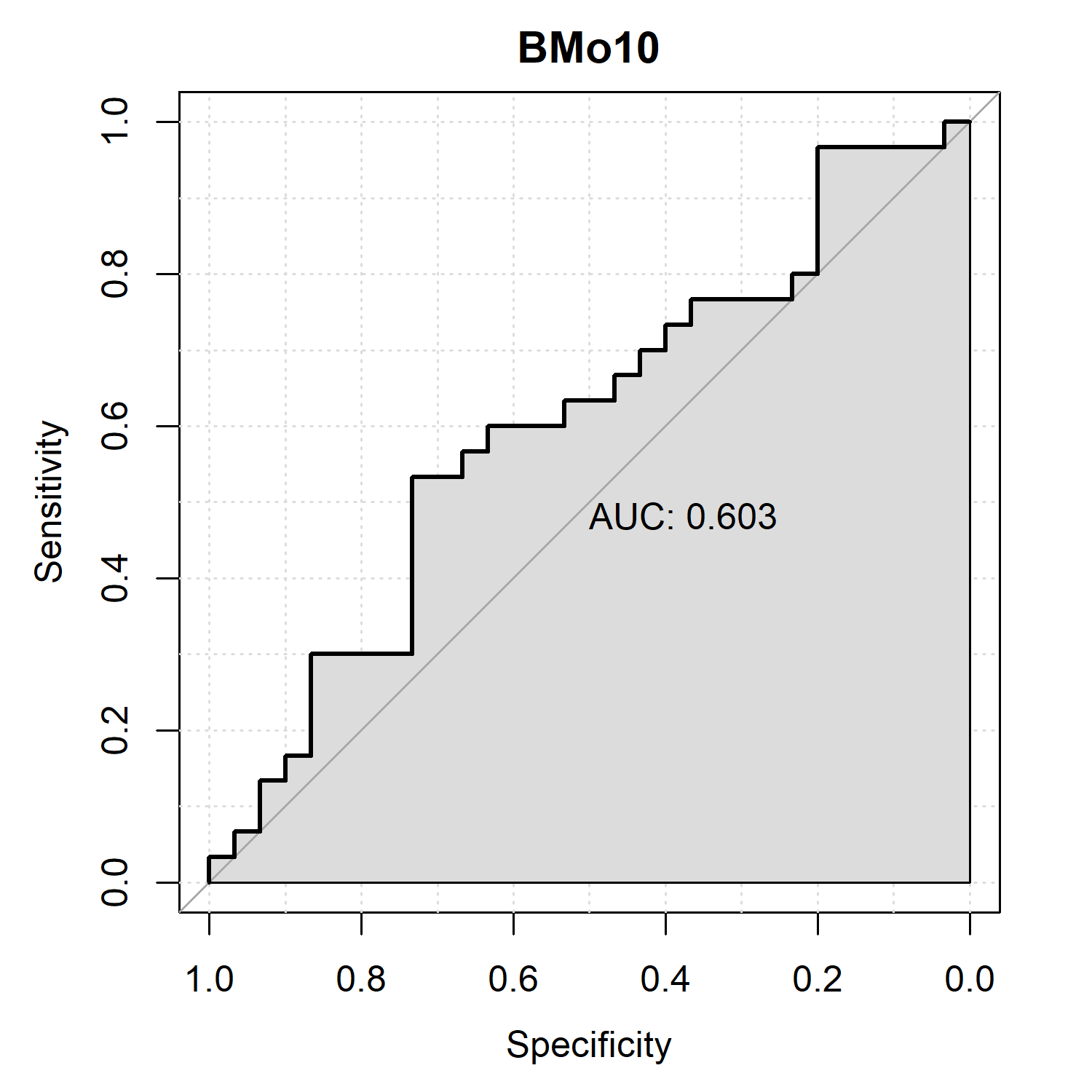

Supplement: Supplementary file 2 — Supplementary Information 2. [file 41598_2023_33504_MOESM2_ESM.zip › BMo010_ROC.png]

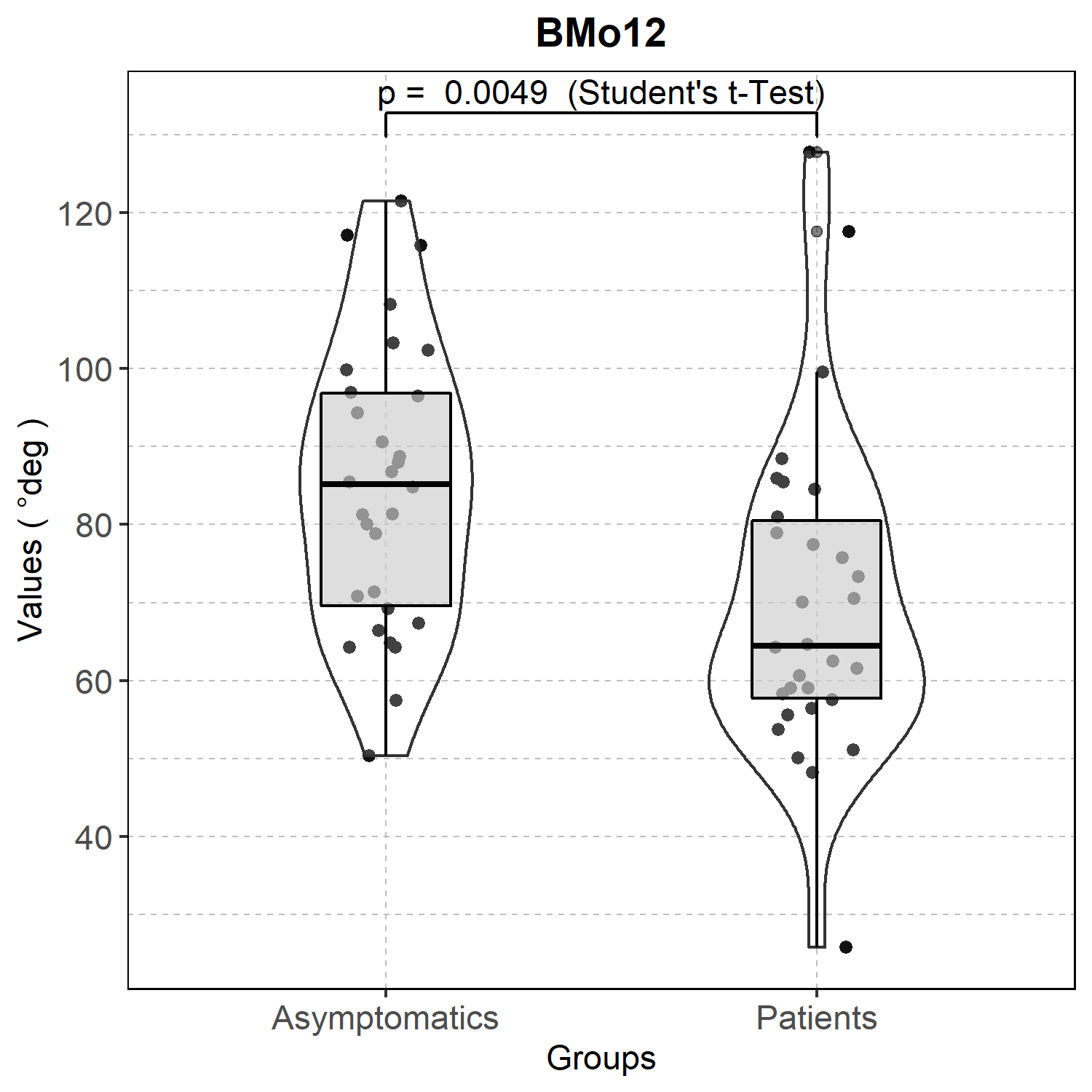

Supplement: Supplementary file 2 — Supplementary Information 2. [file 41598_2023_33504_MOESM2_ESM.zip › BMo012_boxplot.png]

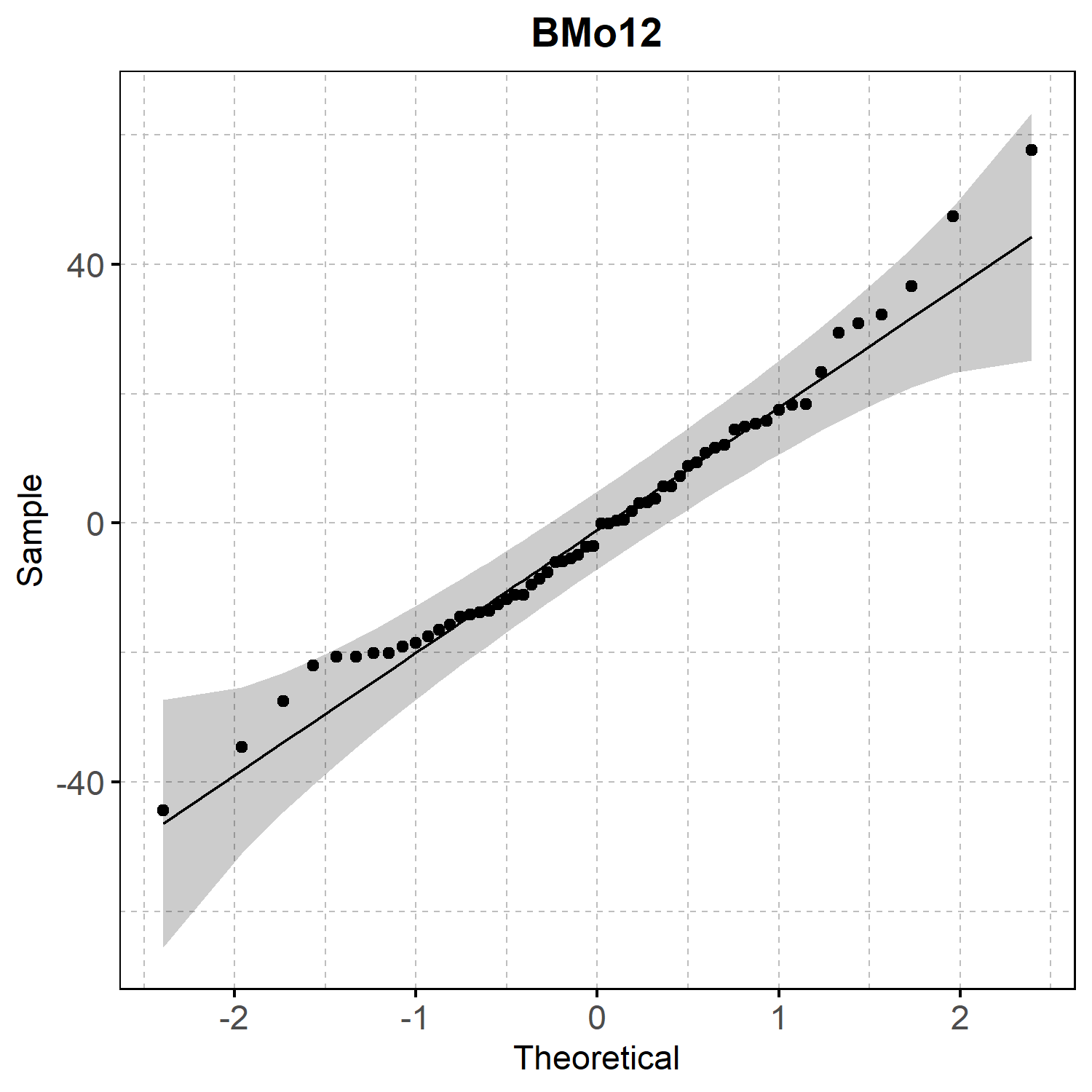

Supplement: Supplementary file 2 — Supplementary Information 2. [file 41598_2023_33504_MOESM2_ESM.zip › BMo012_normality.png]

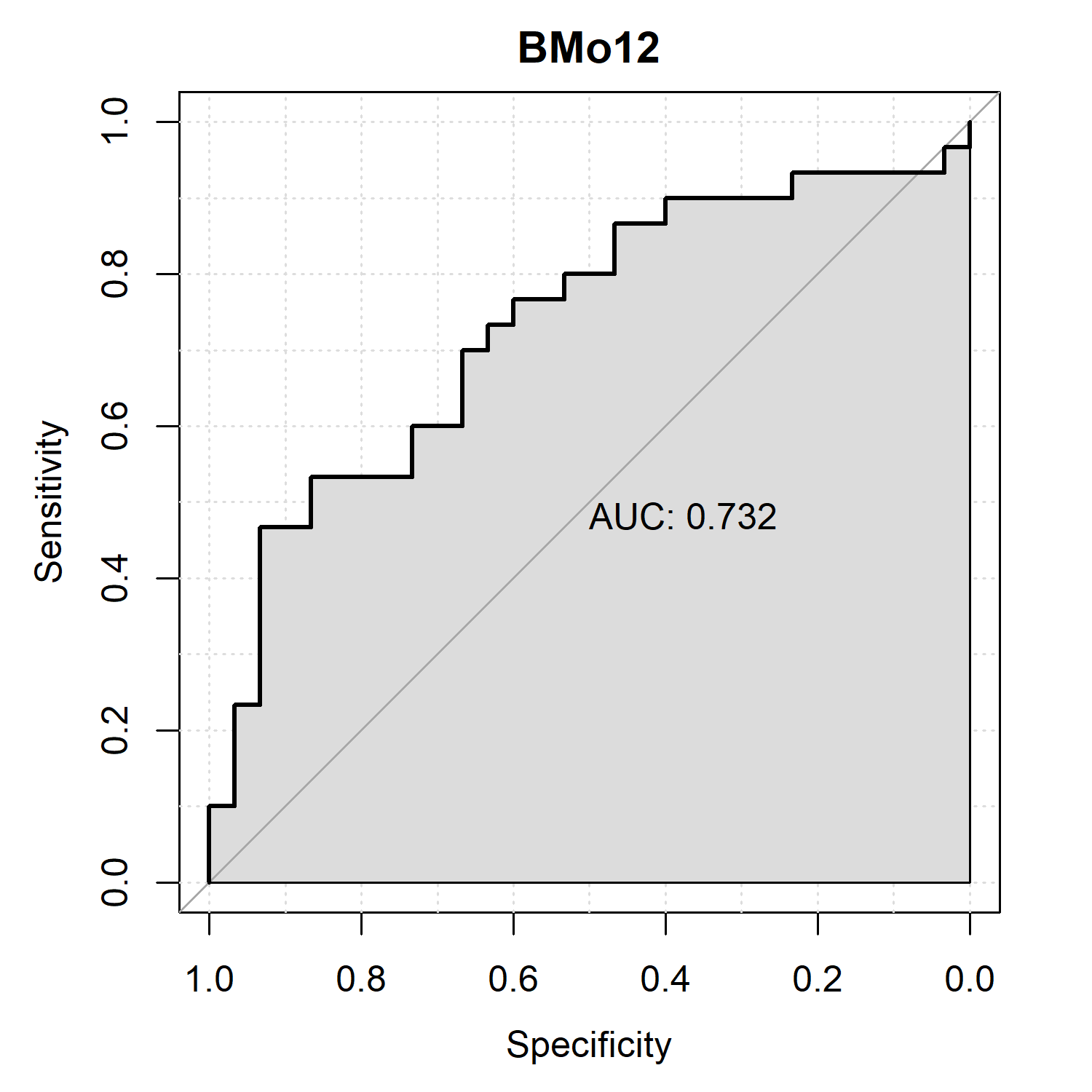

Supplement: Supplementary file 2 — Supplementary Information 2. [file 41598_2023_33504_MOESM2_ESM.zip › BMo012_ROC.png]

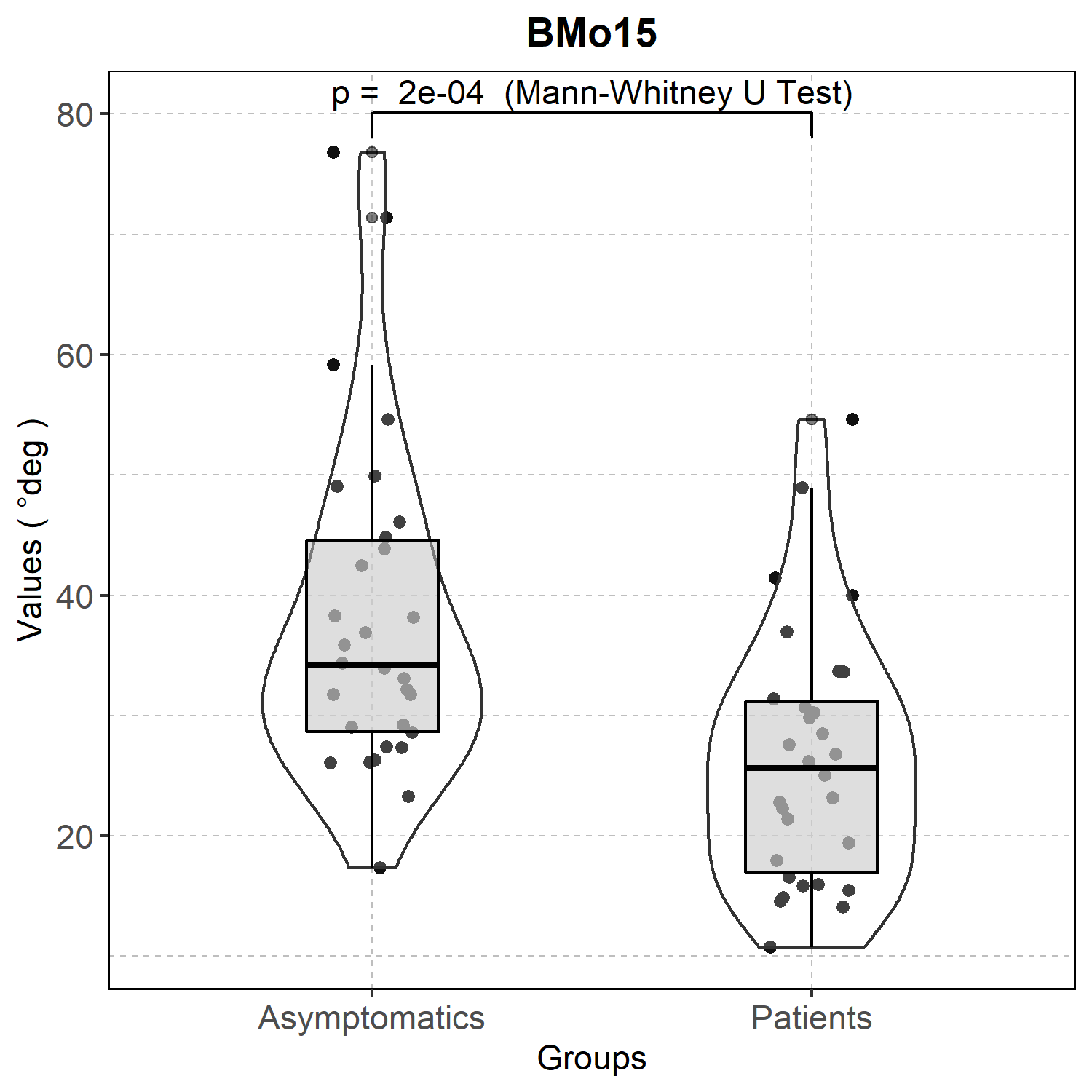

Supplement: Supplementary file 2 — Supplementary Information 2. [file 41598_2023_33504_MOESM2_ESM.zip › BMo015_boxplot.png]

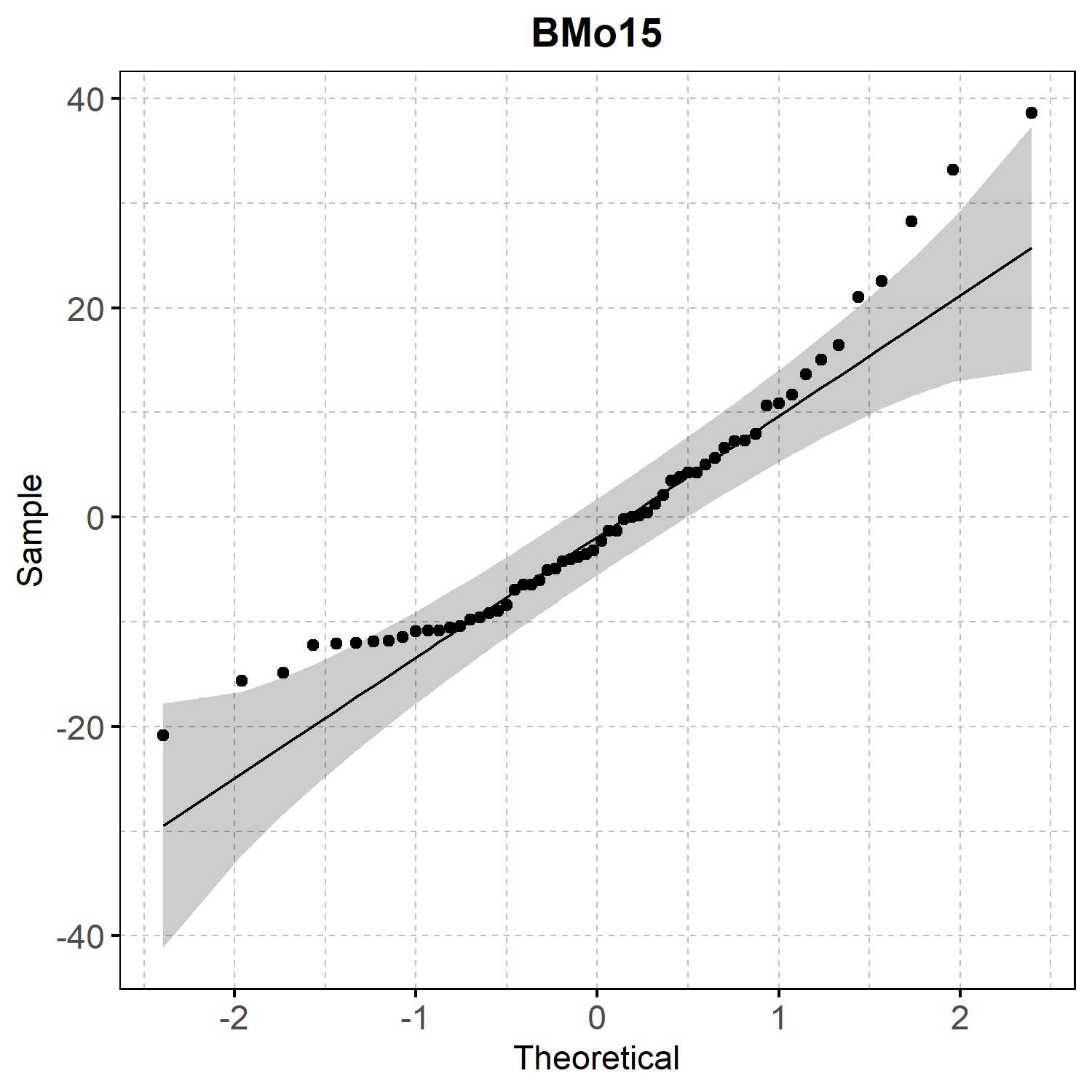

Supplement: Supplementary file 2 — Supplementary Information 2. [file 41598_2023_33504_MOESM2_ESM.zip › BMo015_normality.png]

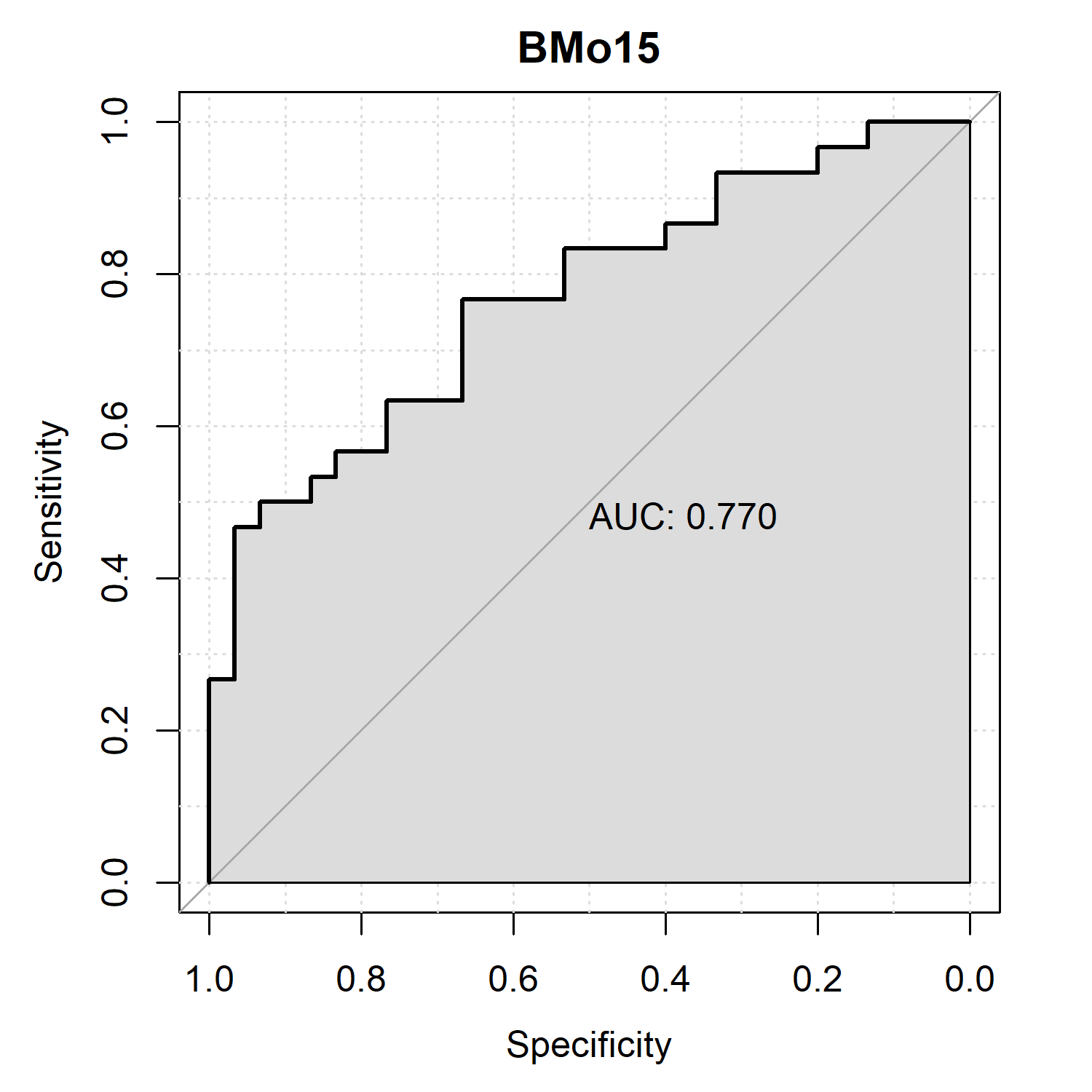

Supplement: Supplementary file 2 — Supplementary Information 2. [file 41598_2023_33504_MOESM2_ESM.zip › BMo015_ROC.png]

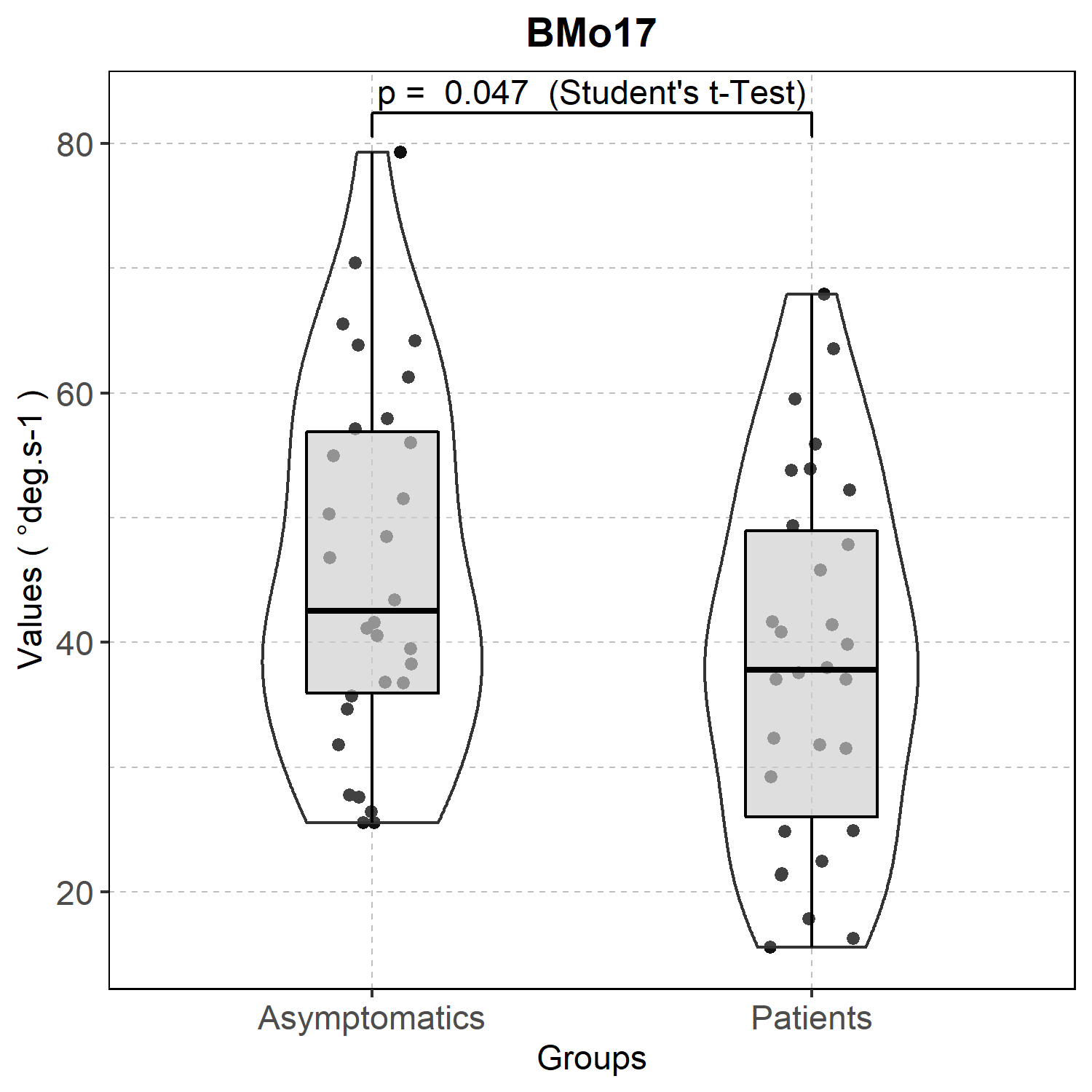

Supplement: Supplementary file 2 — Supplementary Information 2. [file 41598_2023_33504_MOESM2_ESM.zip › BMo017_boxplot.png]

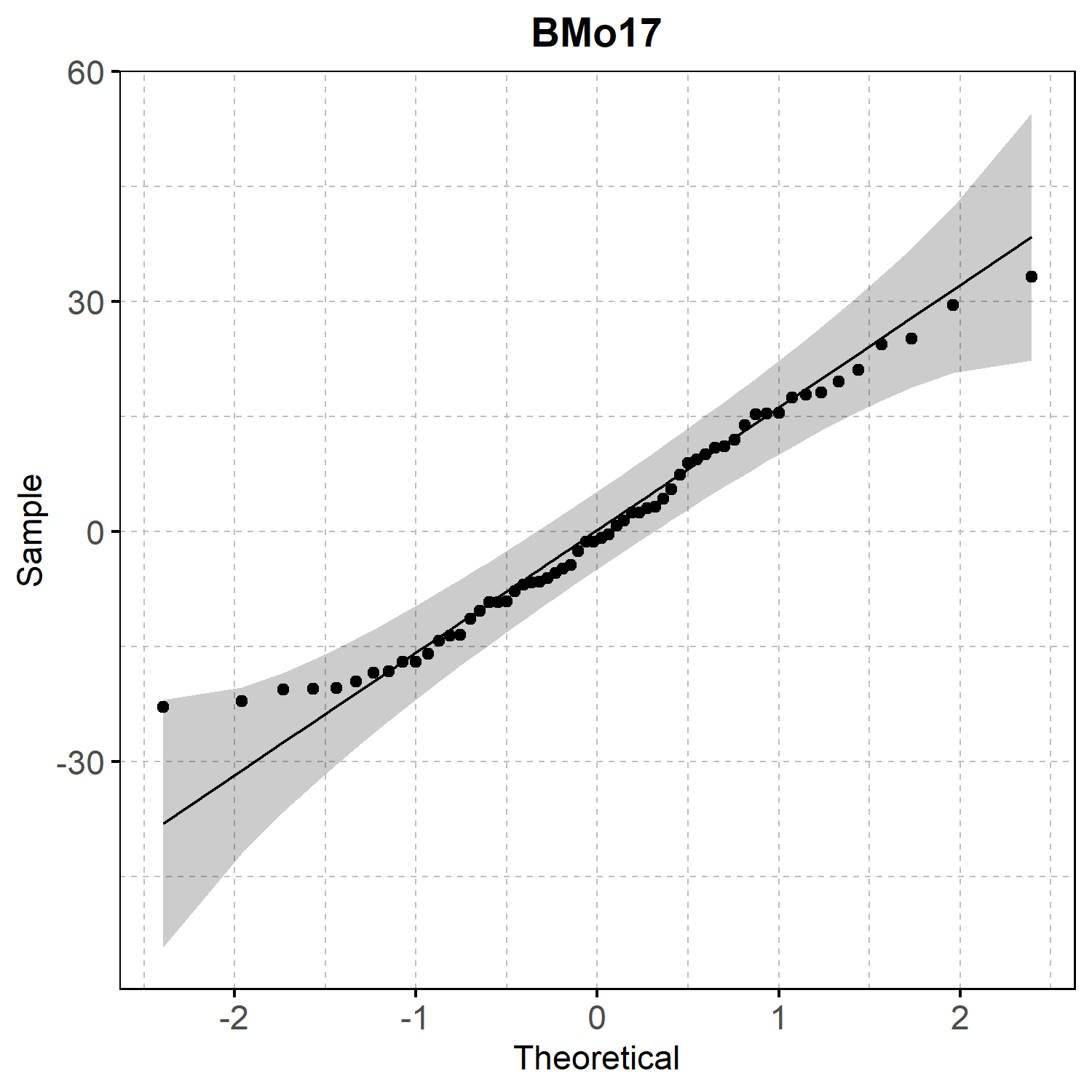

Supplement: Supplementary file 2 — Supplementary Information 2. [file 41598_2023_33504_MOESM2_ESM.zip › BMo017_normality.png]

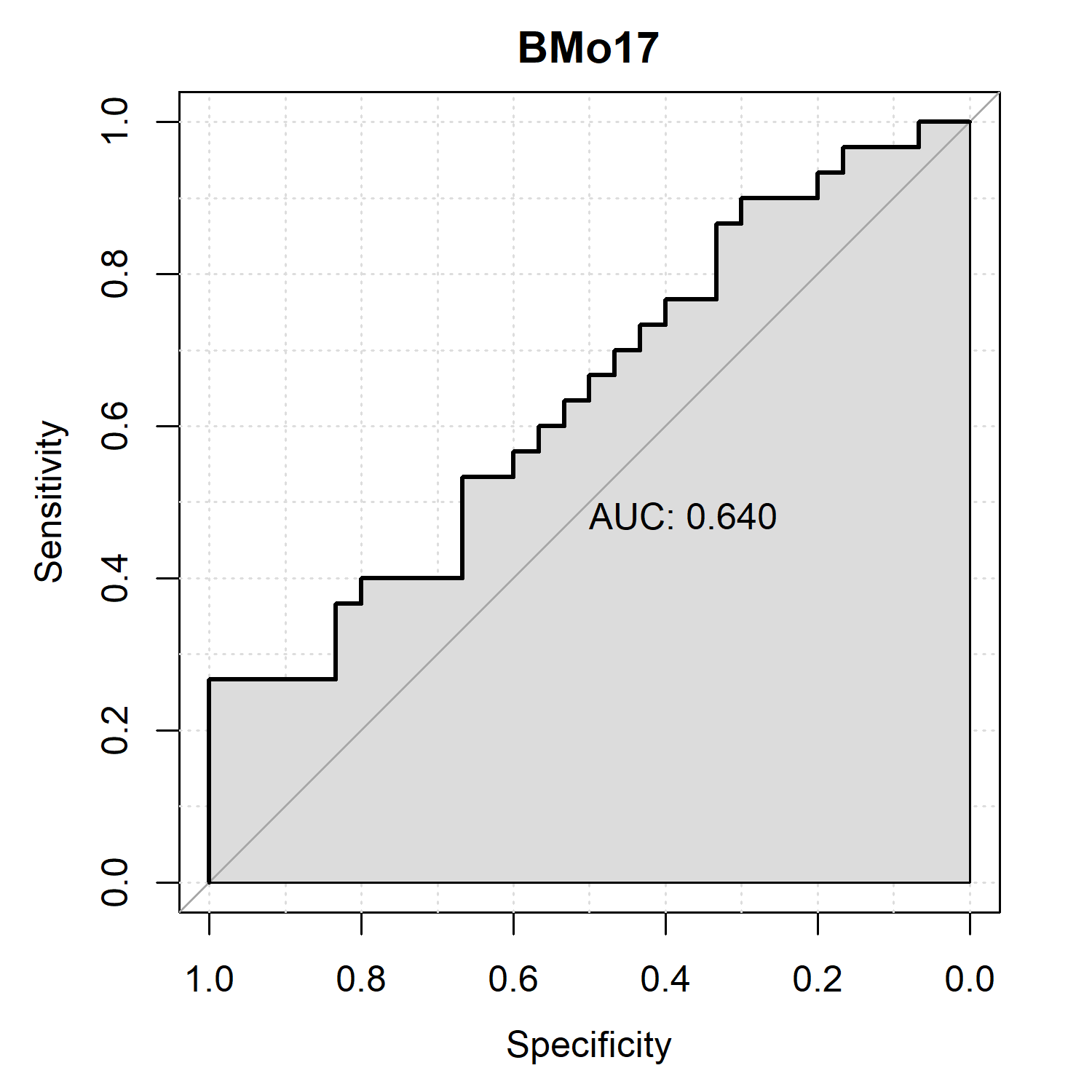

Supplement: Supplementary file 2 — Supplementary Information 2. [file 41598_2023_33504_MOESM2_ESM.zip › BMo017_ROC.png]

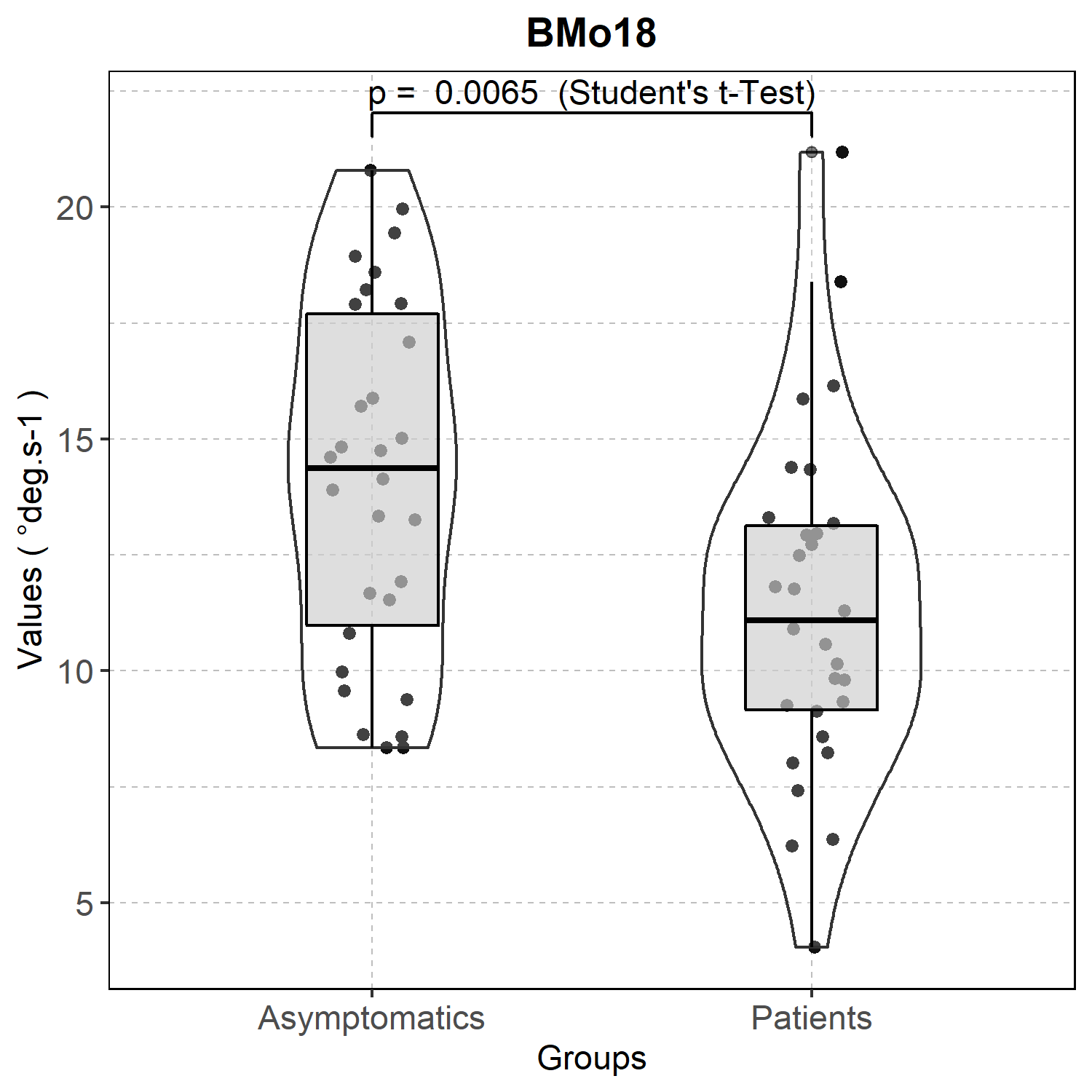

Supplement: Supplementary file 2 — Supplementary Information 2. [file 41598_2023_33504_MOESM2_ESM.zip › BMo018_boxplot.png]

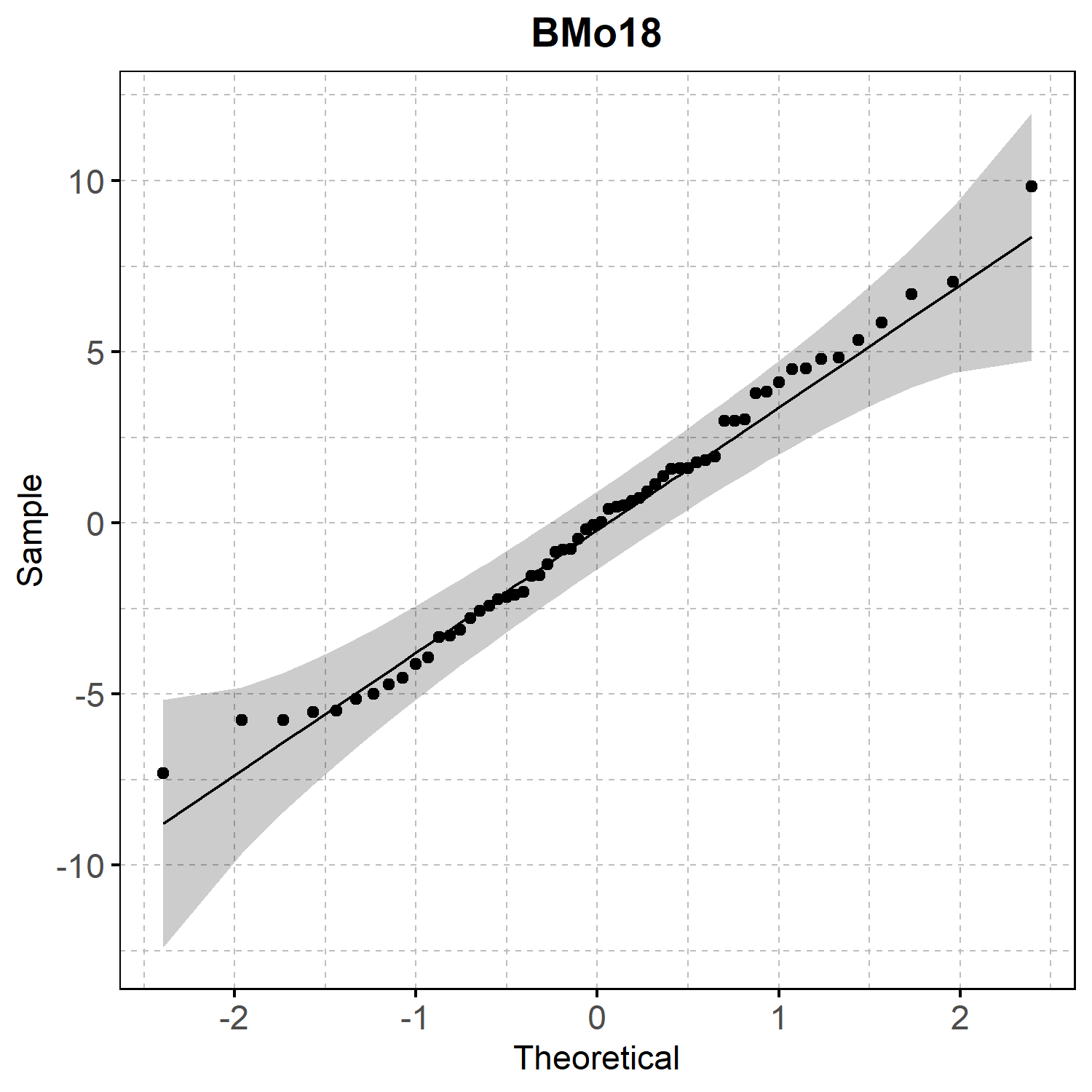

Supplement: Supplementary file 2 — Supplementary Information 2. [file 41598_2023_33504_MOESM2_ESM.zip › BMo018_normality.png]

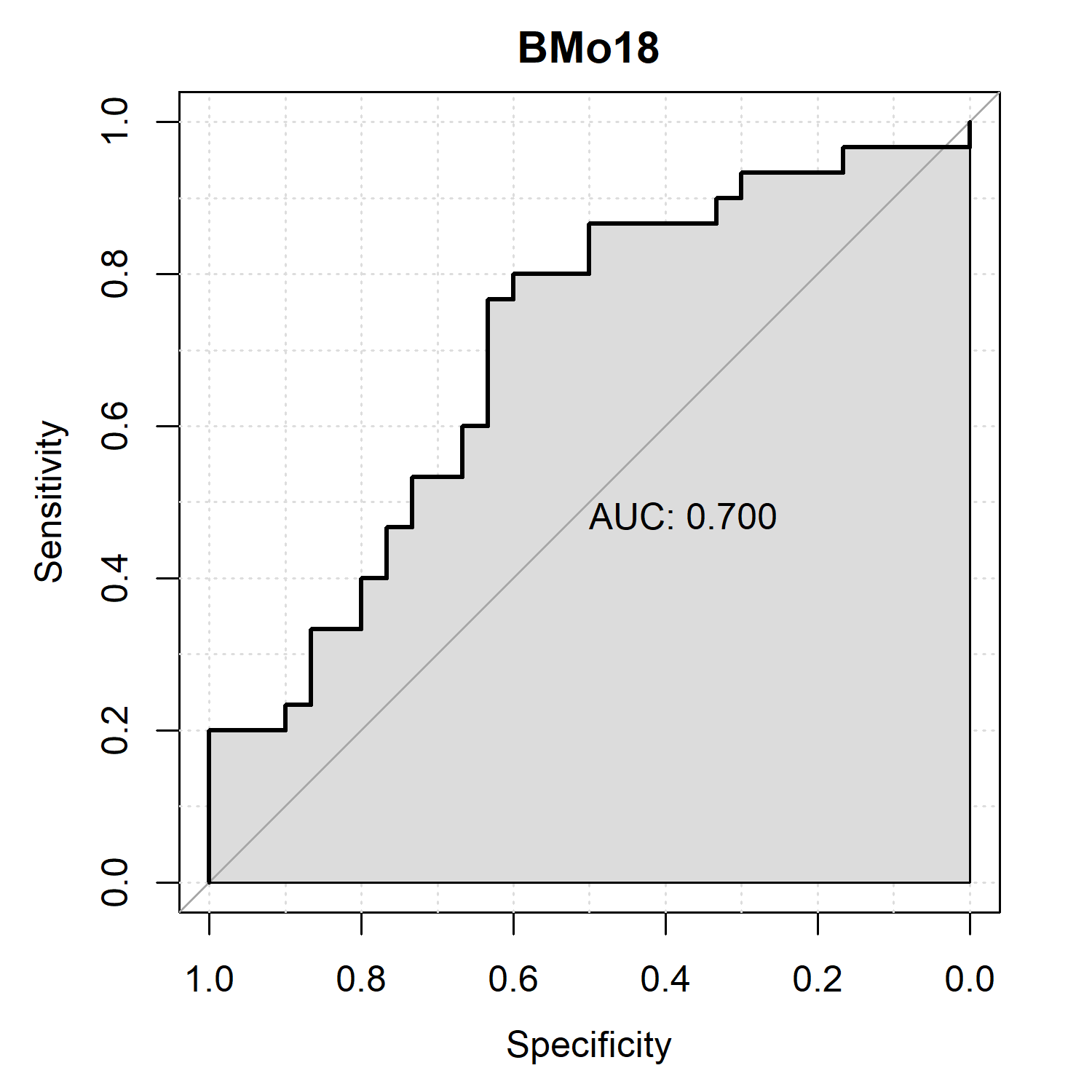

Supplement: Supplementary file 2 — Supplementary Information 2. [file 41598_2023_33504_MOESM2_ESM.zip › BMo018_ROC.png]

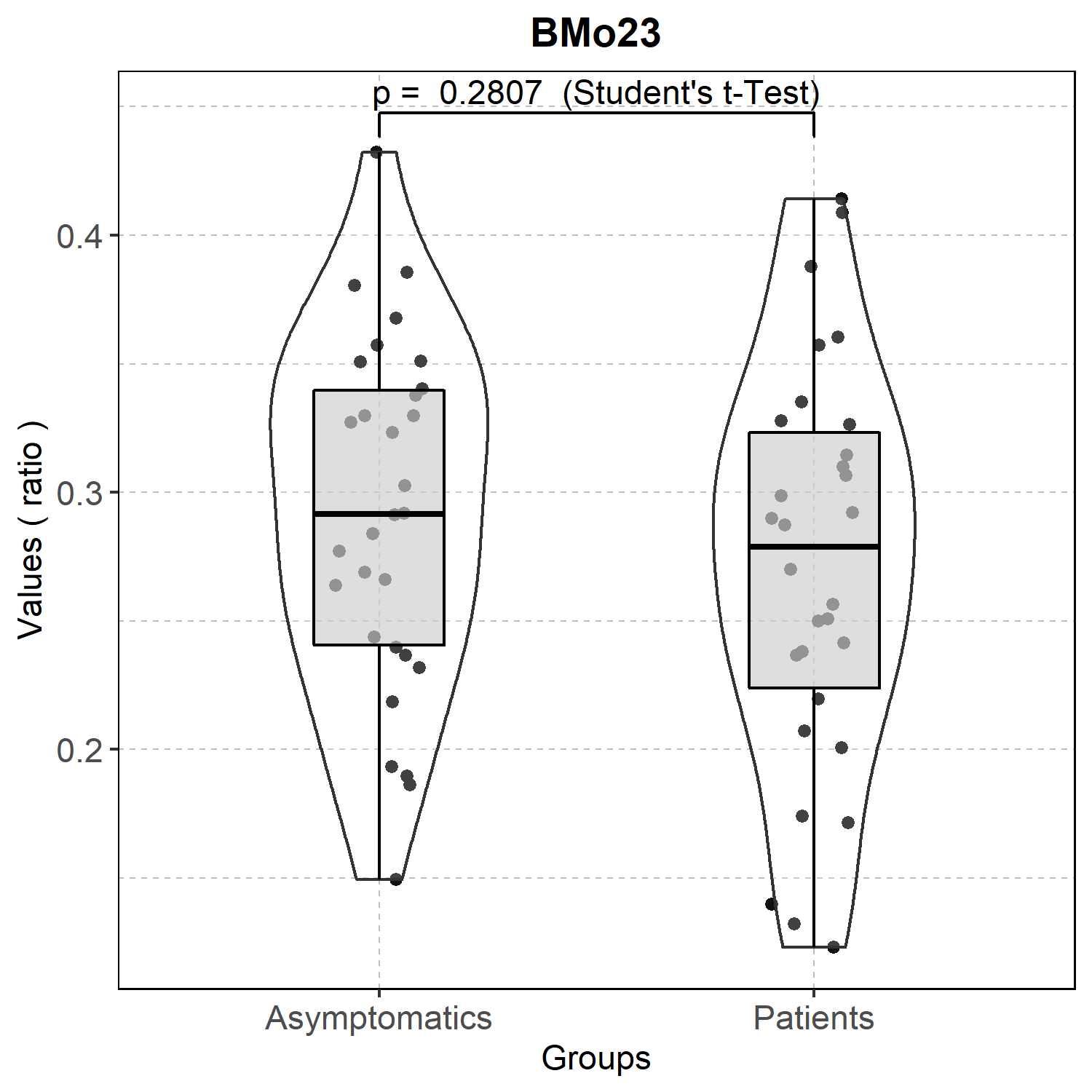

Supplement: Supplementary file 2 — Supplementary Information 2. [file 41598_2023_33504_MOESM2_ESM.zip › BMo023_boxplot.png]

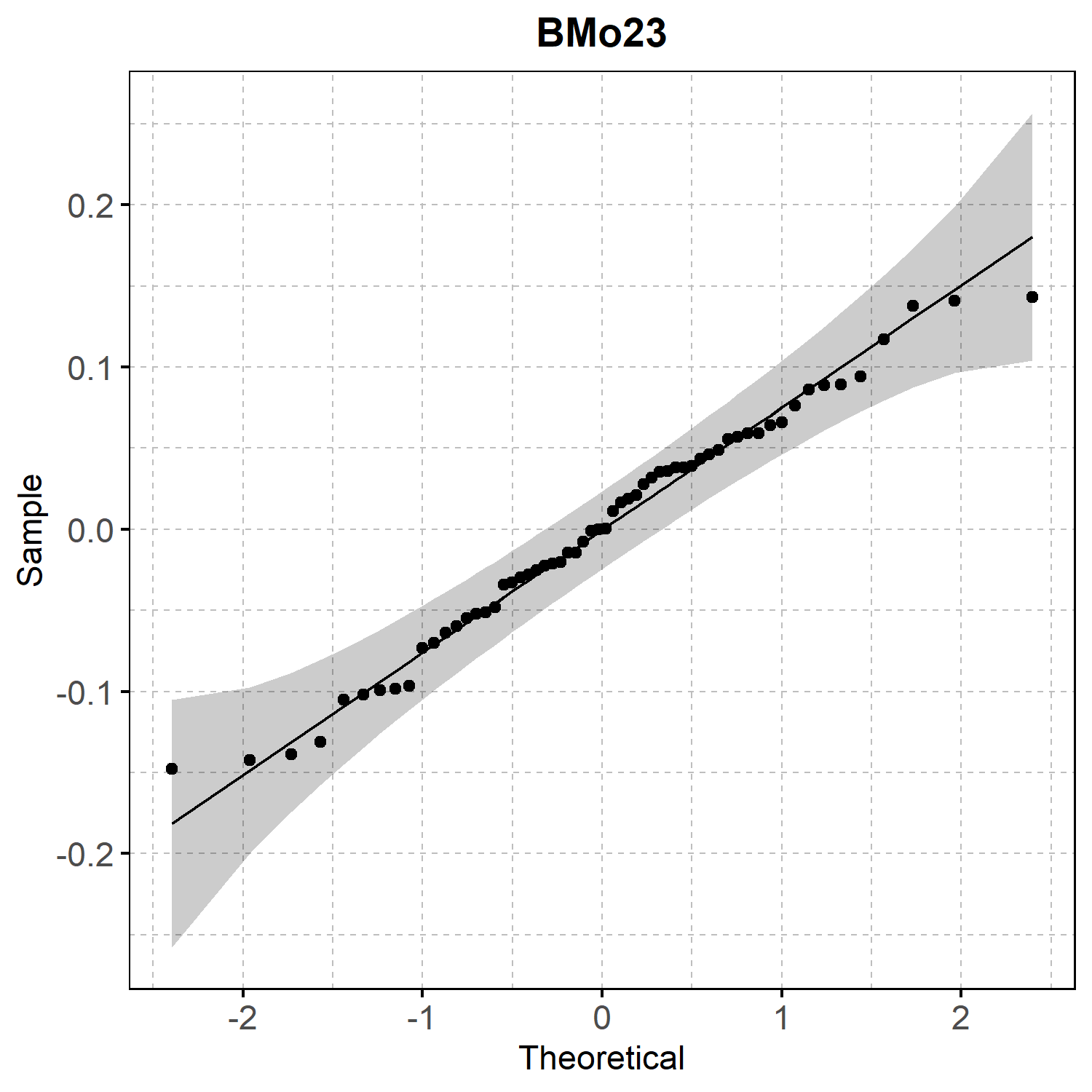

Supplement: Supplementary file 2 — Supplementary Information 2. [file 41598_2023_33504_MOESM2_ESM.zip › BMo023_normality.png]

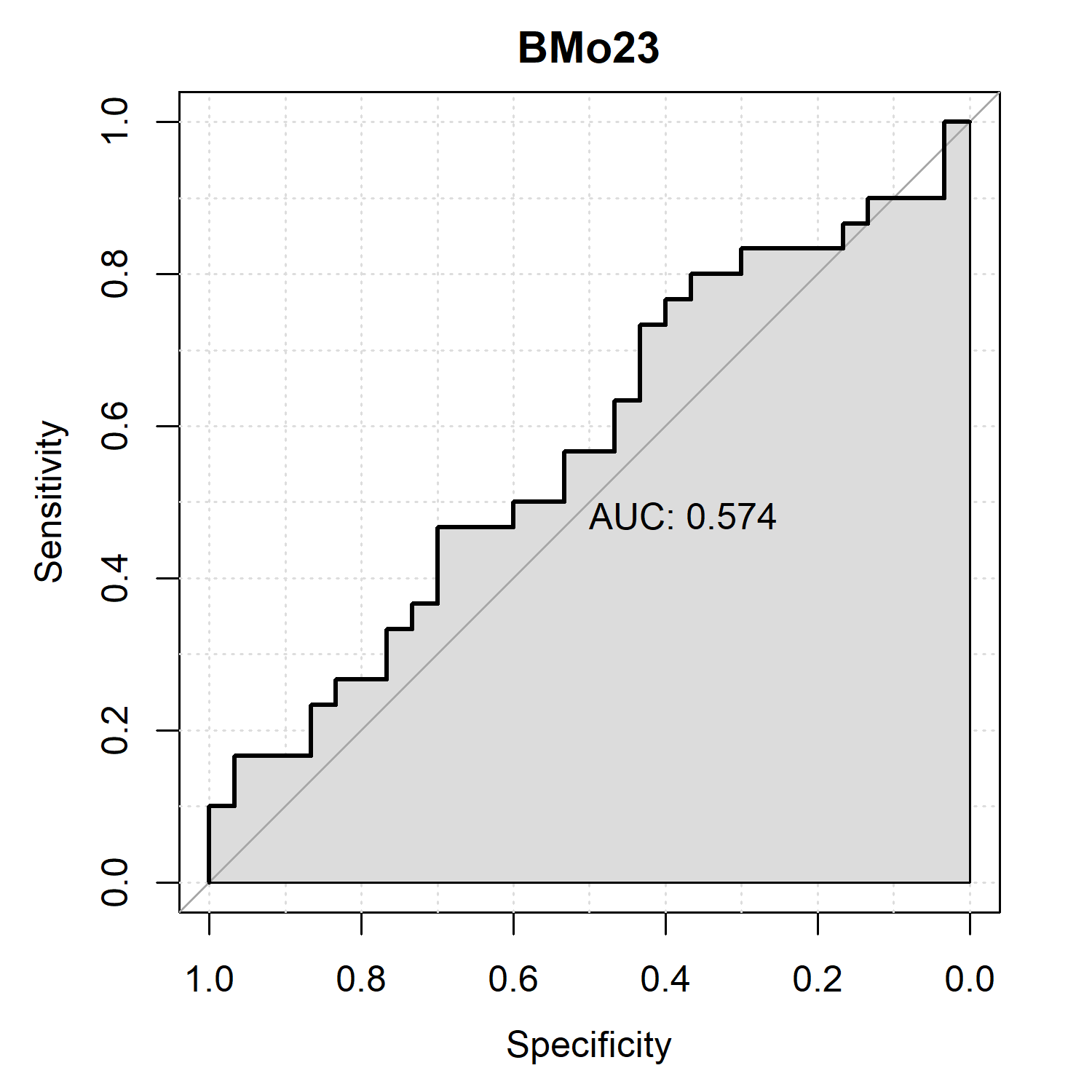

Supplement: Supplementary file 2 — Supplementary Information 2. [file 41598_2023_33504_MOESM2_ESM.zip › BMo023_ROC.png]

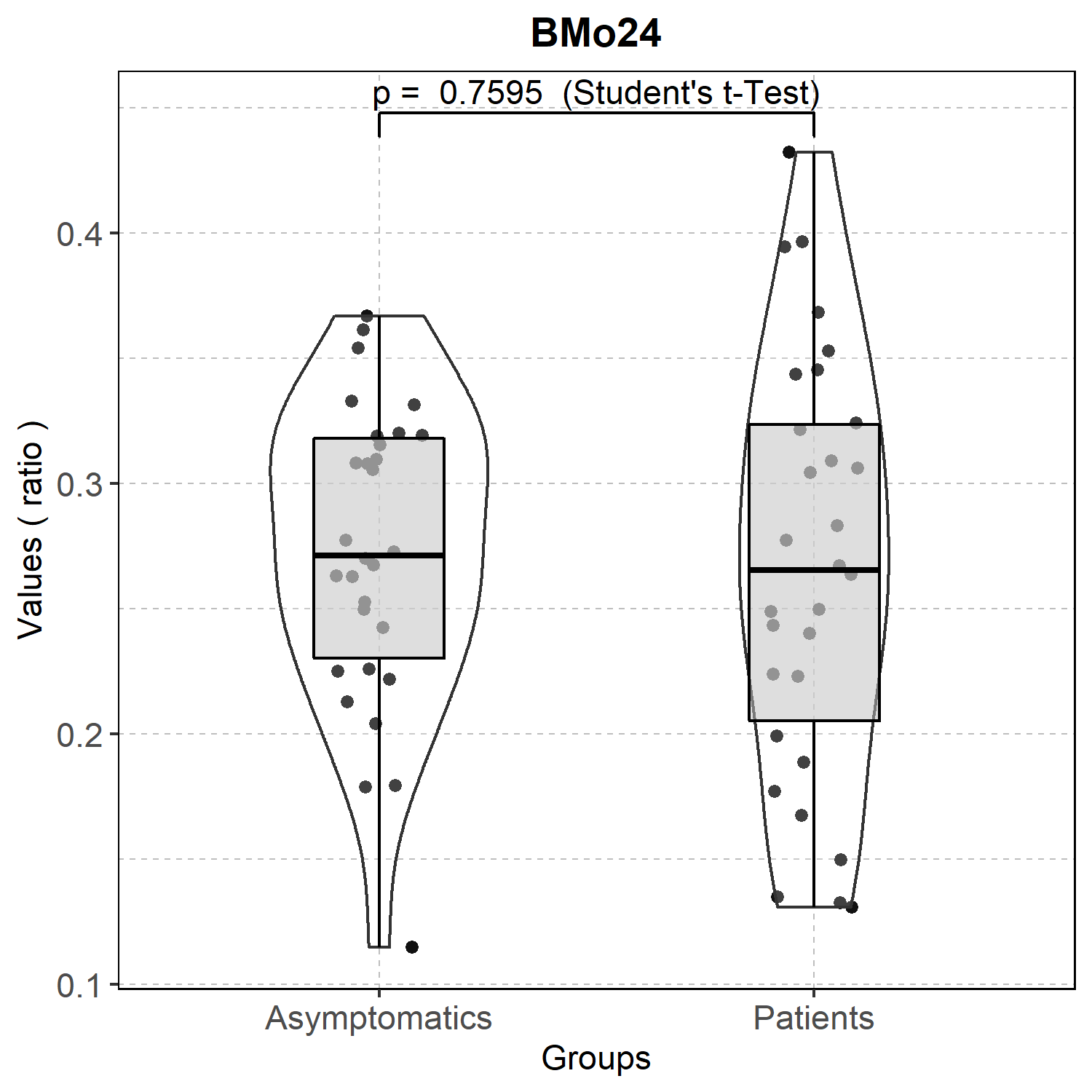

Supplement: Supplementary file 2 — Supplementary Information 2. [file 41598_2023_33504_MOESM2_ESM.zip › BMo024_boxplot.png]

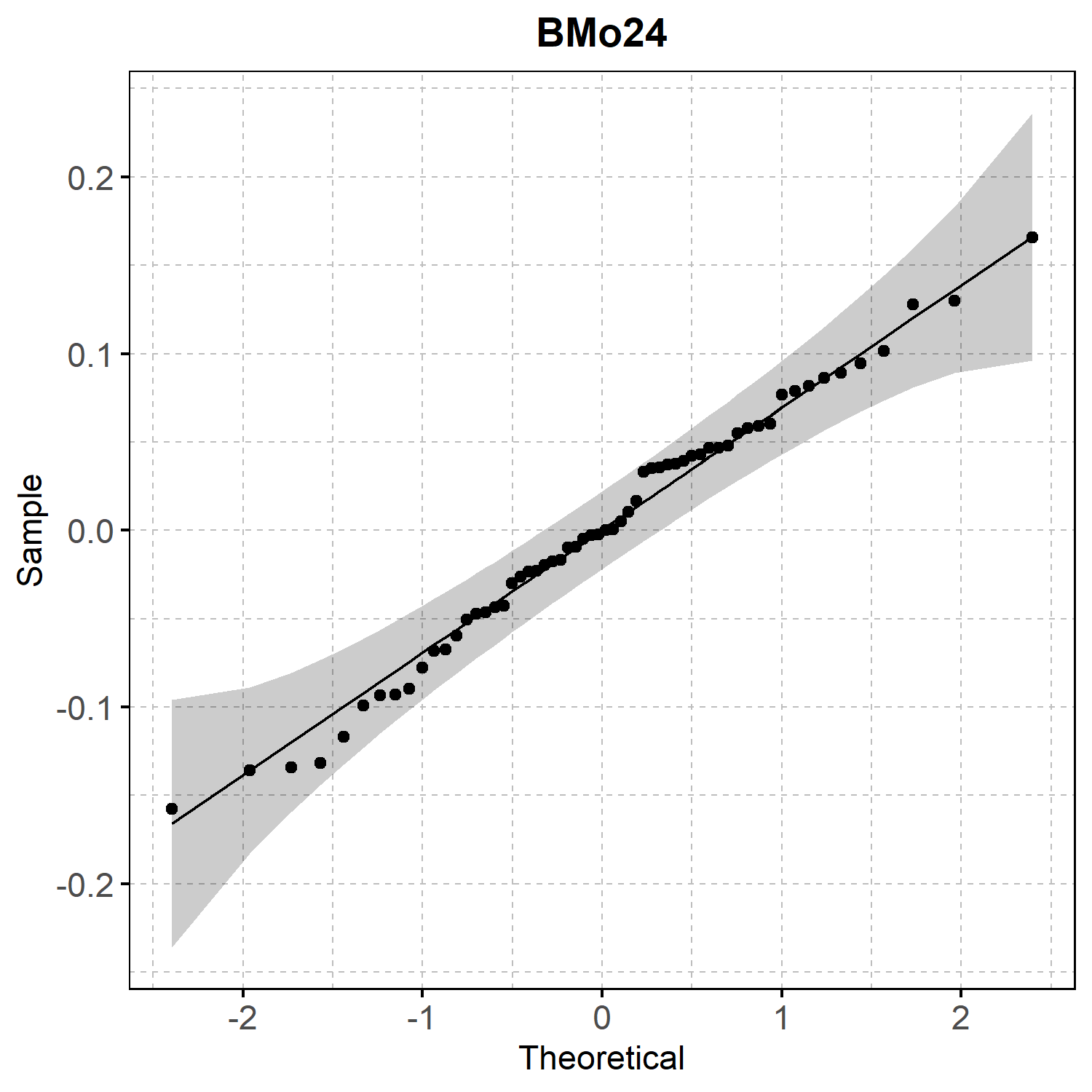

Supplement: Supplementary file 2 — Supplementary Information 2. [file 41598_2023_33504_MOESM2_ESM.zip › BMo024_normality.png]

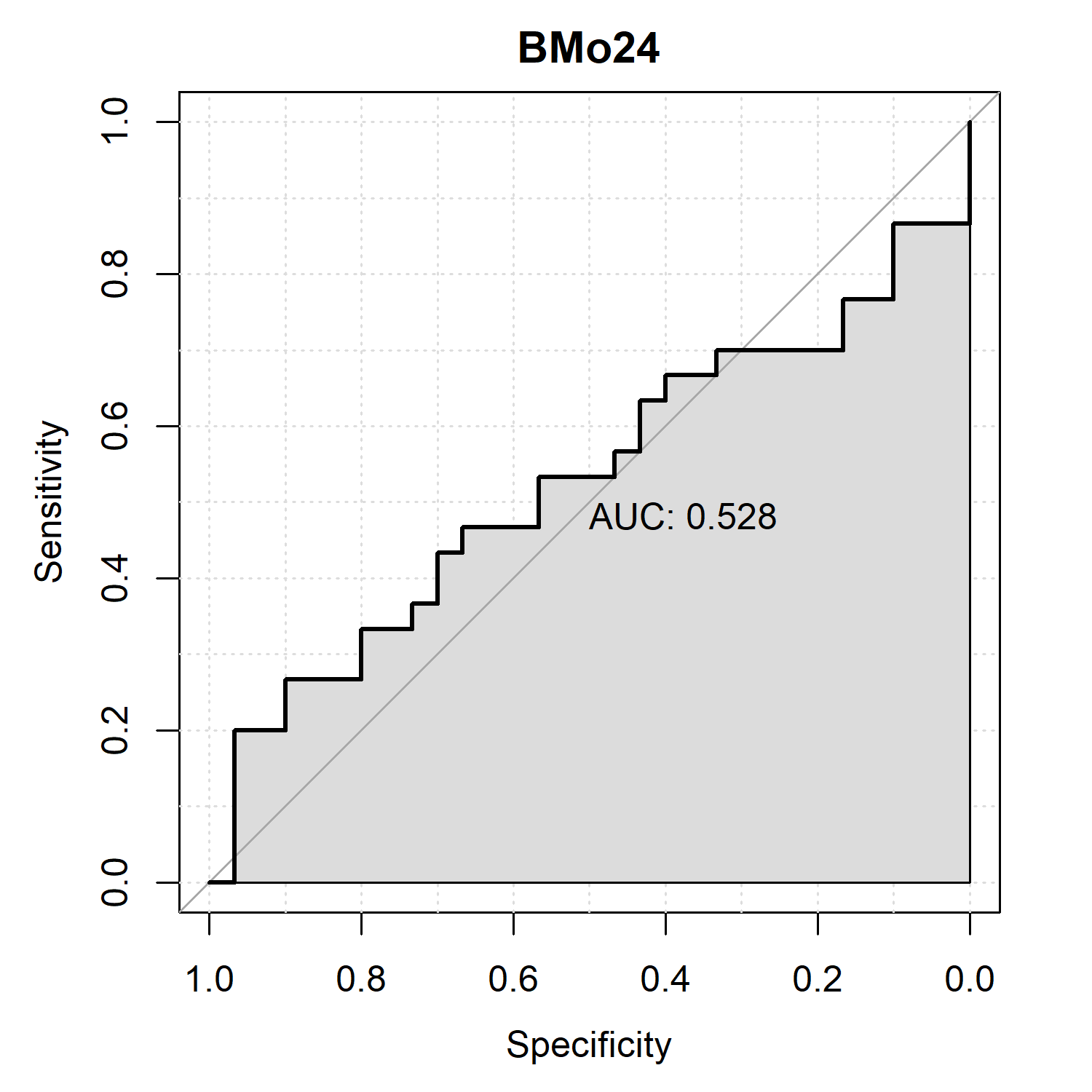

Supplement: Supplementary file 2 — Supplementary Information 2. [file 41598_2023_33504_MOESM2_ESM.zip › BMo024_ROC.png]

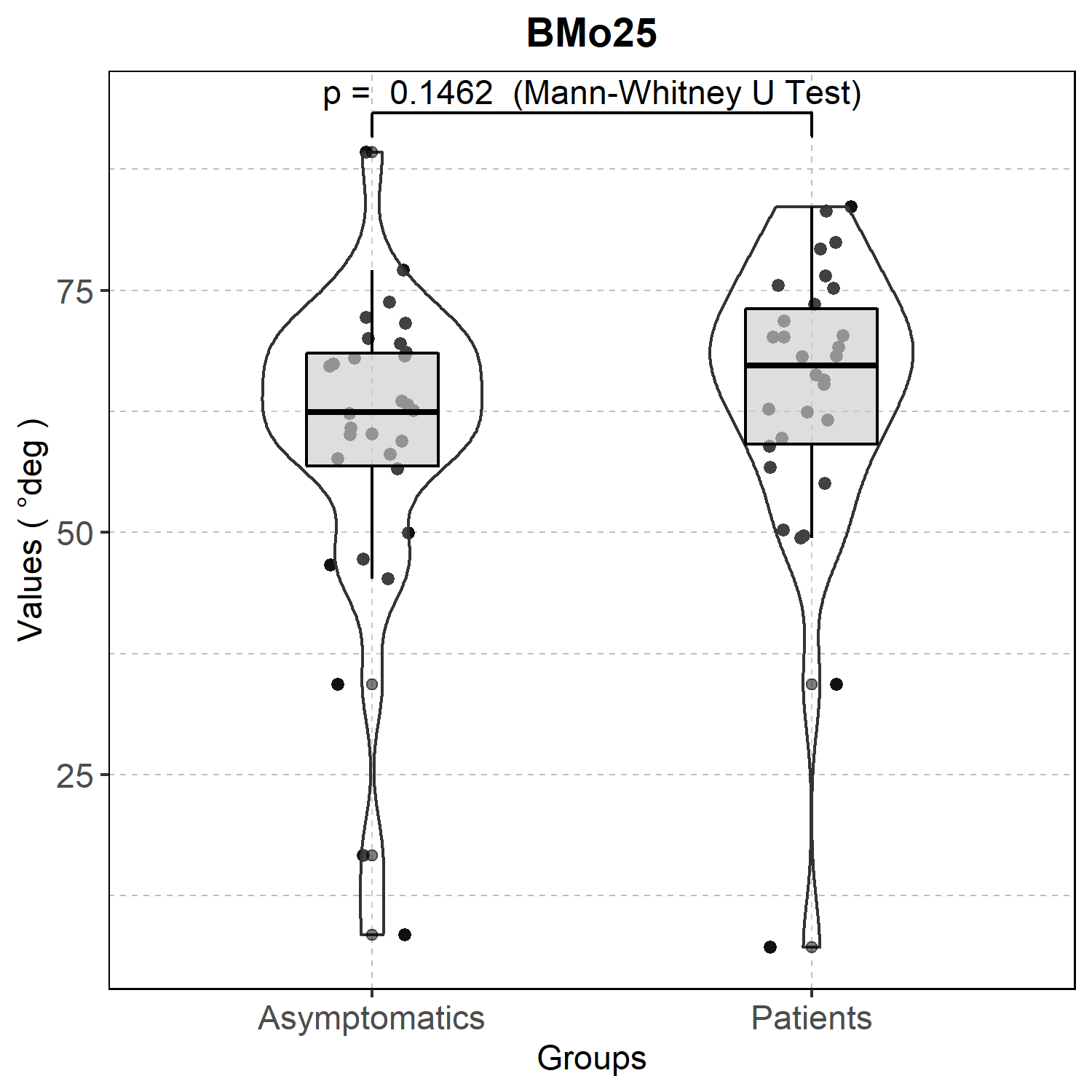

Supplement: Supplementary file 2 — Supplementary Information 2. [file 41598_2023_33504_MOESM2_ESM.zip › BMo025_boxplot.png]

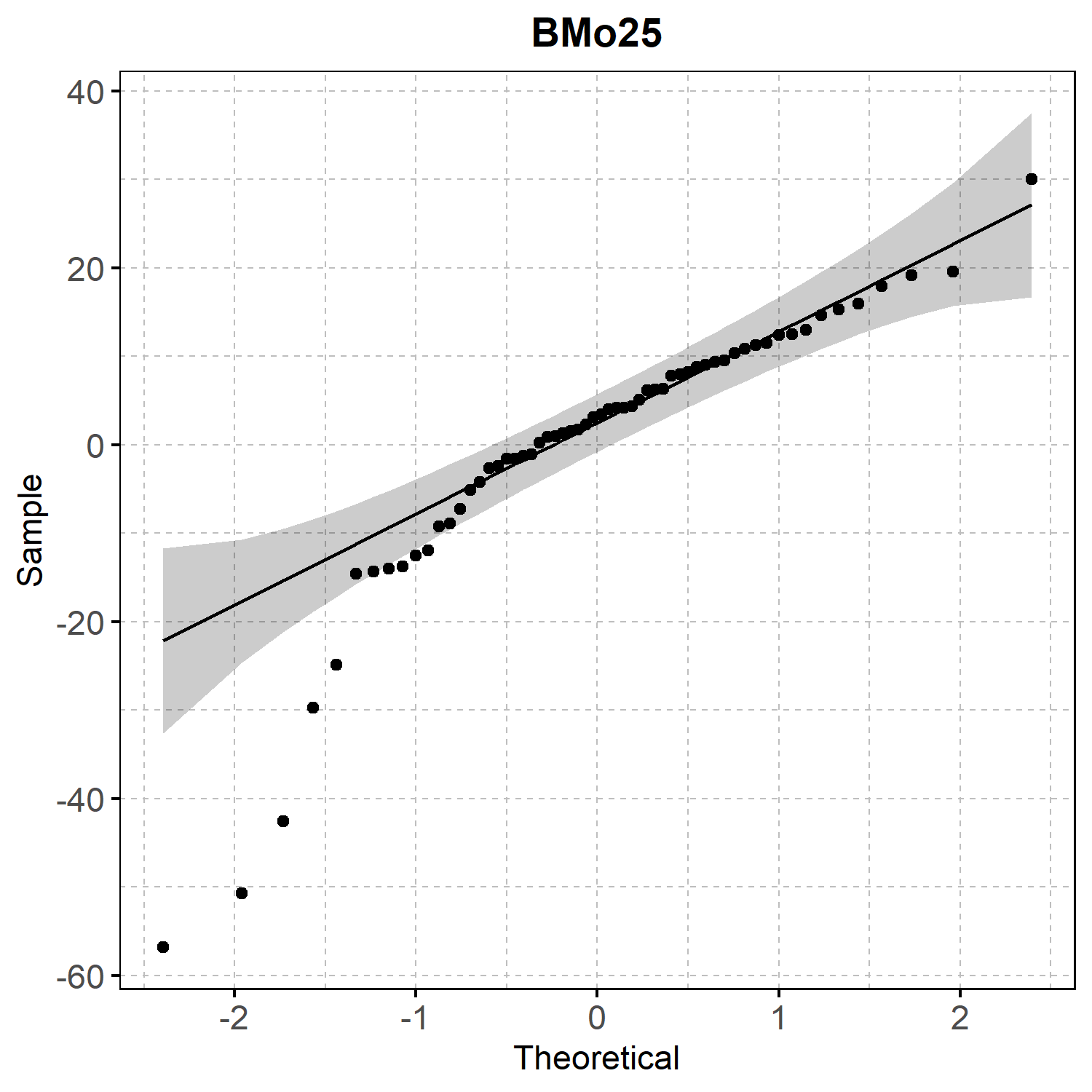

Supplement: Supplementary file 2 — Supplementary Information 2. [file 41598_2023_33504_MOESM2_ESM.zip › BMo025_normality.png]

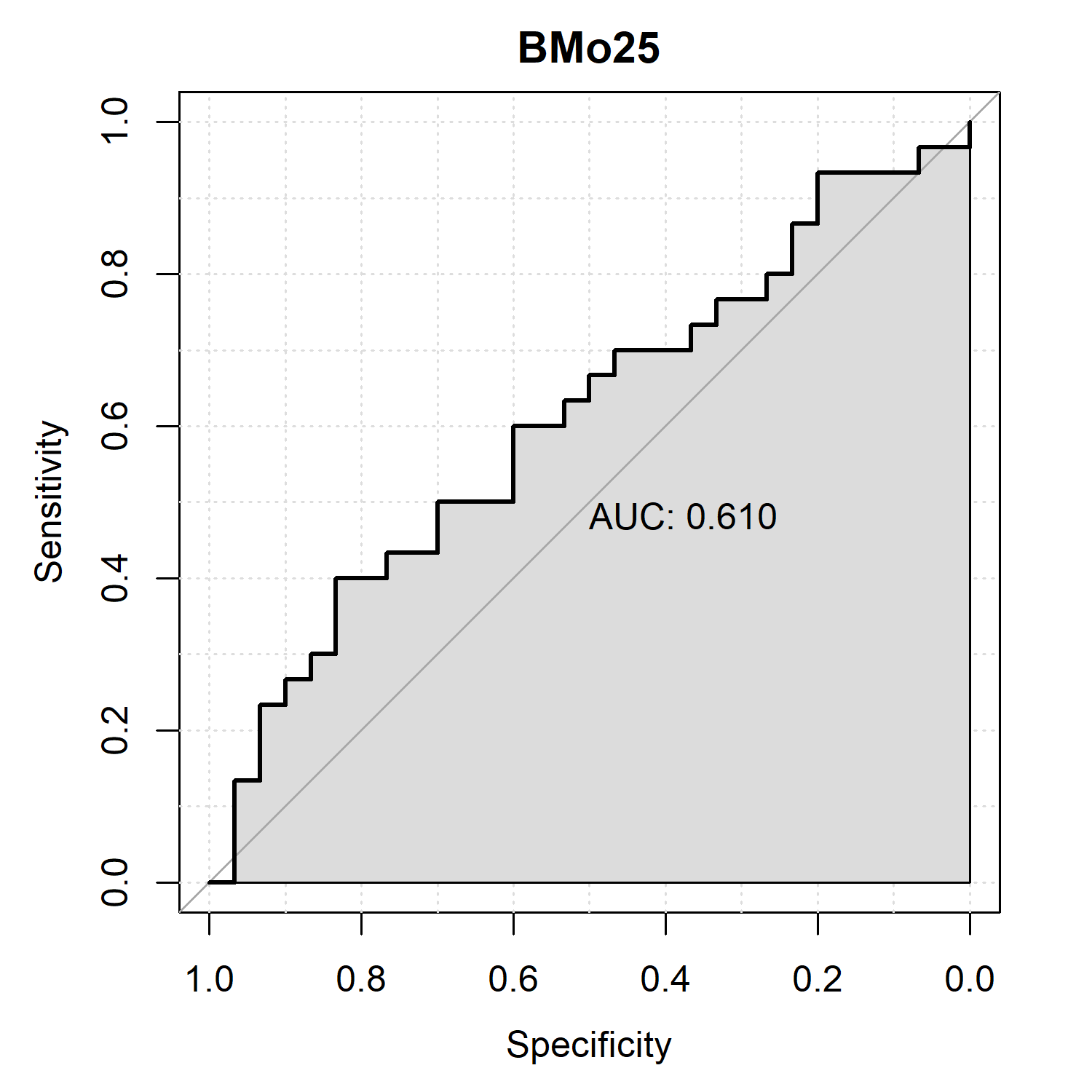

Supplement: Supplementary file 2 — Supplementary Information 2. [file 41598_2023_33504_MOESM2_ESM.zip › BMo025_ROC.png]

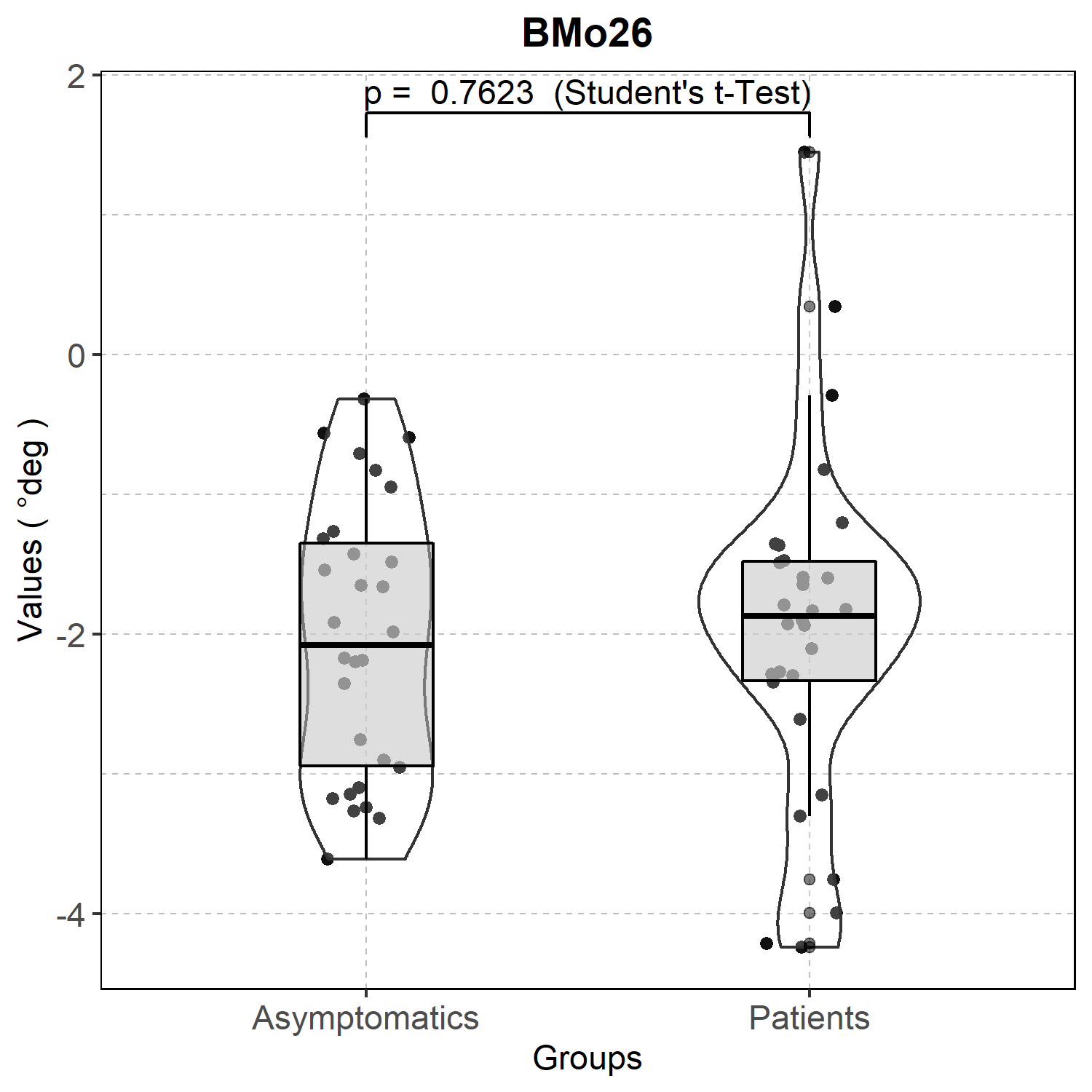

Supplement: Supplementary file 2 — Supplementary Information 2. [file 41598_2023_33504_MOESM2_ESM.zip › BMo026_boxplot.png]

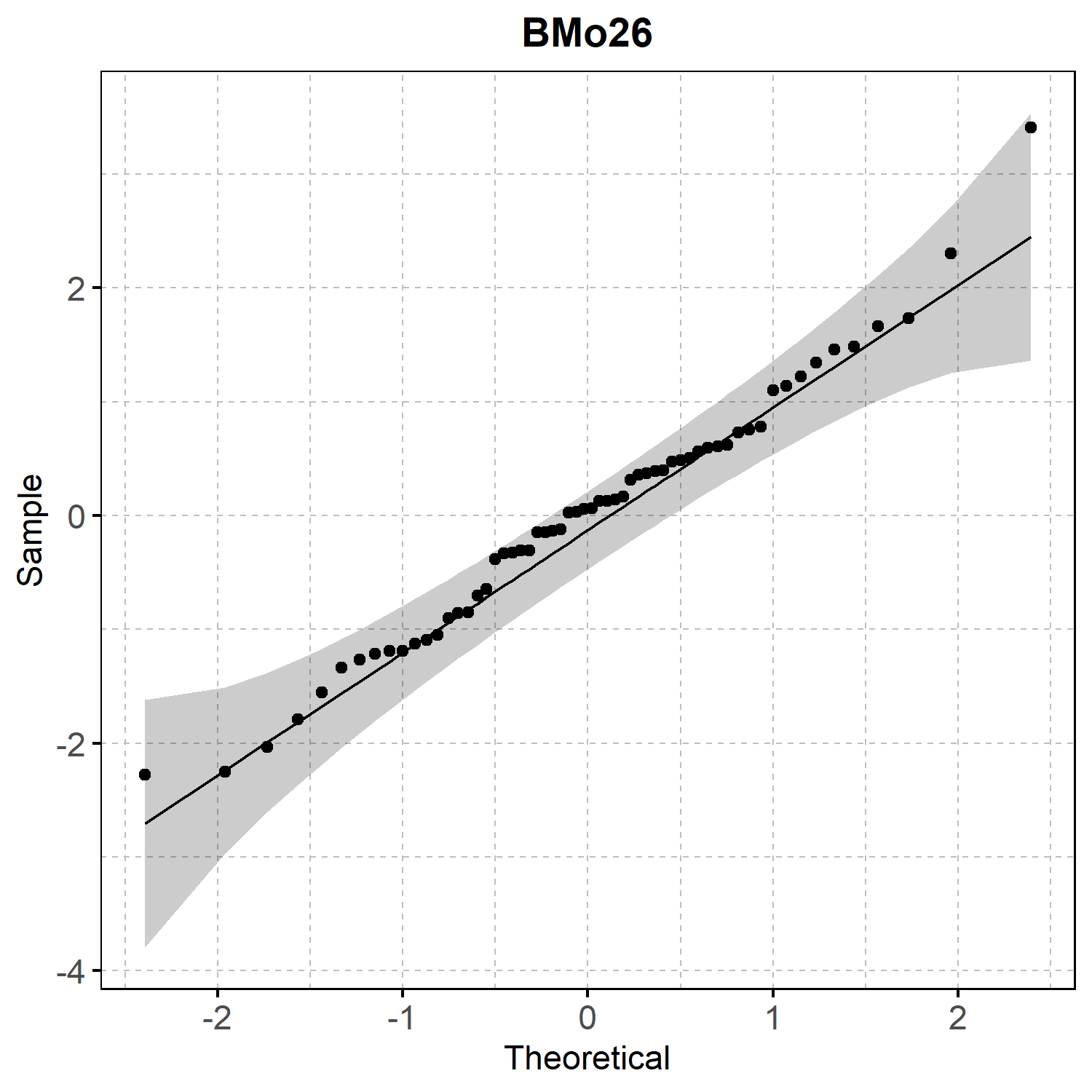

Supplement: Supplementary file 2 — Supplementary Information 2. [file 41598_2023_33504_MOESM2_ESM.zip › BMo026_normality.png]

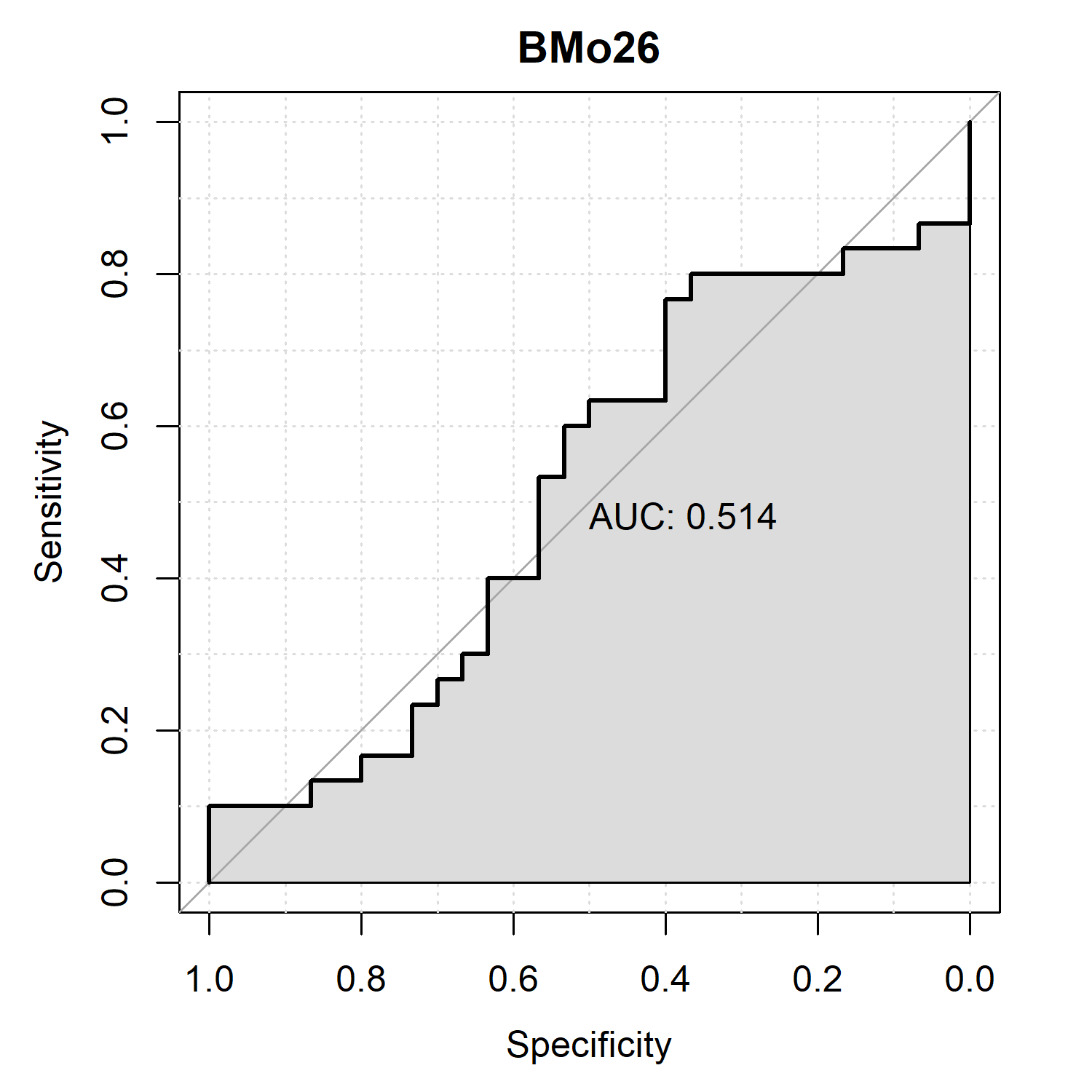

Supplement: Supplementary file 2 — Supplementary Information 2. [file 41598_2023_33504_MOESM2_ESM.zip › BMo026_ROC.png]

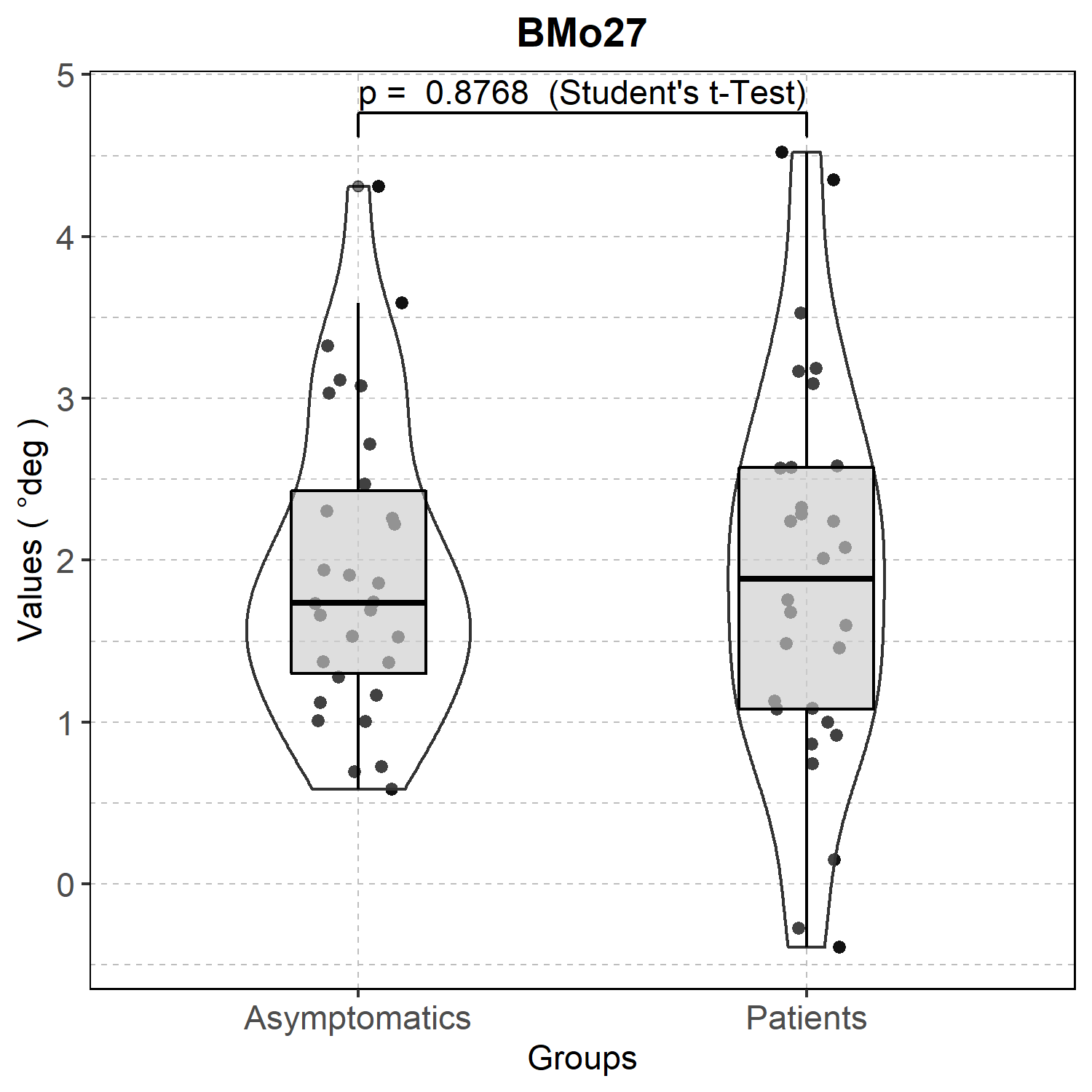

Supplement: Supplementary file 2 — Supplementary Information 2. [file 41598_2023_33504_MOESM2_ESM.zip › BMo027_boxplot.png]

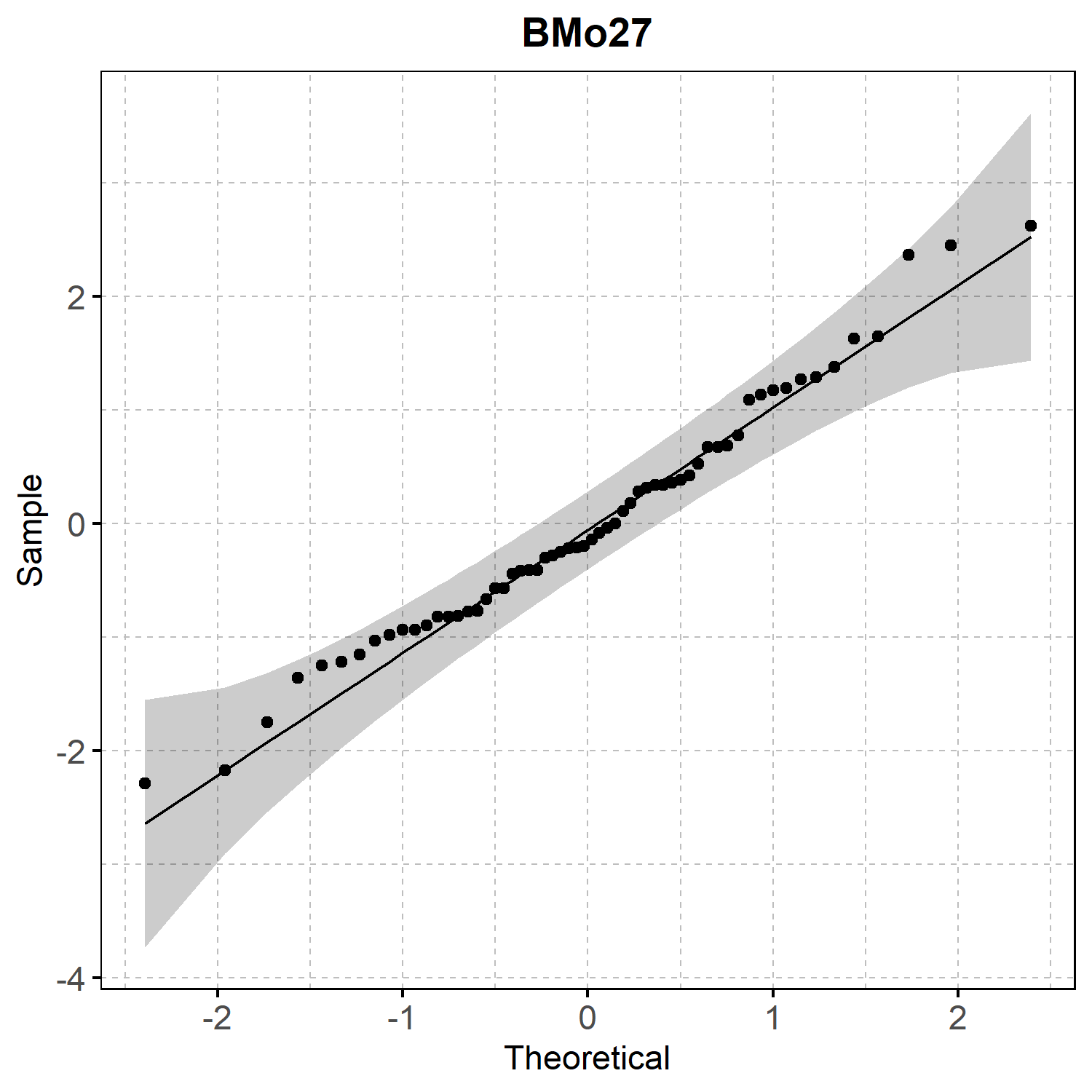

Supplement: Supplementary file 2 — Supplementary Information 2. [file 41598_2023_33504_MOESM2_ESM.zip › BMo027_normality.png]

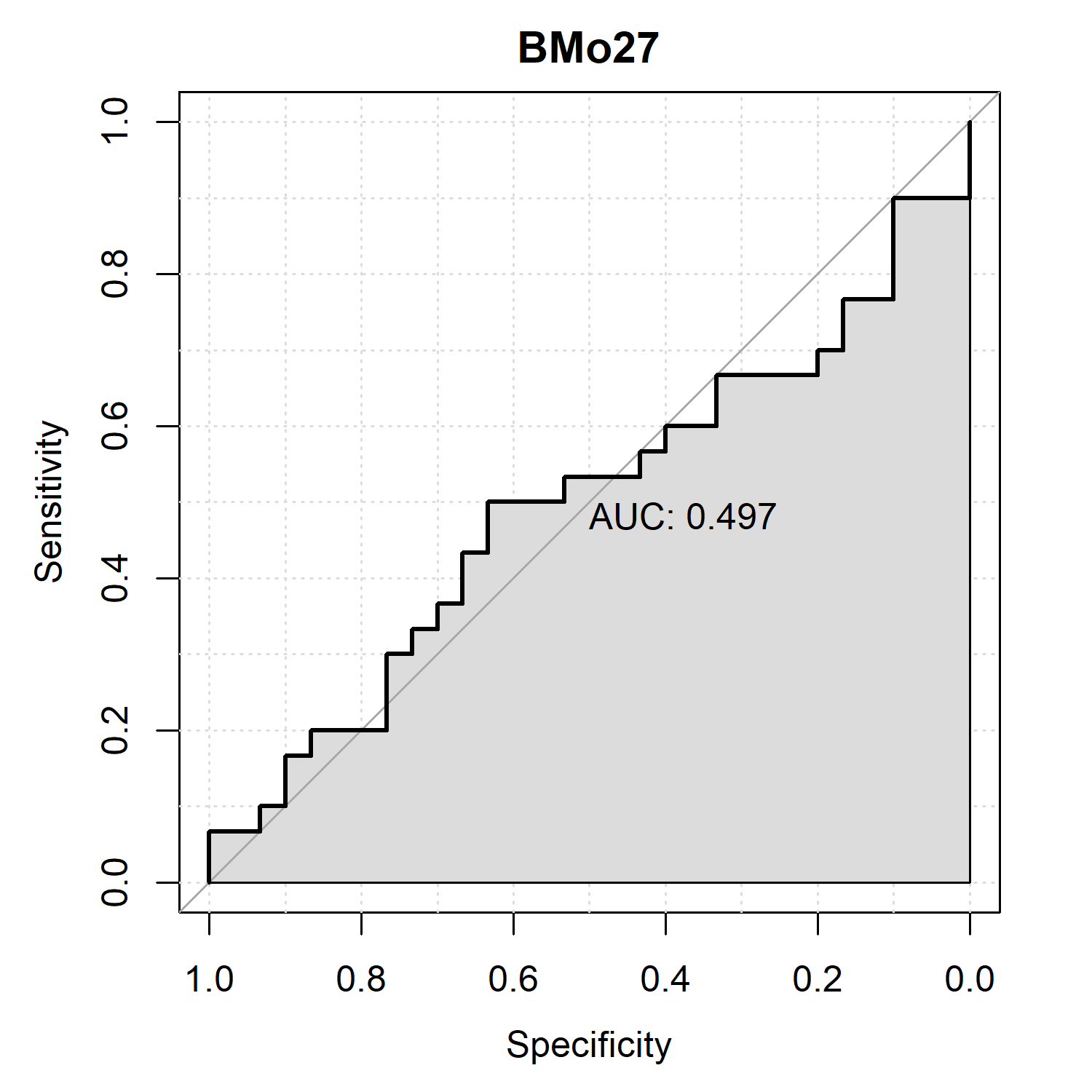

Supplement: Supplementary file 2 — Supplementary Information 2. [file 41598_2023_33504_MOESM2_ESM.zip › BMo027_ROC.png]

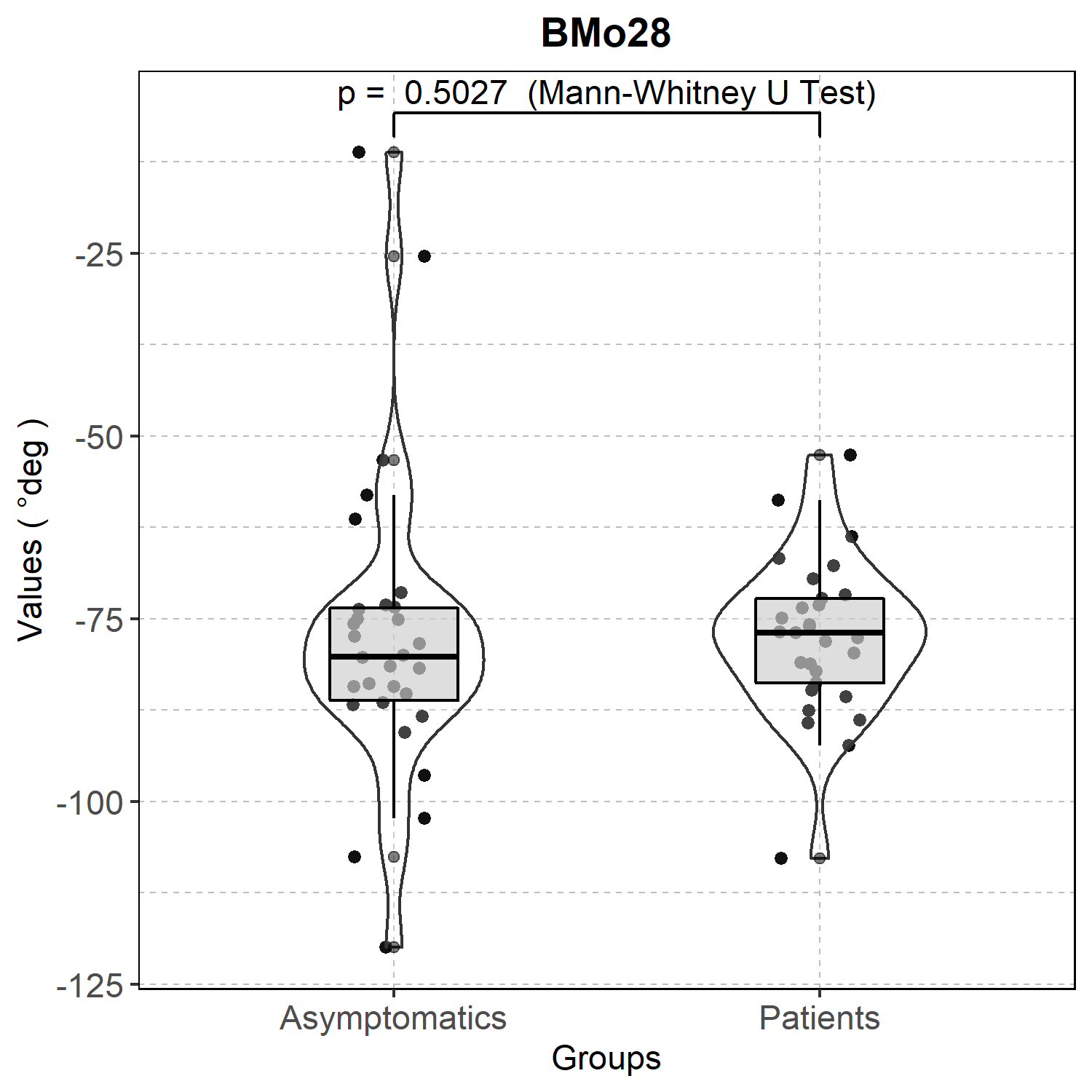

Supplement: Supplementary file 2 — Supplementary Information 2. [file 41598_2023_33504_MOESM2_ESM.zip › BMo028_boxplot.png]

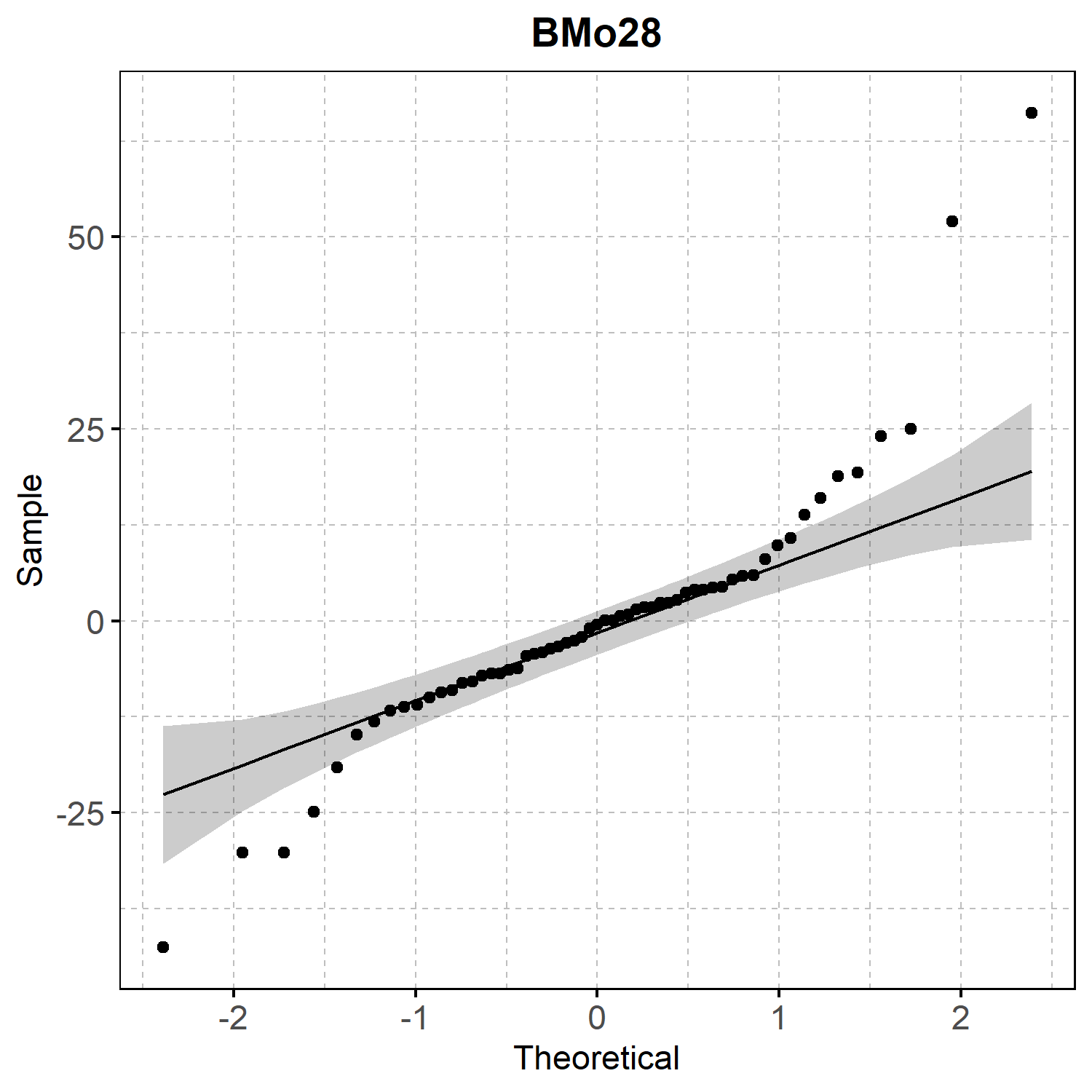

Supplement: Supplementary file 2 — Supplementary Information 2. [file 41598_2023_33504_MOESM2_ESM.zip › BMo028_normality.png]

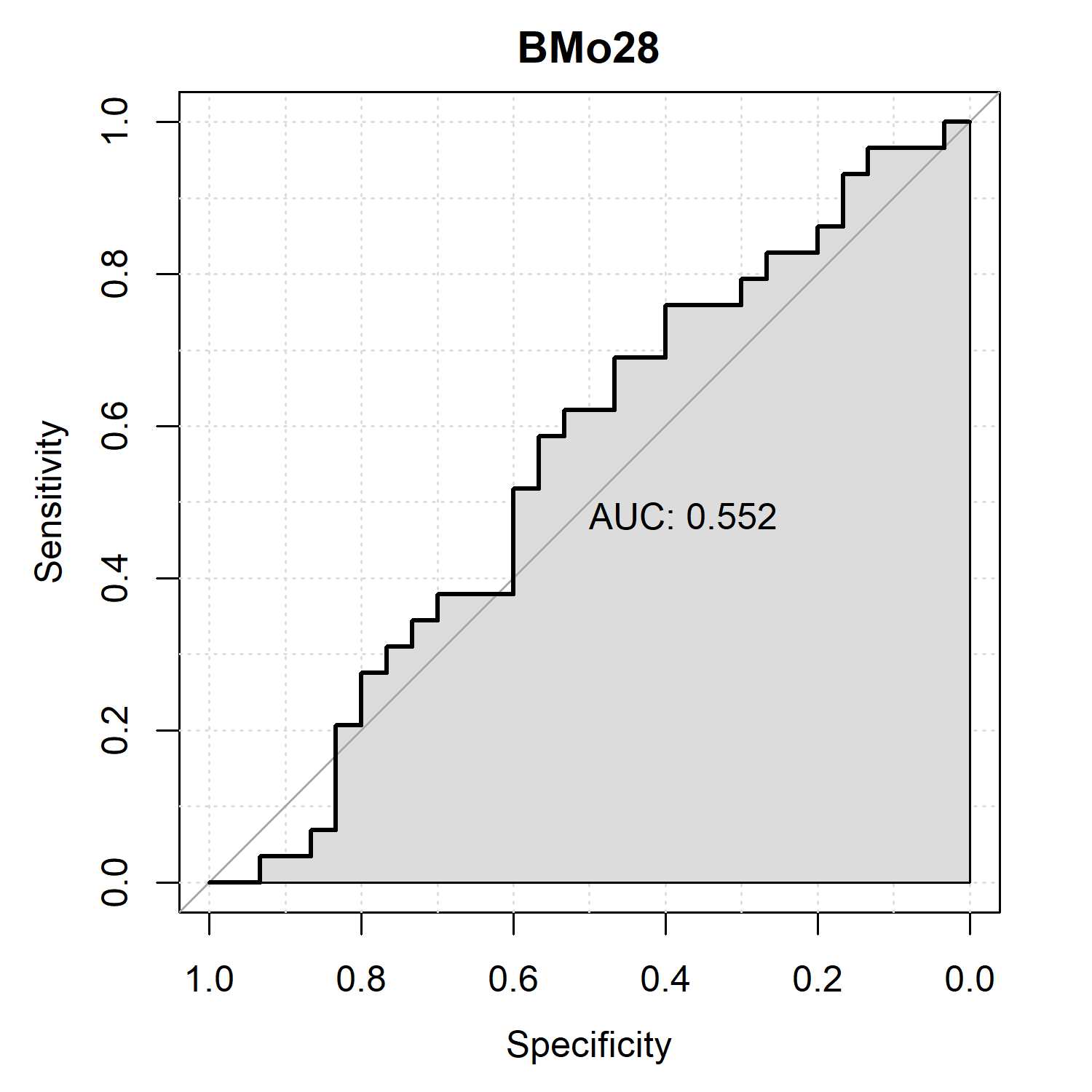

Supplement: Supplementary file 2 — Supplementary Information 2. [file 41598_2023_33504_MOESM2_ESM.zip › BMo028_ROC.png]

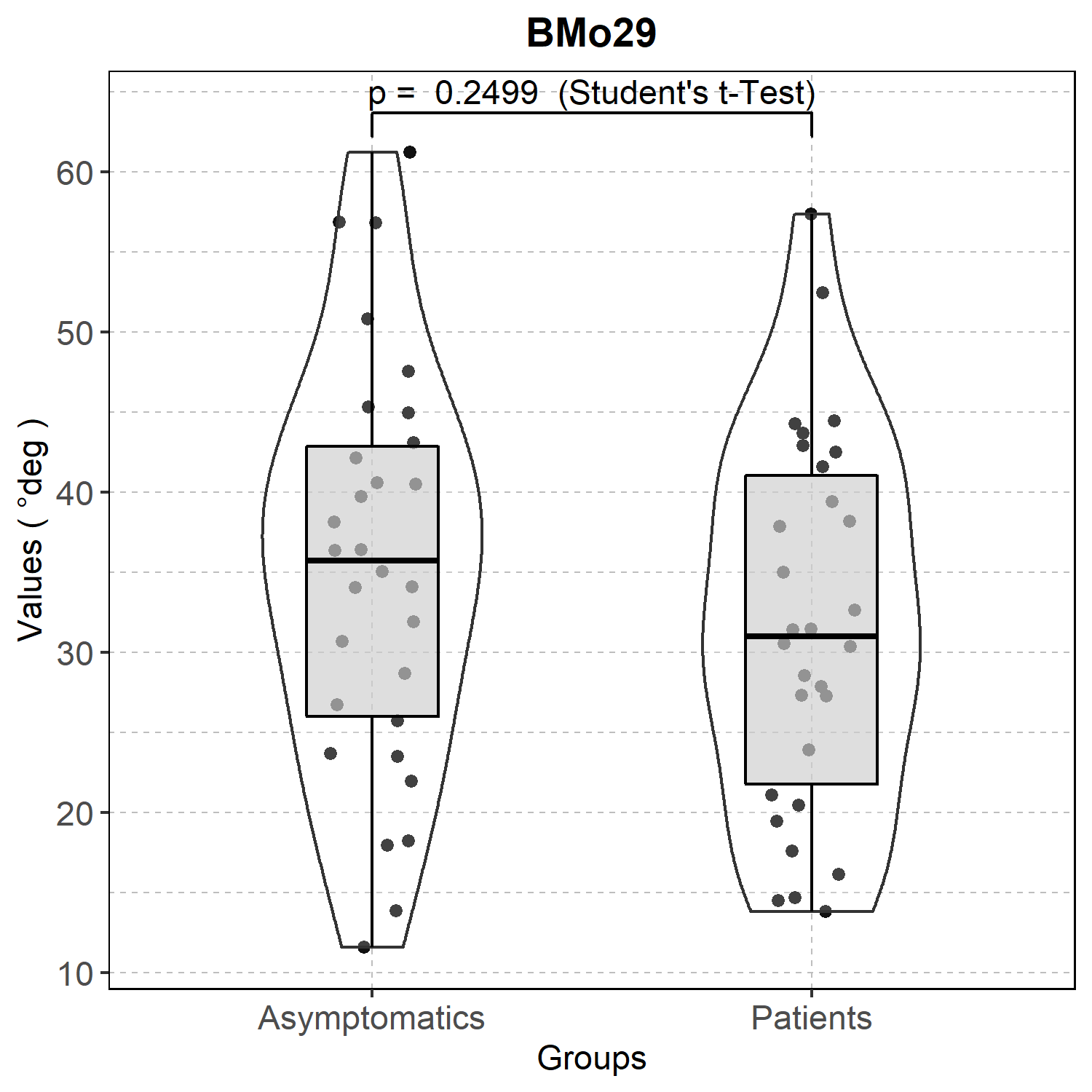

Supplement: Supplementary file 2 — Supplementary Information 2. [file 41598_2023_33504_MOESM2_ESM.zip › BMo029_boxplot.png]

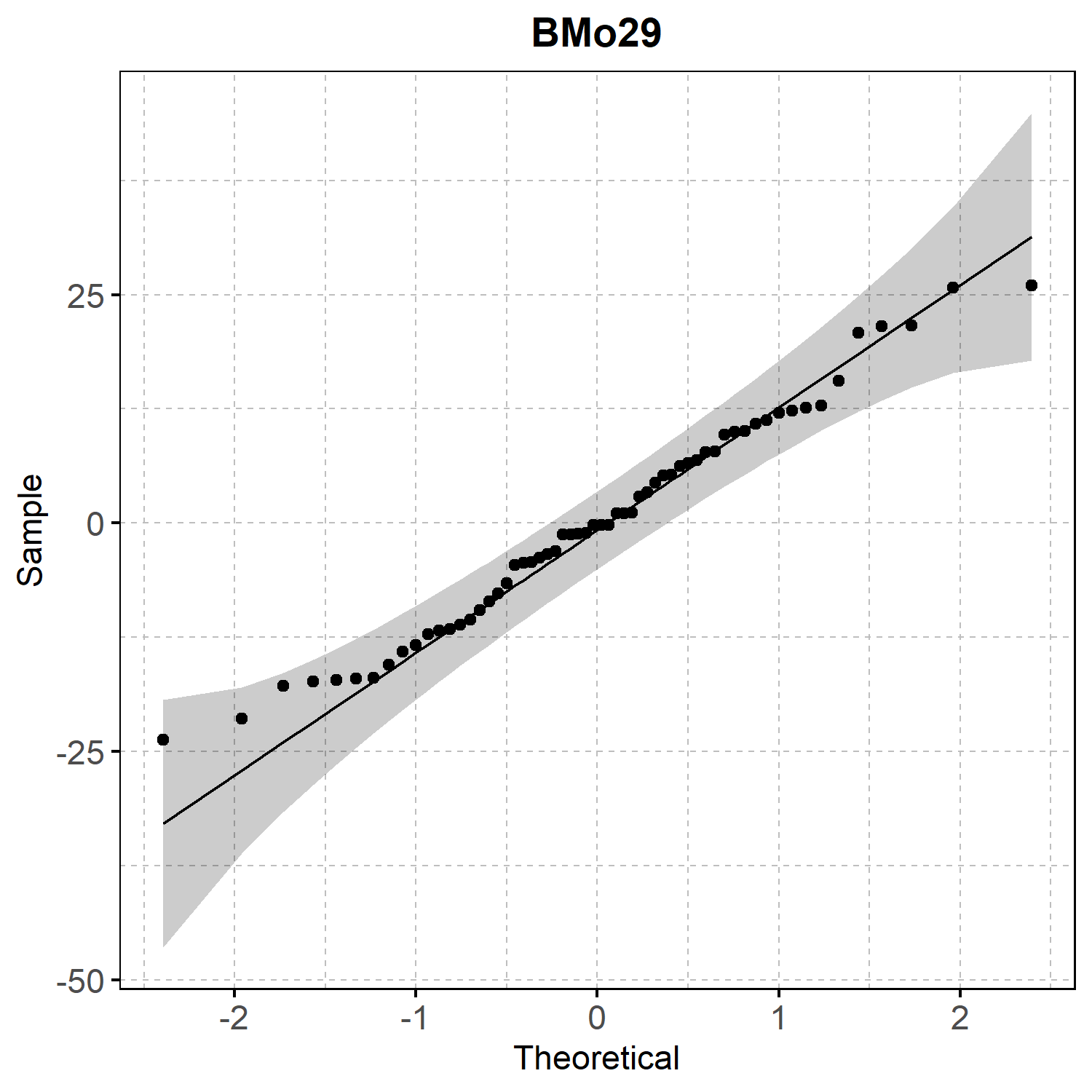

Supplement: Supplementary file 2 — Supplementary Information 2. [file 41598_2023_33504_MOESM2_ESM.zip › BMo029_normality.png]

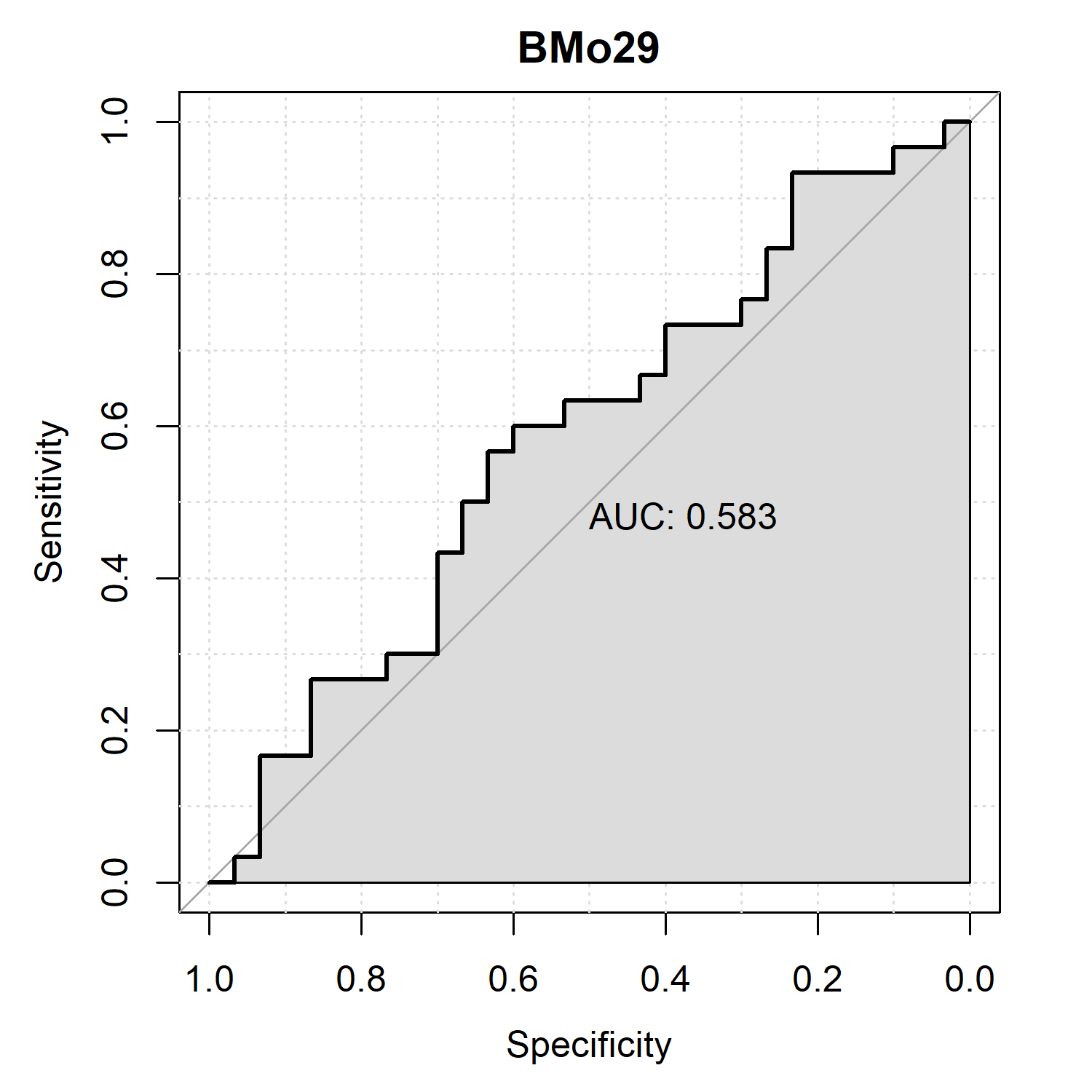

Supplement: Supplementary file 2 — Supplementary Information 2. [file 41598_2023_33504_MOESM2_ESM.zip › BMo029_ROC.png]

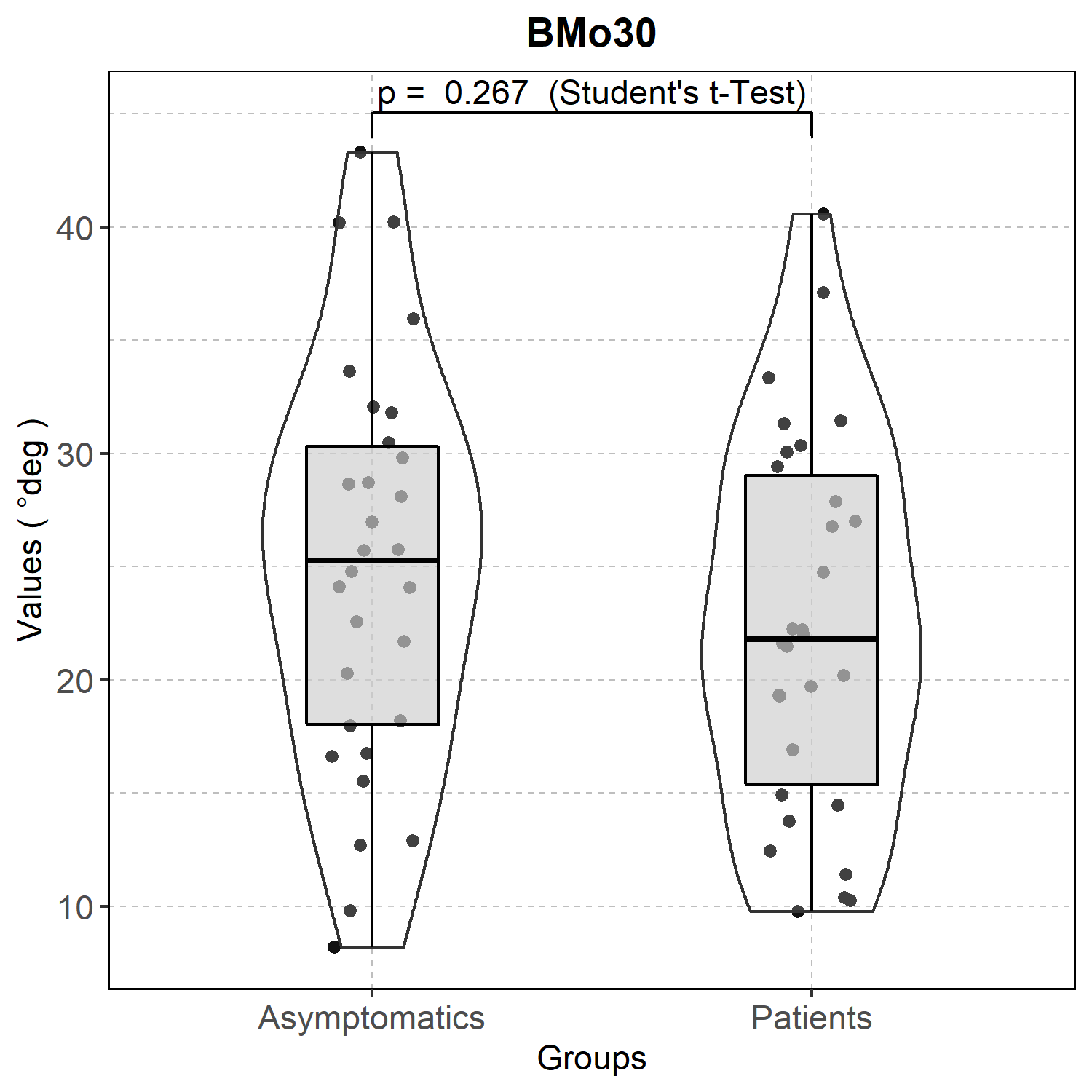

Supplement: Supplementary file 2 — Supplementary Information 2. [file 41598_2023_33504_MOESM2_ESM.zip › BMo030_boxplot.png]

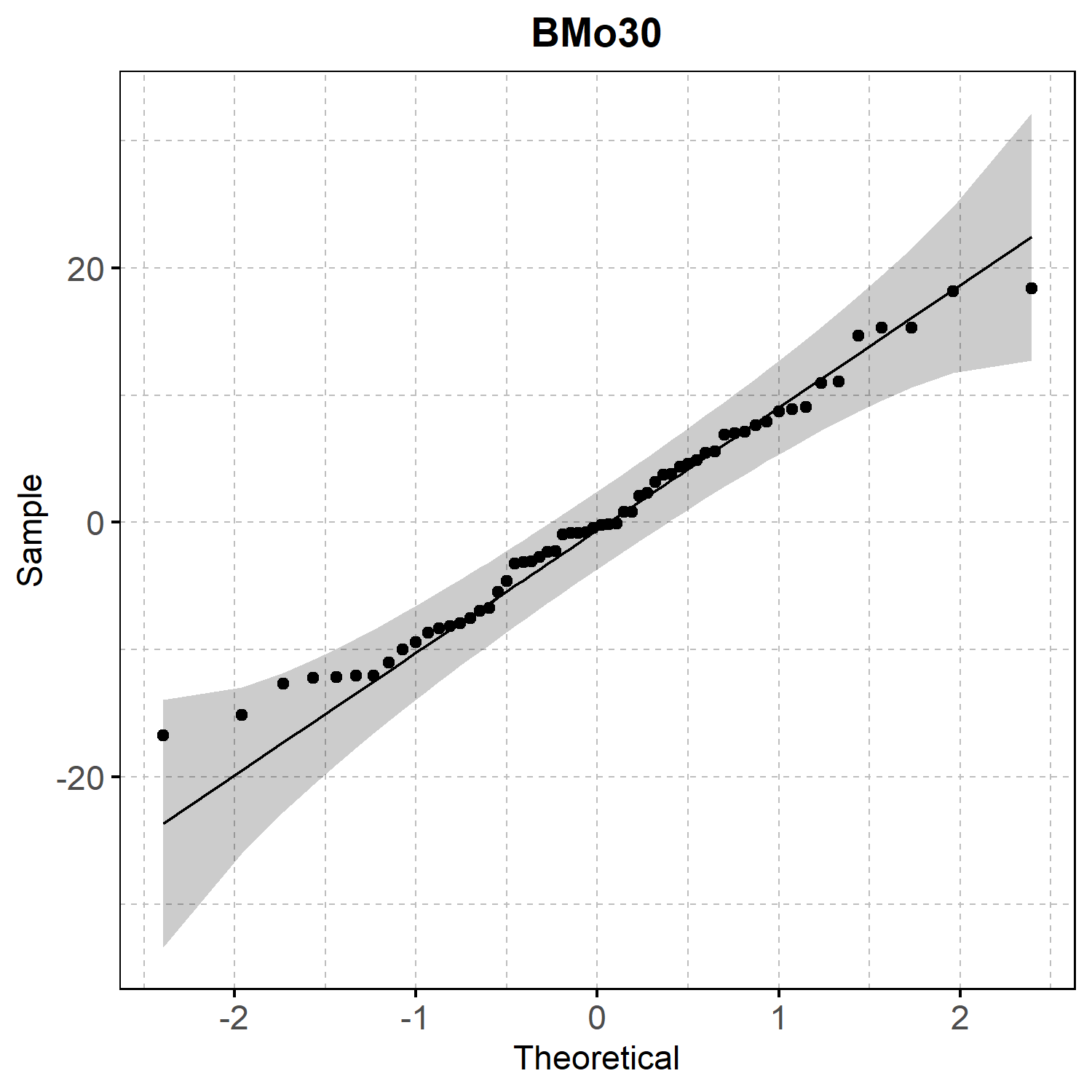

Supplement: Supplementary file 2 — Supplementary Information 2. [file 41598_2023_33504_MOESM2_ESM.zip › BMo030_normality.png]

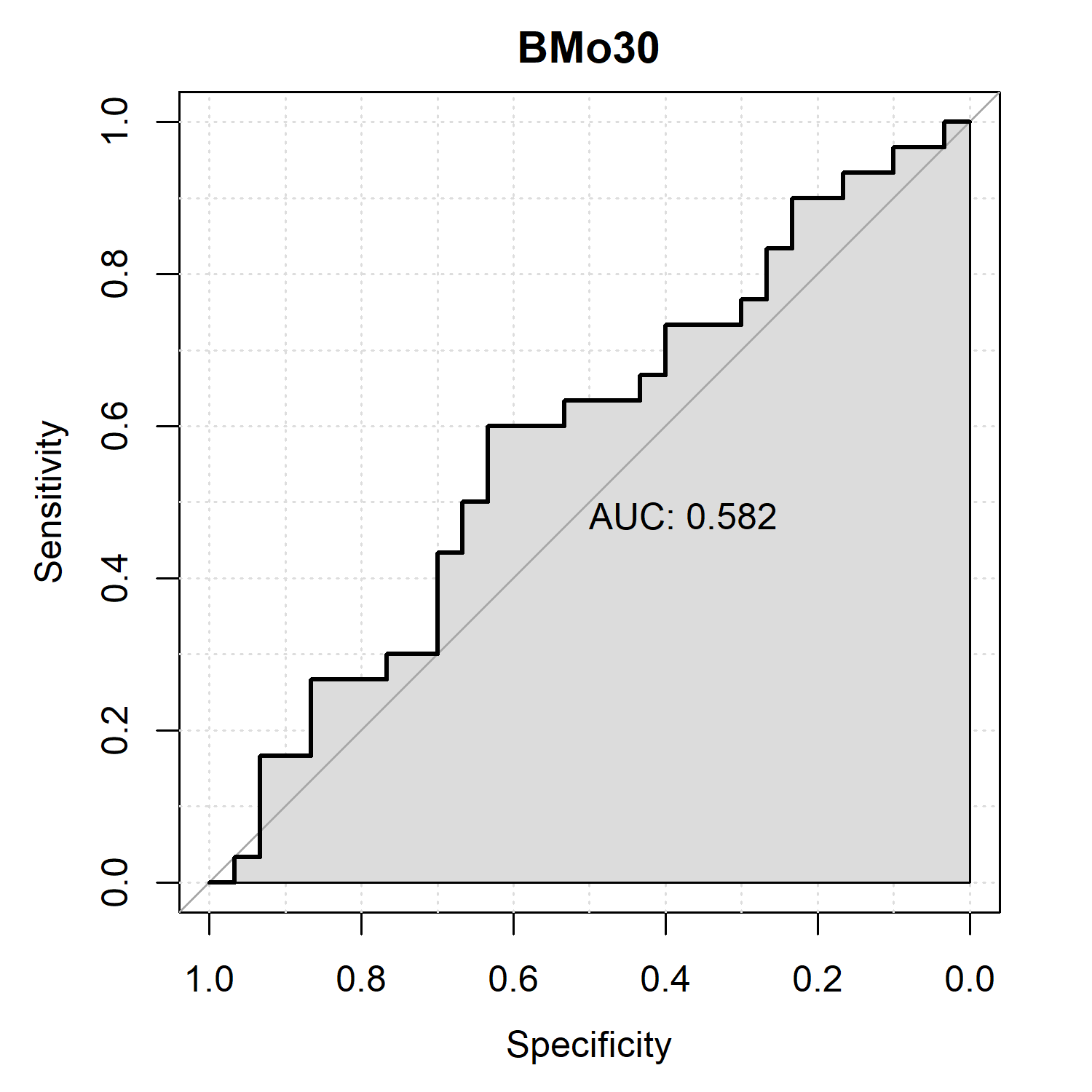

Supplement: Supplementary file 2 — Supplementary Information 2. [file 41598_2023_33504_MOESM2_ESM.zip › BMo030_ROC.png]

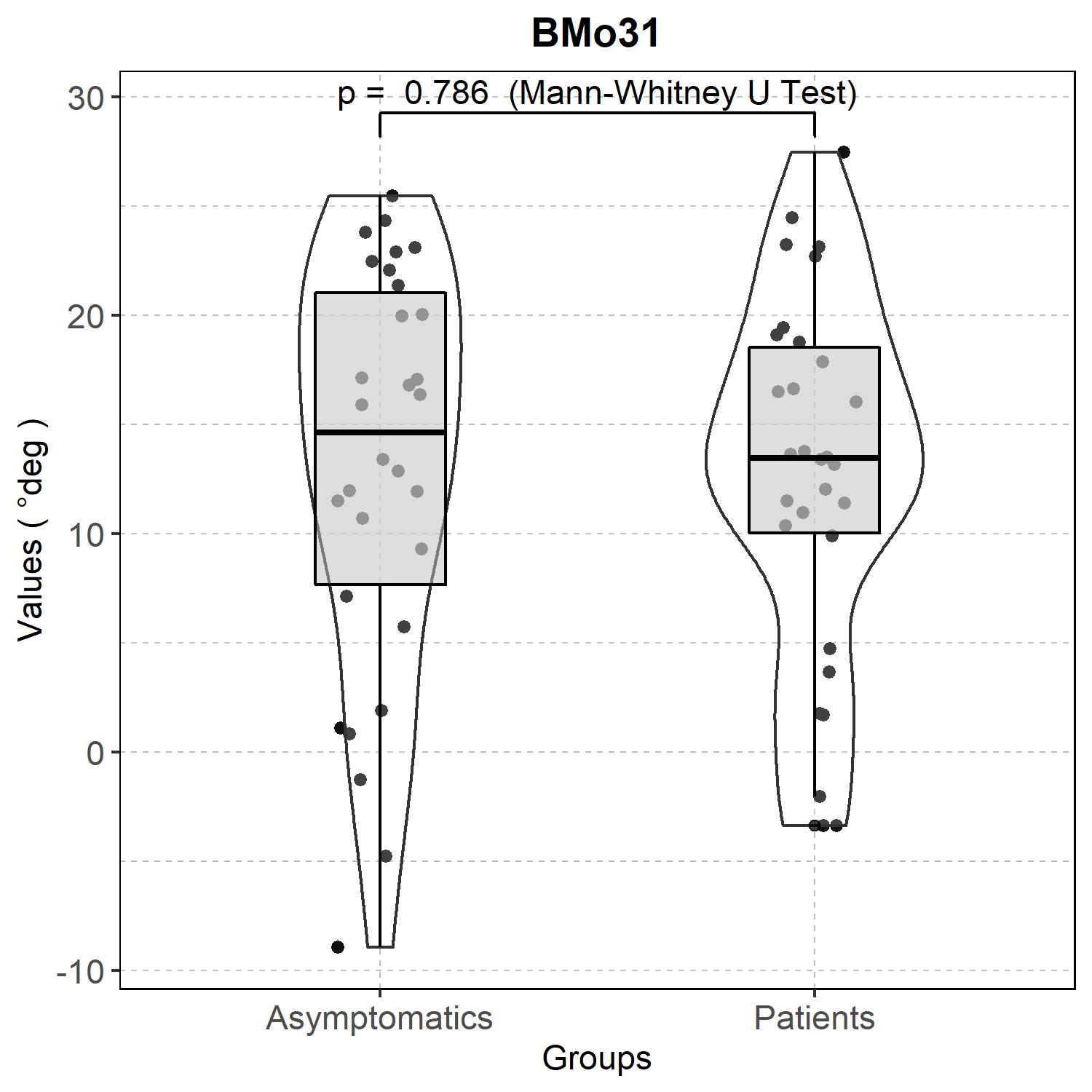

Supplement: Supplementary file 2 — Supplementary Information 2. [file 41598_2023_33504_MOESM2_ESM.zip › BMo031_boxplot.png]

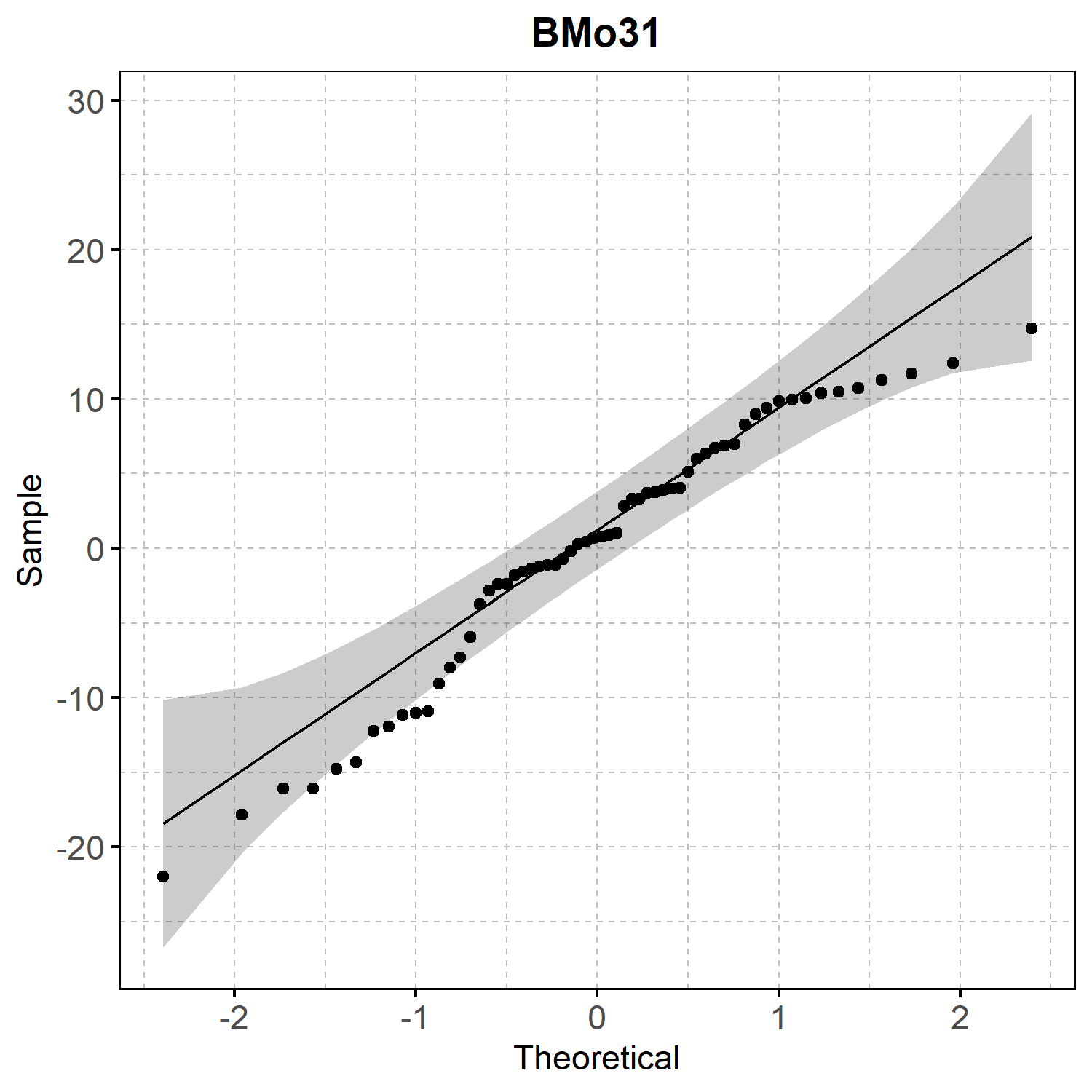

Supplement: Supplementary file 2 — Supplementary Information 2. [file 41598_2023_33504_MOESM2_ESM.zip › BMo031_normality.png]

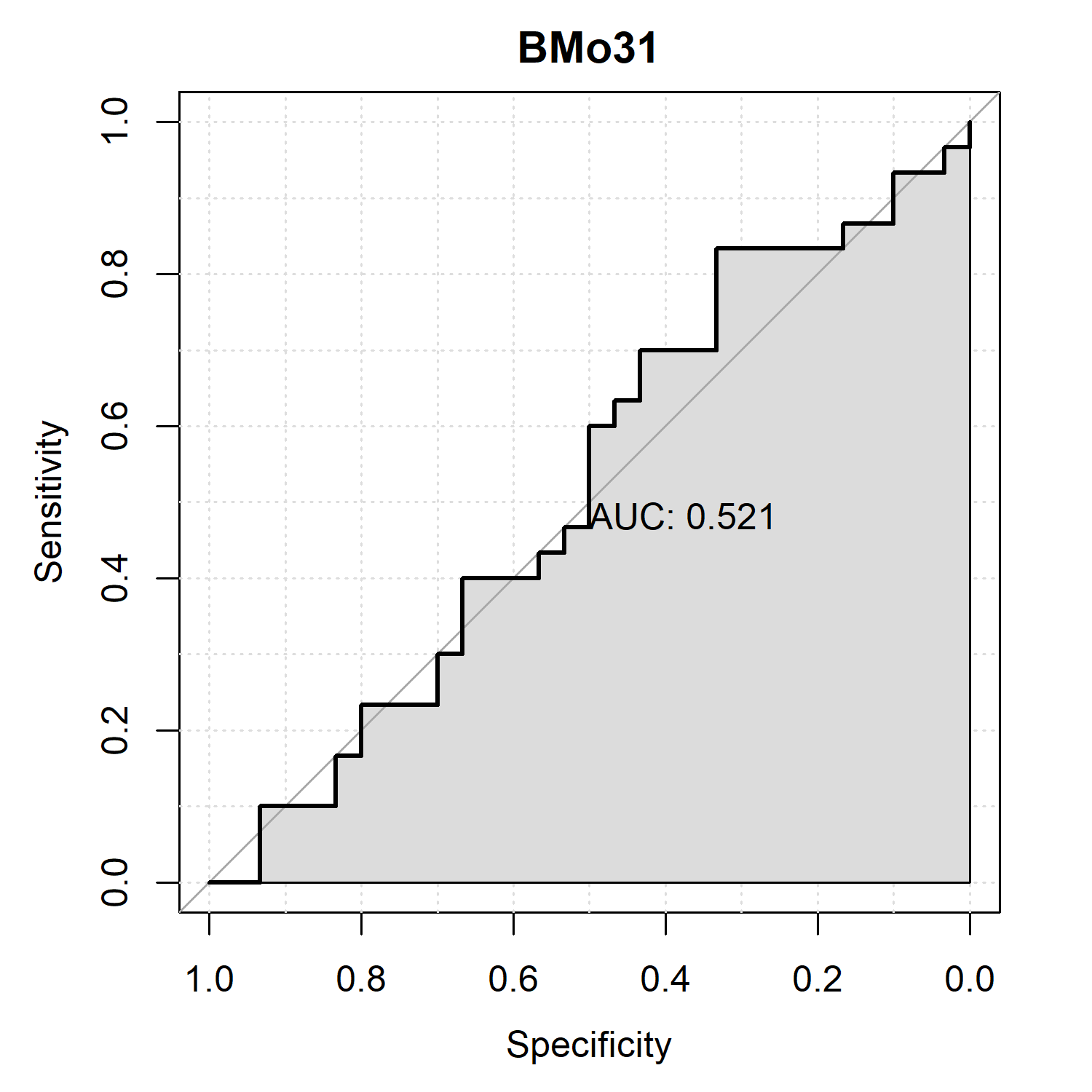

Supplement: Supplementary file 2 — Supplementary Information 2. [file 41598_2023_33504_MOESM2_ESM.zip › BMo031_ROC.png]

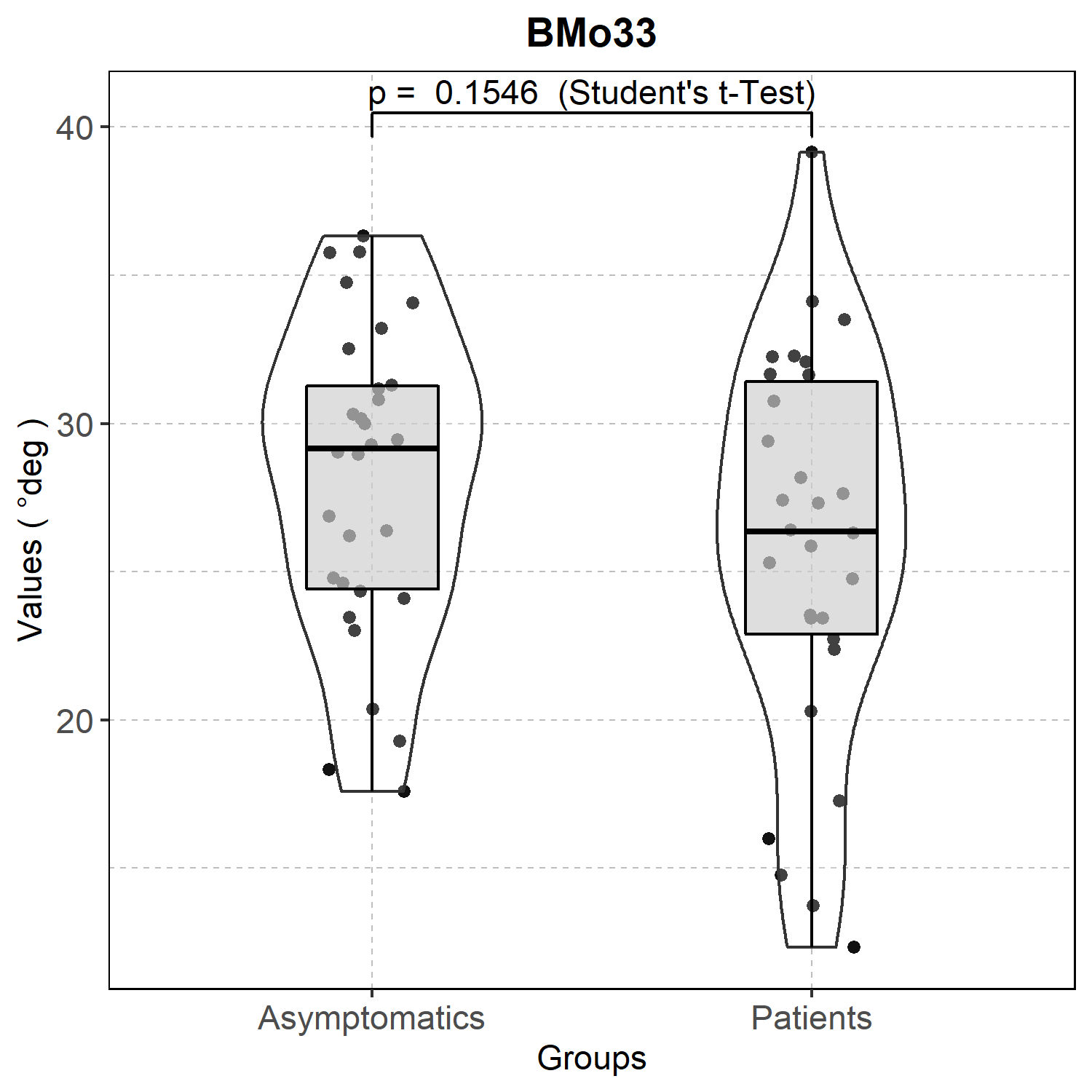

Supplement: Supplementary file 2 — Supplementary Information 2. [file 41598_2023_33504_MOESM2_ESM.zip › BMo033_boxplot.png]

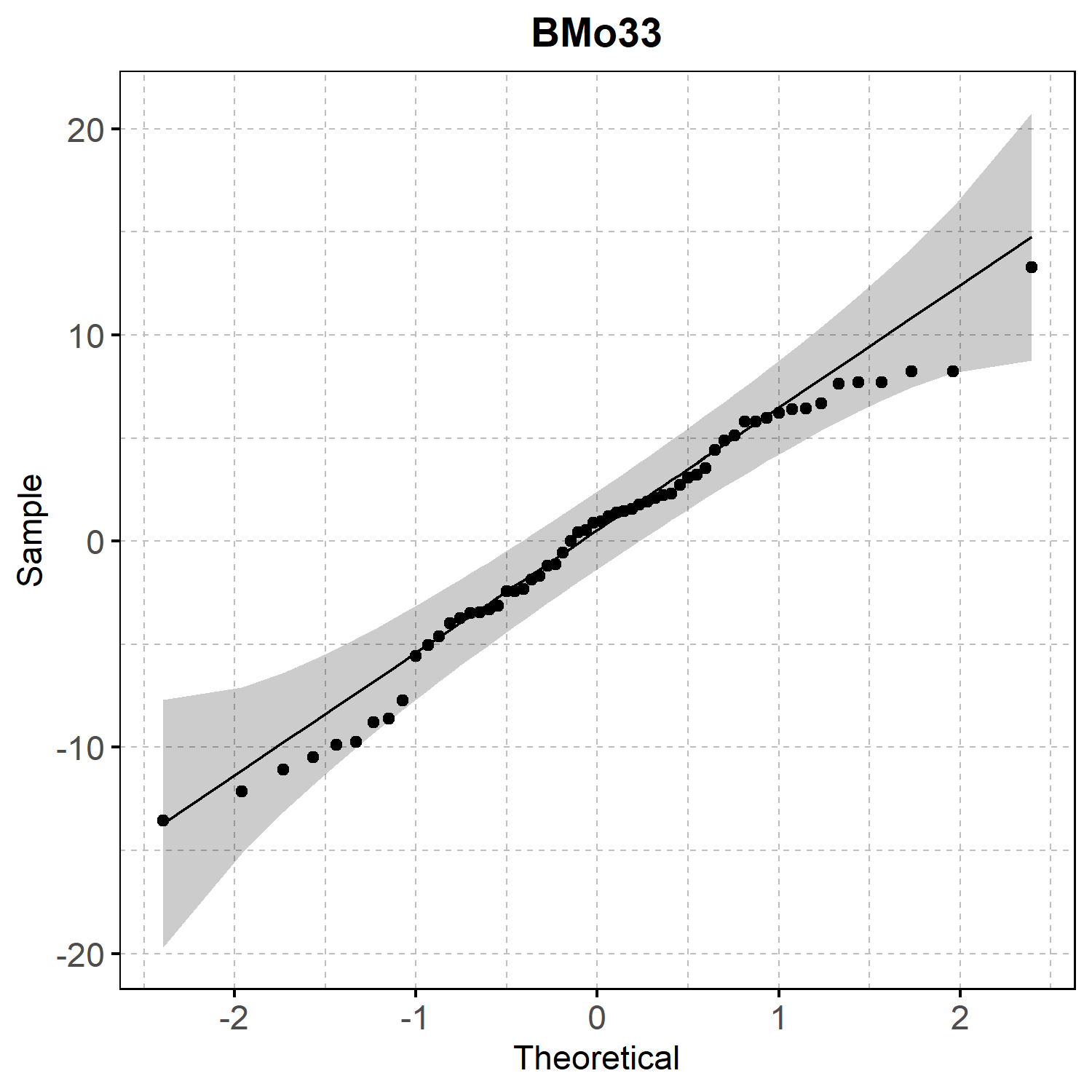

Supplement: Supplementary file 2 — Supplementary Information 2. [file 41598_2023_33504_MOESM2_ESM.zip › BMo033_normality.png]

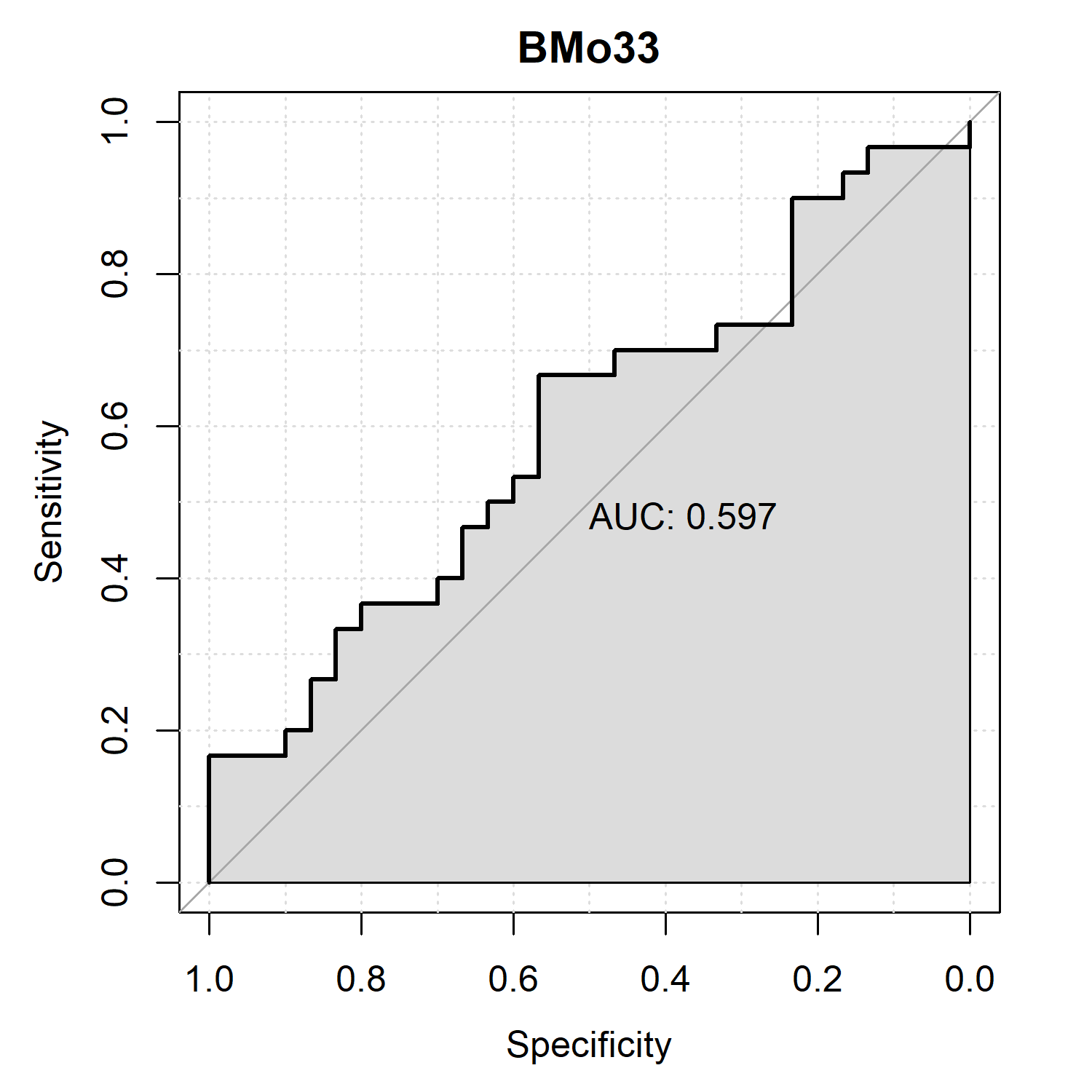

Supplement: Supplementary file 2 — Supplementary Information 2. [file 41598_2023_33504_MOESM2_ESM.zip › BMo033_ROC.png]

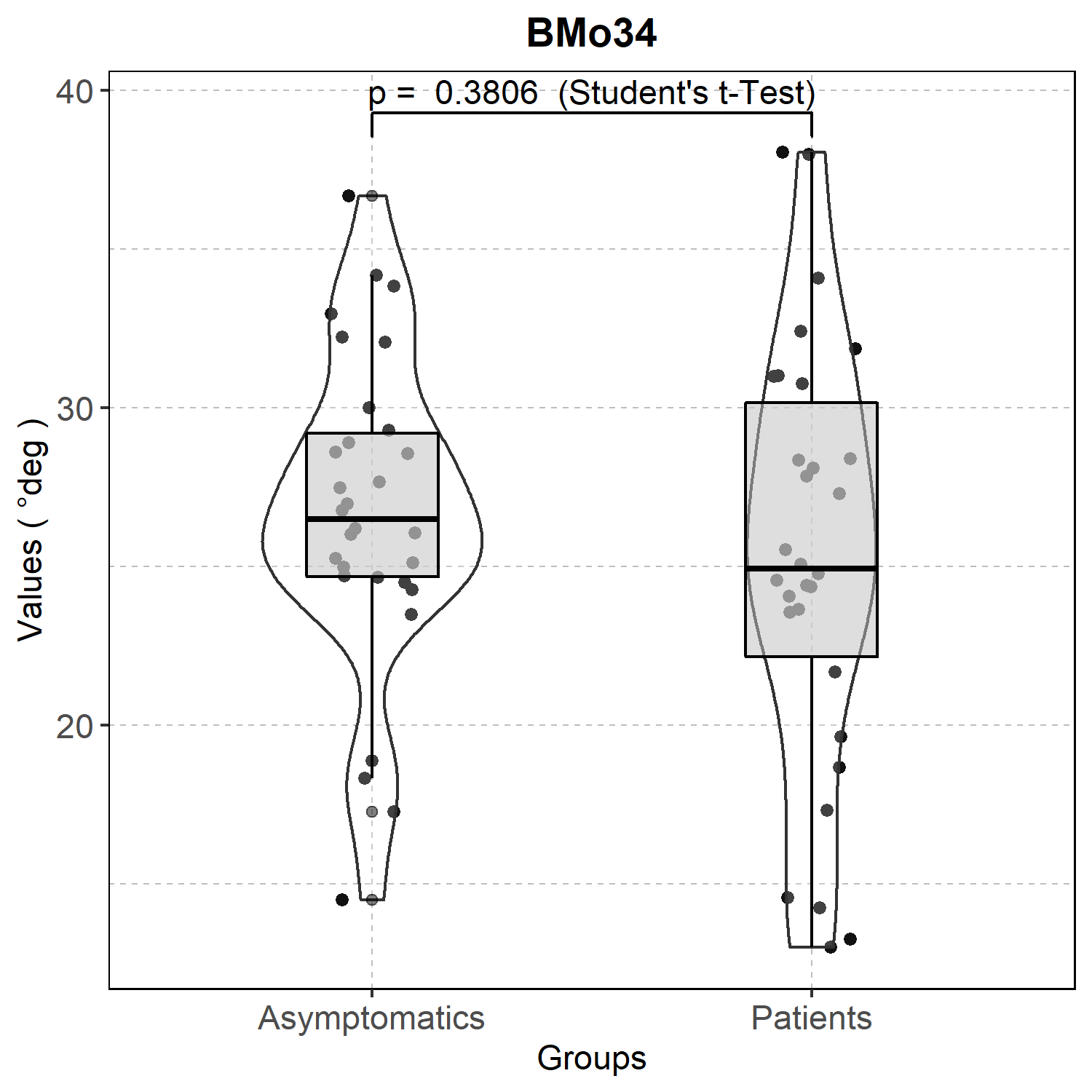

Supplement: Supplementary file 2 — Supplementary Information 2. [file 41598_2023_33504_MOESM2_ESM.zip › BMo034_boxplot.png]

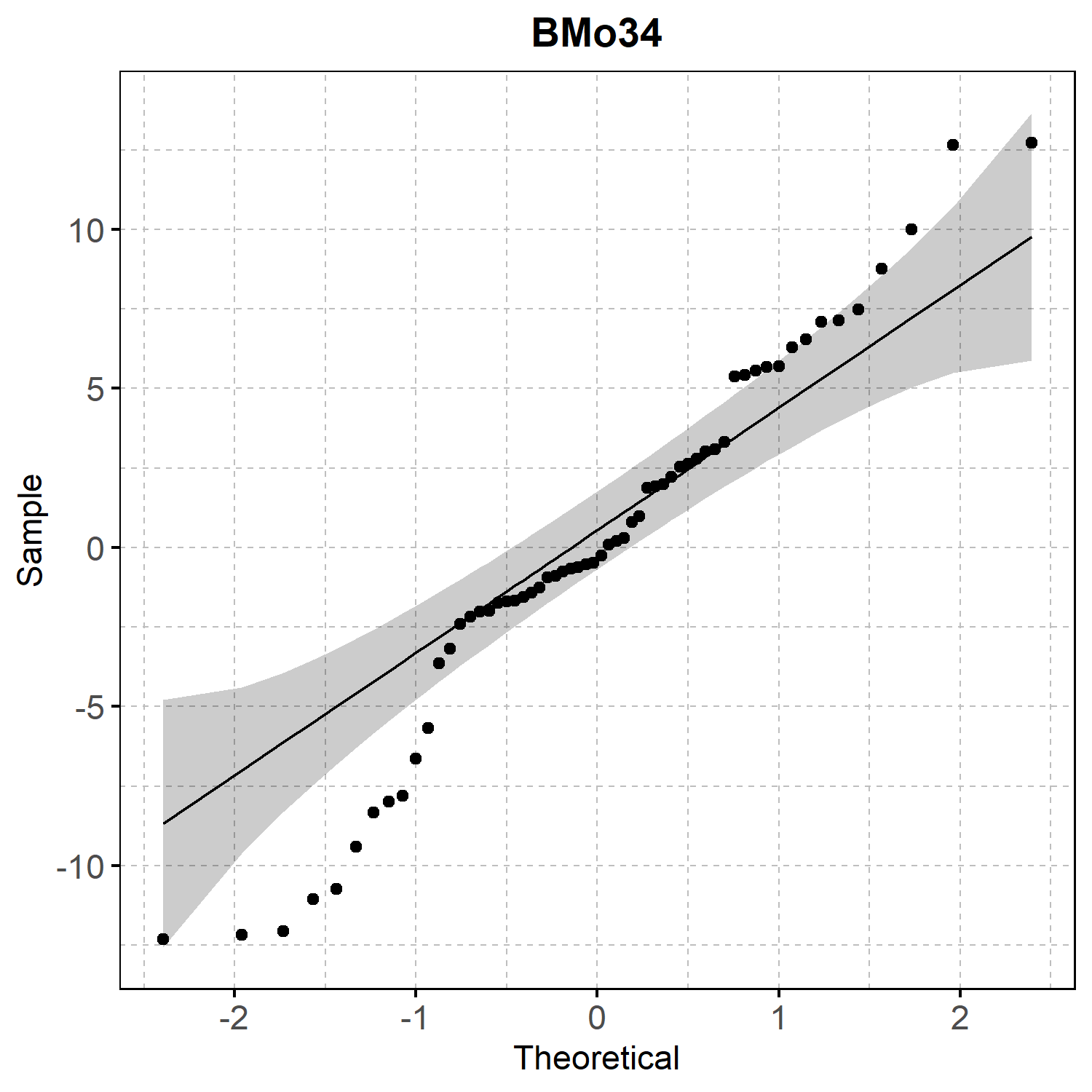

Supplement: Supplementary file 2 — Supplementary Information 2. [file 41598_2023_33504_MOESM2_ESM.zip › BMo034_normality.png]

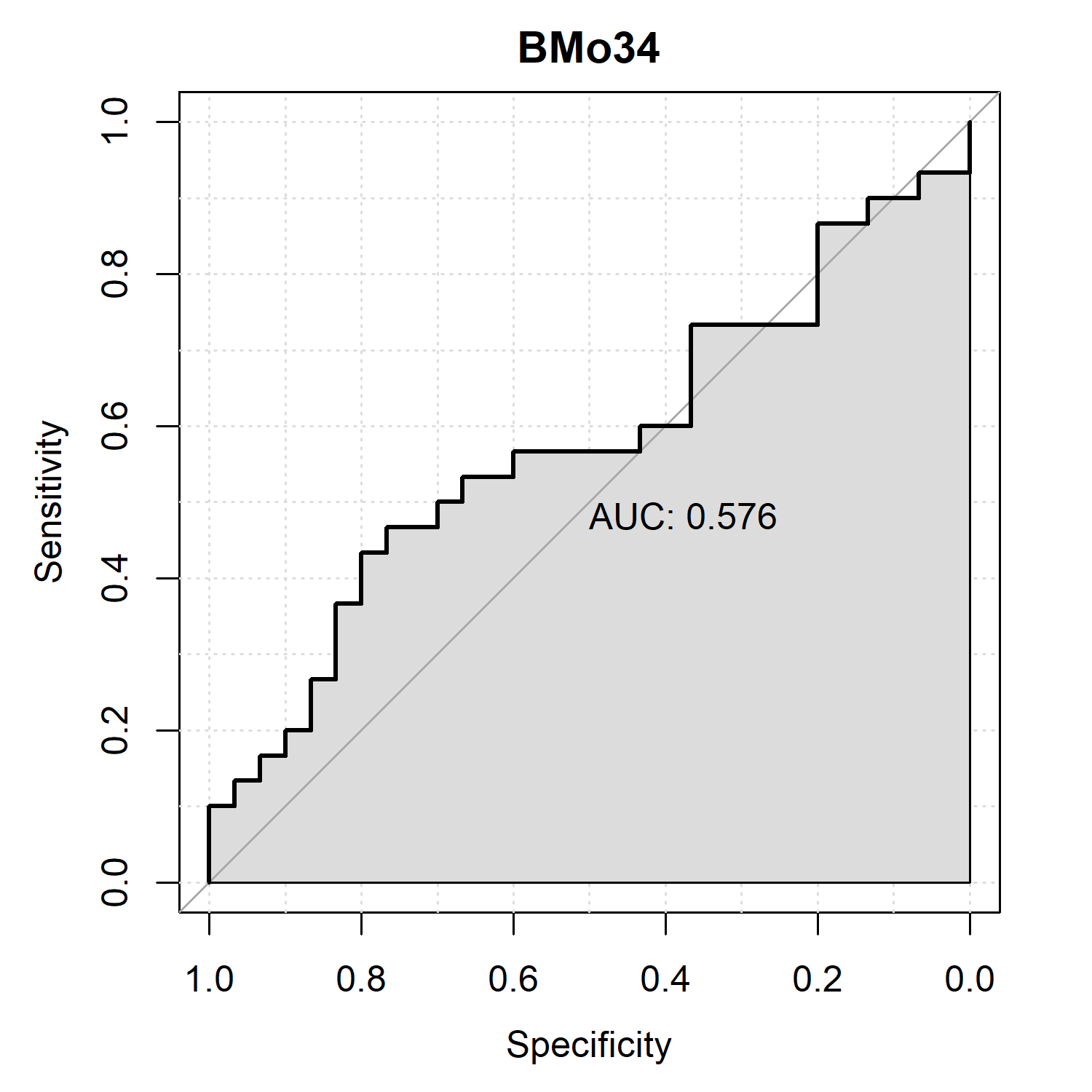

Supplement: Supplementary file 2 — Supplementary Information 2. [file 41598_2023_33504_MOESM2_ESM.zip › BMo034_ROC.png]

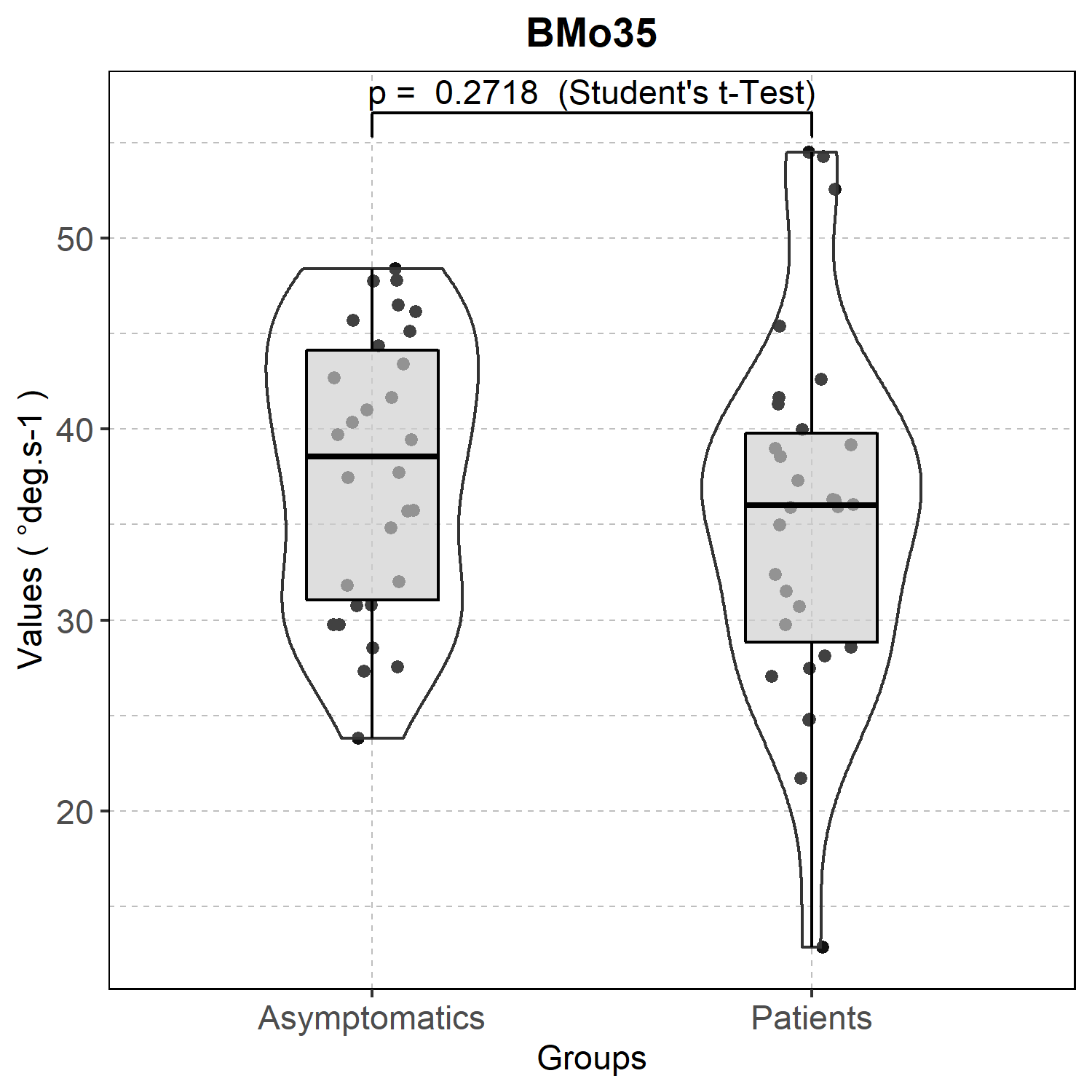

Supplement: Supplementary file 2 — Supplementary Information 2. [file 41598_2023_33504_MOESM2_ESM.zip › BMo035_boxplot.png]

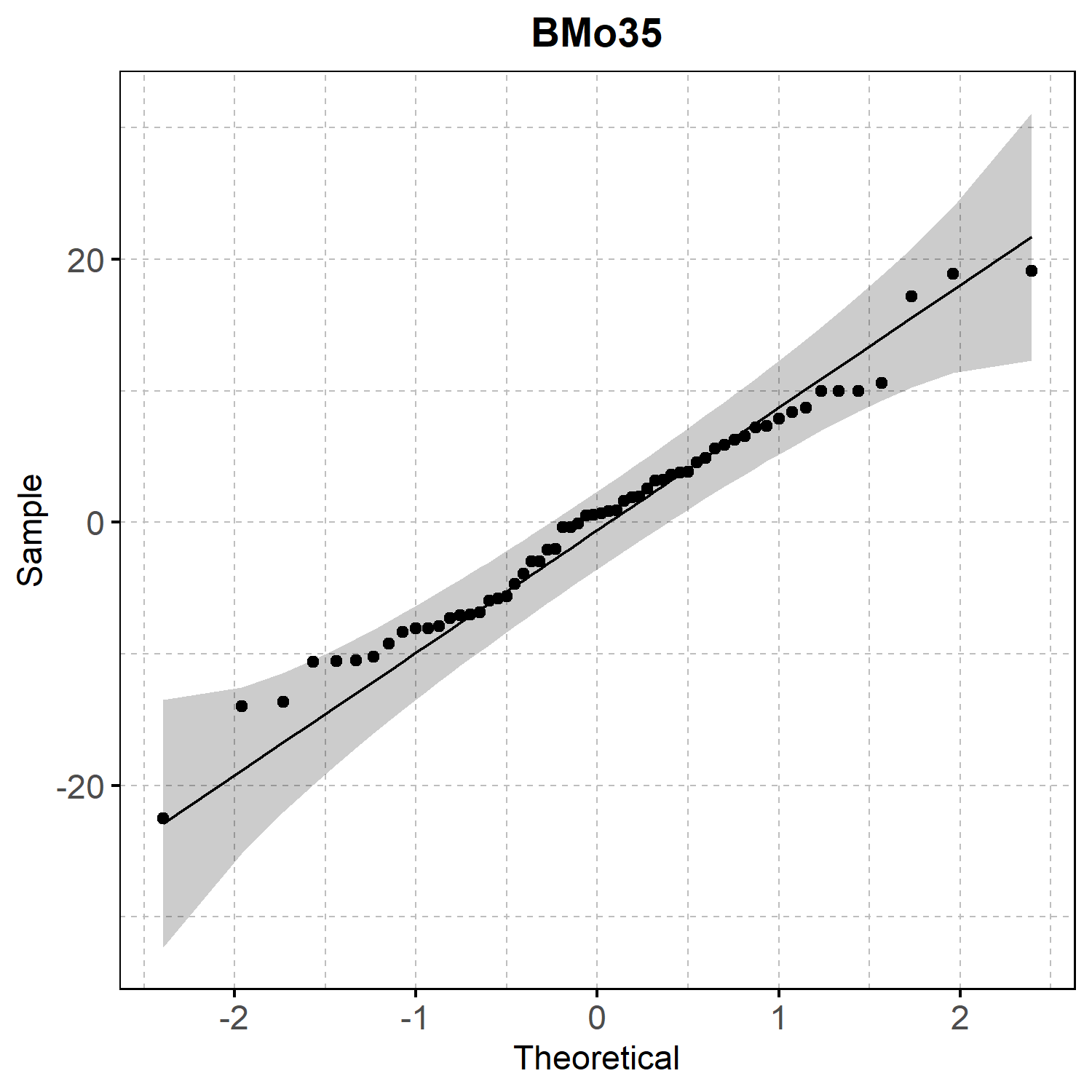

Supplement: Supplementary file 2 — Supplementary Information 2. [file 41598_2023_33504_MOESM2_ESM.zip › BMo035_normality.png]

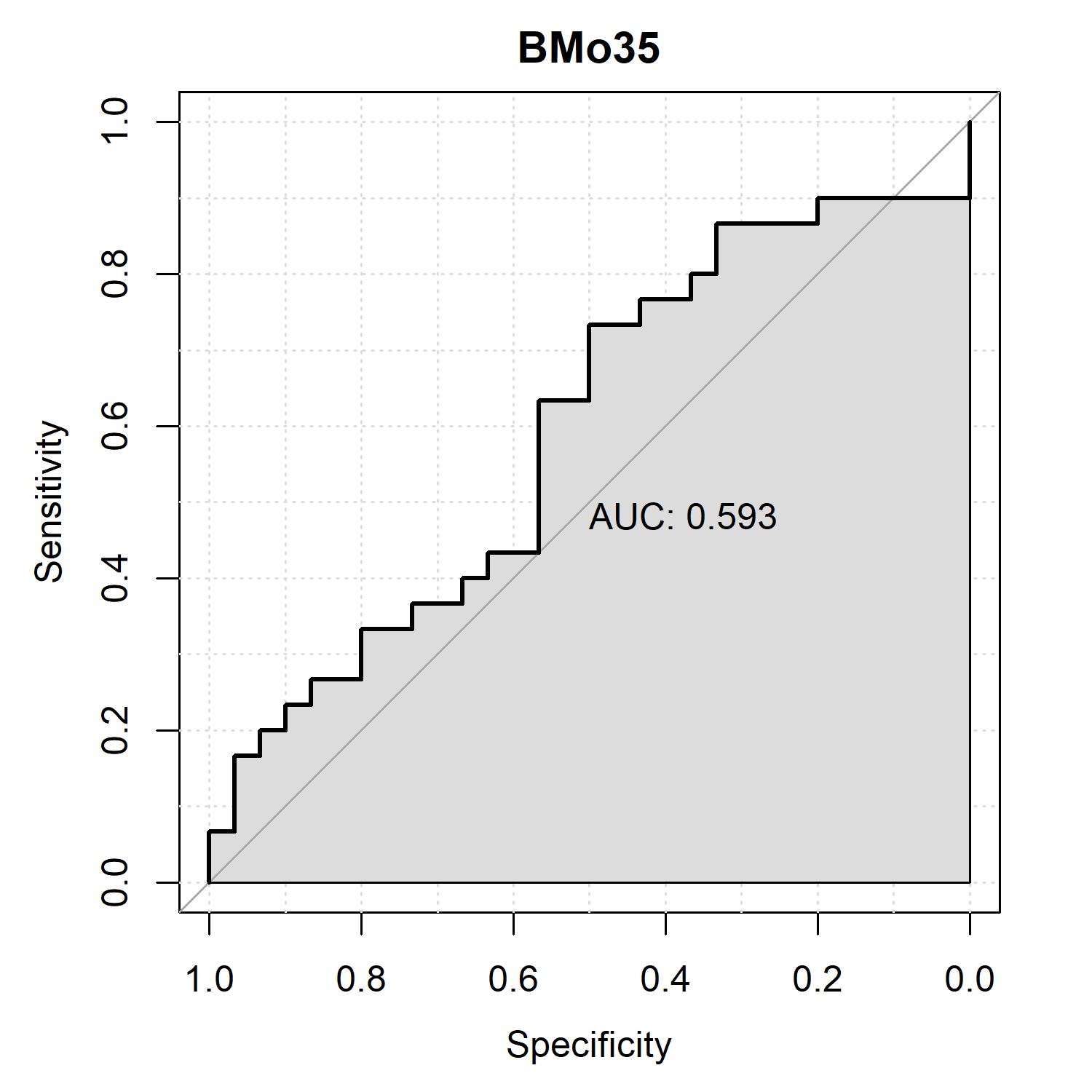

Supplement: Supplementary file 2 — Supplementary Information 2. [file 41598_2023_33504_MOESM2_ESM.zip › BMo035_ROC.png]

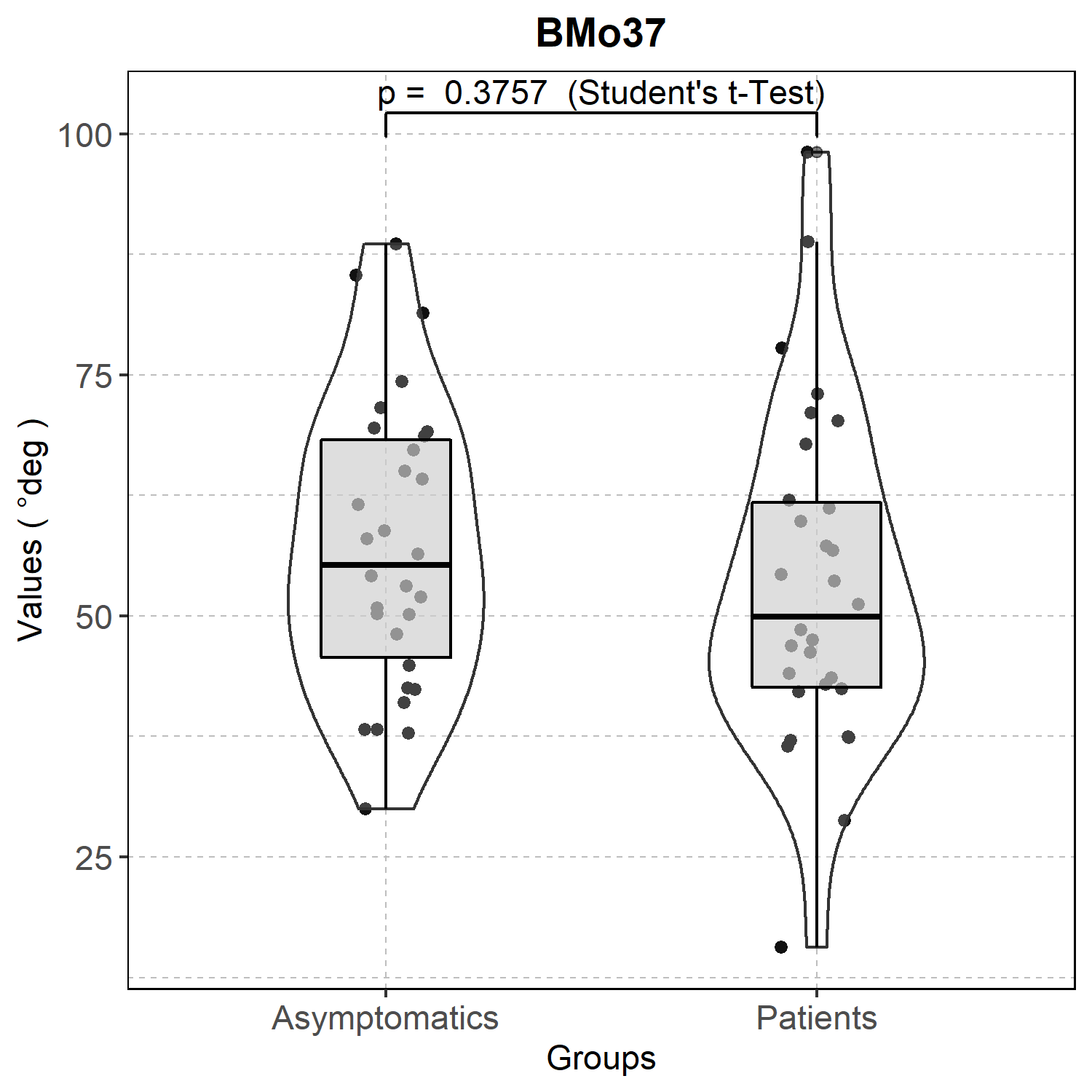

Supplement: Supplementary file 2 — Supplementary Information 2. [file 41598_2023_33504_MOESM2_ESM.zip › BMo037_boxplot.png]

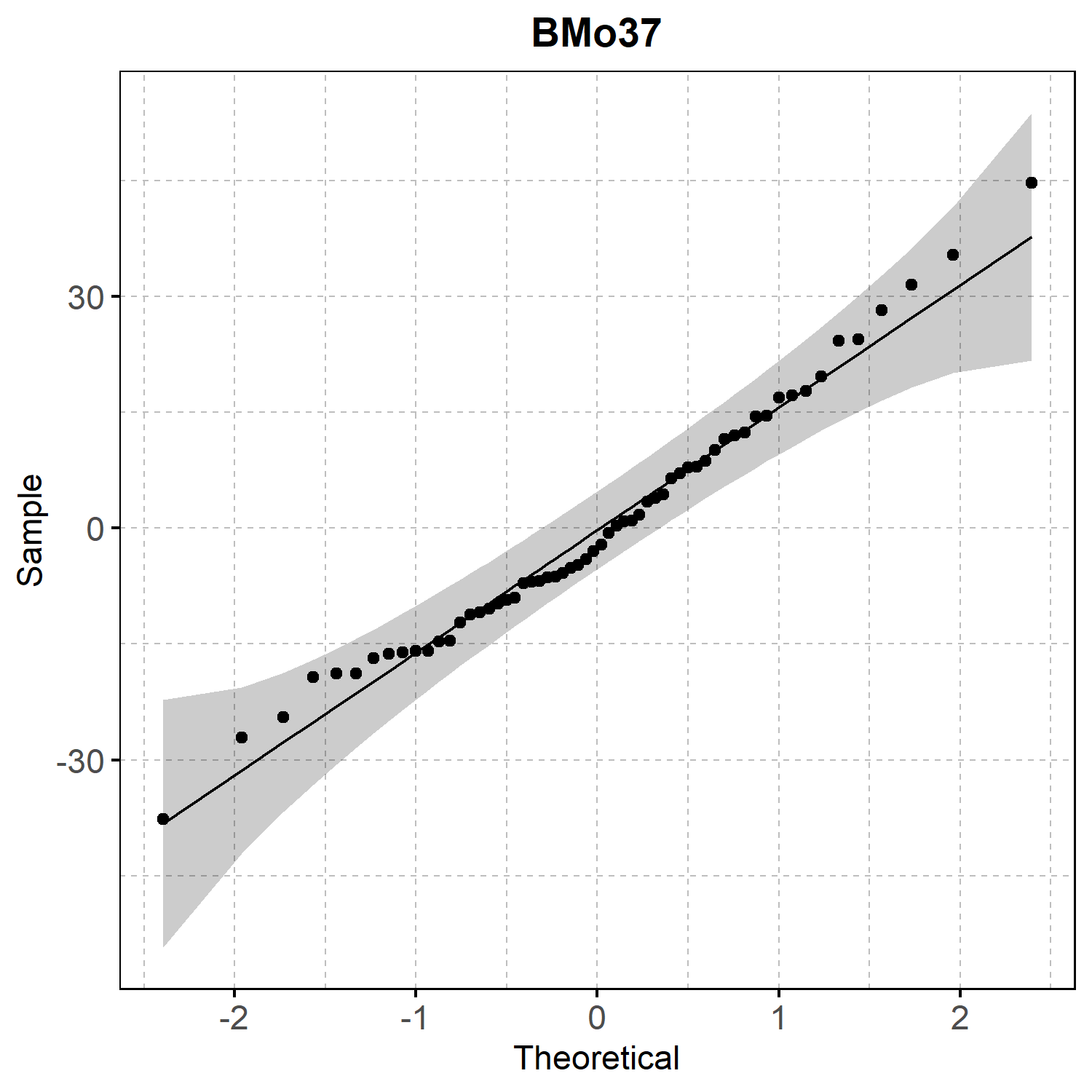

Supplement: Supplementary file 2 — Supplementary Information 2. [file 41598_2023_33504_MOESM2_ESM.zip › BMo037_normality.png]

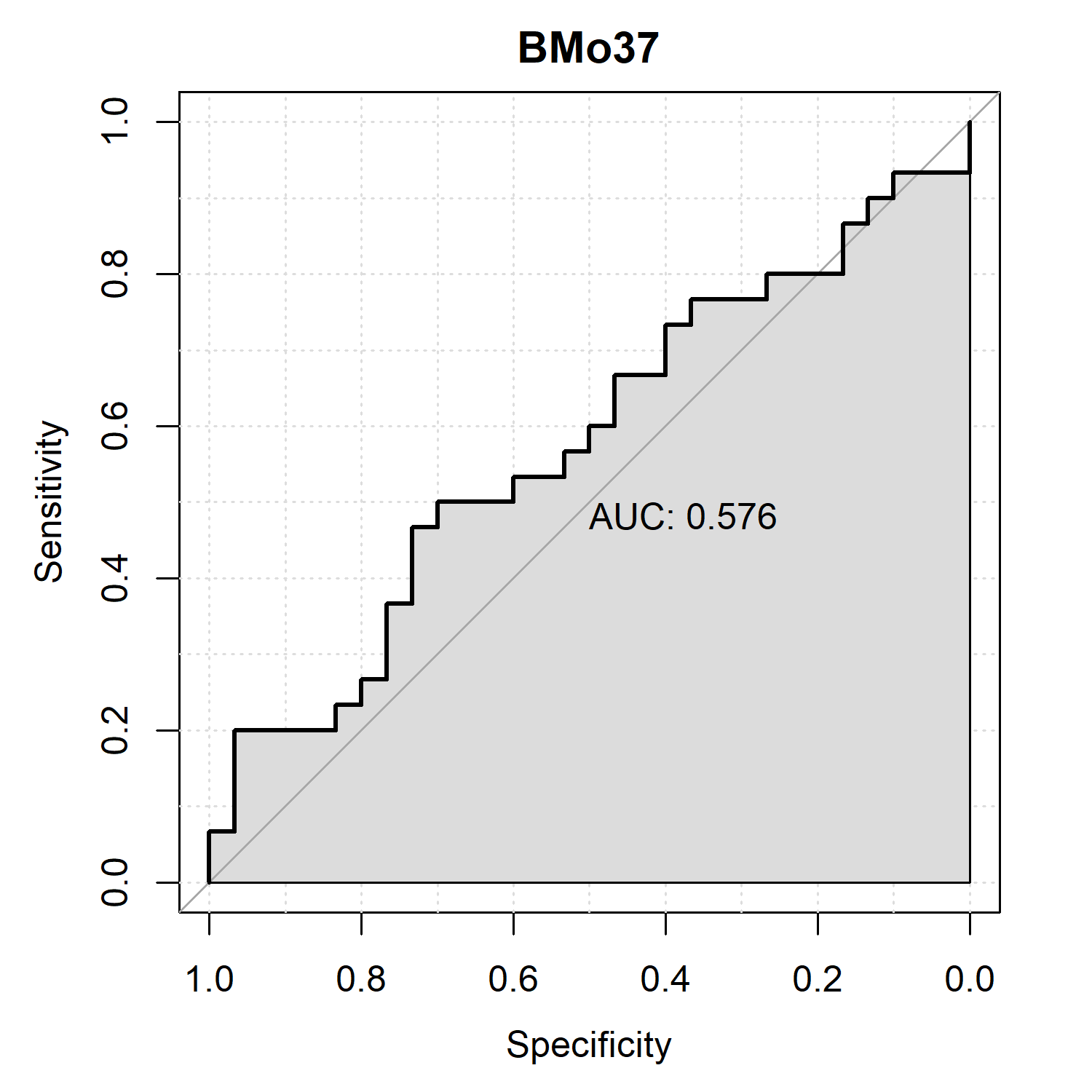

Supplement: Supplementary file 2 — Supplementary Information 2. [file 41598_2023_33504_MOESM2_ESM.zip › BMo037_ROC.png]

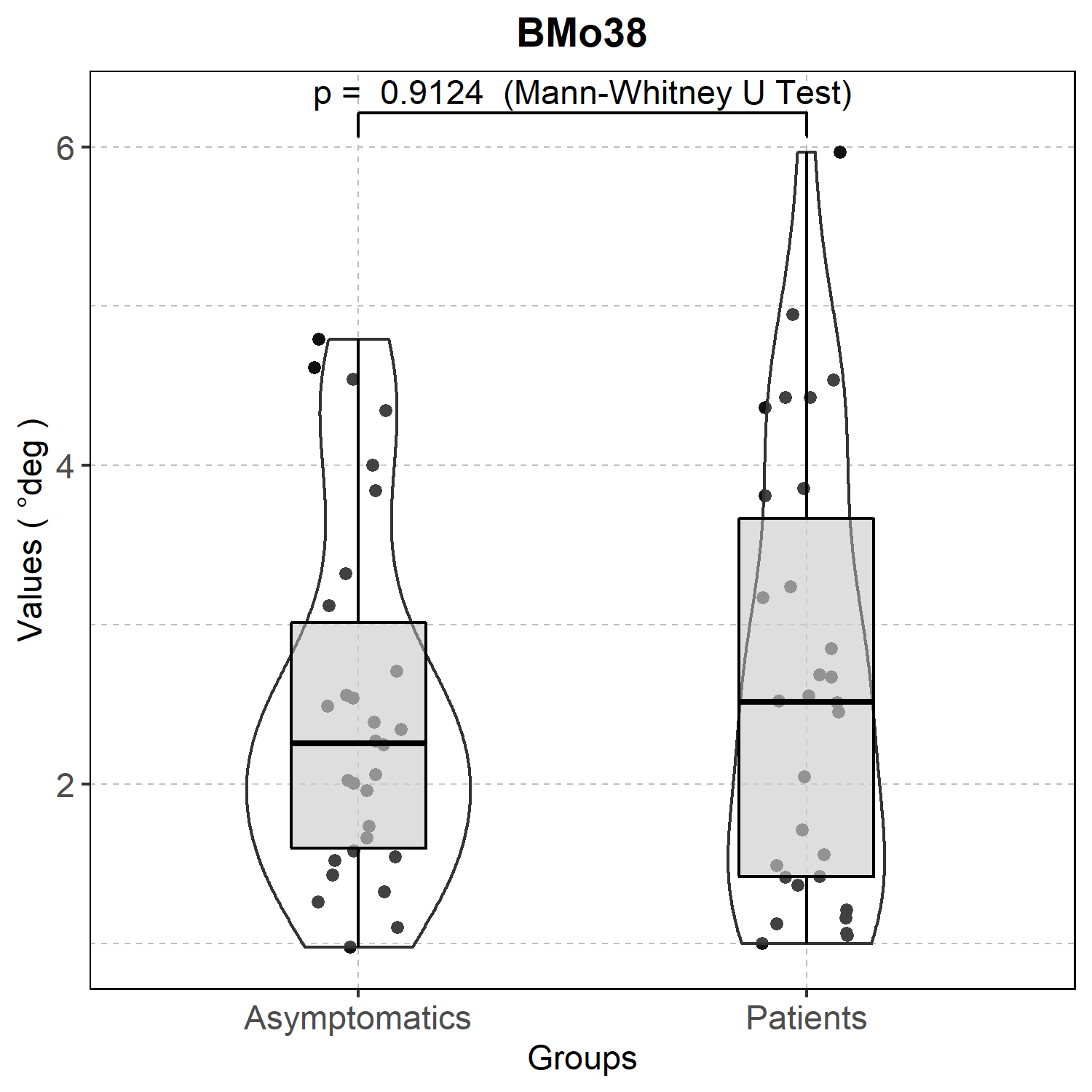

Supplement: Supplementary file 2 — Supplementary Information 2. [file 41598_2023_33504_MOESM2_ESM.zip › BMo038_boxplot.png]

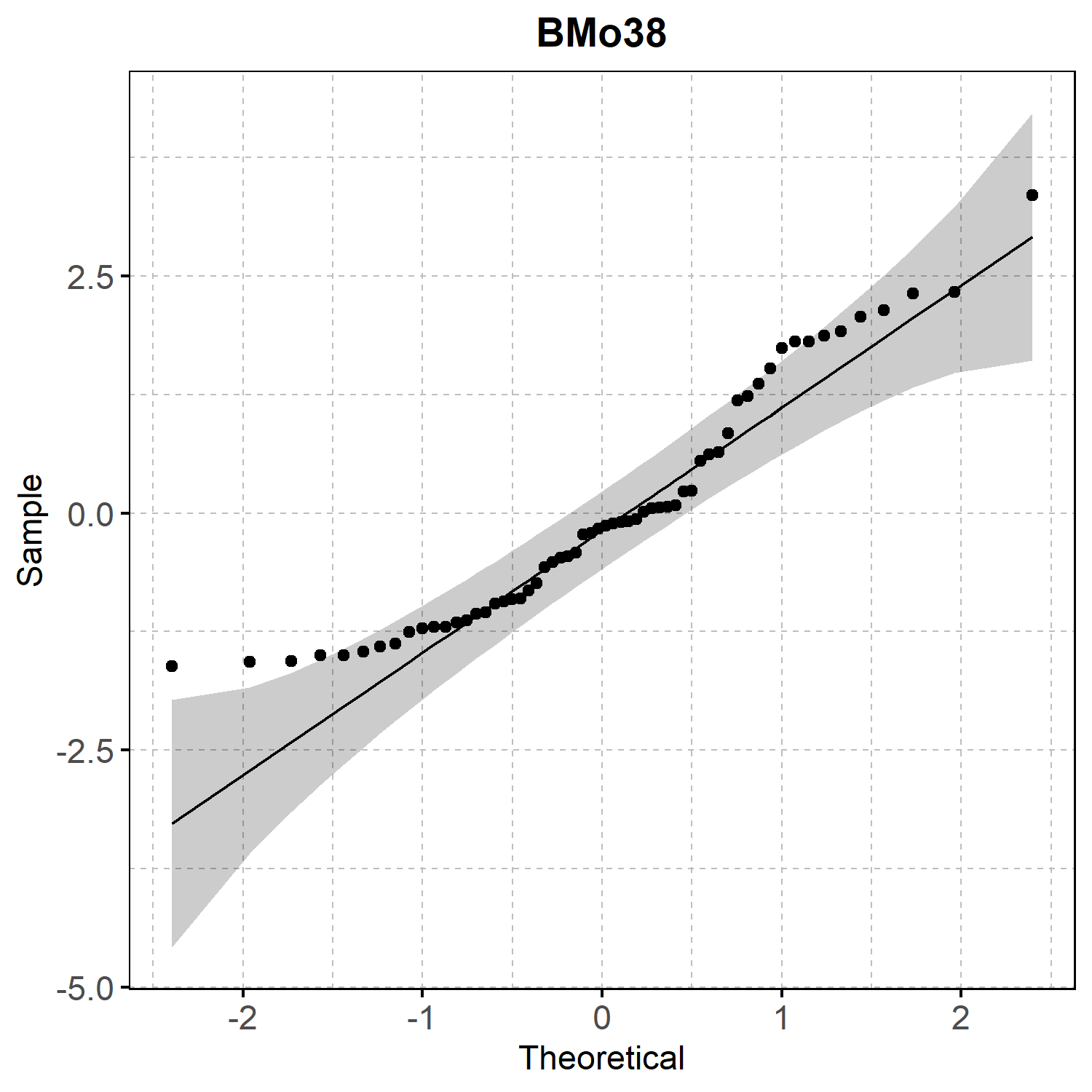

Supplement: Supplementary file 2 — Supplementary Information 2. [file 41598_2023_33504_MOESM2_ESM.zip › BMo038_normality.png]

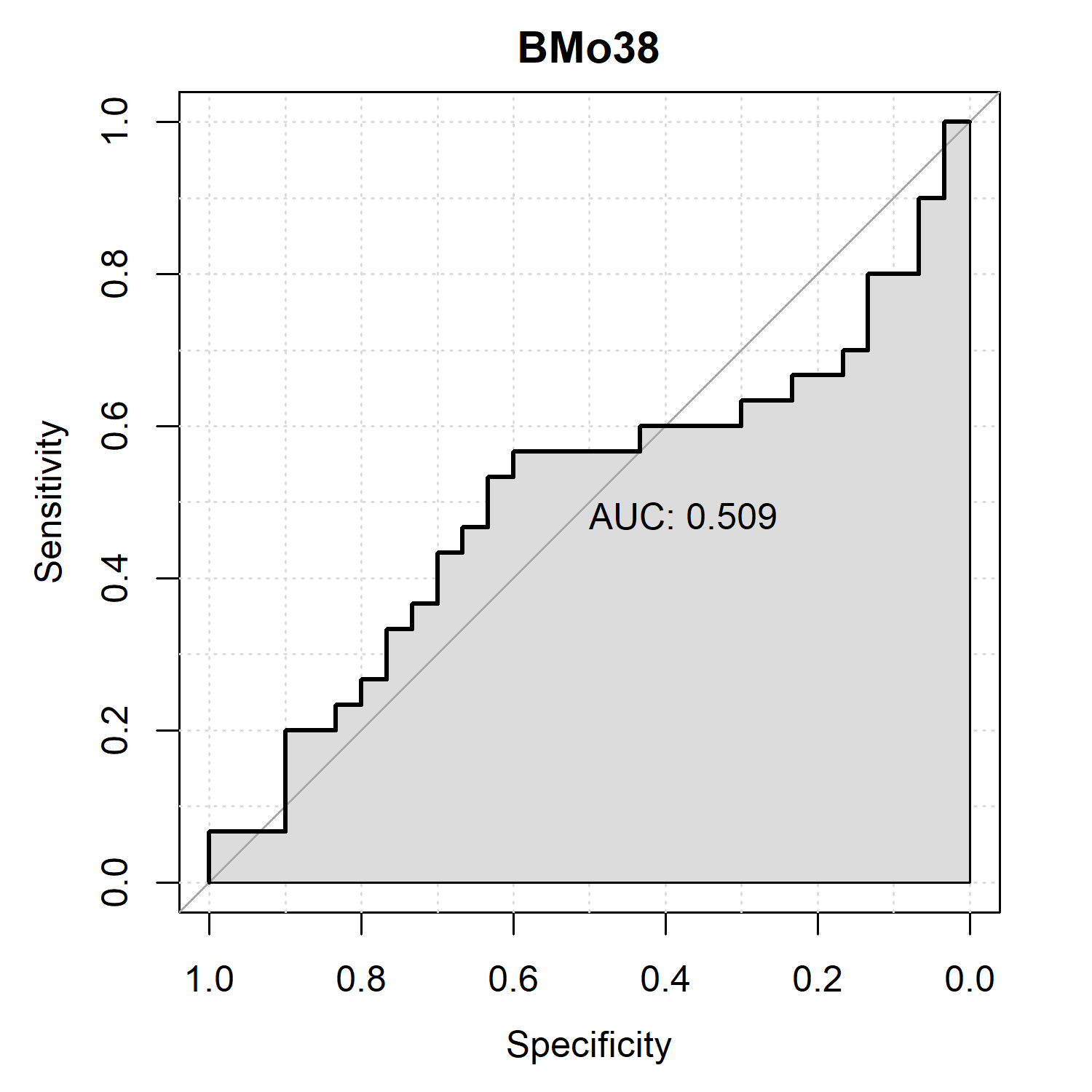

Supplement: Supplementary file 2 — Supplementary Information 2. [file 41598_2023_33504_MOESM2_ESM.zip › BMo038_ROC.png]

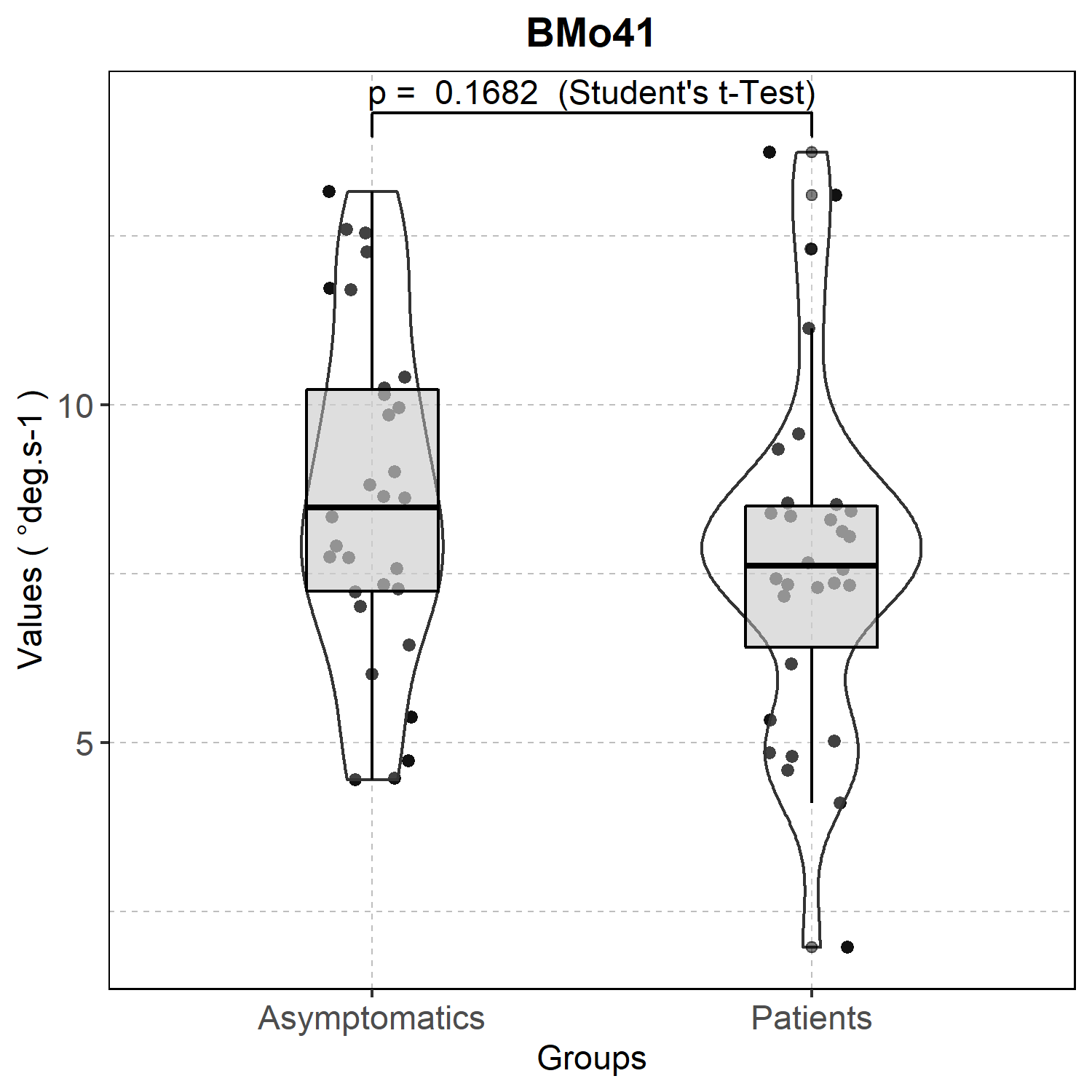

Supplement: Supplementary file 2 — Supplementary Information 2. [file 41598_2023_33504_MOESM2_ESM.zip › BMo041_boxplot.png]

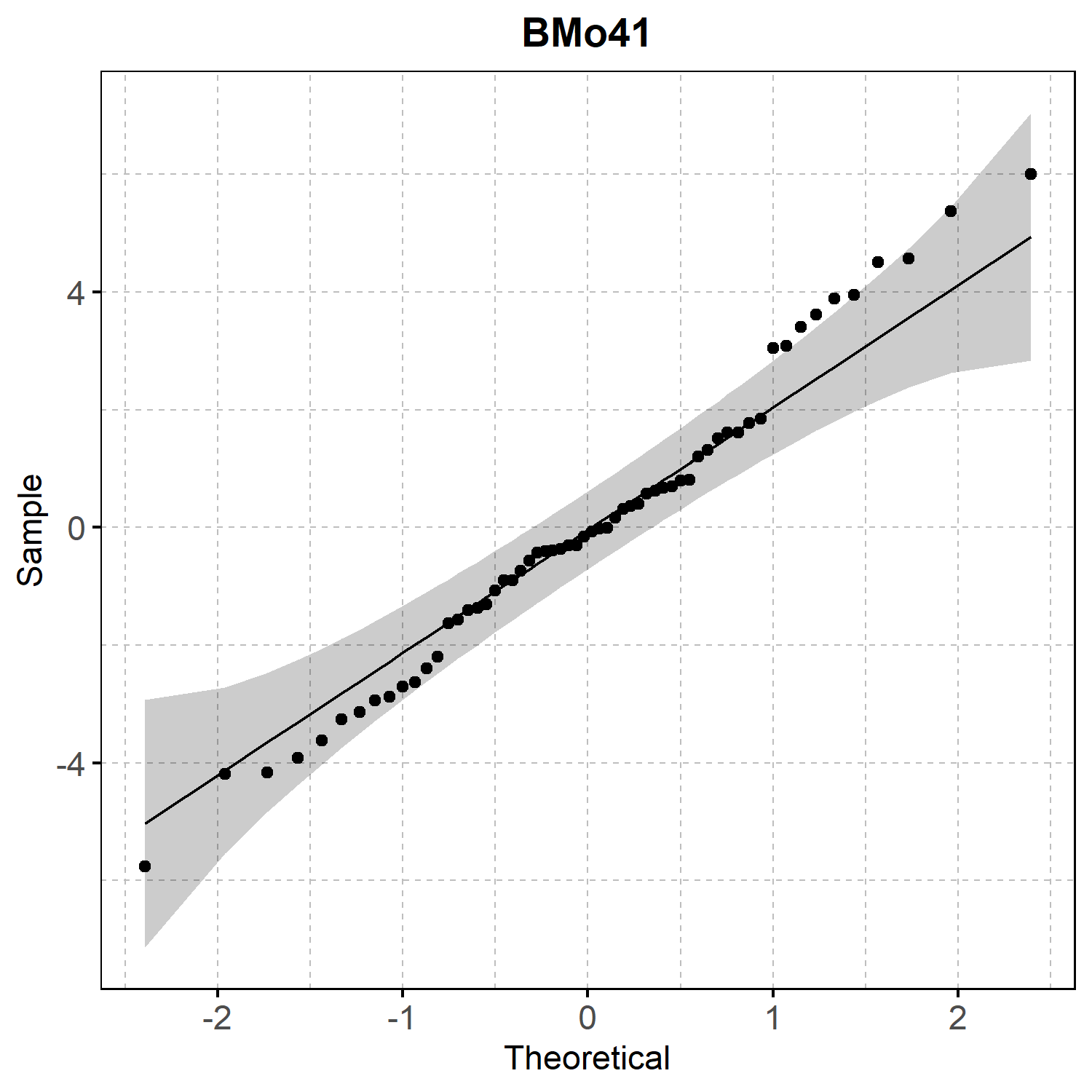

Supplement: Supplementary file 2 — Supplementary Information 2. [file 41598_2023_33504_MOESM2_ESM.zip › BMo041_normality.png]

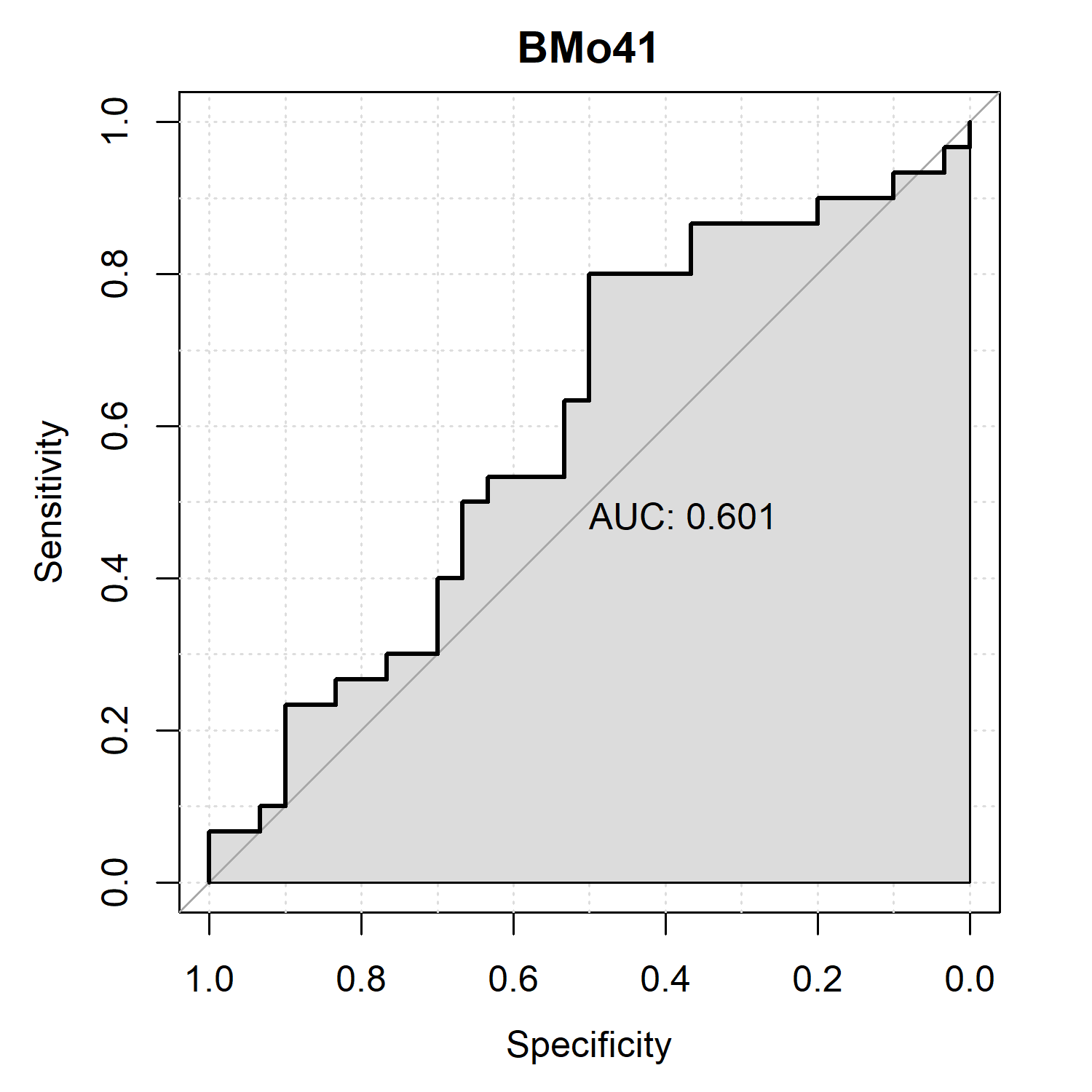

Supplement: Supplementary file 2 — Supplementary Information 2. [file 41598_2023_33504_MOESM2_ESM.zip › BMo041_ROC.png]

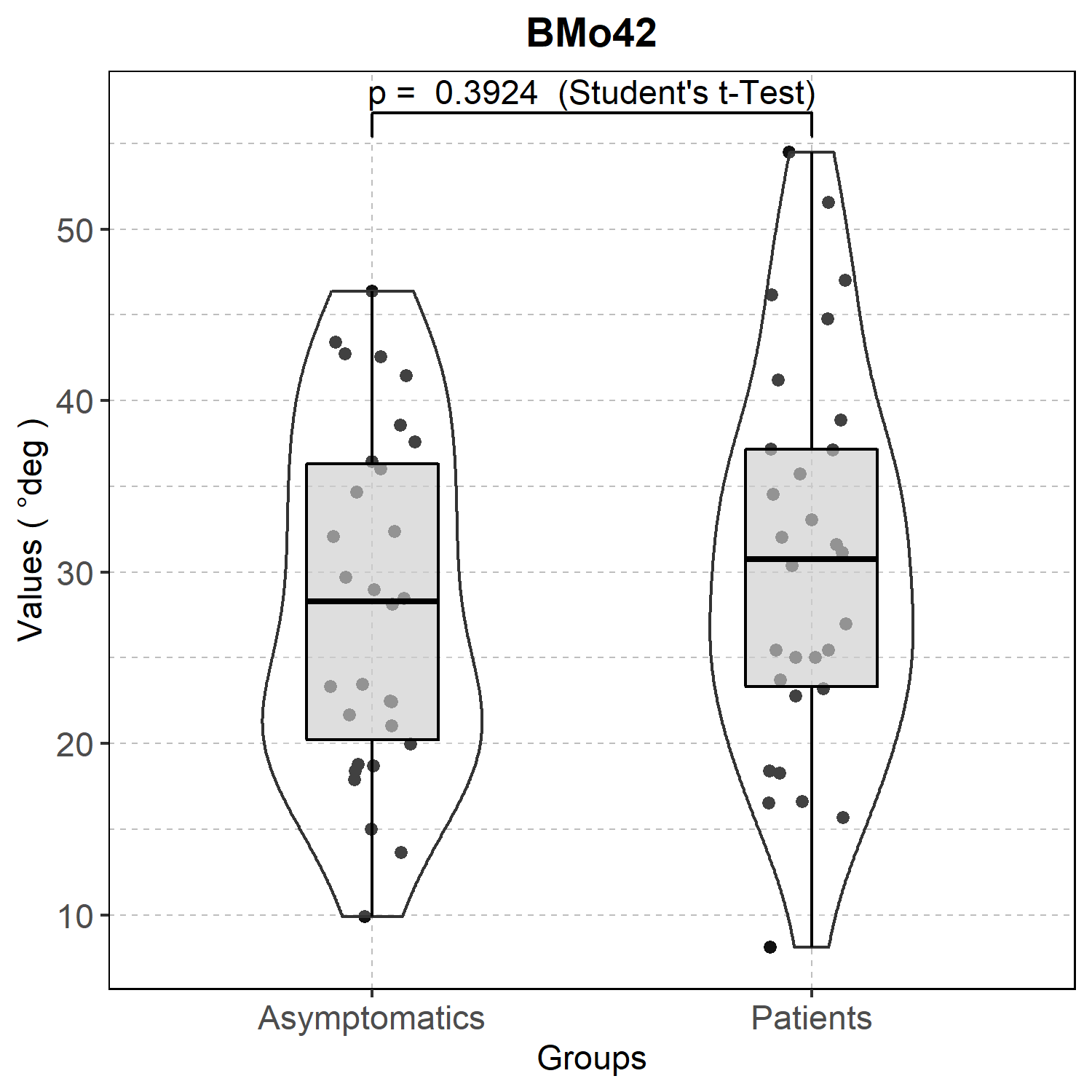

Supplement: Supplementary file 2 — Supplementary Information 2. [file 41598_2023_33504_MOESM2_ESM.zip › BMo042_boxplot.png]

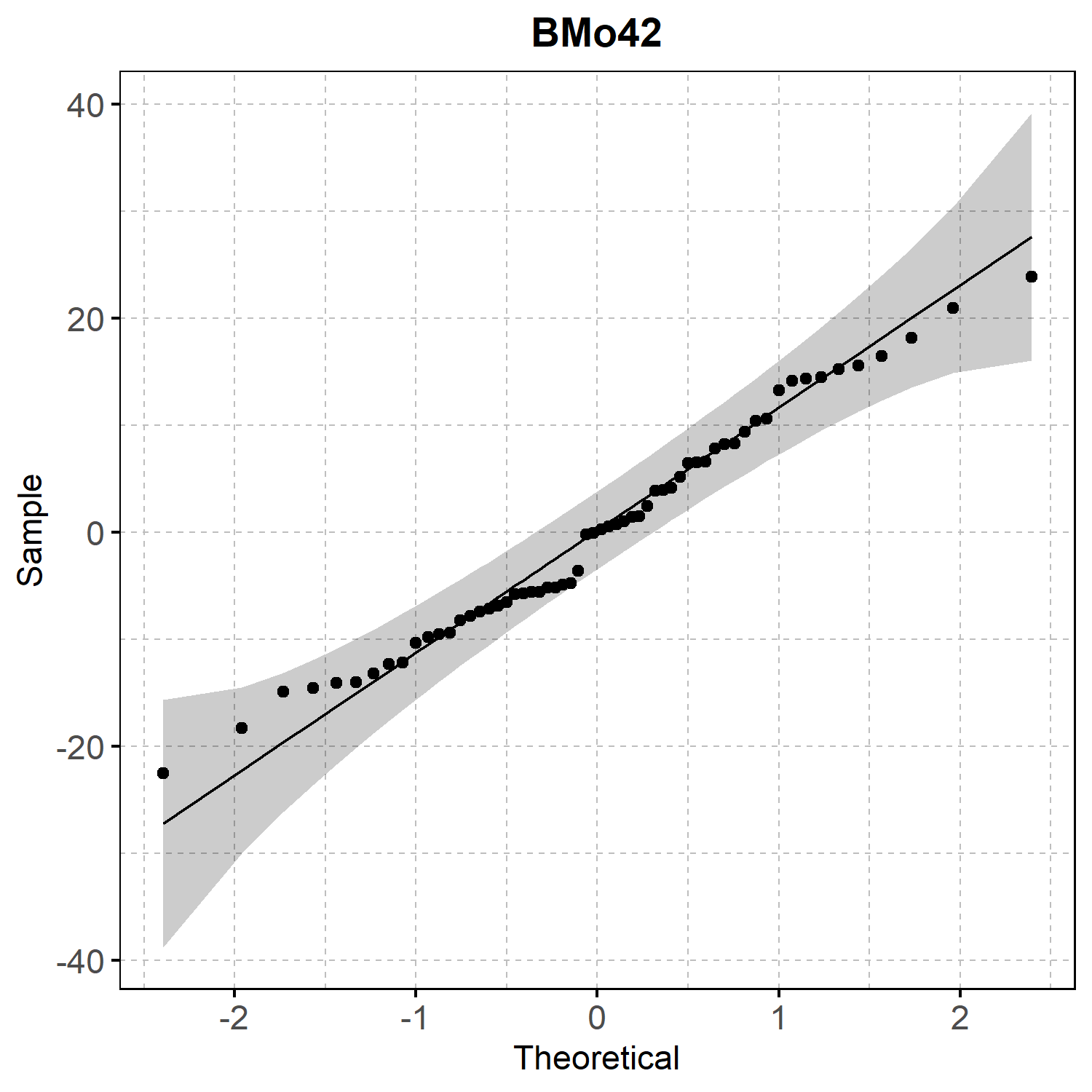

Supplement: Supplementary file 2 — Supplementary Information 2. [file 41598_2023_33504_MOESM2_ESM.zip › BMo042_normality.png]

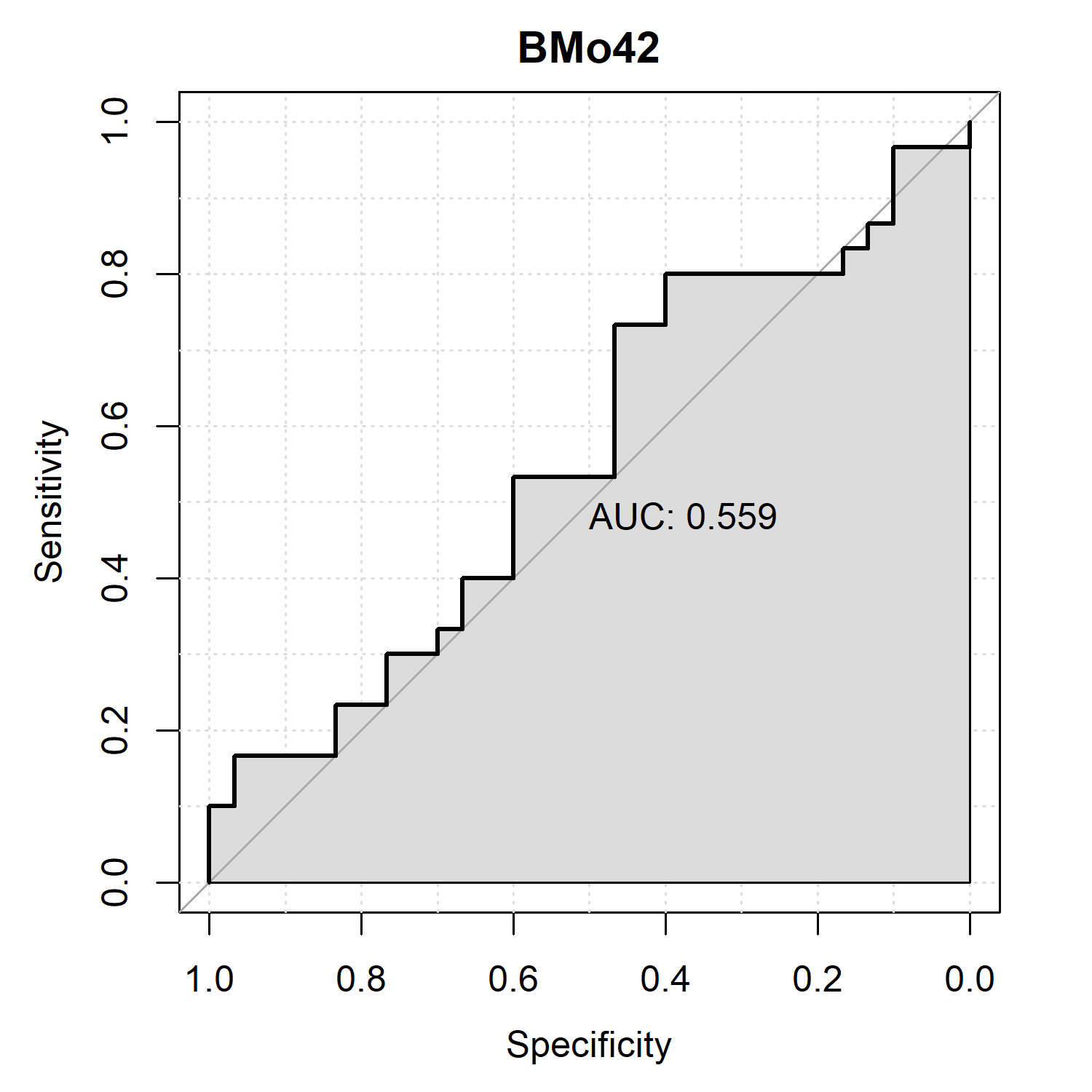

Supplement: Supplementary file 2 — Supplementary Information 2. [file 41598_2023_33504_MOESM2_ESM.zip › BMo042_ROC.png]

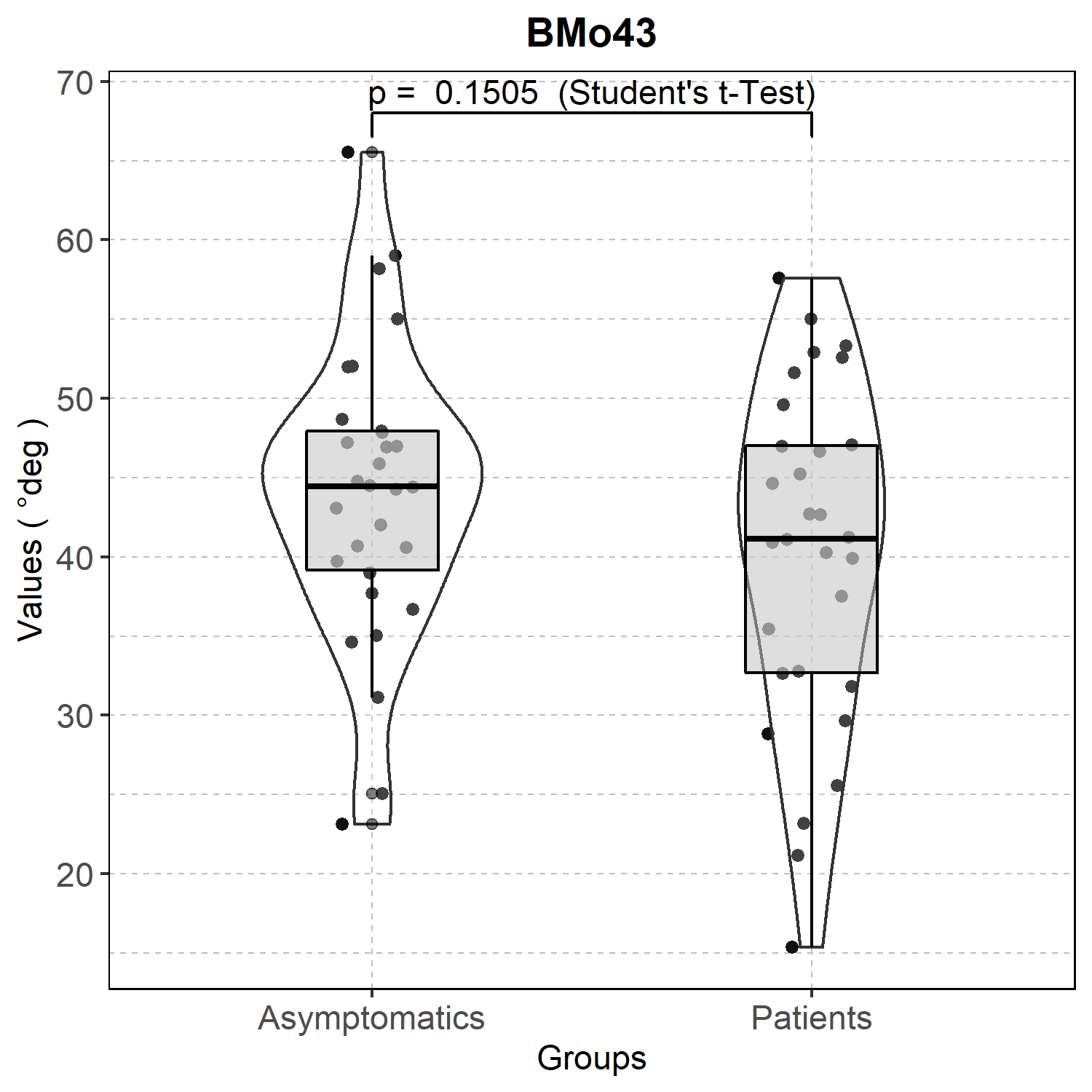

Supplement: Supplementary file 2 — Supplementary Information 2. [file 41598_2023_33504_MOESM2_ESM.zip › BMo043_boxplot.png]

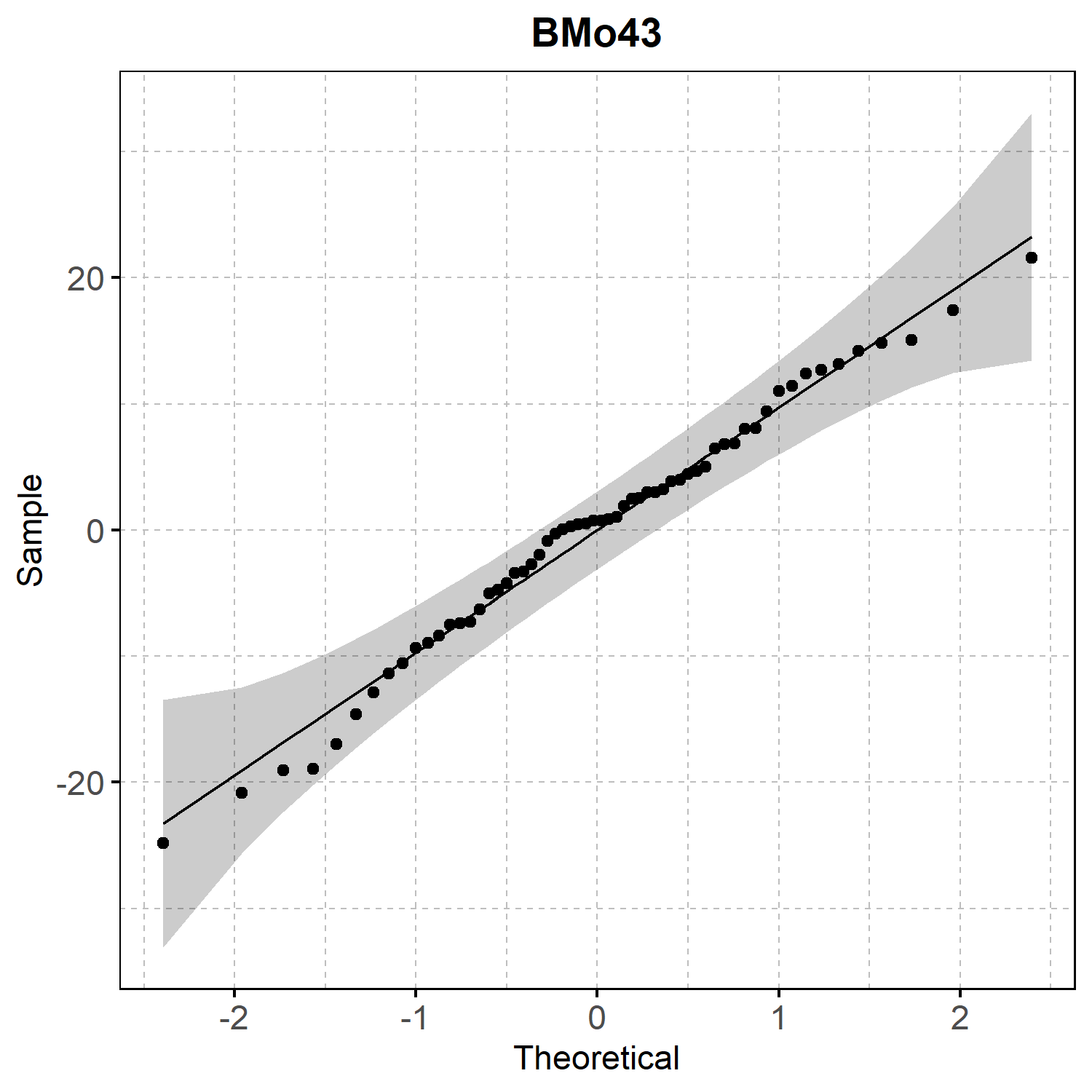

Supplement: Supplementary file 2 — Supplementary Information 2. [file 41598_2023_33504_MOESM2_ESM.zip › BMo043_normality.png]

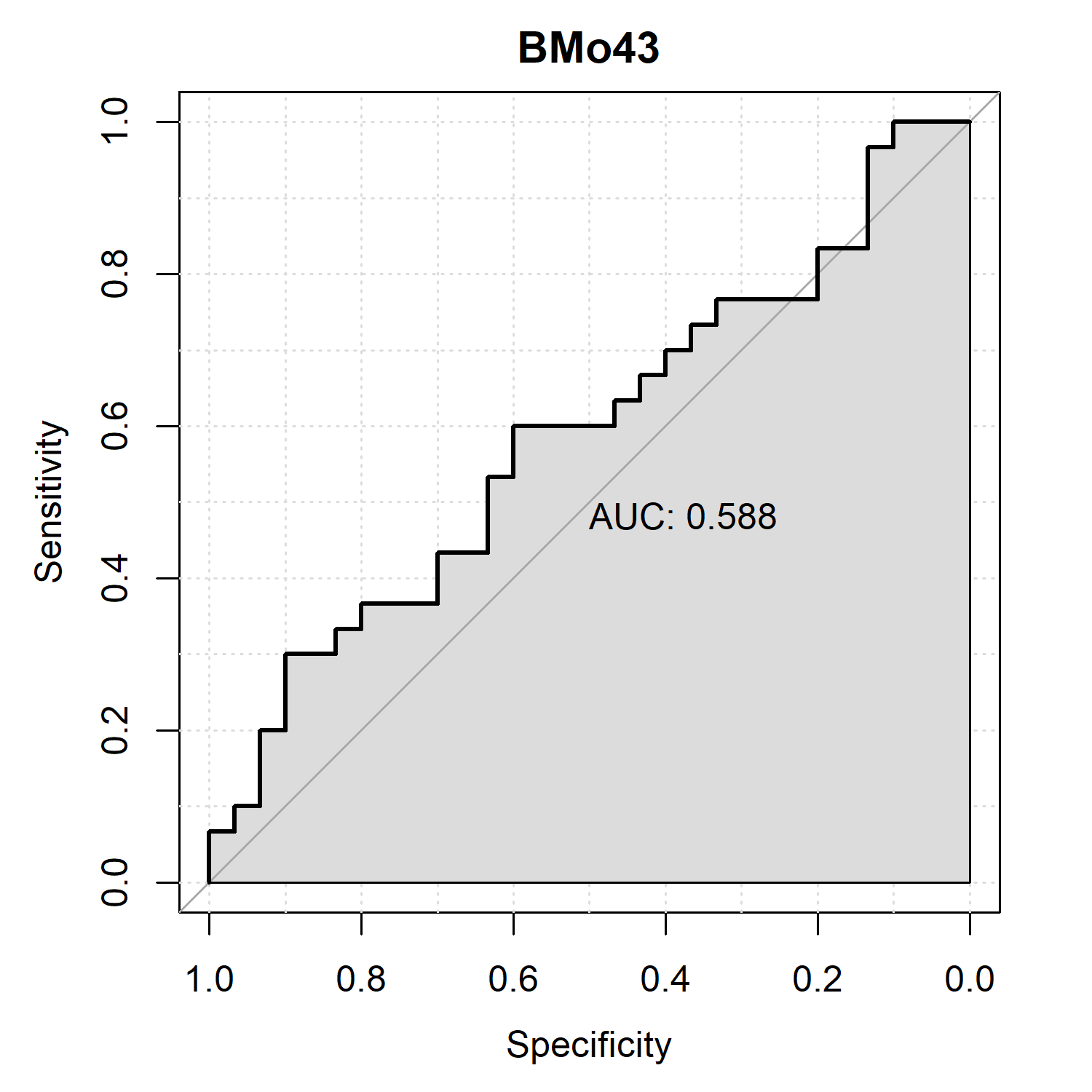

Supplement: Supplementary file 2 — Supplementary Information 2. [file 41598_2023_33504_MOESM2_ESM.zip › BMo043_ROC.png]

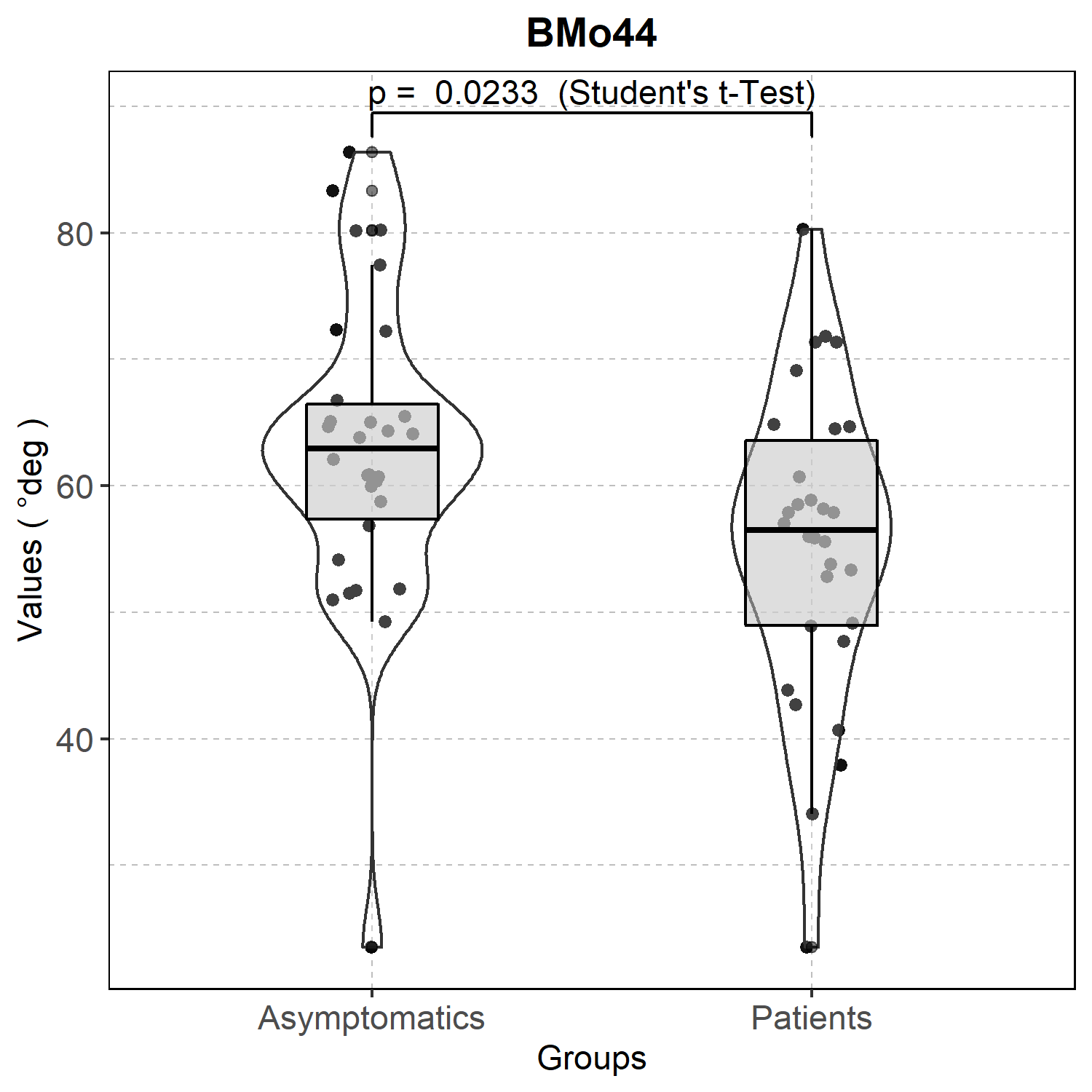

Supplement: Supplementary file 2 — Supplementary Information 2. [file 41598_2023_33504_MOESM2_ESM.zip › BMo044_boxplot.png]

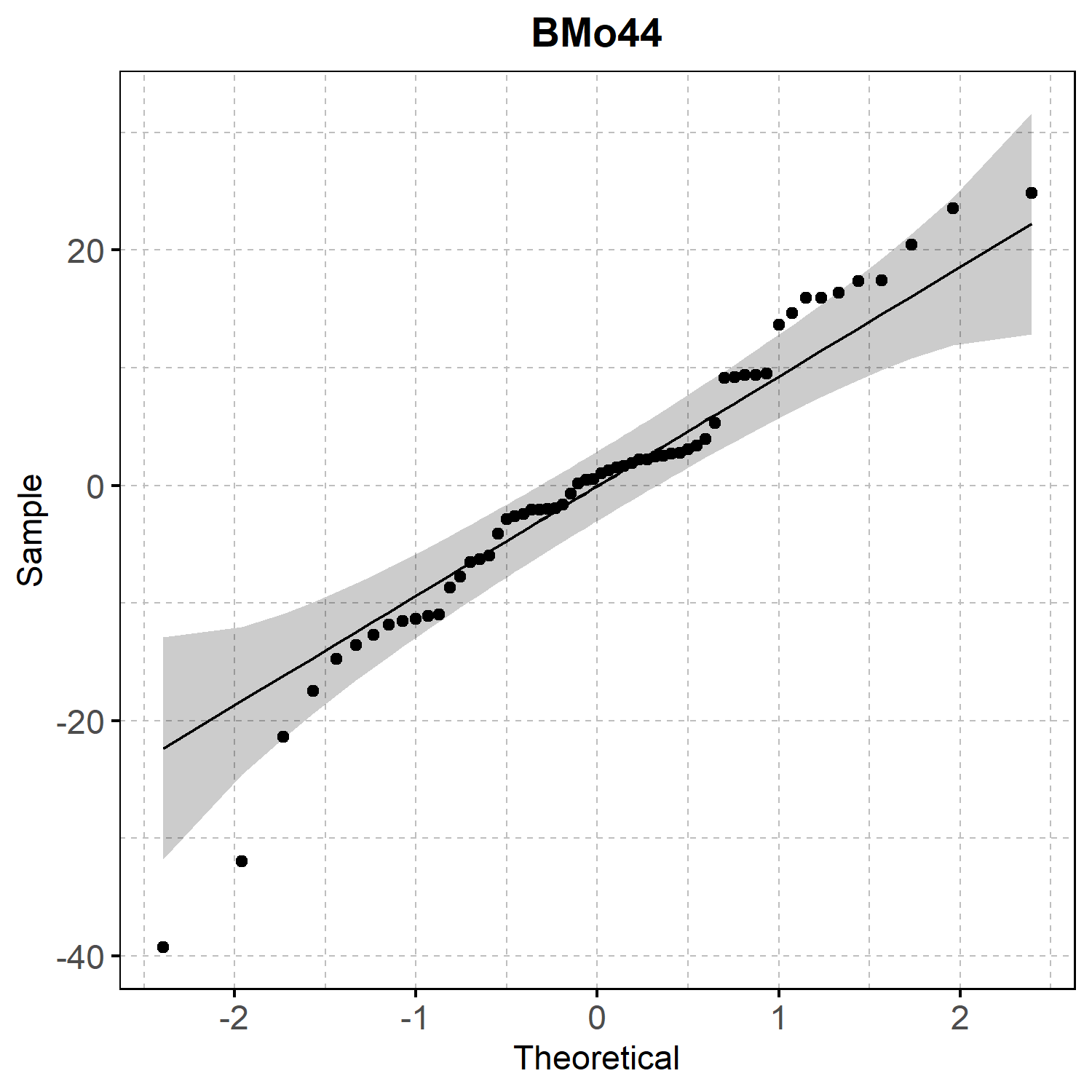

Supplement: Supplementary file 2 — Supplementary Information 2. [file 41598_2023_33504_MOESM2_ESM.zip › BMo044_normality.png]

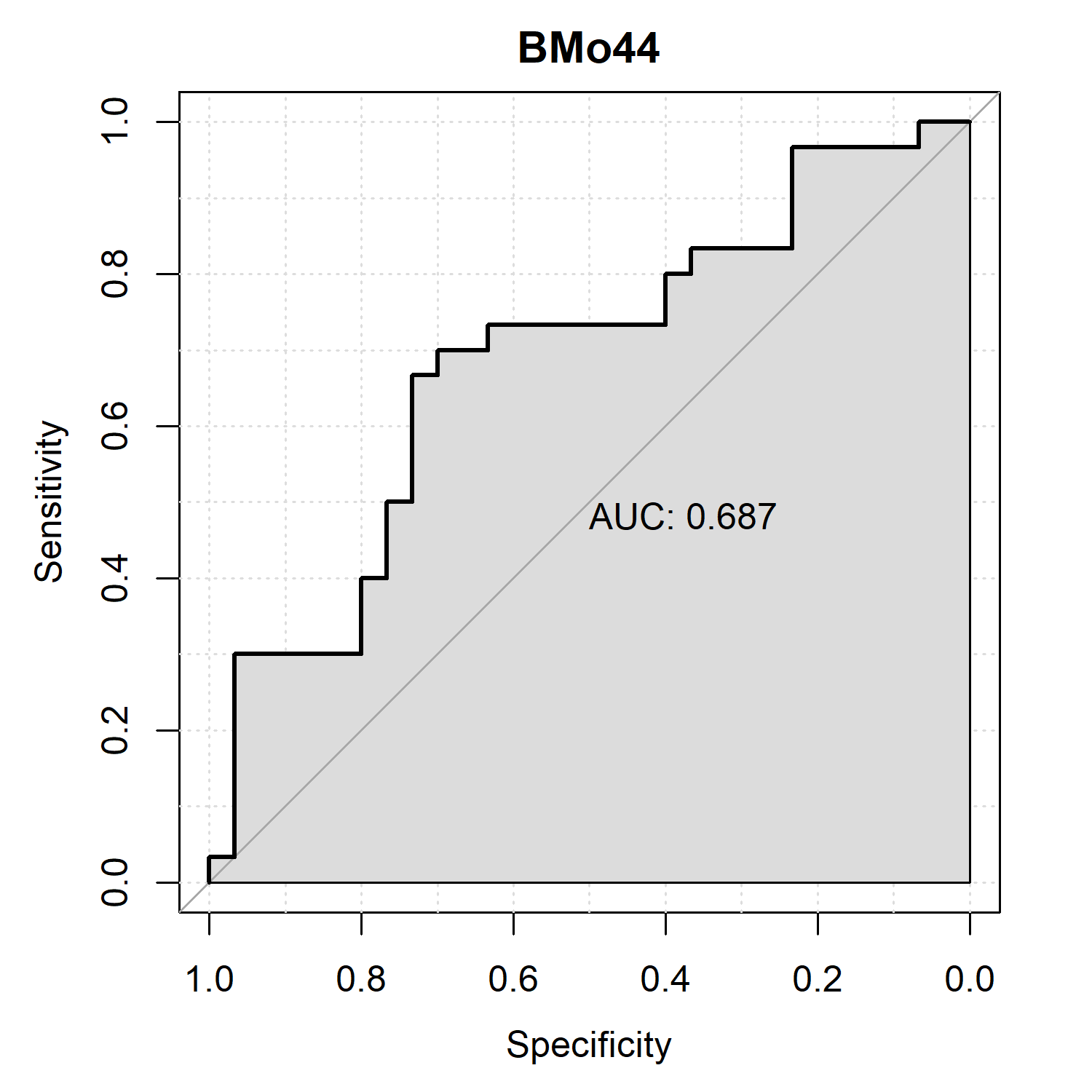

Supplement: Supplementary file 2 — Supplementary Information 2. [file 41598_2023_33504_MOESM2_ESM.zip › BMo044_ROC.png]

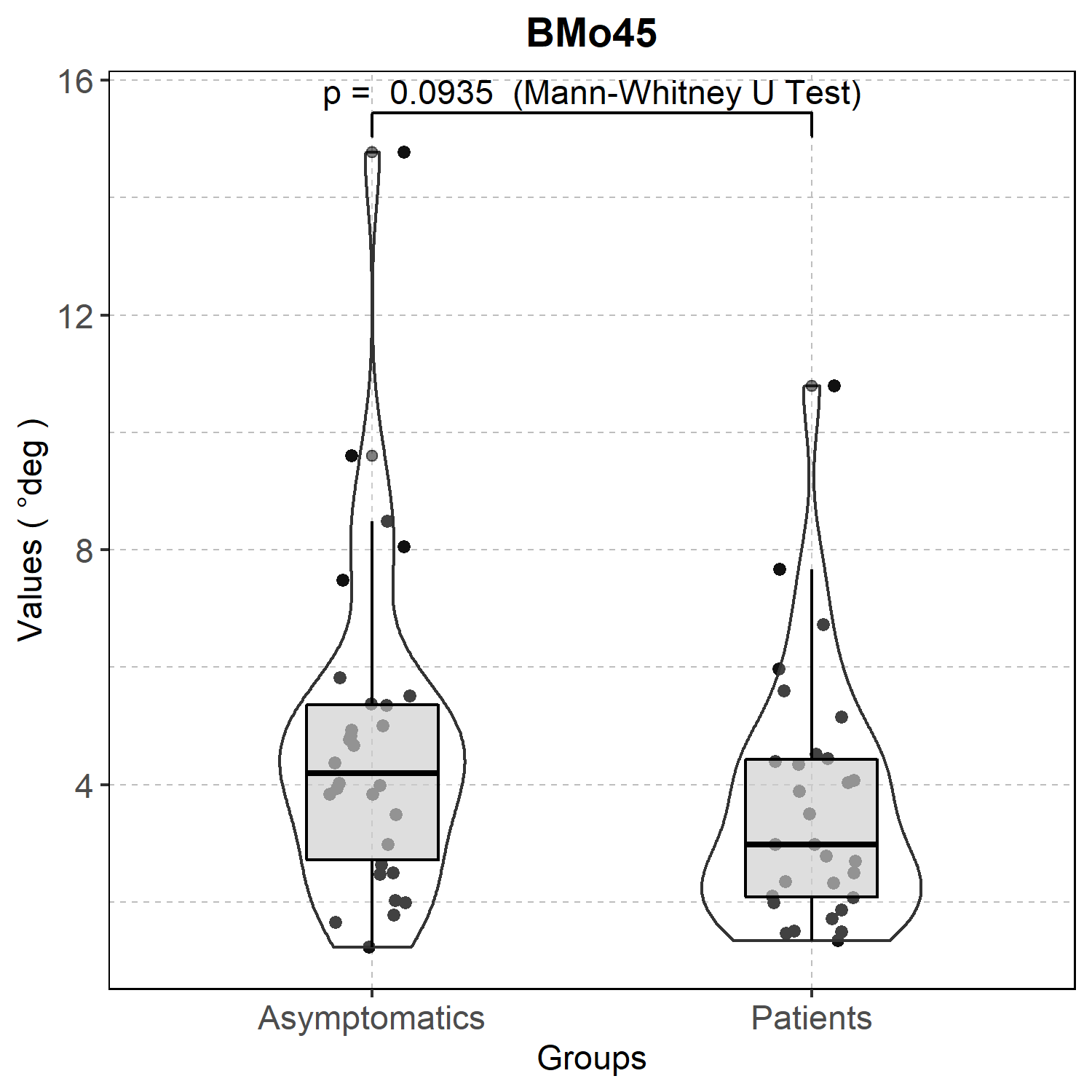

Supplement: Supplementary file 2 — Supplementary Information 2. [file 41598_2023_33504_MOESM2_ESM.zip › BMo045_boxplot.png]

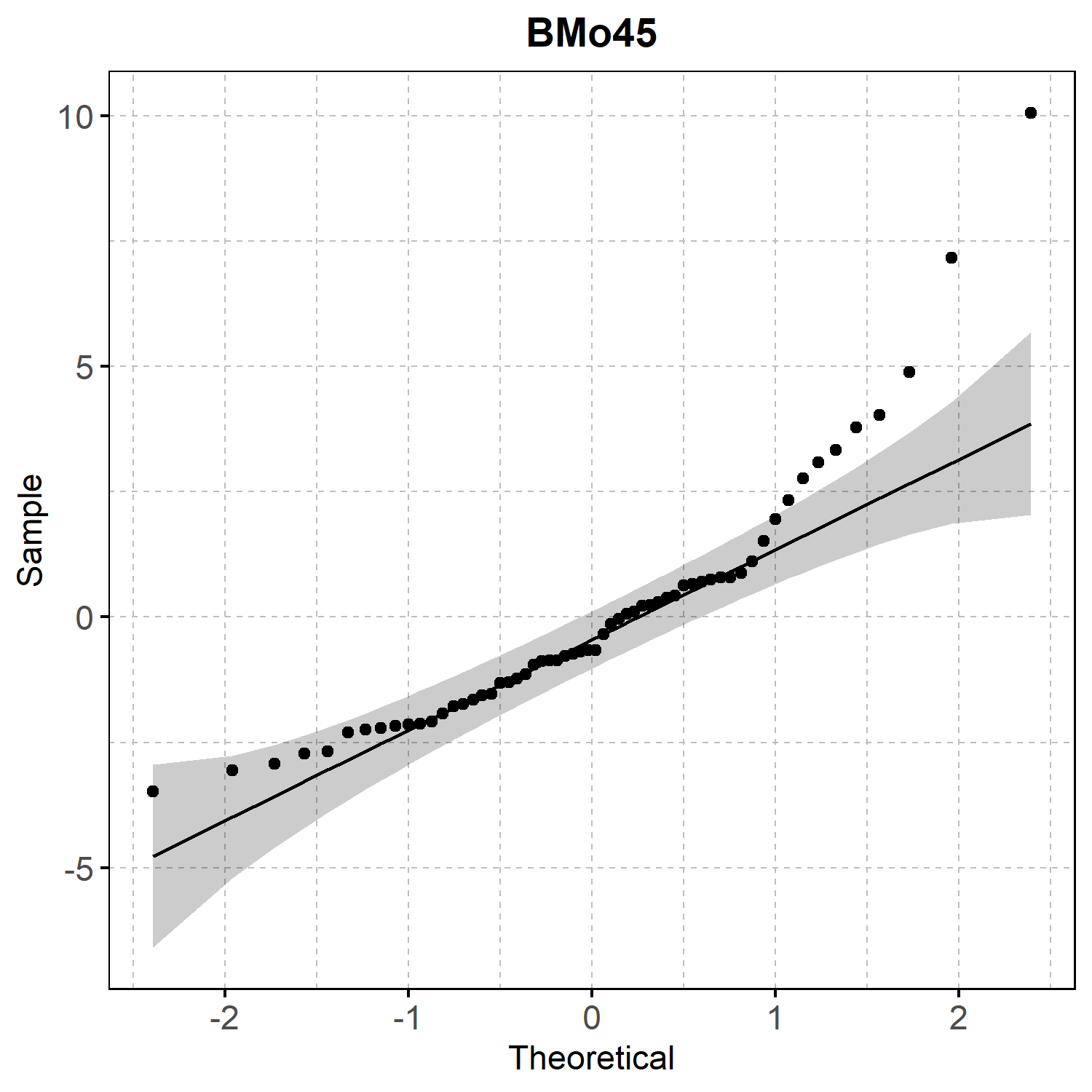

Supplement: Supplementary file 2 — Supplementary Information 2. [file 41598_2023_33504_MOESM2_ESM.zip › BMo045_normality.png]

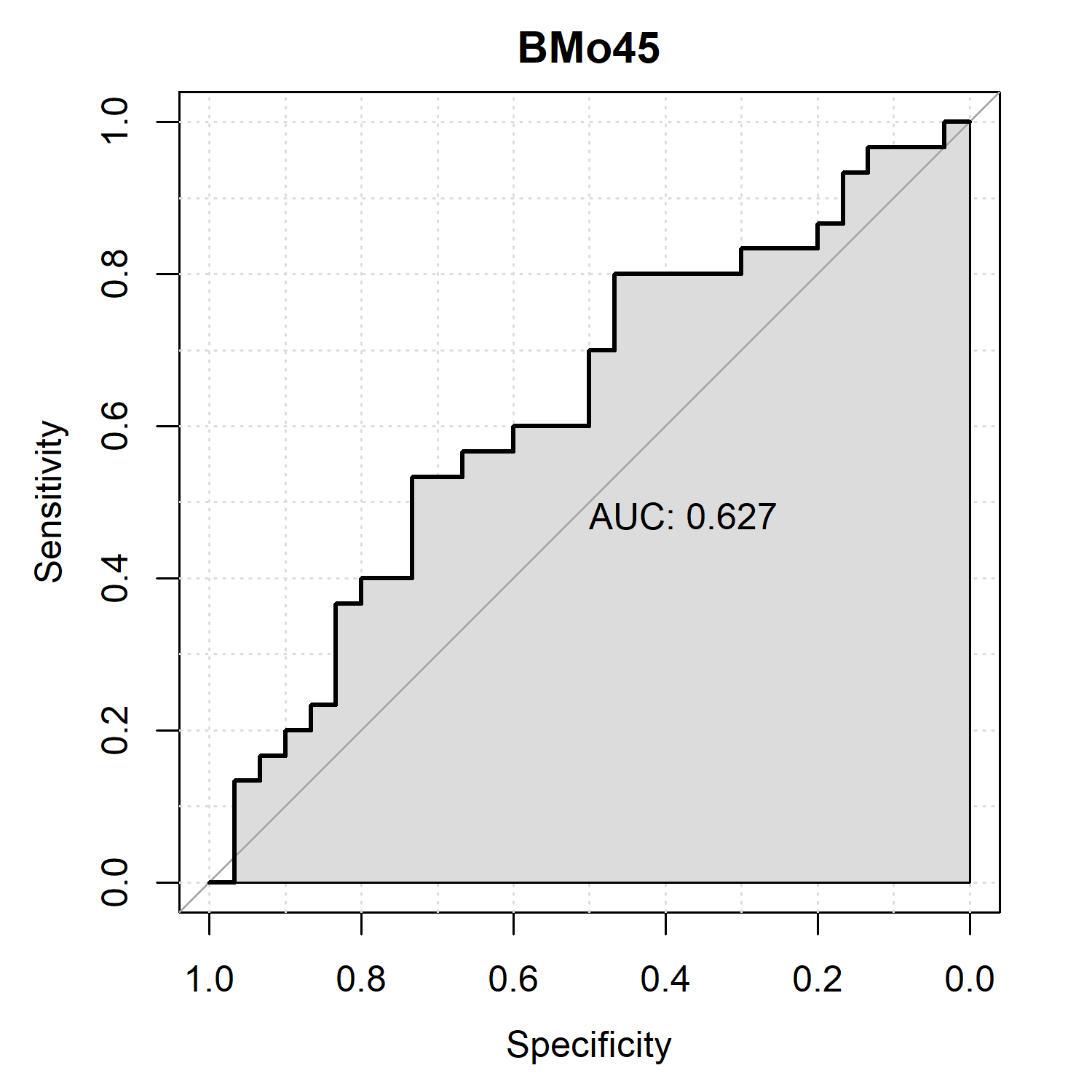

Supplement: Supplementary file 2 — Supplementary Information 2. [file 41598_2023_33504_MOESM2_ESM.zip › BMo045_ROC.png]

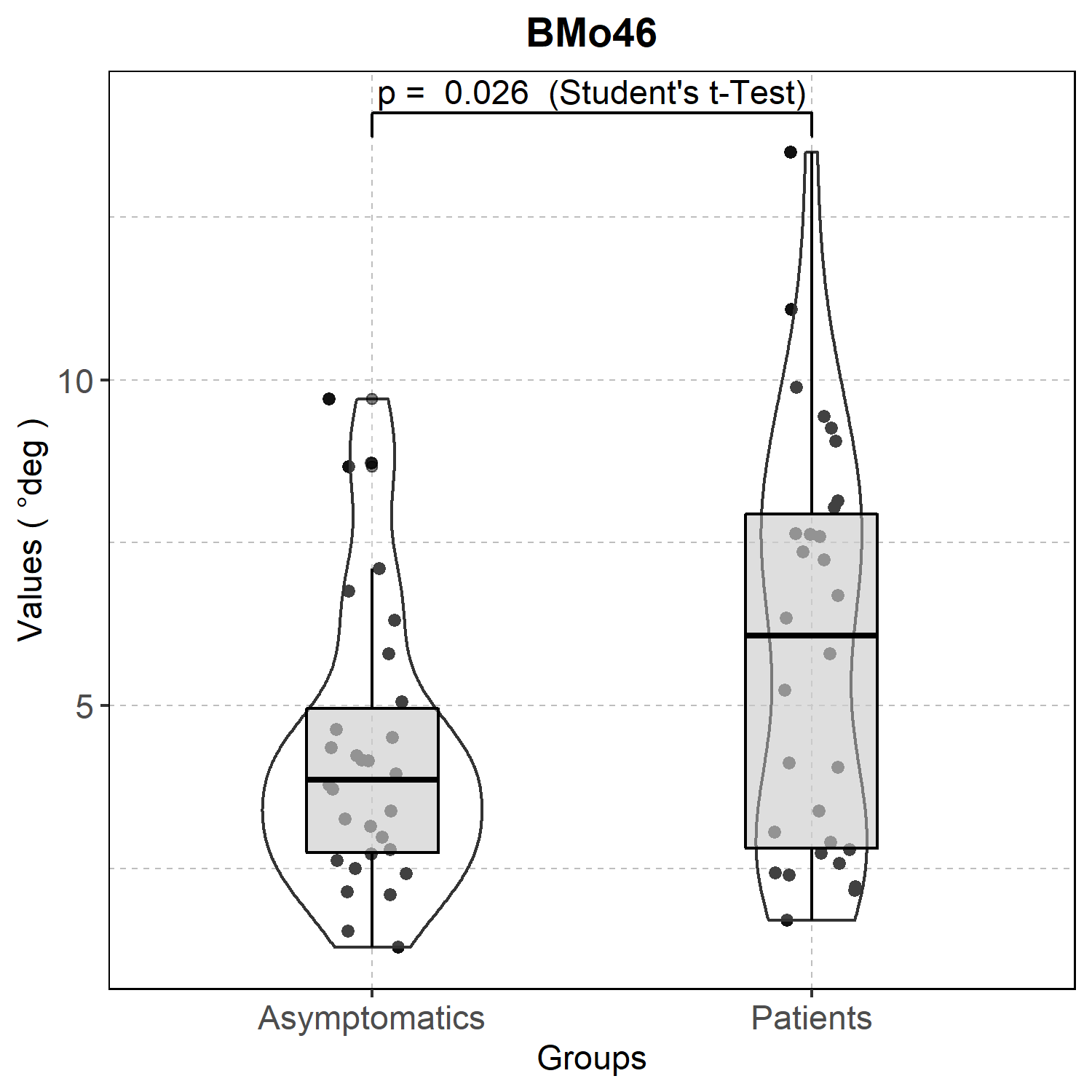

Supplement: Supplementary file 2 — Supplementary Information 2. [file 41598_2023_33504_MOESM2_ESM.zip › BMo046_boxplot.png]

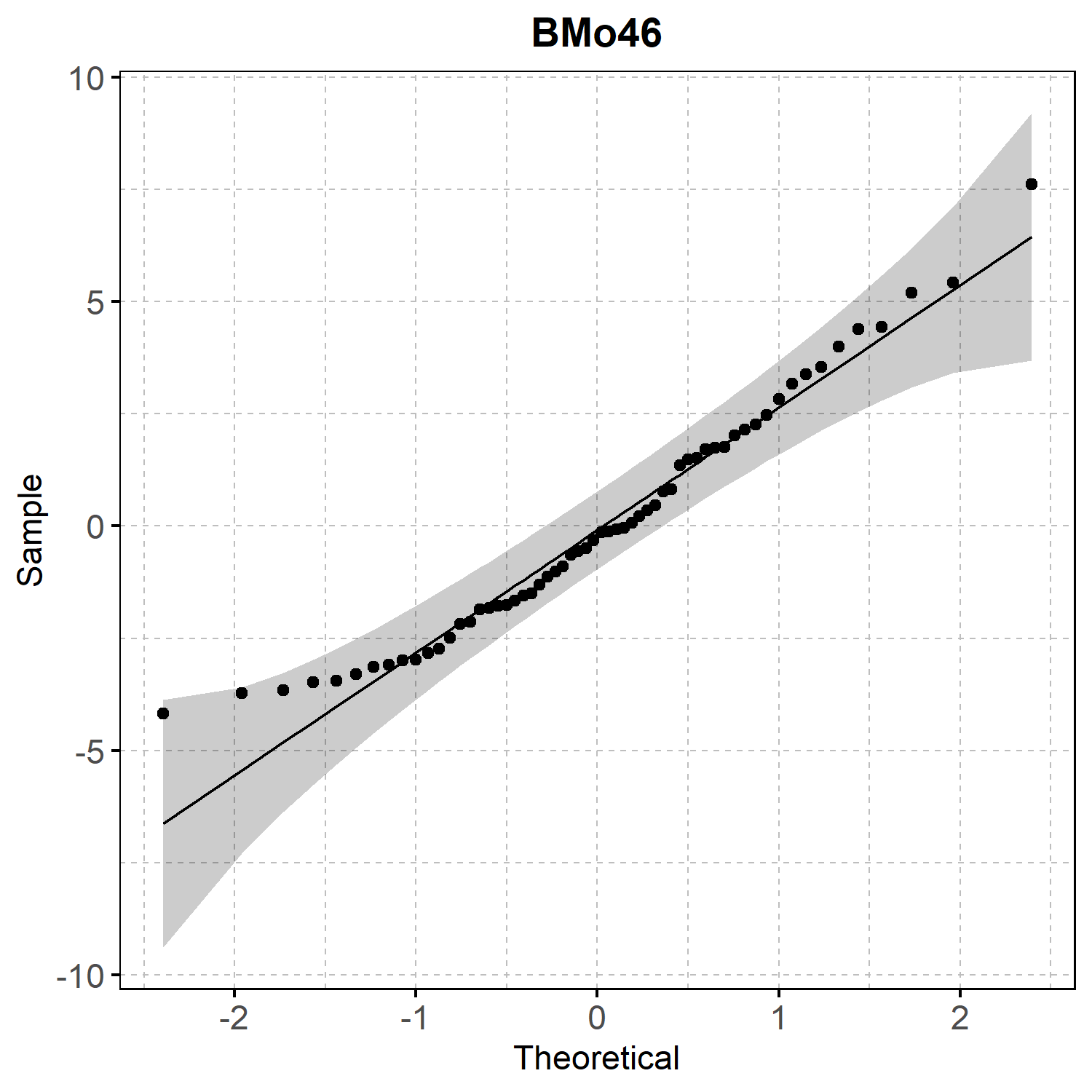

Supplement: Supplementary file 2 — Supplementary Information 2. [file 41598_2023_33504_MOESM2_ESM.zip › BMo046_normality.png]

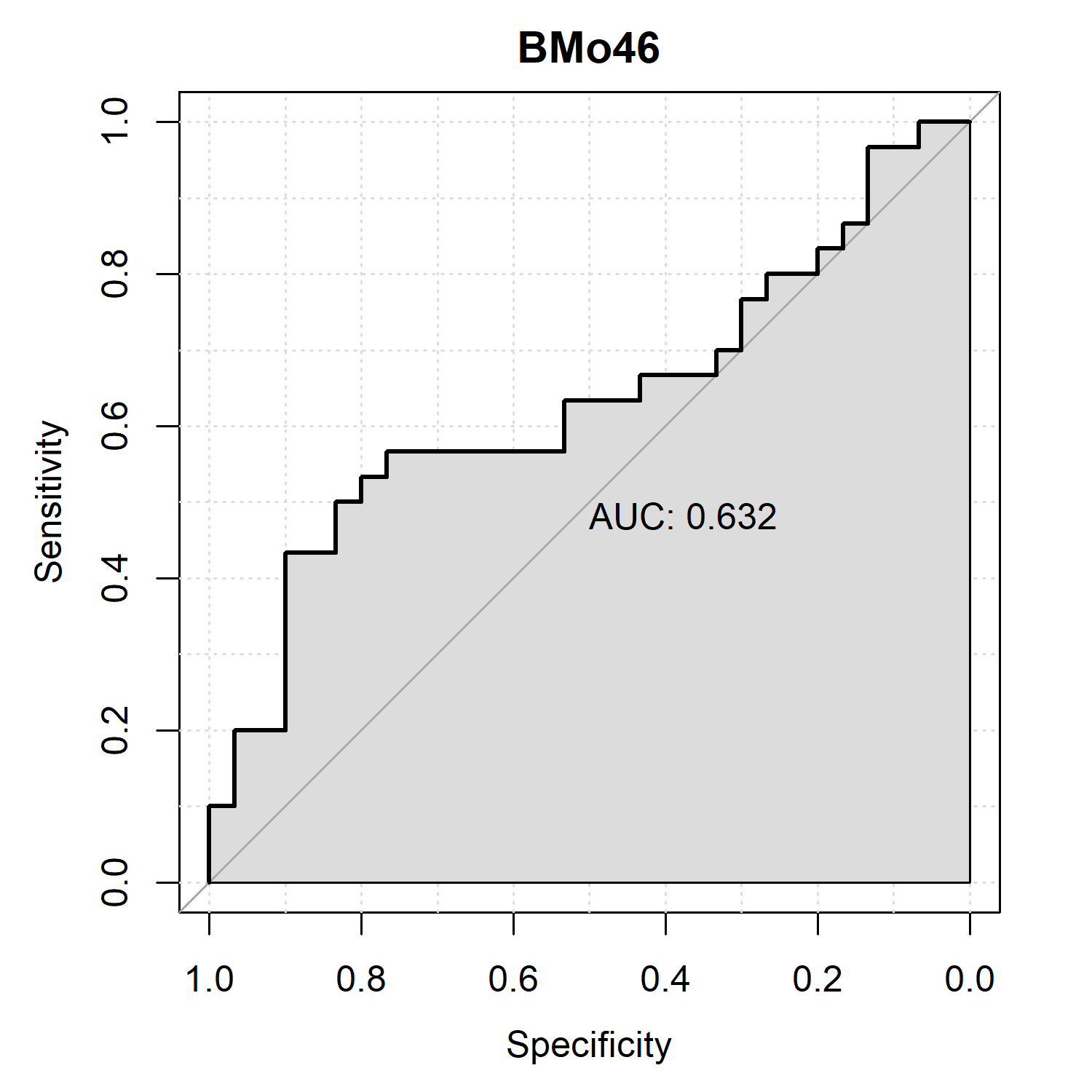

Supplement: Supplementary file 2 — Supplementary Information 2. [file 41598_2023_33504_MOESM2_ESM.zip › BMo046_ROC.png]

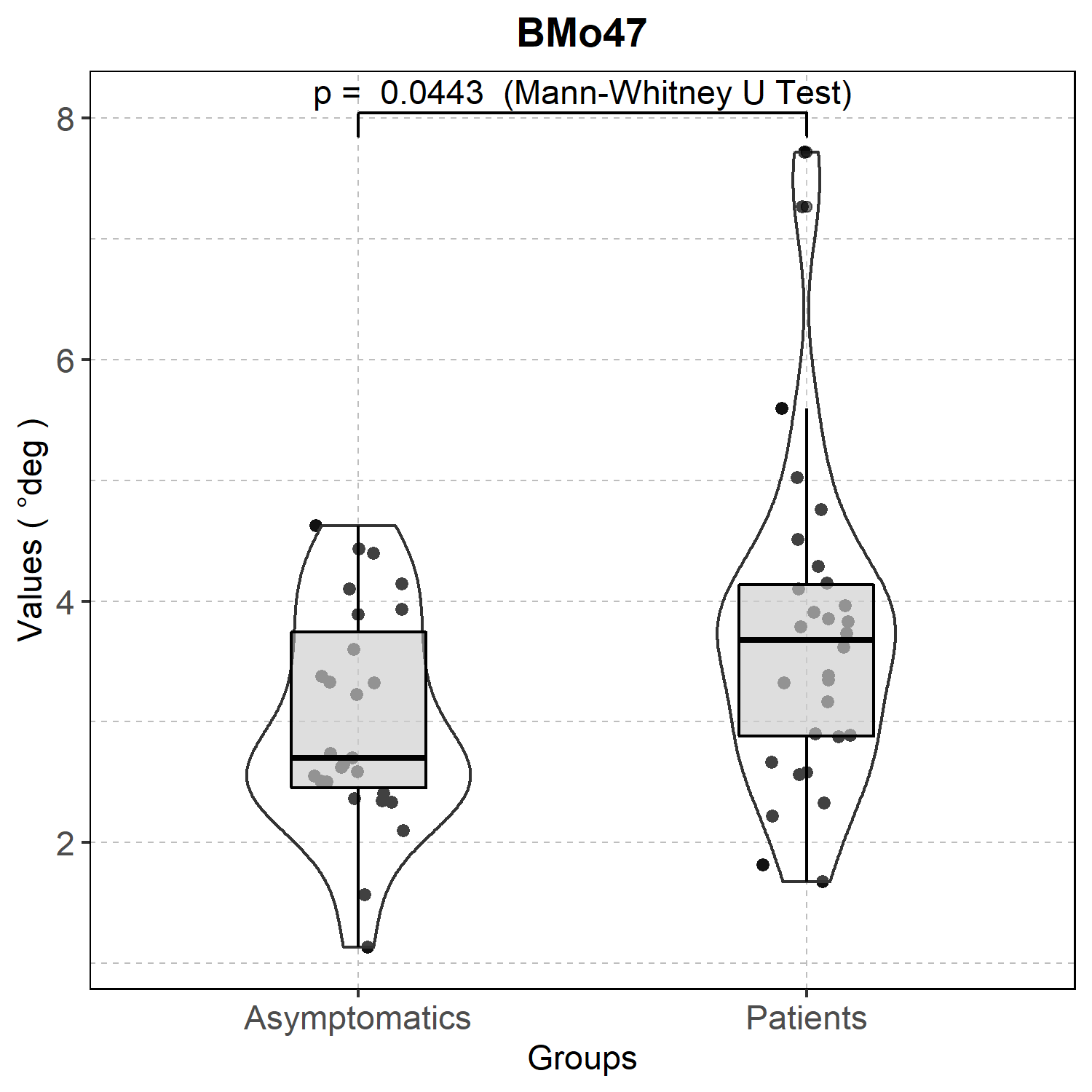

Supplement: Supplementary file 2 — Supplementary Information 2. [file 41598_2023_33504_MOESM2_ESM.zip › BMo047_boxplot.png]
