# Supplementary material for: Measurement properties of 72 movement biomarkers aiming to discriminate non‑specific chronic low back pain patients from an asymptomatic population
Source: Sci Rep. 2023 Apr 20;13:6483. doi: 10.1038/s41598-023-33504-5 (PMC10119171; doi:10.1038/s41598-023-33504-5)
Supplement: Supplementary file 2 — Supplementary Information 2. [file 41598_2023_33504_MOESM2_ESM.zip › _merged.pdf]

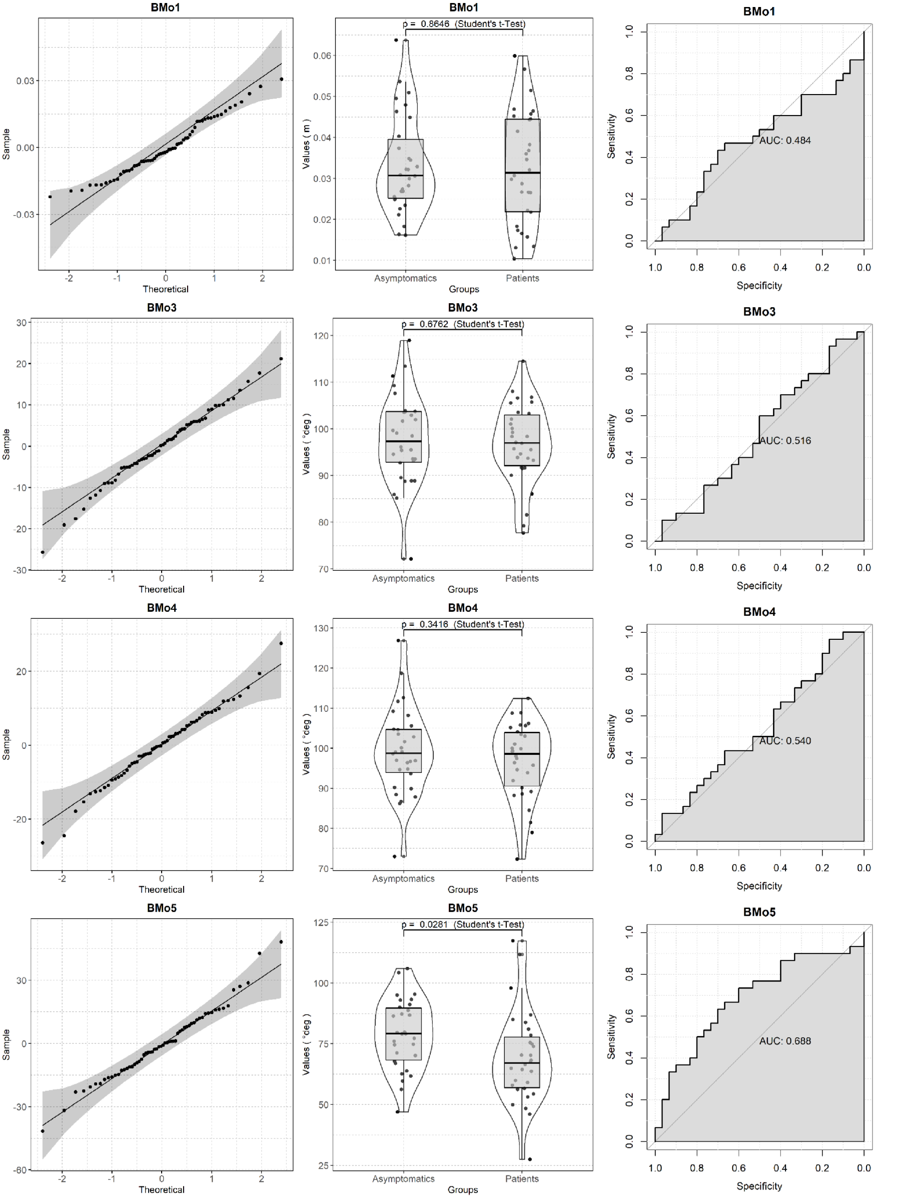
BMo1
Sample
Theoretical
BMo1
p = 0.8646 (Student's t-Test)
Values (m)
Asymptomatics
Patients
Groups
BMo1
Sensitivity
Specificity
AUC: 0.484
BMo3
Sample
Theoretical
BMo3
p = 0.6762 (Student's t-Test)
Values (°deg)
Asymptomatics
Patients
Groups
BMo3
Sensitivity
Specificity
AUC: 0.516
BMo4
Sample
Theoretical
BMo4
p = 0.3416 (Student's t-Test)
Values (°deg)
Asymptomatics
Patients
Groups
BMo4
Sensitivity
Specificity
AUC: 0.540
BMo5
Sample
Theoretical
BMo5
p = 0.0281 (Student's t-Test)
Values (°deg)
Asymptomatics
Patients
Groups
BMo5
Sensitivity
Specificity
AUC: 0.688

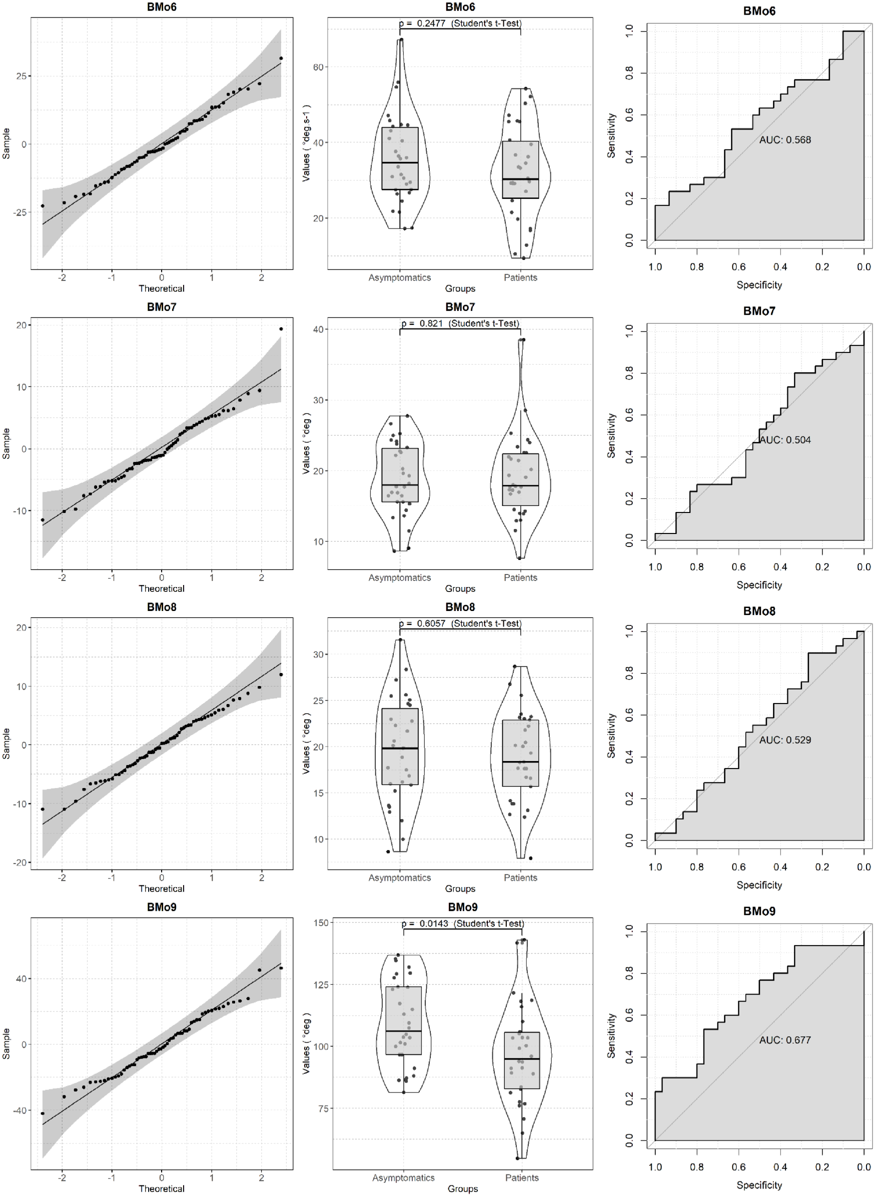

BMo6
Sample
Theoretical
BMo6
p = 0.2477 (Student's t-Test)
Values (°deg.s-1)
Asymptomatics
Patients
Groups
BMo6
AUC: 0.568
Sensitivity
Specificity
BMo7
Sample
Theoretical
BMo7
p = 0.821 (Student's t-Test)
Values (°deg)
Asymptomatics
Patients
Groups
BMo7
AUC: 0.504
Sensitivity
Specificity
BMo8
Sample
Theoretical
BMo8
p = 0.6057 (Student's t-Test)
Values (°deg)
Asymptomatics
Patients
Groups
BMo8
AUC: 0.529
Sensitivity
Specificity
BMo9
Sample
Theoretical
BMo9
p = 0.0143 (Student's t-Test)
Values (°deg)
Asymptomatics
Patients
Groups
BMo9
AUC: 0.677
Sensitivity
Specificity

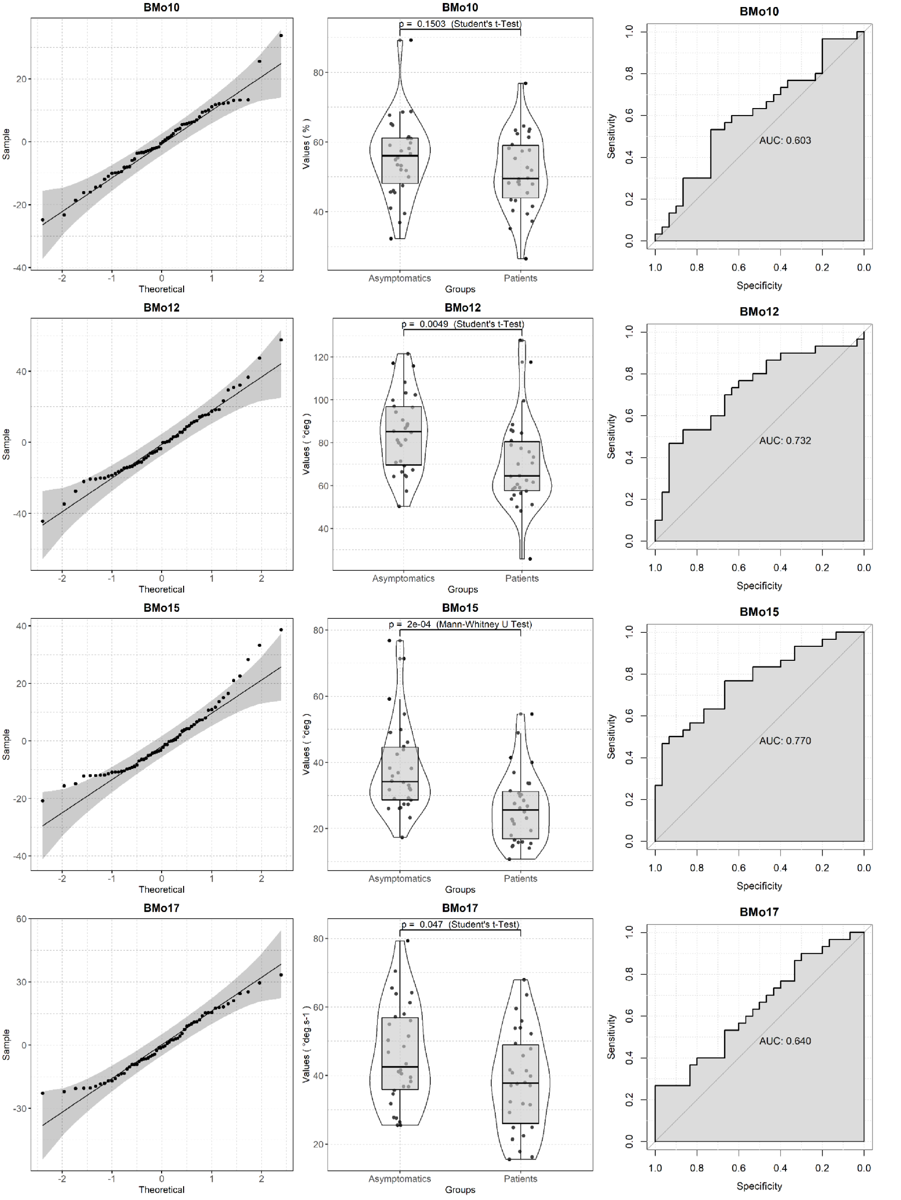

BMo10
Sample
Theoretical
BMo10
p = 0.1503 (Student's t-Test)
Values (%)
Asymptomatics
Patients
Groups
BMo10
AUC: 0.603
Sensitivity
Specificity
BMo12
Sample
Theoretical
BMo12
p = 0.0049 (Student's t-Test)
Values (°deg)
Asymptomatics
Patients
Groups
BMo12
AUC: 0.732
Sensitivity
Specificity
BMo15
Sample
Theoretical
BMo15
p = 2e-04 (Mann-Whitney U Test)
Values (°deg)
Asymptomatics
Patients
Groups
BMo15
AUC: 0.770
Sensitivity
Specificity
BMo17
Sample
Theoretical
BMo17
p = 0.047 (Student's t-Test)
Values (°deg.s-1)
Asymptomatics
Patients
Groups
BMo17
AUC: 0.640
Sensitivity
Specificity

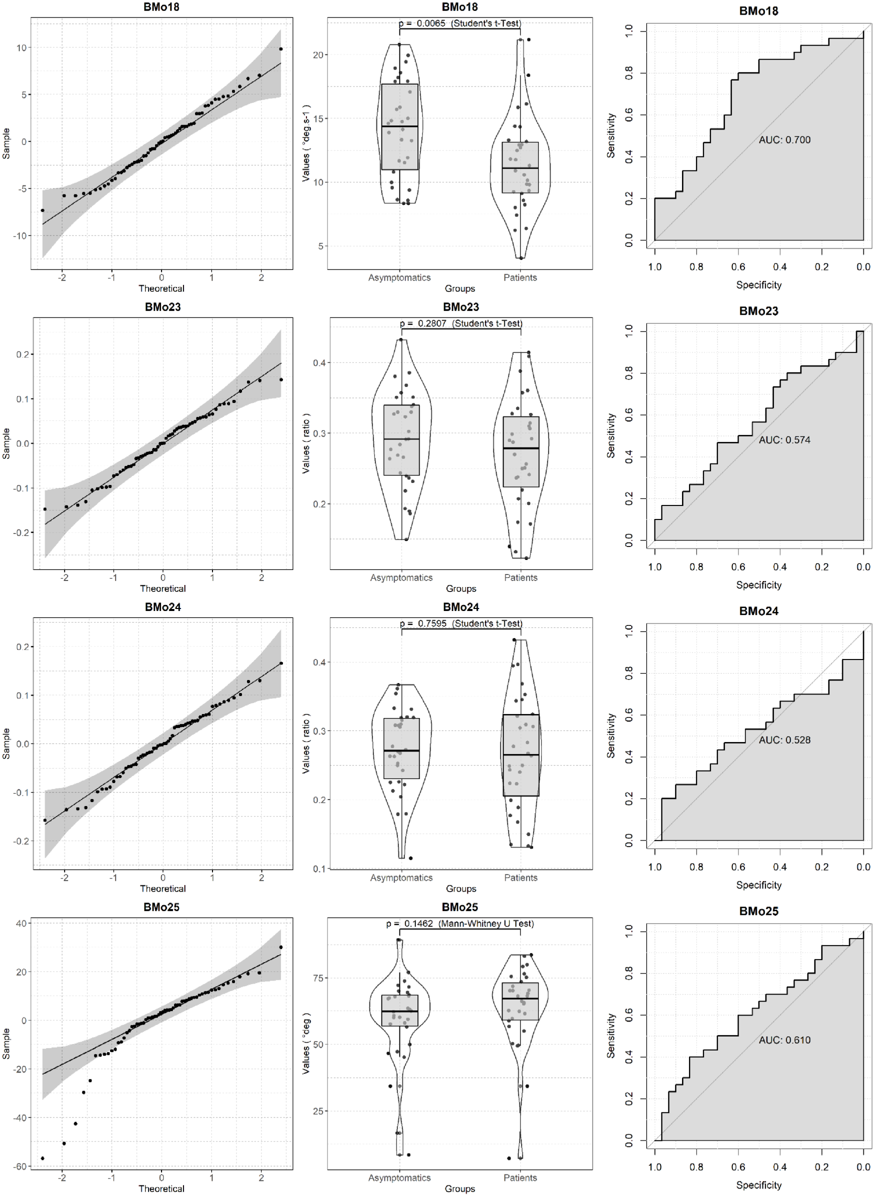
BMo18
Sample
Theoretical
BMo18
p = 0.0065 (Student's t-Test)
Values (°deg.s-1)
Asymptomatics
Patients
Groups
BMo18
Sensitivity
Specificity
AUC: 0.700
BMo23
Sample
Theoretical
BMo23
p = 0.2807 (Student's t-Test)
Values (ratio)
Asymptomatics
Patients
Groups
BMo23
Sensitivity
Specificity
AUC: 0.574
BMo24
Sample
Theoretical
BMo24
p = 0.7595 (Student's t-Test)
Values (ratio)
Asymptomatics
Patients
Groups
BMo24
Sensitivity
Specificity
AUC: 0.528
BMo25
Sample
Theoretical
BMo25
p = 0.1462 (Mann-Whitney U Test)
Values (°deg)
Asymptomatics
Patients
Groups
BMo25
Sensitivity
Specificity
AUC: 0.610

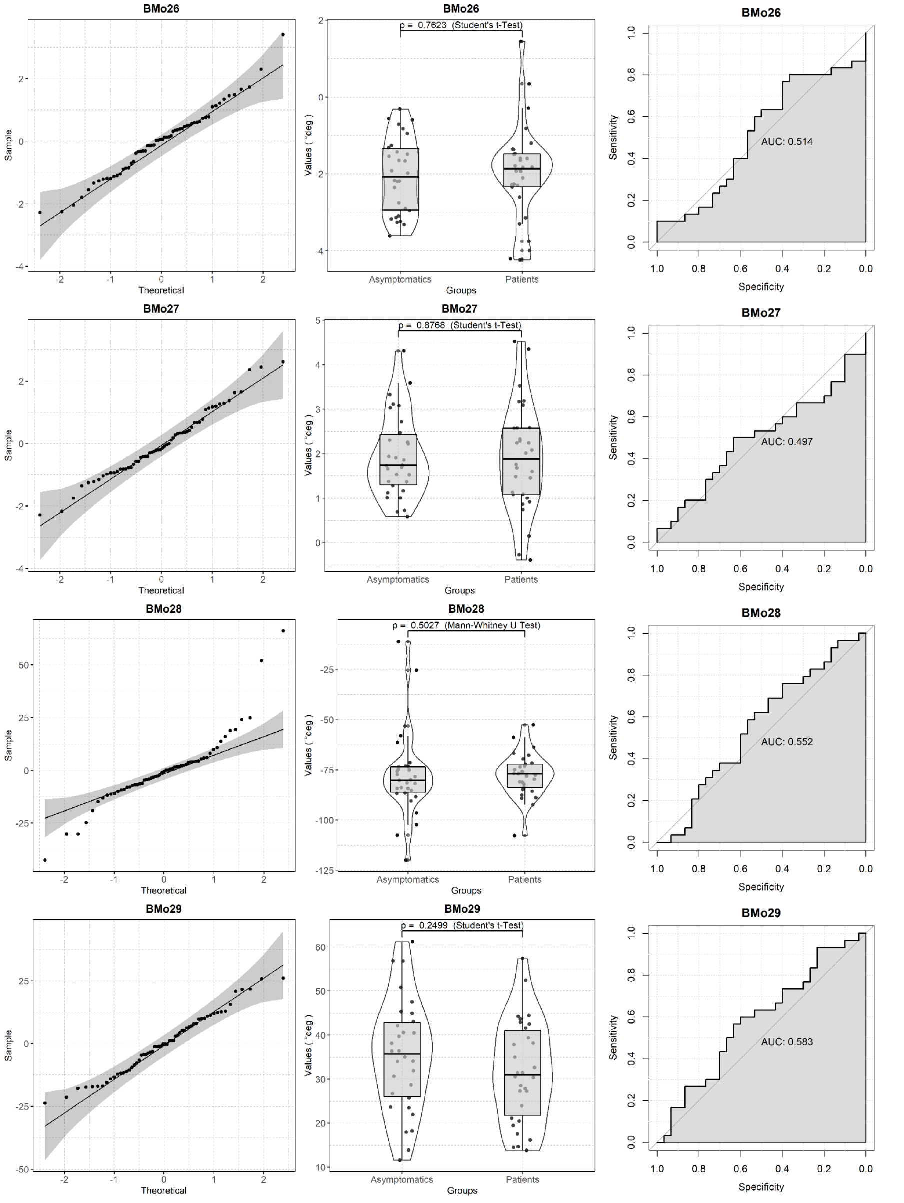
BMo26
Sample
Theoretical
BMo26
p = 0.7623 (Student's t-Test)
Values (°deg)
Asymptomatics
Patients
Groups
BMo26
AUC: 0.514
Sensitivity
Specificity
BMo27
Sample
Theoretical
BMo27
p = 0.8768 (Student's t-Test)
Values (°deg)
Asymptomatics
Patients
Groups
BMo27
AUC: 0.497
Sensitivity
Specificity
BMo28
Sample
Theoretical
BMo28
p = 0.5027 (Mann-Whitney U Test)
Values (°deg)
Asymptomatics
Patients
Groups
BMo28
AUC: 0.552
Sensitivity
Specificity
BMo29
Sample
Theoretical
BMo29
p = 0.2499 (Student's t-Test)
Values (°deg)
Asymptomatics
Patients
Groups
BMo29
AUC: 0.583
Sensitivity
Specificity

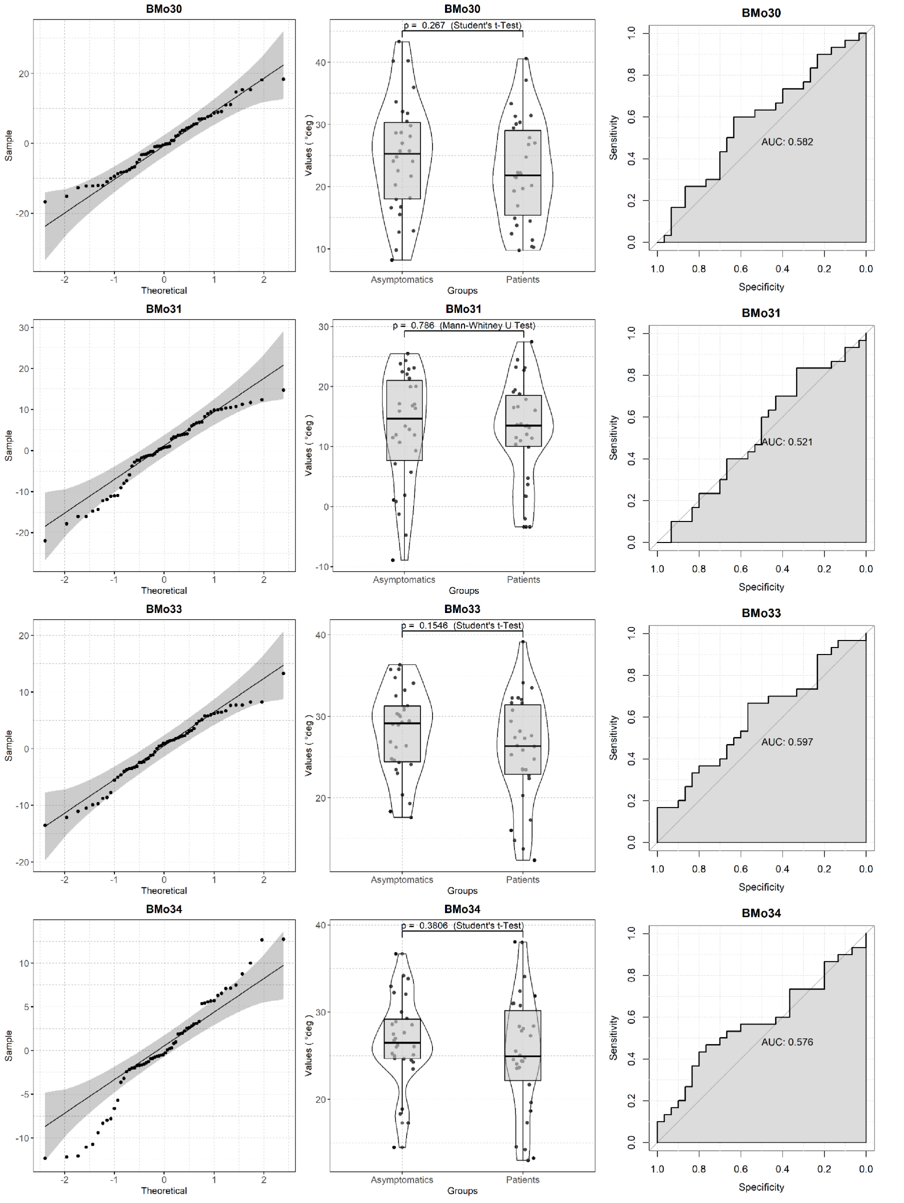
BMo30
Sample
Theoretical
BMo30
p = 0.267 (Student's t-Test)
Values (°deg)
Asymptomatics
Patients
Groups
BMo30
AUC: 0.582
Sensitivity
Specificity
BMo31
Sample
Theoretical
BMo31
p = 0.786 (Mann-Whitney U Test)
Values (°deg)
Asymptomatics
Patients
Groups
BMo31
AUC: 0.521
Sensitivity
Specificity
BMo33
Sample
Theoretical
BMo33
p = 0.1546 (Student's t-Test)
Values (°deg)
Asymptomatics
Patients
Groups
BMo33
AUC: 0.597
Sensitivity
Specificity
BMo34
Sample
Theoretical
BMo34
p = 0.3806 (Student's t-Test)
Values (°deg)
Asymptomatics
Patients
Groups
BMo34
AUC: 0.576
Sensitivity
Specificity

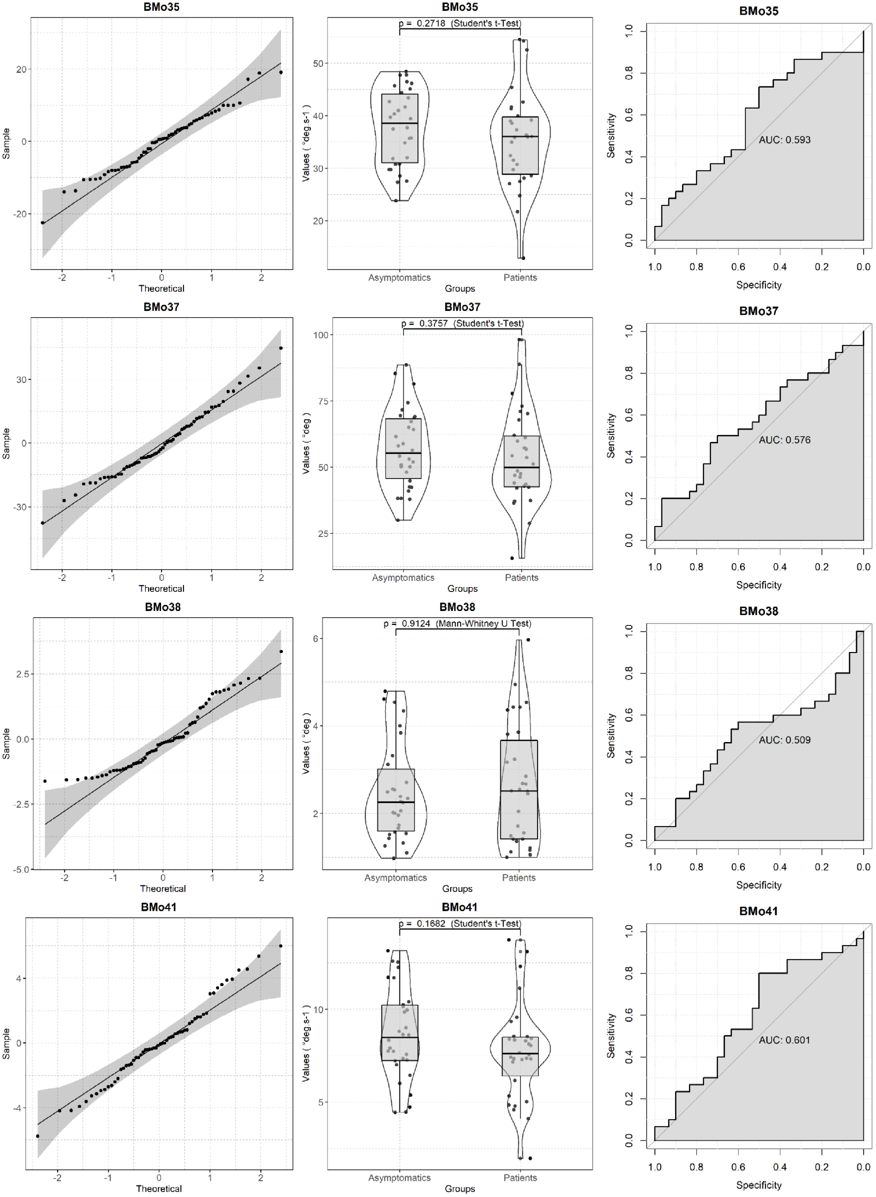
BMo35
Sample
Theoretical
BMo35
p = 0.2718 (Student's t-Test)
Values (°deg.s-1)
Asymptomatics
Patients
Groups
BMo35
AUC: 0.593
Sensitivity
Specificity
BMo37
Sample
Theoretical
BMo37
p = 0.3757 (Student's t-Test)
Values (°deg)
Asymptomatics
Patients
Groups
BMo37
AUC: 0.576
Sensitivity
Specificity
BMo38
Sample
Theoretical
BMo38
p = 0.9124 (Mann-Whitney U Test)
Values (°deg)
Asymptomatics
Patients
Groups
BMo38
AUC: 0.509
Sensitivity
Specificity
BMo41
Sample
Theoretical
BMo41
p = 0.1682 (Student's t-Test)
Values (°deg.s-1)
Asymptomatics
Patients
Groups
BMo41
AUC: 0.601
Sensitivity
Specificity

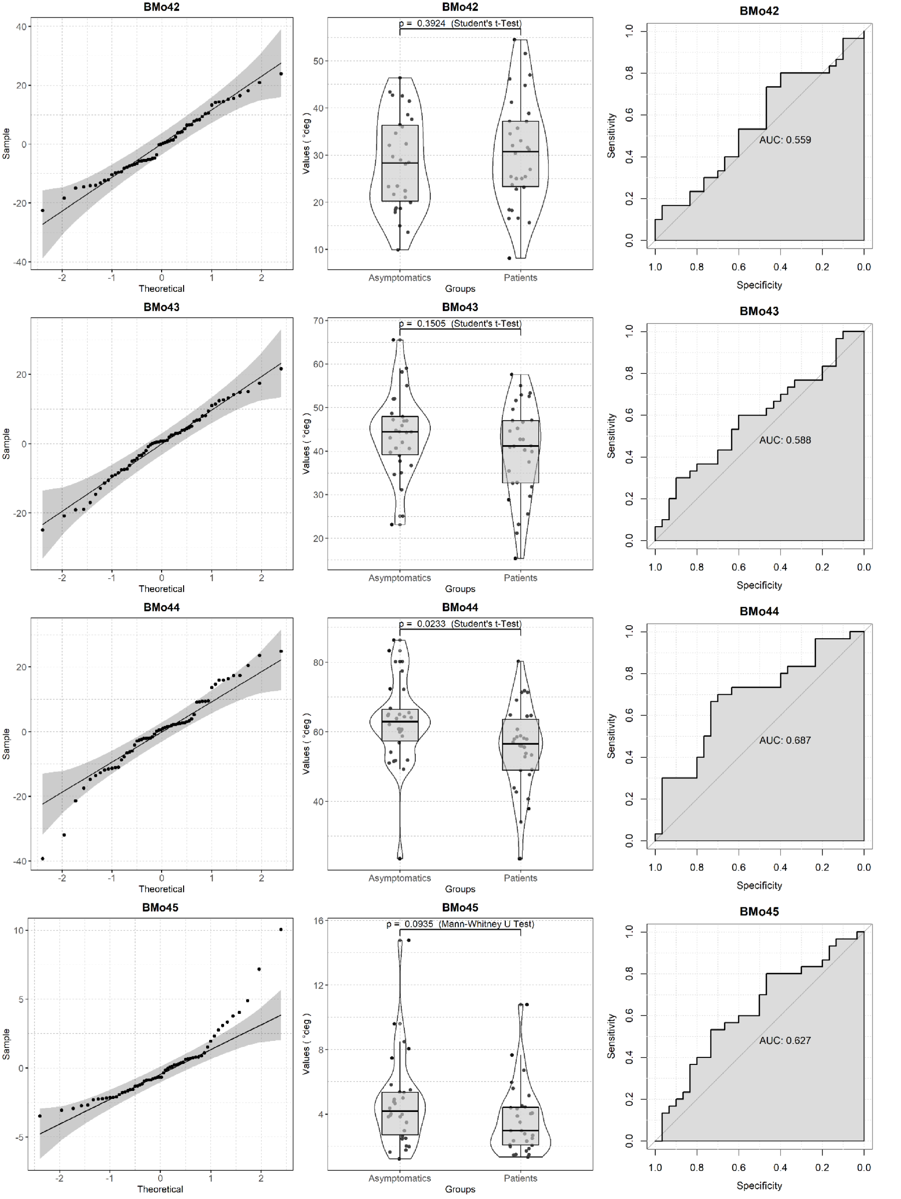

BMo42
Sample
Theoretical
BMo42
p = 0.3924 (Student's t-Test)
Values (°deg)
Asymptomatics
Patients
Groups
BMo42
Sensitivity
Specificity
AUC: 0.559
BMo43
Sample
Theoretical
BMo43
p = 0.1505 (Student's t-Test)
Values (°deg)
Asymptomatics
Patients
Groups
BMo43
Sensitivity
Specificity
AUC: 0.588
BMo44
Sample
Theoretical
BMo44
p = 0.0233 (Student's t-Test)
Values (°deg)
Asymptomatics
Patients
Groups
BMo44
Sensitivity
Specificity
AUC: 0.687
BMo45
Sample
Theoretical
BMo45
p = 0.0935 (Mann-Whitney U Test)
Values (°deg)
Asymptomatics
Patients
Groups
BMo45
Sensitivity
Specificity
AUC: 0.627

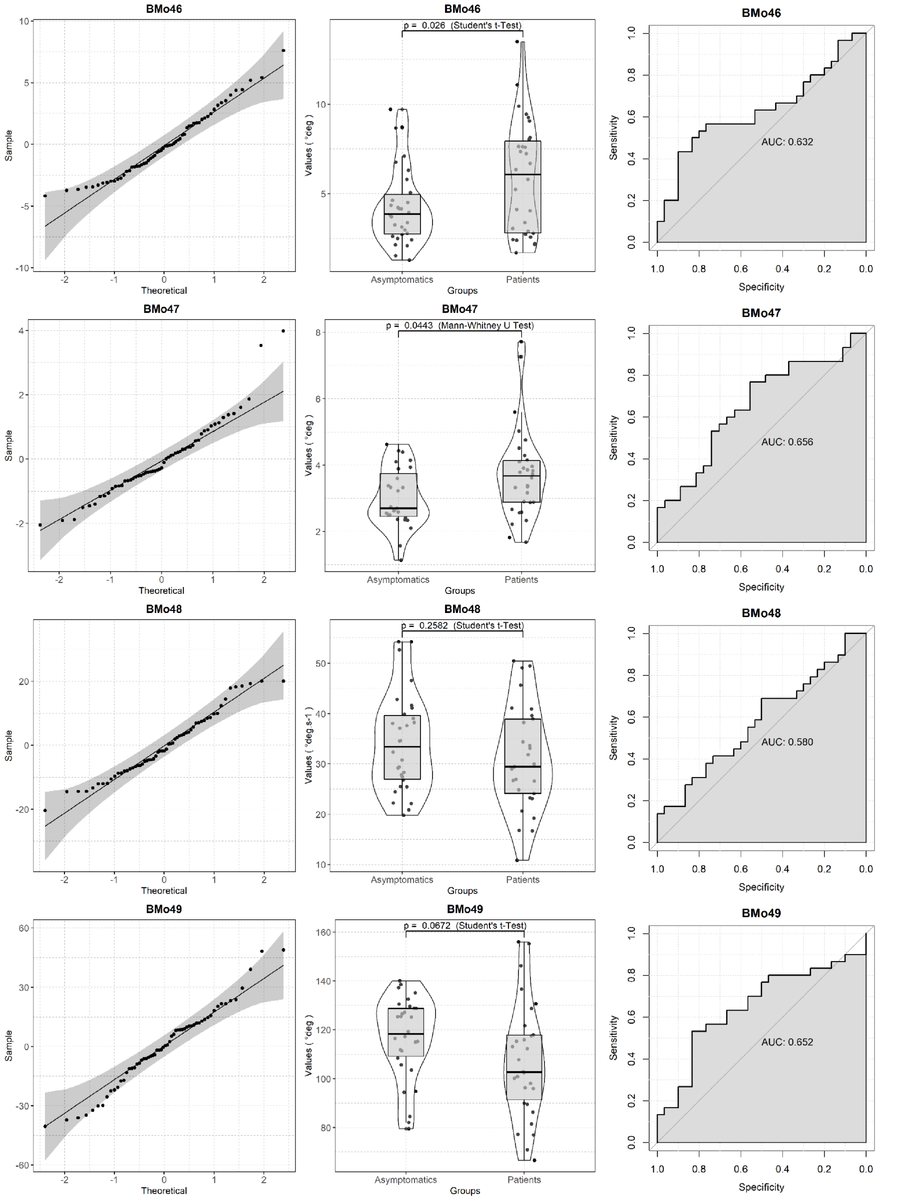

BMo46
Sample
Theoretical
BMo46
p = 0.026 (Student's t-Test)
Values (°deg)
Asymptomatics
Patients
Groups
BMo46
AUC: 0.632
Sensitivity
Specificity
BMo47
Sample
Theoretical
BMo47
p = 0.0443 (Mann-Whitney U Test)
Values (°deg)
Asymptomatics
Patients
Groups
BMo47
AUC: 0.656
Sensitivity
Specificity
BMo48
Sample
Theoretical
BMo48
p = 0.2582 (Student's t-Test)
Values (°deg.s-1)
Asymptomatics
Patients
Groups
BMo48
AUC: 0.580
Sensitivity
Specificity
BMo49
Sample
Theoretical
BMo49
p = 0.0672 (Student's t-Test)
Values (°deg)
Asymptomatics
Patients
Groups
BMo49
AUC: 0.652
Sensitivity
Specificity

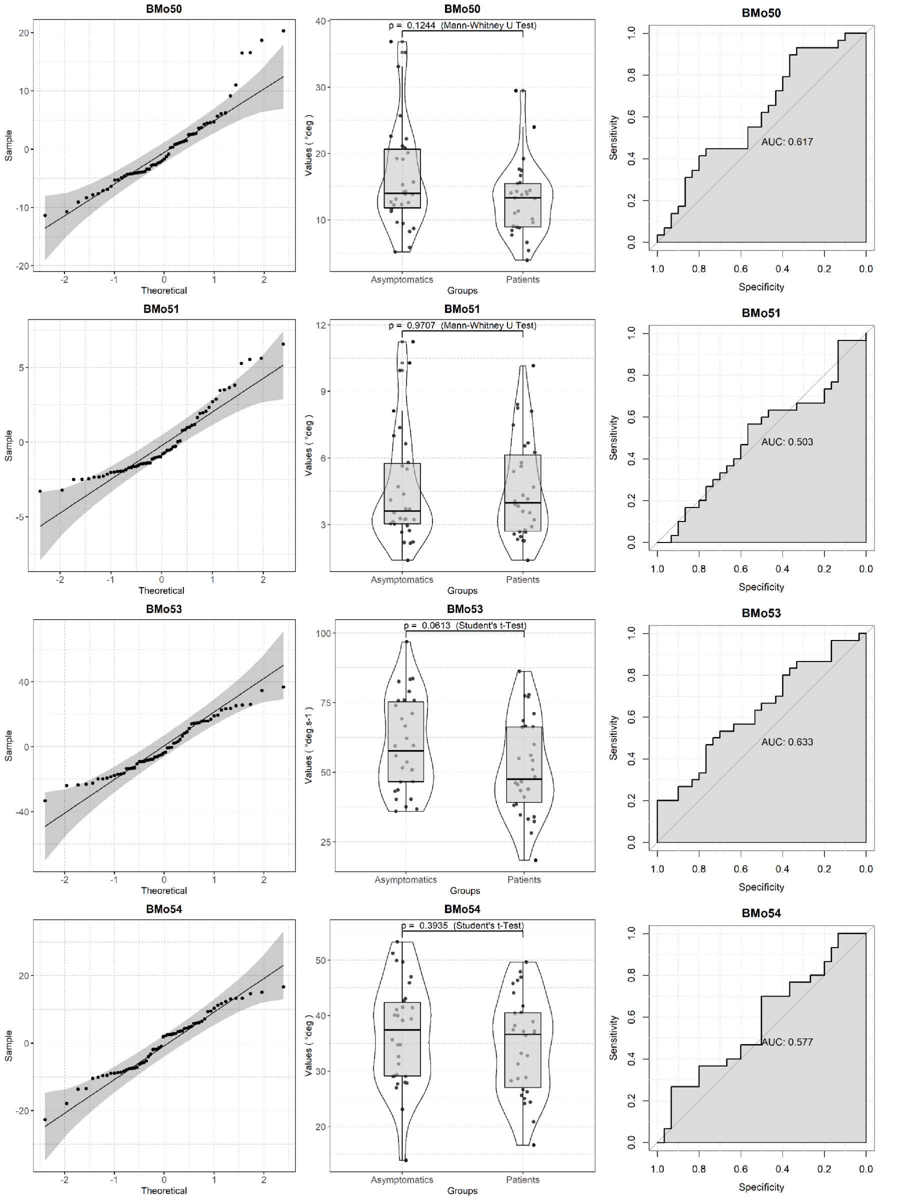
BMo50
Sample
Theoretical
BMo50
p = 0.1244 (Mann-Whitney U Test)
Values (°deg)
Asymptomatics
Patients
Groups
BMo50
AUC: 0.617
Sensitivity
Specificity
BMo51
Sample
Theoretical
BMo51
p = 0.9707 (Mann-Whitney U Test)
Values (°deg)
Asymptomatics
Patients
Groups
BMo51
AUC: 0.503
Sensitivity
Specificity
BMo53
Sample
Theoretical
BMo53
p = 0.0613 (Student's t-Test)
Values (°deg.s-1)
Asymptomatics
Patients
Groups
BMo53
AUC: 0.633
Sensitivity
Specificity
BMo54
Sample
Theoretical
BMo54
p = 0.3935 (Student's t-Test)
Values (°deg)
Asymptomatics
Patients
Groups
BMo54
AUC: 0.577
Sensitivity
Specificity

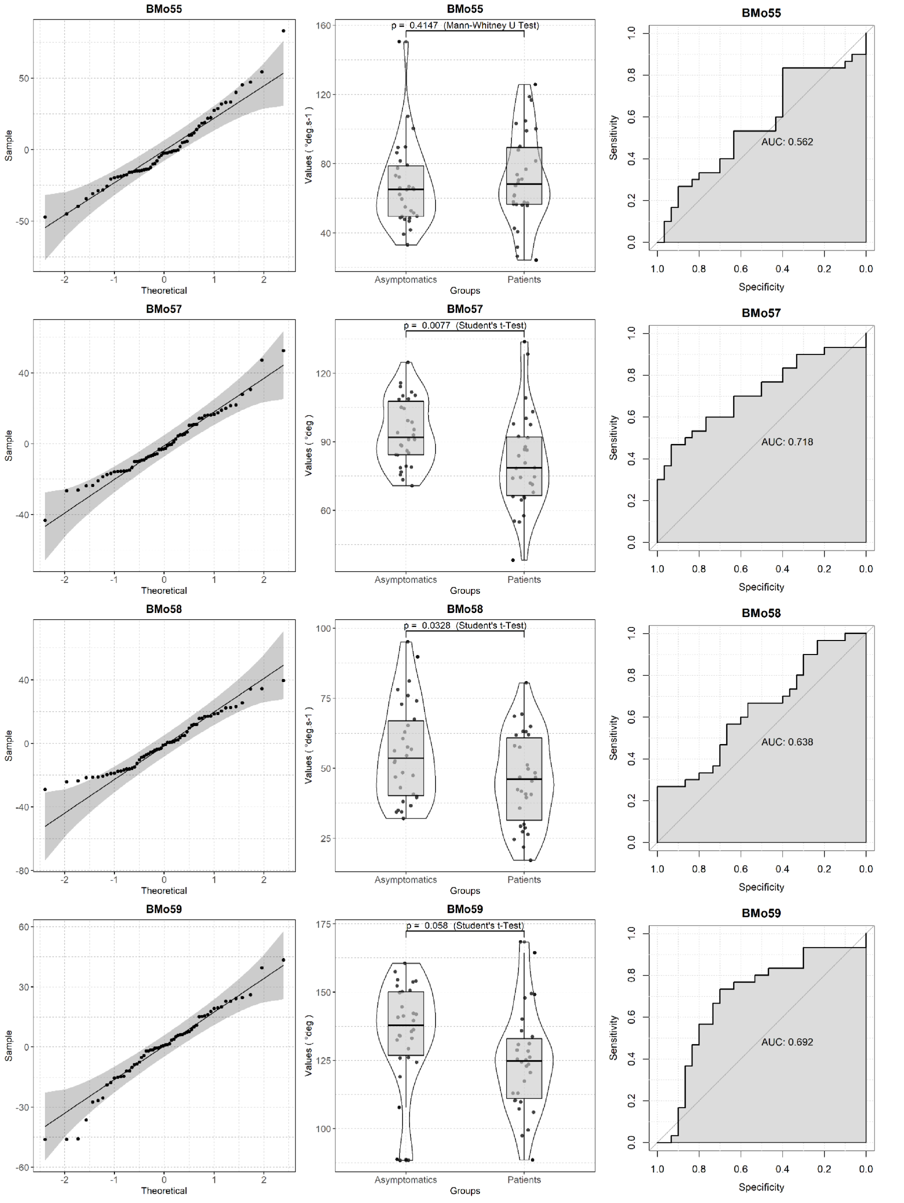
BMo55
Sample
Theoretical
p = 0.4147 (Mann-Whitney U Test)
Values (°deg s-1)
Asymptomatics
Patients
Groups
Sensitivity
Specificity
AUC: 0.562
BMo57
p = 0.0077 (Student's t-Test)
Values (°deg)
AUC: 0.718
BMo58
p = 0.0328 (Student's t-Test)
Values (°deg s-1)
AUC: 0.638
BMo59
p = 0.058 (Student's t-Test)
Values (°deg)
AUC: 0.692

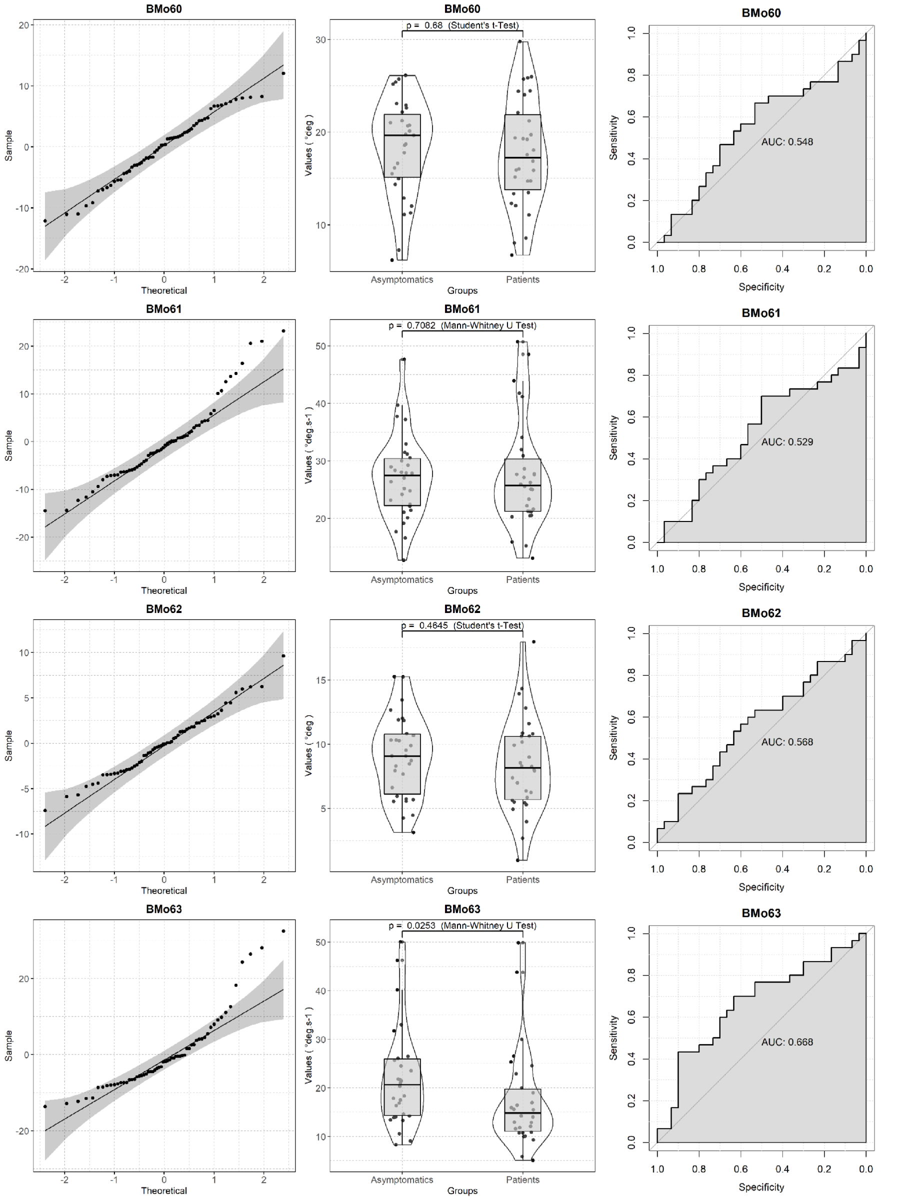

BMo60
Sample
Theoretical
p = 0.68 (Student's t-Test)
Values (°deg)
Asymptomatics
Patients
Groups
Sensitivity
Specificity
AUC: 0.548
BMo61
Sample
Theoretical
p = 0.7082 (Mann-Whitney U Test)
Values (°deg.s-1)
Asymptomatics
Patients
Groups
Sensitivity
Specificity
AUC: 0.529
BMo62
Sample
Theoretical
p = 0.4645 (Student's t-Test)
Values (°deg)
Asymptomatics
Patients
Groups
Sensitivity
Specificity
AUC: 0.568
BMo63
Sample
Theoretical
p = 0.0253 (Mann-Whitney U Test)
Values (°deg.s-1)
Asymptomatics
Patients
Groups
Sensitivity
Specificity
AUC: 0.668

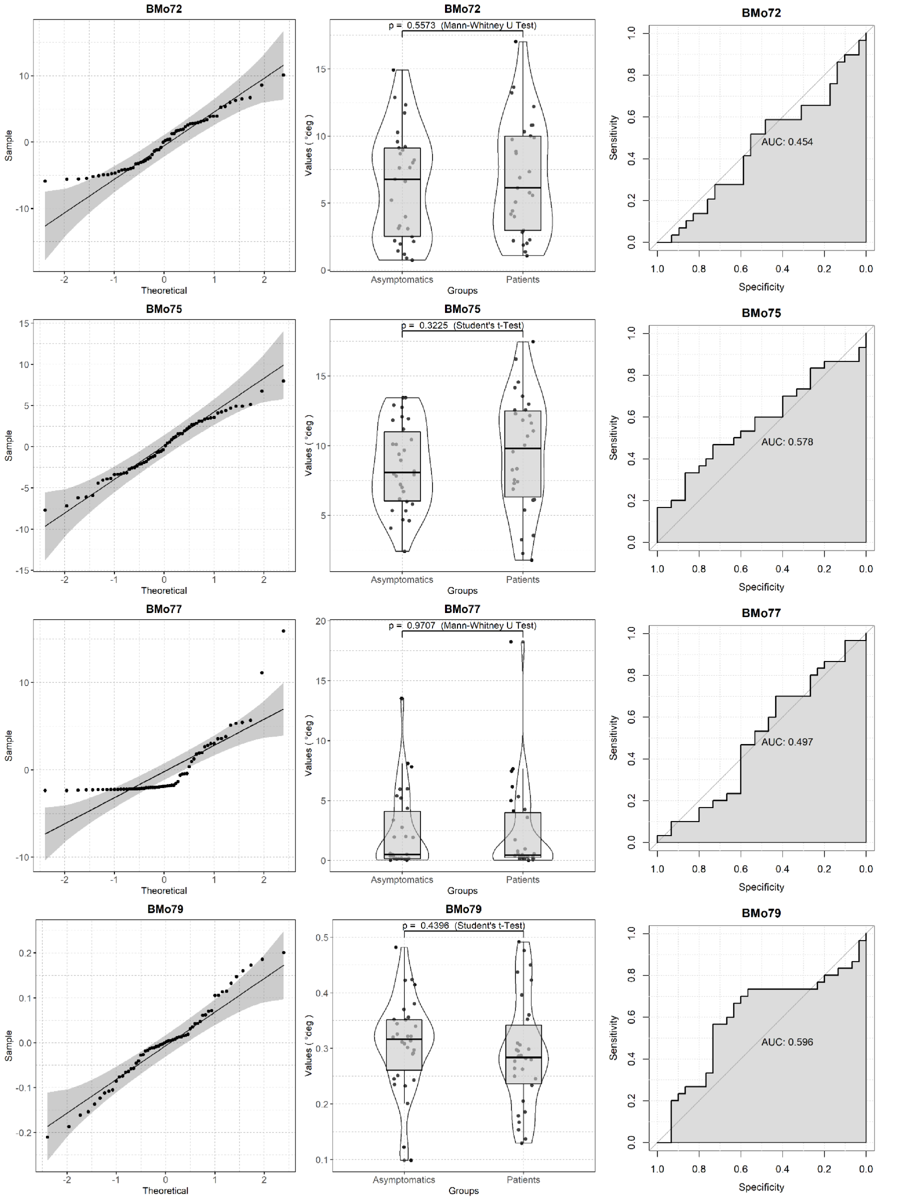
BMo72
Sample
Theoretical
BMo72
p = 0.5573 (Mann-Whitney U Test)
Values (°deg)
Asymptomatics
Patients
Groups
BMo72
AUC: 0.454
Sensitivity
Specificity
BMo75
Sample
Theoretical
BMo75
p = 0.3225 (Student's t-Test)
Values (°deg)
Asymptomatics
Patients
Groups
BMo75
AUC: 0.578
Sensitivity
Specificity
BMo77
Sample
Theoretical
BMo77
p = 0.9707 (Mann-Whitney U Test)
Values (°deg)
Asymptomatics
Patients
Groups
BMo77
AUC: 0.497
Sensitivity
Specificity
BMo79
Sample
Theoretical
BMo79
p = 0.4396 (Student's t-Test)
Values (°deg)
Asymptomatics
Patients
Groups
BMo79
AUC: 0.596
Sensitivity
Specificity

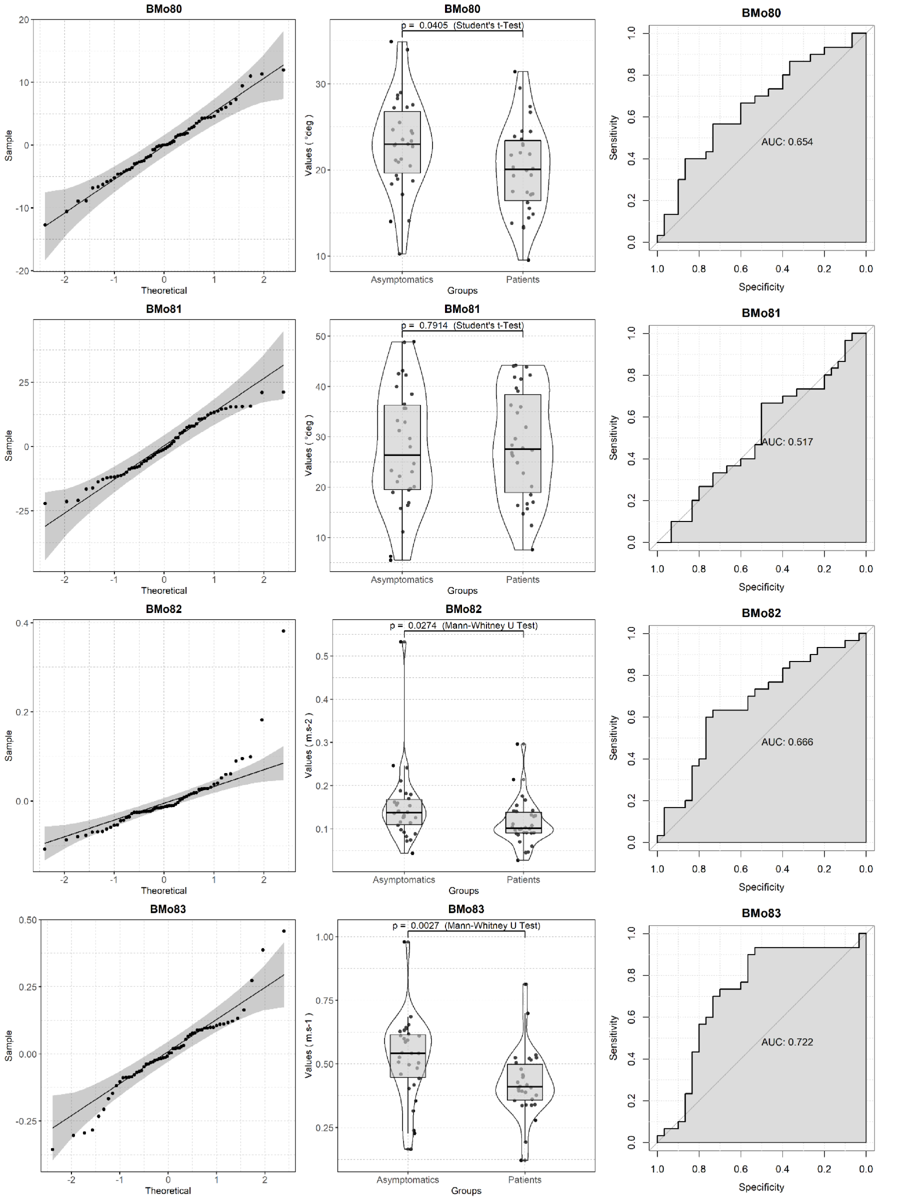

BMo80
Sample
Theoretical
BMo80
p = 0.0405 (Student's t-Test)
Values (°deg)
Asymptomatics
Patients
Groups
BMo80
Sensitivity
Specificity
AUC: 0.654
BMo81
Sample
Theoretical
BMo81
p = 0.7914 (Student's t-Test)
Values (°deg)
Asymptomatics
Patients
Groups
BMo81
Sensitivity
Specificity
AUC: 0.517
BMo82
Sample
Theoretical
BMo82
p = 0.0274 (Mann-Whitney U Test)
Values (m.s-2)
Asymptomatics
Patients
Groups
BMo82
Sensitivity
Specificity
AUC: 0.666
BMo83
Sample
Theoretical
BMo83
p = 0.0027 (Mann-Whitney U Test)
Values (m.s-1)
Asymptomatics
Patients
Groups
BMo83
Sensitivity
Specificity
AUC: 0.722

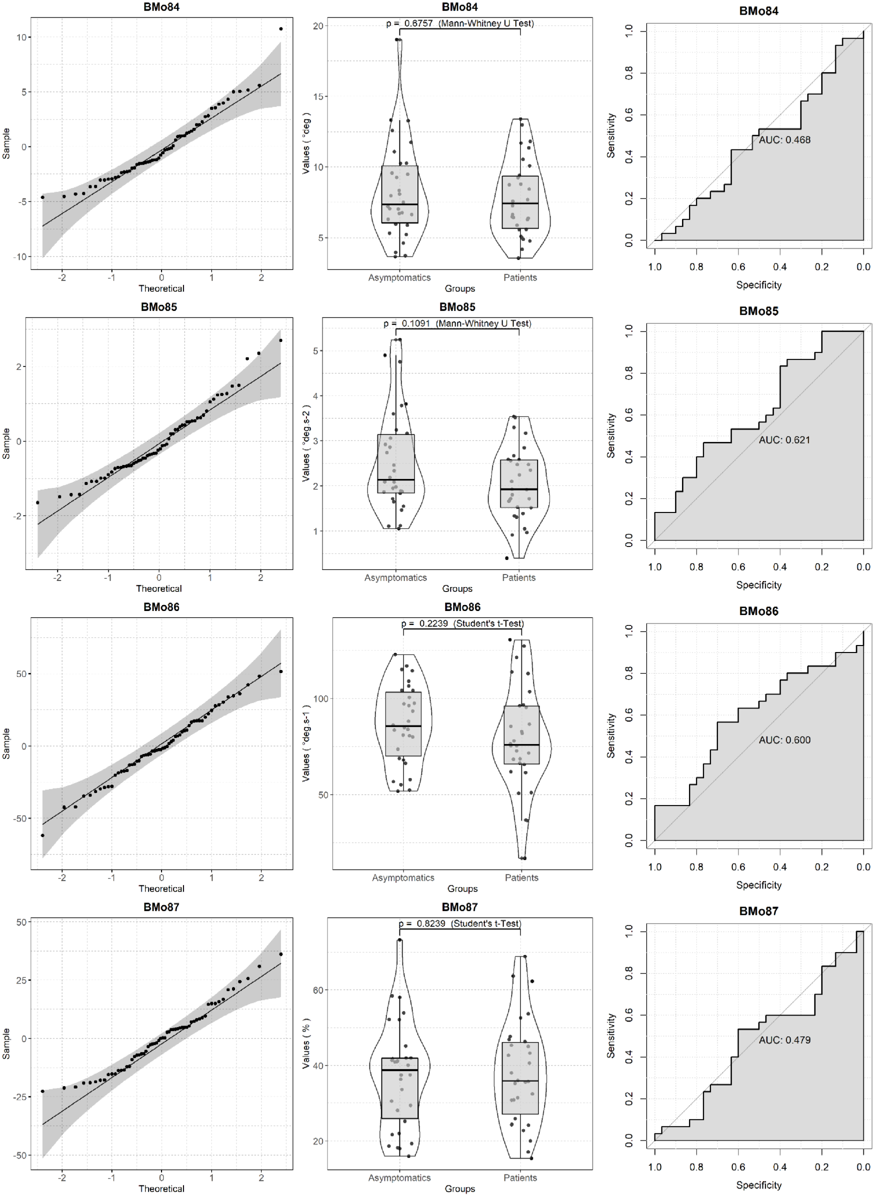

BMo84
Sample
Theoretical
BMo84
p = 0.6757 (Mann-Whitney U Test)
Values (°deg)
Asymptomatics
Patients
Groups
BMo84
AUC: 0.468
Sensitivity
Specificity
BMo85
Sample
Theoretical
BMo85
p = 0.1091 (Mann-Whitney U Test)
Values (°deg.s-2)
Asymptomatics
Patients
Groups
BMo85
AUC: 0.621
Sensitivity
Specificity
BMo86
Sample
Theoretical
BMo86
p = 0.2239 (Student's t-Test)
Values (°deg.s-1)
Asymptomatics
Patients
Groups
BMo86
AUC: 0.600
Sensitivity
Specificity
BMo87
Sample
Theoretical
BMo87
p = 0.8239 (Student's t-Test)
Values (%)
Asymptomatics
Patients
Groups
BMo87
AUC: 0.479
Sensitivity
Specificity

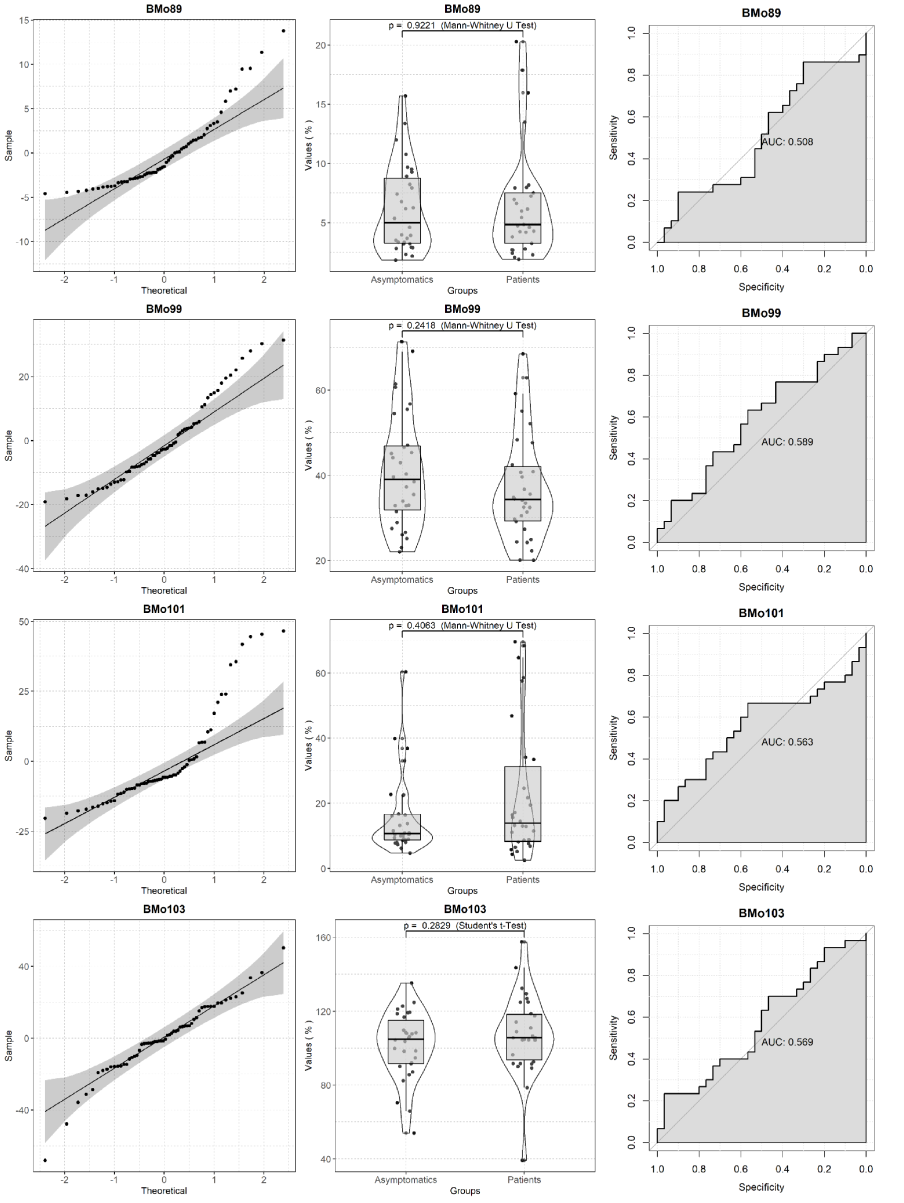

BMo89
Sample
Theoretical
p = 0.9221 (Mann-Whitney U Test)
Values (%)
Asymptomatics
Patients
Groups
Sensitivity
Specificity
AUC: 0.508
BMo99
p = 0.2418 (Mann-Whitney U Test)
AUC: 0.589
BMo101
p = 0.4063 (Mann-Whitney U Test)
AUC: 0.563
BMo103
p = 0.2829 (Student's t-Test)
AUC: 0.569

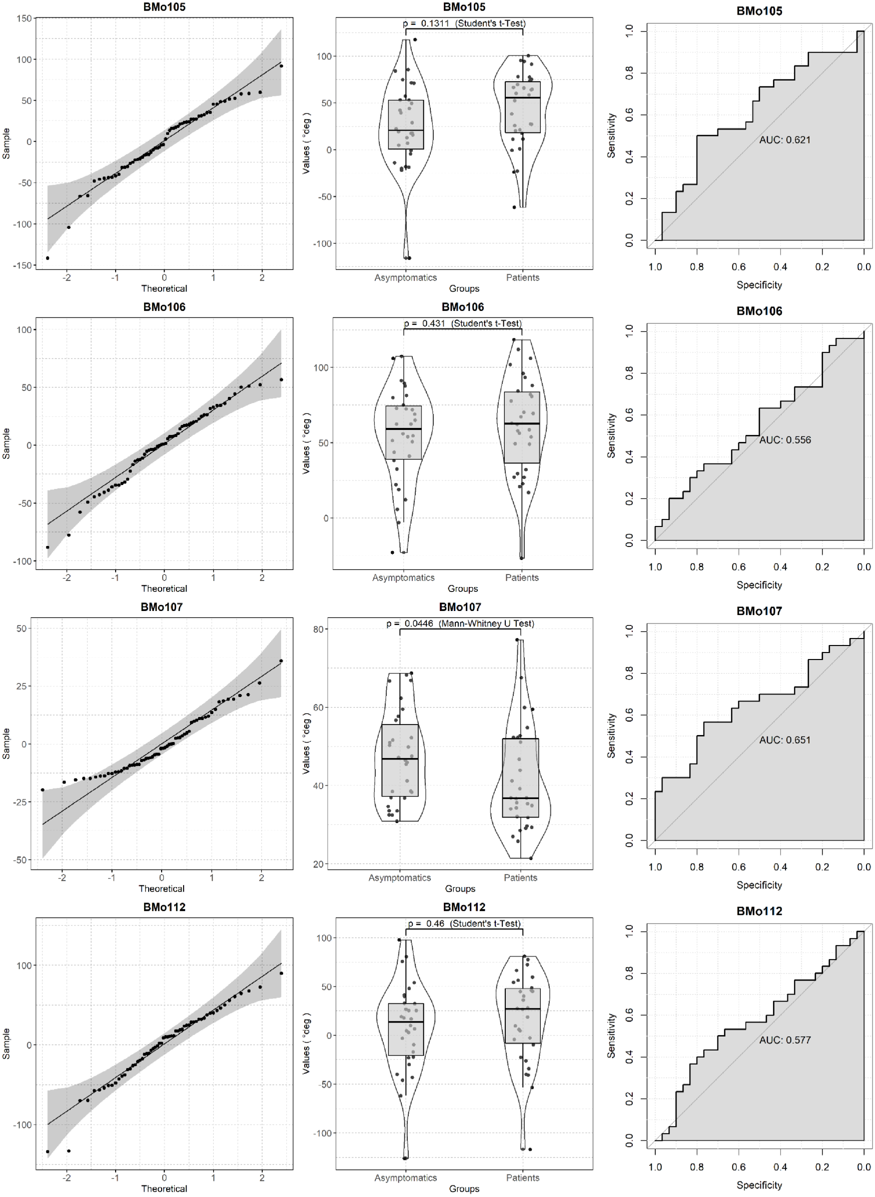
BMo105
Sample
Theoretical
BMo105
p = 0.1311 (Student's t-Test)
Values (°deg)
Asymptomatics
Patients
Groups
BMo105
Sensitivity
Specificity
AUC: 0.621
BMo106
Sample
Theoretical
BMo106
p = 0.431 (Student's t-Test)
Values (°deg)
Asymptomatics
Patients
Groups
BMo106
Sensitivity
Specificity
AUC: 0.556
BMo107
Sample
Theoretical
BMo107
p = 0.0446 (Mann-Whitney U Test)
Values (°deg)
Asymptomatics
Patients
Groups
BMo107
Sensitivity
Specificity
AUC: 0.651
BMo112
Sample
Theoretical
BMo112
p = 0.46 (Student's t-Test)
Values (°deg)
Asymptomatics
Patients
Groups
BMo112
Sensitivity
Specificity
AUC: 0.577

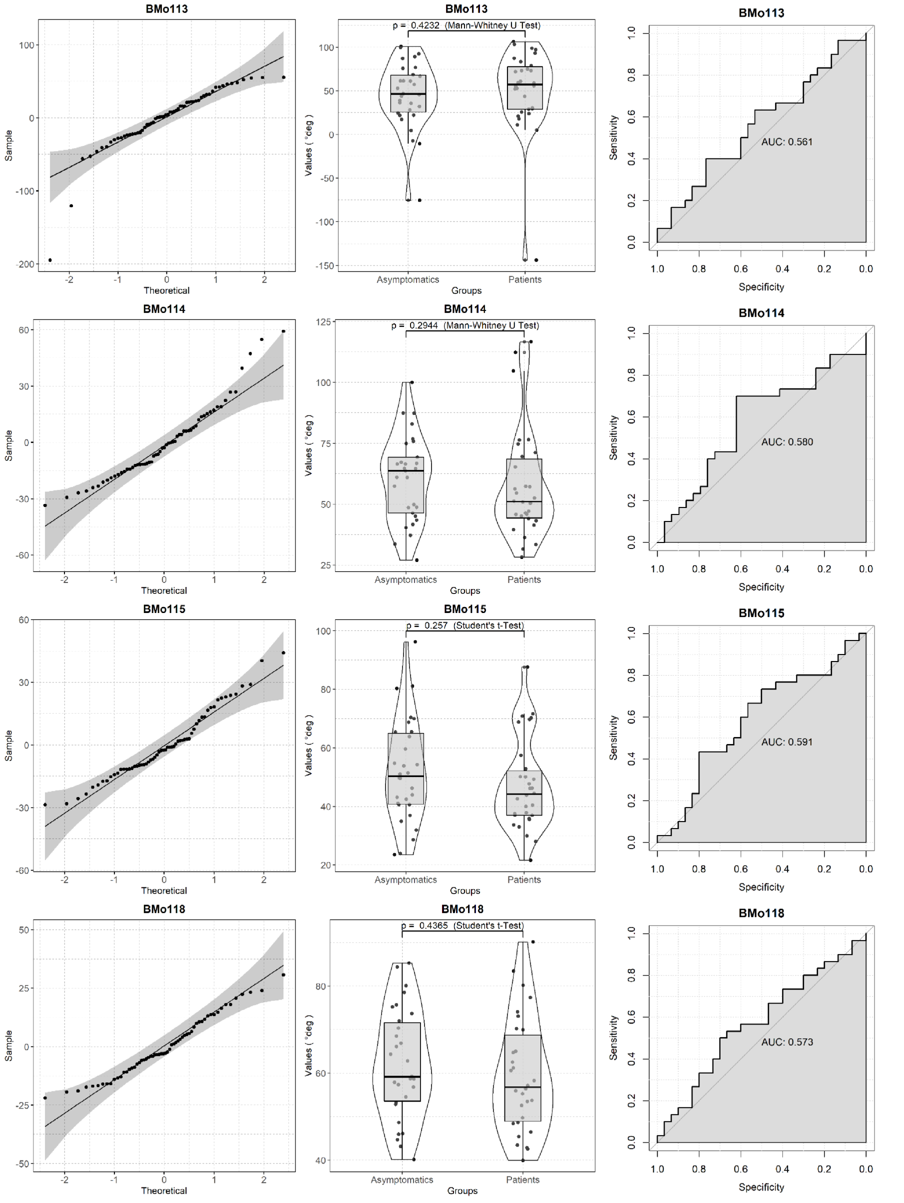
BMo113
Sample
Theoretical
BMo113
p = 0.4232 (Mann-Whitney U Test)
Values (°deg)
Asymptomatics
Patients
Groups
BMo113
Sensitivity
Specificity
AUC: 0.561
BMo114
Sample
Theoretical
BMo114
p = 0.2944 (Mann-Whitney U Test)
Values (°deg)
Asymptomatics
Patients
Groups
BMo114
Sensitivity
Specificity
AUC: 0.580
BMo115
Sample
Theoretical
BMo115
p = 0.257 (Student's t-Test)
Values (°deg)
Asymptomatics
Patients
Groups
BMo115
Sensitivity
Specificity
AUC: 0.591
BMo118
Sample
Theoretical
BMo118
p = 0.4365 (Student's t-Test)
Values (°deg)
Asymptomatics
Patients
Groups
BMo118
Sensitivity
Specificity
AUC: 0.573
